# A Room-Temperature Rh-Catalyzed Kinetic Resolution Pathway for Expedient Access to *P*‑Stereogenic Cyclic Phosphinates

**DOI:** 10.1021/jacsau.6c00283

**Published:** 2026-04-11

**Authors:** Xiaodong Gu, Xin-Yan Ke, Pui Ying Choy, Shao-Fei Ni, Jun Wang, Fuk Yee Kwong

**Affiliations:** † Department of Chemistry, 26679 Hong Kong Baptist University, Kowloon, Hong Kong, China; ‡ Department of Chemistry and Key Laboratory for Preparation and Application of Ordered Structural Materials of Guang-dong Province, 12386 Shantou University, Shantou 515063, China; § Chemistry and Chemical Engineering Guangdong Laboratory, Shantou 515063, China; ∥ Department of Chemistry and State Key Laboratory of Synthetic Chemistry, 26451 The Chinese University of Hong Kong, New Territories, Shatin, Hong Kong, China

**Keywords:** asymmetric catalysis, hydrofunctionalization, kinetic resolution, phosphine, rhodium catalysis

## Abstract

*P*-Stereogenic center-embedded heterocycles are privileged scaffolds in pharmaceuticals and catalysis, yet their synthesis remains a formidable challenge, largely reliant on desymmetrization or asymmetric aromatic C–P cross-coupling. Herein, we report a room temperature Rh-catalyzed enantioselective ring-closing hydrofunctionalization of alkynylphosphinates that directly constructs kinetically favored, nonarene-fused *P*-stereogenic five-membered rings. DFT calculations revealed that the proximal ligation of the phenolic additive plays a critical role in accelerating P–H bond activation, thereby enabling efficient hydrofunctionalization across the alkynyl segment. This method accommodates diverse substituents located at the phosphorus atom and the alkynyl moiety, providing a versatile platform for accessing valuable building blocks for pharmaceutical and chiral ligand design.

## Graphical abstract

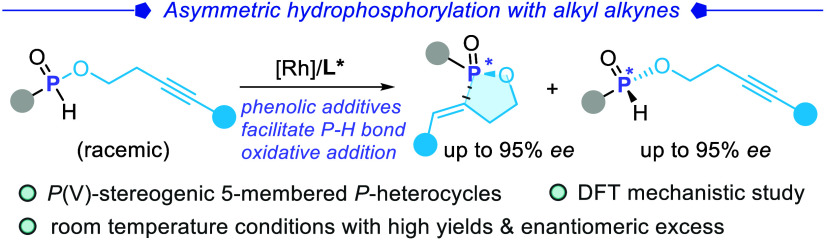

## Introduction

Heterocycles featuring a *P*-stereogenic^1^ center are essential motifs in asymmetric catalysis,^2–7^ medicinal chemistry,^3–13^ and materials science,^14,15^ where their stereochemical configuration dictates biological activity and catalytic performance.^16,17^ Indeed, the synthesis of enantiopure *P*-stereogenic *P*-heterocycles remains challenging. Traditional synthetic strategies rely heavily on chiral auxiliaries,^18,19^ or tedious resolution of racemic compounds,^20–22^ which often involve multistep synthetic schemes and/or stoichiometric amount of chiral reagents, making them less practical to be accessed. Recent developments in constructing *P*-stereocenters are desymmetrization^23–40^ and enantioselective C–P bond cross-coupling^41–57^ (Scheme 1A).^58–61^ Despite established methodologies, five-membered *P*-stereogenic *P*-heterocycles (excluding benzo-fused derivatives)^62–66^ remain less studied than their six-membered or larger ring size counterparts (Scheme 1A,B).^38,67–73^ In fact, there has been a significant demand to develop a general method for building nonfused five-membered ring *P*-stereogenic *P*-heterocycles.

**Scheme 1:**
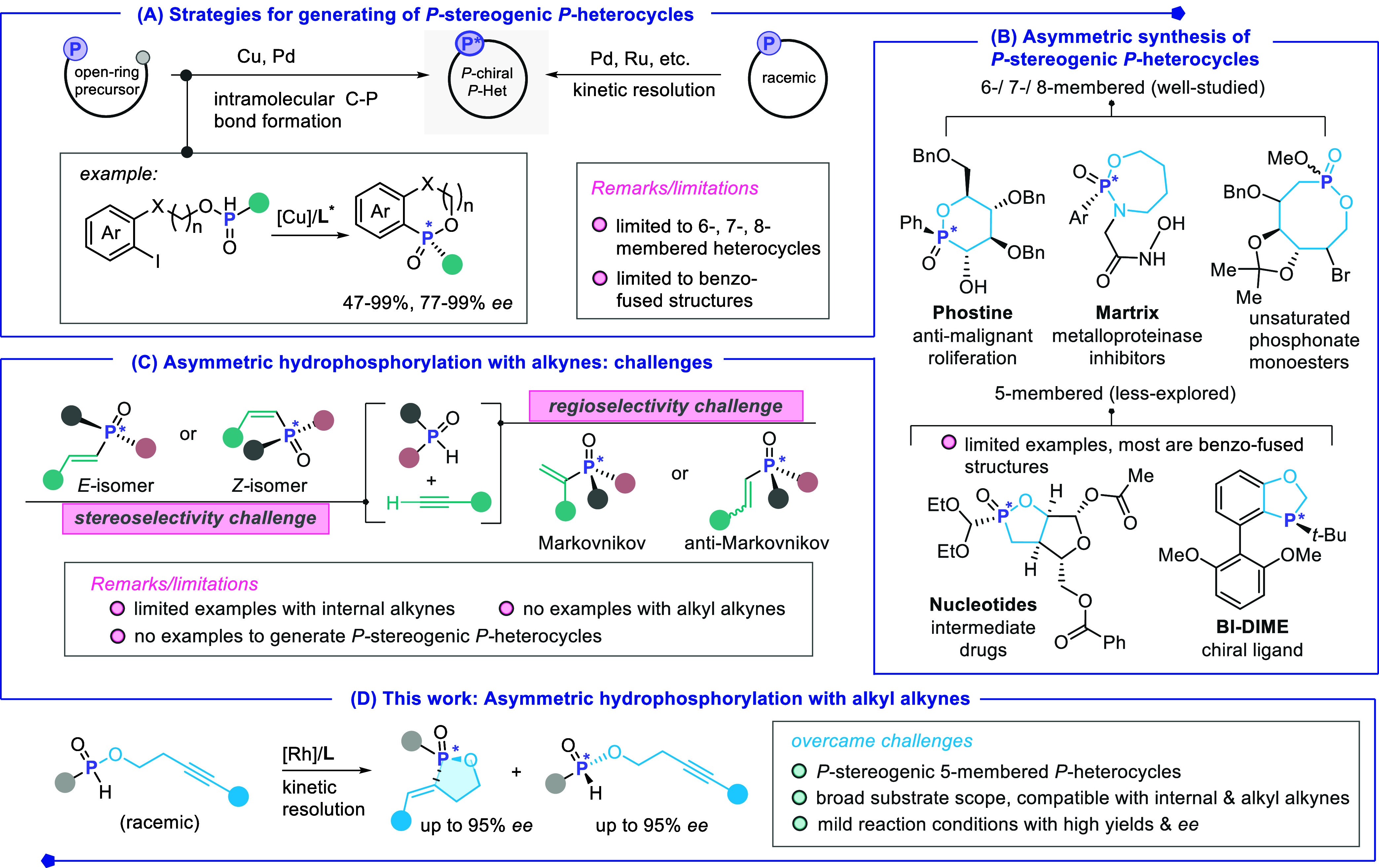
Current Strategies for Accessing *P*-Stereogenic Heterocycles and Our Proposal

Asymmetric hydrofunctionalization of the H–P bond across an alkynyl segment provides a direct and atom-economical pathway to access *P*-stereogenic compounds^74–78^ (Scheme 1C). However, synthetic challenges such as controlling regioselectivity (anti-Markovnikov vs. Markovnikov addition) and stereoselectivity (*E/Z* isomer) remain a concern and hamper its potential applicability.^79^ Moreover, internal alkynes were seldom employed successfully in asymmetric hydrophosphorylation because of their intrinsic low reactivity.^80–84^ To the best of our knowledge, the asymmetric hydrophosphorylation of internal alkyl alkynes, which lack π-system activation, remains refractory to existing catalytic systems for synthesizing *P*-stereogenic *P*-heterocycles. Building on our effort in asymmetric catalysis^85,86^ and prior work in chiral phosphorus chemistry,^87–90^ we herein introduce an expedient Rh-catalyzed kinetic resolution strategy for direct access of cyclic *P*-chiral phosphinates (Scheme 1D). This approach exhibits high product yield and enantiocontrol (up to quantitative yield and 95% *ee*, respectively) for both *P*-stereogenic nonfused five-membered *P*-heterocycles and retained chiral phosphinates.

## Results and Discussion

We initially explored the feasibility of enantioselective Rh-catalyzed intramolecular C–P bond formation by employing oct-3-yn-1-yl phenylphosphinate (**1a**) as the prototypical substrate (Scheme 2). The reaction was found to proceed well using [Rh­(cod)­Cl]_2_ as a precatalyst and diphenyl phosphate (**A1**) as an additive. A series of chiral bisphosphine ligands (**L1**–**L7**) were also evaluated for attaining better product enantioselectivity (Scheme 2, see Supporting Information, Table S3 for details). Attempt using (*R*)-BINAP (**L1**) afforded the desired five-membered *P*-heterocycles **(** ***R*** **)-2a** in 33% yield with 86% *ee*, along with retained **(** ***S*** **)-1a** in 42% yield with 49% *ee*. Screening of other chiral bisphosphine ligands (**L2**-**L7**) delivered **(** ***R*** **)-2a** in 8–49% yields and 60–95% *ee*, accompanied by **(** ***S*** **)-1a** in 42–56% yields and 19–56% *ee* (*S* factor = 6–50). Among these, (*R*)-Cl–MeO-BIPHEP (**L5**) was found to be the most effective ligand, giving an *S* factor of 50 (see Supporting Information, Table S3 for details).

**Scheme 2:**
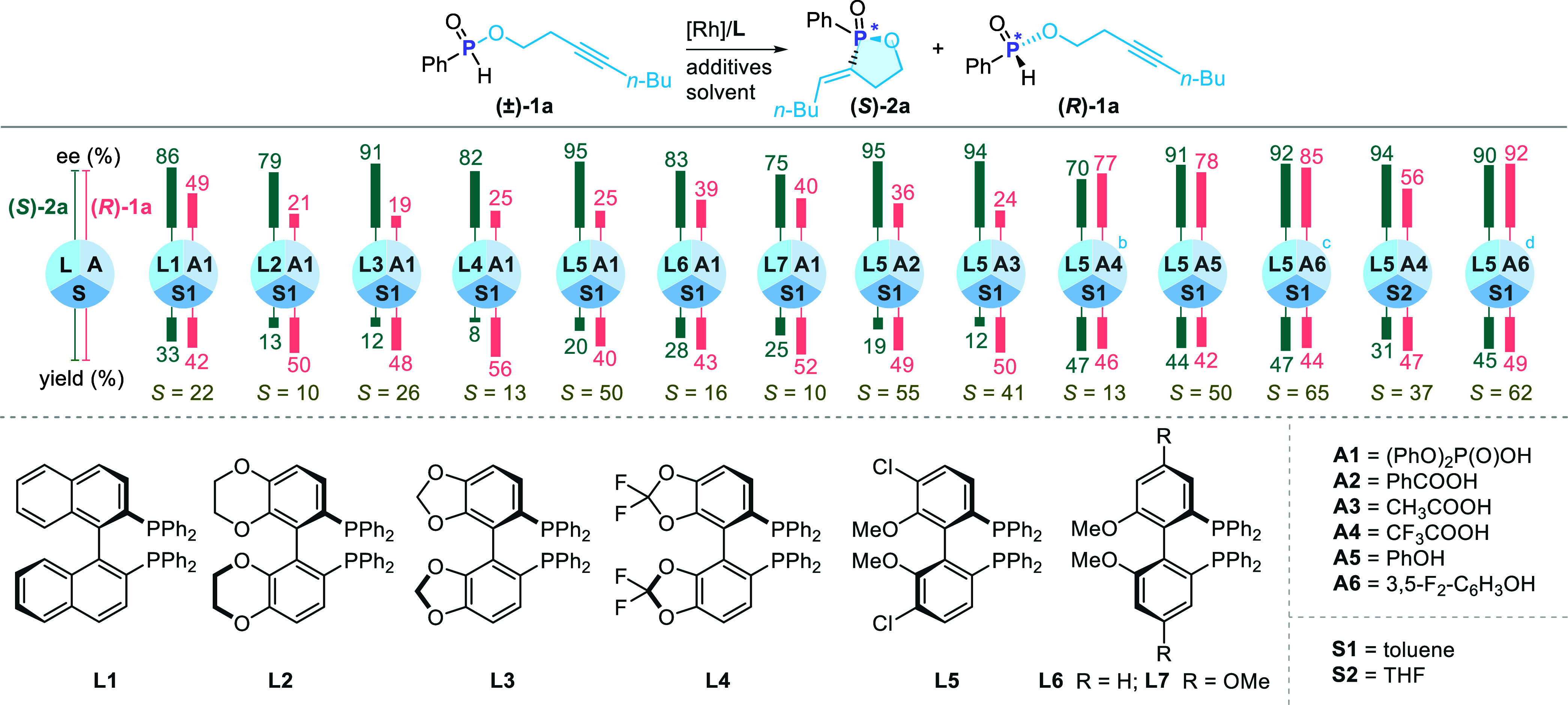
Optimization of Reaction Conditions[Fn s2fn4]

a Reaction conditions: **(±)­1a** (0.10 mmol), [Rh­(cod)­Cl]_2_ (5.0 mol %), ligand **L** (11 mol %), additives **A** (0.10 mmol), and solvent **S** (1.0 mL) were stirred under a nitrogen atmosphere at 25 °C for 20 h. NMR yields were reported using PPh_3_ as internal standard. *S* = Selectivity factor = ln­[(1 – *C*) (1 – *ee* ^SM^)]/ ln­[(1 – *C*) (1 + *ee* ^SM^)]. *C* = Calculated conversion, *C* = *ee* ^SM^/(*ee* ^SM^ + *ee* ^P^).
b The reaction was stirred for 13 h.
c The reaction was stirred for 4 h.
d
**1a** (0.20 mmol), [Rh­(cod)­Cl]_2_ (3.5 mol %), **L5** (7.7 mol %), **A6** (0.10 mmol), and toluene (1.0 mL) were stirred under a nitrogen atmosphere at 25 °C for 8 h.

In addition to the aforementioned parameters, Brønsted acid additives **A1**-**A6** were probed, and **(** ***R*** **)-2a** was afforded in 12–47% yields and 70–95% *ee*. Interestingly, phenol (**A5**) was found to significantly enhance the reaction efficiency while maintaining satisfactory selectivity of **(** ***R*** **)-2a** (44% yield and 91% *ee*). Further optimization revealed that the more acidic difluorophenol **A6** was optimal, furnishing **(** ***R*** **)-2a** in 47% yield with 92% *ee*, and retaining **(** ***S*** **)-1a** in 44% yield with 85% *ee* (*S* factor up to 65). A solvent screening indicated that toluene was in good match with **A6**, showing the best combination probably for achieving enantioselectivity and yields of **(** ***R*** **)-2a**. The *S* factor to 62 was slightly reduced when the catalyst loading was decreased from 5 to 3.5 mol %, with extended reaction time from 4 to 8 h.

With the optimized reaction conditions in hand, we next tested the substrate scope (Scheme 3). In general, 3.5 mol % of the Rh catalyst was found sufficient to promote the conversion of a range of **(±)­1** to the corresponding five-membered cyclic phosphinates **(** ***S*** **)-2** and to retain the enantioenriched alkynylphosphinates **(** ***R*** **)-1** in excellent yields and enantioselectivities (up to 90% *ee* for **(** ***S*** **)-2** and up to 95% *ee* for **(** ***R*** **)-1**). The absolute configuration of **2a** was unambiguously assigned as the *S*-configuration by electronic circular dichroism (ECD) spectroscopy (see Supporting Information for details). The substituents located at the arene ring were well studied. Both electron-rich and electron-deficient substrates were applicable (Scheme 3, products **(** ***S*** **)-2a** to **(** ***S*** **)-2l**). It is worth noting that the bromo and chloro groups remained intact under the stated reaction conditions (products **(** ***S*** **)-2i** to **(** ***S*** **)-2k**), which allow further transformation through the cross-coupling strategy at a later stage.^91–93^ Replacing the aryl group with either a 3-thienyl or a vinyl moiety gave rise to product **(** ***S*** **)-2n** or **(** ***S*** **)-2o** in 89% *ee* or 83% *ee*, respectively. Meanwhile, the modifications of the alkynyl segment of **1** were also examined. Various alkyl-substituted alkynes **1** were found compatible and the product enantioselectivities were essentially not affected (products **(** ***S*** **)-2q** to **(** ***S*** **)-2w**, 85–90% *ee*). Terminal alkynes were also investigated, albeit with a decrease in enantioselectivities (product **(** ***S*** **)-2p**). Aryl alkynes were proven substrates, affording products **(** ***S*** **)-2x** and **(** ***S*** **)-2y** in good yields and enantioselectivities.

**Scheme 3:**
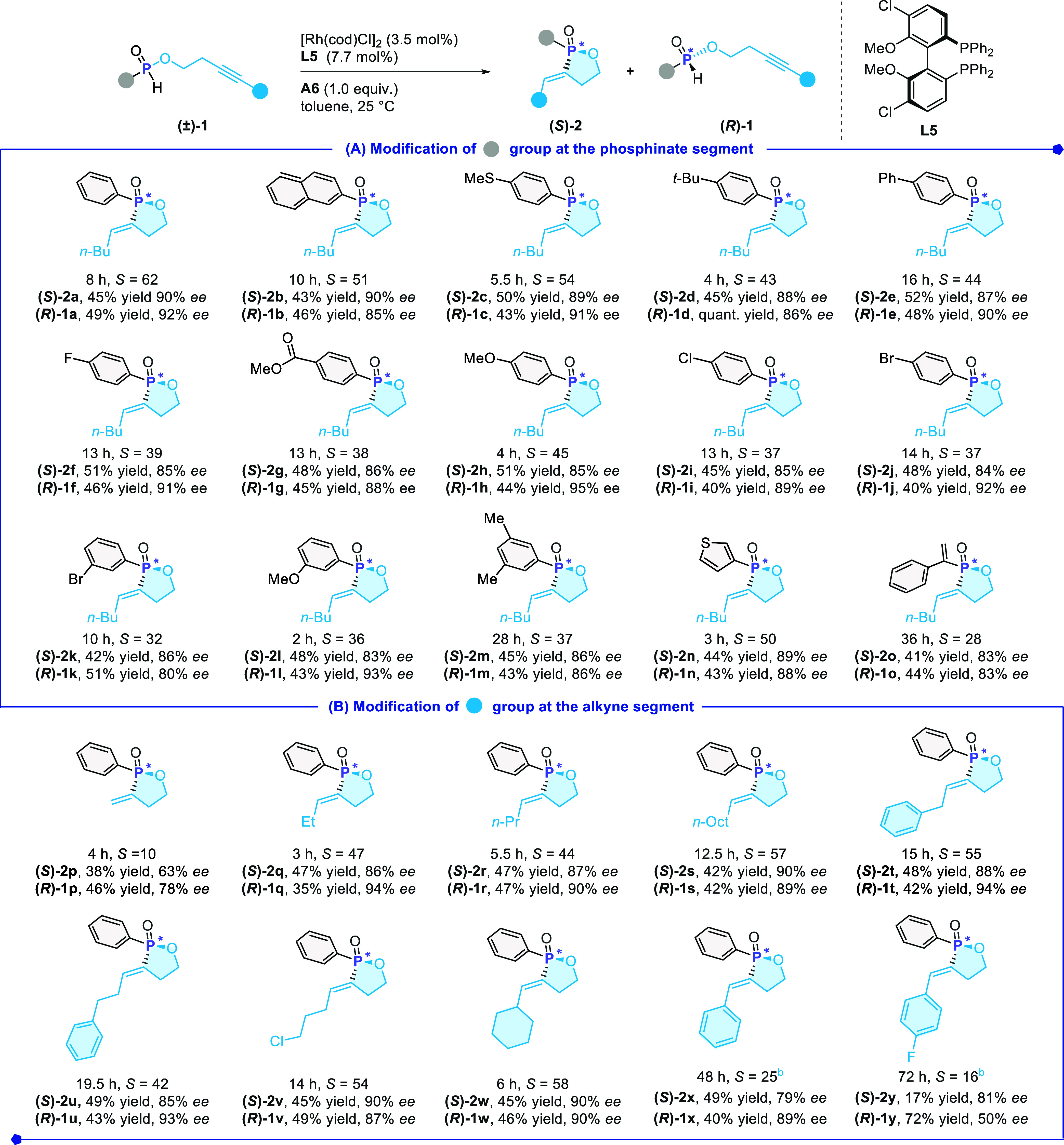
Substrate Scope[Fn s3fn2]

a Reaction conditions: **(±)-1** (0.20 mmol), [Rh­(cod)­Cl]_2_ (3.5 mol %), **L5** (7.7 mol %), **A6** (0.20 mmol), and toluene (2.0 mL) were stirred under a nitrogen atmosphere at 25 °C for stated reaction time, which was determined by monitoring the **(±)-1** conversion using ^31^P NMR. Isolated yields were reported for **(** ***S*** **)-2**. NMR yields were reported for **(** ***R*** **)-1** using PPh_3_ as internal standard.
b
**(±)-1** (0.20 mmol), [Rh­(cod)­Cl]_2_ (5 mol %), **L7** (11 mol %), **A6** (0.20 mmol), and toluene (2.0 mL) were stirred under a nitrogen atmosphere at – 25 °C.

To demonstrate the synthetic potential of the resulting product **2**, a series of further transformations were conducted (Scheme 4A). Treatment of enantioenriched **2j** with methylmagnesium bromide (MeMgBr) yielded tertiary *P*-stereogenic phosphine oxide **3** with retained enantiopurity (82% *ee*). Suzuki-Miyaura cross-coupling and Buchwald-Hartwig amination of **2j** allowed the formation of compounds **4**–**6** in satisfactory yields without erosion of inherent enantiomeric excess. Interestingly, the vinyl group at **2o** successfully underwent 1,4-conjugate hydrophosphination, delivering product **7** in 99% yield with a diastereomeric ratio of 1.6:1, and a full retention of *ee* at the *P*-stereogenic center (Scheme 4B). The absolute configuration of **7b** was also confirmed by an X-ray crystallographic analysis (CCDC 2419354). A time course study of the kinetic resolution of **1a** revealed that **(** ***S)*** **-1a** reacted more rapidly than **(** ***R*** **)-1a** under the standard reaction conditions (Scheme 5).

**Scheme 4:**
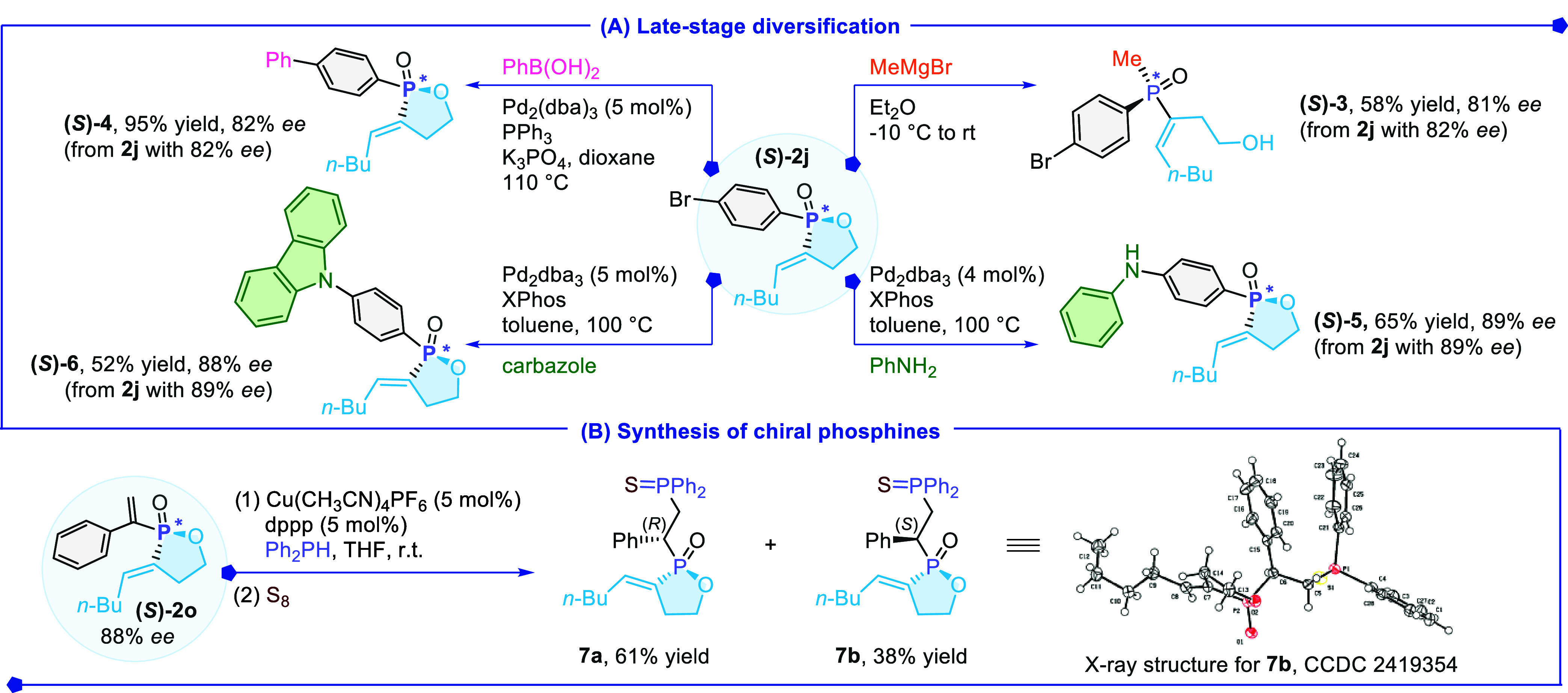
Further Synthetic Transformations

**Scheme 5:**
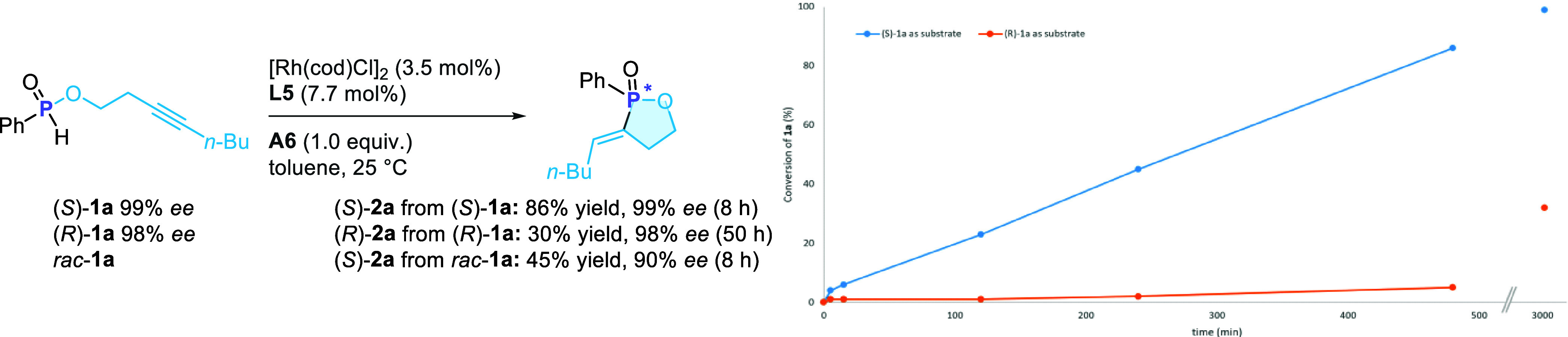
Mechanistic Study: Time Course of Kinetic Resolution of **1a**

Based on our experimental findings and earlier reports,^94–97^ a plausible catalytic cycle is proposed (Scheme 6A). The chiral diphosphine ligand binds to the Rh center in generating complex **I**. ArOH then coordinates to the chiral Rh complex to afford complex **II** (see Supporting Information, Schemes S15, for details). The oxygen atom of ArOH facilitates the oxidative addition of the P–H bond toward Rh center, thus delivering intermediate **III**. Subsequent migratory insertion of the alkyne into the Rh–H bond, in which reductive elimination step serves as stereodiscriminating event. It is worth noting that **(** ***R*** **)-1a** and **(** ***S*** **)-1a** do not interconvert under acidic reaction conditions.

**Scheme 6:**
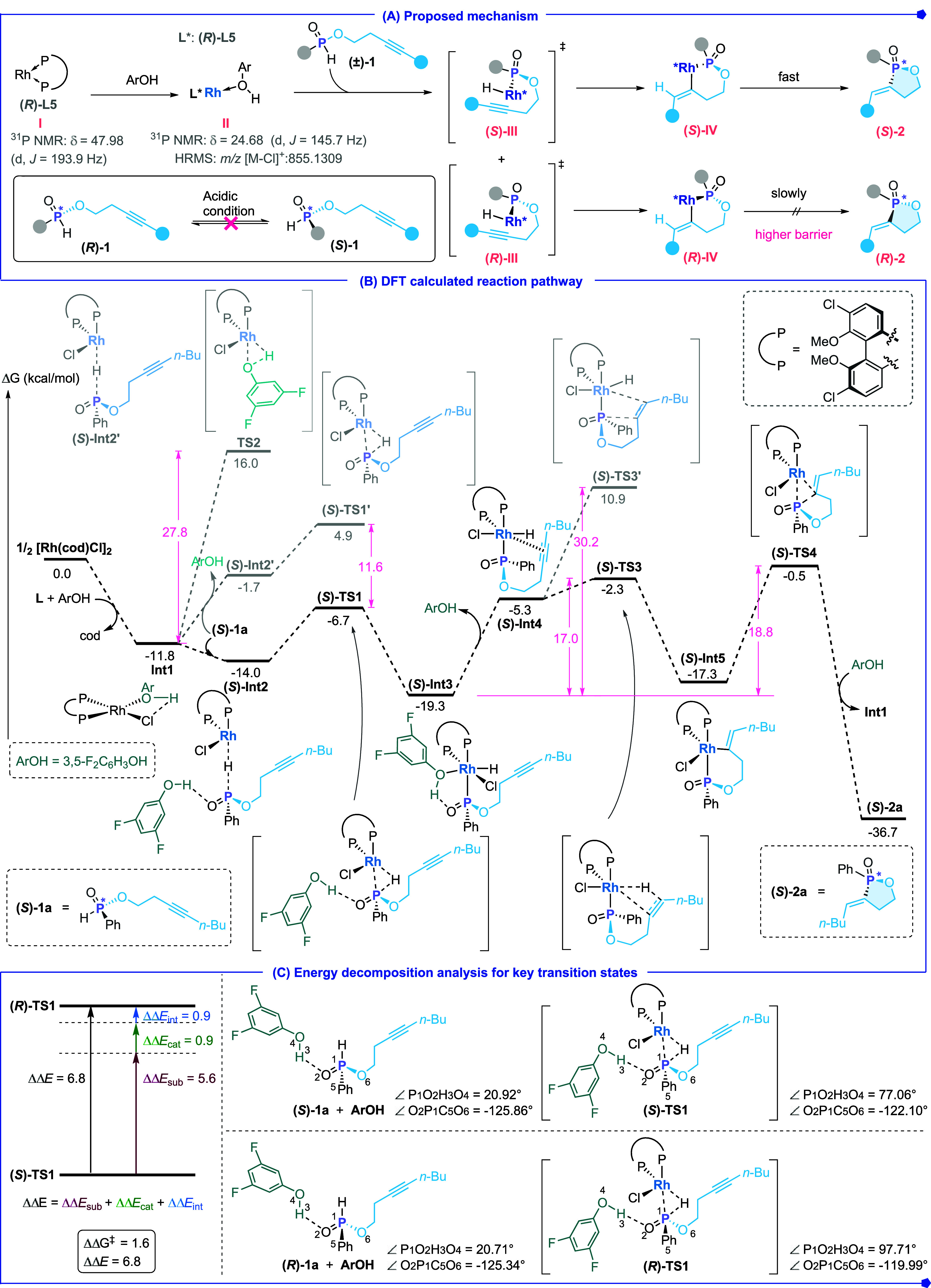
Mechanistic Studies

To gain a deeper understanding of the catalysis mechanism, density functional theory (DFT) calculations were performed at the ωB97X-D/6-311++G**/SDD­(SMD, toluene)//ωB97X-D/6-31G*/LANL2DZ level (Scheme 6B). The catalytic cycle begins with the combination of the Rh precatalyst, ligand **L5**, and acid **A6**, in which this ligation is exergonic (Δ*G* = – 11.8 kcal mol^–1^), and generates the resting state **Int1**. Substrate **(** ***S*** **)-1a** coordination enables the phenolic proton to approach the phosphoryl oxygen within a typical hydrogen-bonding distance. This proximal interaction essentially lowers the energy barrier of P–H oxidative addition from 4.9 kcal mol^–1^ [**(** ***S*** **)-TS1′**, phenol-free pathway] to −6.7 kcal mol^–1^ [**(** ***S*** **)-TS1**, phenol-assisted pathway]. Although, P–H bond cleavage is facile, direct oxidative addition of the phenolic O–H bond to Rh faces an unfavorable activation barrier (**Int1** to **(** ***S*** **)-TS2**, Δ*G* ^‡^ = 27.8 kcal mol^–1^). Next, the alkyne insertion into the Rh–H bond proceeds preferentially, from **(** ***S*** **)-Int3** to **(** ***S*** **)-TS3**, with an energy barrier of Δ*G* ^‡^ = 17.0 kcal mol^–1^. In contrast, competitive insertion into the Rh–P linkage (via **(** ***S*** **)-TS3′**) shows significantly higher energy, Δ*G* ^‡^ = 30.2 kcal mol^–1^. Reductive elimination of [**(** ***S*** **)-TS4**, 18.8 kcal mol^–1^] releases the product **(** ***S*** **)-2a** and finally regenerates **Int1**. Along with the aforementioned computations for **(** ***S*** **)-1a**, an analogous pathway beginning with **(** ***R*** **)-1a** was also investigated (see Scheme S129 for details). The oxidative addition step via **(** ***R*** **)-TS1** has a barrier that is 1.6 kcal mol^–1^ higher than that for **(** ***S*** **)-TS1**. This energy difference rationalizes the extended reaction time observed for **(** ***R*** **)-1a** in Scheme 2 and is consistent with the experimentally measured 90% *ee*. Energy-decomposition analysis (Scheme 6C) reveals an intrinsic gas-phase energy gap of 6.8 kcal mol^–1^ between **(** ***S*** **)-TS1** and **(** ***R*** **)-TS1**. Inclusion of the solvent effect reduces this gap by 5.2 kcal mol^–1^, underscoring the influence of the reaction medium. The decomposition further demonstrates that the positive value of substrate distortion (ΔΔEsub = 5.6 kcal mol^–1^) is the dominant contributor, as **(** ***R*** **)-TS1** undergoes greater structural deformation than **(** ***S*** **)-TS1**. Geometric analysis shows that the degree of pyramidalization at the phosphorus center during P–H bond activation critically influences the steric demands on the chiral rhodium catalyst (Scheme 6C). A comparative analysis of the dihedral angles reveals that the (*R*)-transition state is subjected to a geometric distortion more significant than that of the (*S*)-transition state. Specifically, the torsional angle changes for **(** ***R*** **)-TS1** (∠P1O2H3O4:20.71° to 97.71°; ∠O2P1O6C5: −125.34° to −119.99°) are more pronounced than those for **(** ***S*** **)-TS1** (∠P1O2H3O4: −20.92° to 77.06°; ∠O2P1C5O6: −125.86° to −122.10°), thereby providing a rationale for the observed enantioselectivity favoring the (*S*)-configured product outcome.

## Conclusion

In conclusion, while heterocyclic compounds featuring *P*-stereogenic centers are prevalent in pharmaceuticals and enantioselective catalysis, synthetic access to them remains uneven. Protocols for five-membered *P*-stereogenic heterocycles are notably underdeveloped compared to those of their six- and seven-membered analogs. In particular, the synthesis of nonarene-fused, five-membered *P*-stereogenic heterocycles has remained an unmet challenge. To address this long-standing gap, we developed a chiral diphosphine-rhodium-phenol catalyst system for the asymmetric intramolecular hydrofunctionalization of alkynes at room temperature. This methodology provides direct access to a variety of cyclic phosphinates in good-to-excellent yields with high enantioselectivity (up to 95% ee) and broad functional group tolerance. Notably, halide substituents such as Br and Cl remain intact, enabling further diversification via cross-coupling reactions. DFT calculations revealed that the phenolic additive facilitates the reaction through hydrogen bonding, guiding efficient P–H bond oxidative addition to the Rh center. The subsequent alkynyl insertion proceeds preferentially via the Rh–H bond, with reductive elimination being the enantioselectivity-determining step. Ultimately, this mild and versatile method accommodates diverse substituents on both the phosphorus atom and the alkyne moiety, offering a robust platform for constructing chiral building blocks for pharmaceutical design and chiral ligand development.

## Supplementary Material



## Notes

Experimental procedures, spectral data, and NMR spectra (PDF)
