# Supplementary material for: A Room-Temperature Rh-Catalyzed Kinetic Resolution Pathway for Expedient Access to P‑Stereogenic Cyclic Phosphinates
Source: JACS Au. 2026 Apr 11;6(4):2618–26. doi: 10.1021/jacsau.6c00283 (PMC13126207; doi:10.1021/jacsau.6c00283)
Supplement: Supplementary file 1 [file au6c00283_si_001.pdf]

Supporting Information

A Room Temperature Rh-Catalyzed Kinetic Resolution Pathway for Expedient Access to P-Stereogenic Cyclic Phosphinates

Xiaodong Gu,[‡a,d] Xin-Yan Ke,[‡b] Pui Ying Choy,[d] Shao-Fei Ni,*[b,c] Jun (Joelle) Wang,*[a] and Fuk Yee Kwong*[d]

[a]Department of Chemistry, Hong Kong Baptist University, Kowloon, Hong Kong, China; Email: junwang@hkbu.edu.hk

[b]Department of Chemistry and Key Laboratory for Preparation and Application of Ordered Structural Materials of Guang-dong Province, Shantou University, Shantou, 515063, China; Email: sfni@stu.edu.cn

[c]Chemistry and Chemical Engineering Guangdong Laboratory, Shantou 515063, China

[d]Department of Chemistry and State Key Laboratory of Synthetic Chemistry, The Chinese University of Hong Kong, New Territories, Shatin, Hong Kong, China; Email: fykwong@cuhk.edu.hk

[‡]These authors contributed equally to this work.

Table of Contents

1. General information S1
2. Synthesis of substrates S2
   2.1. Procedures for the synthesis of alcohols (**S1-S11**) S2
   2.2. Procedures for the synthesis of arylphosphinic acid (**S12-S24**) S3
   2.3. Procedures for the synthesis of phosphinic acid (**S25**) S3
   2.4. Procedures for the synthesis of arylphosphinate (**1a-1y**) S4
3. Characterization of substrates S6
4. Optimization of reaction conditions S20
5. Synthesis of *P*-stereogenic cyclic phosphinates S23
6. Characterization of products S25
7. Mechanistic studies S44
8. X-Ray crystallographic analysis S51
9. ^{1}H, ^{13}C, ^{19}F and ^{31}P NMR spectra for compounds S52
10. HPLC spectrum S162
11. Calculation Data S217
12. Quantum chemical ECD calculation method S346
13. References S356

1. General information

NMR Spectra were recorded on a Bruker DPX-600 (or 400) spectrometer at 600 MHz or 400 MHz for ^{1}H NMR, 376 MHz or 565 MHz for ^{19}F NMR,162 MHz or 243 MHz for ^{31}P NMR and 100 MHz or 150 MHz for ^{13}C NMR in $CDCl_3$. Chemical shifts (δ) are given in ppm relative to the TMS scale ($CHCl_3$: δ 7.26 for proton and δ 77.16 for carbon). Chemical shifts (δ) are reported in ppm, and coupling constants (*J*) are in Hertz (Hz). The following abbreviations were used to explain the multiplicities: s = singlet, d = doublet, t = triplet, dd = doublet of doublets, dt = doublet of triplets, q = quartet, m = multiplet, br = broad. Flash column chromatograph was carried out using 200-300 mesh silica gel at medium pressure. High resolution mass spectra (HRMS) were recorded on a MALDI-TOF spectrometer. Sample analysis was conducted with an ultrahigh-resolution mass spectrometer (Q Exactive Hybrid Quadrupole-Orbitrap Mass Spectrometer; Thermo Scientific, USA). Optical rotation was obtained on a Rudolph Research Analytical (Atopol I). HPLC analysis was performed on Waters e2695, UV detection monitored at 210 nm, 254 nm, 270 nm or 290 nm using a CHIRALCEL® OD-H or CHIRALPAK® AD-H, AS-H, or Daicel CHIRALPAK IA, IB, IC, ID, IF, IH column with hexane and *i*-PrOH as the eluent. Unless otherwise noted, all reagents were purchased from commercial suppliers and used without purification. All air- and moisture-sensitive manipulations were carried out in a glove box under nitrogen. Anhydrous toluene and THF were distilled from sodium and benzophenone prior to use.[1] Other solvents are the superdry solvent purchased from TIV.

2. Synthesis of substrates

2.1. Procedures for the synthesis of alcohols (**S1**-**S11**)[2]

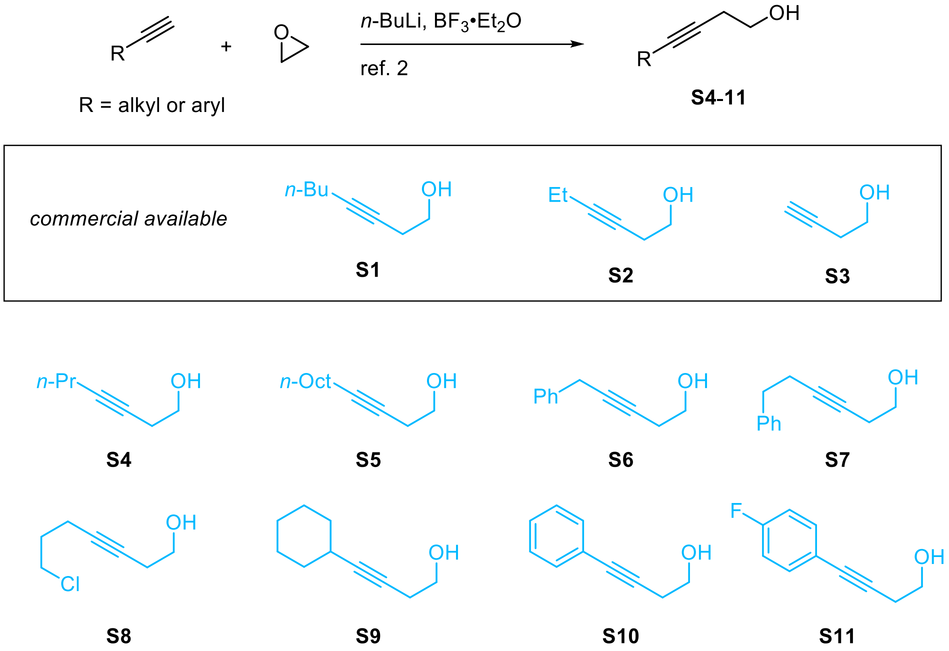

Figure S1. Procedures for the synthesis of alcohols (**S1-11**)

Under an argon atmosphere, a solution of alkyne (6.0 g, 19.5 mmol, 1.2 equiv.) in THF (30 mL) was cooled to –78 °C. To this cold solution was added *n*-BuLi (2.42 M, 8.1 mL, 19.5 mmol, 1.2 equiv) dropwise. After stirring at –78 °C for 1 h, BF_3•Et_2O (4.7 g, 33.3 mmol, 0.8 equiv.) was introduced slowly. The mixture was stirred for 30 min, followed by the dropwise addition of an epoxide solution (6.5 mL, 2.5 M in THF, 16.2 mmol, 1.0 equiv.). Stirring was continued at –78 °C for an additional 6 h. The reaction was quenched with saturated NH_4Cl (aq), extracted with Et_2O, and the combined organic layers were washed with brine, dried over Na_2SO_4, and concentrated under reduced pressure. Flash column chromatography on silica gel (hexanes/EtOAc = 80/20) afforded alcohol **S1-11** in 20-60% isolated yields.

2.2. Procedures for the synthesis of arylphosphinic acid (**S12-S24**)[3]

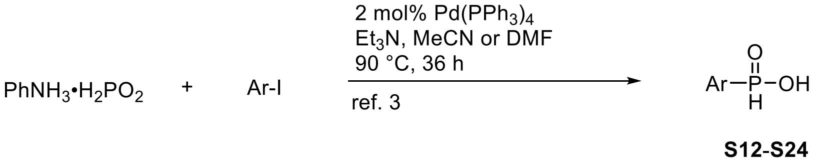

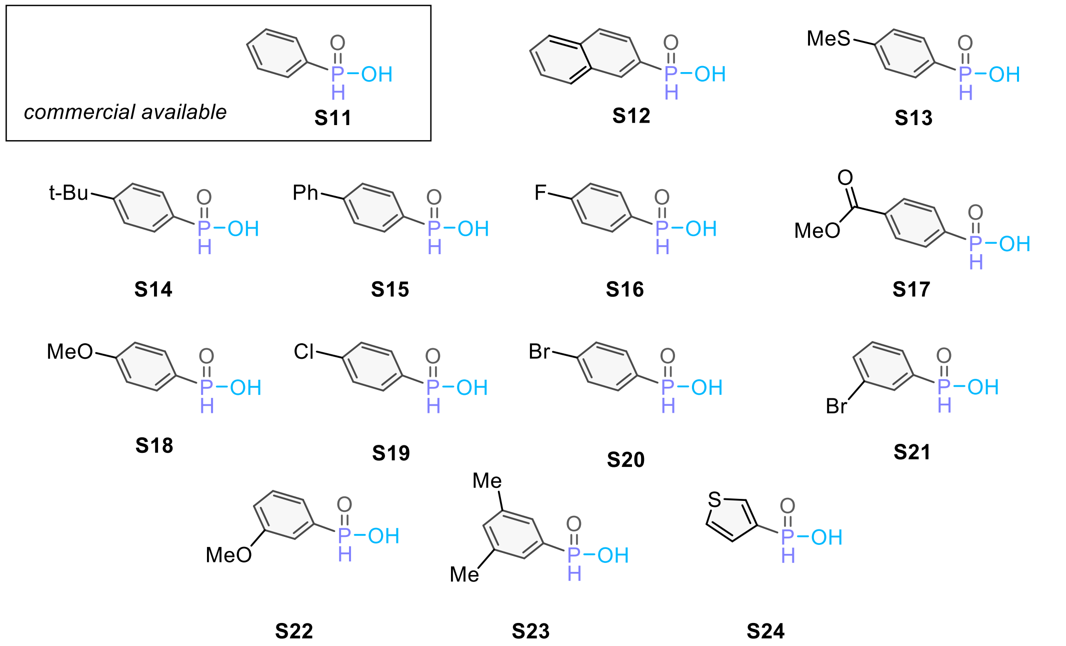

Figure S2. Procedures for the synthesis of phosphinic acid **(S12-S24)**

Under nitrogen, aryl iodides (2 mmol), anilinium hypophosphite (2.2 mmol) and Et_3N (6 mmol) were dissolved in anhydrous DMF (10 mL). After $Pd(PPh_3)_4$ (2 mol%, 0.04 mmol) was introduced, the mixture was heated to 85 °C. Upon completion, the solvent was removed under reduced pressure, the residue acidified with 1 M $KHSO_4$ (saturated with NaCl) and extracted with EtOAc. The combined organic layers were dried over $MgSO_4$, concentrated, and the resulting aryl phosphinic acid was obtained **S12-S24** without additional purification.

2.3. Procedures for the synthesis of phosphinic acid (**S25**)[4]

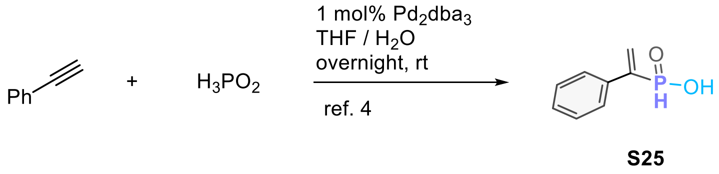

H_3PO_2 (0.396 g, 6 mmol, 3 equiv.) in H_2O was treated with phenylacetylene (2 mmol, 1 equiv.)

in THF. After 5 min of stirring at room temperature, Pd_2dba_3 (0.0092 g, 0.01 mmol, 1 mol% Pd) were introduced, and the mixture was stirred at room temperature for overnight. Upon completion, the solvent was removed under reduced pressure, the residue acidified with 1 M $KHSO_4$ (saturated with NaCl) and extracted with EtOAc. The combined organic layers were dried over $MgSO_4$, concentrated, and the resulting phenyl phosphinic acid was obtained **S25** without additional purification.

2.4. Procedures for the synthesis of arylphosphinate (**1a-1y**)

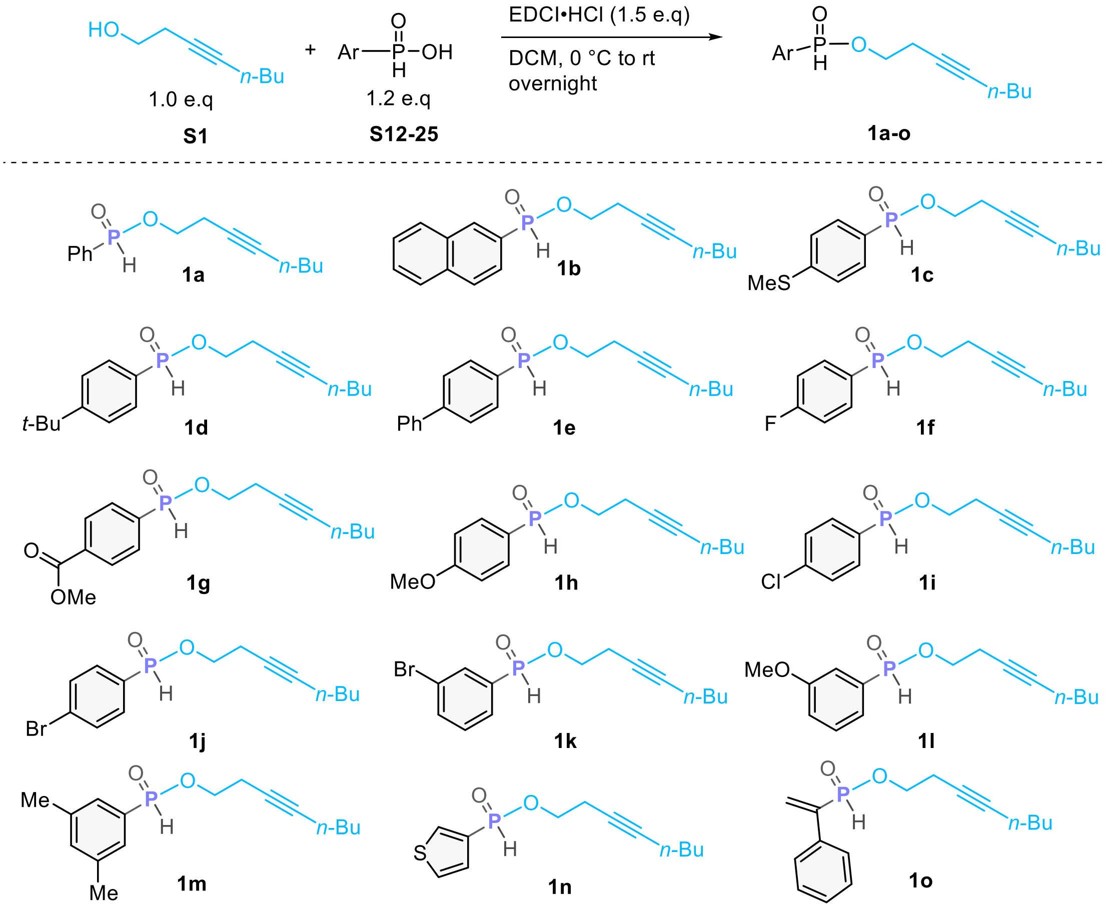

Figure S3. Procedures for the synthesis of arylphosphinate **(1a-o)**

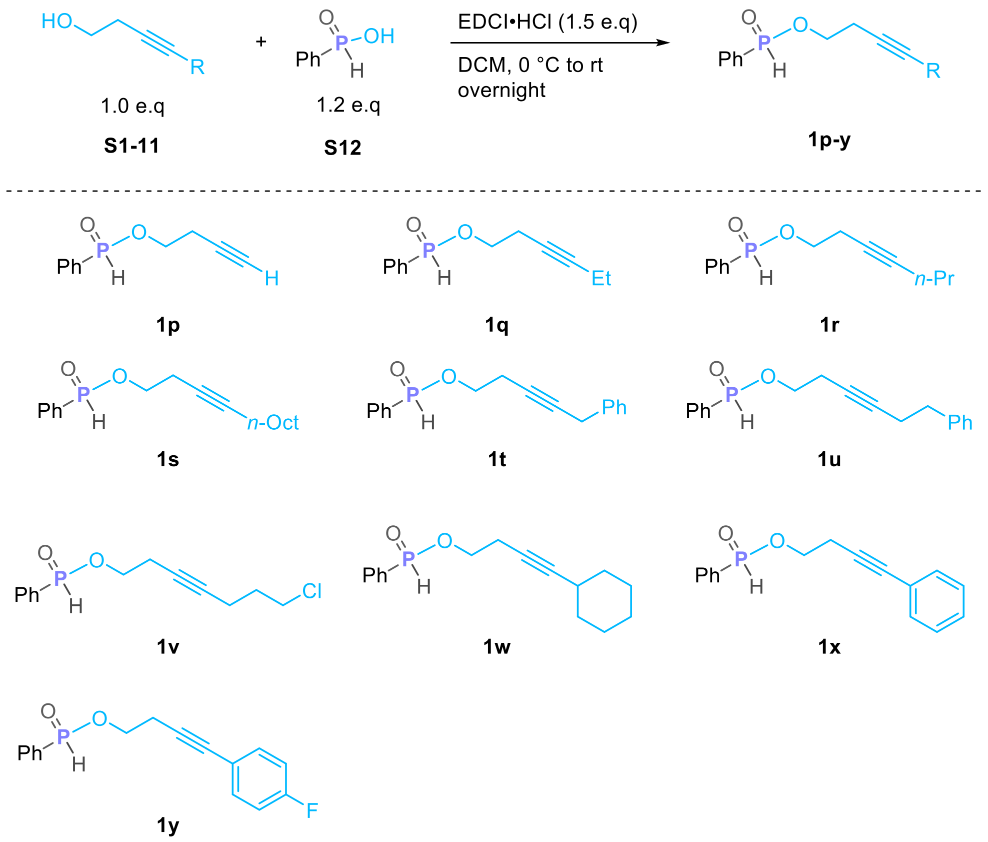

Figure S4. Procedures for the synthesis of arylphosphinate **(1p-y)**

A dichloromethane solution (40 mL) of the alcohol (10 mmol, 1.0 equiv.) was cooled to 0 °C and treated with phenylphosphinic acid (12 mmol, 1.2 equiv.). *N*-(3-Dimethylaminopropyl)-*N*′-ethylcarbodiimide hydrochloride (2.86 g, 15 mmol, 1.5 equiv.) was then introduced while stirring, after which the mixture was allowed to warm to room temperature and stirred overnight. When TLC indicated complete consumption of starting material, the reaction was diluted with H_2O (30 mL). The aqueous layer was extracted with DCM (3 × 20 mL), and the combined organic phases were concentrated in vacuo to give the crude product. Purification by flash column chromatography on silica gel (petroleum ether/ethyl acetate 2:1 to 1:1, R_f = 0.4) delivered the phenylphosphinate as a colorless or pale-yellow oil. (Caution: i. the product is base-labile and up to ~50 % may remain irreversibly adsorbed on the silica column; ii. when R^2 = Ph, a strongly malodorous by-product evolves-appropriate protective measures are advised.)

3. Characterization of substrates

Oct-3-yn-1-yl phenylphosphinate (1a)

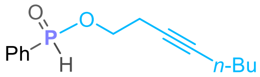

^{1}H NMR (400 MHz, $CDCl_3$) δ 7.79 – 7.68 (m, 2H), 7.56 (d, *J* = 566.9 Hz, 1H), 7.55 – 7.49 (m, 1H), 7.48 – 7.37 (m, 2H), 4.18 – 3.96 (m, 2H), 2.56 – 2.44 (m, 2H), 2.10 – 1.98 (m, 2H), 1.41 – 1.26 (m, 4H), 0.80 (t, *J* = 7.1 Hz, 3H). ^{31}P NMR (162 MHz, $CDCl_3$) δ 25.48. ^{13}C NMR (100 MHz, $CDCl_3$) δ 133.1 (d, *J* = 2.9 Hz), 130.9 (d, *J* = 11.8 Hz), 129.6 (d, *J* = 132.0 Hz), 128.7 (d, *J* = 13.9 Hz), 82.6, 74.9, 64.1 (d, *J* = 6.6 Hz), 30.8, 21.8, 21.2 (d, *J* = 6.6 Hz), 18.2, 13.5.

Following general procedure, the recovered starting material (*R*)-**1a** was obtained in 26% isolated yield (13.0 mg), colorless oil. 49% NMR yield using PPh_3 as internal standard. $[\alpha]^{23}_{D}$ = 4.80 (*c* = 0.5, $CHCl_3$) for 92% *ee*. HPLC analysis: The enantiomeric excess was detected at 210 nm by Daicel Chiralcel ID (0.46 cm x 25 cm), ratio of hexanes/IPA = 60/40, flow rate = 1.0 mL/min, retention time: 7.6 min (minor), 8.5 min (major). HRMS (ESI) *m/z*: $[M+O-H]^-$ calcd for $C_{14}H_{18}O_3P$: 265.0994; found 265.1002.

Oct-3-yn-1-yl naphthalen-2-ylphosphinate (1b)

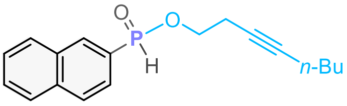

^{1}H NMR (600 MHz, $CDCl_3$) δ 8.39 (d, *J* = 15.9 Hz, 1H), 7.95 – 7.89 (m, 2H), 7.86 (d, *J* = 8.1 Hz, 1H), 7.75 (d, *J* = 568.7 Hz, 1H), 7.77 – 7.71 (m, 1H), 7.62 – 7.53 (m, 2H), 4.25 – 4.06 (m, 2H), 2.63 – 2.56 (m, 2H), 2.14 – 2.08 (m, 2H), 1.45 – 1.31 (m, 4H), 0.85 (t, *J* = 7.3 Hz, 3H). ^{31}P NMR (243 MHz, $CDCl_3$) δ 25.76. ^{13}C NMR (150 MHz, $CDCl_3$) δ 135.3 (d, *J* = 2.4 Hz), 133.6 (d, *J* = 11.9 Hz), 132.3 (d, *J* = 15.2 Hz), 128.9 (d, *J* = 13.9 Hz), 128.8 (d, *J* = 45.2 Hz), 128.7 (d, *J* = 13.5 Hz), 127.9, 127.1, 126.5 (d, *J* = 132.3 Hz), 125.1 (d, *J* = 12.5 Hz), 82.7, 74.9, 64.3 (d, *J* = 6.5 Hz), 30.8, 21.8, 21.3 (d, *J* = 6.7 Hz), 18.3, 13.5.

Following general procedure, the recovered starting material (*R*)-**1b** was obtained in 27% isolated yield (16.2 mg), colorless oil. 46% NMR yield using PPh_3 as internal standard. $[\alpha]^{23}_{D}$ = -15.39 (*c* = 0.5, $CHCl_3$) for 85% *ee*. HPLC analysis: The enantiomeric excess was detected at 254 nm by CHIRALCEL® OD-H (0.46 cm x 25 cm), ratio of hexanes/IPA = 85 /15, flow rate =

1.0 mL/min, retention time: 10.0 min (minor), 12.8 min (major). HRMS (ESI) *m/z*: $[M-C_8H_{13}]^-$ calcd for $C_{10}H_8O_2P$: 191.0262; found 191.0264.

Oct-3-yn-1-yl (4-(methylthio)phenyl)phosphinate (1c)

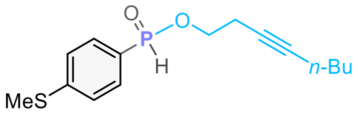

^{1}H NMR (400 MHz, $CDCl_3$) δ 7.74 – 7.62 (m, 2H), 7.61 (d, *J* = 568.7 Hz, 1H), 7.35 – 7.22 (m, 2H), 4.23 – 3.99 (m, 2H), 2.65 – 2.52 (m, 2H), 2.50 (s, 3H), 2.16 – 2.08 (m, 2H), 1.47 – 1.34 (m, 4H), 0.92 – 0.84 (m, 3H). ^{31}P NMR (243 MHz, $CDCl_3$) δ 25.20. ^{13}C NMR (100 MHz, $CDCl_3$) δ 146.0 (d, *J* = 3.1 Hz), 131.1 (d, *J* = 12.4 Hz), 125.0 (d, *J* = 14.3 Hz), 124.8 (d, *J* = 135.9 Hz), 82.5, 74.9, 64.0 (d, *J* = 6.6 Hz), 30.7, 21.7, 21.1 (d, *J* = 6.7 Hz), 18.2, 14.5, 13.4.

Following general procedure, the recovered starting material (*R*)-**1c** was obtained in 22% isolated yield (13.0 mg), colorless oil. 43% NMR yield using PPh_3 as internal standard. $[\alpha]^{23}_D$ = 3.90 (*c* = 1.0, $CHCl_3$) for 91% *ee*. HPLC analysis: The enantiomeric excess was detected at 210 nm by CHIRALCEL® OD-H (0.46 cm x 25 cm), ratio of hexanes/IPA = 80/20, flow = 1.0 mL/min, retention time: 9.0 min (minor), 10.8 min (major). HRMS (ESI) *m/z*: $[M+O-H]^-$ calcd for $C_{15}H_{20}O_3PS$: 311.0871, found 311.0886,.

Oct-3-yn-1-yl (4-(tert-butyl)phenyl)phosphinate (1d)

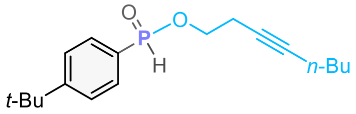

^{1}H NMR (400 MHz, $CDCl_3$) δ 7.70 (dd, *J* = 13.5, 8.4 Hz, 2H), 7.58 (d, *J* = 565.2 Hz, 1H), 7.49 (dd, *J* = 8.4, 3.2 Hz, 2H), 4.18 – 3.98 (m, 2H), 2.58 – 2.49 (m, 2H), 2.13 – 2.03 (m, 2H), 1.44 – 1.29 (m, 4H), 1.29 (s, 9H), 0.84 (t, *J* = 7.1 Hz, 3H). ^{31}P NMR (243 MHz, $CDCl_3$) δ 25.79. ^{13}C NMR (100 MHz, $CDCl_3$) δ 156.8 (d, *J* = 3.0 Hz), 131.0 (d, *J* = 12.2 Hz), 127.0, 125.8 (d, *J* = 14.2 Hz), 82.6, 75.0, 64.1 (d, *J* = 6.6 Hz), 35.2, 31.1, 30.9, 21.9, 21.3 (d, *J* = 6.9 Hz), 18.4, 13.6.

Following general procedure, the recovered starting material (*R*)-**1d** was obtained in 23% isolated yield (14.1 mg), colorless oil. 45% NMR yield using PPh_3 as internal standard. $[\alpha]^{24}_D$ = 6.75 (*c* = 0.5, $CHCl_3$) for 88% *ee*. HPLC analysis: The enantiomeric excess was detected at

210 nm by Daicel Chiralcel ID (0.46 cm x 25 cm), ratio of hexanes/IPA = 60/40, flow = 1.0 mL/min, retention time: 5.8 min (minor), 6.2 min (major). HRMS (ESI) *m/z*: $[M-C_8H_{13}]^-$ calcd for $C_{10}H_{14}O_2P$: 197.0731; found 197.0733.

Oct-3-yn-1-yl [1,1'-biphenyl]-4-ylphosphinate (1e)

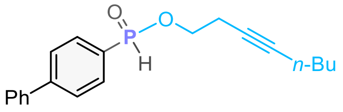

^{1}H NMR (400 MHz, $CDCl_3$) δ 7.90 – 7.79 (m, 2H), 7.72 – 7.64 (m, 2H), 7.64 (d, *J* = 567.8 Hz, 1H), 7.59 – 7.52 (m, 2H), 7.46 – 7.39 (m, 2H), 7.39 – 7.31 (m, 1H), 4.24 – 4.00 (m, 2H), 2.64 – 2.47 (m, 2H), 2.19 – 2.02 (m, 2H), 1.48 – 1.25 (m, 4H), 0.84 (t, *J* = 7.2 Hz, 3H). ^{31}P NMR (162 MHz, $CDCl_3$) δ 25.33. ^{13}C NMR (100 MHz, $CDCl_3$) δ 145.9 (d, *J* = 2.9 Hz), 139.6, 131.5 (d, *J* = 12.2 Hz), 128.9, 128.3, 128.0 (d, *J* = 133.3 Hz), 127.3 (d, *J* = 14.3 Hz), 127.2, 82.6, 75.0, 64.2 (d, *J* = 6.5 Hz), 30.8, 21.8, 21.2 (d, *J* = 6.7 Hz), 18.3, 13.6.

Following general procedure, the recovered starting material (*R*)-**1e** was obtained in 30% isolated yield (19.6 mg), colorless oil. 48% NMR yield using PPh_3 as internal standard. $[\alpha]^{22}_D$ = 33.80 (*c* = 0.5, $CHCl_3$) for 90% *ee*. HPLC analysis: The enantiomeric excess was detected at 210 nm by Daicel Chiralcel IH (0.46 cm x 25 cm), ratio of hexanes/IPA = 70/30, flow rate = 1.0 mL/min, retention time: 23.6 min (minor), 21.4 min (major). HRMS (ESI) *m/z*: $[M-C_8H_{13}]^-$ calcd for $C_{20}H_{22}O_3P$: 217.0424; found 217.0420.

Oct-3-yn-1-yl (4-fluorophenyl)phosphinate (1f)

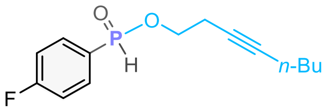

^{1}H NMR (400 MHz, $CDCl_3$) δ 7.54 – 7.34 (m, 2H), 7.24 (d, *J* = 570.7 Hz, 1H), 6.89 – 6.71 (m, 2H), 3.80 – 3.64 (m, 2H), 2.26 – 2.09 (m, 2H), 1.79 – 1.61 (m, 2H), 1.07 – 0.89 (m, 4H), 0.54 – 0.41 (m, 3H). ^{31}P NMR (162 MHz, $CDCl_3$) δ 23.11. ^{19}F NMR (377 MHz, $CDCl_3$) δ -105.01, -105.02. ^{13}C NMR (100 MHz, $CDCl_3$) δ 164.9 (dd, *J* = 254.3, 3.5 Hz), 133.0 (dd, *J* = 13.3, 9.1 Hz), 125.3 (dd, *J* = 134.9, 3.3 Hz), 115.3 (dd, *J* = 21.5, 15.0 Hz), 81.7, 74.3, 63.5 (d, *J* = 6.4 Hz), 30.1, 21.0, 20.4 (d, *J* = 6.5 Hz), 17.4, 12.7.

Following general procedure, the recovered starting material (*R*)-**1f** was obtained in 20% isolated yield (10.7 mg), colorless oil. 46% NMR yield using PPh_3 as internal standard. $[\alpha]^{23}_D$ = -6.80 (*c* = 1.0, $CHCl_3$) for 91% *ee*. HPLC analysis: The enantiomeric excess was detected at 210 nm by Daicel Chiralcel ID (0.46 cm x 25 cm), ratio of hexanes/IPA = 70/30, flow rate = 1.0 mL/min, retention time: 7.5 min (minor), 9.2 min (major). HRMS (ESI) *m/z*: $[M-C_8H_{13}]^-$ calcd for $C_6H_5FO_2P$: 159.0011; found 159.0006.

Methyl 4-(butoxyphosphinyl)benzoate (1g)

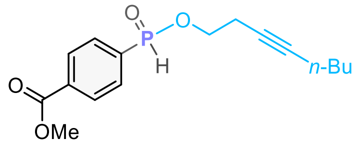

^1H NMR (400 MHz, $CDCl_3$) δ 8.14 (dd, *J* = 8.3, 3.2 Hz, 2H), 7.87 (dd, *J* = 13.4, 8.3 Hz, 2H), 7.65 (d, *J* = 572.6 Hz, 1H), 4.24 – 4.04 (m, 2H), 3.92 (s, 3H), 2.69 – 2.49 (m, 2H), 2.12 – 2.06 (m, 2H), 1.45 – 1.31 (m, 4H), 0.85 (t, *J* = 7.1 Hz, 3H). ^{31}P NMR (162 MHz, $CDCl_3$) δ 23.81. ^{13}C NMR (100 MHz, $CDCl_3$) δ 166.1, 134.4 (d, *J* = 3.0 Hz), 134.3 (d, *J* = 129.7 Hz), 131.2 (d, *J* = 12.0 Hz), 129.7 (d, *J* = 13.9 Hz), 82.9, 74.9, 64.7 (d, *J* = 6.6 Hz), 52.6, 30.9, 22.0, 21.4 (d, *J* = 6.7 Hz), 18.4, 13.6.

Following general procedure, the recovered starting material (*R*)-**1g** was obtained in 21% isolated yield (12.9 mg), colorless oil. 45% NMR yield using PPh_3 as internal standard. $[\alpha]^{23}_D$ = 23.99 (*c* = 0.5, $CHCl_3$) for 88% *ee*. HPLC analysis: The enantiomeric excess was detected at 254 nm by Daicel Chiralcel ID (0.46 cm x 25 cm), ratio of hexanes/IPA = 60/40, flow rate = 1.0 mL/min, retention time: 10.0 min (minor), 11.8 min (major). HRMS (ESI) *m/z*: $[M-C_8H_{13}]^-$ calcd for $C_8H_8O_4P$: 199.0160; found 199.0162.

Oct-3-yn-1-yl (4-methoxyphenyl)phosphinate (1h)

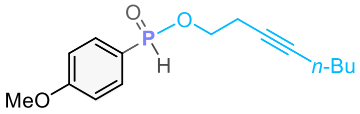

^1H NMR (400 MHz, $CDCl_3$) δ 7.47 (d, *J* = 569.2 Hz, 1H), 7.33 – 7.11 (m, 3H), 7.04 – 6.91 (m, 1H), 4.09 – 3.87 (m, 2H), 3.69 (s, 3H), 2.51 – 2.35 (m, 2H), 2.04 – 1.91 (m, 2H), 1.35 – 1.15 (m, 4H), 0.78 – 0.67 (m, 3H). ^{31}P NMR (243 MHz, $CDCl_3$) δ 25.26. ^{13}C NMR (100 MHz, $CDCl_3$) δ

159.5 (d, J = 17.1 Hz), 130.6 (d, J = 130.8 Hz), 129.9 (d, J = 16.4 Hz), 122.7 (d, J = 11.9 Hz), 119.3 (d, J = 2.9 Hz), 115.2 (d, J = 13.1 Hz), 82.4, 74.8, 64.0 (d, J = 6.6 Hz), 55.2, 30.6, 21.7, 21.0 (d, J = 6.7 Hz), 18.1, 13.4.

Following general procedure, the recovered starting material (*R*)-**1h** was obtained in 30% isolated yield (16.8 mg), colorless oil. 44% NMR yield using PPh_3 as internal standard. $[\alpha]^{24}_D$ = 2.90 (*c* = 1.0, $CHCl_3$) for 95% *ee*. HPLC analysis: The enantiomeric excess was detected at 270 nm by Daicel Chiralcel ID (0.46 cm x 25 cm), ratio of hexanes/IPA = 60/40, flow rate = 1.0 mL/min, retention time: 9.3 min (minor), 12.5 min (major). HRMS (ESI) *m/z*: $[M+O-H]^-$ calcd for $C_{15}H_{20}O_4P$: 295.1099; found 295.1112.

Oct-3-yn-1-yl (4-chlorophenyl)phosphinate (1i)

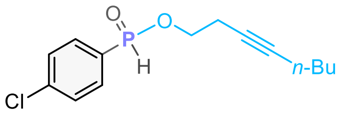

^1H NMR (400 MHz, $CDCl_3$) δ 7.68 (dd, J = 13.3, 8.4 Hz, 2H), 7.56 (d, J = 573.0 Hz, 1H), 7.43 (dd, J = 8.4, 2.7 Hz, 2H), 4.20 – 3.94 (m, 2H), 2.60 – 2.38 (m, 2H), 2.10 – 2.01 (m, 2H), 1.40 – 1.26 (m, 4H), 0.89 – 0.77 (m, 3H). ^{31}P NMR (162 MHz, $CDCl_3$) δ 23.85 ^{13}C NMR (100 MHz, $CDCl_3$) δ 139.9 (d, J = 3.6 Hz), 132.6 (d, J = 12.7 Hz), 129.2 (d, J = 14.5 Hz), 128.1 (d, J = 133.6 Hz), 82.8, 74.9, 64.4 (d, J = 6.6 Hz), 30.9, 21.9, 21.3 (d, J = 6.7 Hz), 18.3, 13.6.

Following general procedure, the recovered starting material (*R*)-**1i** was obtained in 15% isolated yield (8.5 mg), colorless oil. 40% NMR yield using PPh_3 as internal standard. $[\alpha]^{24}_D$ = 1.40 (*c* = 2.0, $CHCl_3$) for 89% *ee*. HPLC analysis: The enantiomeric excess was detected at 220 nm by Daicel Chiralcel ID (0.46 cm x 25 cm), ratio of hexanes/IPA = 60/40, flow rate = 1.0 mL/min, retention time: 6.9 min (minor), 8.3 min (major). HRMS (ESI) *m/z*: $[M-C_8H_{13}]^-$ calcd for $C_6H_5ClO_2P$: 174.9716; found 174.9715.

Oct-3-yn-1-yl (4-bromophenyl)phosphinate (1j)

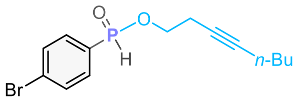

^{1}H NMR (400 MHz, $CDCl_3$) δ 7.69 – 7.43 (m, 4H), 7.50 (d, *J* = 572.9 Hz, 1H), 4.14 – 3.90 (m, 2H), 2.46 (t, *J* = 6.9 Hz, 2H), 2.08 – 1.91 (m, 2H), 1.35 – 1.18 (m, 4H), 0.75 (t, *J* = 7.0 Hz, 3H). ^{31}P NMR (162 MHz, $CDCl_3$) δ 23.83. ^{13}C NMR (100 MHz, $CDCl_3$) δ 132.4 (d, *J* = 12.5 Hz), 131.9 (d, *J* = 14.3 Hz), 128.4 (d, *J* = 132.9 Hz), 128.3 (d, *J* = 3.6 Hz), 82.6, 74.8, 64.3 (d, *J* = 6.5 Hz), 30.7, 21.7, 21.1 (d, *J* = 6.6 Hz), 18.2, 13.4.

Following general procedure, the recovered starting material (*R*)-**1j** was obtained in 21% isolated yield (13.8 mg), colorless oil. 40% NMR yield using PPh_3 as internal standard. $[\alpha]^{23}_{D}$ = -6.30 (*c* = 1.0, $CHCl_3$) for 92% *ee*. HPLC analysis: The enantiomeric excess was detected at 230 nm by CHIRALCEL® OD-H (0.46 cm x 25 cm), ratio of hexanes/IPA = 90/10, flow rate = 1.0 mL/min, retention time: 10.6 min (minor), 13.7 min (major). HRMS (ESI) *m/z*: $[M-C_8H_{13}]^-$ calcd for $C_6H_5BrO_2P$: 218.9211; found 218.9215.

Oct-3-yn-1-yl (3-bromophenyl)phosphinate (1k)

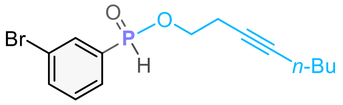

^{1}H NMR (400 MHz, $CDCl_3$) δ7.77 (dt, *J* = 13.8, 1.7 Hz, 1H), 7.62 – 7.51 (m, 2H), 7.46 (d, *J* = 574.8 Hz, 1H), 7.23 (td, *J* = 7.8, 3.9 Hz, 1H), 4.06 – 3.90 (m, 2H), 2.47 – 2.36 (m, 2H), 2.02 – 1.90 (m, 2H), 1.30 – 1.16 (m, 4H), 0.70 (t, *J* = 7.2 Hz, 3H). ^{31}P NMR (243 MHz, $CDCl_3$) δ 22.57. ^{13}C NMR (100 MHz, $CDCl_3$) δ 135.9 (d, *J* = 2.8 Hz), 133.5 (d, *J* = 12.5 Hz), 131.9 (d, *J* = 129.5 Hz), 130.2 (d, *J* = 14.6 Hz), 129.3 (d, *J* = 11.3 Hz), 122.9 (d, *J* = 17.7 Hz), 82.5, 74.6, 64.3 (d, *J* = 6.6 Hz), 30.6, 21.6, 21.0 (d, *J* = 6.6 Hz), 18.1, 13.3.

Following general procedure, the recovered starting material (*R*)-**1k** was obtained in 15% isolated yield (9.8 mg), colorless oil. 42% NMR yield using PPh_3 as internal standard. $[\alpha]^{22}_{D}$ = -9.00 (*c* = 0.5, $CHCl_3$) for 80% *ee*. HPLC analysis: The enantiomeric excess was detected at 210 nm by Daicel Chiralcel ID (0.46 cm x 25 cm), ratio of hexanes/IPA = 70/30, flow = 1.0 mL/min, retention time: 13.6 min (minor), 8.9 min(major). HRMS (ESI) *m/z*: $[M-C_8H_{13}]^-$ calcd for $C_6H_5BrO_2P$: 218.9211; found 218.9215.

Oct-3-yn-1-yl (3-methoxyphenyl)phosphinate (1l)

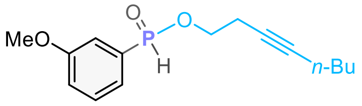

^{1}H NMR (400 MHz, $CDCl_3$) δ 7.47 (d, *J* = 569.2 Hz, 1H), 7.32 – 7.14 (m, 3H), 7.02 – 6.93 (m, 1H), 4.05 – 3.90 (m, 2H), 3.69 (s, 3H), 2.52 – 2.35 (m, 2H), 2.04 – 1.91 (m, 2H), 1.33 – 1.16 (m, 4H), 0.79 – 0.65 (m, 3H). ^{31}P NMR (243 MHz, $CDCl_3$) δ 25.26. ^{13}C NMR (100 MHz, $CDCl_3$) δ 159.4 (d, *J* = 17.1 Hz), 130.6 (d, *J* = 130.8 Hz), 129.8 (d, *J* = 16.4 Hz), 122.7 (d, *J* = 11.9 Hz), 119.3 (d, *J* = 2.9 Hz), 115.2 (d, *J* = 13.1 Hz), 82.4, 74.8, 64.0 (d, *J* = 6.6 Hz), 55.2, 30.6, 21.7, 21.0 (d, *J* = 6.7 Hz), 18.1, 13.4.

Following general procedure, the recovered starting material (*R*)-**1l** was obtained in 22% isolated yield (12.3 mg), colorless oil. 43% NMR yield using PPh_3 as internal standard. $[\alpha]^{23}_D$ = -0.40 (*c* = 1.0, $CHCl_3$) for 93% *ee*. HPLC analysis: The enantiomeric excess was detected at 210 nm by Daicel Chiralcel IC (0.46 cm x 25 cm), ratio of hexanes/IPA = 60/40, flow rate = 1.0 mL/min, retention time: 12.8 min (minor), 15.0 min (major). HRMS (ESI) *m/z*: $[M-C_8H_{13}]^-$ calcd for $C_7H_8O_3P$: 171.0211; found 171.0209.

Oct-3-yn-1-yl (3,5-dimethylphenyl)phosphinate (1m)

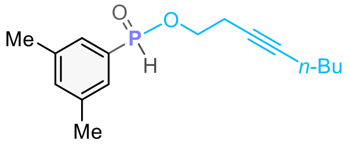

^{1}H NMR (600 MHz, $CDCl_3$) δ 7.57 (d, *J* = 564.2 Hz, 1H), 7.43 – 7.32 (m, 2H), 7.23 – 7.16 (m, 1H), 4.19 – 4.03 (m, 2H), 2.62 – 2.51 (m, 2H), 2.39 – 2.31 (m, 6H), 2.15 – 2.06 (m, 2H), 1.48 – 1.30 (m, 4H), 0.90 – 0.77 (m, 3H). ^{31}P NMR (243 MHz, $CDCl_3$) δ 26.56. ^{13}C NMR (150 MHz, $CDCl_3$) δ 138.7 (d, *J* = 14.5 Hz), 135.0 (d, *J* = 3.3 Hz), 129.4 (d, *J* = 131.4 Hz), 128.6 (d, *J* = 12.1 Hz), 82.8, 75.1, 64.2 (d, *J* = 6.6 Hz), 31.0 (d, *J* = 2.2 Hz), 22.0, 21.4 (d, *J* = 6.9 Hz), 21.3, 18.5, 13.7.

Following general procedure, the recovered starting material (*R*)-**1m** was obtained in 19% isolated yield (10.6 mg), colorless oil. 43% NMR yield using PPh_3 as internal standard. $[\alpha]^{23}_D$ = 5.80 (*c* = 1.0, $CHCl_3$) for 86% *ee*. HPLC analysis: The enantiomeric excess was detected at 230 nm by Daicel Chiralcel ID (0.46 cm x 25 cm), ratio of hexanes/IPA = 70/30, flow rate = 1.0

mL/min, retention time: 8.3 min (minor), 7.6 min (major). HRMS (ESI) *m/z*: $[M+O-H]^-$ calcd for $C_{16}H_{22}O_3P$: 293.1307; found 293.1319.

Oct-3-yn-1-yl thiophen-3-ylphosphinate (1n)

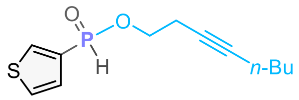

^{1}H NMR (400 MHz, $CDCl_3$) δ 7.99 (dd, *J* = 8.4, 2.8 Hz, 1H), 7.58 (d, *J* = 577.3 Hz, 1H), 7.40 (dt, *J* = 5.3, 2.8 Hz, 1H), 7.29 (t, *J* = 4.7 Hz, 1H), 4.13 – 3.96 (m, 2H), 2.57 – 2.45 (m, 2H), 2.11 – 2.01 (m, 2H), 1.39 – 1.23 (m, 4H), 0.80 (t, *J* = 7.0 Hz, 3H). ^{31}P NMR (162 MHz, $CDCl_3$) δ 17.62. ^{13}C NMR (100 MHz, $CDCl_3$) δ 136.0 (d, *J* = 17.5 Hz), 131.3 (d, *J* = 137.8 Hz), 128.1 (d, *J* = 17.8 Hz), 127.7 (d, *J* = 17.6 Hz), 82.6, 74.9, 64.2 (d, *J* = 6.2 Hz), 30.8, 21.8, 21.2 (d, *J* = 6.8 Hz), 18.3.

Following general procedure, the recovered starting material (*R*)-**1n** was obtained in 19% isolated yield (10.2 mg), colorless oil. 43% NMR yield using PPh_3 as internal standard. $[\alpha]^{22}_D$ = -2.20 (*c* = 0.5, $CHCl_3$) for 88% *ee*. HPLC analysis: The enantiomeric excess was detected at 254 nm by Daicel Chiralcel ID (0.46 cm x 25 cm), ratio of hexanes/IPA = 70/30, flow rate = 1.0 mL/min, retention time: 8.8 min (minor), 9.6 min (major). HRMS (ESI) *m/z*: $[M-C_8H_{13}]^-$ calcd for $C_4H_4O_2PS$: 146.9670; found 146.9662.

Oct-3-yn-1-yl (1-phenylvinyl)phosphinate (1o)

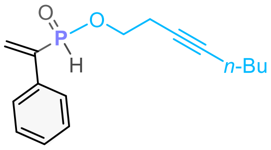

^{1}H NMR (400 MHz, $CDCl_3$) δ 7.58 – 7.40 (m, 2H), 7.37 (d, *J* = 568.0 Hz, 1H), 7.39 – 7.23 (m, 3H), 6.34 – 6.05 (m, 2H), 4.14 – 3.96 (m, 2H), 2.56 – 2.39 (m, 2H), 2.14 – 2.01 (m, 2H), 1.45 – 1.27 (m, 4H), 0.95 – 0.78 (m, 3H). ^{31}P NMR (162 MHz, $CDCl_3$) δ 27.96. ^{13}C NMR (100 MHz, $CDCl_3$) δ 140.9 (d, *J* = 118.6 Hz), 134.4 (d, *J* = 11.9 Hz), 129.6 (d, *J* = 13.1 Hz), 128.1 (d, *J* = 3.1 Hz), 128.07 (d, *J* = 10.4 Hz), 126.5 (d, *J* = 5.8 Hz), 81.9, 74.4, 63.8 (d, *J* = 6.6 Hz), 30.2, 21.2, 20.5 (d, *J* = 6.4 Hz), 17.7, 13.0.

Following general procedure, the recovered starting material (*R*)-**1o** was obtained in 20% isolated yield (11.0 mg), colorless oil. 44% NMR yield using PPh_3 as internal standard. $[\alpha]^{22}_D$ = 24.59 (*c* = 0.5, $CHCl_3$) for 83% *ee*. HPLC analysis: The enantiomeric excess was detected at 210 nm by Daicel Chiralcel IF (0.46 cm x 25 cm), ratio of hexanes/IPA = 95/5, flow rate = 1.0 mL/min, retention time: 28.0 min (minor), 32.4 min (major). HRMS (ESI) *m/z*: $[M+O-H]^-$ calcd for $C_{16}H_{20}O_3P$: 291.1150; found 291.1167.

But-3-yn-1-yl phenylphosphinate (1p)

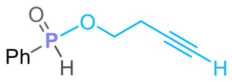

^{1}H NMR (400 MHz, $CDCl_3$) δ 7.62 – 7.41 (m, 2H), 7.33 (d, *J* = 572.2 Hz, 1H), 7.37 – 7.12 (m, 3H), 4.00 – 3.77 (m, 2H), 2.39 – 2.22 (m, 2H), 1.95 – 1.85 (m, 1H). ^{31}P NMR (162 MHz, $CDCl_3$) δ 25.76. ^{13}C NMR (100 MHz, $CDCl_3$) δ 132.7 (d, *J* = 2.8 Hz), 130.3 (d, *J* = 12.1 Hz), 128.6 (d, *J* = 131.8 Hz), 128.2 (d, *J* = 13.9 Hz), 79.1, 70.4, 63.0 (d, *J* = 6.4 Hz), 20.3 (d, *J* = 6.7 Hz).

Following general procedure, the recovered starting material (*R*)-**1p** was obtained in 20% isolated yield (7.8 mg), colorless oil. 46% NMR yield using PPh_3 as internal standard. $[\alpha]^{23}_D$ = -27.99 (*c* = 0.3, $CHCl_3$) for 78% *ee*. HPLC analysis: The enantiomeric excess was detected at 210 nm by Daicel Chiralcel ID (0.46 cm x 25 cm), ratio of hexanes/IPA = 60/40, flow = 1.0 mL/min, retention time: 8.8 min (minor), 9.4 min(major). HRMS (ESI) *m/z*: $[M+Na]^+$ calcd for $C_{10}H_{11}PO_2Na$, 217.0394; found 217.0366.

Hex-3-yn-1-yl phenylphosphinate (1q)

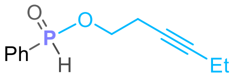

colorless oil. 19% isolated yield. ^{1}H NMR (400 MHz, $CDCl_3$) δ 7.84 – 7.65 (m, 2H), 7.61 (dd, *J* = 567.7 Hz, 1.4 Hz, 1H), 7.60 – 7.52 (m, 1H), 7.52 – 7.40 (m, 2H), 4.22 – 3.92 (m, 2H), 2.65 – 2.40 (m, 2H), 2.17 – 2.00 (m, 2H), 1.23 – 0.79 (m, 3H). ^{31}P NMR (162 MHz, $CDCl_3$) δ 25.62. ^{13}C NMR (100 MHz, $CDCl_3$) δ 133.3 (d, *J* = 3.0 Hz), 131.1 (d, *J* = 11.8 Hz), 129.6 (d, *J* = 131.9 Hz), 128.8 (d, *J* = 13.9 Hz), 84.1, 74.4, 64.2 (d, *J* = 6.6 Hz), 21.3 (d, *J* = 6.8 Hz), 14.1, 12.4.

Following general procedure, the recovered starting material (*R*)-**1q** was obtained in 19% isolated yield (8.4 mg), colorless oil. 35% NMR yield using PPh_3 as internal standard. $[\alpha]^{24}_D$ =

1.95 (c = 2.0, $CHCl_3$) for 94% *ee*. HPLC analysis: The enantiomeric excess was detected at 210 nm by Daicel Chiralcel ID (0.46 cm x 25 cm), ratio of hexanes/IPA = 60/40, flow = 1.0 mL/min, retention time: 7.1 min (minor), 7.5 min(major). HRMS (ESI) *m/z*: $[M+O-H]^-$ calcd for $C_{12}H_{14}O_3P$: 237.0681; found 237.0682.

Hept-3-yn-1-yl phenylphosphinate (1r)

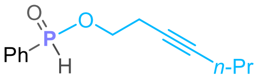

^1H NMR (400 MHz, $CDCl_3$) δ 7.85 – 7.66 (m, 2H), 7.60 (d, J = 567.0 Hz, 1H), 7.60 – 7.50 (m, 1H), 7.50 – 7.37 (m, 2H), 4.20 – 3.94 (m, 2H), 2.62 – 2.29 (m, 2H), 2.16 – 1.96 (m, 2H), 1.50 – 1.37 (m, 2H), 0.95 – 0.83 (m, 3H). ^{31}P NMR (162 MHz, $CDCl_3$) δ 25.52. ^{13}C NMR (100 MHz, $CDCl_3$) δ 133.2 (d, J = 2.9 Hz), 131.0 (d, J = 11.8 Hz), 129.6 (d, J = 131.8 Hz), 128.8 (d, J = 13.7 Hz), 82.5, 75.2, 64.2 (d, J = 6.5 Hz), 22.2, 21.3 (d, J = 6.7 Hz), 20.7, 13.5.

Following general procedure, the recovered starting material (*R*)-**1r** was obtained in 21% isolated yield (9.9 mg), colorless oil. 47% NMR yield using PPh_3 as internal standard. $[\alpha]^{24}_D$ = -4.80 (c = 0.25, $CHCl_3$) for 90% *ee*. HPLC analysis: The enantiomeric excess was detected at 210 nm by Daicel Chiralcel ID (0.46 cm x 25 cm), ratio of hexanes/IPA = 60/40, flow = 1.0 mL/min, retention time: 7.0 min (minor), 7.6 min(major). HRMS (ESI) *m/z*: $[M+O-H]^-$ calcd for $C_{13}H_{16}O_3P$: 251.0837; found 251.0848.

Dodec-3-yn-1-yl phenylphosphinate (1s)

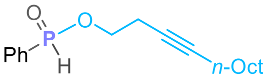

^1H NMR (600 MHz, $CDCl_3$) δ7.78 – 7.69 (m, 2H), 7.56 (d, J = 566.8 Hz, 1H), 7.55 – 7.49 (m, 1H), 7.43 (td, J = 7.6, 3.4 Hz, 2H), 4.15 – 3.98 (m, 2H), 2.55 – 2.44 (m, 2H), 2.07 – 2.00 (m, 2H), 1.40 – 1.35 (m, 2H), 1.31 – 1.10 (m, 10H), 0.79 (t, J = 7.0 Hz, 3H). ^{31}P NMR (243 MHz, $CDCl_3$) δ 25.36. ^{13}C NMR (150 MHz, $CDCl_3$) δ 133.0 (d, J = 2.9 Hz), 130.9 (d, J = 11.8 Hz), 129.6 (d, J = 131.9 Hz), 128.6 (d, J = 13.8 Hz), 82.6, 74.9, 64.1 (d, J = 6.6 Hz), 31.7, 29.0, 29.0, 28.7, 28.7, 21.1 (d, J = 6.7 Hz), 18.5, 14.0.

Following general procedure, the recovered starting material (*R*)-**1s** was obtained in 21% isolated yield (12.9 mg), colorless oil. 42% NMR yield using PPh_3 as internal standard. $[\alpha]^{23}_D$ =

-2.60 (c = 0.5, $CHCl_3$) for 89% *ee*. HPLC analysis: The enantiomeric excess was detected at 210 nm by Daicel Chiralcel ID (0.46 cm x 25 cm), ratio of hexanes/IPA = 85/15, flow = 1.0 mL/min, retention time: 10.6 min (minor), 11.4 min(major). HRMS (ESI) *m/z*: $[M-C_{12}H_{21}]^-$ calcd for $C_6H_6O_2P$: 141.0105; found 141.0099.

5-phenylpent-3-yn-1-yl phenylphosphinate (1t)

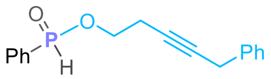

colorless oil. 22% isolated yield. ^{1}H NMR (400 MHz, $CDCl_3$) δ 7.72 – 7.55 (m, 2H), 7.46 (d, J = 567.1 Hz, 1H), 7.45 – 7.36 (m, 1H), 7.35 – 7.22 (m, 2H), 7.21 – 7.08 (m, 4H), 7.08 – 6.99 (m, 1H), 4.11 – 3.87 (m, 2H), 3.40 (d, J = 2.5 Hz, 2H), 2.55 – 2.40 (m, 2H). ^{31}P NMR (162 MHz, $CDCl_3$) δ 25.41. ^{13}C NMR (100 MHz, $CDCl_3$) δ 136.3, 132.6 (d, J = 2.9 Hz), 130.4 (d, J = 11.9 Hz), 129.1 (d, J = 131.6 Hz), 128.2 (d, J = 13.9 Hz), 127.9, 127.3, 126.0, 79.6, 77.2, 63.5 (d, J = 6.5 Hz), 24.5, 20.8 (d, J = 6.7 Hz).

Following general procedure, the recovered starting material (*R*)-**1t** was obtained in 22% isolated yield (12.5 mg), colorless oil. 42% NMR yield using PPh_3 as internal standard. $[\alpha]^{24}_D$ = -1.60 (c = 1.0, $CHCl_3$) for 94% *ee*. HPLC analysis: The enantiomeric excess was detected at 210 nm by Daicel Chiralcel ID (0.46 cm x 25 cm), ratio of hexanes/IPA = 60/40, flow = 1.0 mL/min, retention time: 9.7 min (minor), 11.2 min(major). HRMS (ESI) *m/z*: $[M+O-H]^-$ calcd for $C_{17}H_{16}O_3P$: 299.0837; found 299.0852.

6-Phenylhex-3-yn-1-yl phenylphosphinate (1u)

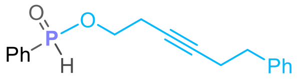

^{1}H NMR (600 MHz, $CDCl_3$) δ 7.83 – 7.69 (m, 2H), 7.60 – 7.51 (m, 1H), 7.55 (d, J = 567.7 Hz, 1H), 7.51 – 7.38 (m, 2H), 7.29 – 7.19 (m, 2H), 7.19 – 7.12 (m, 3H), 4.16 – 3.98 (m, 2H), 2.75 (t, J = 7.5 Hz, 2H), 2.58 – 2.46 (m, 2H), 2.40 (tt, J = 7.5, 2.4 Hz, 2H). ^{31}P NMR (243 MHz, $CDCl_3$) δ 25.50. ^{13}C NMR (150 MHz, $CDCl_3$) δ140.3, 132.9 (d, J = 2.9 Hz), 130.7 (d, J = 11.7 Hz), 129.4 (d, J = 131.7 Hz), 128.5 (d, J = 14.0 Hz), 128.1, 128.0, 125.9, 81.6, 75.7, 63.8 (d, J = 6.6 Hz), 34.8, 20.9 (d, J = 6.6 Hz), 20.5.

Following general procedure, the recovered starting material (*R*)-**1u** was obtained in 20% isolated yield (11.9 mg), colorless oil. 43% NMR yield using PPh_3 as internal standard. $[\alpha]^{23}_{D}$ = 9.0 (*c* = 1.0, $CHCl_3$) for 93% *ee*. HPLC analysis: The enantiomeric excess was detected at 270 nm by Daicel Chiralcel ID (0.46 cm x 25 cm), ratio of hexanes/IPA = 60/40, flow = 1.0 mL/min, retention time: 8.8 min (minor), 9.2 min(major). HRMS (ESI) *m/z*: $[M-C_{12}H_{13}]^-$ calcd for $C_6H_6O_2P$: 141.0105; found 141.0099.

7-chlorohept-3-yn-1-yl phenylphosphinate (1v)

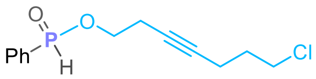

^{1}H NMR (400 MHz, $CDCl_3$) δ 7.75 – 7.56 (m, 2H), 7.47 (d, *J* = 568.4 Hz, 1H), 7.56 – 7.29 (m, 3H), 4.09 – 3.85 (m, 2H), 3.58 – 3.34 (m, 2H), 2.53 – 2.08 (m, 4H), 1.82 – 1.64 (m, 2H). ^{31}P NMR (162 MHz, $CDCl_3$) δ 25.41. ^{13}C NMR (100 MHz, $CDCl_3$) δ 132.9, 130.6 (d, *J* = 11.9 Hz), 129.2 (d, *J* = 132.1 Hz), 128.5 (d, *J* = 13.9 Hz), 80.2, 76.0, 63.7 (d, *J* = 6.6 Hz), 43.4, 31.1, 20.9 (d, *J* = 6.5 Hz), 15.8.

Following general procedure, the recovered starting material (*R*)-**1v** was obtained in 24% isolated yield (13.0 mg), colorless oil. 49% NMR yield using PPh_3 as internal standard. $[\alpha]^{24}_{D}$ = 27.75 (*c* = 2.5, $CHCl_3$) for 87% *ee*. HPLC analysis: The enantiomeric excess was detected at 210 nm by Daicel Chiralcel ID (0.46 cm x 25 cm), ratio of hexanes/IPA = 60/40, flow = 1.0 mL/min, retention time: 9.7 min (minor), 11.9 min(major). HRMS (ESI) *m/z*: $[M+O-H]^-$ calcd for $C_{13}H_{15}ClO_3P$: 285.0447; found 285.0464.

4-Cyclohexylbut-3-yn-1-yl phenylphosphinate (1w)

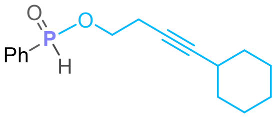

^{1}H NMR (400 MHz, $CDCl_3$) δ 7.85 – 7.71 (m, 2H), 7.62 (d, *J* = 566.7 Hz, 1H), 7.61 – 7.42 (m, 3H), 4.17 – 4.02 (m, 2H), 2.70 – 2.43 (m, 2H), 2.26 (d, *J* = 10.3 Hz, 1H), 1.75 – 1.56 (m, 4H), 1.40 – 1.13 (m, 6H). ^{31}P NMR (162 MHz, $CDCl_3$) δ 25.55. ^{13}C NMR (100 MHz, $CDCl_3$) δ 133.2, 131.1 (d, *J* = 12.9 Hz), 129.7 (d, *J* = 132.2 Hz), 128.8 (d, *J* = 14.6 Hz), 87.0, 74.9, 64.4 (d, *J* = 6.7 Hz), 32.9, 29.1, 25.9, 24.9, 21.3 (d, *J* = 6.9 Hz).

Following general procedure, the recovered starting material (*R*)-**1w** was obtained in 26% isolated yield (14.4 mg), colorless oil. 46% NMR yield using PPh_3 as internal standard. $[\alpha]^{23}_D$ = -27.79 (*c* = 0.5, $CHCl_3$) for 90% *ee*. HPLC analysis: The enantiomeric excess was detected at 210 nm by Daicel Chiralcel ID (0.46 cm x 25 cm), ratio of hexanes/IPA = 85/15, flow = 1.0 mL/min, retention time: 15.8 min (minor), 18.0 min(major). HRMS (ESI) *m/z*: $[M+O-H]^-$ calcd for $C_{16}H_{20}O_3P$: 291.1150; found 291.1166.

4-Phenylbut-3-yn-1-yl phenylphosphinate (1x)

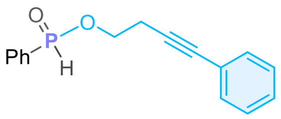

^{1}H NMR (400 MHz, $CDCl_3$) δ 7.92 – 7.78 (m, 2H), 7.68 (d, *J* = 568.4 Hz, 1H), 7.64 – 7.55 (m, 1H), 7.56 – 7.44 (m, 2H), 7.45 – 7.35 (m, 2H), 7.33 – 7.20 (m, 3H), 4.39 – 4.16 (m, 2H), 2.85 (t, *J* = 6.7 Hz, 2H). ^{31}P NMR (162 MHz, $CDCl_3$) δ 25.86. ^{13}C NMR (100 MHz, $CDCl_3$) δ 133.3 (d, *J* = 2.9 Hz), 131.6, 131.1 (d, *J* = 11.8 Hz), 129.5 (d, *J* = 131.7 Hz), 128.8 (d, *J* = 13.9 Hz), 128.3, 128.1, 123.1, 85.5, 82.6, 63.7 (d, *J* = 6.6 Hz), 21.9 (d, *J* = 6.6 Hz).

Following general procedure, the recovered starting material (*R*)-**1x** was obtained in 23% isolated yield (12.4 mg), Light yellow oil. 40% NMR yield using PPh_3 as internal standard. $[\alpha]^{23}_D$ = -29.04 (*c* = 0.5, $CHCl_3$) for 89% *ee*. HPLC analysis: The enantiomeric excess was detected at 254 nm by Daicel Chiralcel ID (0.46 cm x 25 cm), ratio of hexanes/IPA = 60/40, flow = 1.0 mL/min, retention time: 13.3 min (minor), 14.2 min(major). HRMS (ESI) *m/z*: $[M+O-H]^-$ calcd for $C_{16}H_{14}O_3P$: 285.0681; found 285.0690.

4-(4-Fluorophenyl)but-3-yn-1-yl phenylphosphinate (1y)

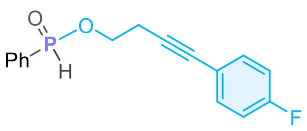

^{1}H NMR (600 MHz, $CDCl_3$) δ 7.82 (dd, *J* = 14.1, 7.5 Hz, 2H), 7.67 (d, *J* = 568.4 Hz, 1H), 7.60 (t, *J* = 7.6 Hz, 1H), 7.50 (td, *J* = 7.5, 3.7 Hz, 2H), 7.38 – 7.31 (m, 2H), 6.97 (t, *J* = 8.5 Hz, 2H), 4.34 – 4.15 (m, 2H), 2.83 (t, *J* = 6.9 Hz, 2H). ^{31}P NMR (243 MHz, $CDCl_3$) δ 25.78. ^{19}F NMR (565 MHz, $CDCl_3$) δ -111.29. ^{13}C NMR (150 MHz, $CDCl_3$) δ 133.4 (d, *J* = 3.0 Hz), 131.8, 131.2 (d, *J*

= 12.0 Hz), 129.7 (d, J = 131.8 Hz), 129.0 (d, J = 13.9 Hz), 128.4, 128.2, 123.2, 85.1, 82.7, 63.8 (d, J = 6.5 Hz), 22.1 (d, J = 6.7 Hz).

Following general procedure, the recovered starting material (*R*)-**1y** was obtained in 38% isolated yield (21.9 mg), White solid. 72% NMR yield using PPh_3 as internal standard. $[\alpha]^{23}_D$ = -9.19 (*c* = 1.0, $CHCl_3$) for 50% *ee*. HPLC analysis: The enantiomeric excess was detected at 210 nm by Daicel CHIRALPAK IC (0.46 cm x 25 cm), ratio of hexanes/IPA = 60/40, flow = 1.0 mL/min, retention time: 12.7 min (minor), 13.5 min(major). HRMS (ESI) *m/z*: $[M+Na]^+$ calcd for $C_{16}H_{15}FPO_2$, 289.0794; found 289.0758.

4. Optimization of reaction conditions

Table S1. Initially screening of Additives[a]

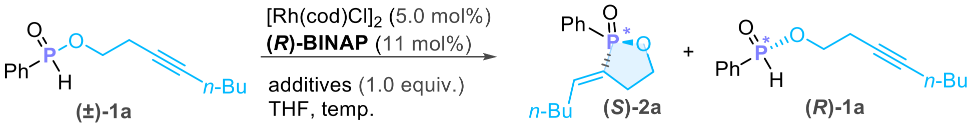

entry	additives	temp. (°C)	conv. of **(±)-1a**	NMR yield of **(*S*)-2a**	*ee* of **(*S*)-2a**
1	$(PhO)_2P(O)OH$	40	60%	23%	87%
2	TsOH	40	34%	54%	62%
3	KOAc	80	< 5%	trace	35%
4	DIEPA	80	< 5%	trace	43%
5	DBU	80	> 99%	ND	-
6	*t*-BuONa	25	> 99%	ND	-

[a]Reaction condition: **(±)-1a** (0.10 mmol), $[Rh(cod)Cl]_2$ (5 mol%), **(*R*)-BINAP** (11 mol%), additives (0.10 mmol), and THF (1.0 mL) were stirred under N_2 atmosphere at reaction temperature indicated. NMR yields were reported using PPh_3 as internal standard. ND = Not Determined.

Table S2. Screening of acid[a]

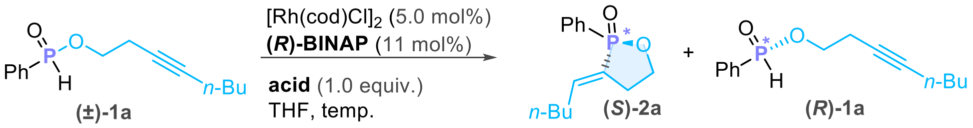

entry	acids	temp. & time	yield and *ee* of **(*R*)-1a**	Yield and *ee* of **(*S*)-2a**	*C*(%)	*S*
1	$PhP(O)(OH)_2$	rt, 48 h	40% yield, 17% *ee*	19% yield, 87% *ee*	16	17
2	$(PhO)_2P(O)OH$	rt, 36 h	49% yield, 44% *ee*	24% yield, 89% *ee*	33	26
3	$(BnO)_2P(O)OH$	rt, 48 h	32% yield, 20% *ee*	27% yield, 85% *ee*	19	15
4	$(BuO)_2P(O)OH$	rt, 24 h	47% yield, 19% *ee*	11% yield, 85% *ee*	18	15
5	C_6F_5COOH	rt, 24 h	37% yield, 23% *ee*	24% yield, 88% *ee*	21	20
6	TFA	rt, 24 h	33% yield, 94% *ee*	57% yield, 70% *ee*	57	19
7	AcOH	40 °C, 24 h	53% yield, 66% *ee*	41% yield, 78% *ee*	46	16
8	PhCOOH	40 °C, 24 h	60% yield, 49%*ee*	23% yield, 80% *ee*	38	15
9	TsOH	rt, 36 h	24% yield, 90% *ee*	66% yield, 66% *ee*	58	14
10	MsOH	rt, 36 h	70% yield, 45% *ee*	14% yield, 80% *ee*	36	14
11	$Ph_2P(O)OH$	40 °C, 24 h	13% yield, 80% *ee*	58% yield, 35% *ee*	70	5

[a]Reaction condition: **(±)-1a** (0.10 mmol), $[Rh(cod)Cl]_2$ (5 mol %), **(*R*)-BINAP** (11 mol %), acids (0.10 mmol), and THF (1.0 mL) were stirred under N_2 atmosphere at reaction temperature indicated. NMR yields were reported using PPh_3 as internal standard. Calculated conversion, C = $ee^{SM/}(ee^{SM} + ee^{P})$. Selectivity factor ($S$) = ln[(1 –C) (1 –$ee^{SM}$)]/ ln[(1 –C) (1 + ee^{SM})].

Table S3. Screening of ligands[a]

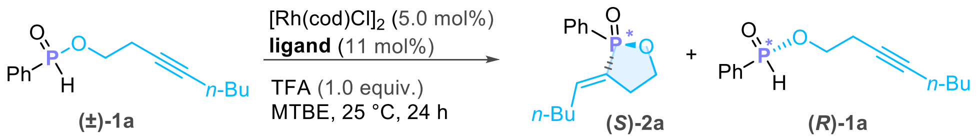

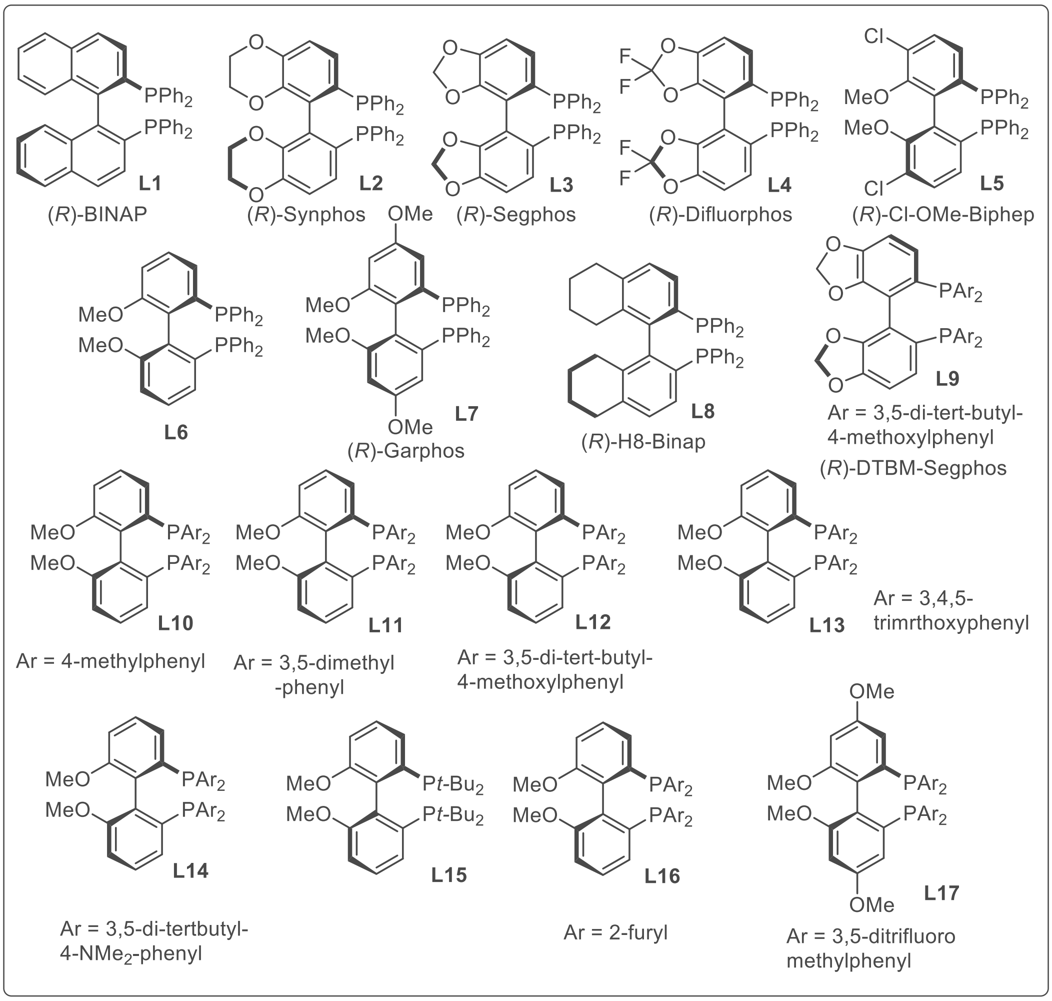

entry	ligand	yield and *ee* of **(*S*)-2a**	entry	ligand	yield and *ee* of **(*S*)-2a**
1	**L1**	31% yield, 90% *ee*	9	**L10**	trace, 78% *ee*
2	**L3**	trace, 91% *ee*	10	**L11**	30% yield, 77% *ee*
3	**L4**	NR	11	**L12**	54% yield, 5% *ee*
4	**L5**	46% yield, 93% *ee*	12	**L13**	34% yield, 8% *ee*
5	**L6**	20% yield, 76% *ee*	13	**L14**	37% yield, 47% *ee*
6	**L7**	NR	14	**L15**	NR
7	**L8**	34% yield, 84% *ee*	15	**L16**	NR
8	**L9**	16% yield, 85% *ee*	16	**L17**	NR

[a]Reaction condition: **(±)-1a** (0.10 mmol), $[Rh(cod)Cl]_2$ (5 mol %), **ligand** (11 mol %), trifluoroacetic acid (0.10 mmol), and MTBE (1.0 mL) were stirred under N_2 atmosphere at reaction temperature indicated. NMR yields were reported using PPh_3 as internal standard. NR = No Reaction.

Table S4. Screening of solvents[a]

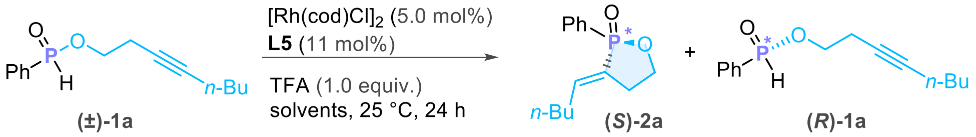

entry	solvent	yield and *ee* of **(*R*)-1a**	yield and *ee* of **(*S*)-2a**	*C*(%)	*S*
1	THF	47% yield, 56% *ee*	31% yield, 91% *ee*	38	37
2	IPA	49% yield, 24% *ee*	15% yield, 77% *ee*	24	10
3	EA	38% yield, 94% *ee*	56% yield, 76% *ee*	55	25
4	MeCN	56% yield, 64% *ee*	40% yield, 71% *ee*	47	11
5	DCE	33% yield, 83% *ee*	51% yield, 56% *ee*	60	9
6	DCM	38% yield, 78% *ee*	49% yield, 60% *ee*	57	9
7	1,4-dioxane	38% yield, 99% *ee*	49% yield, 77% *ee*	56	39
8[b]	toluene	46% yield, 77% *ee*	47% yield, 70% *ee*	52	13

[a]Reaction condition: **(±)-1a** (0.10 mmol), $[Rh(cod)Cl]_2$ (5 mol %), **L6** (11 mol %), trifluoroacetic acid (0.10 mmol), and solvent (1.0 mL) were stirred under N_2 atmosphere at 25 °C for 24 h. NMR yields were reported using PPh_3 as internal standard. [b] 13 h.

Table S5. Screening of ArOH[a]

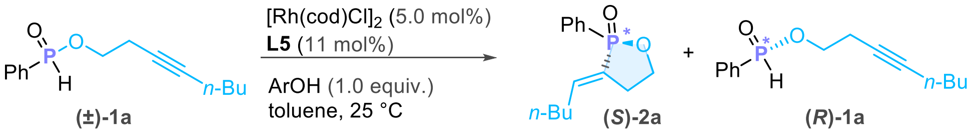

entry	ArOH	time	yield and *ee* of **(*S*)-2a**
1	3,4-difluorophenol	7.5 h	45% yield, 91% *ee*
2	2,6-difluorophenol	7.5 h	21% yield, 94% *ee*
3	2,3-difluorophenol	7.5 h	25% yield, 93% *ee*
4	3,4,5-trifluorophenol	1.5 h	22% yield, 96% *ee*
5	3,4,5-trifluorophenol	8.5 h	46% yield, 90% *ee*
6	2,3,5,6-tetrafluorophenol	7.5 h	37% yield, 92% *ee*
7	pentafluorophenol	1.5 h	27% yield, 95% *ee*
8	3,5-difluorophenol	4 h	47% yield, 92% *ee*

[a]Reaction condition: **(±)-1a** (0.10 mmol), $[Rh(cod)Cl]_2$ (5 mol %), **L6** (11 mol %), ArOH (0.10 mmol), and toluene (1.0 mL) were stirred under N_2 atmosphere at 25 °C for time indicated. NMR yields were reported using PPh_3 as internal standard.

5. Synthesis of *P*-stereogenic cyclic phosphinates

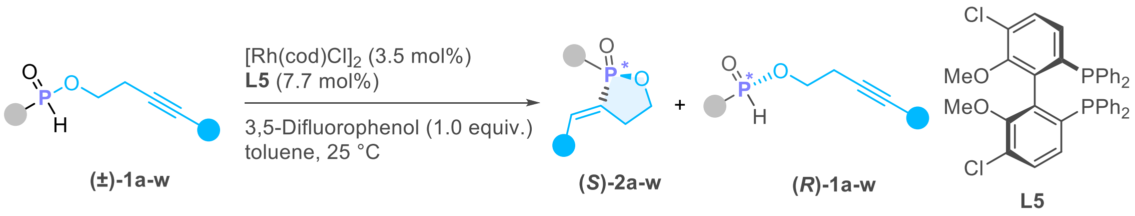

Figure S6. Procedures of the Rh-catalyzed kinetic resolution for the synthesis of *P*-stereogenic cyclic phosphinates with **L5** as ligand

To an oven-dried 10 mL airtight pressure tube were added $[Rh(cod)Cl]_2$ (3.5 mg, 3.5 mol%), **L5** (10.0 mg, 7.7 mol %) and toluene (2 mL) under strict nitrogen atmosphere glove box. Until the mixture turned red, 3,5-difluorophenol (26.0 mg, 0.2 mmol) and **(±)-1a-w** (0.2 mmol) were sequentially added. The reaction was stirred at 25 °C and monitored every 2–8 h by ^{31}P NMR until the ratio of **(*R*)-1a-w** to product **(*S*)-2a-w** approached 1:1, at which point stirring was stopped. The resulting mixture was filtered through a short column of silica gel eluted with ethyl acetate (7 mL × 3). After removal of the solvent under vacuum, the residue was purified by flash chromatography on silica gel to afford desired product **(*S*)-2a-w** [eluent: petroleum ether/ethyl acetate = 2/1 (100ml) to 1/1 or 1/2 (cal~200 mL)]. As **(*R*)-1a-w** is partially lost on the silica, NMR yield of **(*R*)-1a-w** was calculated from the isolated yield of **(*S*)-2a-w** with ^{31}P NMR ratio of **(*R*)-1a-w** to product **(*S*)-2a-w** using PPh_3 as internal standard.

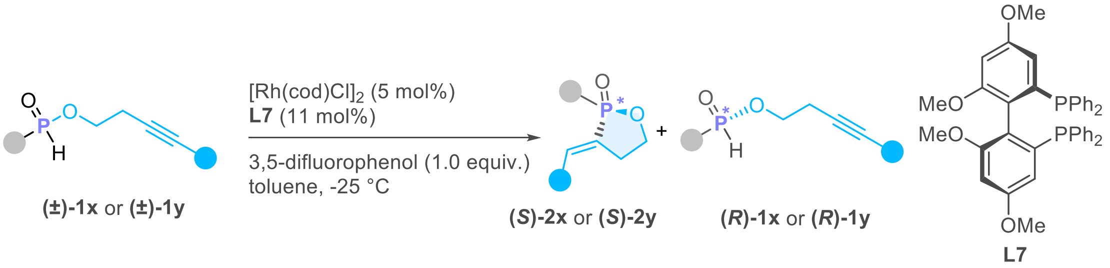

Figure S7. Procedures of the Rh-catalyzed kinetic resolution for the synthesis of *P*-stereogenic cyclic phosphinates with **L7** as ligand

To an oven-dried 10 mL airtight pressure tube were added $[Rh(cod)Cl]_2$ (4.9 mg, 5 mol %), **L7** (14.1 mg,11 mol %) and Toluene (2 mL) under strict nitrogen atmosphere glove box. Until the mixture turned to red. Sequentially adding acid, 3,5-Difluorophenol (26.0 mg, 0.2 mmol) and **1x** or **1y** (0.2 mmol). The reaction was stirred at -25 °C and monitored every 36–60 h by ^{31}P NMR

until the ratio of and **1x** or **1y** to product and **2x** or **2y** approached 1:1, at which point stirring was stopped. The resulting mixture was filtered through a short column of silica gel eluted with ethyl acetate (7 mL × 3). After removal of the solvent under vacuum, the residue was purified by flash chromatography on silica gel to afford desire product **2x** or **2y** [eluent: Petroleum ether/ethyl acetate = 2/1 (100ml) to 1/1 or 1/2 (cal~200 mL). The same reaction was repeated under identical reaction time to determine the isolated yield of **2x** or **2y**; because unreacted **2x** or **2y** is partially lost on the silica, the NMR yield was calculated from the isolated yield of **2x** or **2y** with the ^{31}P NMR ratio of **1x** or **1y** to product **2x** or **2y**.

To an oven-dried 10 mL airtight pressure tube were added $[Rh(cod)Cl]_2$ (4.9 mg, 5 mol %), **L8** (14.1 mg,11 mol %) and toluene (2 mL) under strict nitrogen atmosphere glove box. Until the mixture turned red, 3,5-difluorophenol (26.0 mg, 0.2 mmol) and **(±)-1x or (±)-1y** (0.2 mmol) were sequentially added. The reaction was stirred at 25 °C and monitored every 2–8 h by ^{31}P NMR until the ratio of **(*R*)-1a-w** to product **(*S*)-2a-w** approached 1:1, at which point stirring was stopped. The resulting mixture was filtered through a short column of silica gel eluted with ethyl acetate (7 mL × 3). After removal of the solvent under vacuum, the residue was purified by flash chromatography on silica gel to afford desired product **(*S*)-2a-w** [eluent: petroleum ether/ethyl acetate = 2/1 (100ml) to 1/1 or 1/2 (cal~200 mL)]. As **(*R*)-1a-w** is partially lost on the silica, NMR yield of **(*R*)-1a-w** was calculated from the isolated yield of **(*S*)-2a-w** with ^{31}P NMR ratio of **(*R*)-1a-w** to product **(*S*)-2a-w** using PPh_3 as internal standard.

6. Characterization of products

(*S,E*)-3-pentylidene-2-phenyl-1,2-oxaphospholane 2-oxide ((*S*)-2a)

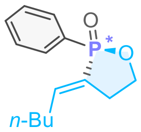

^{1}H NMR (400 MHz, $CDCl_3$) δ 7.83 – 7.70 (m, 2H), 7.60 – 7.39 (m, 3H), 6.39 – 6.20 (m, 1H), 4.60 – 4.34 (m, 2H), 3.06 – 2.70 (m, 2H), 2.23 – 2.06 (m, 2H), 1.47 – 1.18 (m, 4H), 0.85 (t, *J* = 7.2 Hz, 3H). ^{31}P NMR (162 MHz, $CDCl_3$) δ 42.56. ^{13}C NMR (100 MHz, $CDCl_3$) δ 143.8 (d, *J* = 12.2 Hz), 132.5 (d, *J* = 2.9 Hz), 131.7 (d, *J* = 11.1 Hz), 131.2 (d, *J* = 137.6 Hz), 131.0 (d, *J* = 120.2 Hz), 128.6 (d, *J* = 13.6 Hz), 67.9 (d, *J* = 2.7 Hz), 31.1 (d, *J* = 16.0 Hz), 30.4 (d, *J* = 1.5 Hz), 28.8 (d, *J* = 21.0 Hz), 22.4, 13.9.

Following general procedure, the recovered starting material **(*S*)-2a** was obtained in 45% isolated yield (22.5 mg), colorless oil. $[\alpha]^{24}_{D}$ = 12.94 (*c* = 2.0, $CHCl_3$) for 90% *ee*. HPLC analysis: The enantiomeric excess was detected at 210 nm by CHIRALPAK® AD-H (0.46 cm x 25 cm), ratio of hexanes/IPA = 80/20, flow = 1.0 mL/min, retention time: 7.2 min (minor), 8.6 min(major). HRMS (ESI) *m/z*: $[M+H]^+$ calcd for $C_{14}H_{20}PO_2$, 251.1201; found 251.1170.

(*S,E*)-2-(Naphthalen-2-yl)-3-pentylidene-1,2-oxaphospholane 2-oxide ((*S*)-2b)

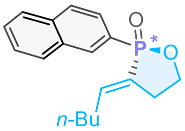

^{1}H NMR (400 MHz, $CDCl_3$) δ 8.43 (dd, *J* = 14.8, 1.4 Hz, 1H), 8.05 – 7.77 (m, 3H), 7.69 – 7.44 (m, 3H), 6.30 (dtt, *J* = 19.5, 7.3, 2.6 Hz, 1H), 4.70 – 4.35 (m, 2H), 3.12 – 2.73 (m, 2H), 2.22 – 2.09 (m, 2H), 1.42 – 1.22 (m, 4H), 0.84 (t, *J* = 7.2 Hz, 3H). ^{31}P NMR (162 MHz, $CDCl_3$) δ 42.68. ^{13}C NMR (100 MHz, $CDCl_3$) δ 143.9 (d, *J* = 12.2 Hz), 135.1 (d, *J* = 2.6 Hz), 134.1 (d, *J* = 10.4 Hz), 132.4 (d, *J* = 15.0 Hz), 131.1 (d, *J* = 120.3 Hz), 129.0, 128.5 (d, *J* = 13.3 Hz), 128.4, 128.0 (d, *J* = 138.3 Hz), 127.9, 127.0, 126.2 (d, *J* = 11.8 Hz), 68.1 (d, *J* = 2.9 Hz), 31.1 (d, *J* = 16.0 Hz), 30.4 (d, *J* = 1.5 Hz), 28.9 (d, *J* = 20.9 Hz), 22.4, 13.9.

Following general procedure, the recovered starting material ***(S)*-2b** was obtained in 43% isolated yield (25.8 mg), colorless oil. $[\alpha]^{24}_{D}$ = 60.87 (*c* = 1.0, $CHCl_3$) for 90% *ee*. HPLC analysis:

The enantiomeric excess was detected at 210 nm by Daicel Chiralcel IF (0.46 cm x 25 cm), ratio of hexanes/IPA = 70/30, flow = 1.0 mL/min, retention time: 16.0 min (minor), 14.2 min(major). HRMS (ESI) *m/z*: $[M+H]^+$ calcd for $C_{18}H_{22}PO_2$, 301.1357; found 301.1320.

(*S,E*)-2-(4-(Methylthio)phenyl)-3-pentylidene-1,2-oxaphospholane 2-oxide ((*S*)-2c)

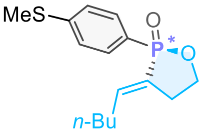

^{1}H NMR (600 MHz, $CDCl_3$) δ 7.65 – 7.57 (m, 2H), 7.24 (dd, *J* = 8.4, 3.0 Hz, 2H), 6.23 (dtt, *J* = 19.6, 7.2, 2.6 Hz, 1H), 4.52 – 4.34 (m, 2H), 2.98 – 2.72 (m, 2H), 2.46 (s, 3H), 2.19 – 2.07 (m, 2H), 1.39 – 1.23 (m, 4H), 0.84 (t, *J* = 7.3 Hz, 3H). ^{31}P NMR (243 MHz, $CDCl_3$) δ 42.53. ^{13}C NMR (150 MHz, $CDCl_3$) δ 145.0 (d, *J* = 3.2 Hz), 143.5 (d, *J* = 12.4 Hz), 131.9 (d, *J* = 11.5 Hz), 131.1 (d, *J* = 121.0 Hz), 126.5 (d, *J* = 141.7 Hz), 125.2 (d, *J* = 14.0 Hz), 67.8 (d, *J* = 2.9 Hz), 31.0 (d, *J* = 16.0 Hz), 30.3, 28.7 (d, *J* = 21.0 Hz), 22.3, 14.8, 13.8.

Following general procedure, the recovered starting material **(*S*)-2c** was obtained in 50% isolated yield (29.6 mg), colorless oil. $[\alpha]^{24}_D$ = 67.37 (*c* = 1.0, $CHCl_3$) for 89% *ee*. HPLC analysis: The enantiomeric excess was detected at 210 nm by CHIRALPAK® AD-H (0.46 cm x 25 cm), ratio of hexanes/IPA = 80/20, flow = 1.0 mL/min, retention time: 12.6 min (minor), 9.9 min(major). HRMS (ESI) *m/z*: $[M+H]^+$ calcd for $C_{15}H_{22}PSO_3$, 297.1078; found 297.1042.

(*S,E*)-2-(4-(Tert-butyl)phenyl)-3-pentylidene-1,2-oxaphospholane 2-oxide ((*S*)-2d)

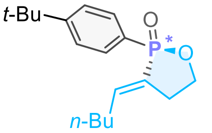

^{1}H NMR (600 MHz, $CDCl_3$) δ 7.71 – 7.62 (m, 2H), 7.48 – 7.41 (m, 2H), 6.27 (dtd, *J* = 19.3, 7.2, 3.3 Hz, 1H), 4.55 – 4.34 (m, 2H), 2.99 – 2.74 (m, 2H), 2.19 – 2.08 (m, 2H), 1.42 – 1.26 (m, 14H), 0.85 (t, *J* = 7.3 Hz, 3H). ^{31}P NMR (243 MHz, $CDCl_3$) δ 42.99. ^{13}C NMR (150 MHz, $CDCl_3$) δ 156.0 (d, *J* = 2.9 Hz), 143.5 (d, *J* = 12.1 Hz), 131.5 (d, *J* = 11.5 Hz), 131.1 (d, *J* = 120.1 Hz), 127.8 (d, *J* = 140.2 Hz), 125.6 (d, *J* = 14.0 Hz), 67.7 (d, *J* = 2.7 Hz), 35.1, 31.1, 31.0 (d, *J* = 16.1 Hz), 30.4, 28.8 (d, *J* = 21.0 Hz), 22.4, 13.9.

Following general procedure, the recovered starting material **(*S*)-2d** was obtained in 45% isolated yield (27.5 mg), colorless oil. $[\alpha]^{24}_{D}$ = 73.32 (*c* = 2.5, $CHCl_3$) for 88% *ee*. HPLC analysis: The enantiomeric excess was detected at 210 nm by CHIRALPAK® AD-H (0.46 cm x 25 cm), ratio of hexanes/IPA = 80/20, flow = 1.0 mL/min, retention time: 7.1 min (minor), 6.6 min(major). HRMS (ESI) *m/z*: $[M+H]^+$ calcd for $C_{18}H_{28}PO_2$, 307.1827; found 307.1789.

(*S,E*)-2-([1,1'-Biphenyl]-4-yl)-3-pentylidene-1,2-oxaphospholane 2-oxide ((*S*)-2e)

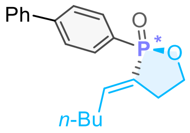

^1H NMR (600 MHz, $CDCl_3$) δ 7.87 – 7.77 (m, 2H), 7.66 (dd, *J* = 8.2, 3.2 Hz, 2H), 7.62 – 7.52 (m, 2H), 7.48 – 7.40 (m, 2H), 7.40 – 7.32 (m, 1H), 6.32 (dtt, *J* = 19.6, 7.3, 2.6 Hz, 1H), 4.59 – 4.37 (m, 2H), 3.03 – 2.75 (m, 2H), 2.24 – 2.10 (m, 2H), 1.43 – 1.25 (m, 4H), 0.86 (t, *J* = 7.3 Hz, 3H). ^{31}P NMR (243 MHz, $CDCl_3$) δ 42.69. ^{13}C NMR (150 MHz, $CDCl_3$) δ 145.2 (d, *J* = 3.0 Hz), 143.8 (d, *J* = 12.3 Hz), 139.9, 132.2 (d, *J* = 11.4 Hz), 131.0 (d, *J* = 120.5 Hz), 129.6 (d, *J* = 139.2 Hz), 129.0, 128.2, 127.3, 127.2 (d, *J* = 14.1 Hz), 67.9 (d, *J* = 2.8 Hz), 31.0 (d, *J* = 16.1 Hz), 30.3, 28.8 (d, *J* = 21.1 Hz), 22.4, 13.8.

Following general procedure, the recovered starting material **(*S*)-2e** was obtained in 52% isolated yield (33.9 mg), colorless oil. $[\alpha]^{24}_{D}$ = 64.17 (*c* = 2.5, $CHCl_3$) for 87% *ee*. HPLC analysis: The enantiomeric excess was detected at 254 nm by CHIRALPAK® AD-H (0.46 cm x 25 cm), ratio of hexanes/IPA = 80/20, flow = 1.0 mL/min, retention time: 10.7 min (minor), 13.1 min(major). HRMS (ESI) *m/z*: $[M+H]^+$ calcd for $C_{20}H_{24}PO_2$, 327.1514; found 327.1476.

(*S,E*)-2-(4-Fluorophenyl)-3-pentylidene-1,2-oxaphospholane 2-oxide ((*S*)-2f)

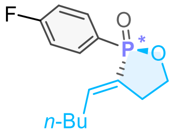

^1H NMR (600 MHz, $CDCl_3$) δ 7.81 – 7.64 (m, 2H), 7.10 (td, *J* = 8.7, 2.5 Hz, 2H), 6.24 (dtt, *J* = 19.6, 7.2, 2.6 Hz, 1H), 4.63 – 4.33 (m, 2H), 2.99 – 2.71 (m, 2H), 2.20 – 2.05 (m, 2H), 1.40 – 1.21 (m, 4H), 0.83 (t, *J* = 7.3 Hz, 3H). ^{31}P NMR (243 MHz, $CDCl_3$) δ 41.54. ^{19}F NMR (565 MHz,

$CDCl_3$) δ -106.05. ^{13}C NMR (150 MHz, $CDCl_3$) δ 165.4 (dd, *J* = 253.8, 3.4 Hz), 144.0 (d, *J* = 12.3 Hz), 134.2 (dd, *J* = 12.5, 9.0 Hz), 130.8 (d, *J* = 121.5 Hz), 127.1 (dd, *J* = 141.2, 3.3 Hz), 115.9 (dd, *J* = 21.6, 14.8 Hz), 67.9 (d, *J* = 2.9 Hz), 31.0 (d, *J* = 15.9 Hz), 30.3, 28.7 (d, *J* = 21.0 Hz), 22.3, 13.8.

Following general procedure, the recovered starting material **(*S*)-2f** was obtained in 51% isolated yield (27.3 mg), colorless oil. $[\alpha]^{24}_D$ = 54.69 (*c* = 2.5, $CHCl_3$) for 85% *ee*. HPLC analysis: The enantiomeric excess was detected at 254 nm by Daicel Chiralcel IF (0.46 cm x 25 cm), ratio of hexanes/IPA = 70/30, flow = 1.0 mL/min, retention time: 10.2 min (minor), 9.3 min(major). HRMS (ESI) *m/z*: $[M+H]^+$ calcd for $C_{14}H_{19}FPO_2$, 269.1107; found 269.1072.

Methyl (*S,E*)-4-(2-oxido-3-pentylidene-1,2-oxaphospholan-2-yl)benzoate ((*S*)-2g)

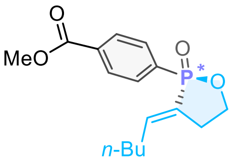

^1H NMR (600 MHz, $CDCl_3$) δ 8.06 (dd, *J* = 8.2, 3.2 Hz, 2H), 7.86 – 7.74 (m, 2H), 6.28 (dtt, *J* = 19.4, 7.1, 2.5 Hz, 1H), 4.56 – 4.38 (m, 2H), 3.89 (s, 3H), 3.01 – 2.74 (m, 2H), 2.19 – 2.08 (m, 2H), 1.39 – 1.22 (m, 4H), 0.83 (t, *J* = 7.3 Hz, 3H). ^{31}P NMR (243 MHz, $CDCl_3$) δ 41.29. ^{13}C NMR (150 MHz, $CDCl_3$) δ 166.2, 144.7 (d, *J* = 12.2 Hz), 136.1 (d, *J* = 134.8 Hz), 133.6 (d, *J* = 2.8 Hz), 131.6 (d, *J* = 11.0 Hz), 130.5 (d, *J* = 120.7 Hz), 129.5 (d, *J* = 13.4 Hz), 68.1 (d, *J* = 3.1 Hz), 52.5, 31.1 (d, *J* = 15.9 Hz), 30.3, 28.7 (d, *J* = 21.3 Hz), 22.3, 13.8.

Following general procedure, the recovered starting material **(*S*)-2g** was obtained in 48% isolated yield (29.6 mg), colorless oil. $[\alpha]^{23}_D$ = 54.07 (*c* = 1.0, $CHCl_3$) for 86% *ee*. HPLC analysis: The enantiomeric excess was detected at 254 nm by CHIRALPAK® AD-H (0.46 cm x 25 cm), ratio of hexanes/IPA = 80/20, flow = 1.0 mL/min, retention time: 15.1 min (minor), 13.0 min(major). HRMS (ESI) *m/z*: $[M+H]^+$ calcd for $C_{16}H_{22}PO_4$, 309.1256; found 309.1219.

(*S,E*)-2-(4-Methoxyphenyl)-3-pentylidene-1,2-oxaphospholane 2-oxide ((*S*)-2h)

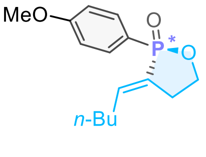

^{1}H NMR (600 MHz, $CDCl_3$) δ 7.69 – 7.60 (m, 2H), 6.95 – 6.88 (m, 2H), 6.20 (dtt, *J* = 19.6, 7.2, 2.6 Hz, 1H), 4.51 – 4.32 (m, 2H), 3.79 (s, 3H), 2.96 – 2.71 (m, 2H), 2.19 – 2.05 (m, 2H), 1.37 – 1.23 (m, 4H), 0.83 (t, *J* = 7.3 Hz, 3H). ^{31}P NMR (243 MHz, $CDCl_3$) δ 42.96. ^{13}C NMR (150 MHz, $CDCl_3$) δ 163.0 (d, *J* = 3.1 Hz), 143.1 (d, *J* = 12.3 Hz), 133.6 (d, *J* = 12.4 Hz), 131.3 (d, *J* = 121.0 Hz), 122.0 (d, *J* = 145.0 Hz), 114.1 (d, *J* = 14.7 Hz), 67.6 (d, *J* = 2.8 Hz), 55.4, 30.9 (d, *J* = 15.9 Hz), 30.3 (d, *J* = 1.6 Hz), 28.7 (d, *J* = 20.8 Hz), 22.3, 13.8.

Following general procedure, the recovered starting material **(*S*)-2h** was obtained in 51% isolated yield (28.6 mg), colorless oil. $[\alpha]^{24}_{D}$ = 62.97 (*c* = 2.5, $CHCl_3$) for 85% *ee*. HPLC analysis: The enantiomeric excess was detected at 210 nm by CHIRALPAK® AD-H (0.46 cm x 25 cm), ratio of hexanes/IPA = 80/20, flow = 1.0 mL/min, retention time: 13.0 min (minor), 9.2 min(major). HRMS (ESI) *m/z*: $[M+H]^+$ calcd for $C_{15}H_{22}PO_3$, 281.1307; found 281.1269.

(*S,E*)-2-(4-Chlorophenyl)-3-pentylidene-1,2-oxaphospholane 2-oxide ((*S*)-2i)

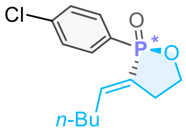

^{1}H NMR (600 MHz, $CDCl_3$) δ 7.66 (dd, *J* = 12.5, 8.4 Hz, 2H), 7.47 – 7.36 (m, 2H), 6.26 (dtt, *J* = 19.7, 7.2, 2.6 Hz, 1H), 4.56 – 4.35 (m, 2H), 2.99 – 2.72 (m, 2H), 2.21 – 2.07 (m, 2H), 1.39 – 1.22 (m, 4H), 0.84 (t, *J* = 7.3 Hz, 3H). ^{31}P NMR (243 MHz, $CDCl_3$) δ 41.55. ^{13}C NMR (150 MHz, $CDCl_3$) δ 144.3 (d, *J* = 12.1 Hz), 139.1 (d, *J* = 3.7 Hz), 133.1 (d, *J* = 11.9 Hz), 130.6 (d, *J* = 121.5 Hz), 129.7 (d, *J* = 139.3 Hz), 128.9 (d, *J* = 14.2 Hz), 68.0 (d, *J* = 3.0 Hz), 31.1 (d, *J* = 16.0 Hz), 30.3, 28.7 (d, *J* = 21.3 Hz), 22.4, 13.8.

Following general procedure, the recovered starting material **(*S*)-2i** was obtained in 45% isolated yield (25.6 mg), colorless oil. $[\alpha]^{24}_{D}$ = 62.89 (*c* = 2.5, $CHCl_3$) for 85% *ee*. HPLC analysis: The enantiomeric excess was detected at 254 nm by CHIRALPAK® AD-H (0.46 cm x 25 cm), ratio of hexanes/IPA = 80/20, flow = 1.0 mL/min, retention time: 10.4 min (minor), 7.4 min(major). HRMS (ESI) *m/z*: $[M+H]^+$ calcd for $C_{14}H_{19}ClPO_2$, 285.0811; found 285.0778.

(*S,E*)-2-(4-Bromophenyl)-3-pentylidene-1,2-oxaphospholane 2-oxide ((*S*)-2j)

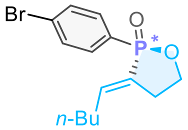

^{1}H NMR (600 MHz, $CDCl_3$) δ 7.66 – 7.52 (m, 4H), 6.27 (dtt, *J* = 19.7, 7.2, 2.6 Hz, 1H), 4.56 – 4.37 (m, 2H), 3.00 – 2.71 (m, 2H), 2.21 – 2.09 (m, 2H), 1.40 – 1.23 (m, 4H), 0.85 (t, *J* = 7.3 Hz, 3H). ^{31}P NMR (243 MHz, $CDCl_3$) δ 41.64. ^{13}C NMR (150 MHz, $CDCl_3$) δ 144.3 (d, *J* = 12.2 Hz), 133.2 (d, *J* = 11.9 Hz), 131.9 (d, *J* = 14.1 Hz), 130.6 (d, *J* = 121.1 Hz), 130.2 (d, *J* = 138.6 Hz), 127.7 (d, *J* = 3.5 Hz), 68.0 (d, *J* = 3.1 Hz), 31.1 (d, *J* = 16.1 Hz), 30.3, 28.7 (d, *J* = 21.2 Hz), 22.4, 13.9.

Following general procedure, the recovered starting material **(*S*)-2j** was obtained in 48% isolated yield (31.5 mg), colorless oil. $[\alpha]^{24}_{D}$ = 81.20 (*c* = 2.5, $CHCl_3$) for 84% *ee*. HPLC analysis: The enantiomeric excess was detected at 210 nm by CHIRALPAK® AD-H (0.46 cm x 25 cm), ratio of hexanes/IPA = 80/20, flow = 1.0 mL/min, retention time: 11.5 min (minor), 8.0 min(major). HRMS (ESI) *m/z*: $[M+H]^+$ calcd for $C_{14}H_{19}BrPO_2$, 329.0306; found 329.0269.

(*S,E*)-2-(3-Bromophenyl)-3-pentylidene-1,2-oxaphospholane 2-oxide ((*S*)-2k)

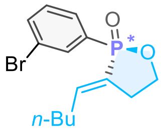

^{1}H NMR (600 MHz, $CDCl_3$) δ 7.85 (dt, *J* = 13.1, 1.7 Hz, 1H), 7.74 – 7.63 (m, 2H), 7.33 (td, *J* = 7.8, 4.0 Hz, 1H), 6.32 (dtt, *J* = 19.7, 7.2, 2.6 Hz, 1H), 4.58 – 4.39 (m, 2H), 3.02 – 2.75 (m, 2H), 2.23 – 2.12 (m, 2H), 1.43 – 1.26 (m, 4H), 0.87 (t, *J* = 7.3 Hz, 3H). ^{31}P NMR (243 MHz, $CDCl_3$) δ 40.52. ^{13}C NMR (150 MHz, $CDCl_3$) δ 144.7 (d, *J* = 12.0 Hz), 135.5 (d, *J* = 2.8 Hz), 134.4 (d, *J* = 11.9 Hz), 133.9 (d, *J* = 135.2 Hz), 130.4 (d, *J* = 121.1 Hz), 130.3 (d, *J* = 10.5 Hz), 130.3 (d, *J* = 6.6 Hz), 123.1 (d, *J* = 17.6 Hz), 68.2 (d, *J* = 3.2 Hz), 31.2 (d, *J* = 16.2 Hz), 30.4, 28.8 (d, *J* = 21.5 Hz), 22.5, 13.9.

Following general procedure, the recovered starting material **(*S*)-2k** was obtained in 42% isolated yield (27.6 mg), colorless oil. $[\alpha]^{23}_{D}$ = 41.88 (*c* = 1.0, $CHCl_3$) for 86% *ee*. HPLC analysis: The enantiomeric excess was detected at 210 nm by Daicel Chiralcel ID (0.46 cm x 25 cm),

ratio of hexanes/IPA = 80/20, flow = 1.0 mL/min, retention time: 19.8 min (minor), 18.4 min(major). HRMS (ESI) *m/z*: $[M+H]^+$ calcd for $C_{14}H_{19}BrPO_2$, 329.0306; found 329.0269.

(*S,E*)-2-(3-Methoxyphenyl)-3-pentylidene-1,2-oxaphospholane 2-oxide ((*S*)-2l)

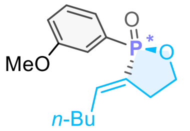

^{1}H NMR (600 MHz, $CDCl_3$) δ 7.38 – 7.23 (m, 3H), 7.05 (ddt, *J* = 8.3, 2.7, 1.0 Hz, 1H), 6.30 (dtt, *J* = 19.6, 7.3, 2.7 Hz, 1H), 4.55 – 4.37 (m, 2H), 3.82 (s, 3H), 3.00 – 2.75 (m, 2H), 2.22 – 2.10 (m, 2H), 1.41 – 1.27 (m, 4H), 0.86 (t, *J* = 7.3 Hz, 3H). ^{31}P NMR (243 MHz, $CDCl_3$) δ 42.66 ^{13}C NMR (150 MHz, $CDCl_3$) δ 159.5 (d, *J* = 16.8 Hz), 143.8 (d, *J* = 12.0 Hz), 132.5 (d, *J* = 136.8 Hz), 130.8 (d, *J* = 120.4 Hz), 129.8 (d, *J* = 16.0 Hz), 123.7 (d, *J* = 10.9 Hz), 118.7 (d, *J* = 2.8 Hz), 116.4 (d, *J* = 12.0 Hz), 67.9 (d, *J* = 2.8 Hz), 55.5, 31.0 (d, *J* = 15.9 Hz), 30.3 (d, *J* = 1.6 Hz), 28.7 (d, *J* = 21.2 Hz), 22.3, 13.8.

Following general procedure, the recovered starting material **(*S*)-2l** was obtained in 48% isolated yield (26.9 mg), colorless oil. $[\alpha]^{24}_{D}$ = 56.07 (*c* = 2.0, $CHCl_3$) for 83% *ee*. HPLC analysis: The enantiomeric excess was detected at 210 nm by CHIRALCEL® OD-H (0.46 cm x 25 cm), ratio of hexanes/IPA = 85/15, flow = 1.0 mL/min, retention time: 11.4 min (minor), 9.8 min(major). HRMS (ESI) *m/z*: $[M+H]^+$ calcd for $C_{15}H_{22}PO_3$, 281.1307; found 281.1269.

(*S,E*)-2-(3,5-Dimethylphenyl)-3-pentylidene-1,2-oxaphospholane 2-oxide ((*S*)-2m)

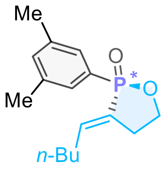

^{1}H NMR (400 MHz, $CDCl_3$) δ 7.42 – 7.28 (m, 2H), 7.19 – 7.03 (m, 1H), 6.25 (dtt, *J* = 19.5, 7.3, 2.6 Hz, 1H), 4.57 – 4.30 (m, 2H), 2.99 – 2.72 (m, 2H), 2.31 (s, 6H), 2.18 – 2.07 (m, 2H), 1.43 – 1.22 (m, 4H), 0.85 (t, *J* = 7.2 Hz, 3H). ^{31}P NMR (162 MHz, $CDCl_3$) δ 43.31. ^{13}C NMR (100 MHz, $CDCl_3$) δ 143.4 (d, *J* = 12.3 Hz), 138.3 (d, *J* = 14.3 Hz), 134.2 (d, *J* = 3.1 Hz), 131.1 (d, *J* =

119.6 Hz), 130.7 (d, J = 136.8 Hz), 129.2 (d, J = 11.0 Hz), 67.8 (d, J = 2.6 Hz), 31.0 (d, J = 15.9 Hz), 30.4 (d, J = 1.5 Hz), 28.8 (d, J = 20.7 Hz), 22.4, 21.2, 13.8.

Following general procedure, the recovered starting material **(*S*)-2m** was obtained in 45% isolated yield (25.0 mg), colorless oil. $[\alpha]^{22}_D$ = 30.24 (c = 1.0, $CHCl_3$) for 86% *ee*. HPLC analysis: The enantiomeric excess was detected at 254 nm by CHIRALCEL® OD-H (0.46 cm x 25 cm), ratio of hexanes/IPA = 80/20, flow = 1.0 mL/min, retention time: 7.8 min (minor), 6.5 min(major). HRMS (ESI) *m/z*: $[M+H]^+$ calcd for $C_{16}H_{24}PO_2$, 279.1514; found 279.1486.

(*S,E*)-3-Pentylidene-2-(thiophen-3-yl)-1,2-oxaphospholane 2-oxide ((*S*)-2n)

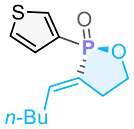

^{1}H NMR (600 MHz, $CDCl_3$) δ 7.95 (ddd, J = 7.9, 2.9, 1.2 Hz, 1H), 7.39 (dt, J = 5.0, 2.8 Hz, 1H), 7.21 (ddd, J = 5.1, 4.0, 1.2 Hz, 1H), 6.31 (dtt, J = 19.7, 7.3, 2.6 Hz, 1H), 4.51 – 4.30 (m, 2H), 2.97 – 2.72 (m, 2H), 2.18 – 2.11 (m, 2H), 1.40 – 1.26 (m, 4H), 0.85 (t, J = 7.3 Hz, 3H). ^{31}P NMR (243 MHz, $CDCl_3$) δ 36.50. ^{13}C NMR (150 MHz, $CDCl_3$) δ 143.7 (d, J = 12.3 Hz), 135.3 (d, J = 16.8 Hz), 132.5 (d, J = 143.8 Hz), 130.9 (d, J = 123.7 Hz), 128.7 (d, J = 17.4 Hz), 127.5 (d, J = 17.4 Hz), 67.6 (d, J = 3.7 Hz), 31.0 (d, J = 16.2 Hz), 30.4, 28.4 (d, J = 22.0 Hz), 22.4, 13.9.

Following general procedure, the recovered starting material **(*S*)-2n** was obtained in 44% isolated yield (22.5 mg), colorless oil. $[\alpha]^{24}_D$ = 43.98 (c = 1.0, $CHCl_3$) for 89% *ee*. HPLC analysis: The enantiomeric excess was detected at 254 nm by CHIRALPAK® AD-H (0.46 cm x 25 cm), ratio of hexanes/IPA = 85/15, flow = 1.0 mL/min, retention time: 14.4 min (minor), 12.7 min(major). HRMS (ESI) *m/z*: $[M+H]^+$ calcd for $C_{12}H_{18}PSO_2$, 257.0765; found 257.0732.

(*S,E*)-3-Pentylidene-2-(1-phenylvinyl)-1,2-oxaphospholane 2-oxide ((*S*)-2o)

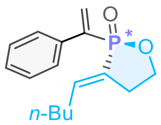

^{1}H NMR (600 MHz, $CDCl_3$) δ 7.41 – 7.27 (m, 5H), 6.40 (dd, J = 20.5, 1.7 Hz, 1H), 6.34 – 6.24 (m, 1H), 6.06 (dd, J = 41.5, 1.7 Hz, 1H), 4.47 – 4.34 (m, 1H), 4.16 – 4.01 (m, 1H), 2.82 – 2.71

(m, 1H), 2.42 – 2.29 (m, 1H), 2.15 – 2.01 (m, 2H), 1.36 – 1.19 (m, 4H), 0.85 (t, *J* = 7.3 Hz, 3H). ^{31}P NMR (243 MHz, $CDCl_3$) δ 41.11. ^{13}C NMR (150 MHz, $CDCl_3$) δ 143.9 (d, *J* = 12.0 Hz), 142.76 (d, *J* = 122.6 Hz), 137.29 (d, *J* = 12.2 Hz), 132.19 (d, *J* = 9.0 Hz), 129.61 (d, *J* = 118.8 Hz), 128.71 (d, *J* = 107.3 Hz), 128.35 (d, *J* = 37.9 Hz), 128.27 (d, *J* = 4.6 Hz), 67.83 (d, *J* = 2.6 Hz), 30.98 (d, *J* = 15.9 Hz), 30.36, 28.33 (d, *J* = 21.2 Hz), 22.30, 13.92.

Following general procedure, the recovered starting material **(*S*)-2o** was obtained in 41% isolated yield (22.6 mg), colorless oil. $[\alpha]^{24}_D$ = 22.95 (*c* = 2.0, $CHCl_3$) for 83% *ee*. HPLC analysis: The enantiomeric excess was detected at 210 nm by Daicel Chiralcel ID (0.46 cm x 25 cm), ratio of hexanes/IPA = 80/20, flow = 1.0 mL/min, retention time: 18.6 min (minor), 17.4 min(major). HRMS (ESI) *m/z*: $[M+H]^+$ calcd for $C_{16}H_{22}PO_2$, 277.1357; found 277.1323.

(*S,E*)-3-methylene-2-phenyl-1,2-oxaphospholane 2-oxide ((*S*)-2p)

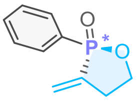

H NMR (600 MHz, $CDCl_3$) δ 7.87 – 7.70 (m, 2H), 7.68 – 7.53 (m, 1H), 7.53 – 7.42 (m, 2H), 5.72 – 5.58 (m, 1H), 5.50 – 5.35 (m, 1H), 4.66 – 4.52 (m, 1H), 4.27 – 4.14 (m, 1H), 2.98 – 2.82 (m, 1H), 2.63 – 2.47 (m, 1H). ^{31}P NMR (243 MHz, $CDCl_3$) δ 31.69. ^{13}C NMR (150 MHz, $CDCl_3$) δ 141.0 (d, *J* = 113.7 Hz), 132.8 (d, *J* = 2.8 Hz), 132.0 (d, *J* = 10.5 Hz), 128.7 (d, *J* = 137.5 Hz), 128.6 (d, *J* = 13.4 Hz), 125.8 (d, *J* = 10.2 Hz), 67.6 (d, *J* = 6.7 Hz), 32.9 (d, *J* = 7.1 Hz), 28.7 (d, *J* = 4.8 Hz).

Following general procedure, the recovered starting material **(*S*)-2p** was obtained in 38% isolated yield (14.7 mg), colorless oil. $[\alpha]^{23}_D$ = 8.23 (*c* = 0.5, $CHCl_3$) for 63% *ee*. HPLC analysis: The enantiomeric excess was detected at 210 nm by CHIRALPAK® OD-H (0.46 cm x 25 cm), ratio of hexanes/IPA = 90/10, flow = 1.0 mL/min, retention time: 27.2 min (minor), 28.5 min(major). HRMS (ESI) *m/z*: $[M+H]^+$ calcd for $C_{10}H_{12}PO_2$, 195.0575; found 195.0542.

(*S,E*)-2-phenyl-3-propylidene-1,2-oxaphospholane 2-oxide ((*S*)-2q)

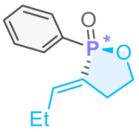

^1H NMR (400 MHz, $CDCl_3$) δ 7.84 – 7.65 (m, 2H), 7.54 – 7.34 (m, 3H), 6.36 – 6.11 (m, 1H), 4.61 – 4.25 (m, 2H), 3.02 – 2.67 (m, 2H), 2.27 – 2.00 (m, 2H), 0.96 (t, *J* = 7.6 Hz, 3H). ^{31}P NMR (162 MHz, $CDCl_3$) δ 42.69. ^{13}C NMR (100 MHz, $CDCl_3$) δ 144.9 (d, *J* = 12.0 Hz), 132.4 (d, *J* = 3.0 Hz), 131.5 (d, *J* = 11.0 Hz), 131.0 (d, *J* = 137.7 Hz), 130.4 (d, *J* = 120.2 Hz), 128.5 (d, *J* = 13.6 Hz), 67.8 (d, *J* = 2.9 Hz), 28.5 (d, *J* = 20.9 Hz), 24.6 (d, *J* = 16.3 Hz), 12.7 (d, *J* = 1.6 Hz).

Following general procedure, the recovered starting material **(*S*)-2q** was obtained in 47% isolated yield (20.9 mg), colorless oil. $[\alpha]^{24}_D$ = 75.12 (*c* = 2.5, $CHCl_3$) for 86% *ee*. HPLC analysis: The enantiomeric excess was detected at 210 nm by CHIRALPAK® AD-H (0.46 cm x 25 cm), ratio of hexanes/IPA = 80/20, flow = 1.0 mL/min, retention time: 7.9 min (minor), 7.4 min(major). HRMS (ESI) *m/z*: $[M+H]^+$ calcd for $C_{12}H_{16}PO_2$, 223.0888; found 223.0861.

(*S,E*)-3-Butylidene-2-phenyl-1,2-oxaphospholane 2-oxide ((*S*)-2r)

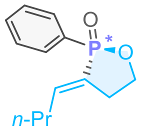

^1H NMR (400 MHz, $CDCl_3$) δ 7.82 – 7.68 (m, 2H), 7.56 – 7.48 (m, 1H), 7.48 – 7.38 (m, 2H), 6.36 – 6.17 (m, 1H), 4.59 – 4.33 (m, 2H), 3.03 – 2.71 (m, 2H), 2.22 – 2.03 (m, 2H), 1.53 – 1.32 (m, 2H), 0.87 (t, *J* = 7.4 Hz, 3H). ^{31}P NMR (162 MHz, $CDCl_3$) δ 42.63, 40.26. ^{13}C NMR (100 MHz, $CDCl_3$) δ 143.6 (d, *J* = 12.2 Hz), 132.5 (d, *J* = 2.9 Hz), 131.6 (d, *J* = 11.0 Hz), 131.2 (d, *J* = 120.2 Hz), 131.1 (d, *J* = 137.7 Hz), 128. 6 (d, *J* = 13.5 Hz), 67.9 (d, *J* = 2.9 Hz), 33.3 (d, *J* = 16.0 Hz), 28.8 (d, *J* = 20.9 Hz), 21.6 (d, *J* = 1.5 Hz), 13.8.

Following general procedure, the recovered starting material **(*S*)-2r** was obtained in 47% isolated yield (22.2 mg), Light yellow oil. $[\alpha]^{23}_D$ = 68.97 (*c* = 0.5, $CHCl_3$) for 87% *ee*. HPLC analysis: The enantiomeric excess was detected at 210 nm by CHIRALPAK® AD-H (0.46 cm x 25 cm), ratio of hexanes/IPA = 80/20, flow = 1.0 mL/min, retention time: 8.4 min (minor), 7.3 min(major). HRMS (ESI) *m/z*: $[M+H]^+$ calcd for $C_{13}H_{18}PO_2$, 237.1044; found 237.1005.

(*S,E*)-3-nonylidene-2-phenyl-1,2-oxaphospholane 2-oxide ((*S*)-2s)

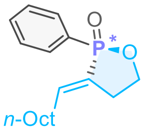

^{1}H NMR (400 MHz, $CDCl_3$) δ 7.81 – 7.67 (m, 2H), 7.55 – 7.48 (m, 1H), 7.47 – 7.40 (m, 2H), 6.27 (dtt, *J* = 19.6, 7.2, 2.6 Hz, 1H), 4.62 – 4.31 (m, 2H), 3.05 – 2.68 (m, 2H), 2.21 – 2.06 (m, 2H), 1.46 – 1.33 (m, 2H), 1.33 – 1.12 (m, 10H), 0.84 (t, *J* = 6.9 Hz, 3H). ^{31}P NMR (162 MHz, $CDCl_3$) δ 42.62. ^{13}C NMR (100 MHz, $CDCl_3$) δ 143.9 (d, *J* = 12.1 Hz), 132.4 (d, *J* = 2.9 Hz), 131.6 (d, *J* = 11.0 Hz), 131.1 (d, *J* = 137.7 Hz), 130.9 (d, *J* = 120.1 Hz), 128.5 (d, *J* = 13.6 Hz), 67.9 (d, *J* = 2.8 Hz), 31.8, 31.4 (d, *J* = 15.9 Hz), 29.4, 29.3, 29.2, 28.8 (d, *J* = 20.9 Hz), 28.3 (d, *J* = 1.5 Hz), 22.7, 14.1.

Following general procedure, the recovered starting material **(*S*)-2s** was obtained in 42% isolated yield (25.7 mg), Light yellow oil. $[\alpha]^{23}_{D}$ = 77.11 (*c* = 2.0, $CHCl_3$) for 90% *ee*. HPLC analysis: The enantiomeric excess was detected at 210 nm by CHIRALPAK® OD-H (0.46 cm x 25 cm), ratio of hexanes/IPA = 85/15, flow = 1.0 mL/min, retention time: 8.6 min (minor), 9.6 min(major). HRMS (ESI) *m/z*: $[M+H]^+$ calcd for $C_{18}H_{28}PO_2$, 307.1827; found 307.1789.

(*S,E*)-2-Phenyl-3-(2-phenylethylidene)-1,2-oxaphospholane 2-oxide ((*S*)-2t)

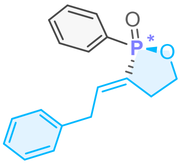

^{1}H NMR (400 MHz, $CDCl_3$) δ 7.83 – 7.73 (m, 2H), 7.58 – 7.51 (m, 1H), 7.51 – 7.41 (m, 2H), 7.31 – 7.15 (m, 3H), 7.15 – 7.07 (m, 2H), 6.52 – 6.41 (m, 1H), 4.65 – 4.36 (m, 2H), 3.62 – 3.41 (m, 2H), 3.14 – 2.79 (m, 2H). ^{31}P NMR (162 MHz, $CDCl_3$) δ 42.33. ^{13}C NMR (100 MHz, $CDCl_3$) δ 141.3 (d, *J* = 12.9 Hz), 137.9, 132.6 (d, *J* = 2.9 Hz), 132.4 (d, *J* = 118.0 Hz), 131.7 (d, *J* = 10.8 Hz), 130.8 (d, *J* = 138.3 Hz), 128.8, 128.6 (d, *J* = 13.6 Hz), 128.5, 126.7, 67.9 (d, *J* = 2.5 Hz), 37.4 (d, *J* = 16.5 Hz), 29.0 (d, *J* = 20.5 Hz).

Following general procedure, the recovered starting material **(*S*)-2t** was obtained in 48% isolated yield (27.3 mg), Light yellow oil. $[\alpha]^{24}_{D}$ = 44.73 (*c* = 2.0, $CHCl_3$) for 88% *ee*. HPLC analysis: The enantiomeric excess was detected at 210 nm by CHIRALPAK® AD-H (0.46 cm x

25 cm), ratio of hexanes/IPA = 85/15, flow = 1.0 mL/min, retention time: 24.7 min (minor), 16.4 min(major). HRMS (ESI) *m/z*: $[M+H]^+$ calcd for $C_{17}H_{18}PO_2$, 285.1044; found 285.1009.

(*S,E*)-2-phenyl-3-(3-phenylpropylidene)-1,2-oxaphospholane 2-oxide ((*S*)-2u)

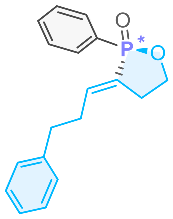

^{1}H NMR (400 MHz, $CDCl_3$) δ 7.77 – 7.62 (m, 2H), 7.58 – 7.51 (m, 1H), 7.51 – 7.40 (m, 2H), 7.25 – 7.13 (m, 3H), 7.13 – 6.98 (m, 2H), 6.38 – 6.18 (m, 1H), 4.48 – 4.16 (m, 2H), 2.83 – 2.39 (m, 6H). ^{31}P NMR (162 MHz, $CDCl_3$) δ 42.47. ^{13}C NMR (100 MHz, $CDCl_3$) δ 141.9 (d, *J* = 12.4 Hz), 140.7, 132.4 (d, *J* = 2.9 Hz), 132.2 (d, *J* = 119.5 Hz), 131.6 (d, *J* = 11.2 Hz), 130.9 (d, *J* = 138.3 Hz), 128.5 (d, *J* = 13.3 Hz), 128.5, 128.4, 126.2, 67.8 (d, *J* = 2.7 Hz), 34.3 (d, *J* = 1.7 Hz), 33.2 (d, *J* = 16.2 Hz), 28.6 (d, *J* = 20.8 Hz).

Following general procedure, the recovered starting material **(*S*)-2u** was obtained in 49% isolated yield (29.2 mg), Light yellow oil. $[\alpha]^{24}_D$ = 20.79 (*c* = 2.0, $CHCl_3$) for 85% *ee*. HPLC analysis: The enantiomeric excess was detected at 210 nm by Daicel Chiralcel IH (0.46 cm x 25 cm), ratio of hexanes/IPA = 60/40, flow = 1.0 mL/min, retention time: 25.2 min (minor), 21.8 min(major). HRMS (ESI) *m/z*: $[M+H]^+$ calcd for $C_{18}H_{19}PO_2$, 299.1201; found 299.1164.

(*S,E*)-3-(4-Chlorobutylidene)-2-phenyl-1,2-oxaphospholane 2-oxide ((*S*)-2v)

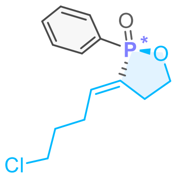

^{1}H NMR (600 MHz, $CDCl_3$) δ 7.90 – 7.65 (m, 2H), 7.60 – 7.40 (m, 3H), 6.21 (ddq, *J* = 19.3, 7.2, 4.5, 3.5 Hz, 1H), 4.61 – 4.37 (m, 2H), 3.60 – 3.43 (m, 2H), 3.09 – 2.77 (m, 2H), 2.46 – 2.27 (m, 2H), 1.97 – 1.78 (m, 2H). ^{31}P NMR (243 MHz, $CDCl_3$) δ 42.16. ^{13}C NMR (100 MHz, $CDCl_3$) δ 141.1 (d, *J* = 12.5 Hz), 133.2 (d, *J* = 119.2 Hz), 132.6 (d, *J* = 3.0 Hz), 131.7 (d, *J* = 11.1 Hz),

130.9 (d, *J* = 138.3 Hz), 128.7 (d, *J* = 13.6 Hz), 68.0 (d, *J* = 2.6 Hz), 44.2, 30.8, 28.9 (d, *J* = 20.4 Hz), 28.2 (d, *J* = 16.2 Hz).

Following general procedure, the recovered starting material **(*S*)-2v** was obtained in 45% isolated yield (24.3 mg), colorless oil. $[\alpha]^{24}_D$ = 27.75 (*c* = 2.5, $CHCl_3$) for 90% *ee*. HPLC analysis: The enantiomeric excess was detected at 210 nm by CHIRALPAK® AD-H (0.46 cm x 25 cm), ratio of hexanes/IPA = 70/30, flow = 1.0 mL/min, retention time: 7.9 min (minor), 6.9 min(major). HRMS (ESI) *m/z*: $[M+H]^+$ calcd for $C_{13}H_{17}ClPO_2$, 271.0655; found 271.0621.

(*S,E*)-3-(cyclohexylmethylene)-2-phenyl-1,2-oxaphospholane 2-oxide ((*S*)-2w)

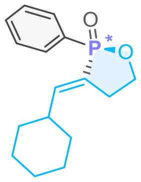

^1H NMR (400 MHz, $CDCl_3$) δ 7.86 – 7.64 (m, 2H), 7.58 – 7.34 (m, 3H), 6.20 – 5.94 (m, 1H), 4.59 – 4.29 (m, 2H), 3.08 – 2.72 (m, 2H), 2.29 – 2.12 (m, 1H), 1.81 – 1.47 (m, 5H), 1.32 – 0.98 (m, 5H). ^{31}P NMR (162 MHz, $CDCl_3$) δ 43.10, 40.00. ^{13}C NMR (100 MHz, $CDCl_3$) δ 148.56 (d, *J* = 11.2 Hz), 132.39 (d, *J* = 2.9 Hz), 131.59 (d, *J* = 11.0 Hz), 131.08 (d, *J* = 119.9 Hz), 128.86 (d, *J* = 119.6 Hz), 128.51 (d, *J* = 13.6 Hz), 67.87 (d, *J* = 3.0 Hz), 40.56 (d, *J* = 15.5 Hz), 31.67 (d, *J* = 1.9 Hz), 31.62 (d, *J* = 1.4 Hz), 28.61 (d, *J* = 21.1 Hz), 25.74, 25.49, 25.49.

Following general procedure, the recovered starting material **(*S*)-2w** was obtained in 45% isolated yield (24.3 mg), Light yellow oil. $[\alpha]^{23}_D$ = 32.68 (*c* = 1.0, $CHCl_3$) for 90% *ee*. HPLC analysis: The enantiomeric excess was detected at 210 nm by CHIRALPAK® OD-H (0.46 cm x 25 cm), ratio of hexanes/IPA = 85/15, flow = 1.0 mL/min, retention time: 10.3 min (minor), 11.0 min(major). HRMS (ESI) *m/z*: $[M+H]^+$ calcd for $C_{16}H_{22}PO_2$, 277.1357; found 277.1327.

(*S,E*)-3-benzylidene-2-phenyl-1,2-oxaphospholane 2-oxide ((*S*)-2x)

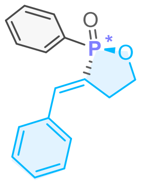

^{1}H NMR (600 MHz, $CDCl_3$) δ 7.89 – 7.78 (m, 2H), 7.59 – 7.53 (m, 1H), 7.48 (td, *J* = 7.7, 3.6 Hz, 2H), 7.42 – 7.31 (m, 5H), 7.15 (dt, *J* = 20.7, 2.7 Hz, 1H), 4.64 – 4.48 (m, 2H), 3.39 – 3.14 (m, 2H). ^{31}P NMR (243 MHz, $CDCl_3$) δ 45.15. ^{13}C NMR (150 MHz, $CDCl_3$) δ 139.6 (d, *J* = 14.1 Hz), 135.8 (d, *J* = 19.7 Hz), 132.7 (d, *J* = 3.0 Hz), 131.8 (d, *J* = 11.0 Hz), 131.5 (d, *J* = 12.5 Hz), 130.6 (d, *J* = 32.2 Hz), 129.4, 129.3, 128.8, 128.7, 68.1 (d, *J* = 3.3 Hz), 30.6 (d, *J* = 19.3 Hz).

Following general procedure, the recovered starting material **(*S*)-2x** was obtained in 49% isolated yield (26.5 mg), Light yellow oil. $[\alpha]^{23}_{D}$ = -26.39 (*c* = 0.5, $CHCl_3$) for 79% *ee*. HPLC analysis: The enantiomeric excess was detected at 254 nm by CHIRALPAK® AD-H (0.46 cm x 25 cm), ratio of hexanes/IPA = 60/40, flow = 1.0 mL/min, retention time: 9.8 min (minor), 8.1 min(major). HRMS (ESI) *m/z*: $[M+H]^+$ calcd for $C_{16}H_{16}PO_2$, 271.0888; found 271.0853.

(*S,E*)-3-(4-Fluorobenzylidene)-2-phenyl-1,2-oxaphospholane 2-oxide ((*S*)-2y)

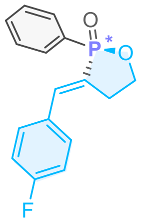

^{1}H NMR (600 MHz, $CDCl_3$) δ 7.83 (dd, *J* = 13.0, 7.6 Hz, 2H), 7.59 – 7.54 (m, 1H), 7.52 – 7.45 (m, 2H), 7.36 (dd, *J* = 8.5, 5.3 Hz, 2H), 7.16 – 7.04 (m, 3H), 4.65 – 4.49 (m, 2H), 3.34 – 3.11 (m, 2H). ^{31}P NMR (243 MHz, $CDCl_3$) δ 45.15. ^{19}F NMR (565 MHz, $CDCl_3$) δ -110.60. ^{13}C NMR (150 MHz, $CDCl_3$) δ 163.0 (d, *J* = 250.8 Hz), 138.4 (d, *J* = 14.3 Hz), 132.8 (d, *J* = 2.9 Hz), 132.1 (dd, *J* = 20.2, 3.4 Hz), 131.9 (d, *J* = 10.9 Hz), 131.3 (d, *J* = 8.2 Hz), 130.8 (dd, *J* = 119.2, 1.8 Hz), 130.4, 128.8 (d, *J* = 13.7 Hz), 116.0 (d, *J* = 21.8 Hz), 68.1 (d, *J* = 3.2 Hz), 30.5 (d, *J* = 19.1 Hz).

Following general procedure, the recovered starting material **(*S*)-2y** was obtained in 17% isolated yield (9.8 mg), Light yellow oil. $[\alpha]^{23}_{D}$ = -10.99 (*c* = 0.3, $CHCl_3$) for 81% *ee*. HPLC analysis: The enantiomeric excess was detected at 254 nm by CHIRALPAK® AD-H (0.46 cm x 25 cm), ratio of hexanes/IPA = 60/40, flow = 1.0 mL/min, retention time: 10.7 min (minor), 8.7 min(major). HRMS (ESI) *m/z*: $[M+H]^+$ calcd for $C_{16}H_{15}PFO_2$, 289.0794; found 289.0759.

Procedure for (*S*)-3 with methylmagnesium bromide (MeMgBr): To -10°C, magnetically stirred solution of **(*S*)-2j** (0.2 mmol) in dry Et_2O (1 ml) was added MeMgBr (0.3 mL, 0.3 mmol) dropwise. The reaction mixture was then allowed to warm to rt slowly over a period of 5 hours. The reaction mixture was quenched using saturated aq NH_4Cl (2 mL) and extracted with DCM (3x5 mL). The organic layer was dried over anhydrous Na2SO4, filtered, and concentrated under vacuum. The residue was purified by column chromatography on silica gel to get the tertiary P-stereogenic phosphine oxide **(*S*)-3** in colorless oil.

(*S,E*)-(4-Bromophenyl)(1-hydroxyoct-3-en-3-yl)(methyl)phosphine oxide ((*S*)-3)

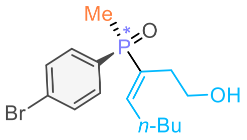

^1H NMR (600 MHz, $CDCl_3$) δ 7.73 – 7.34 (m, 4H), 6.22 (dt, *J* = 22.1, 7.3 Hz, 1H), 3.74 – 3.47 (m, 2H), 2.72 – 2.49 (m, 1H), 2.36 – 2.25 (m, 1H), 2.25 – 2.11 (m, 2H), 1.81 (d, *J* = 13.0 Hz, 3H), 1.47 – 1.27 (m, 4H), 0.90 (t, *J* = 7.4 Hz, 3H). ^{31}P NMR (243 MHz, $CDCl_3$) δ 37.27. ^{13}C NMR (150 MHz, $CDCl_3$) δ 145.6 (d, *J* = 9.9 Hz), 133.3 (d, *J* = 96.0 Hz), 132.5 (d, *J* = 10.4 Hz), 132.2 (d, *J* = 12.1 Hz), 131.3 (d, *J* = 90.0 Hz), 127.2, 62.1, 31.2, 31.2, 28.7 (d, *J* = 14.8 Hz), 22.7, 15.1 (d, *J* = 74.5 Hz), 14.1.

The enantiomeric excess was determined by Daicel Chiralcel IC (0.46 cm x 25 cm), hexanes/IPA = 60/40, 1.0 mL/min, λ = 210 nm, t_r (major) = 20.4 min, t_r (minor) = 40.5 min. HRMS (ESI) *m/z*: $[M+H]^+$ calcd for $C_{15}H_{23}BrPO_2$, 345.0619; found 345.0582.

Suzuki-Miyaura coupling-procedure for chiral (S)-2j with phenylboronic acid: Inside an argon glovebox, a vial that had been dried in an oven, fitted with a magnetic stir bar, was loaded with Pd_2dba_3 (0.005 mmol) and (R)-PPh_3 (0.012 mmol) in 1,4-dioxane (1.0 mL); the resulting solution was stirred at ambient temperature for 10 min. Subsequently, phenylboronic acid (0.30 mmol) and K_3PO_4 (0.60 mmol) were introduced and stirring was continued for an additional 10 min. Chiral substrate **(*S*)-2j** (0.10 mmol) was then added. The Schlenk vial was sealed with a rubber plug, removed from the glovebox, and immersed in an oil bath pre-equilibrated to 110 °C while being stirred overnight. After cooling, the reaction mixture was diluted with EtOAc (10 mL) and H_2O (3 mL). The aqueous phase was extracted with EtOAc (2 × 10 mL), and the combined

organic layers were dried over anhydrous Na_2SO_4, filtered, and concentrated in vacuo. Purification by silica-gel column chromatography afforded the desired arylated product **(*S*)-4**. (NMR data can refer to compound **2e**)

Buchwald-Hartwig amination *procedure for chiral 2j with aniline:* Within an argon-purged glovebox, a magnetic-stirrer-equipped vial that had been thoroughly dried was loaded with Pd_2dba_3 (0.004 mmol) and XPhos (0.008 mmol) in toluene (1.0 mL) and agitated at ambient temperature for 10 min. Aniline (0.15 mmol) and the chiral substrate **(*S*)-2j** (0.10 mmol) were added sequentially. The Schlenk tube was sealed with a rubber septum, removed from the glovebox, and placed in an oil bath pre-heated to 100 °C where it was stirred overnight. After cooling, the mixture was diluted with EtOAc (10 mL) and H_2O (3 mL). The aqueous phase was extracted with EtOAc (2 × 10 mL), and the combined organic extracts were dried over anhydrous Na_2SO_4, filtered, and concentrated in vacuo. Purification by silica-gel column chromatography afforded the desired arylation product **(*S*)-5** as a colorless oil.

(*S,E*)-3-Pentylidene-2-(4-(phenylamino)phenyl)-1,2-oxaphospholane 2-oxide ((*S*)-5)

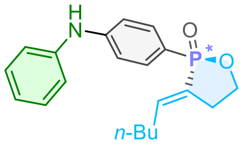

^{1}H NMR (600 MHz, $CDCl_3$) δ 7.66 – 7.51 (m, 2H), 7.36 – 7.28 (m, 2H), 7.21 – 7.11 (m, 2H), 7.09 – 6.99 (m, 3H), 6.36 – 6.17 (m, 2H), 4.59 – 4.30 (m, 2H), 2.99 – 2.73 (m, 2H), 2.22 – 2.07 (m, 2H), 1.43 – 1.24 (m, 4H), 0.87 (t, *J* = 7.3, 3H). ^{31}P NMR (243 MHz, $CDCl_3$) δ 43.51. ^{13}C NMR (150 MHz, $CDCl_3$) δ 148.0 (d, *J* = 2.8 Hz), 142.9 (d, *J* = 12.2 Hz), 141.0, 133.5 (d, *J* = 12.3 Hz), 131.5 (d, *J* = 120.9 Hz), 129.6, 123.1, 120.5, 119.9 (d, *J* = 147.2 Hz), 115.1 (d, *J* = 14.5 Hz), 67.6 (d, *J* = 2.7 Hz), 31.1 (d, *J* = 15.9 Hz), 30.5, 28.8 (d, *J* = 20.7 Hz), 22.5, 14.0.

The enantiomeric excess was determined by Daicel Chiralcel IC (0.46 cm x 25 cm), hexanes/IPA = 40/60, 1.0 mL/min, λ = 254 nm, t_r (minor) = 24.2 min, t_r (major) = 29.1 min. HRMS (ESI) *m/z*: $[M+H]^+$ calcd for $C_{20}H_{25}NPO_2$, 342.1623; found 342.1584.

Buchwald-Hartwig amination procedure for chiral 2j with 9H-carbazole: Under an argon atmosphere inside a glovebox, a stir bar-equipped vial that had been oven-dried was charged with Pd_2dba_3 (0.004 mmol) and XPhos (0.008 mmol) in toluene (1.0 mL) and stirred at ambient temperature for 10 min. 9H-Carbazole (0.15 mmol) and chiral substrate **(*S*)-2j** (0.10 mmol) were then introduced sequentially. After sealing the Schlenk vial with a rubber septum, it was removed from the glovebox and submerged in an oil bath preheated to 100 °C, where the mixture was stirred overnight. The reaction was quenched by dilution with EtOAc (10 mL) and H_2O (3 mL). The aqueous layer was extracted with EtOAc (2 × 10 mL), and the combined organic phases were dried over anhydrous Na_2SO_4, filtered, and concentrated under reduced pressure. Purification by silica-gel column chromatography provided the desired arylation product **(*S*)-6** as a colorless oil.

(*S,E*)-2-(4-(9H-Carbazol-9-yl)phenyl)-3-pentylidene-1,2-oxaphospholane 2-oxide ((*S*)-6)

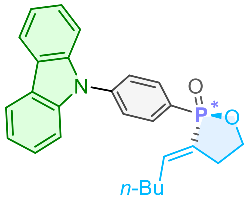

^{1}H NMR (600 MHz, $CDCl_3$) δ 8.14 (d, *J* = 7.8 Hz, 2H), 8.05 – 7.97 (m, 2H), 7.74 – 7.67 (m, 2H), 7.49 – 7.45 (m, 2H), 7.45 – 7.39 (m, 2H), 7.34 – 7.27 (m, 2H), 6.52 – 6.40 (m, 1H), 4.65 – 4.46 (m, 2H), 3.11 – 2.82 (m, 2H), 2.33 – 2.15 (m, 2H), 1.52 – 1.28 (m, 4H), 0.91 (t, *J* = 7.3 Hz, 3H). ^{31}P NMR (243 MHz, $CDCl_3$) δ 41.72. ^{13}C NMR (150 MHz, $CDCl_3$) δ 144.4 (d, *J* = 12.0 Hz), 141.7 (d, *J* = 3.3 Hz), 140.3, 133.5 (d, *J* = 11.7 Hz), 130.7 (d, *J* = 121.0 Hz), 129.9 (d, *J* = 139.0 Hz), 126.7 (d, *J* = 13.9 Hz), 126.3, 123.9, 120.7, 120.5, 109.8, 68.1 (d, *J* = 3.0 Hz), 31.2 (d, *J* = 15.9 Hz), 30.4, 28.9 (d, *J* = 21.2 Hz), 22.5, 14.0.

The enantiomeric excess was determined by Daicel CHIRALPAK IC (0.46 cm x 25 cm), hexanes/IPA = 40/60, 1.0 mL/min, λ = 254 nm, t_r (minor) = 28.7min, t_r (major) = 46.4 min. HRMS (ESI) *m/z*: $[M+H]^+$ calcd for $C_{26}H_{27}NPO_2$, 416.1779; found 416.1729.

1,4-Conjugate hydrophosphination reaction procedure for chiral* (*S*)-2o *with diphenylphosphine: Inside an argon-purged glovebox, a vial that had been dried in an oven and equipped with a magnetic stir bar was charged with $Cu(MeCN)_4PF_6$ (0.005 mmol) and dppp (0.008 mmol) in THF (1.0 mL) and stirred at ambient temperature for 10 min. Diphenylphosphine (0.15 mmol) and **(*S*)-2o** (0.10 mmol) were then introduced. The Schlenk vial was sealed with a rubber septum and stirred at room temperature overnight. After reaction completion, S_8 was added to quench the mixture, which was subsequently diluted with EtOAc (10 mL) and H_2O (3 mL). The aqueous layer was extracted with EtOAc (2 × 10 mL), and the combined organic phases were dried over anhydrous Na_2SO_4, filtered, and concentrated in vacuo. Purification by silica-gel column chromatography delivered the corresponding arylation products **(*R*)-7a** and **(*R*)-7b** as colorless solids.

(*R,E*)-2-((*R*)-2-(Diphenylphosphorothioyl)-1-phenylethyl)-3-pentylidene-1,2-oxaphospholane 2-oxide ((*R*)-7a)

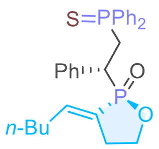

^{1}H NMR (600 MHz, $CDCl_3$) δ 7.89 – 7.75 (m, 2H), 7.51 – 7.37 (m, 5H), 7.22 – 6.88 (m, 8H), 5.46 – 5.31 (m, 1H), 4.39 – 4.29 (m, 1H), 4.19 – 4.10 (m, 1H), 3.90 – 3.76 (m, 1H), 3.48 – 3.36 (m, 1H), 3.19 – 3.05 (m, 1H), 2.79 – 2.66 (m, 1H), 2.55 – 2.43 (m, 1H), 1.98 – 1.84 (m, 2H), 1.15 – 1.04 (m, 4H), 0.82 – 0.75 (m, 3H). ^{31}P NMR (243 MHz, $CDCl_3$) δ52.77 (d, *J* = 55.6 Hz), 42.71 (d, *J* = 55.6 Hz).. ^{13}C NMR (150 MHz, $CDCl_3$) δ 145.48 (d, *J* = 10.2 Hz), 134.26 (d, *J* = 82.8 Hz), 133.57, 131.26 (d, *J* = 114.1 Hz), 131.13 (dd, *J* = 86.3, 10.4 Hz), 130.33 (d, *J* = 6.4 Hz), 128.81 (d, *J* = 12.0 Hz), 128.07 (d, *J* = 2.3 Hz), 127.93 (d, *J* = 12.4 Hz), 127.33 (d, *J* = 3.0 Hz), 66.92, 41.70 (d, *J* = 89.1 Hz), 31.75 (d, *J* = 54.1 Hz), 30.84 (d, *J* = 14.9 Hz), 30.13, 28.33 (d, *J* = 21.2 Hz), 22.19, 13.82.

The enantiomeric excess was determined by CHIRALPAK® OD-H (0.46 cm x 25 cm), hexanes/IPA = 80/20, 1.0 mL/min, λ = 210 nm, t_r (major) = 8.0 min, t_r (minor) = 9.2 min. HRMS (ESI) *m/z*: $[M+H]^+$ calcd for $C_{28}H_{33}P_2SO_2$, 495.1676; found 495.1621.

(*R,E*)-2-((*S*)-2-(Diphenylphosphorothioyl)-1-phenylethyl)-3-pentylidene-1,2-oxaphospholane 2-oxide ((*R*)-7b)

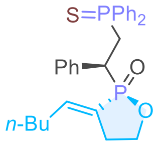

^{1}H NMR (600 MHz, $CDCl_3$) δ 7.91 – 7.78 (m, 2H), 7.50 – 7.39 (m, 5H), 7.24 – 7.17 (m, 1H), 7.12 – 7.05 (m, 2H), 7.03 – 6.86 (m, 5H), 6.44 (dt, *J* = 17.2, 7.8 Hz, 1H), 4.21 – 4.05 (m, 2H), 3.82 – 3.72 (m, 1H), 3.40 – 3.24 (m, 2H), 2.55 – 2.44 (m, 1H), 2.11 – 1.98 (m, 2H), 1.67 – 1.59 (m, 1H), 1.37 – 1.25 (m, 4H), 0.87 (t, *J* = 7.3 Hz, 3H). ^{31}P NMR (243 MHz, $CDCl_3$) δ 54.75 (d, *J* = 57.5 Hz), 42.59 (d, *J* = 57.3 Hz). ^{13}C NMR (150 MHz, $CDCl_3$) δ 144.17 (d, *J* = 11.4 Hz), 134.26 (d, *J* = 82.9 Hz), 133.44 (d, *J* = 5.7 Hz), 131.63 (d, *J* = 3.1 Hz), 131.42 (d, *J* = 10.6 Hz), 130.98 (d, *J* = 9.9 Hz), 130.84 (d, *J* = 3.0 Hz), 130.39 (d, *J* = 5.9 Hz), 128.84 (d, *J* = 12.1 Hz), 127.97 (d, *J* = 7.6 Hz), 127.92 (d, *J* = 2.2 Hz), 127.09 (d, *J* = 3.4 Hz), 68.14, 41.95 (dd, *J* = 88.3, 2.3 Hz), 30.92 (d, *J* = 15.7 Hz), 30.70 (d, *J* = 53.2 Hz), 30.34, 28.26 (d, *J* = 20.0 Hz), 22.44, 13.91. The enantiomeric excess was determined by CHIRALPAK® OD-H (0.46 cm x 25 cm), hexanes/IPA = 80/20, 1.0 mL/min, λ = 210 nm, t_r (major) = 14.5 min, t_r (minor) = 17.5 min. HRMS (ESI) *m/z*: $[M+H]^+$ calcd for $C_{28}H_{33}P_2SO_2$, 495.1676; found 495.1621.

7. Mechanistic studies

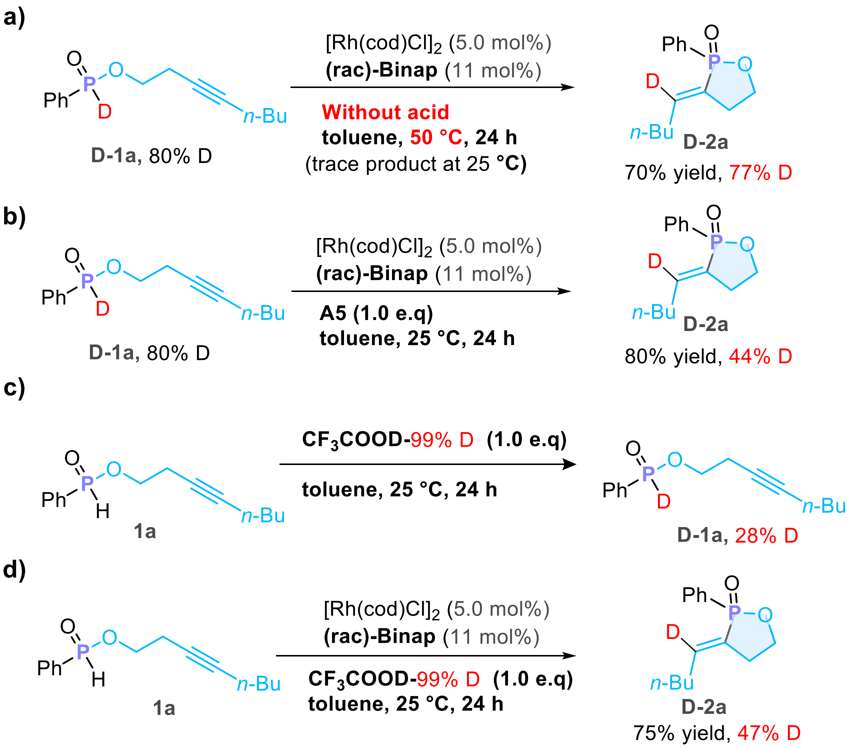

Figure S8. Deuterium-labelling experiment

To investigate the possible reaction mechanism, a deuterium-labelling experiment has been conducted by using **D-1a** (80% D) as starting material, which afforded **2a** with 74% D. The second experiment involving an acid additive, we observed a lower level of deuteration in the product D-2a (44% D).

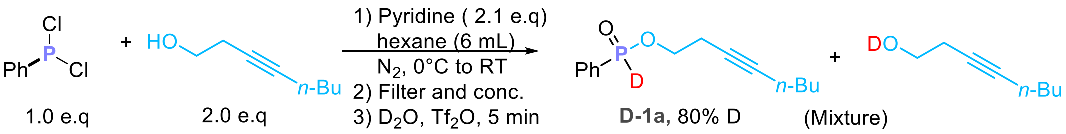

Figure S9. Procedures for the synthesis of **D-1a**.

Procedure for oct-3-yn-1-yl deuteriumphenylphosphinate* (D-1a)*: To 0°C, magnetically stirred solution of dichlorophenylphosphine (3.5 mmol) in dry hexane (6 ml) was added Pyridine (7.3 mmol). The oct-3-yn-1-ol **S1** was added dropwisely and then allowed to warm to rt slowly over a period of 20 min. The reaction mixture was filtered in glove box (Note: Be careful with

moisture and use dry glassware) and the filtrate was concentrated with Rotary evaporator. Then adding 70 ul D_2O with 1 drop of Tf_2O, and shake for 5 min. Finally, use an oil pump to remove water and acid. (We also tried to remove the D-alcohols through vacuum distillation, but in the end we could only obtain **D-1a** with 40% D). ^{1}H NMR (600 MHz, $CDCl_3$) δ 7.84 – 7.70 (m, 2H), 7.66 – 7.58 (m, 1H), 7.57 – 7.43 (m, 2H), 4.21 – 4.02 (m, 2H), 2.69 – 2.54 (m, 2H), 2.18 – 1.99 (m, 2H), 1.50 – 1.30 (m, 4H), 1.02 – 0.80 (m, 3H). ^{31}P NMR (243 MHz, $CDCl_3$) δ 25.20 (t, J = 87.0 Hz).

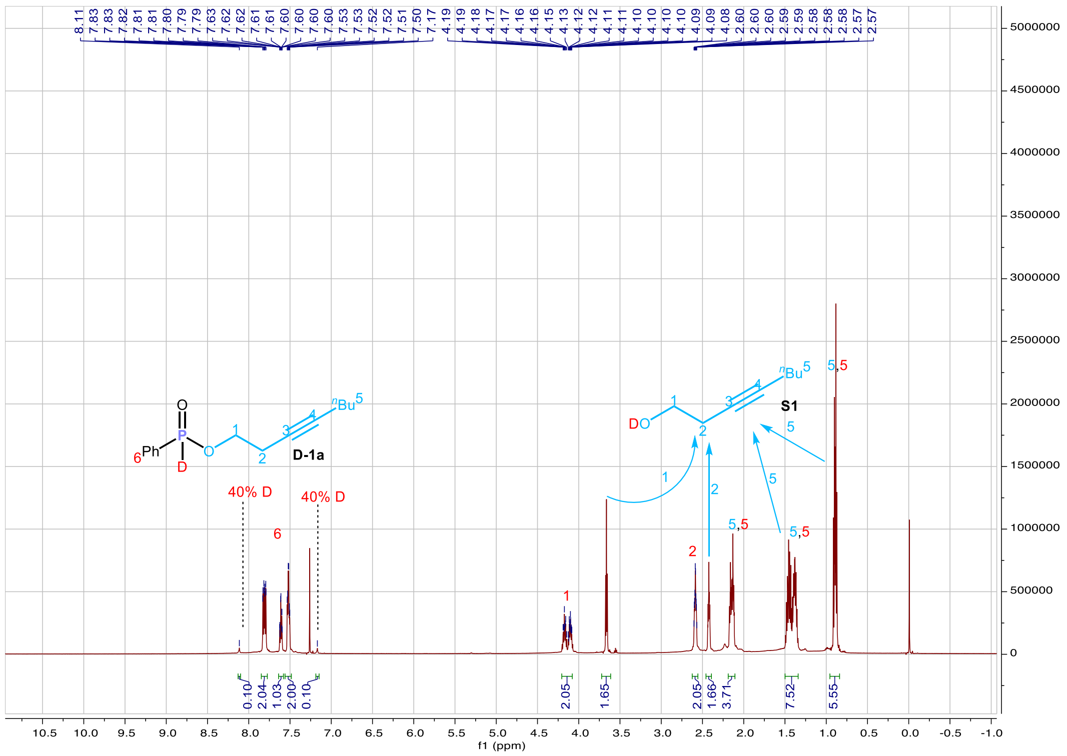

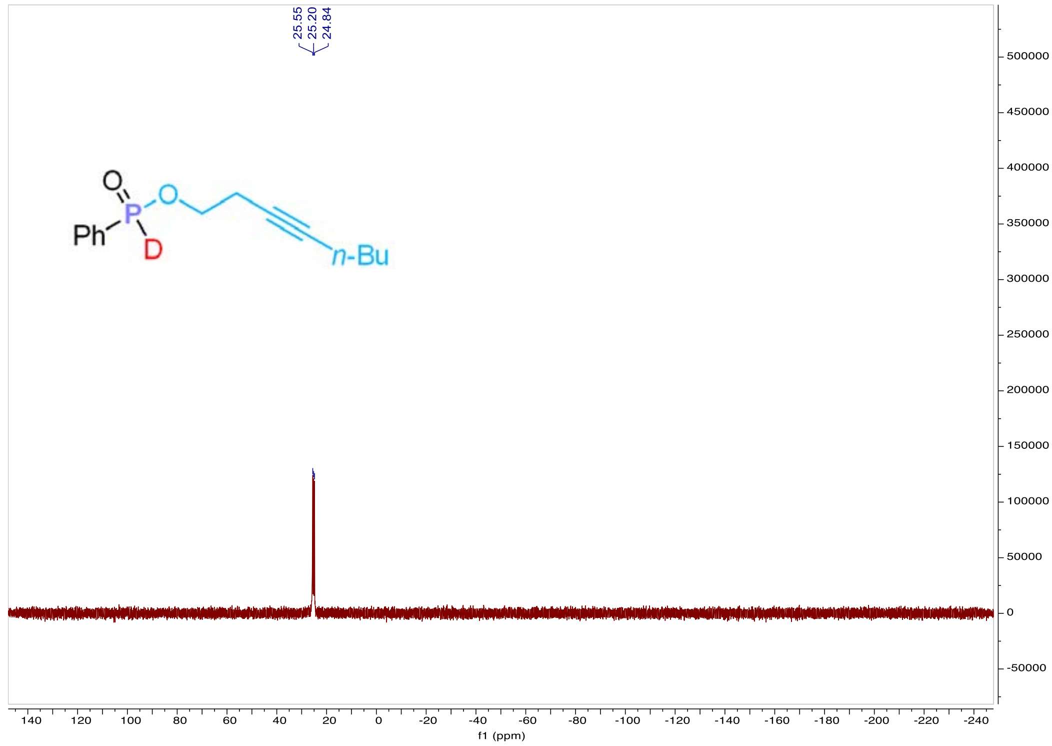

Figure S10. ^{1}H NMR and ^{31}P NMR (400 MHz, $CDCl_3$, 25 °C) spectra for **S1** and **D-1a**

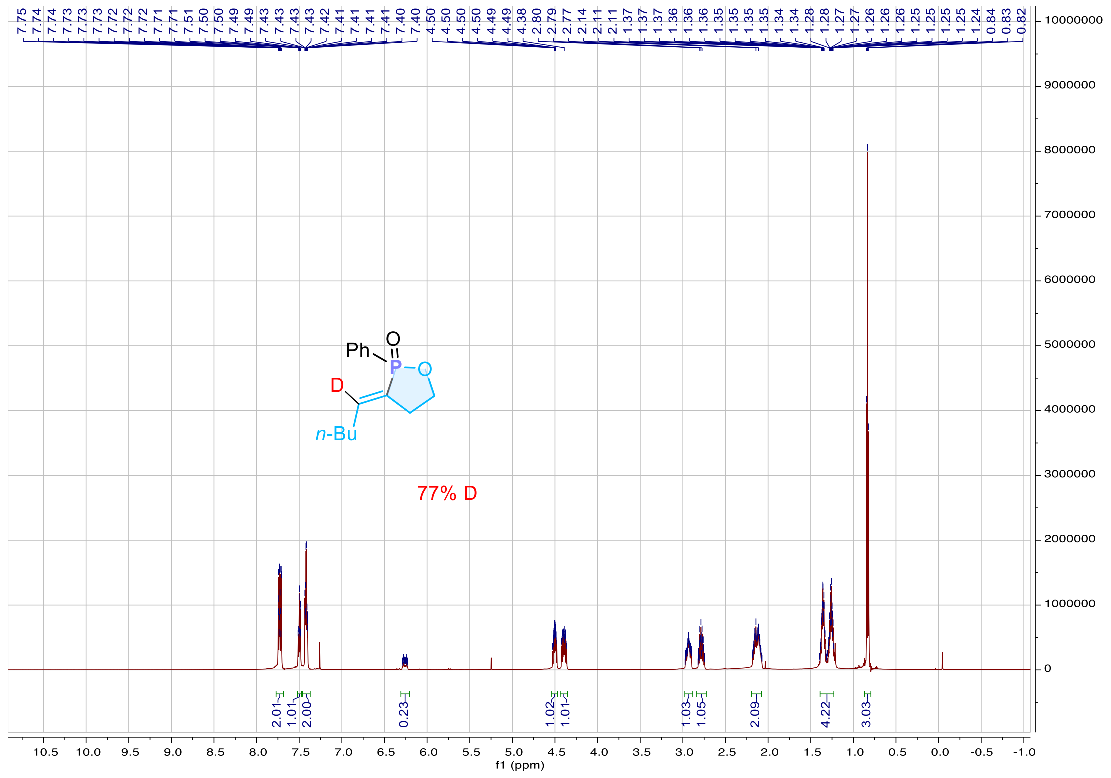

Figure S11. ^{1}H NMR (400 MHz, $CDCl_3$, 25 °C) spectra for **74% D-2a**.

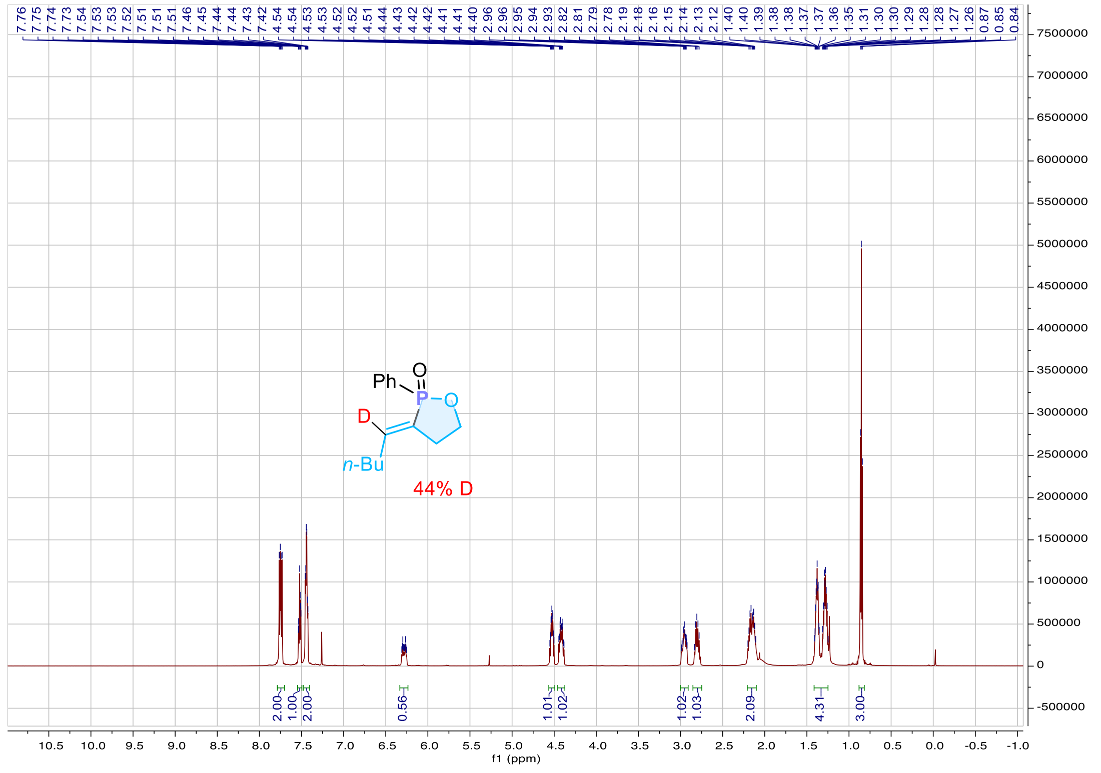

Figure S12. ^{1}H NMR (400 MHz, $CDCl_3$, 25 °C) spectra for **44% D-2a**.

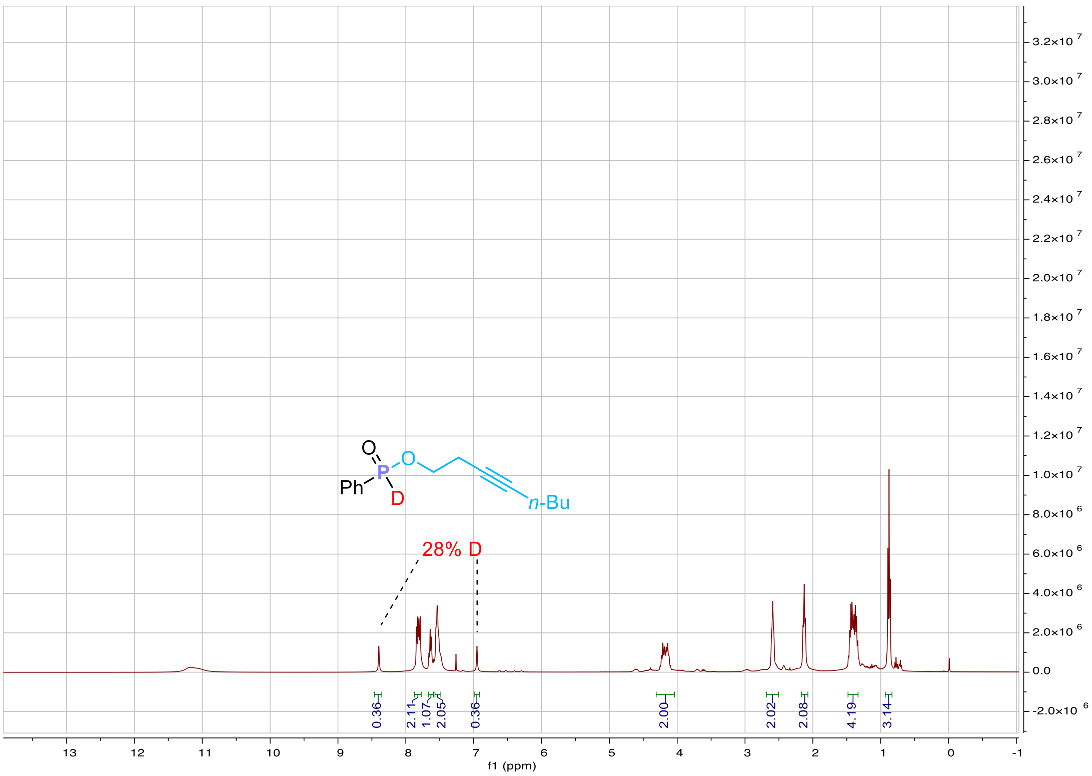

Figure S13. ^{1}H NMR (400 MHz, $CDCl_3$, 25 °C) spectra for **28% D-1a**.

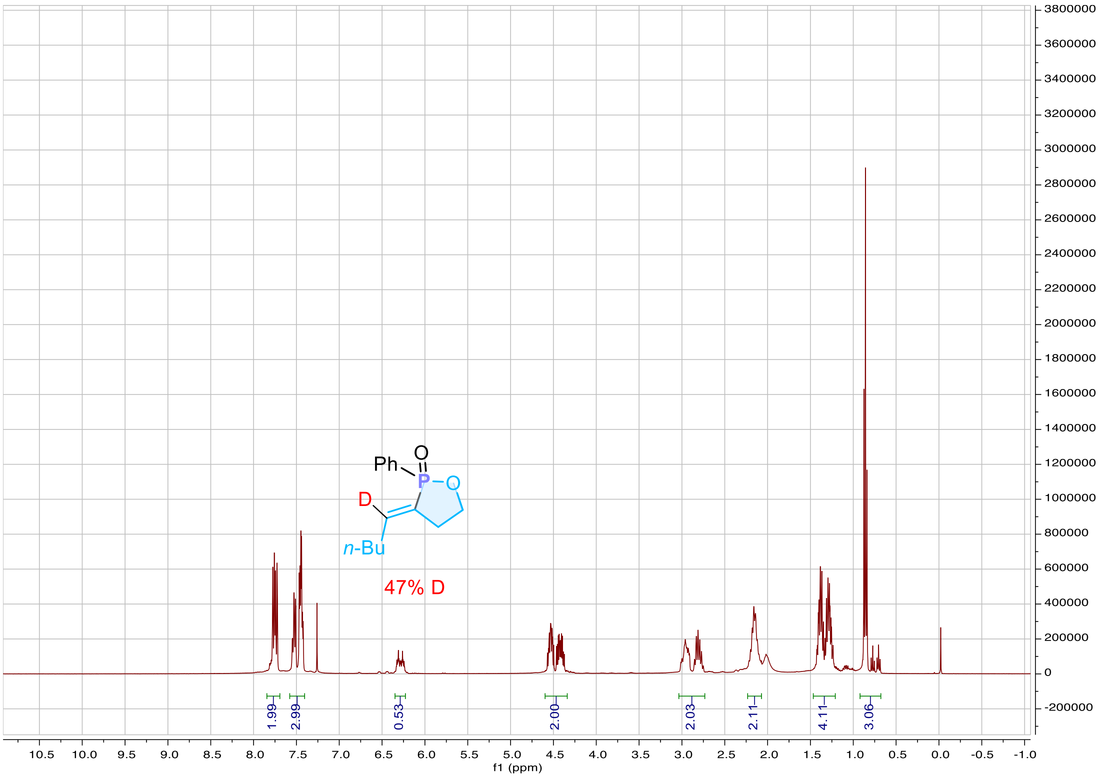

Figure S14. ^{1}H NMR (400 MHz, $CDCl_3$, 25 °C) spectra for **47% D-2a**.

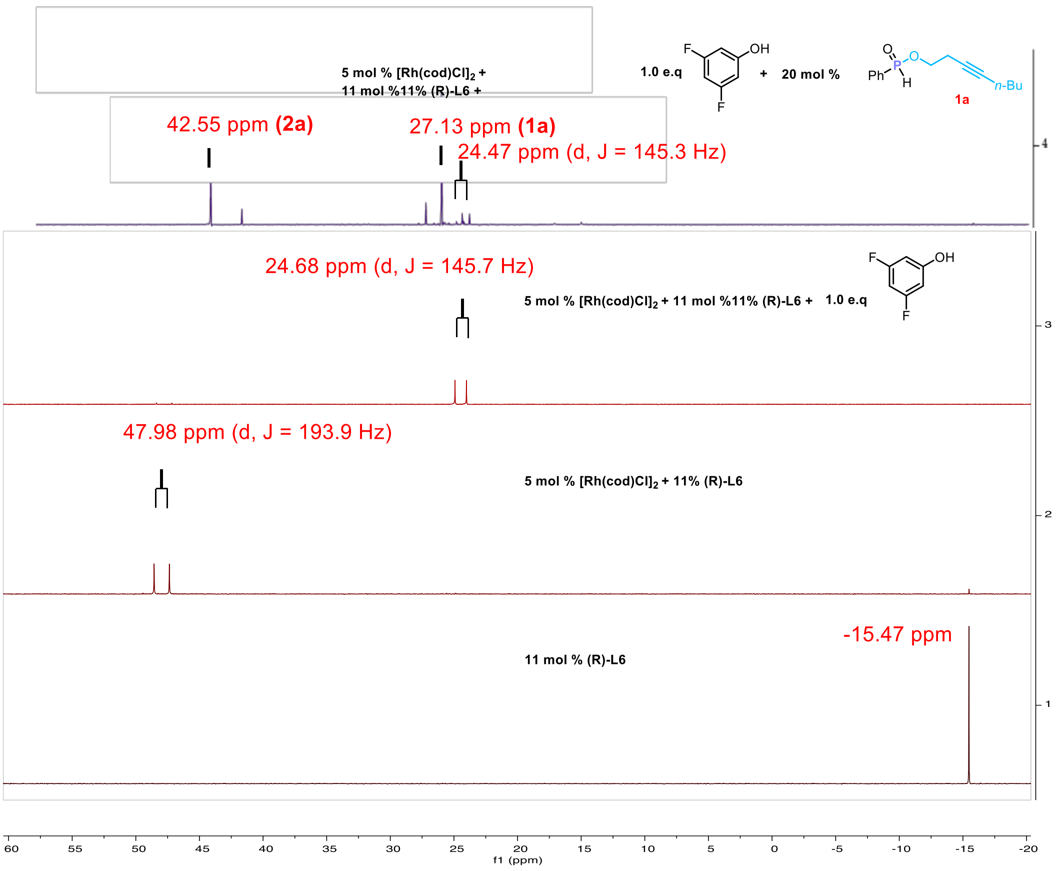

Figure S15. Study on existence of possible intermediates via ^{31}P NMR.

For study on oxidative addition process was employed 7.1 mg (*R*)-**L6** (0.011 mmol) in 0.5 ml CDCl3. ^{31}P NMR (162 MHz, $CDCl_3$) δ -15.47. 2.4 mg $[Rh(cod)Cl]_2$ and and 7.1 mg (*R*)-**L6** (0.011 mmol) was stir at rt under glove box for 10 min, then transferred into the NMR tube under N_2 atmosphere. ^{31}P NMR (162 MHz, $CDCl_3$) δ 48.96 (d, *J* = 193.9 Hz). Then, 3,5-Difluorophenol (0.1 mmol) was added to the mixture of $[Rh(cod)Cl]_2$ and (*R*)-**L6**, stired at RT in glove box for 30 min, then transferred into the NMR tube under N_2 atmosphere. ^{31}P NMR (162 MHz, $CDCl_3$) δ 24.68 (d, *J* = 145.7 Hz). Then, **1a** (0.1 mmol) was added to the mixture of $[Rh(cod)Cl]_2$, (R)-Binap and 3,5-Difluorophenol in glove box, then transferred into the NMR tube under N_2 atmosphere. ^{31}P NMR (202 MHz, $CDCl_3$) δ 42.55 (**2a**), 27.22(**1a**), 24.47 (d, *J* = 145.3 Hz). With same procedure, the mixture of 2.4 mg $[Rh(cod)Cl]_2$, 6.8 mg (R)-Binap (0.011 mmol) and 3,5-Difluorophenol (0.1 mmol) was detected by HRMS (ESI, MeCN) *m/z*: $[M\text{-}Cl]^+$ calcd for $C_{50}H_{36}F_2OP_2Rh^+$, 855.1259; found 855.1309.

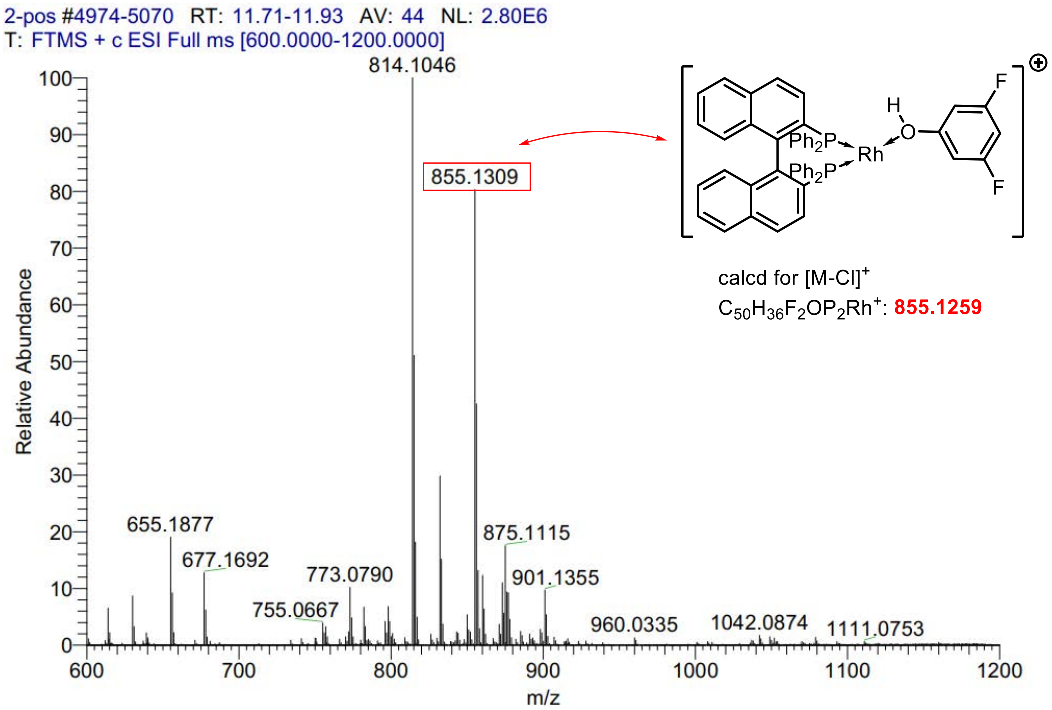

Figure S16. Study on existence of possible intermediates via HRMS

Table S6. Mechanistic study: Time course of kinetic resolution of **1a**.

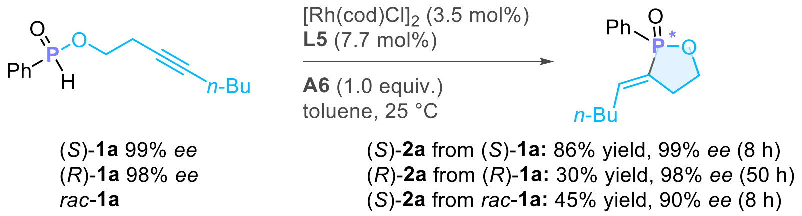

entry	time (min)	NMR yield of **(*S*)-1a**	NMR yield of (*R*)-**1a**
1	5	4%	<1 %
2	15	6 %	<1 %
3	120	23%	1 %
4	240	45%	2 %
5	480	86%	5 %
6	3000	99%, 99% *ee*	32 %, 98% *ee*

8. X-Ray crystallographic analysis

Identification code	cxy5732_0m
Empirical formula	C28H32O2P2S
Formula weight	494.57
Temperature/K	100.0(2)
Crystal system	monoclinic
Space group	$P2_1$
a/Å	11.9565(10)
b/Å	9.0501(8)
c/Å	12.1560(10)
α/°	90
β/°	97.368(3)
γ/°	90
Volume/Å3	1304.51(19)
Z	2
ρcalcg/cm3	1.259
μ/mm-1	1.598
F(000)	524.0
Crystal size/mm3	0.25 × 0.19 × 0.18
Radiation	GaKα (λ = 1.34138)
2Θ range for data collection/°	6.484 to 114.196
Index ranges	-14 ≤ h ≤ 14, -11 ≤ k ≤ 11, -15 ≤ l ≤ 12
Reflections collected	26048
Independent reflections	5319 [R_{int} = 0.0561, R_{sigma} = 0.0435]
Data/restraints/parameters	5319/1/299
Goodness-of-fit on F2	1.033
Final R indexes [I>=2σ (I)]	R_1 = 0.0279, wR_2 = 0.0719
Final R indexes [all data]	R_1 = 0.0315, wR_2 = 0.0725
Largest diff. peak/hole/e Å-3	0.28/-0.20
Flack parameter	-0.038(11)

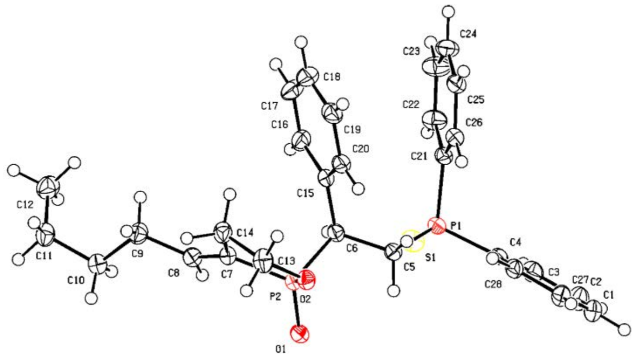

Figure S17. X-Ray crystal structure of **7b (CCDC 2419354)**

9. ^{1}H, ^{13}C, ^{19}F and ^{31}P NMR spectra for compounds

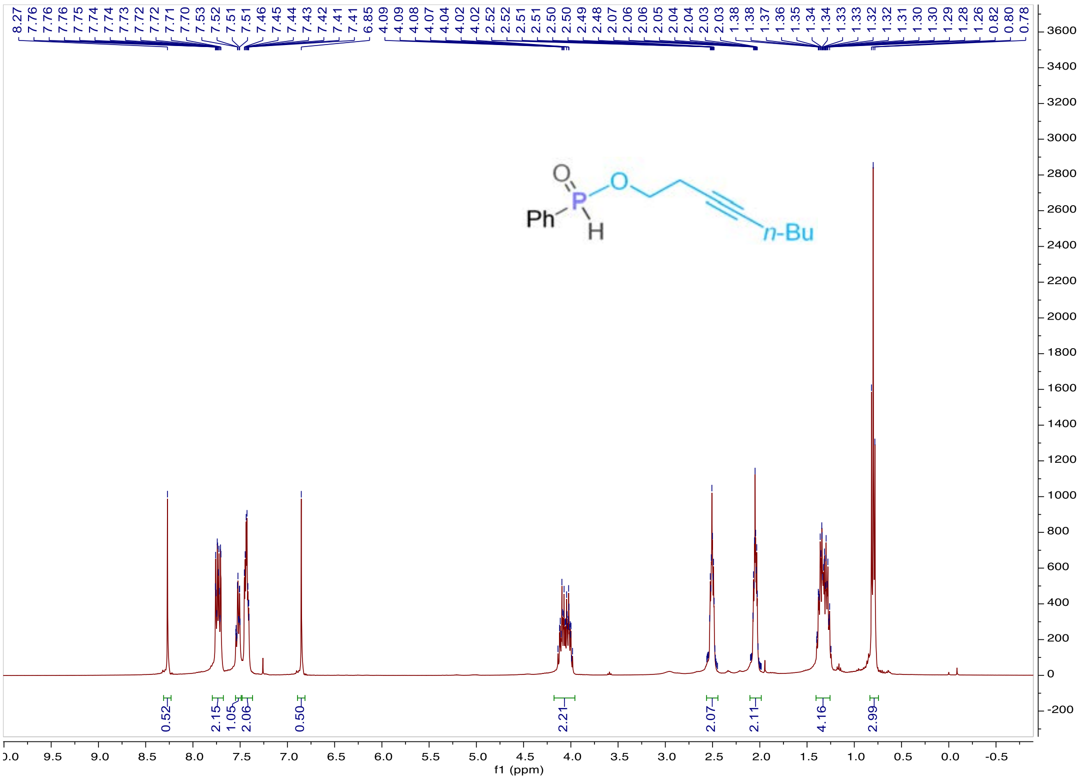

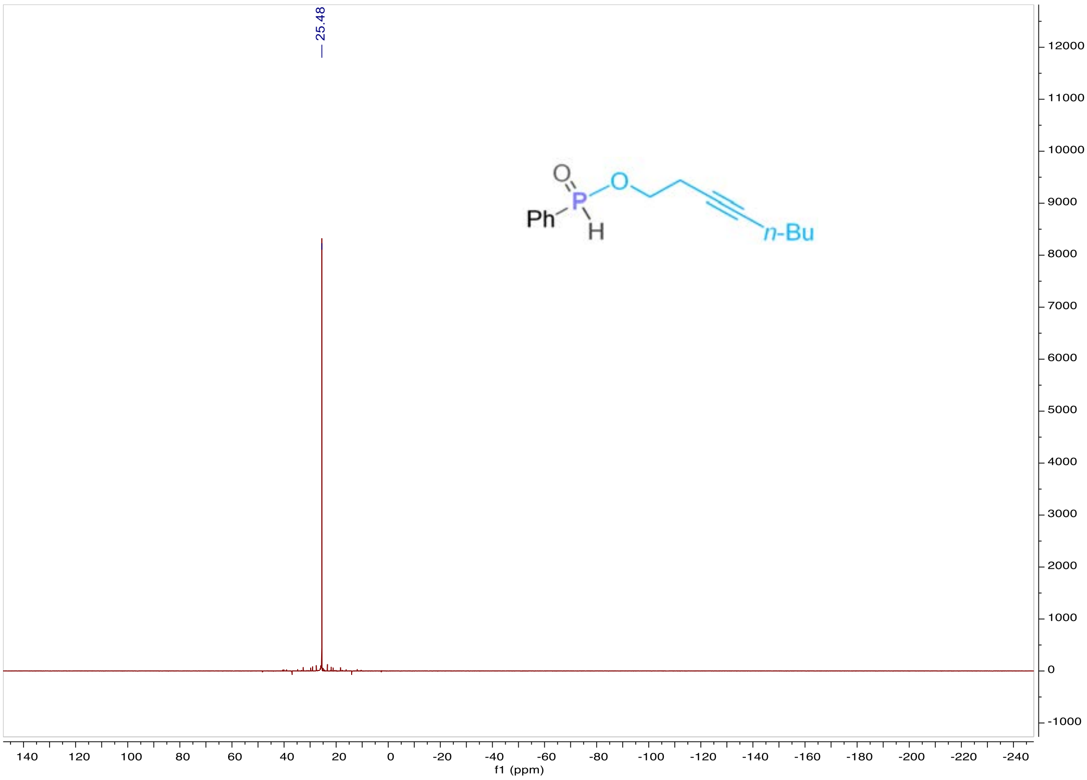

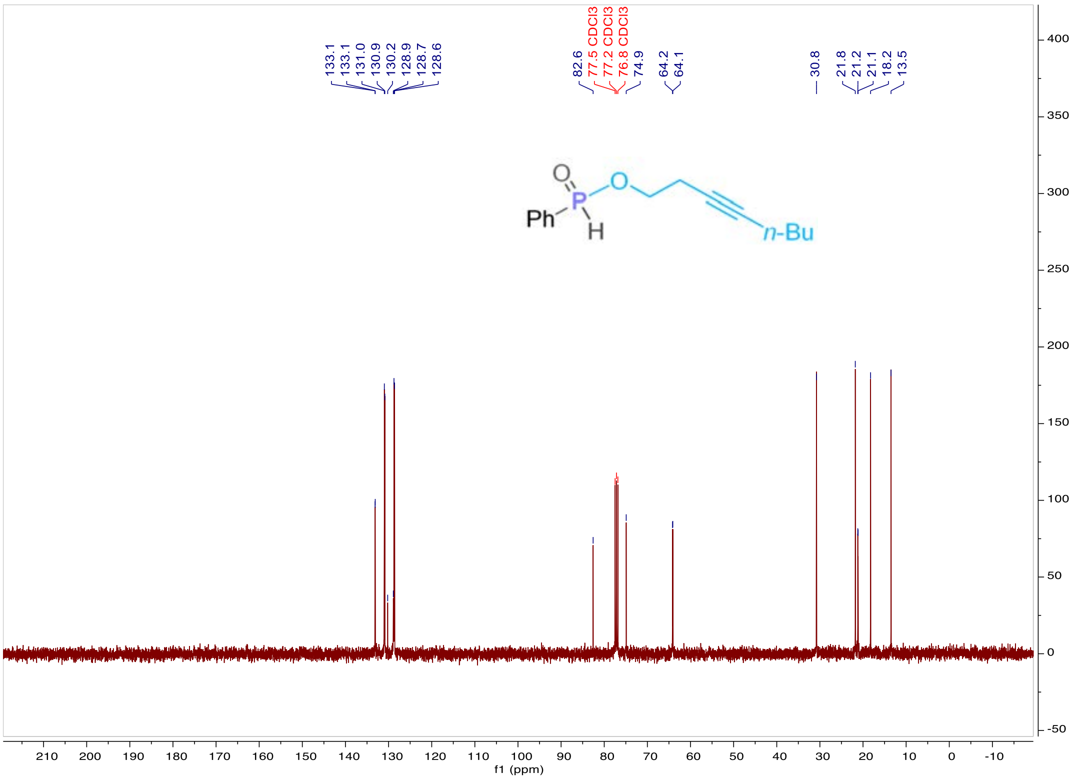

Figure S18. ^{1}H NMR, ^{31}P NMR and ^{13}C NMR spectra for **1a**

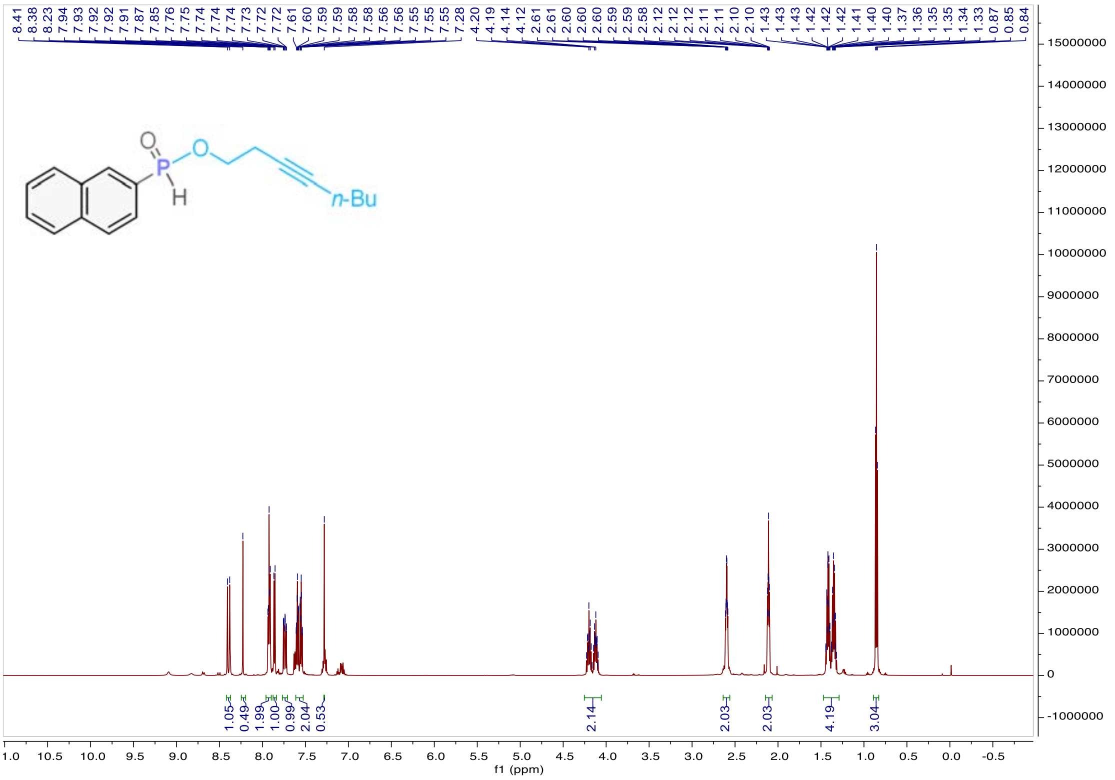

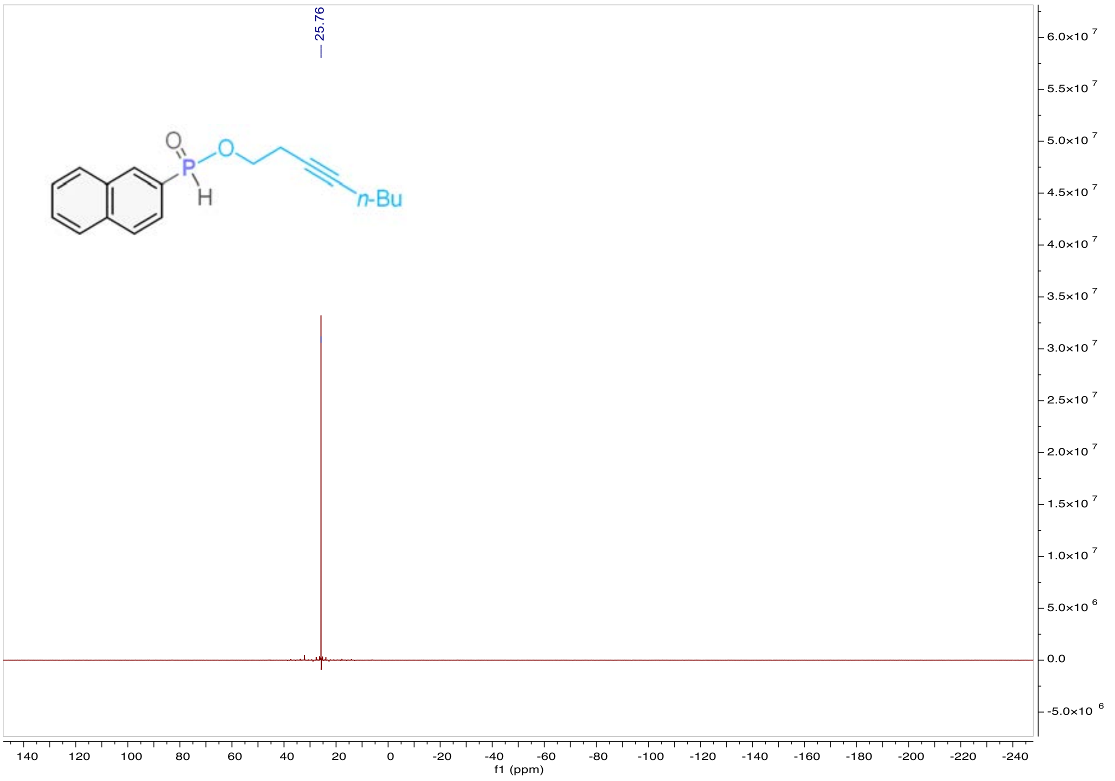

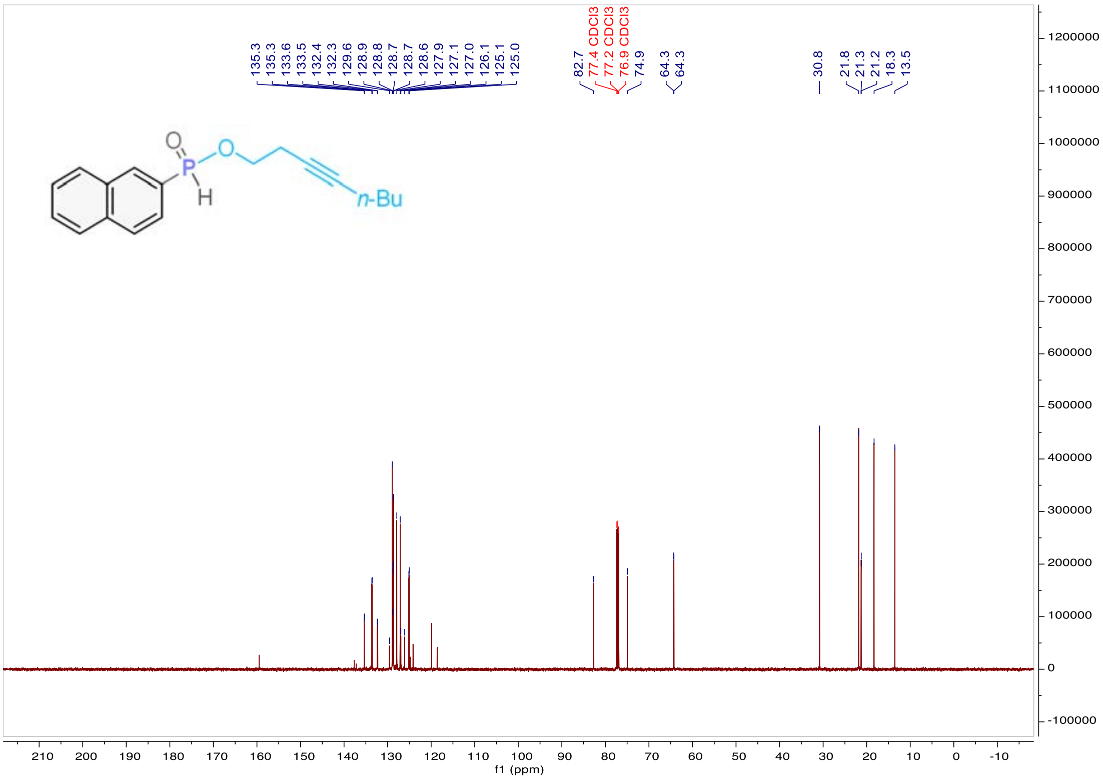

Figure S19. ^{1}H NMR, ^{31}P NMR and ^{13}C NMR spectra for **1b**

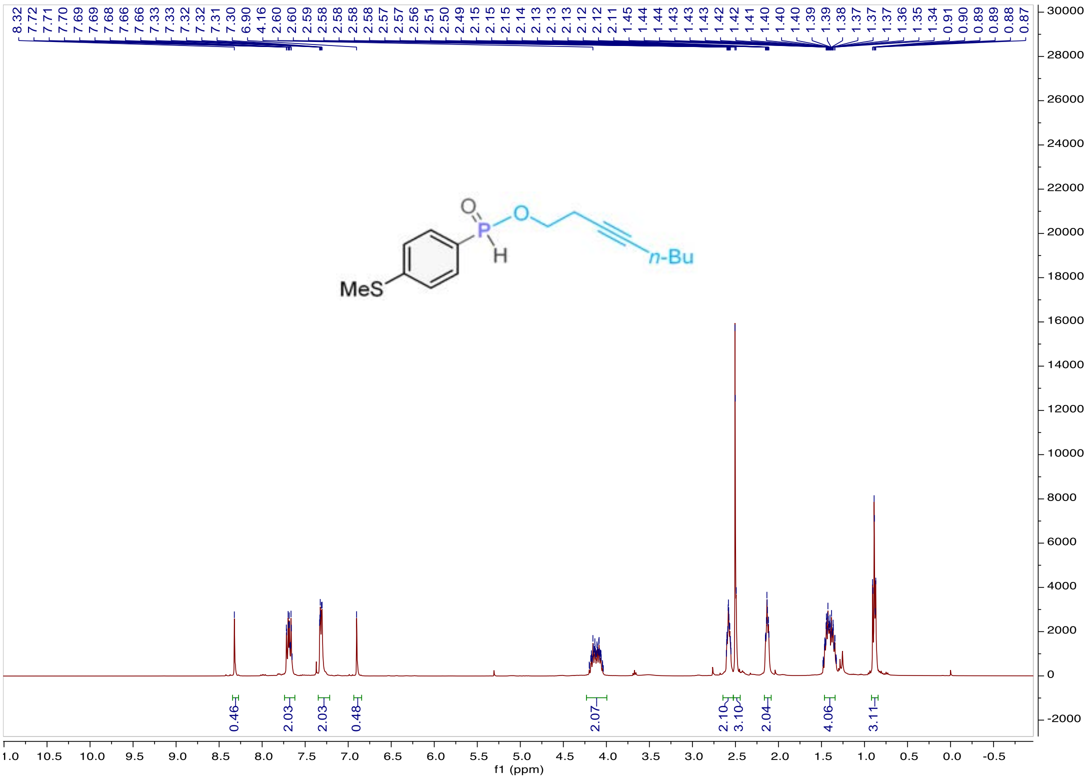
8.32
7.72
7.71
7.70
7.69
7.68
7.66
7.33
7.32
7.32
7.31
7.30
6.90
4.16
2.60
2.60
2.59
2.58
2.58
2.58
2.57
2.57
2.56
2.51
2.50
2.49
2.15
2.15
2.14
2.13
2.13
2.13
2.12
2.12
2.11
1.45
1.44
1.44
1.43
1.43
1.43
1.42
1.42
1.41
1.40
1.40
1.39
1.39
1.38
1.37
1.37
1.36
1.35
1.34
0.91
0.90
0.89
0.89
0.88
0.87
O
P
O
H
MeS
n-Bu
0.46
2.03
2.03
0.48
2.07
2.10
3.10
2.04
4.06
3.11
f1 (ppm)

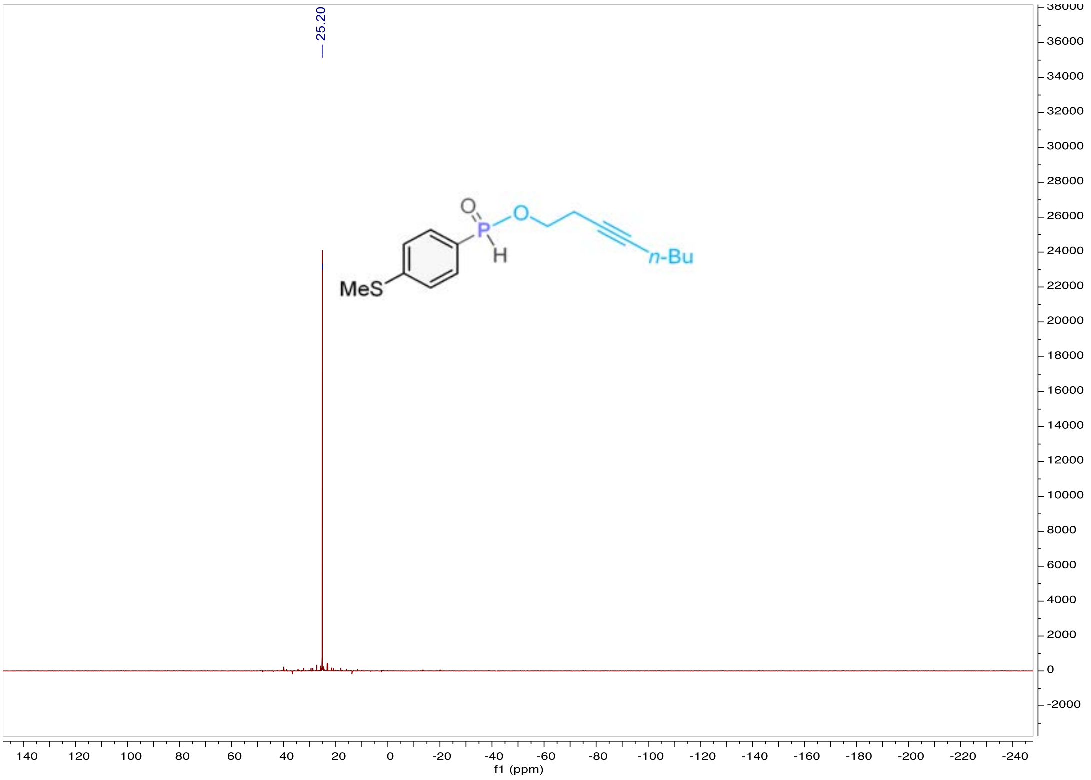
25.20
O
P
O
H
MeS
n-Bu
f1 (ppm)

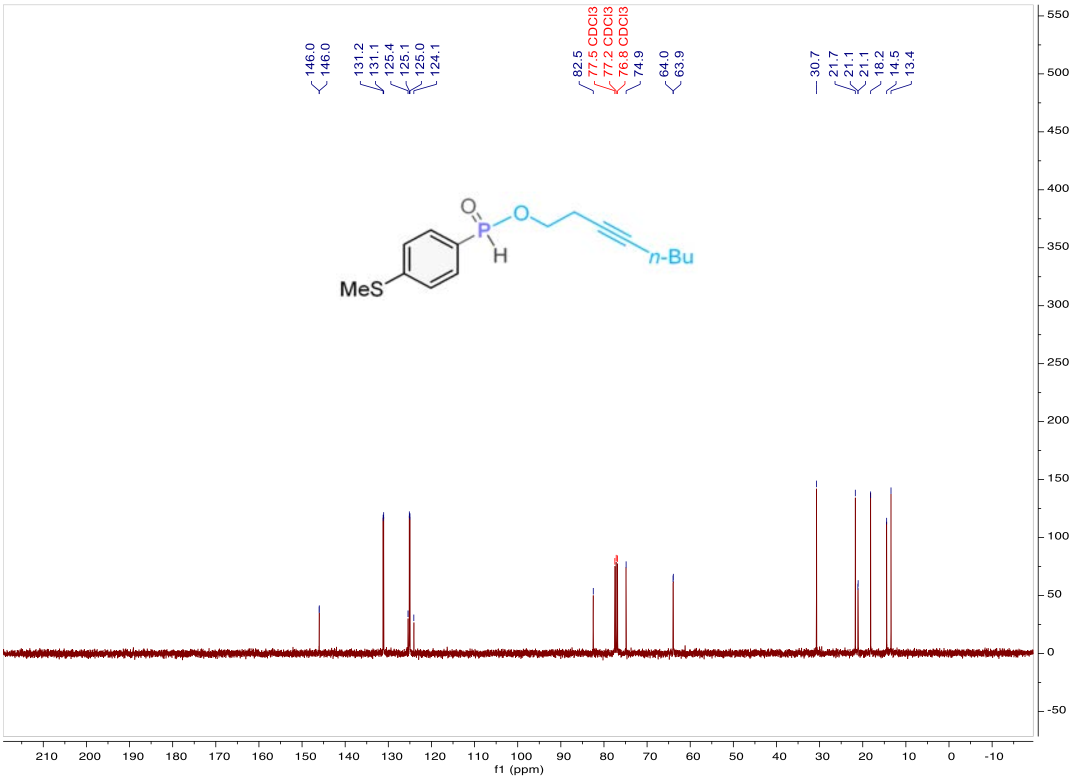

Figure S20. ^{1}H NMR, ^{31}P NMR and ^{13}C NMR spectra for **1c**

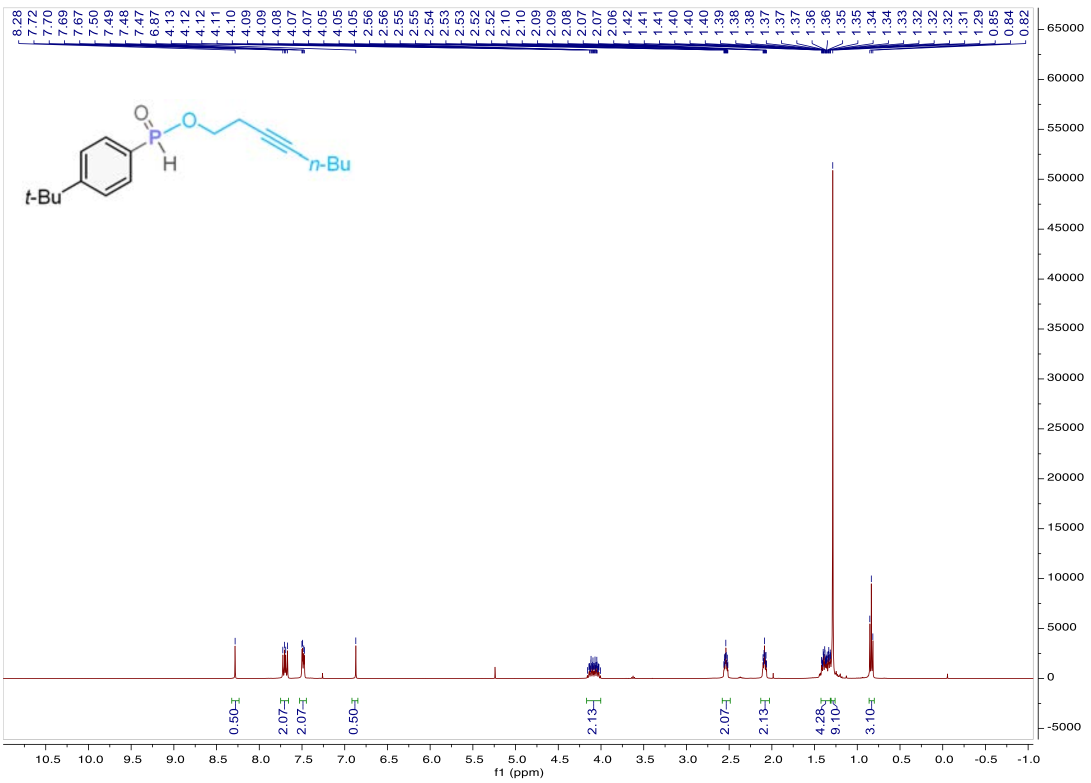

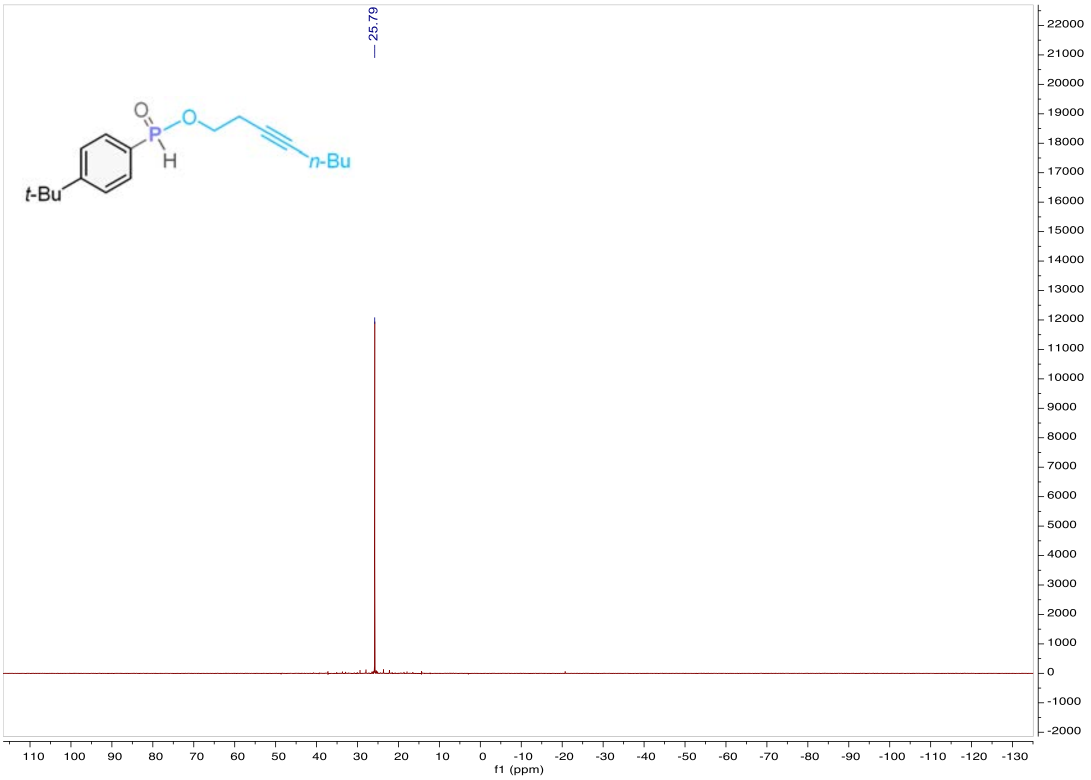

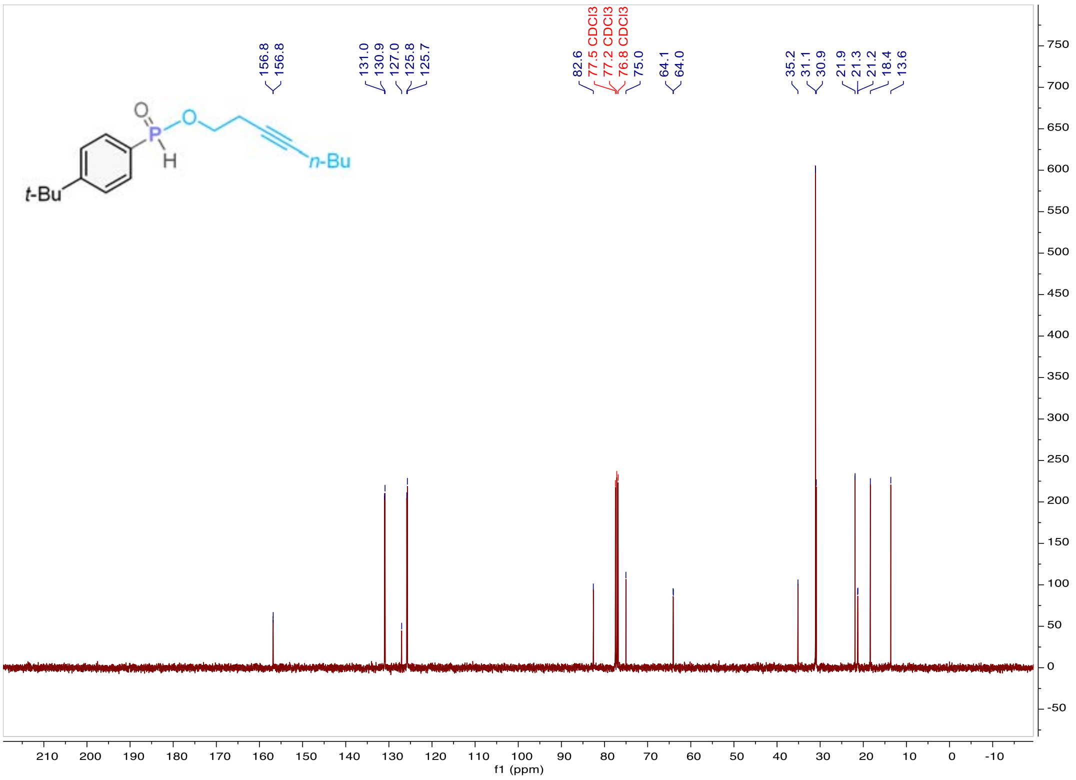

Figure S21. ^{1}H NMR, ^{31}P NMR and ^{13}C NMR spectra for **1d**

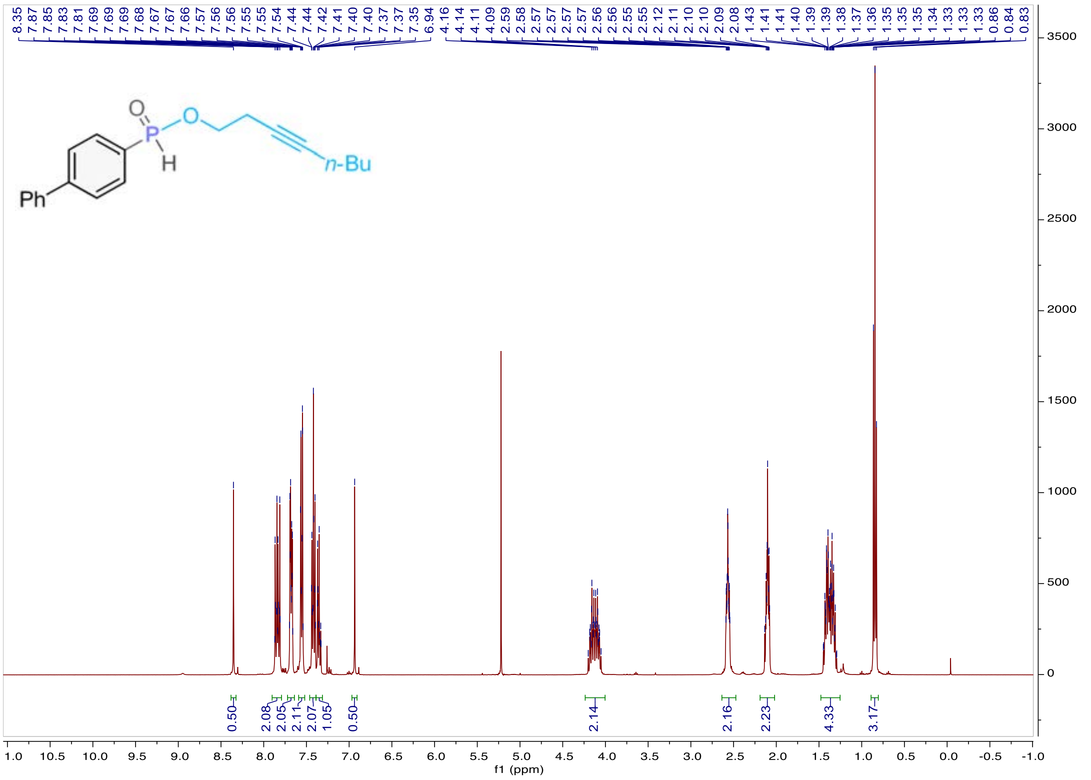

Ph
O
P
O
H
n-Bu
8.35
7.87
7.85
7.83
7.81
7.69
7.69
7.68
7.67
7.67
7.66
7.57
7.56
7.55
7.55
7.54
7.44
7.44
7.42
7.41
7.40
7.40
7.37
7.37
7.35
6.94
4.16
4.14
4.11
4.09
2.59
2.58
2.57
2.57
2.57
2.57
2.56
2.56
2.55
2.55
2.55
2.12
2.11
2.10
2.10
2.09
2.08
1.43
1.41
1.41
1.40
1.39
1.39
1.38
1.37
1.36
1.35
1.35
1.34
1.33
1.33
1.33
0.86
0.84
0.83
0.50
2.08
2.05
2.11
2.07
1.05
0.50
2.14
2.16
2.23
4.33
3.17
f1 (ppm)

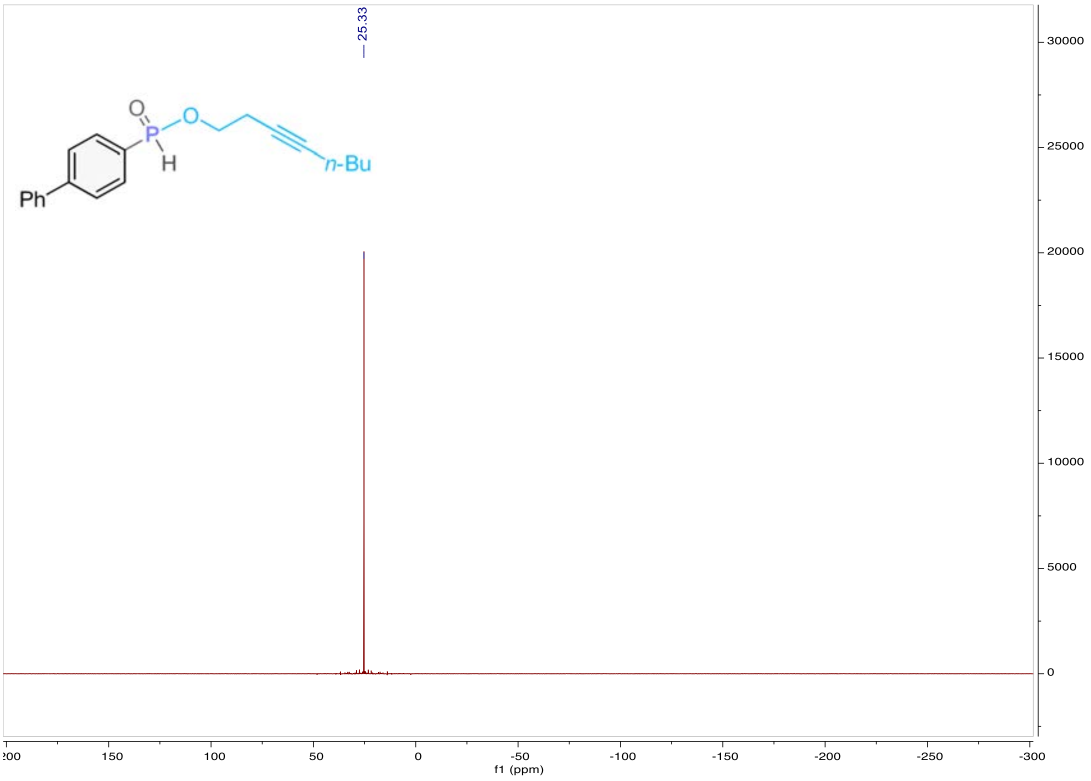

Ph
O
P
O
H
n-Bu
25.33
f1 (ppm)

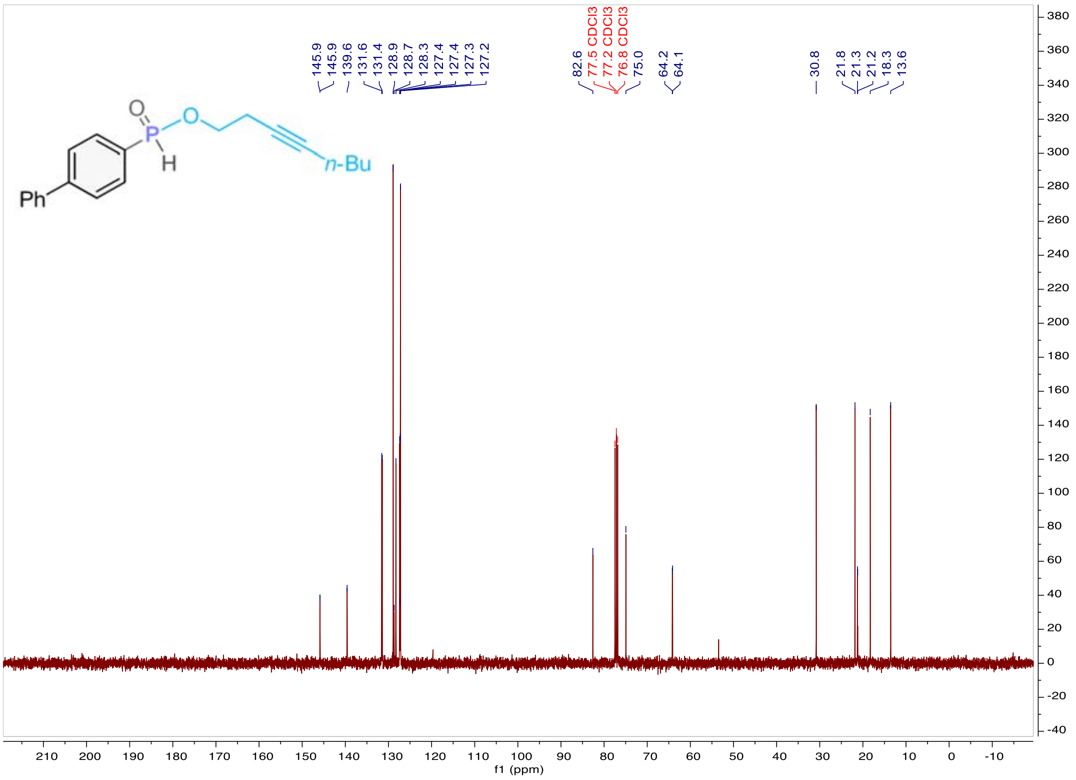

Figure S22. ^{1}H NMR, ^{31}P NMR and ^{13}C NMR spectra for **1e**

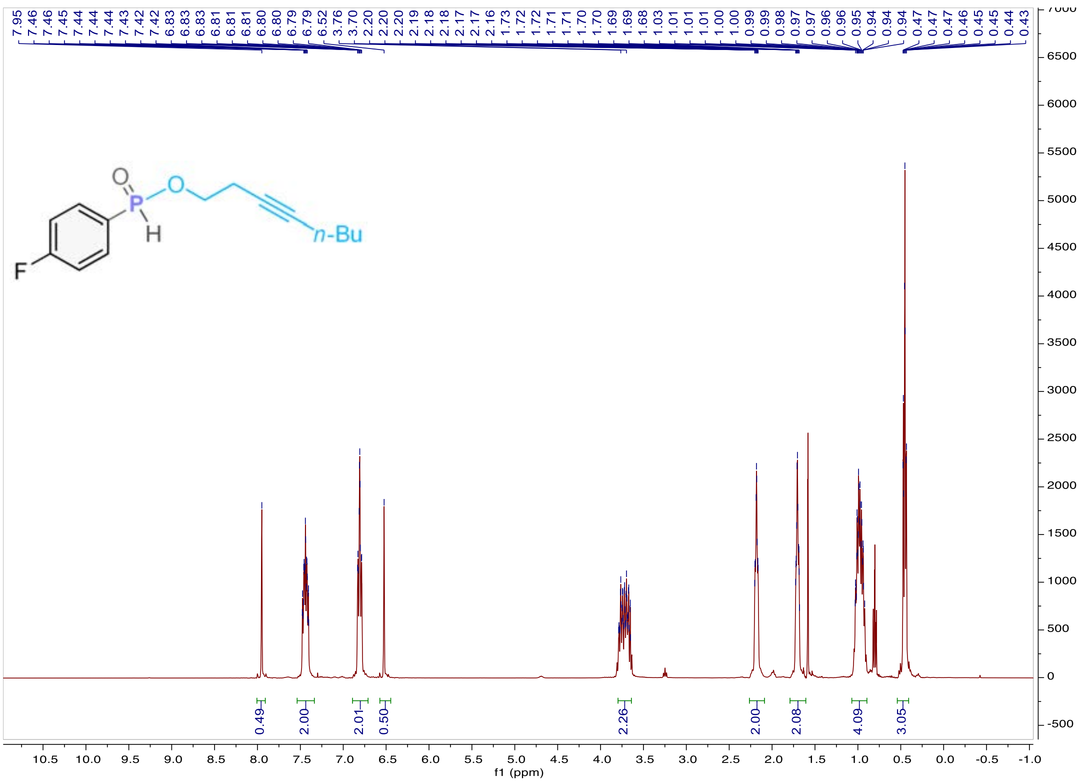

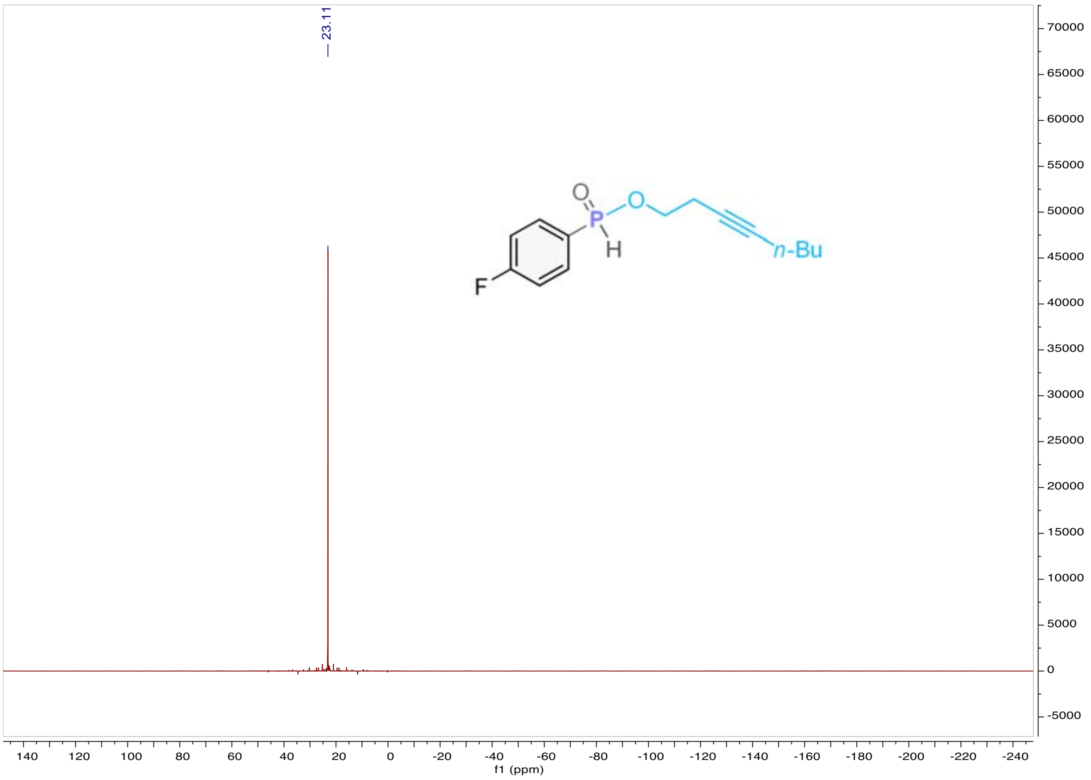

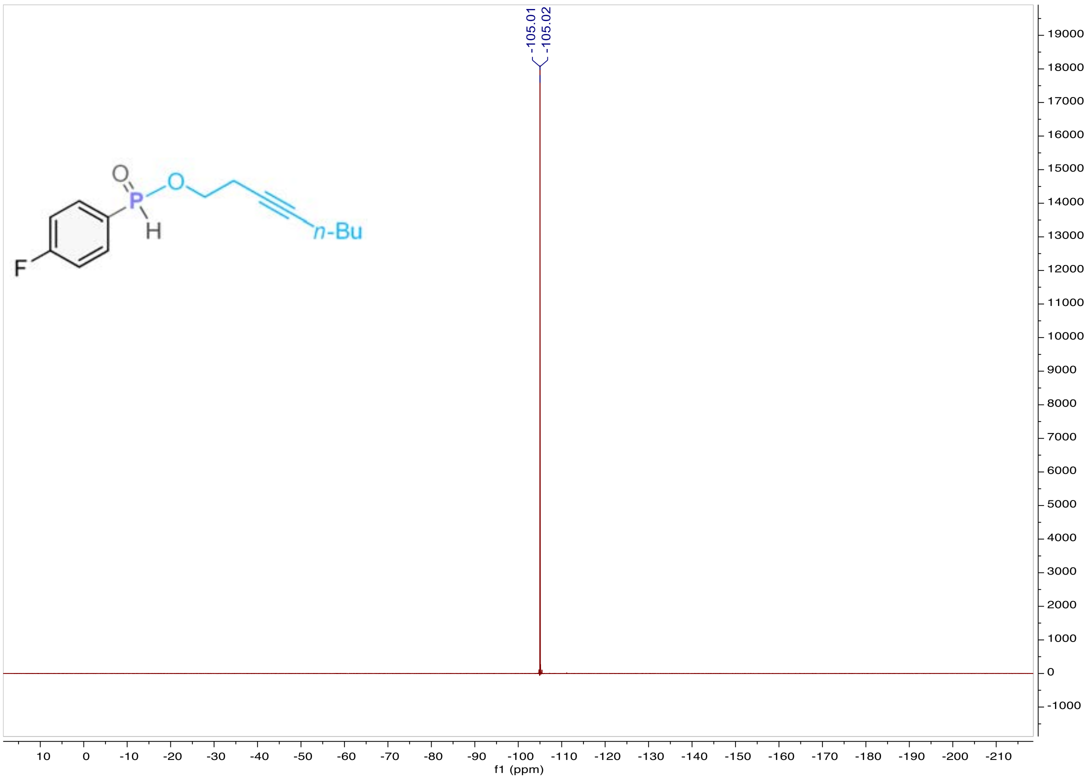

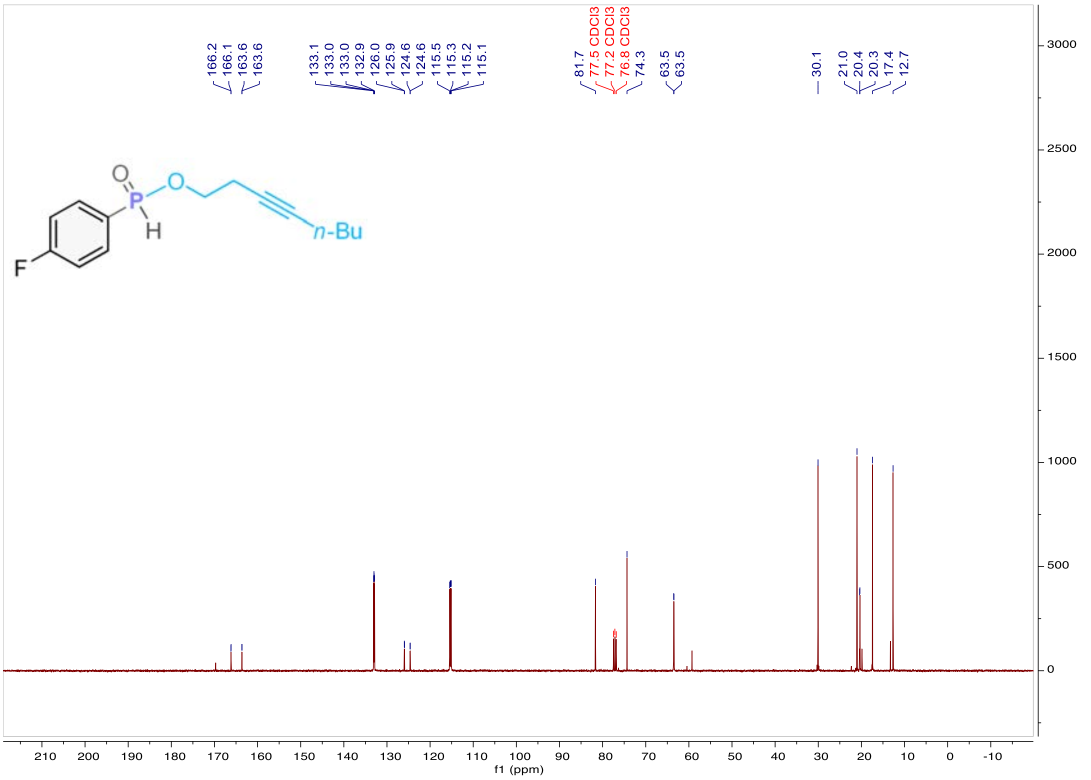

Figure S23. ^{1}H NMR, ^{31}P NMR, ^{19}F NMR and ^{13}C NMR spectra for **1f**

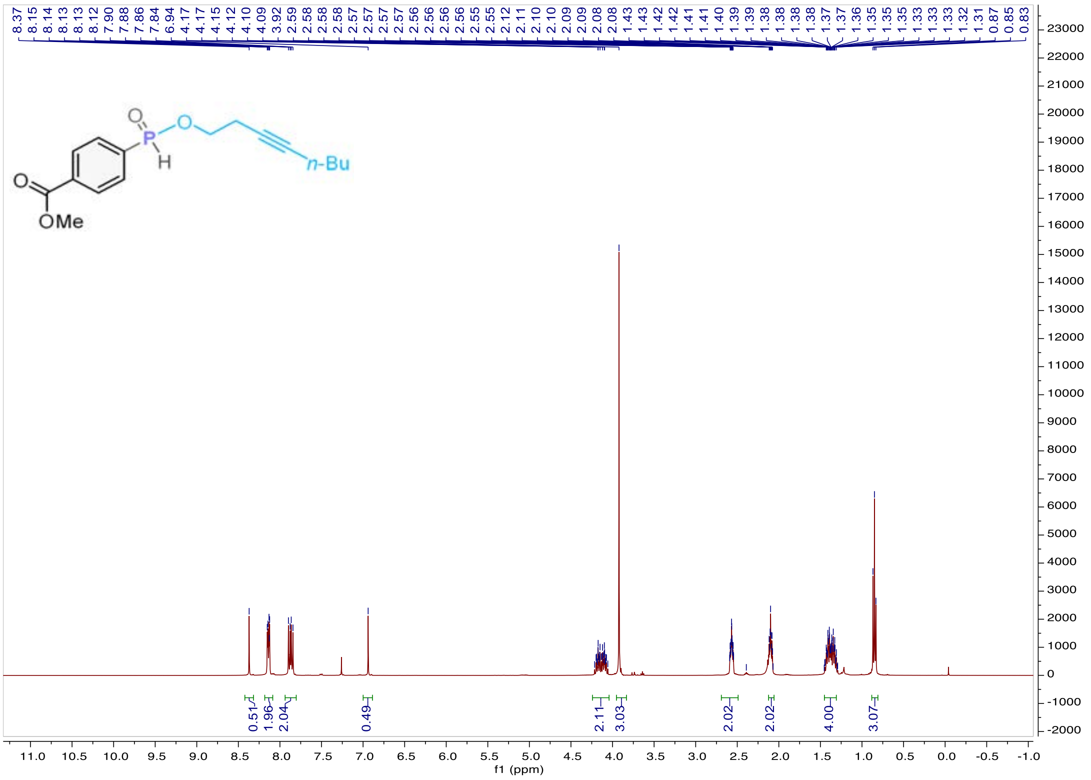
O
P
H
O
n-Bu
O
OMe
f1 (ppm)

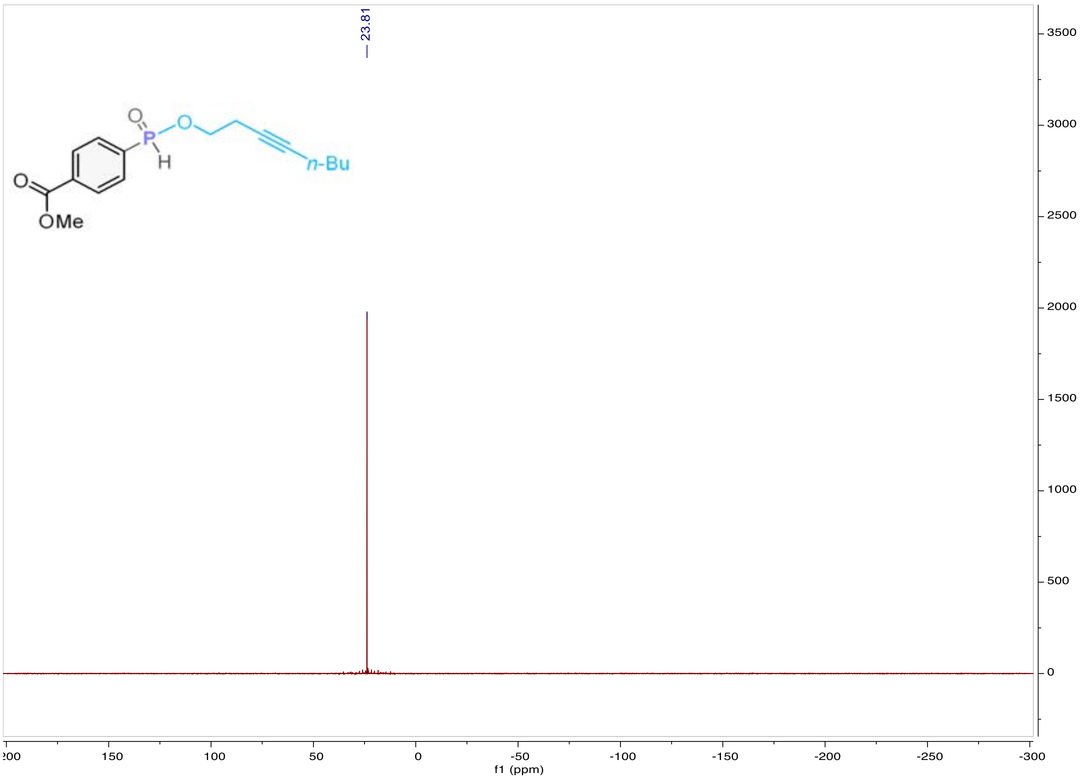
23.81
O
P
H
O
n-Bu
O
OMe
f1 (ppm)

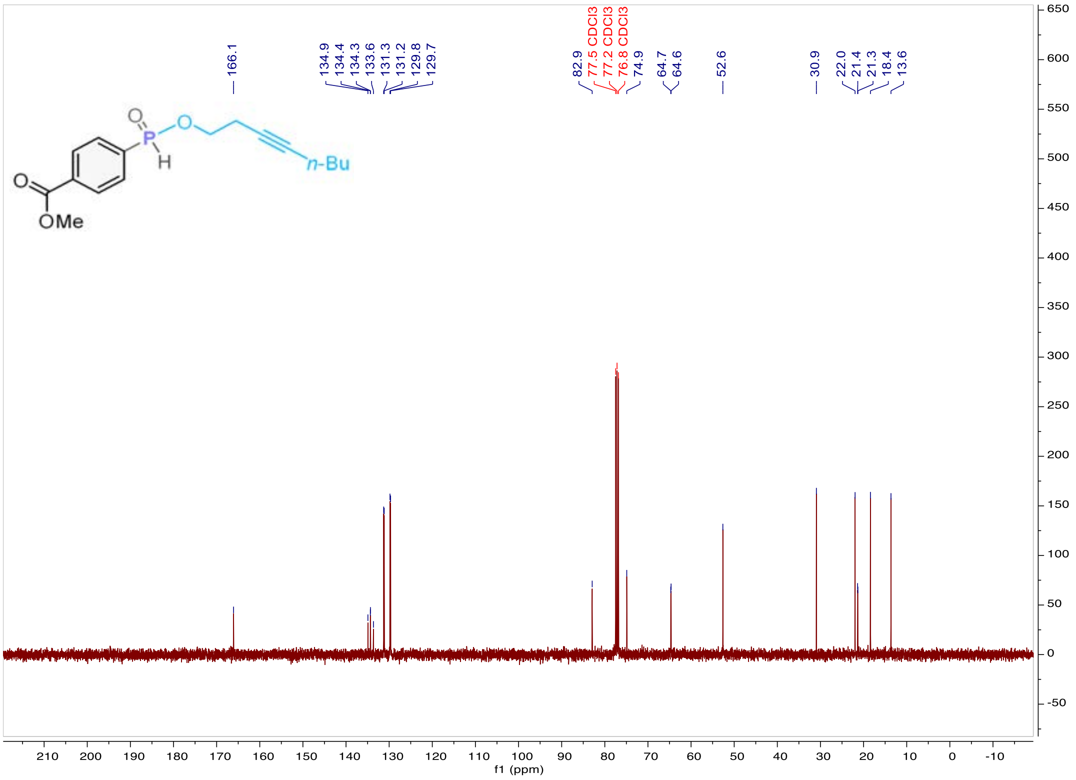

Figure S24. ^{1}H NMR, ^{31}P NMR and ^{13}C NMR spectra for **1g**

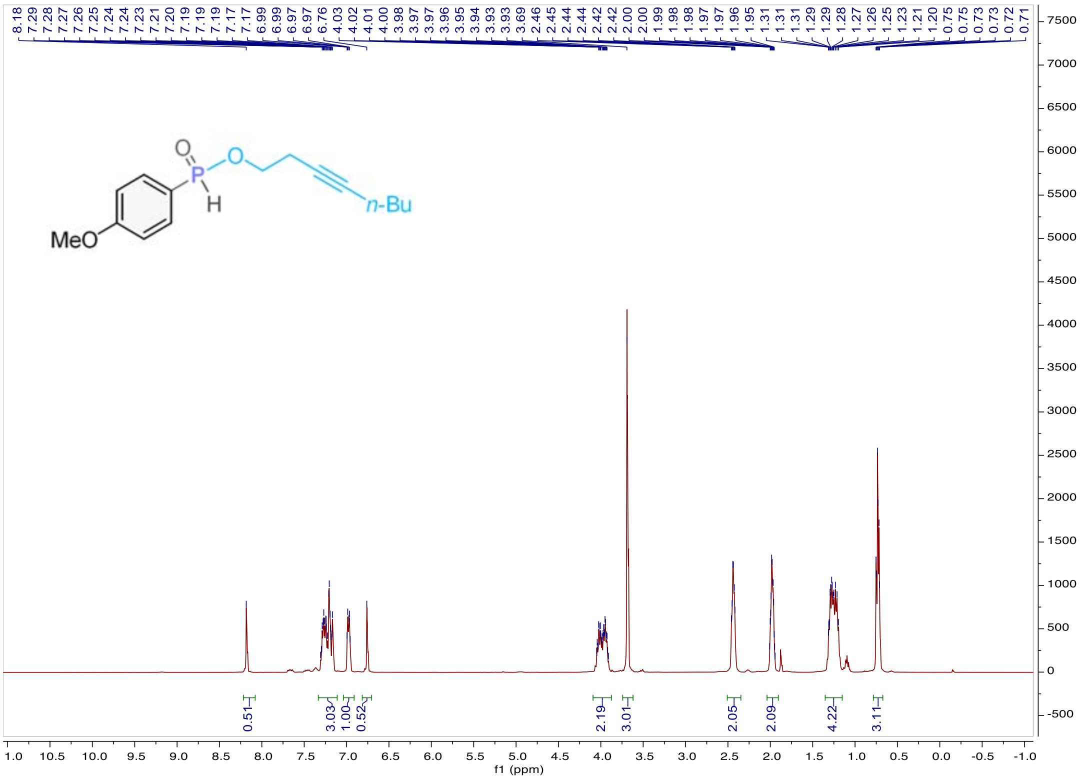
MeO
n-Bu
f1 (ppm)

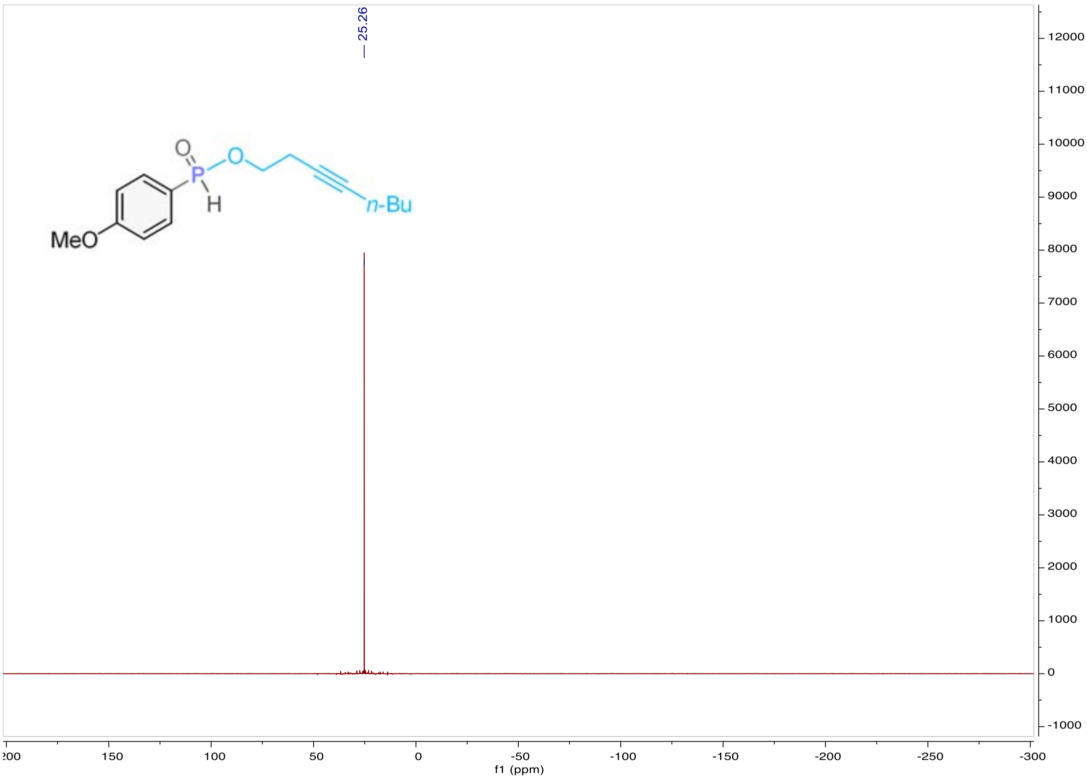
25.26
MeO
n-Bu
f1 (ppm)

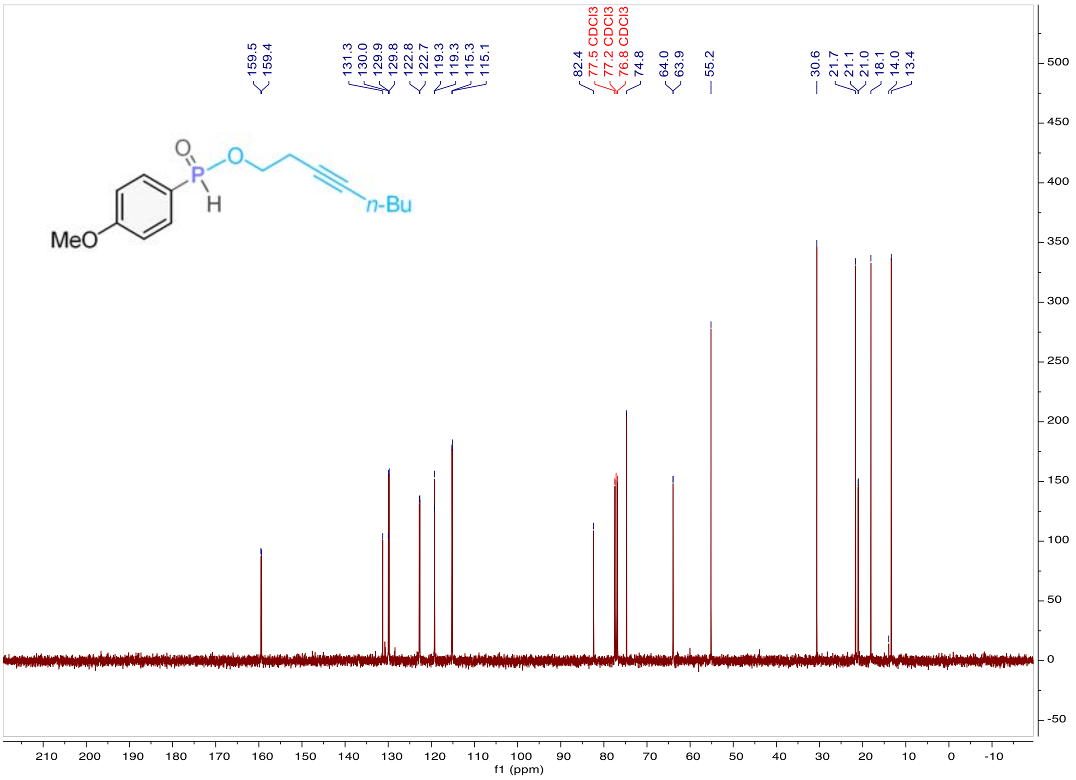

Figure S25. ^{1}H NMR, ^{31}P NMR and ^{13}C NMR spectra for **1h**

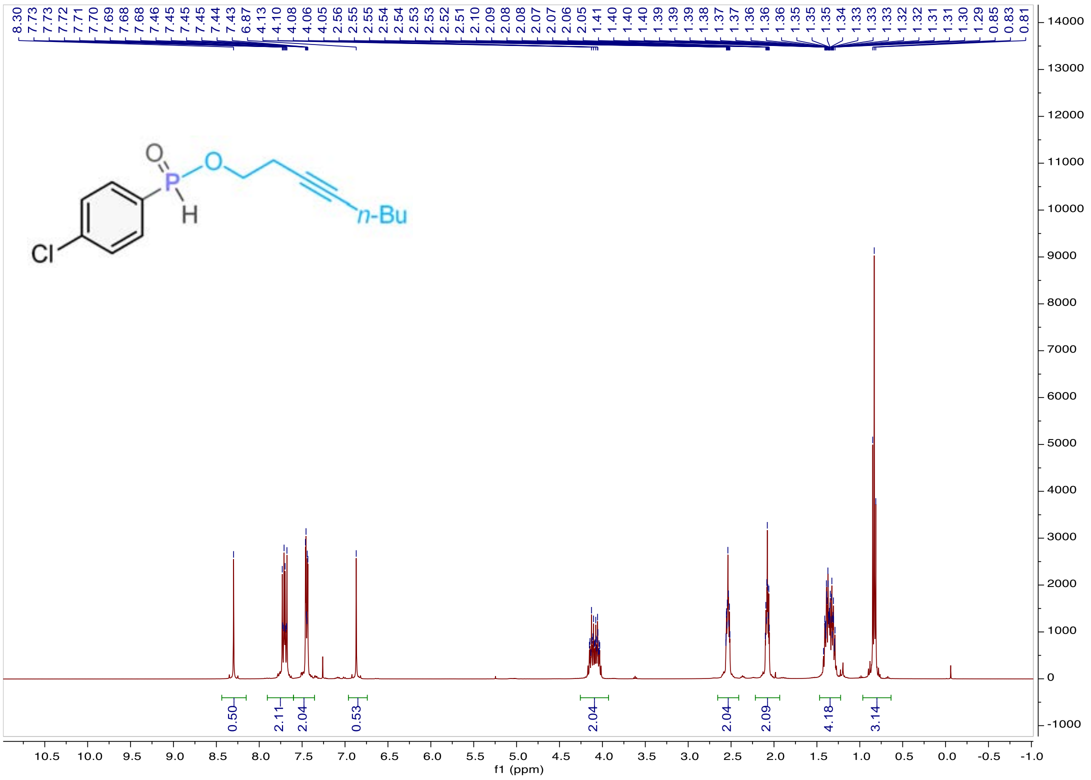

8.30 7.73 7.73 7.72 7.71 7.70 7.69 7.68 7.46 7.45 7.45 7.45 7.44 7.43 6.87 4.13 4.10 4.08 4.06 4.05 2.56 2.55 2.55 2.54 2.54 2.53 2.53 2.52 2.51 2.10 2.09 2.09 2.08 2.08 2.07 2.06 2.05 1.41 1.40 1.40 1.39 1.39 1.39 1.38 1.37 1.37 1.36 1.36 1.36 1.35 1.35 1.35 1.34 1.33 1.33 1.33 1.32 1.32 1.31 1.31 1.30 1.29 0.85 0.83 0.81
O
P
O
H
Cl
n-Bu
0.50
2.11
2.04
0.53
2.04
2.04
2.09
4.18
3.14
f1 (ppm)

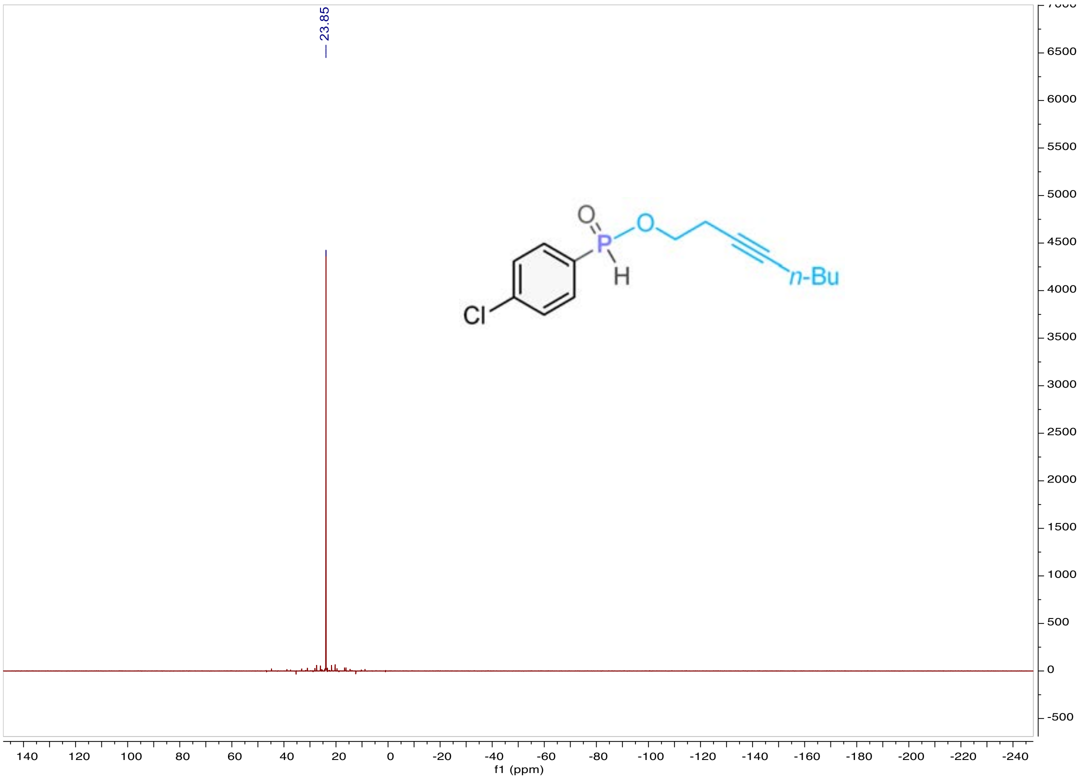

23.85
O
P
O
H
Cl
n-Bu
f1 (ppm)

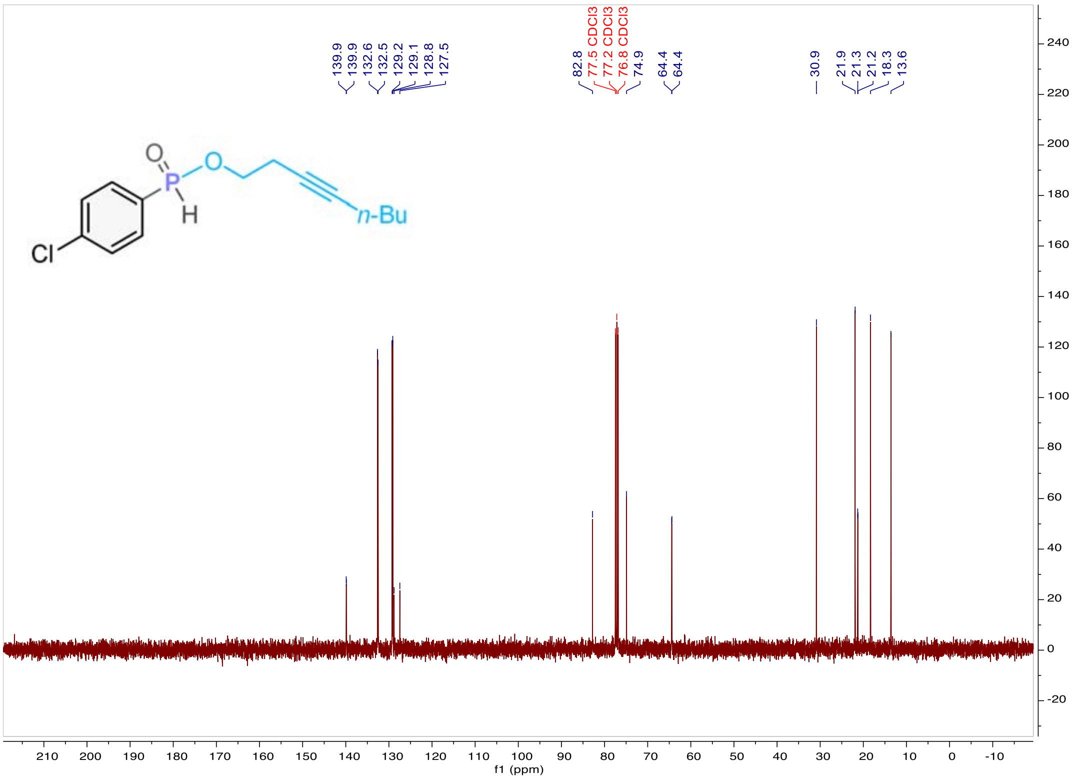

Figure S26. ^{1}H NMR, ^{31}P NMR and ^{13}C NMR spectra for **1i**

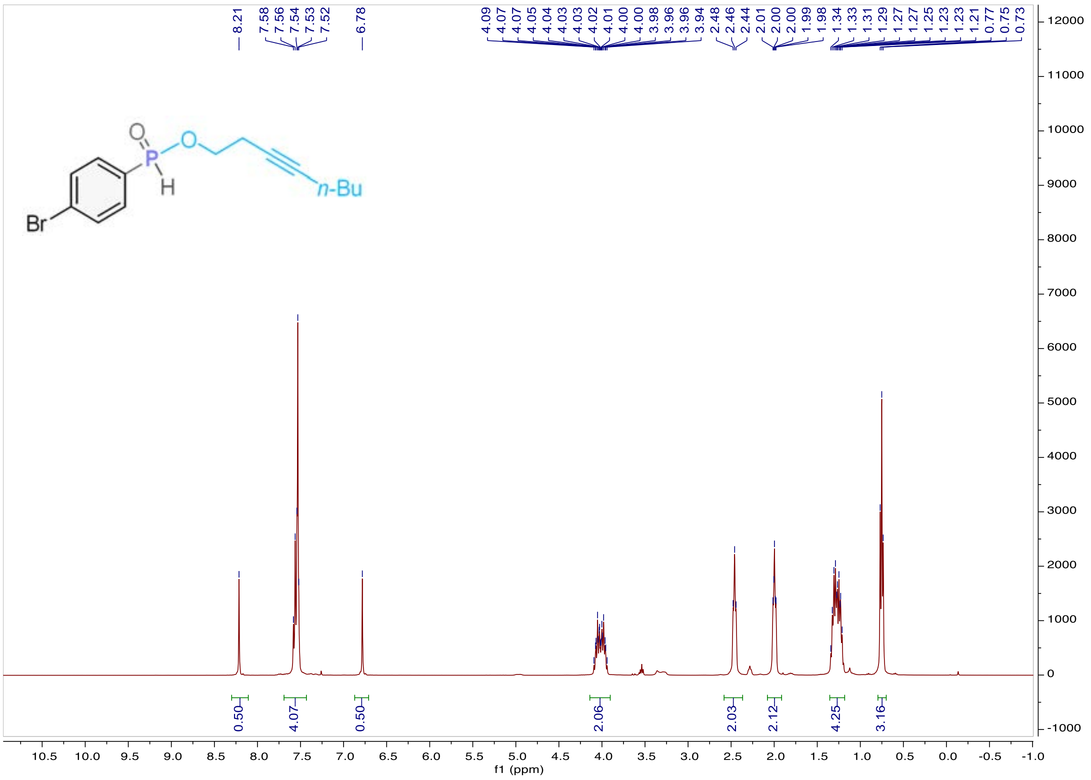

8.21
7.58
7.56
7.54
7.53
7.52
6.78
4.09
4.07
4.07
4.05
4.04
4.03
4.03
4.02
4.01
4.00
4.00
3.98
3.96
3.96
3.94
2.48
2.46
2.44
2.01
2.00
2.00
1.99
1.98
1.34
1.33
1.31
1.29
1.27
1.27
1.25
1.23
1.23
1.21
0.77
0.75
0.73
O
P
O
H
n-Bu
Br
0.50
4.07
0.50
2.06
2.03
2.12
4.25
3.16
f1 (ppm)

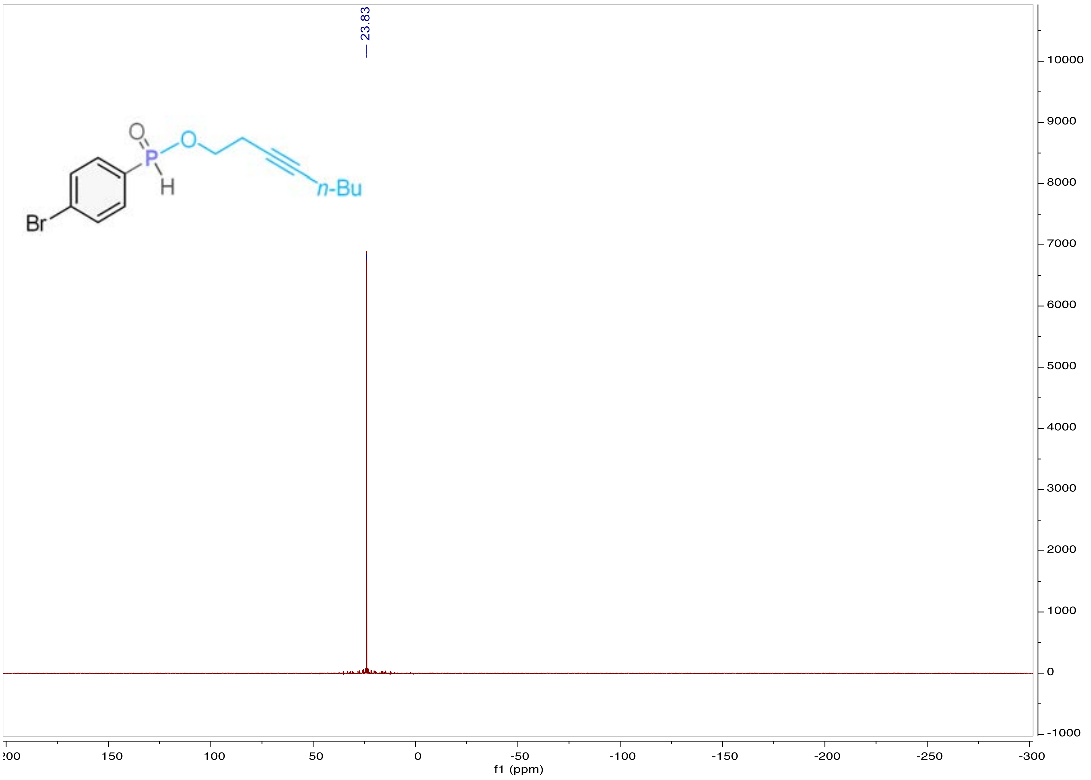

23.83
O
P
O
H
n-Bu
Br
f1 (ppm)

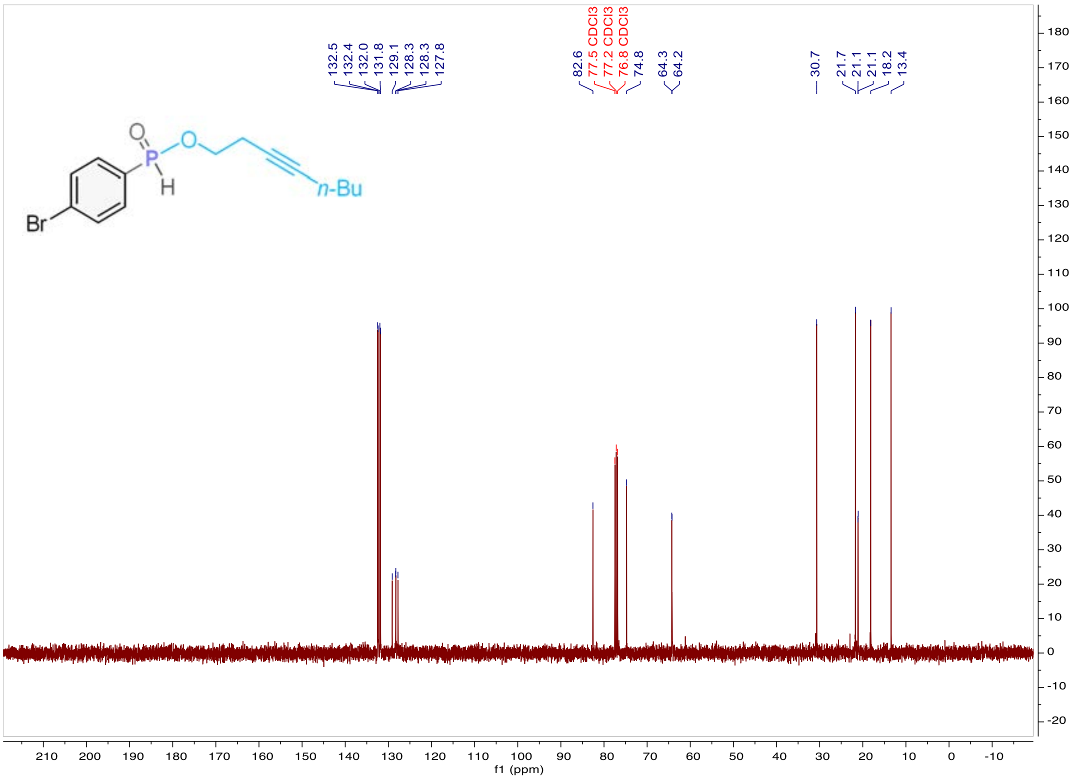

Figure S27. ^{1}H NMR, ^{31}P NMR and ^{13}C NMR spectra for **1j**

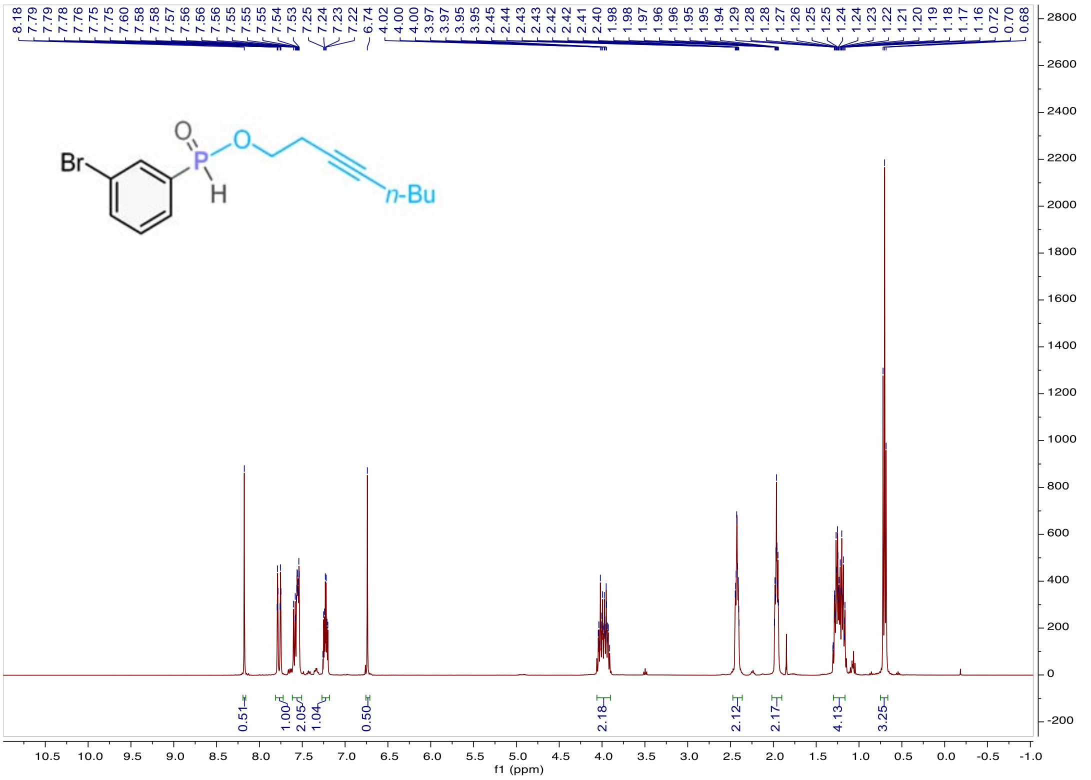

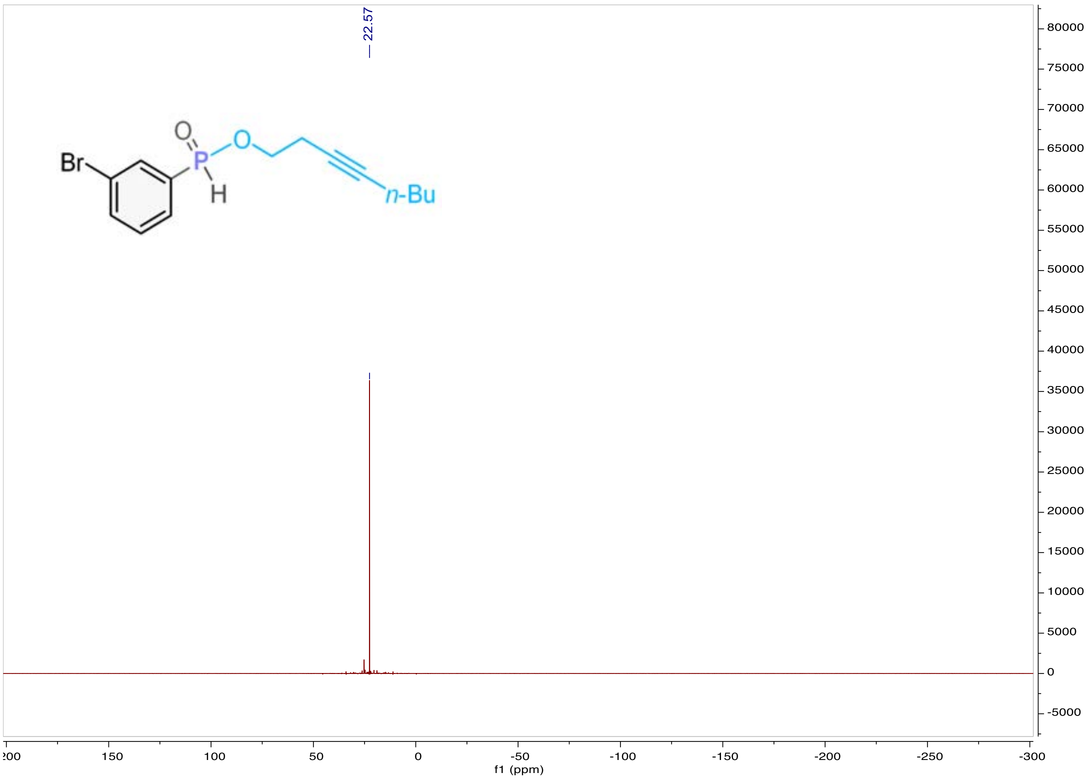

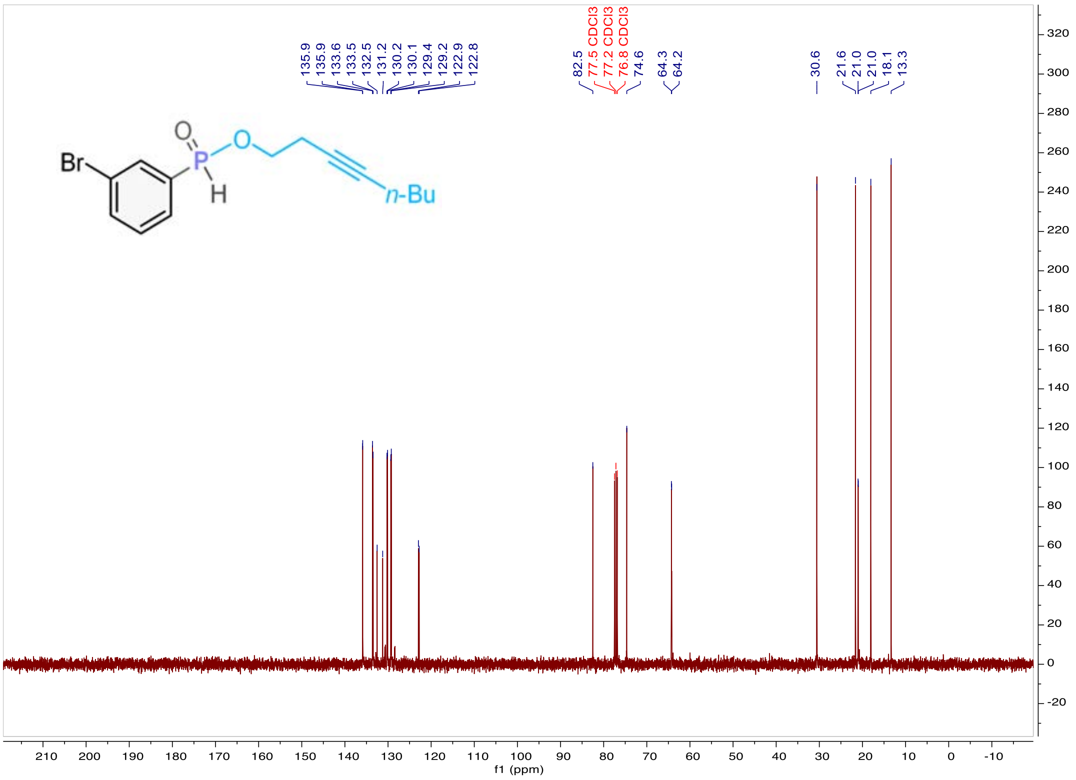

Figure S28. ^{1}H NMR, ^{31}P NMR and ^{13}C NMR spectra for **1k**

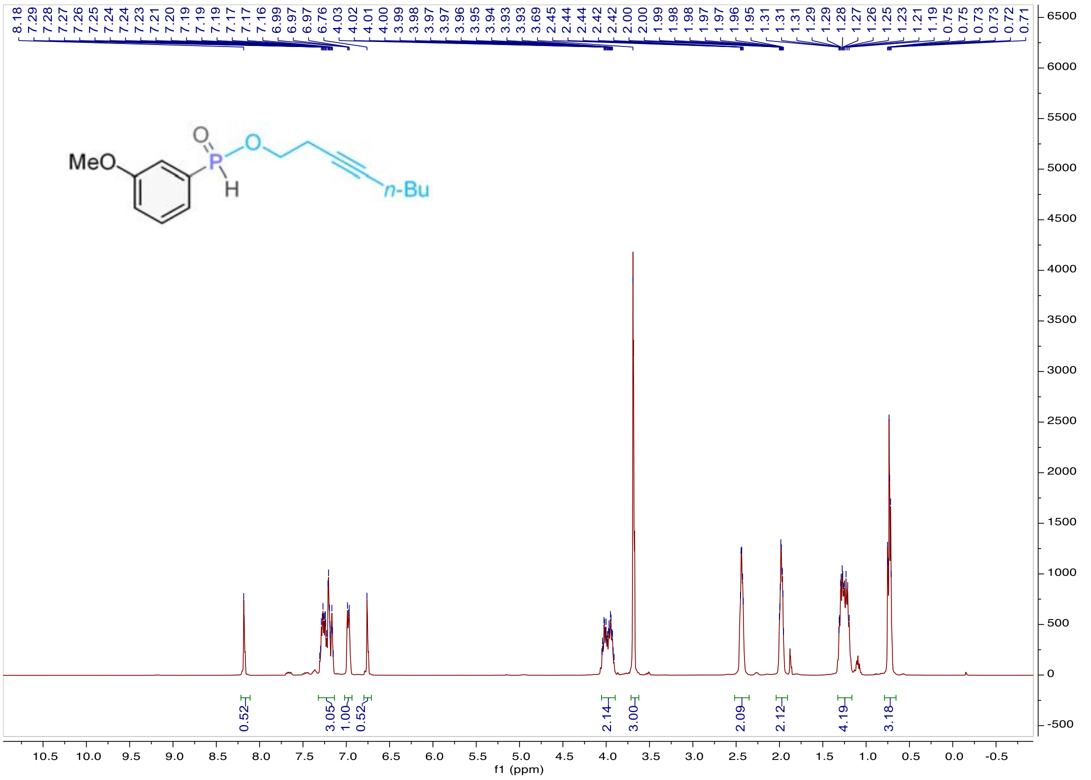

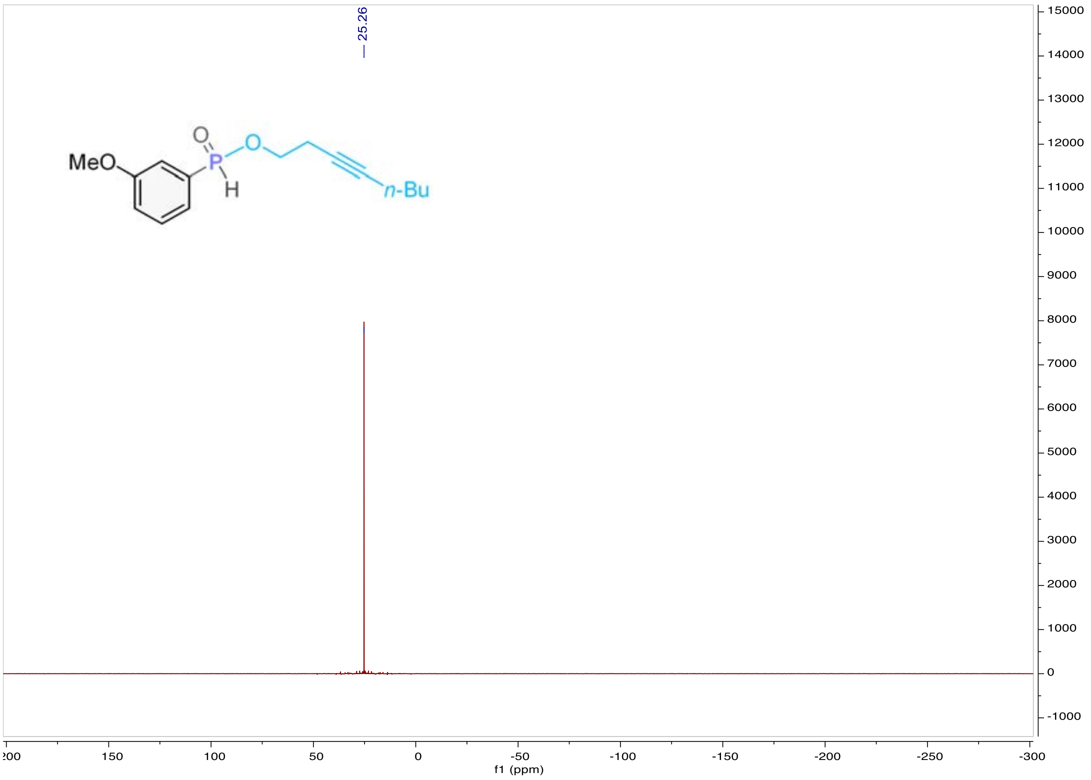

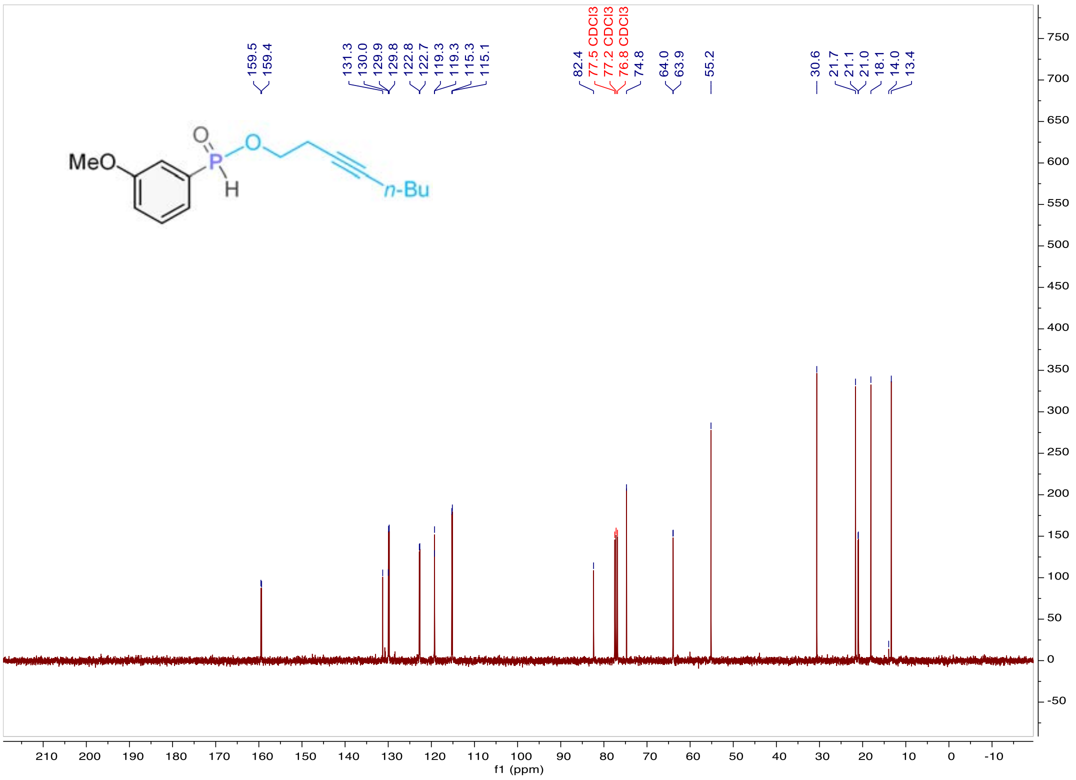

Figure S29. ^{1}H NMR, ^{31}P NMR and ^{13}C NMR spectra for **1l**

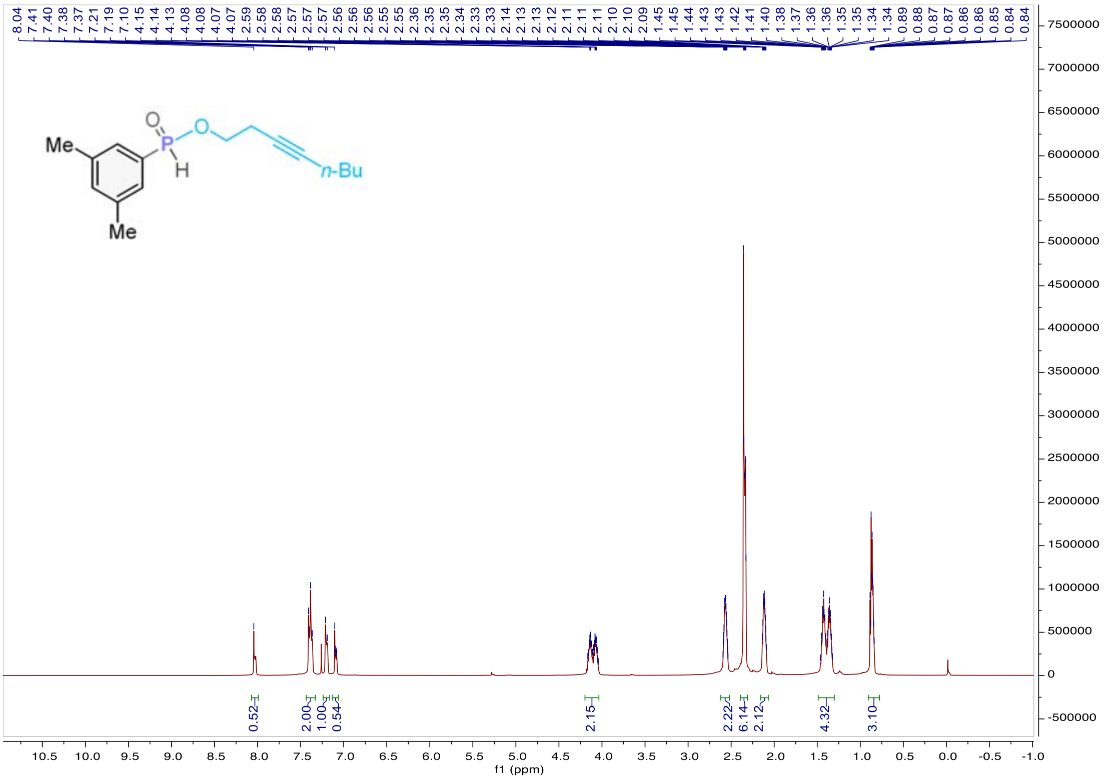

Me
Me
O
P
H
O
n-Bu
f1 (ppm)

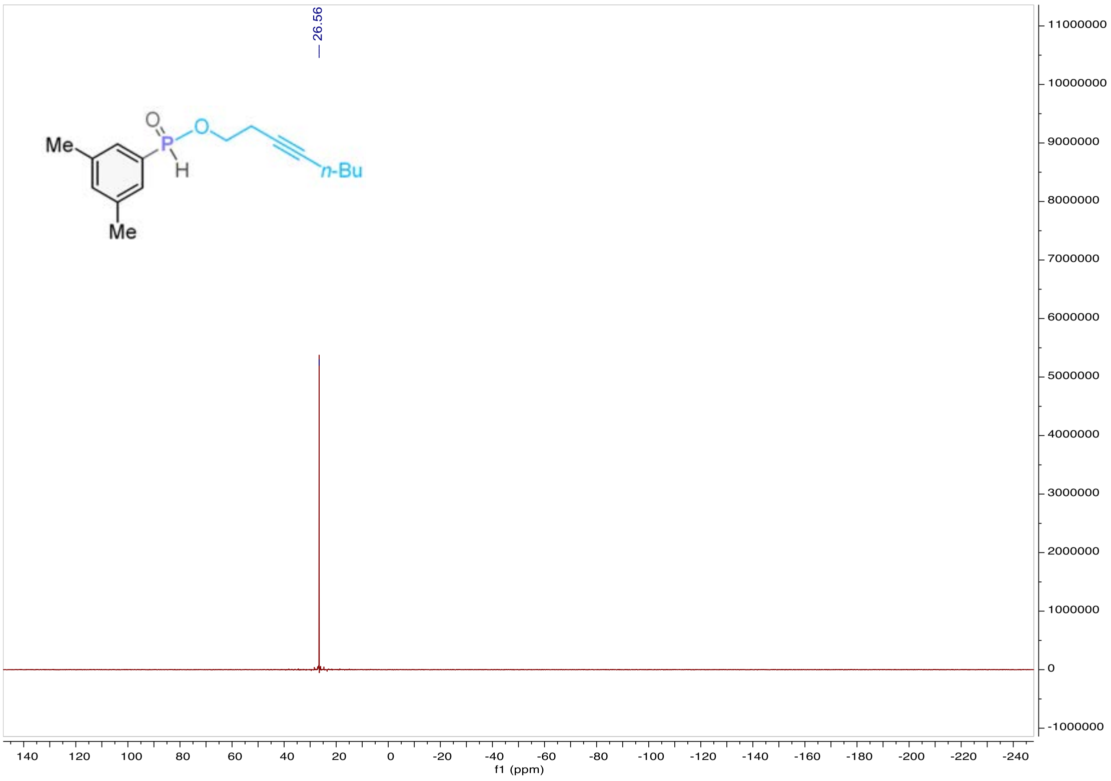

26.56
Me
Me
O
P
H
O
n-Bu
f1 (ppm)

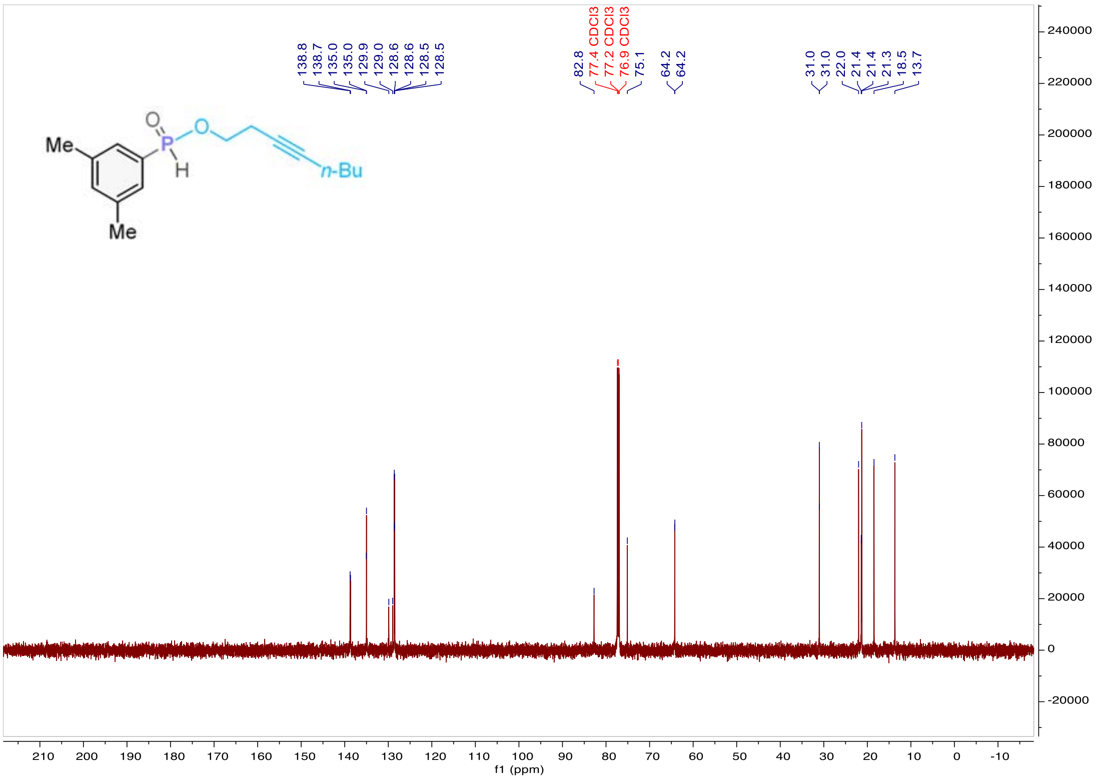

Figure S30. ^{1}H NMR, ^{31}P NMR and ^{13}C NMR spectra for **1m**

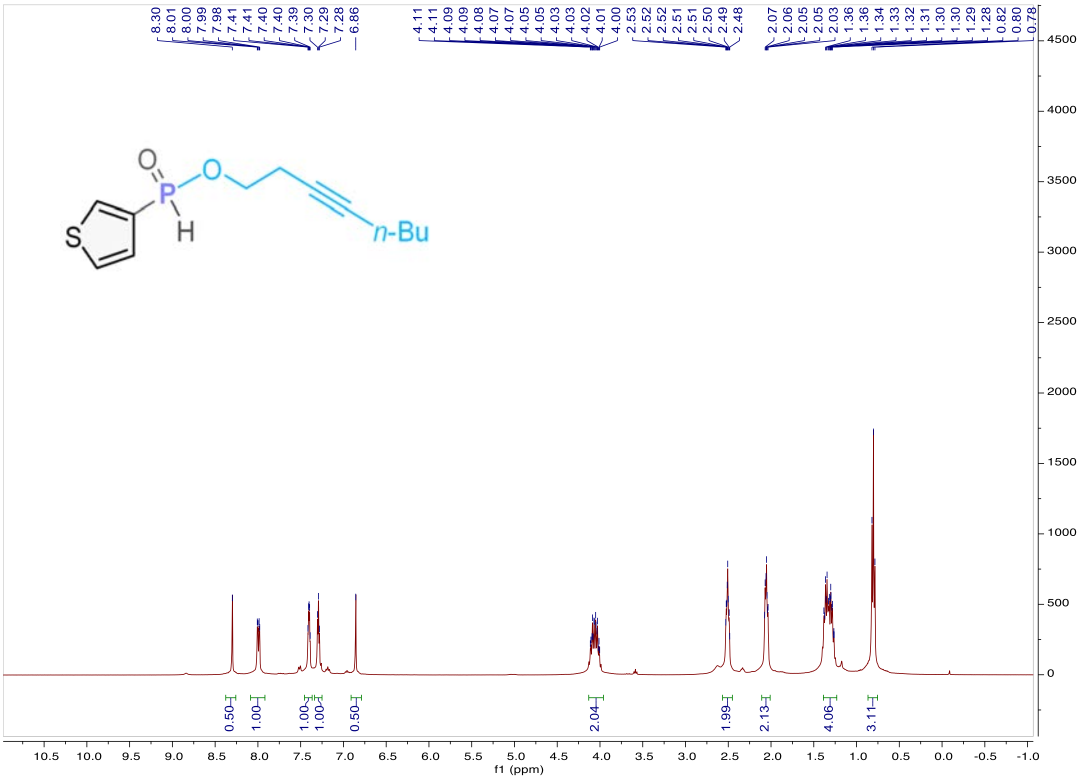

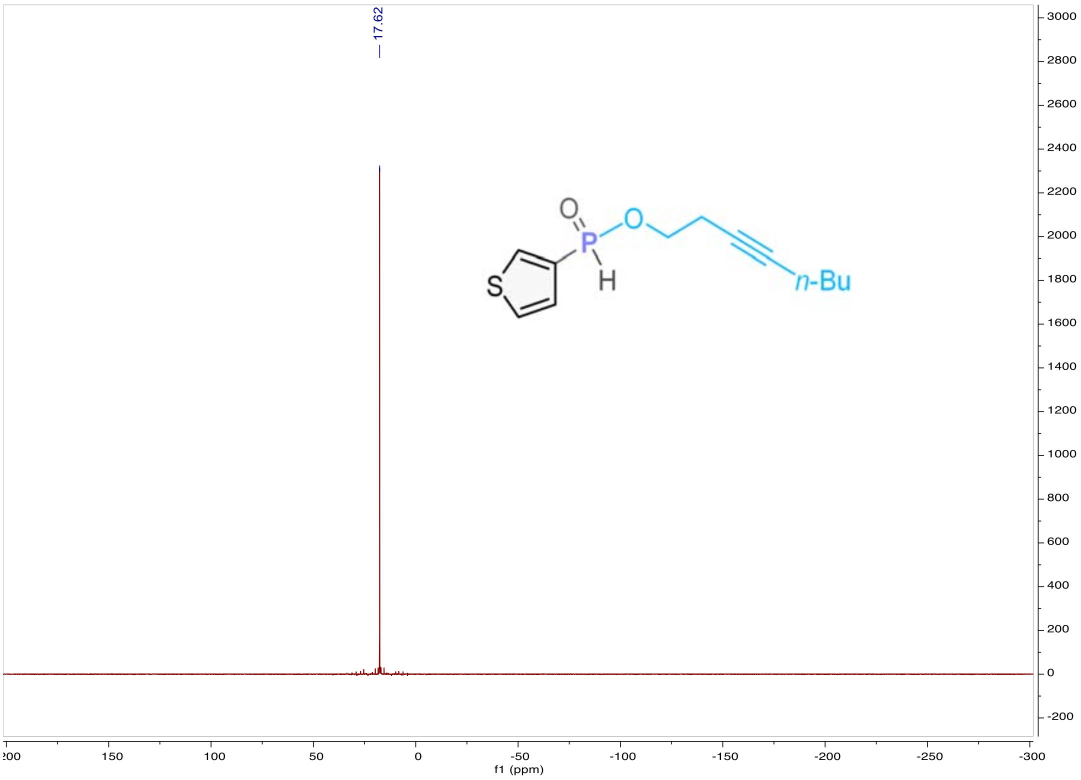

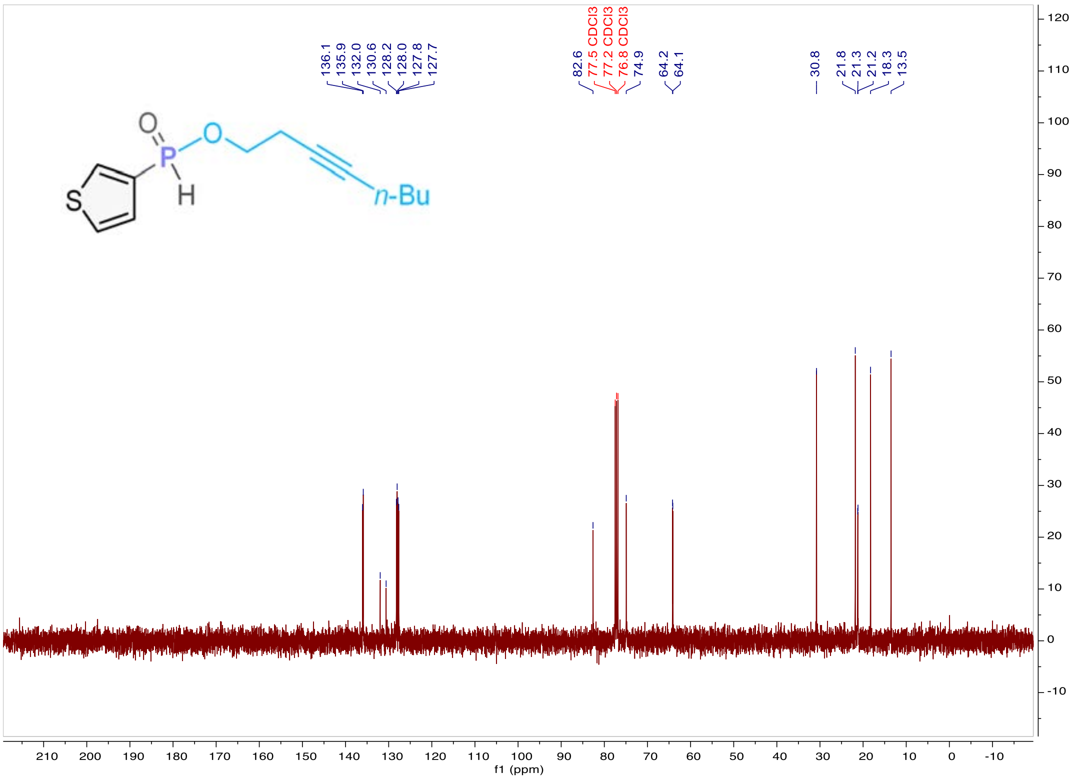

Figure S31. ^{1}H NMR, ^{31}P NMR and ^{13}C NMR spectra for **1n**

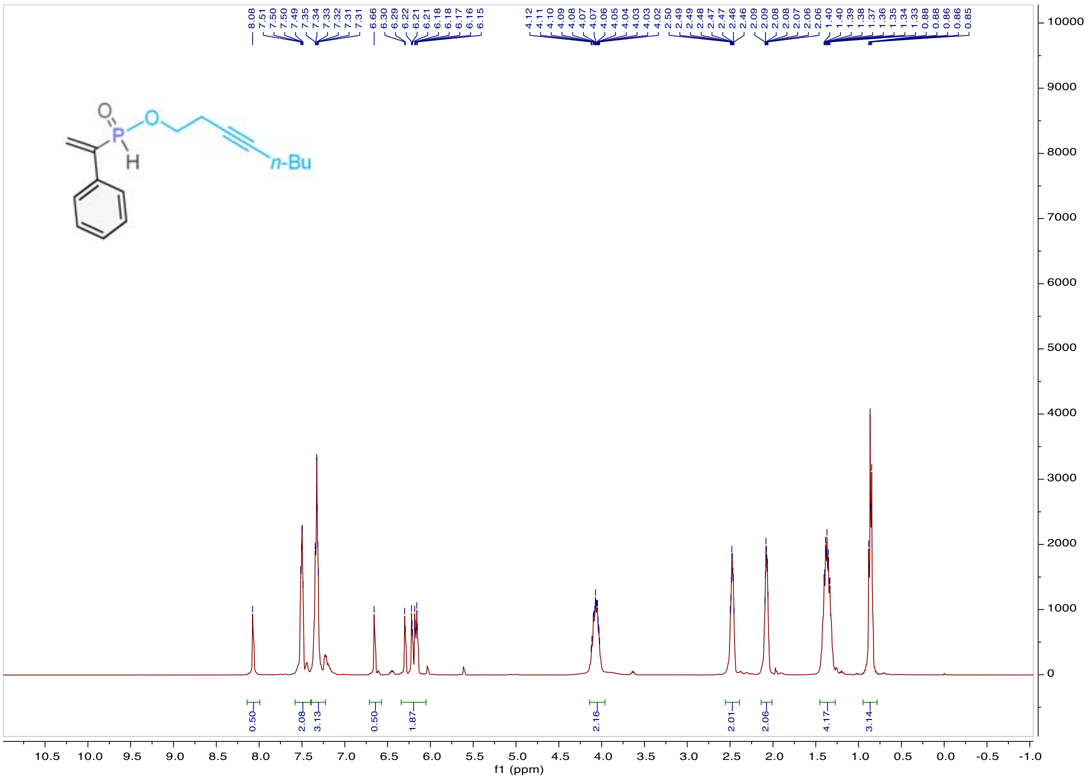

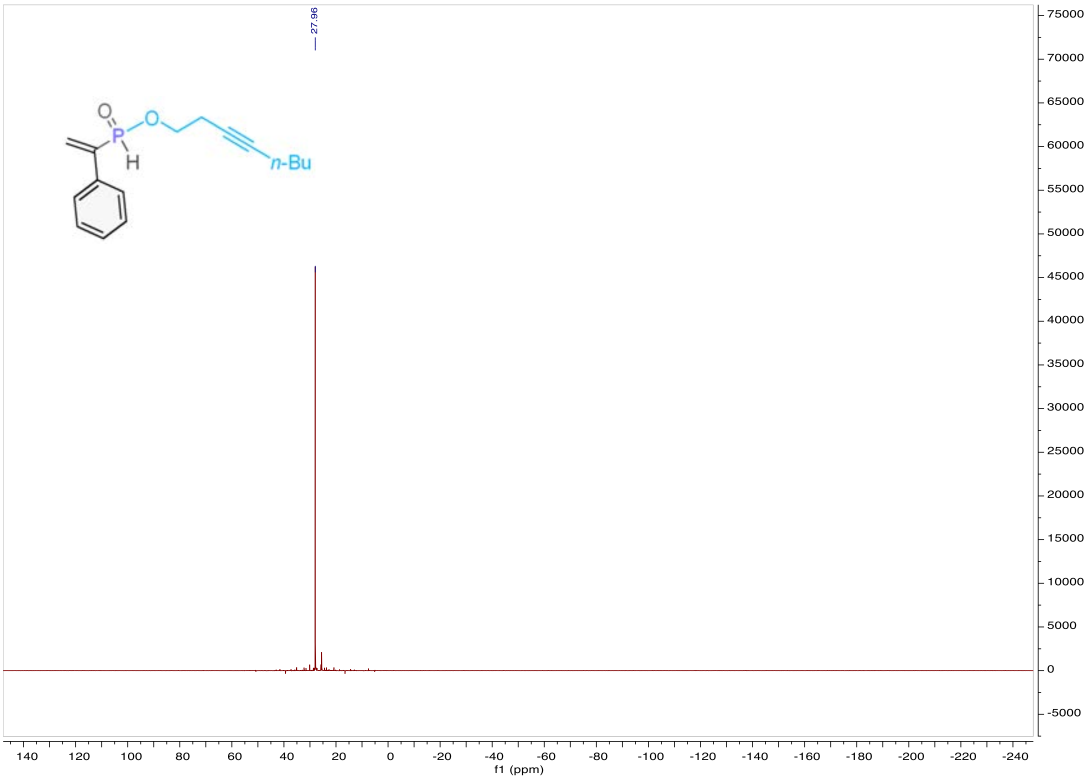

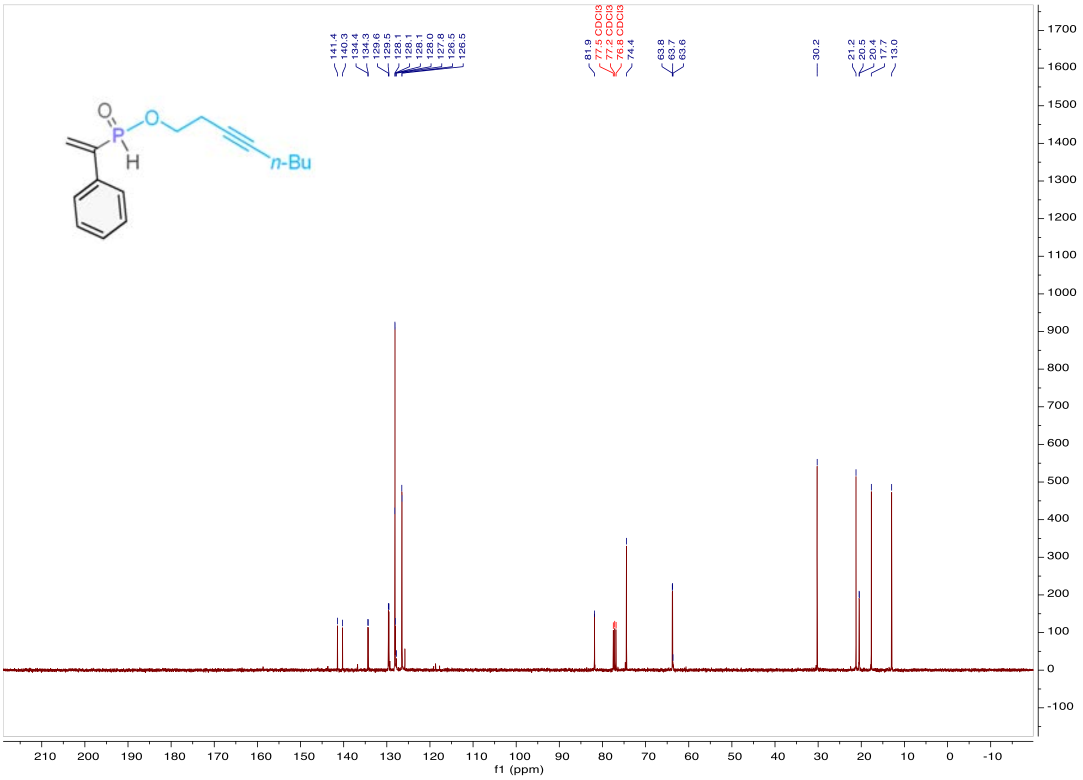

Figure S32. ^{1}H NMR, ^{31}P NMR and ^{13}C NMR spectra for **1o**

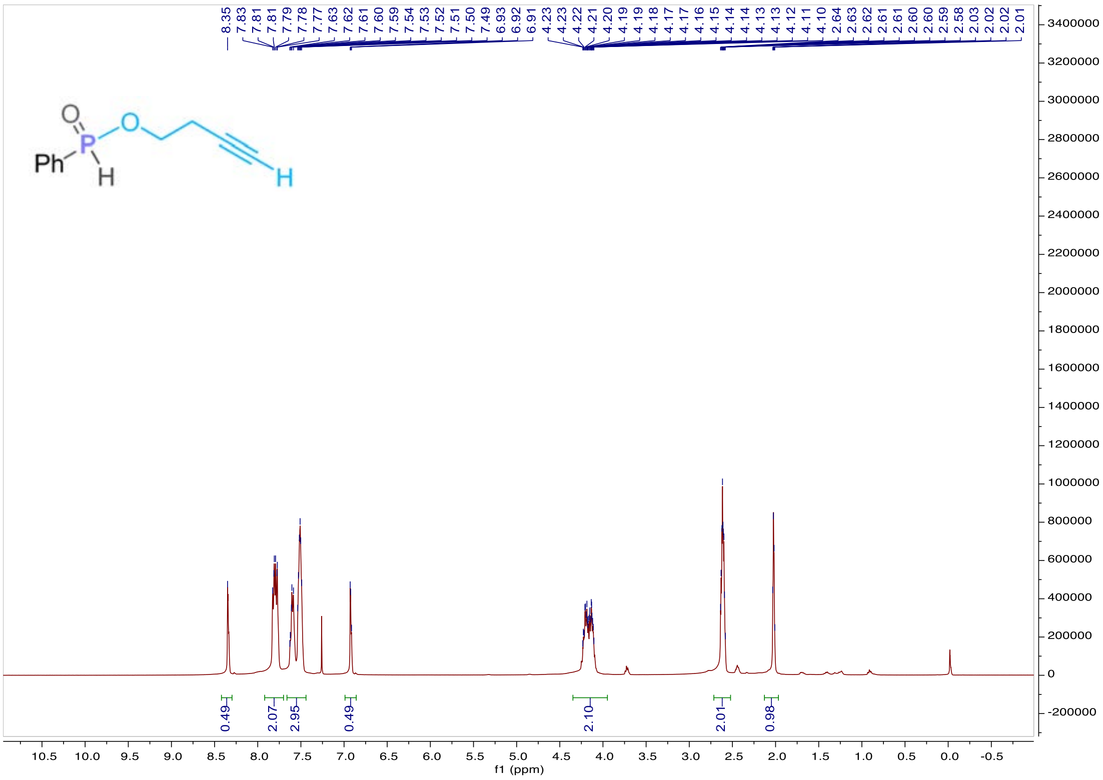

8.35
7.83
7.81
7.81
7.79
7.78
7.77
7.63
7.62
7.61
7.60
7.59
7.54
7.53
7.52
7.51
7.50
7.49
6.93
6.92
6.91
4.23
4.23
4.22
4.21
4.20
4.19
4.19
4.18
4.17
4.16
4.15
4.14
4.14
4.13
4.13
4.12
4.11
4.10
2.64
2.63
2.62
2.61
2.60
2.59
2.58
2.03
2.02
2.02
2.01
Ph
H
O
P
O
H
0.49
2.07
2.95
0.49
2.10
2.01
0.98
f1 (ppm)

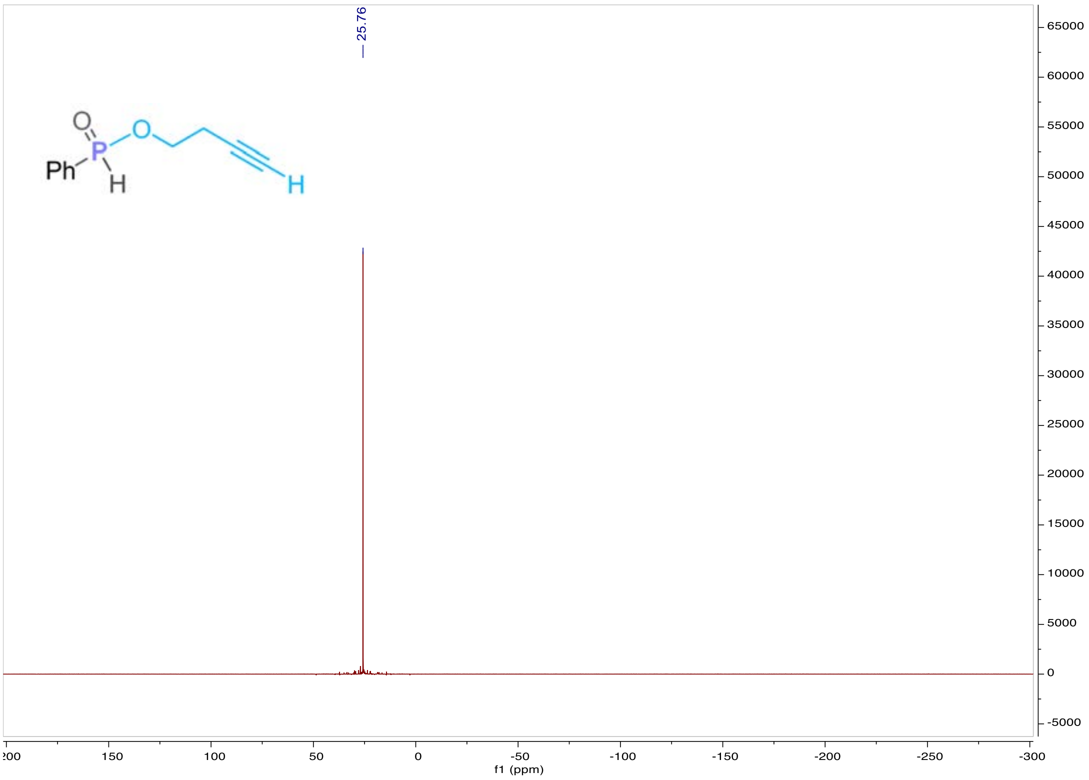

25.76
Ph
H
O
P
O
H
f1 (ppm)

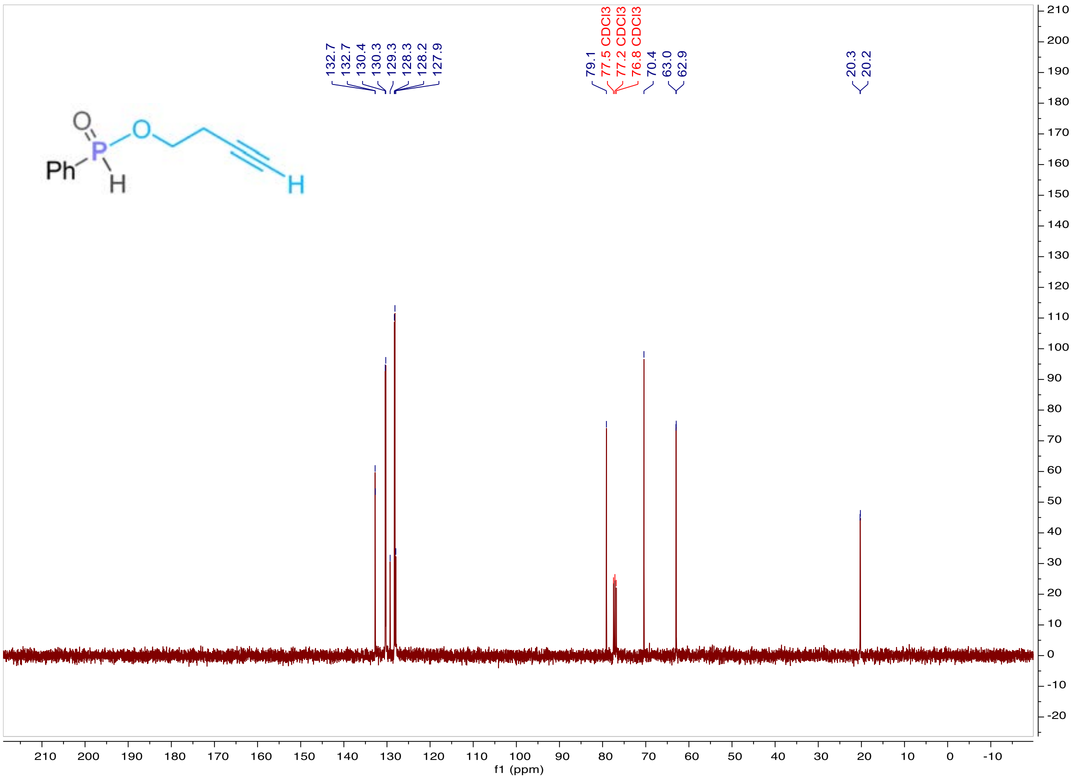

Figure S33. ^{1}H NMR, ^{31}P NMR and ^{13}C NMR spectra for **1p**

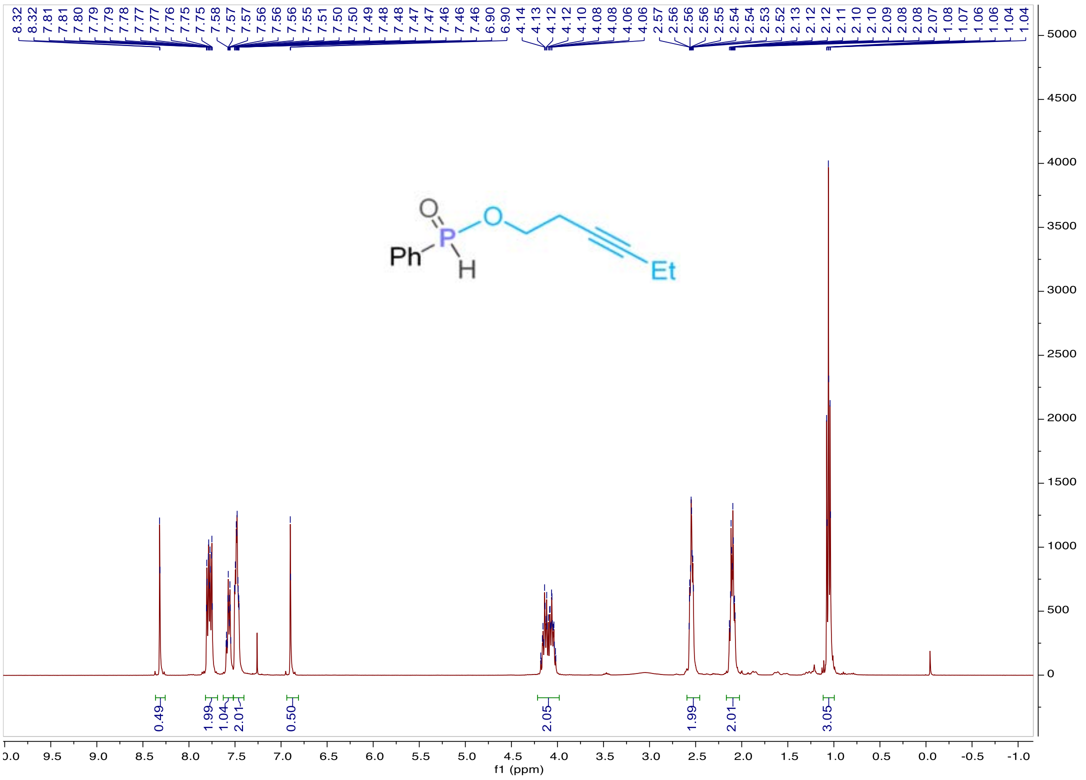
Ph
P
O
O
H
Et
f1 (ppm)

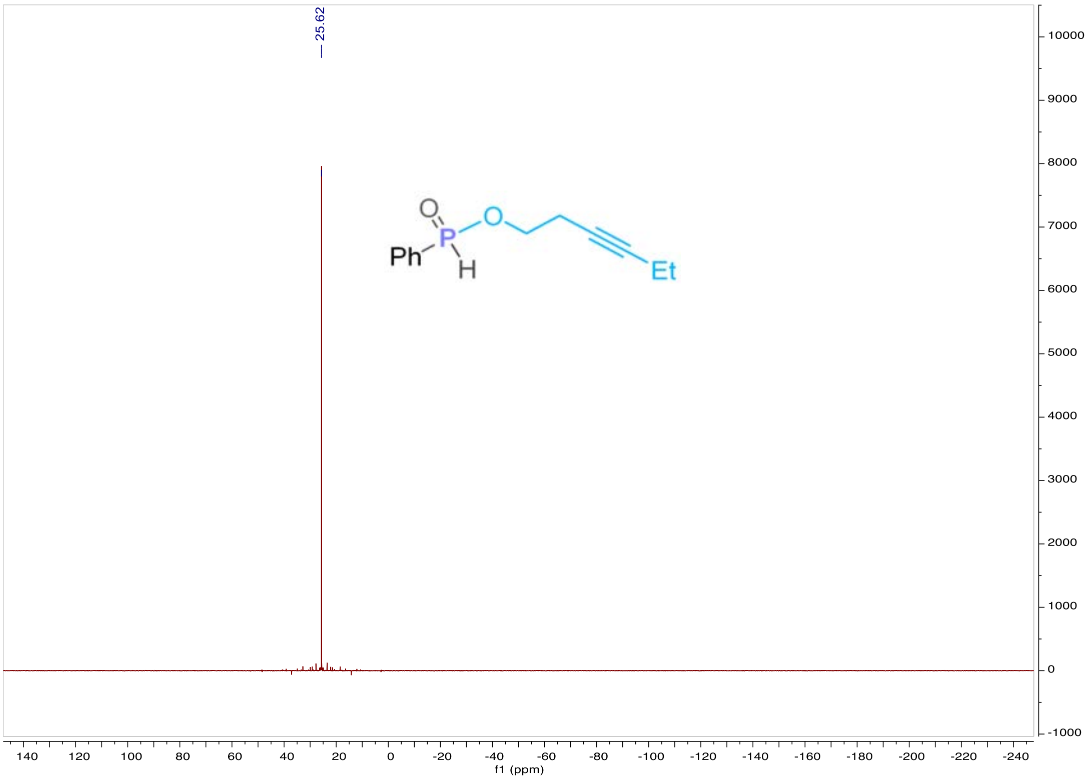
25.62
Ph
P
O
O
H
Et
f1 (ppm)

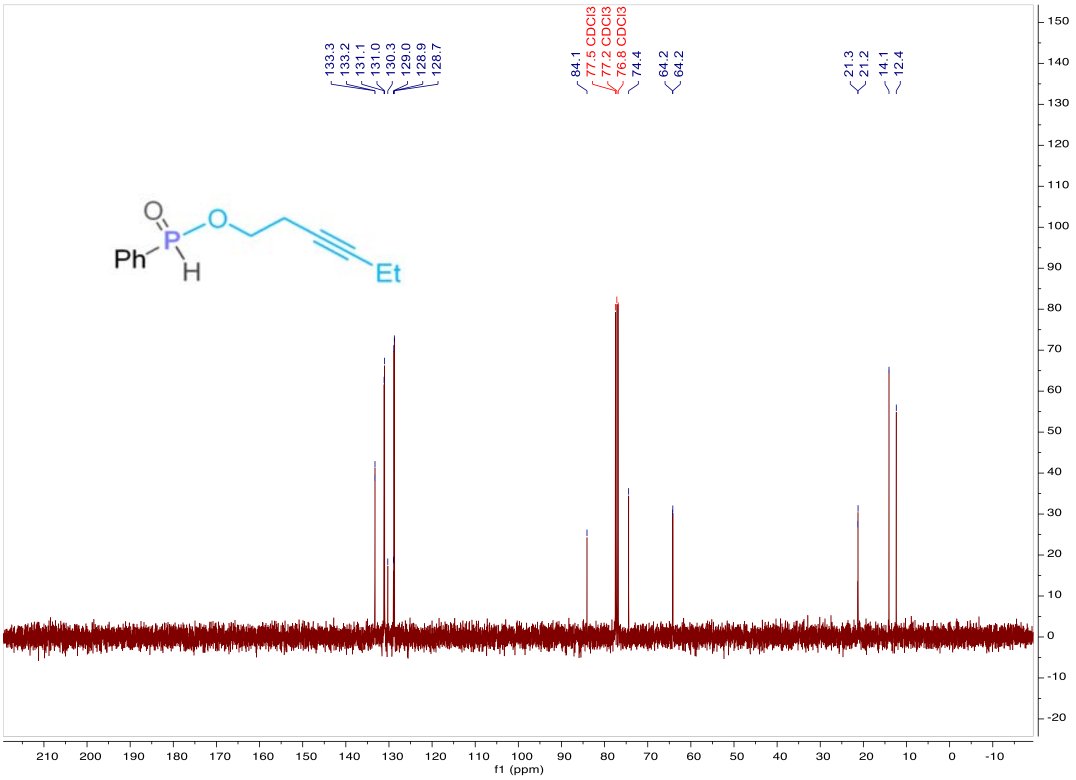

Figure S34. ^{1}H NMR, ^{31}P NMR and ^{13}C NMR spectra for **1q**

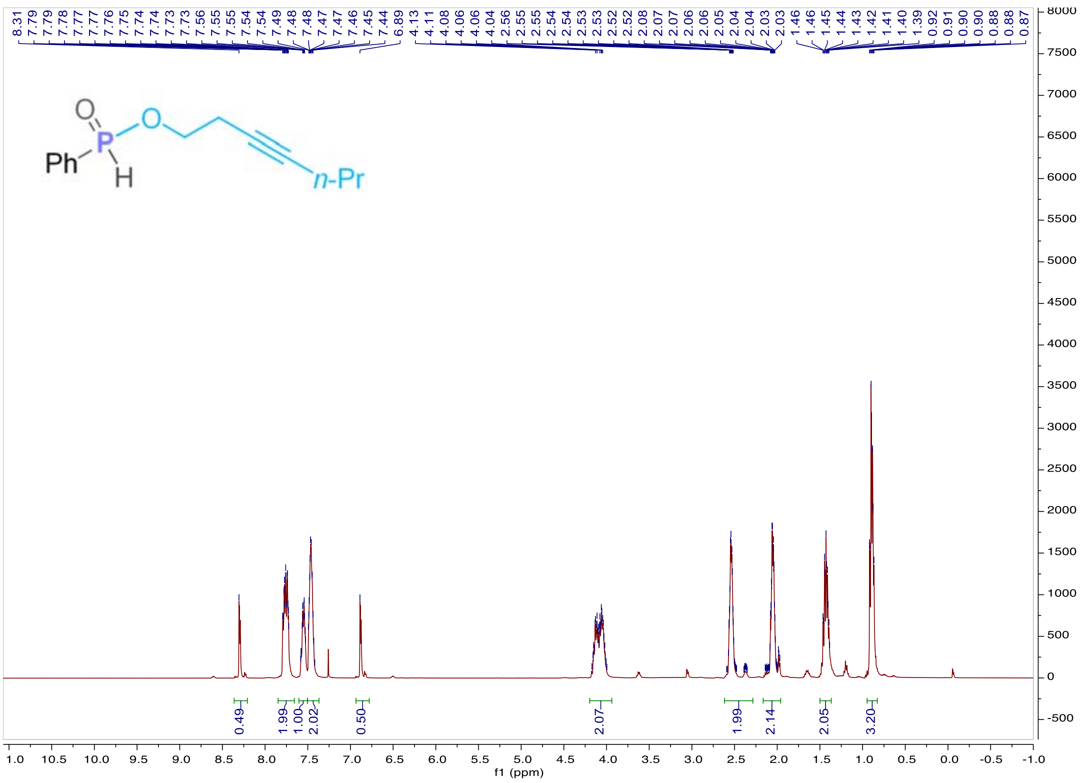

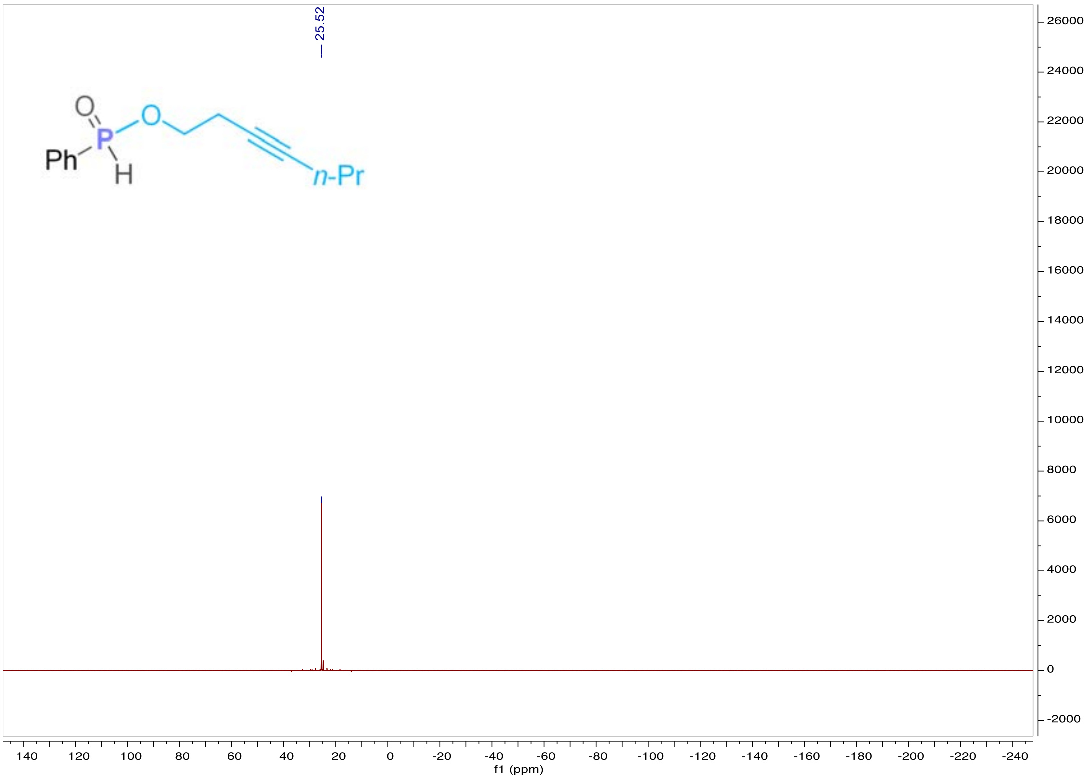

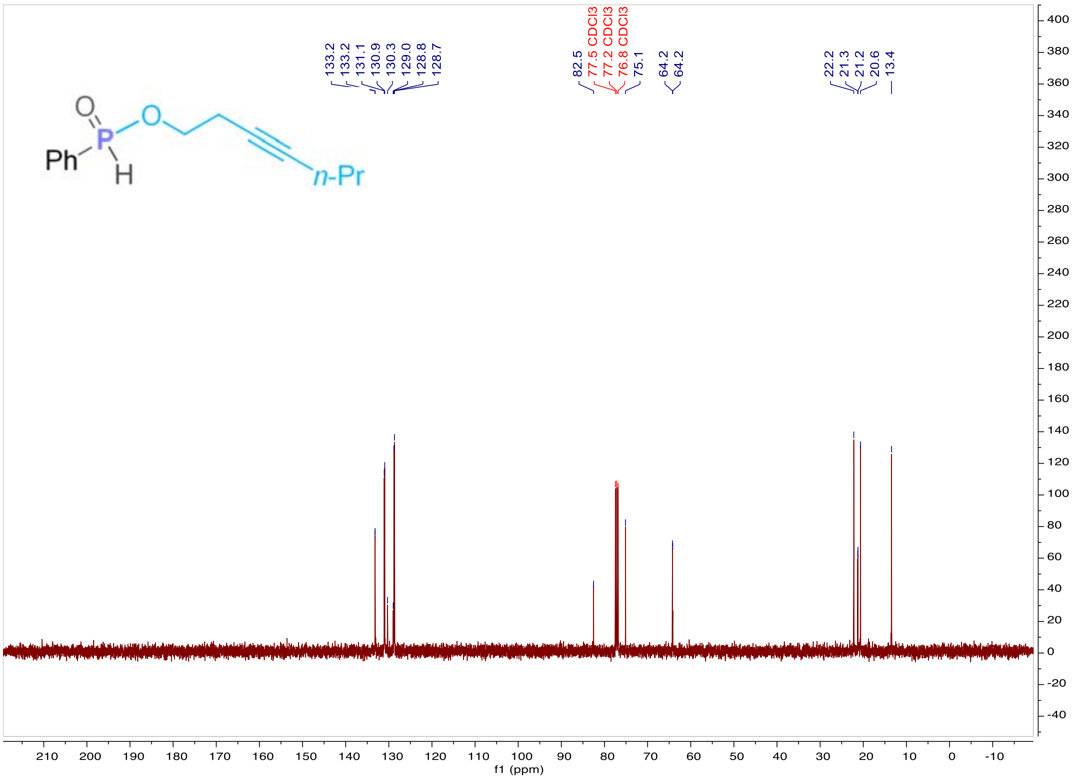

Figure S35. ^{1}H NMR, ^{31}P NMR and ^{13}C NMR spectra for **1r**

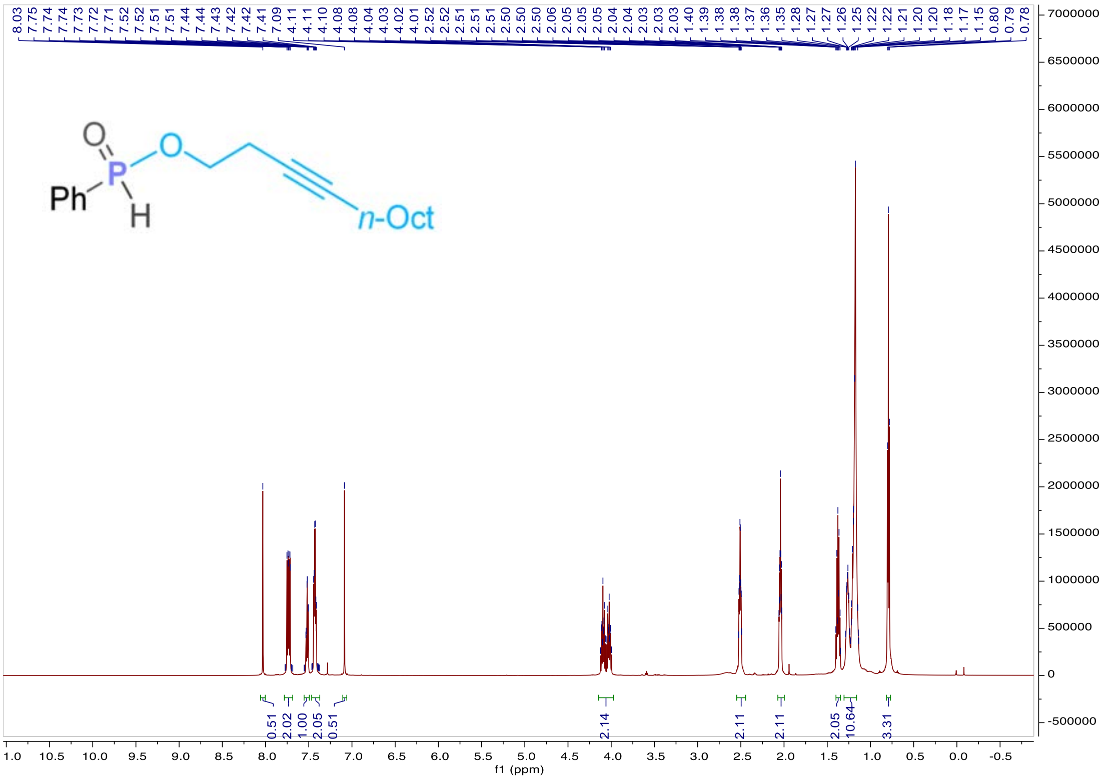

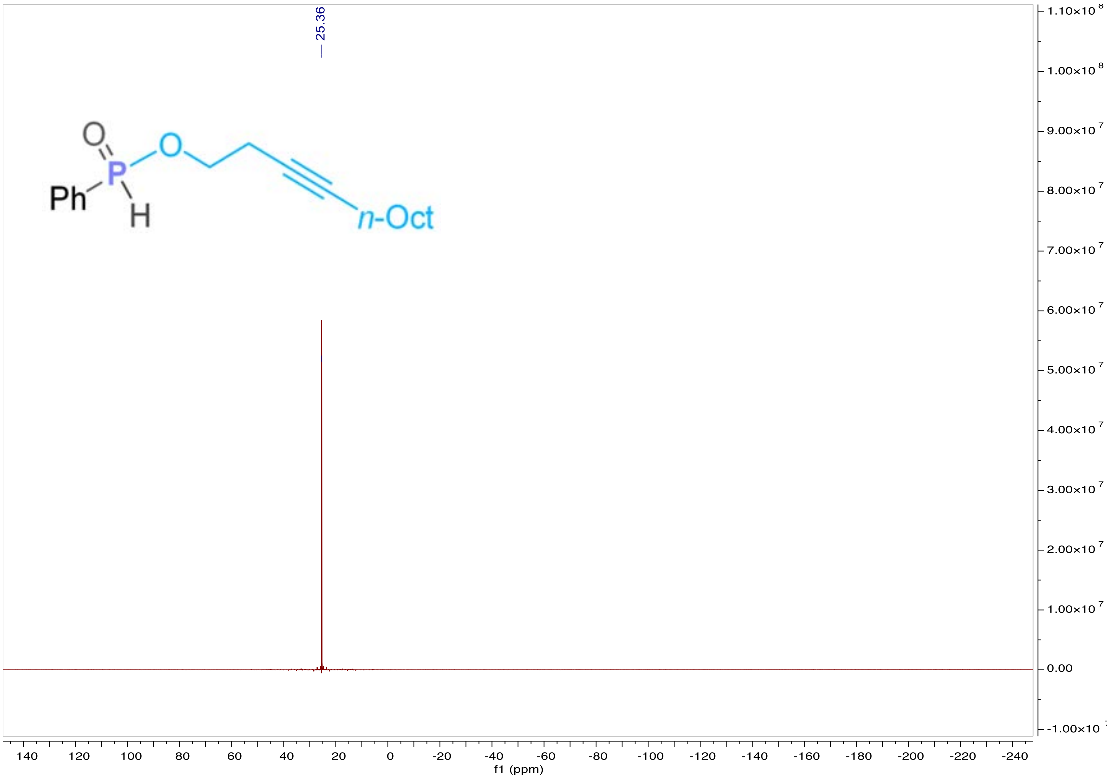

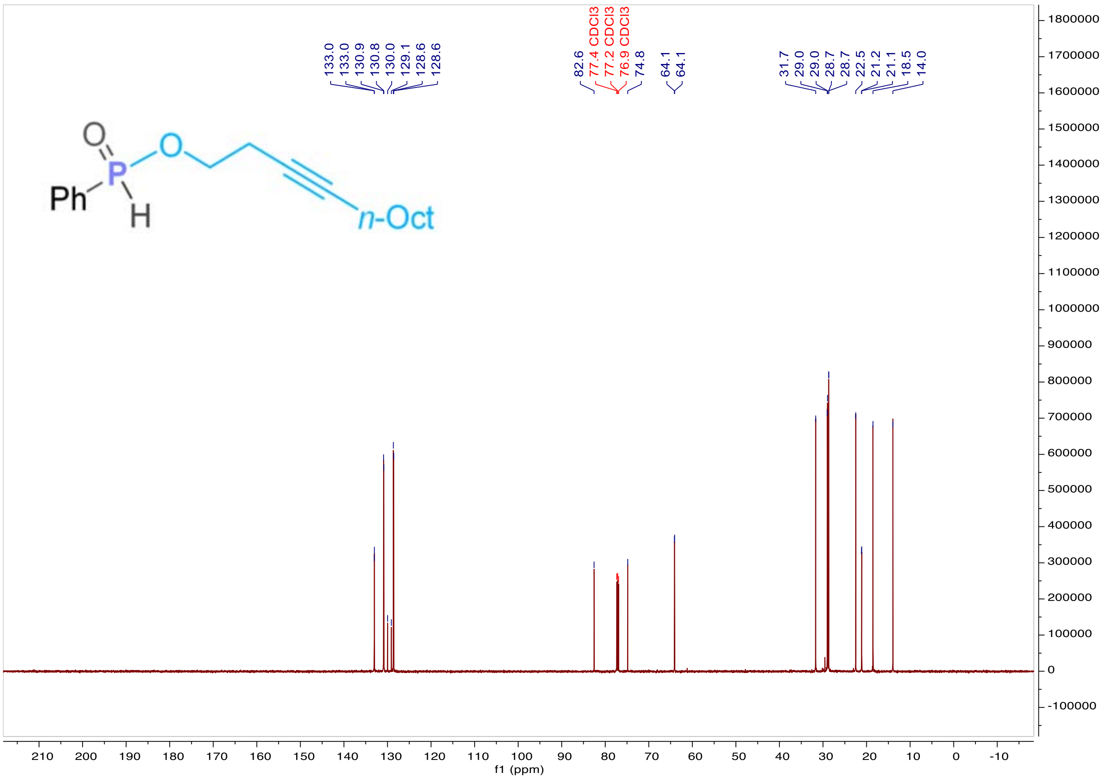

Figure S36. ^{1}H NMR, ^{31}P NMR and ^{13}C NMR spectra for **1s**

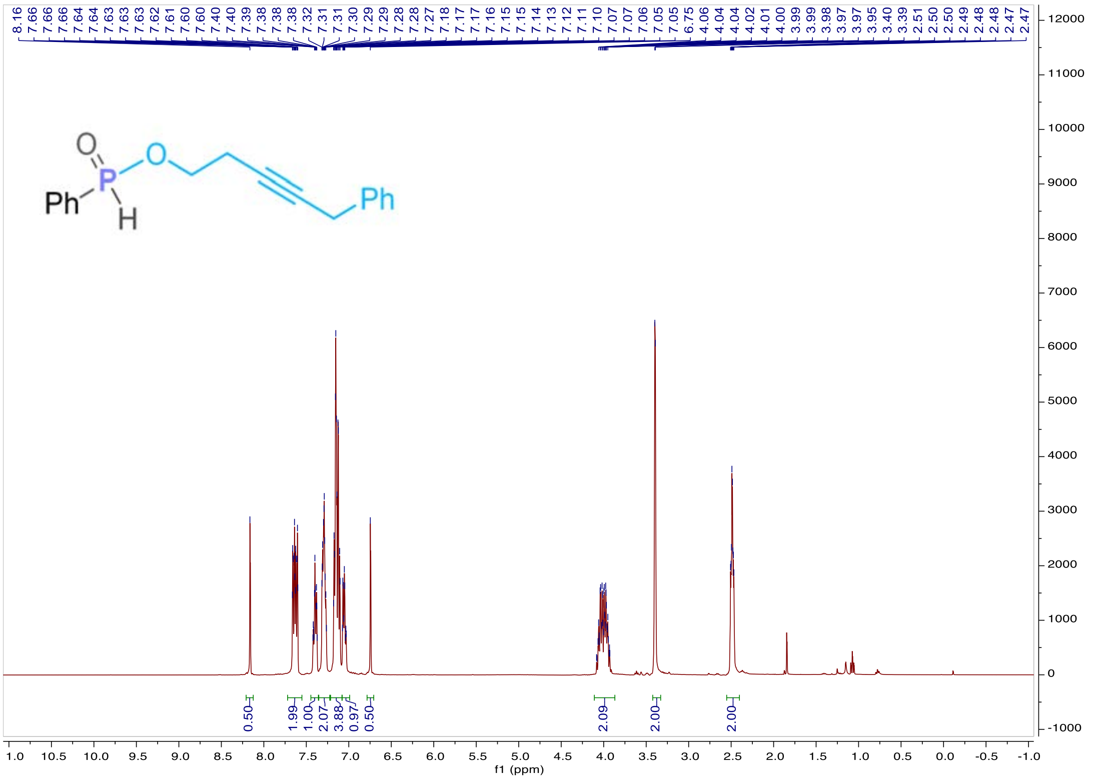

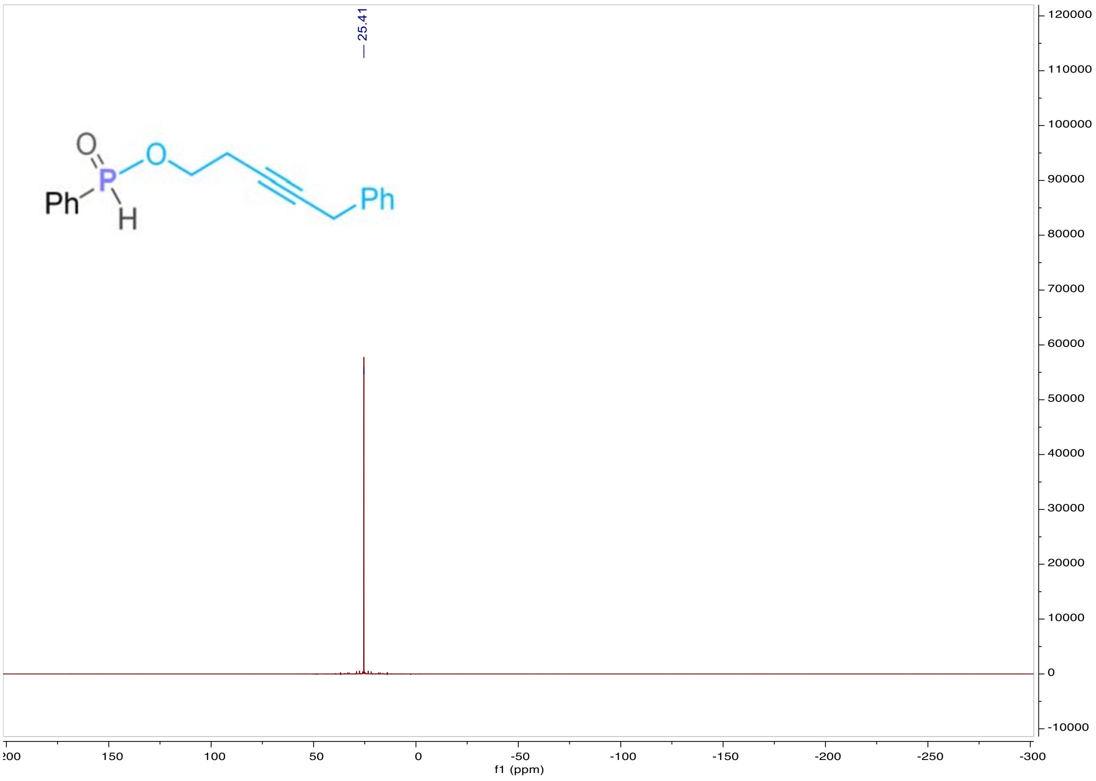

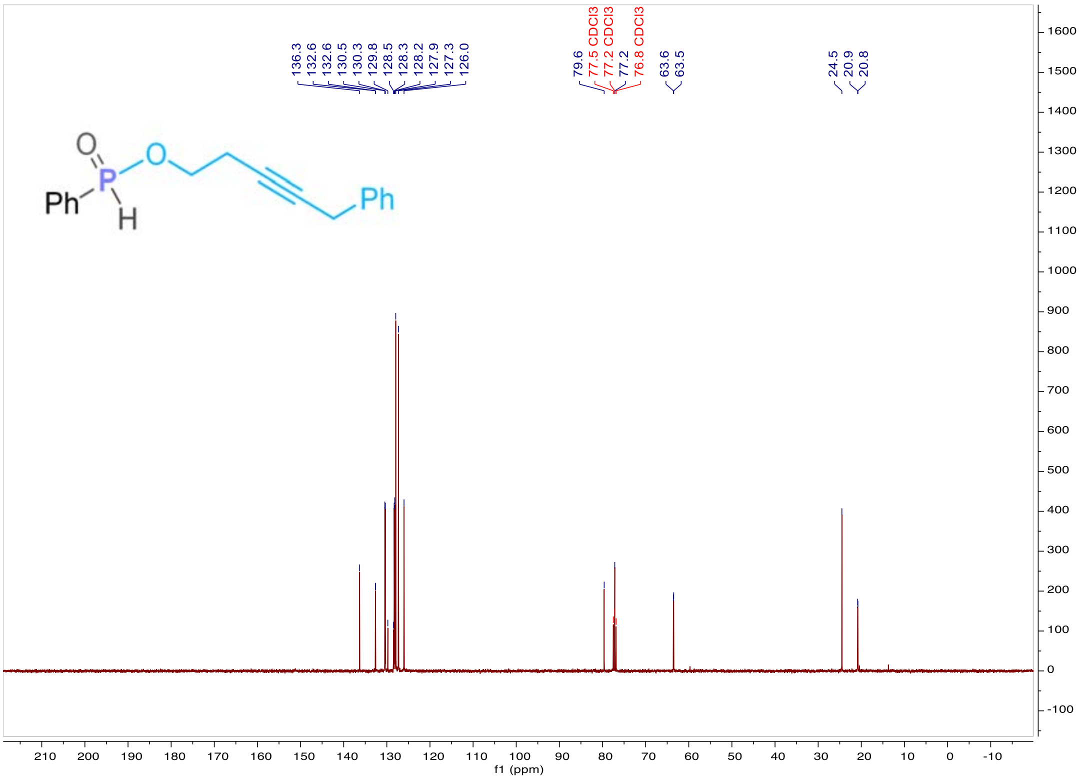

Figure S37. ^{1}H NMR, ^{31}P NMR and ^{13}C NMR spectra for **1t**

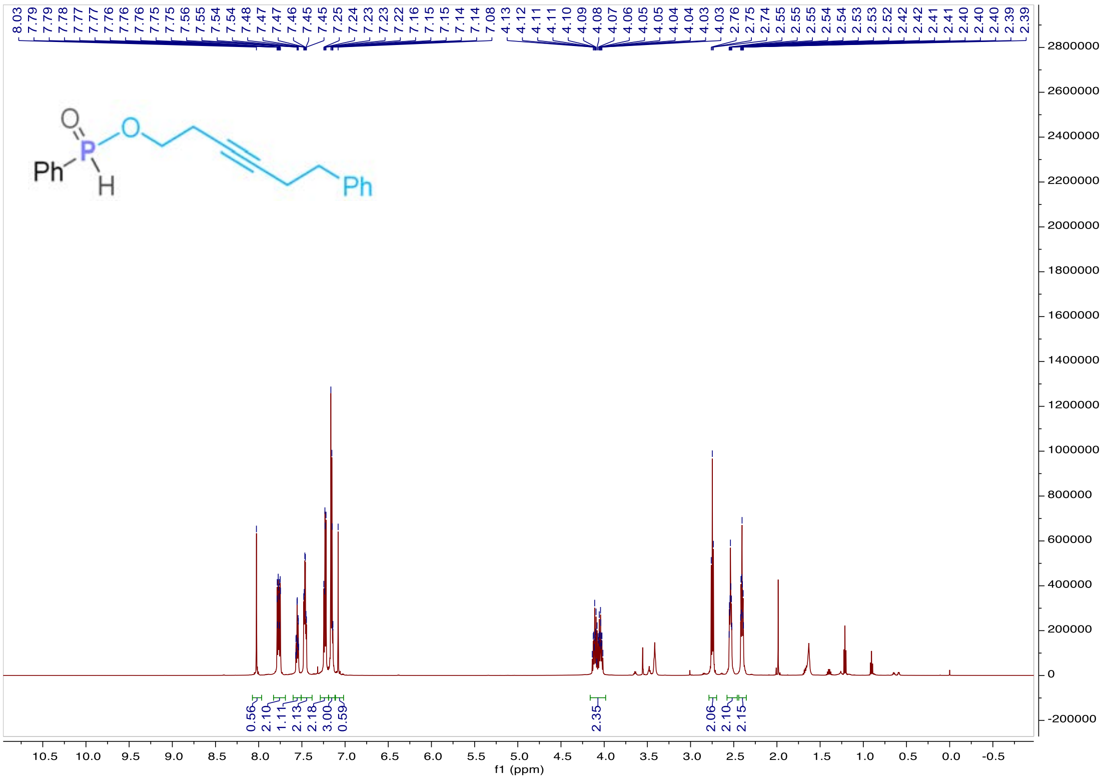

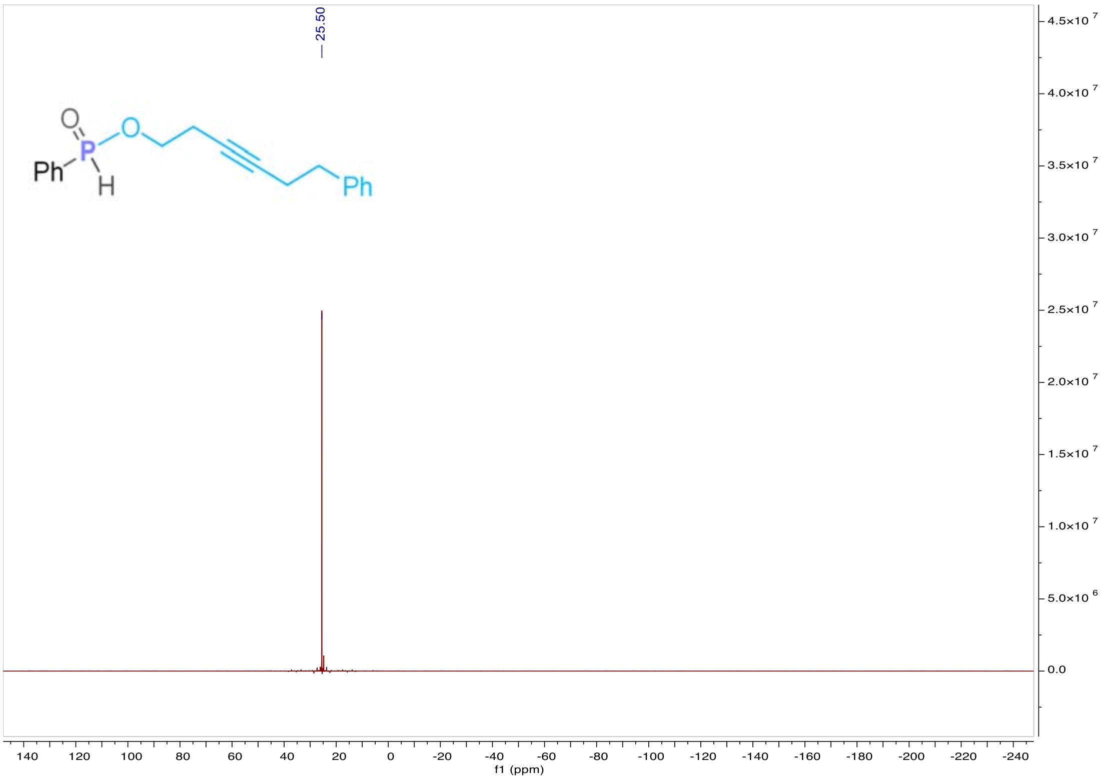

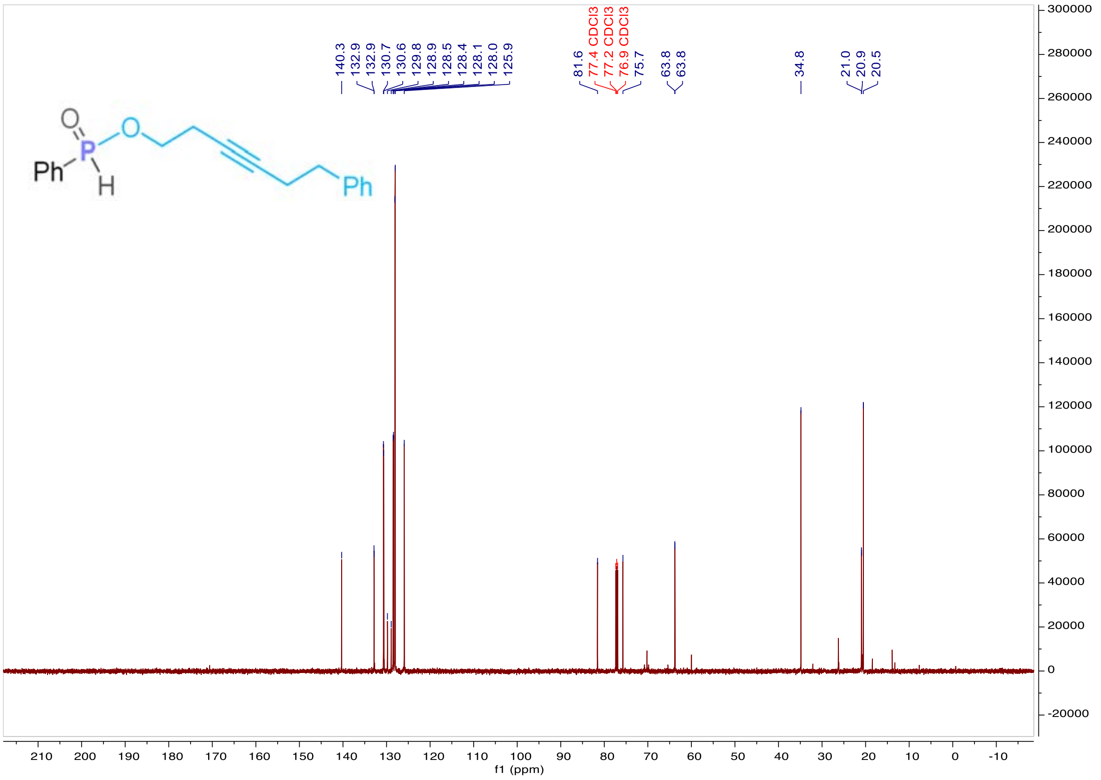

Figure S38. ^{1}H NMR, ^{31}P NMR and ^{13}C NMR spectra for **1u**

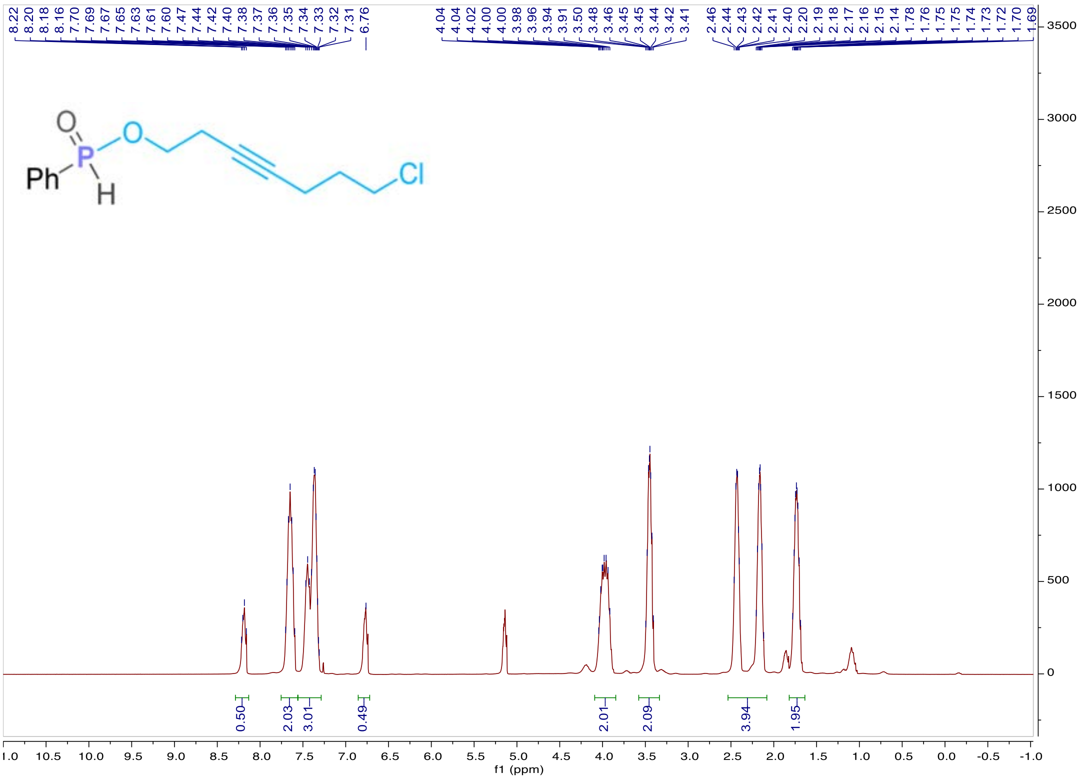
8.22
8.20
8.18
8.16
7.70
7.69
7.67
7.65
7.63
7.61
7.60
7.47
7.44
7.42
7.40
7.38
7.37
7.36
7.35
7.34
7.33
7.32
7.31
6.76
4.04
4.04
4.02
4.00
4.00
3.98
3.96
3.94
3.91
3.50
3.48
3.46
3.45
3.45
3.44
3.42
3.41
2.46
2.44
2.43
2.42
2.41
2.40
2.20
2.19
2.18
2.17
2.16
2.15
2.14
1.78
1.76
1.75
1.75
1.74
1.73
1.72
1.70
1.69
Ph
P
O
H
O
Cl
0.50
2.03
3.01
0.49
2.01
2.09
3.94
1.95
f1 (ppm)

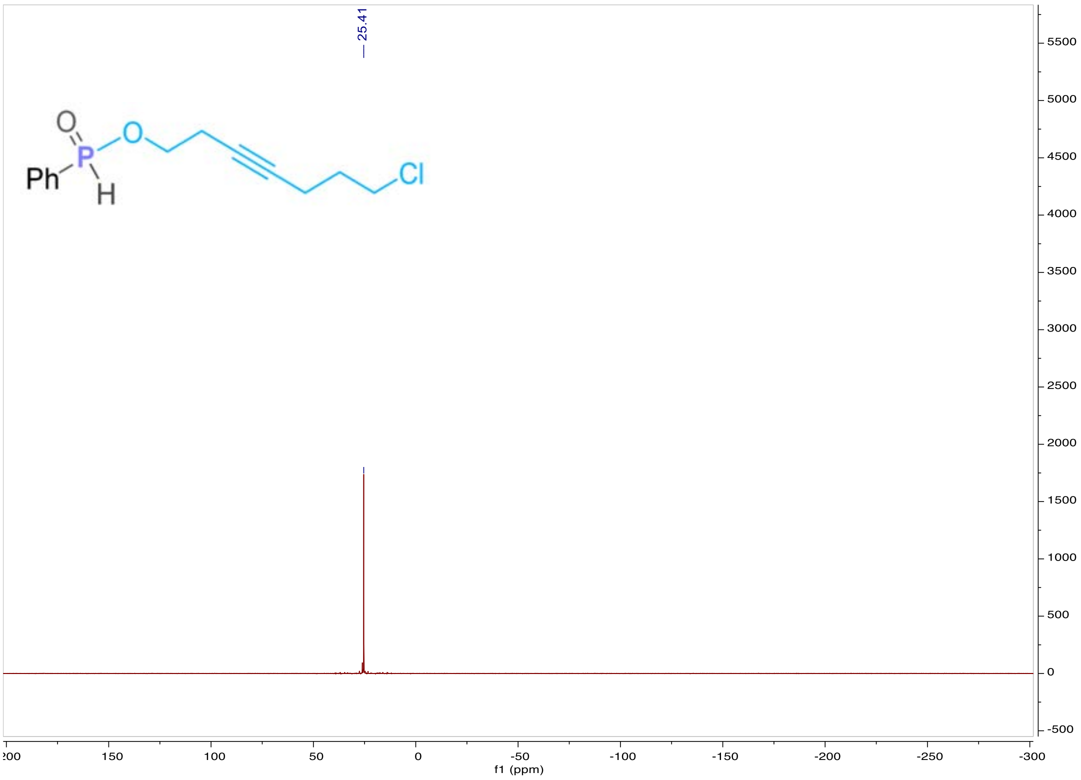
25.41
Ph
P
O
H
O
Cl
f1 (ppm)

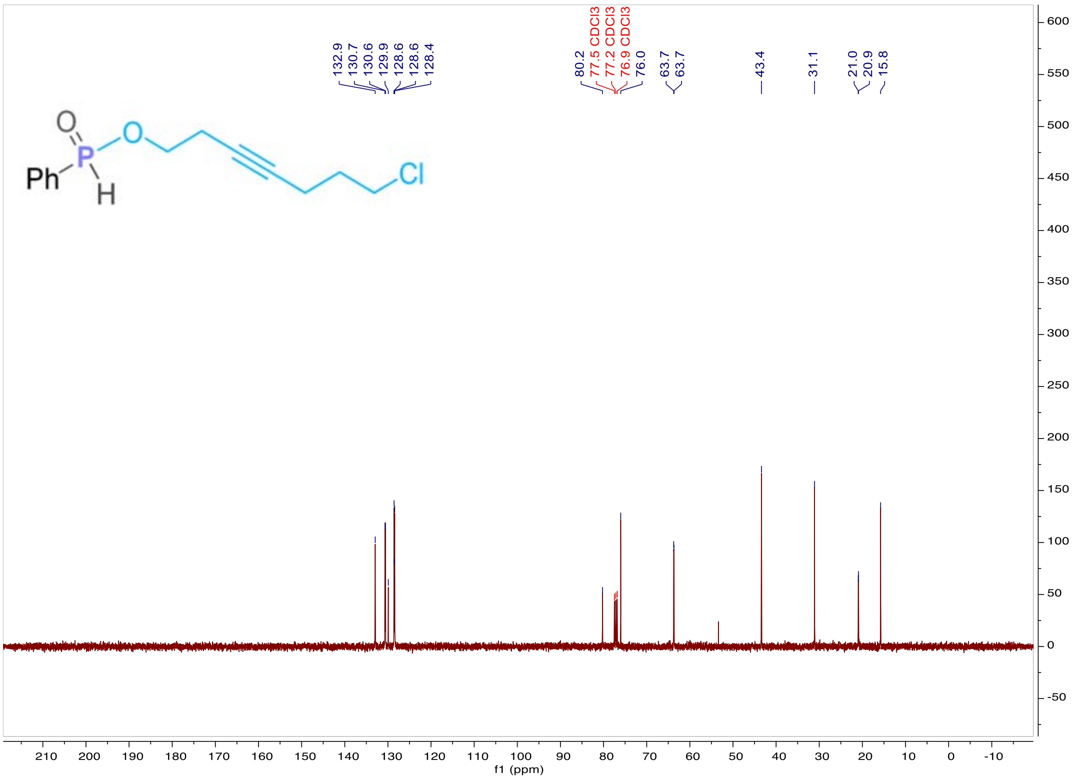

Figure S39. ^{1}H NMR, ^{31}P NMR and ^{13}C NMR spectra for **1v**

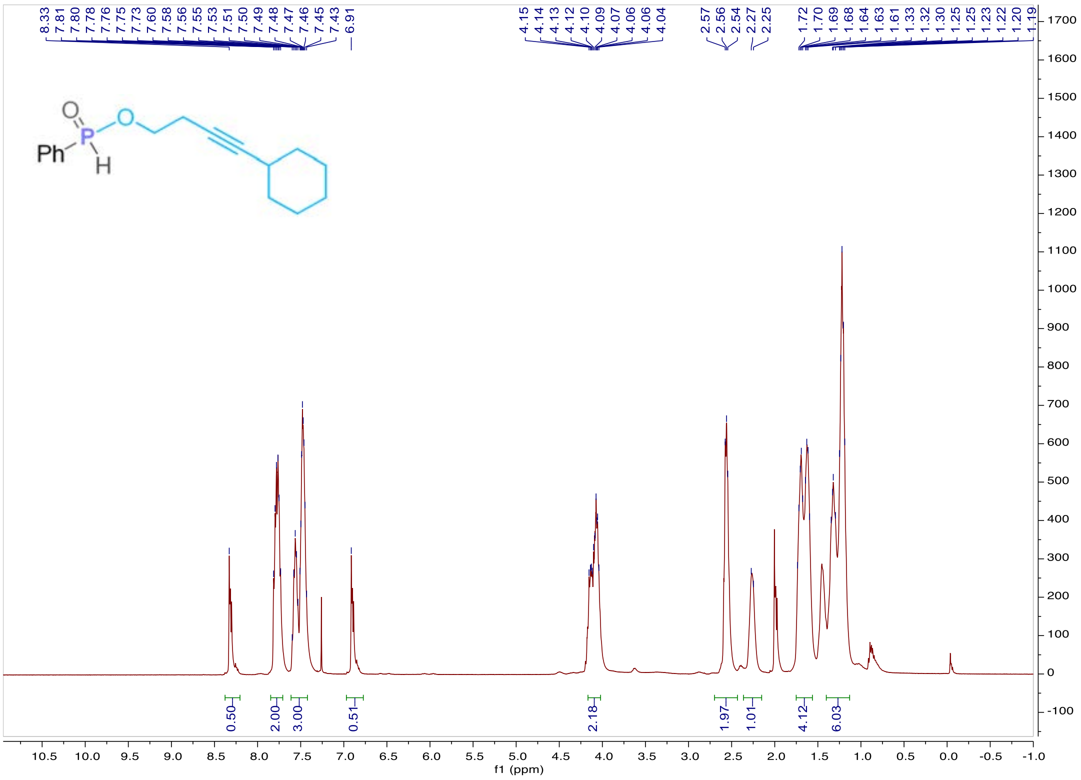
8.33
7.81
7.80
7.78
7.76
7.75
7.73
7.60
7.58
7.56
7.55
7.53
7.51
7.50
7.49
7.48
7.47
7.46
7.45
7.43
6.91
4.15
4.14
4.13
4.12
4.10
4.09
4.07
4.06
4.06
4.04
2.57
2.56
2.54
2.27
2.25
1.72
1.70
1.69
1.68
1.64
1.63
1.61
1.33
1.32
1.30
1.25
1.23
1.22
1.20
1.19
Ph
P
O
H
0.50
2.00
3.00
0.51
2.18
1.97
1.01
4.12
6.03
f1 (ppm)

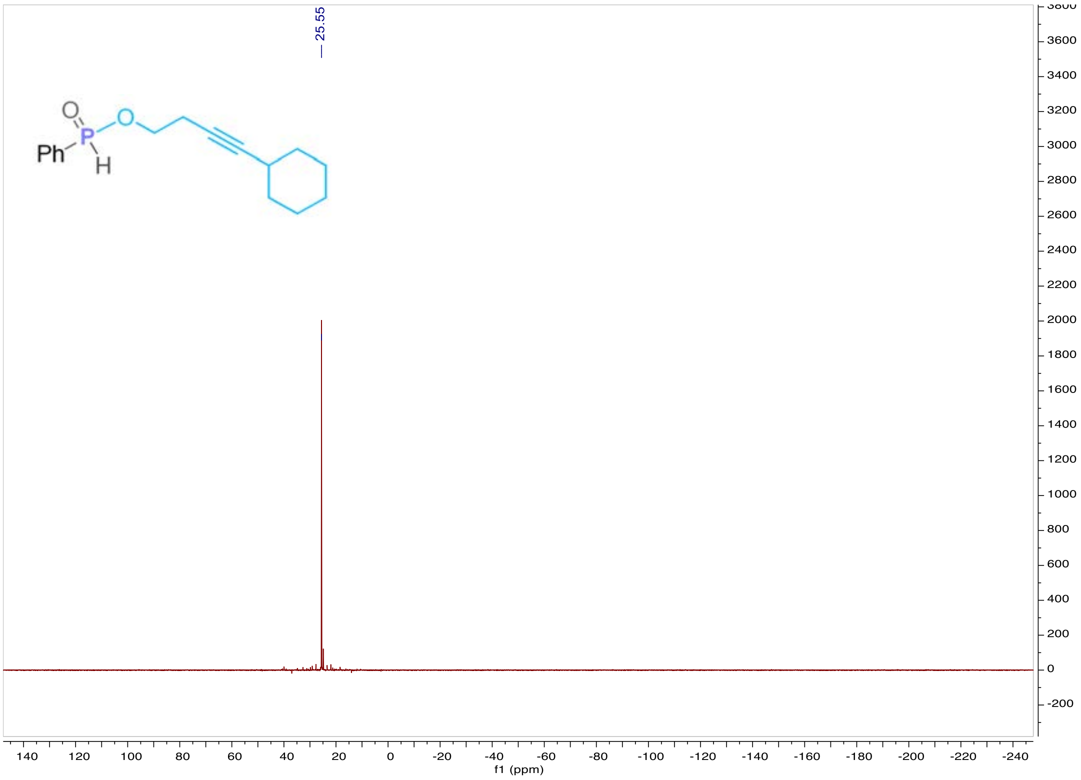
25.55
Ph
P
O
H
f1 (ppm)

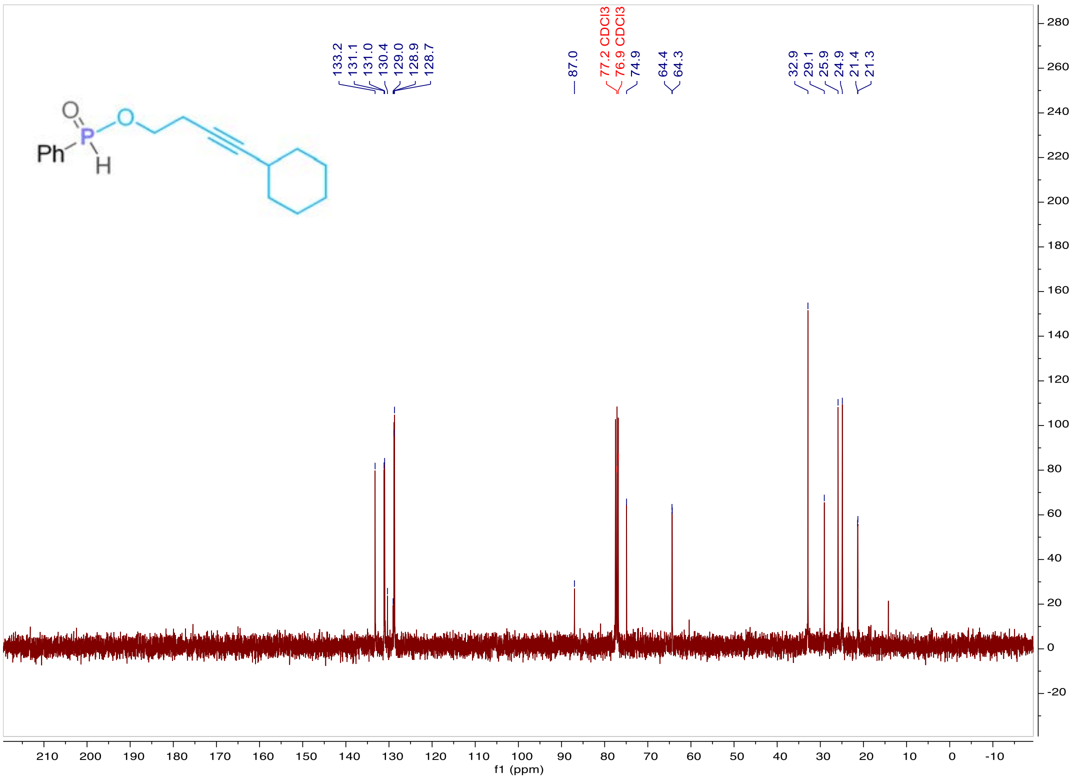

Figure S40. ^{1}H NMR, ^{31}P NMR and ^{13}C NMR spectra for **1w**

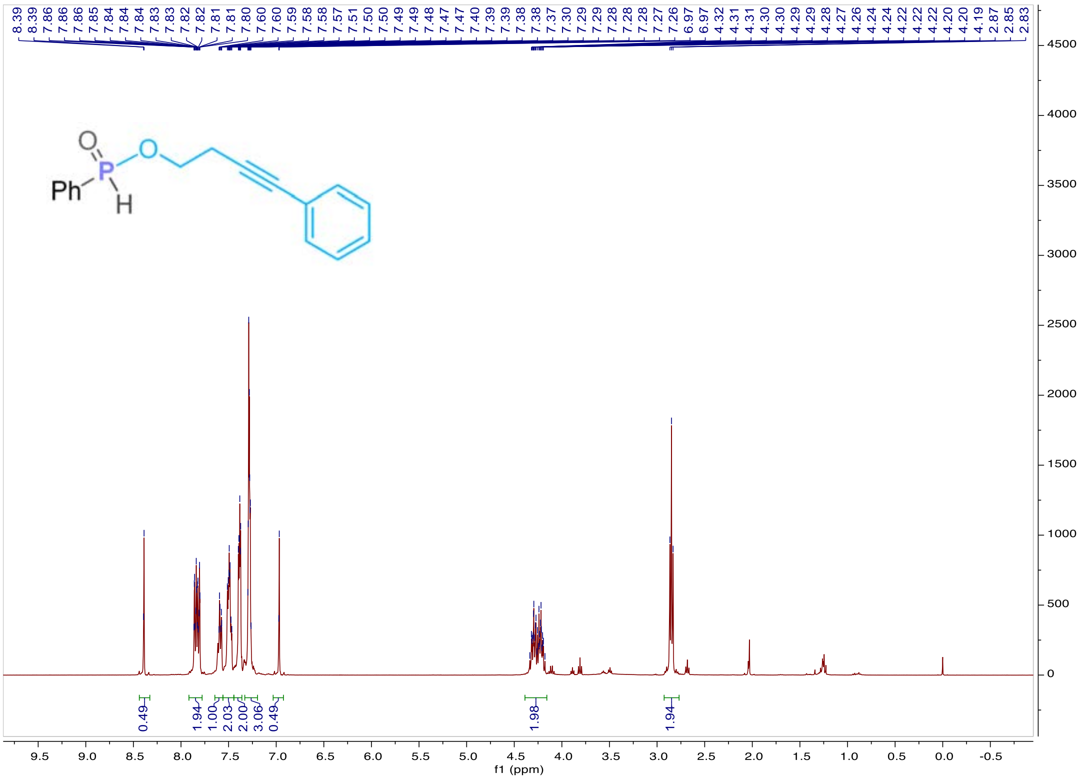
Ph
H
O
P
O
f1 (ppm)
0.49
1.94
1.00
2.03
2.00
3.06
0.49
1.98
1.94

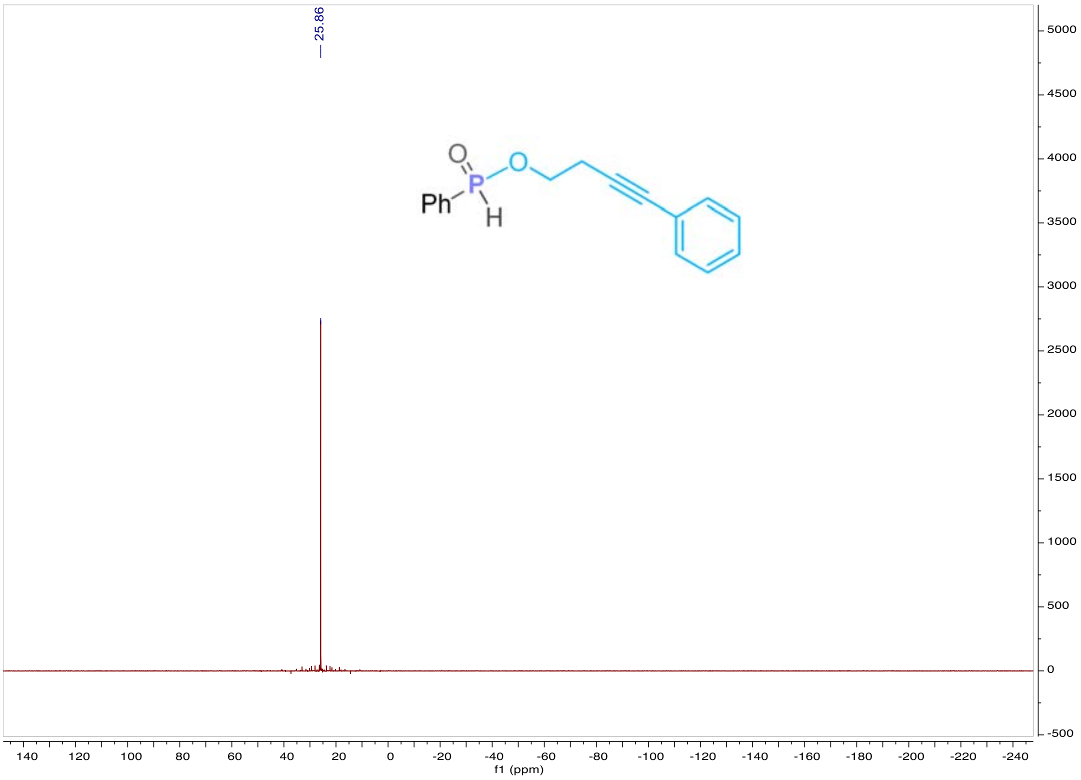
25.86
Ph
H
O
P
O
f1 (ppm)

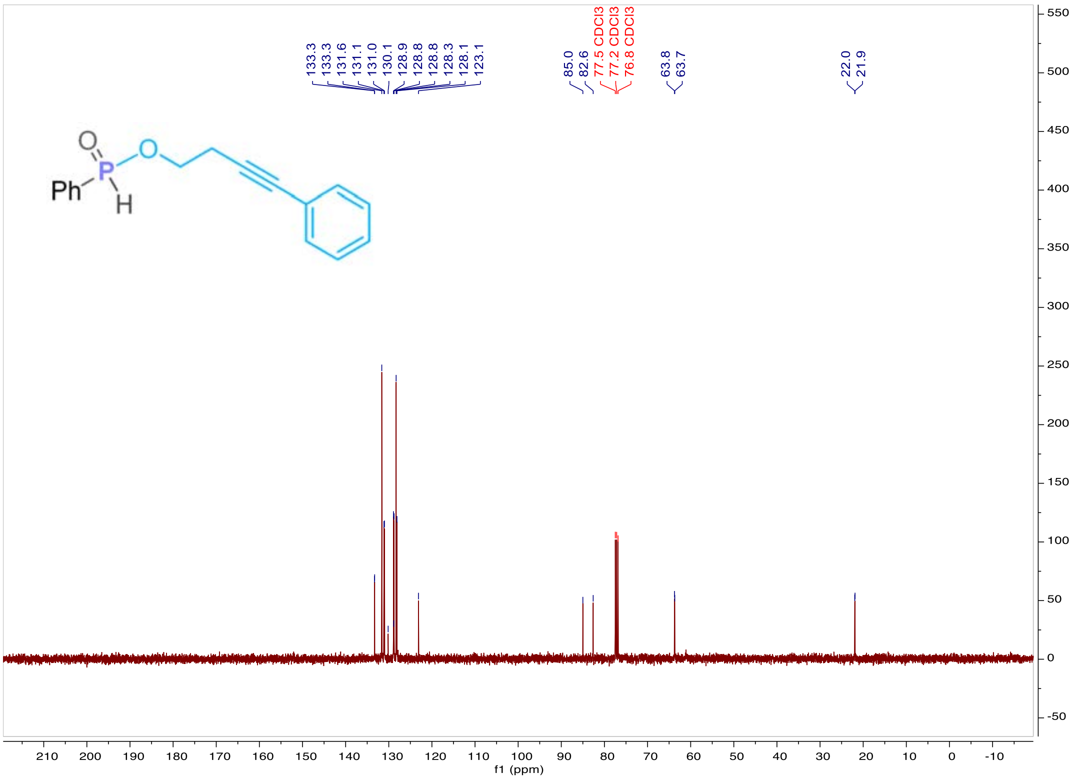

Figure S41. ^{1}H NMR, ^{31}P NMR and ^{13}C NMR spectra for **1x**

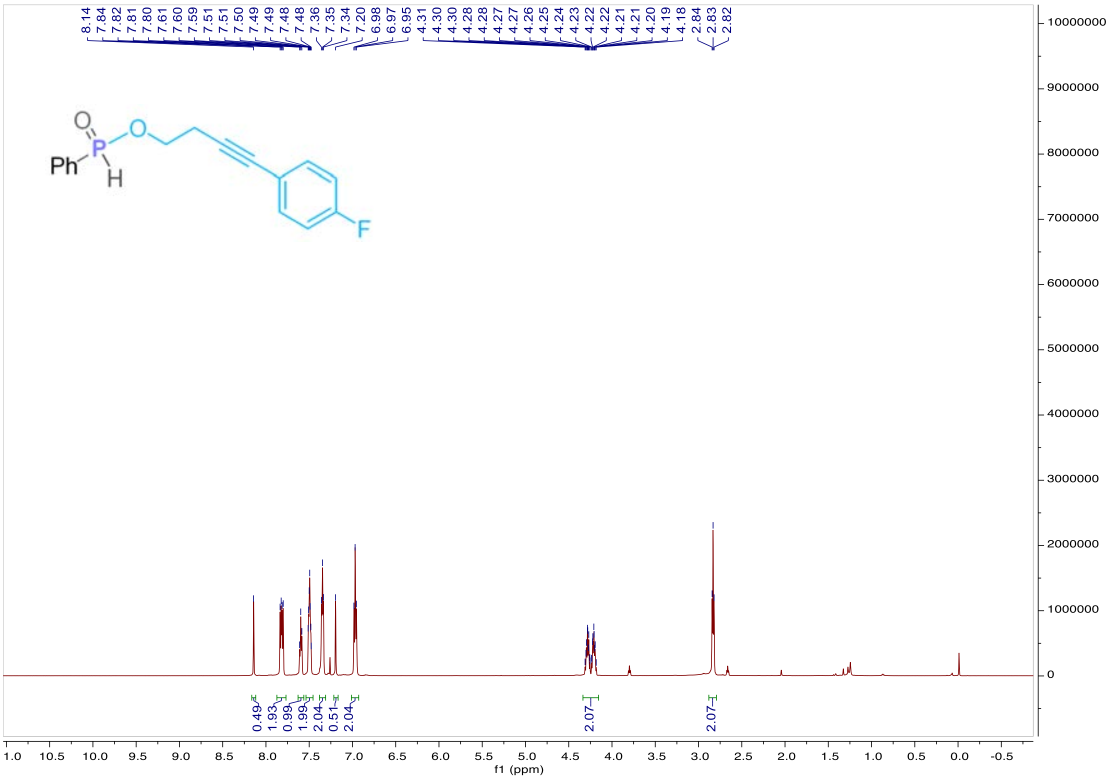
8.14
7.84
7.82
7.81
7.80
7.61
7.60
7.59
7.51
7.50
7.49
7.49
7.48
7.36
7.35
7.34
7.20
6.98
6.97
6.95
4.31
4.30
4.28
4.28
4.27
4.27
4.26
4.25
4.24
4.23
4.22
4.22
4.21
4.21
4.20
4.19
4.18
2.84
2.83
2.82
O
P
O
Ph
H
F
0.49
1.93
0.99
1.99
2.04
0.51
2.04
2.07
2.07
f1 (ppm)

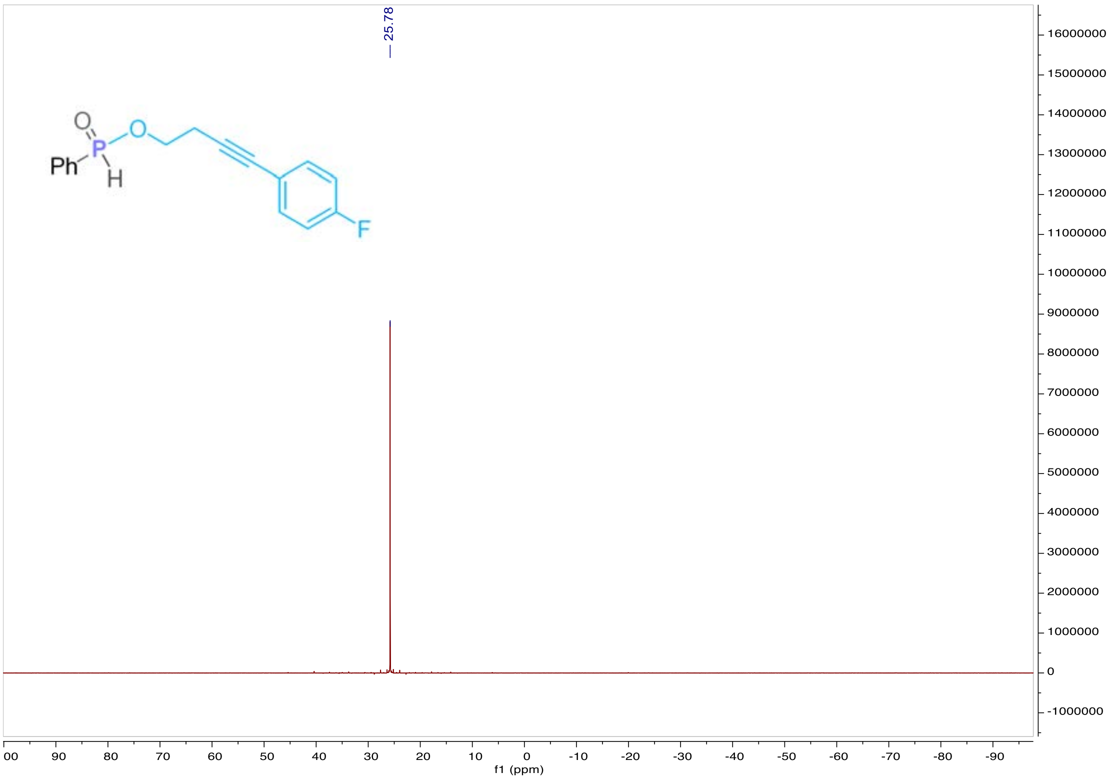
25.78
O
P
O
Ph
H
F
f1 (ppm)

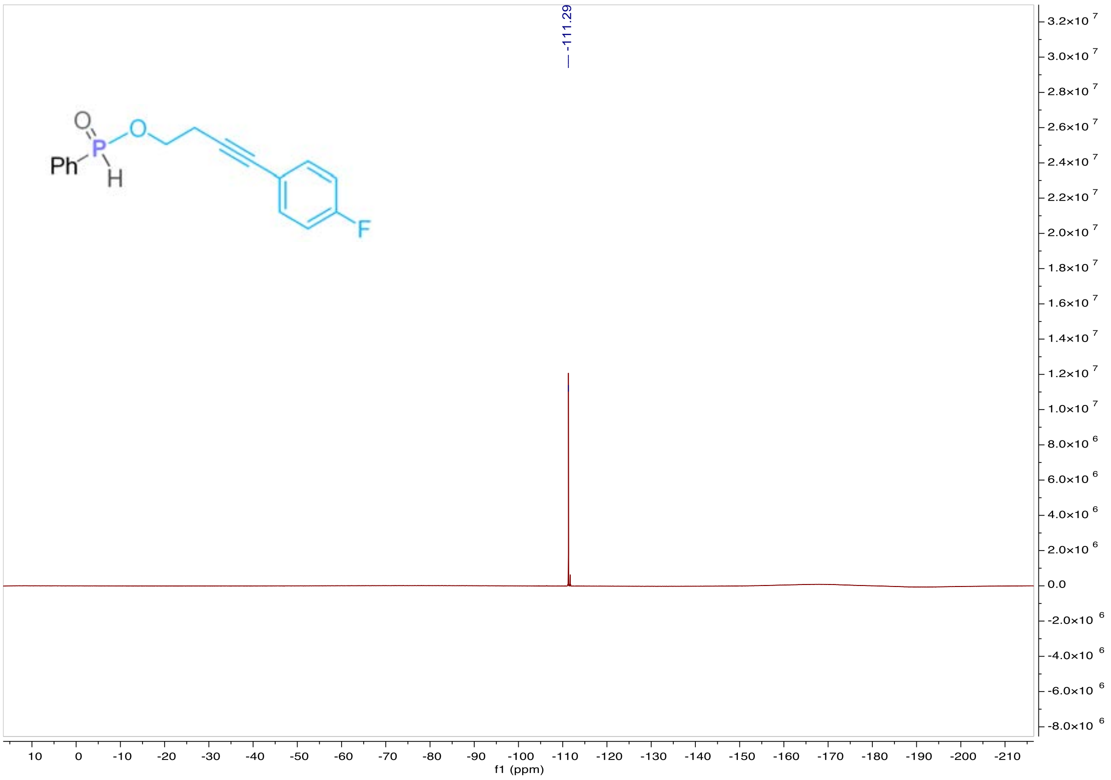

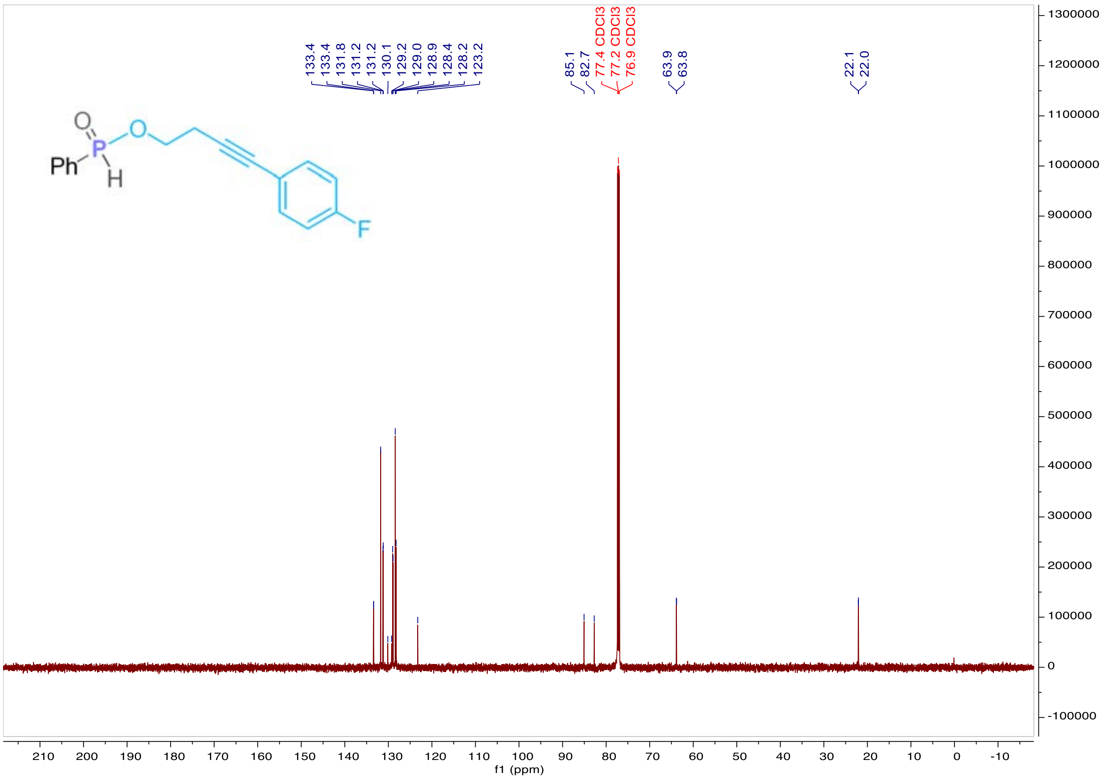

Figure S42. ^{1}H NMR, ^{19}F NMR, ^{31}P NMR and ^{13}C NMR spectra for **1y**

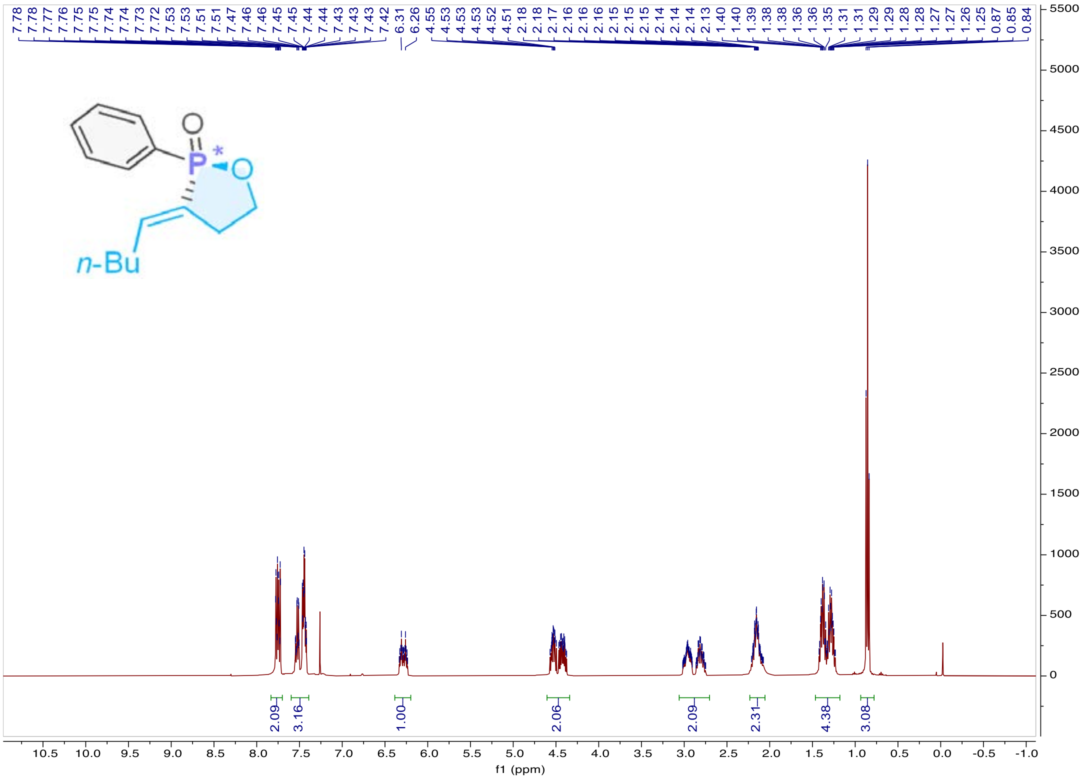

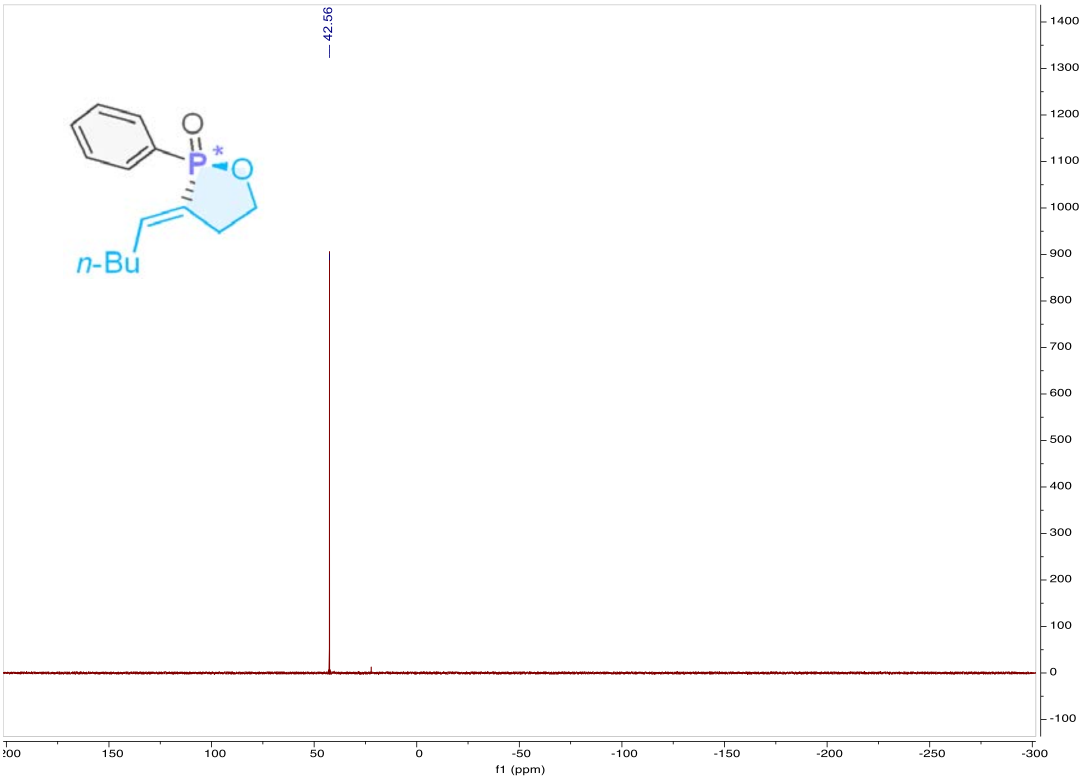

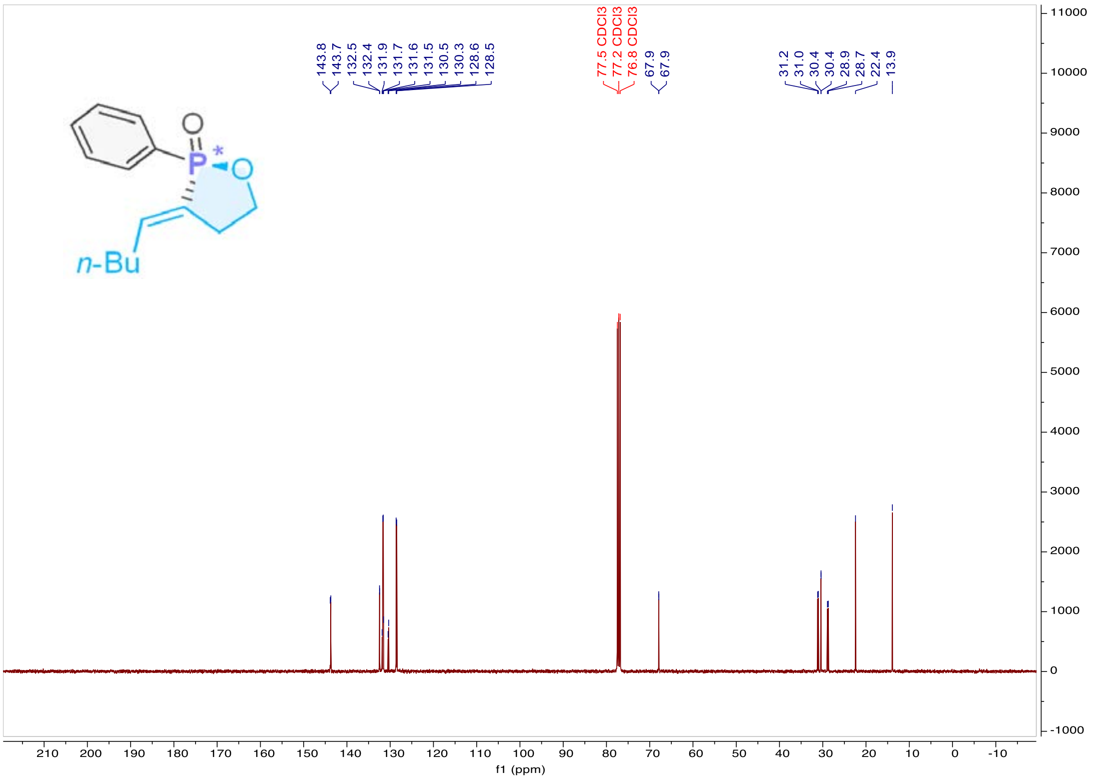

Figure S43. ^{1}H NMR, ^{31}P NMR and ^{13}C NMR spectra for **2a**

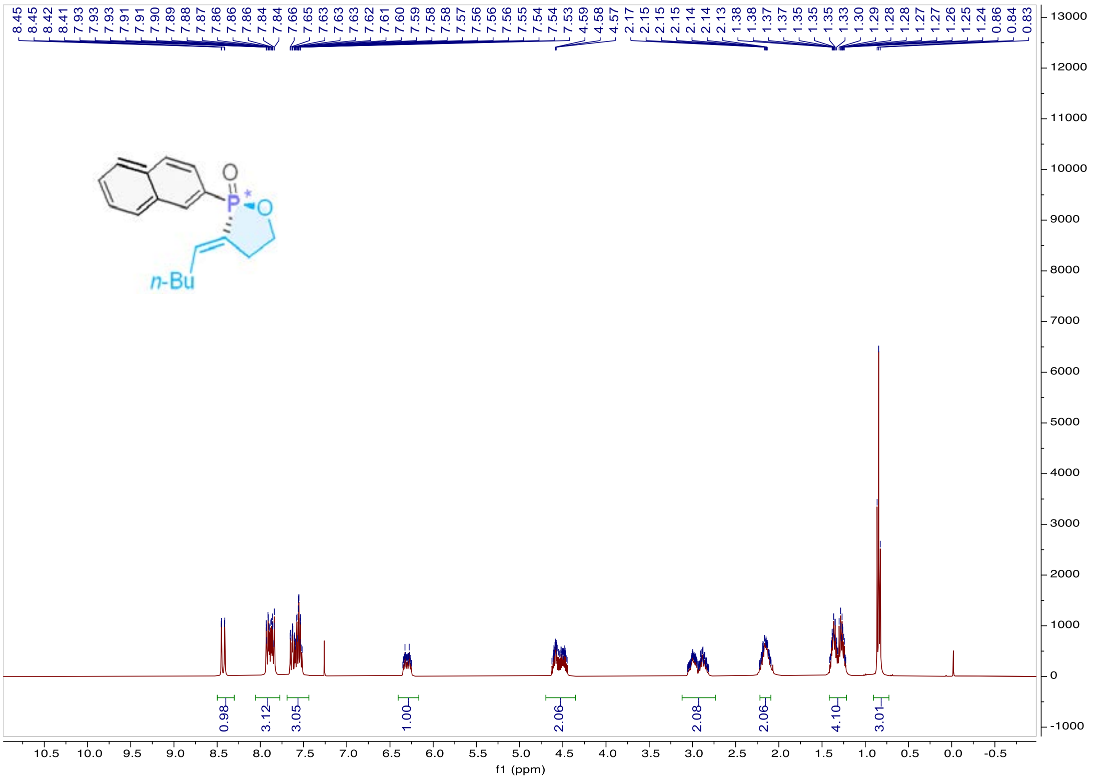

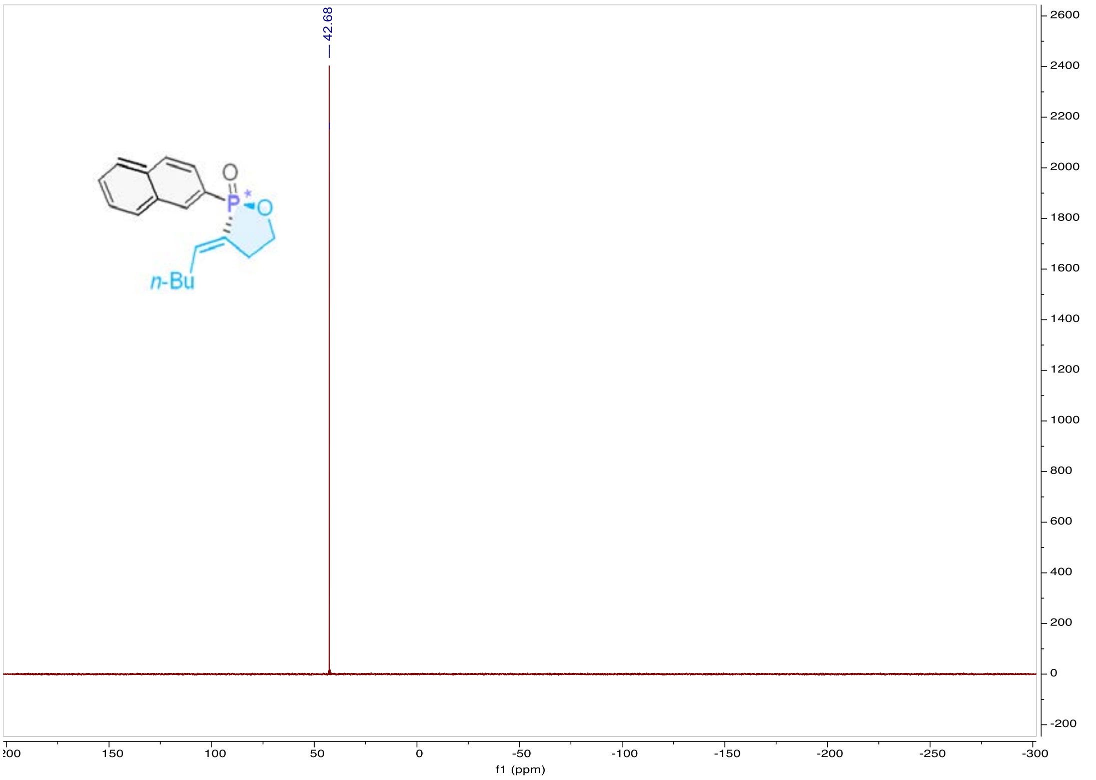

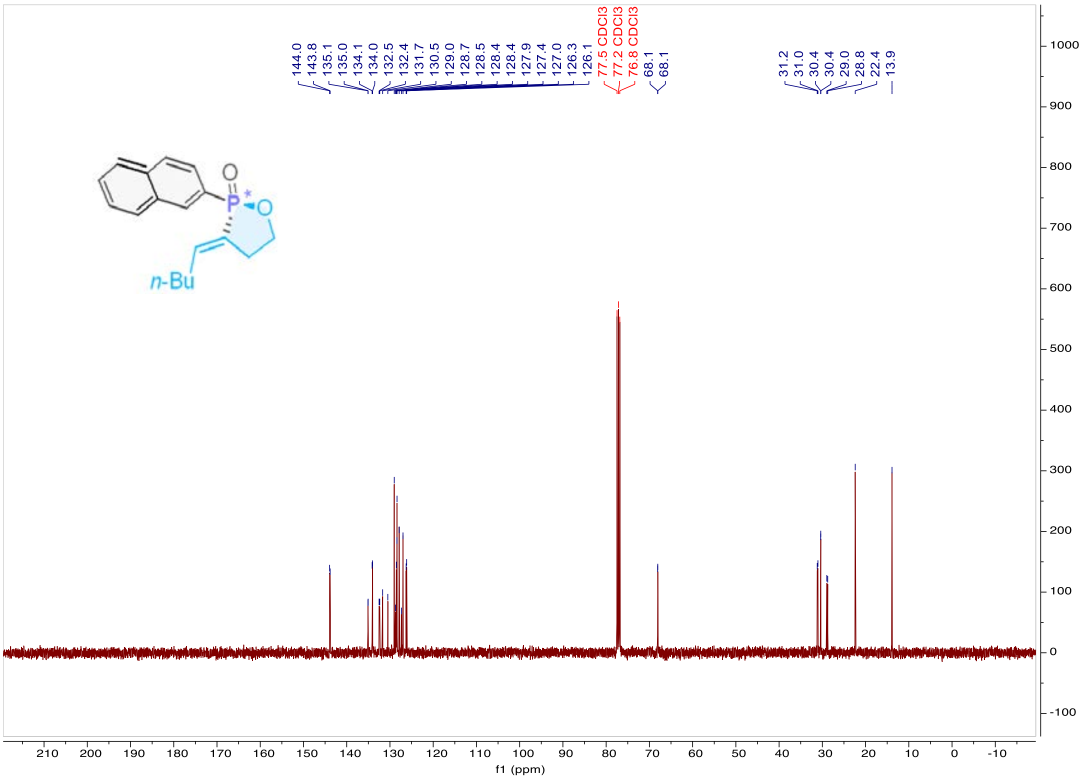

Figure S44. ^{1}H NMR, ^{31}P NMR and ^{13}C NMR spectra for **2b**

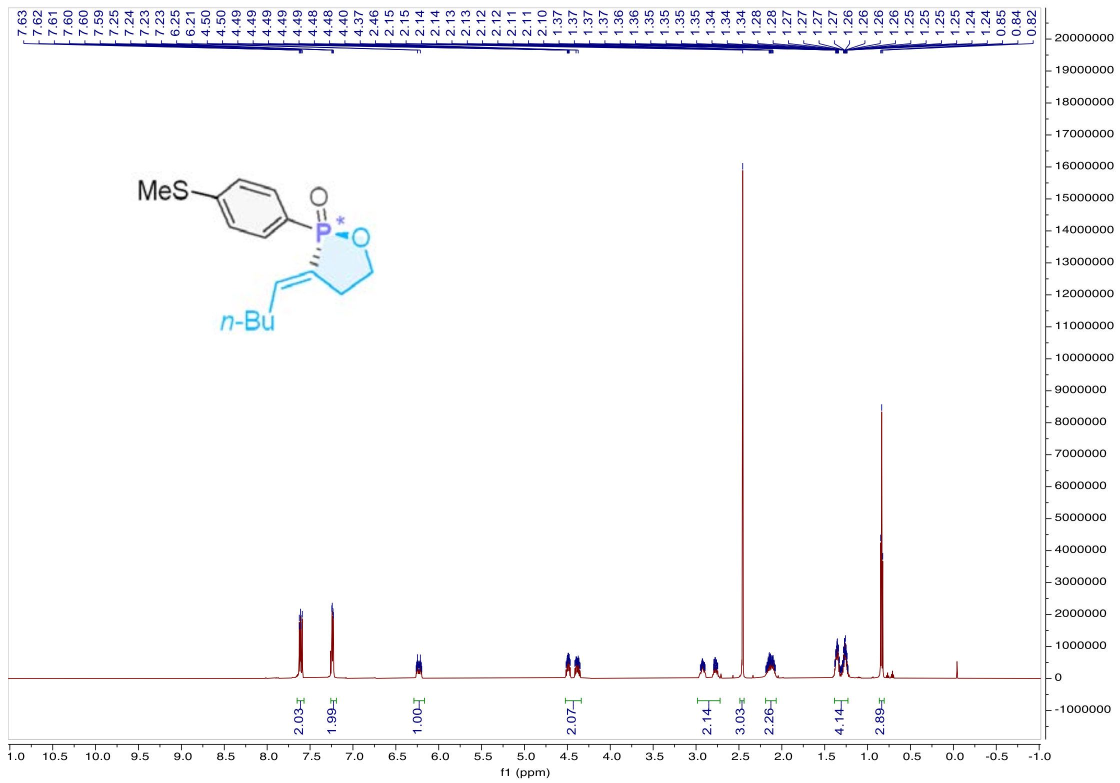

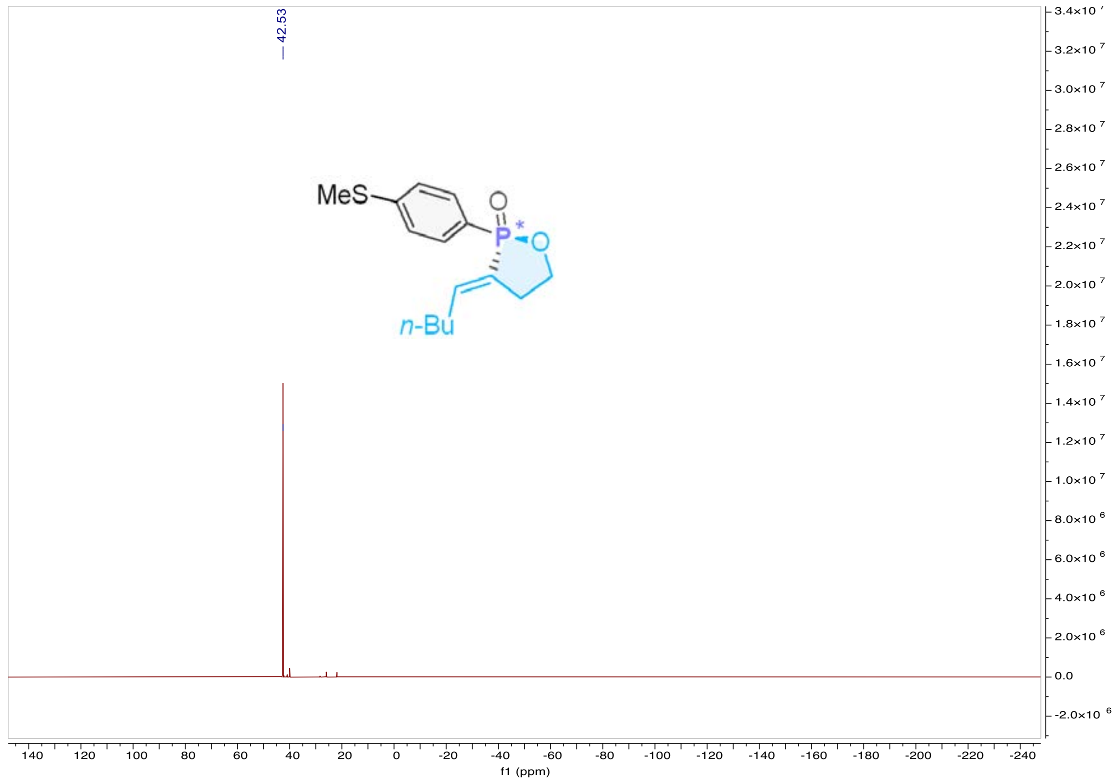

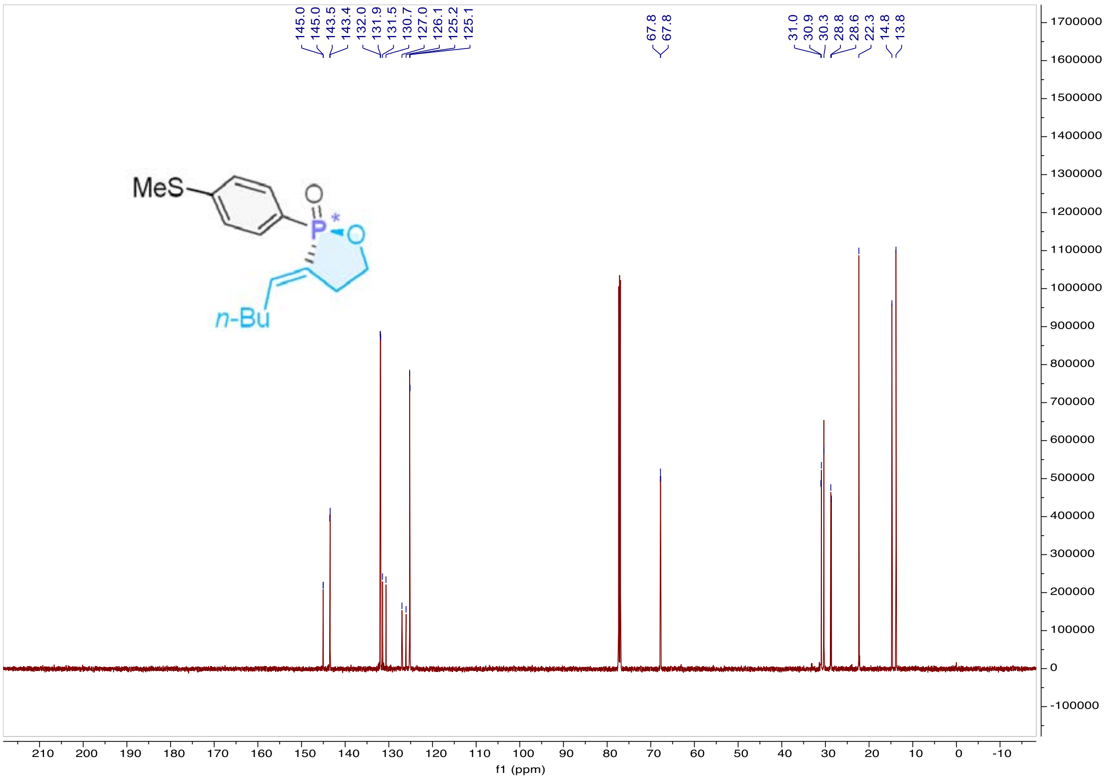

Figure S45. ^{1}H NMR, ^{31}P NMR and ^{13}C NMR spectra for **2c**

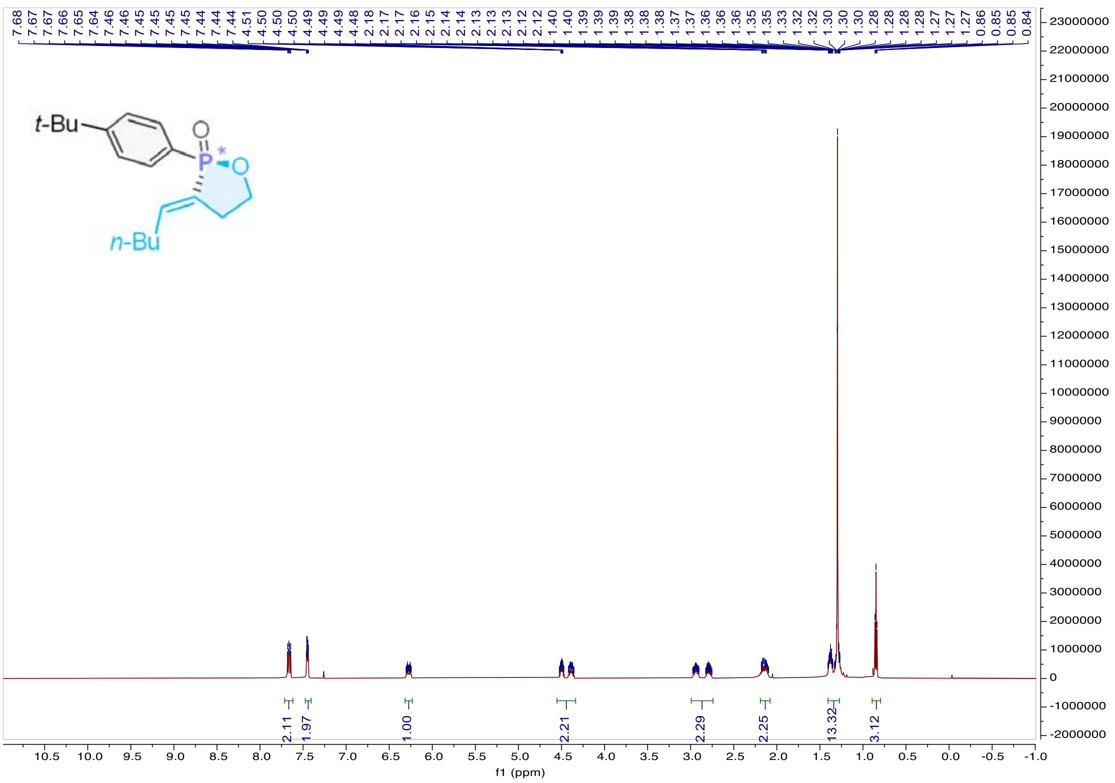

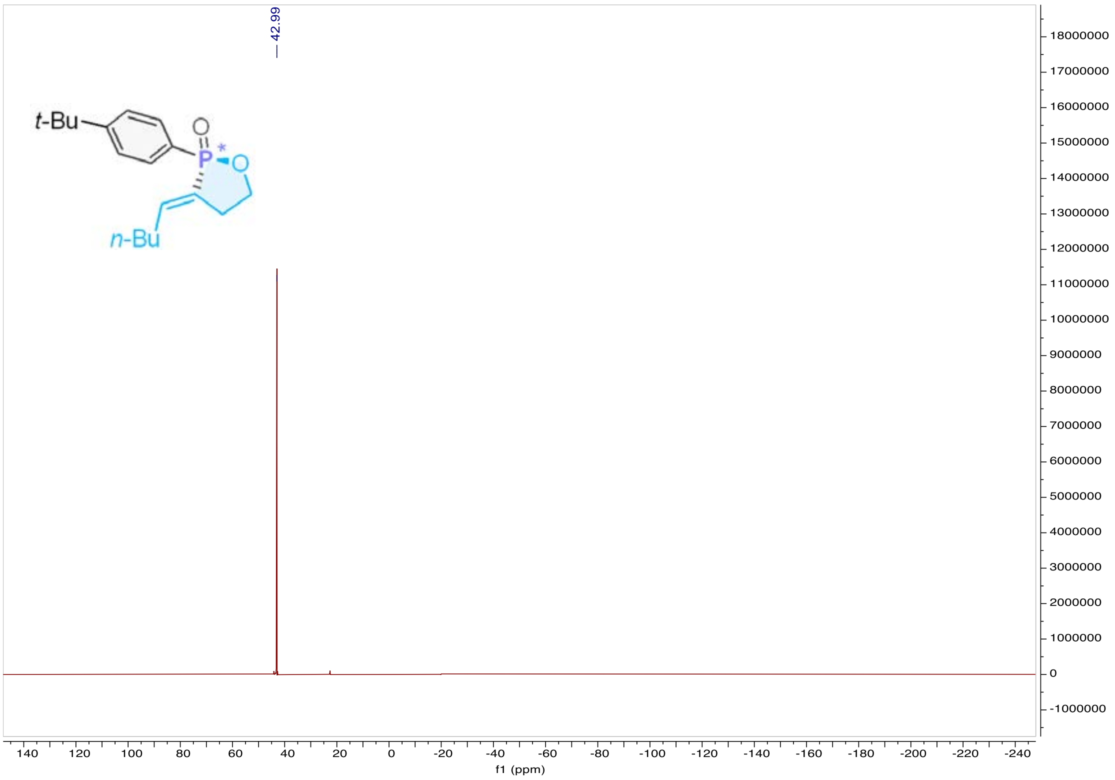

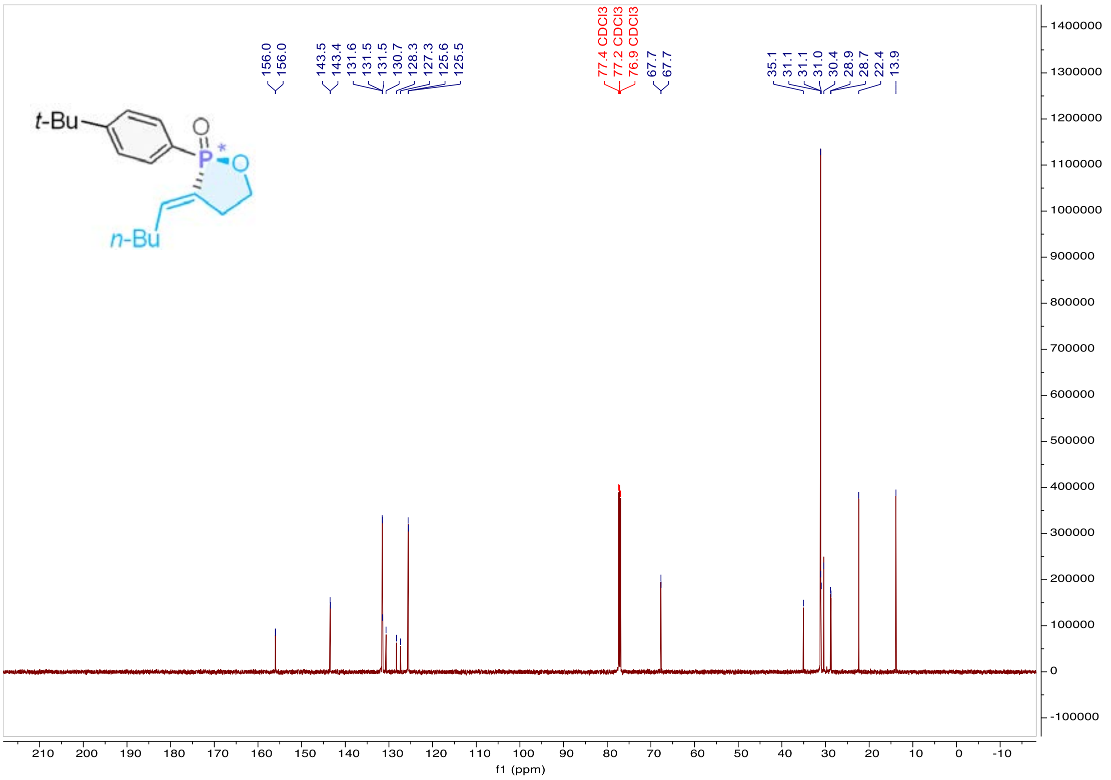

Figure S46. ^{1}H NMR, ^{31}P NMR and ^{13}C NMR spectra for **2d**

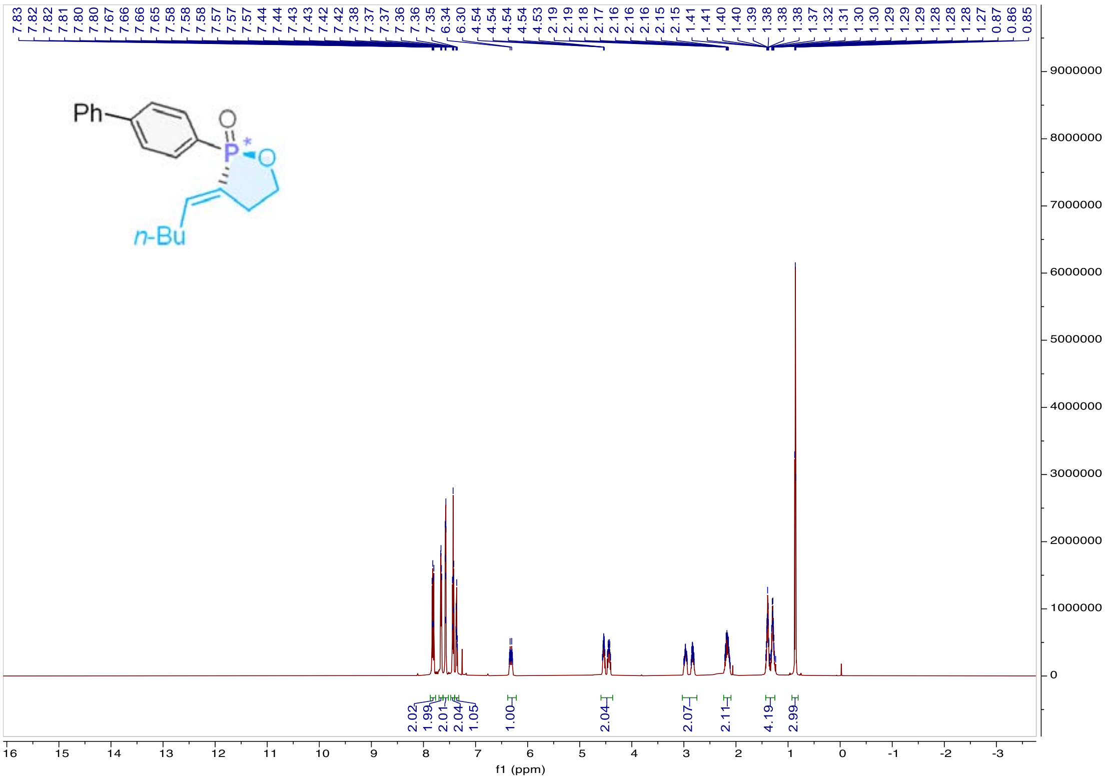

7.83 7.82 7.82 7.81 7.80 7.80 7.67 7.66 7.66 7.65 7.58 7.58 7.58 7.57 7.57 7.57 7.44 7.44 7.43 7.43 7.42 7.42 7.38 7.37 7.37 7.36 7.36 7.35 6.34 6.30 4.54 4.54 4.54 4.54 4.53 2.19 2.19 2.18 2.17 2.16 2.16 2.15 2.15 1.41 1.41 1.40 1.40 1.39 1.38 1.38 1.38 1.38 1.37 1.32 1.31 1.30 1.30 1.29 1.29 1.29 1.28 1.28 1.28 1.27 0.87 0.86 0.85
Ph
O
P
*
O
n-Bu
2.02 1.99 2.01 2.04 1.05 1.00 2.04 2.07 2.11 4.19 2.99
f1 (ppm)

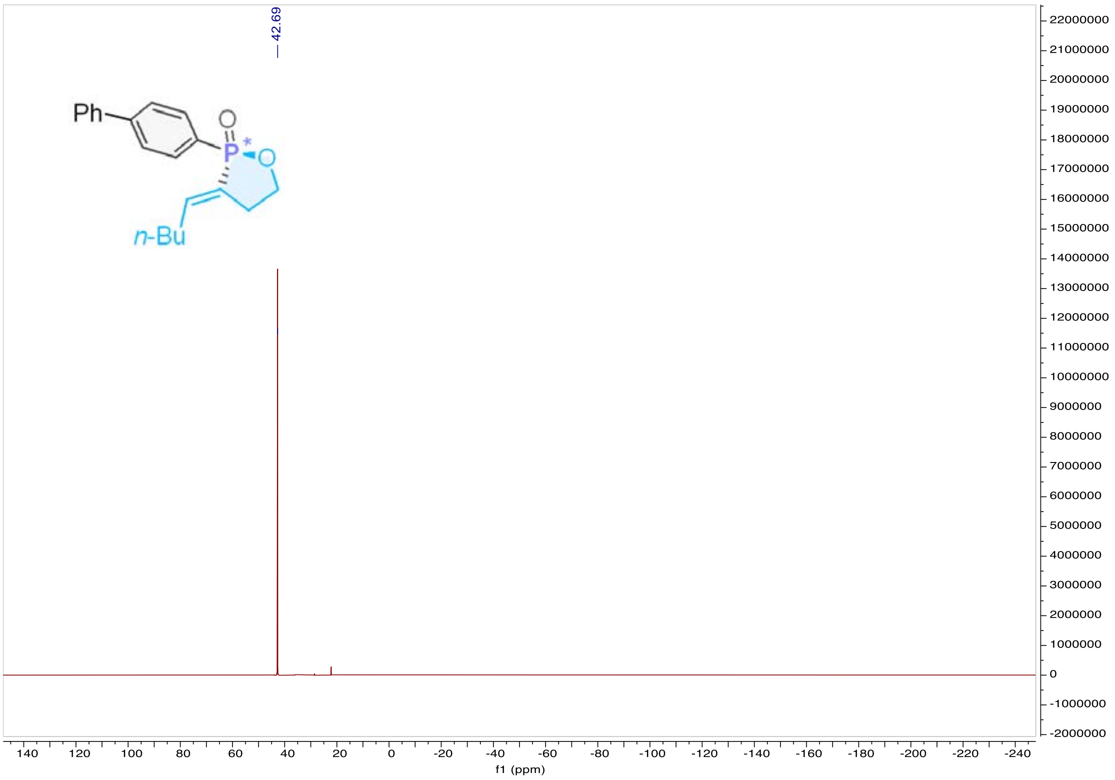

42.69
Ph
O
P
*
O
n-Bu
f1 (ppm)

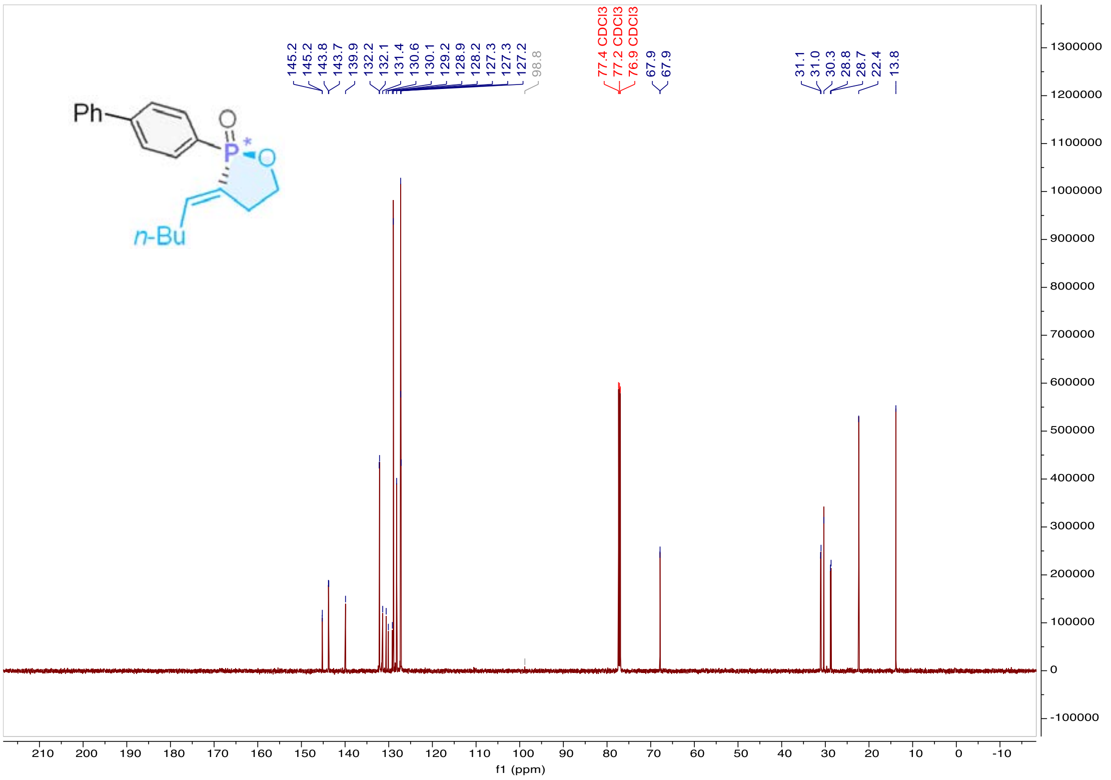

Figure S47. ^{1}H NMR, ^{31}P NMR and ^{13}C NMR spectra for **2e**

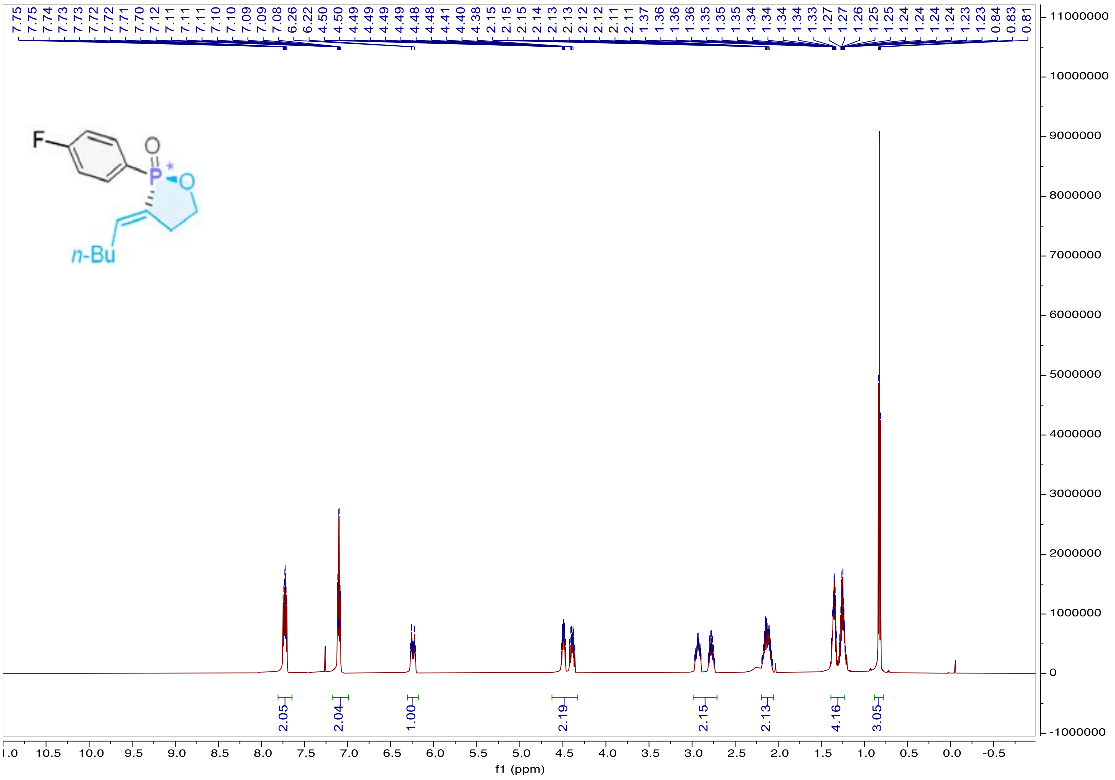
7.75
7.75
7.74
7.73
7.73
7.72
7.72
7.71
7.70
7.12
7.11
7.11
7.11
7.10
7.10
7.09
7.09
7.08
6.26
6.22
4.50
4.50
4.49
4.49
4.49
4.49
4.48
4.48
4.41
4.40
4.38
2.15
2.15
2.14
2.13
2.13
2.12
2.12
2.11
2.11
1.37
1.36
1.36
1.35
1.35
1.35
1.34
1.34
1.34
1.33
1.27
1.27
1.26
1.25
1.25
1.24
1.24
1.24
1.24
1.23
1.23
0.84
0.83
0.81
F
O
P
*
O
n-Bu
2.05
2.04
1.00
2.19
2.15
2.13
4.16
3.05
f1 (ppm)

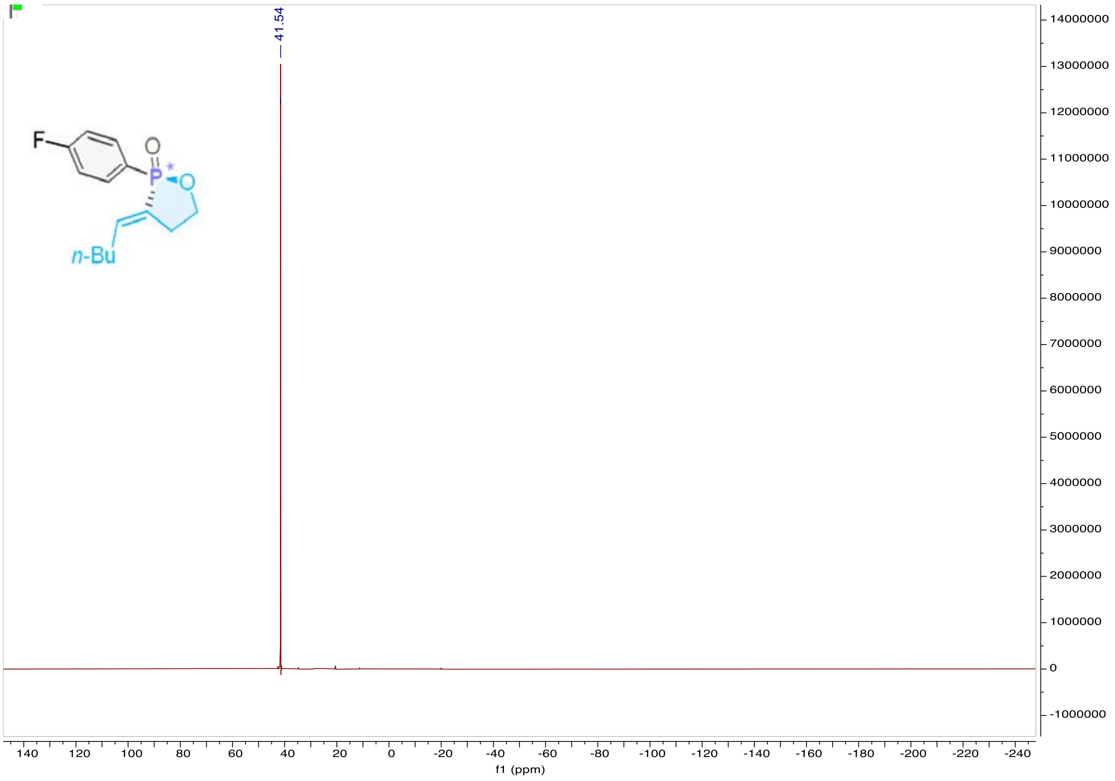
41.54
F
O
P
*
O
n-Bu
f1 (ppm)

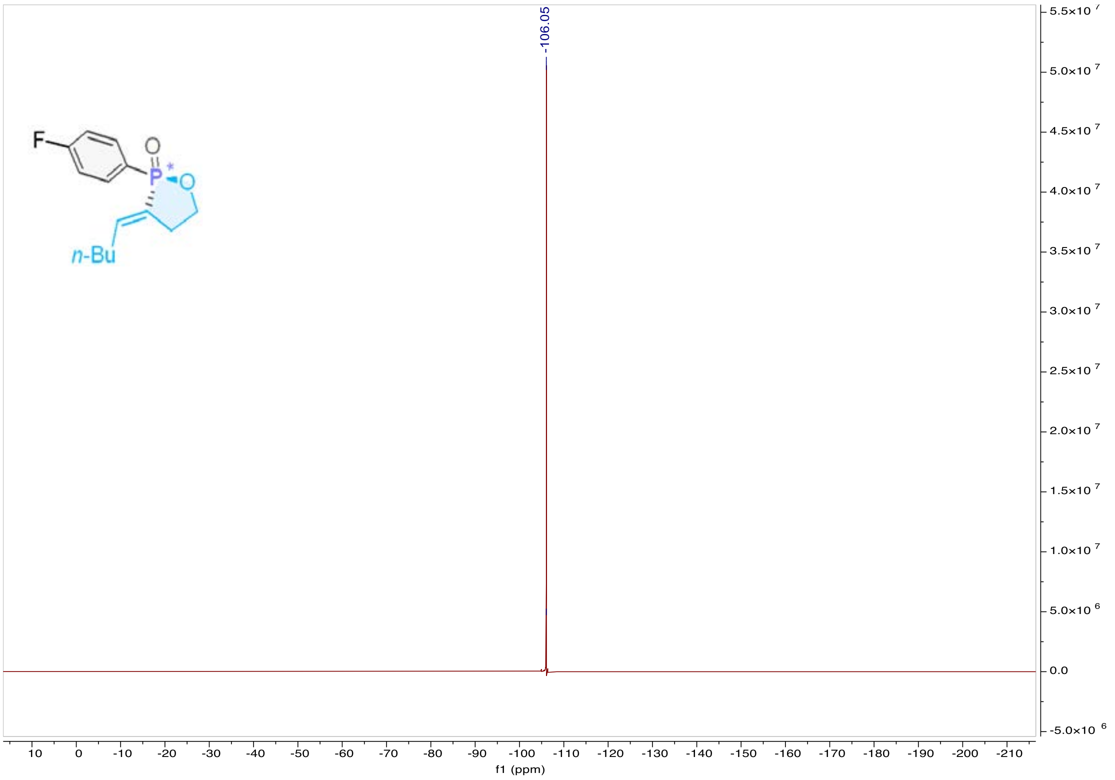

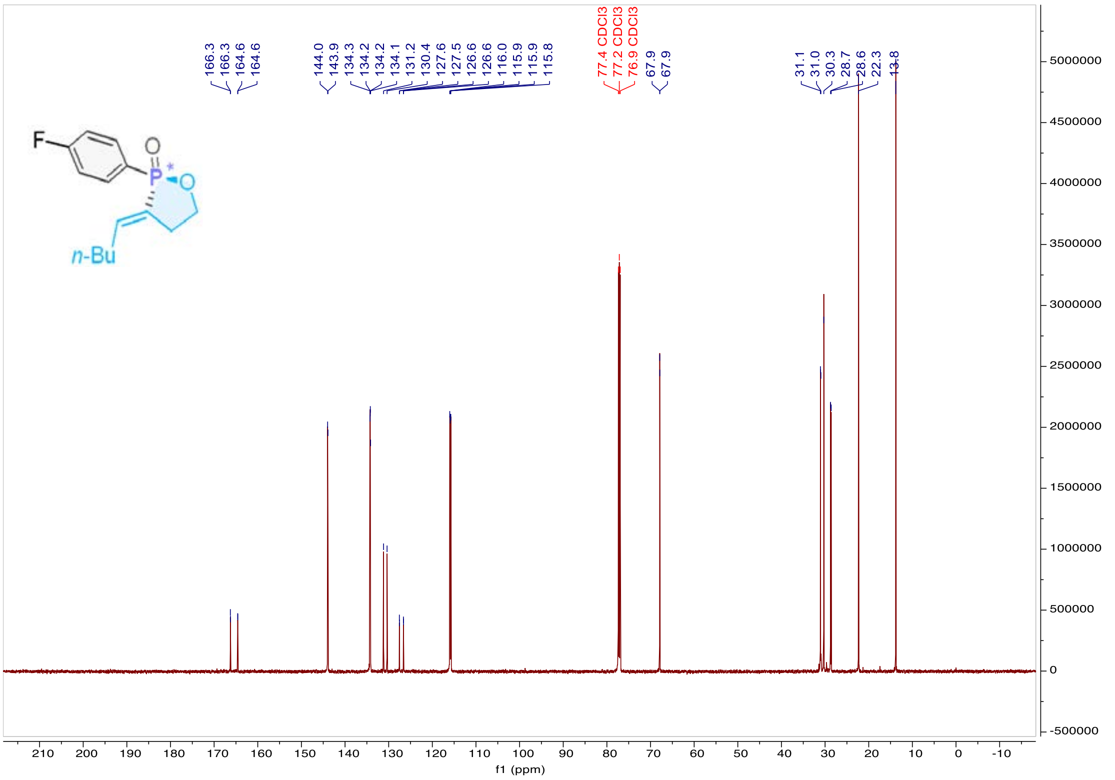

Figure S48. ^{1}H NMR, ^{19}F NMR, ^{31}P NMR and ^{13}C NMR spectra for **2f**

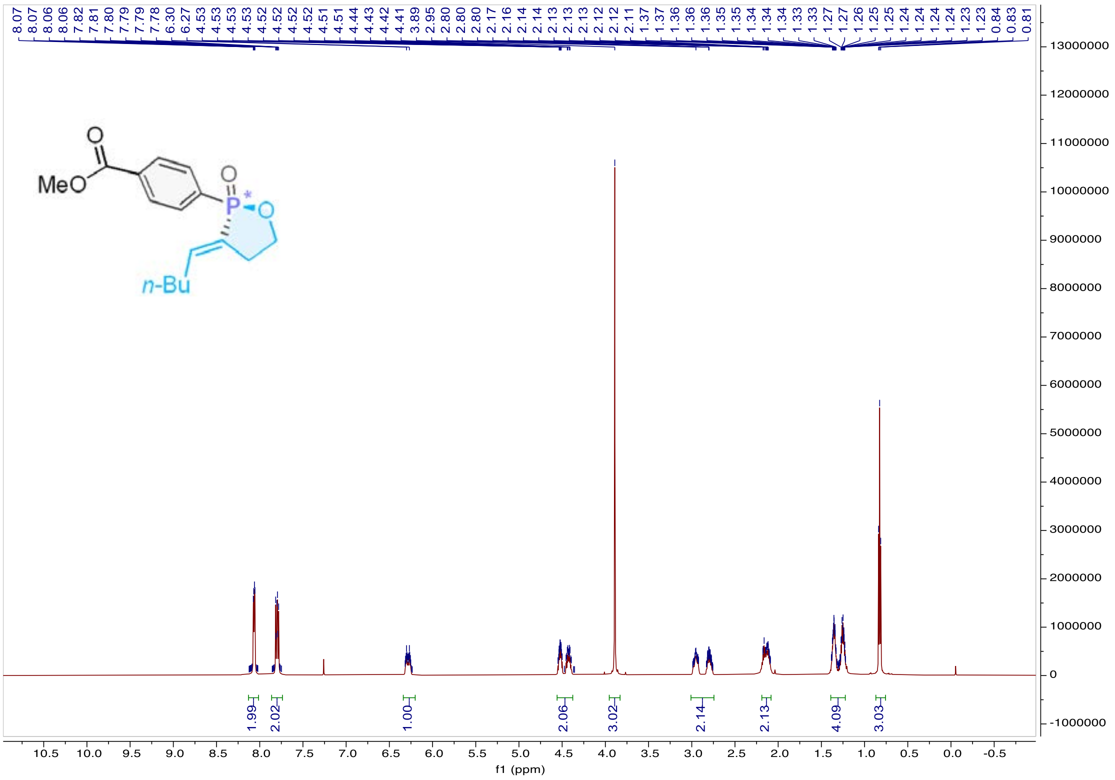

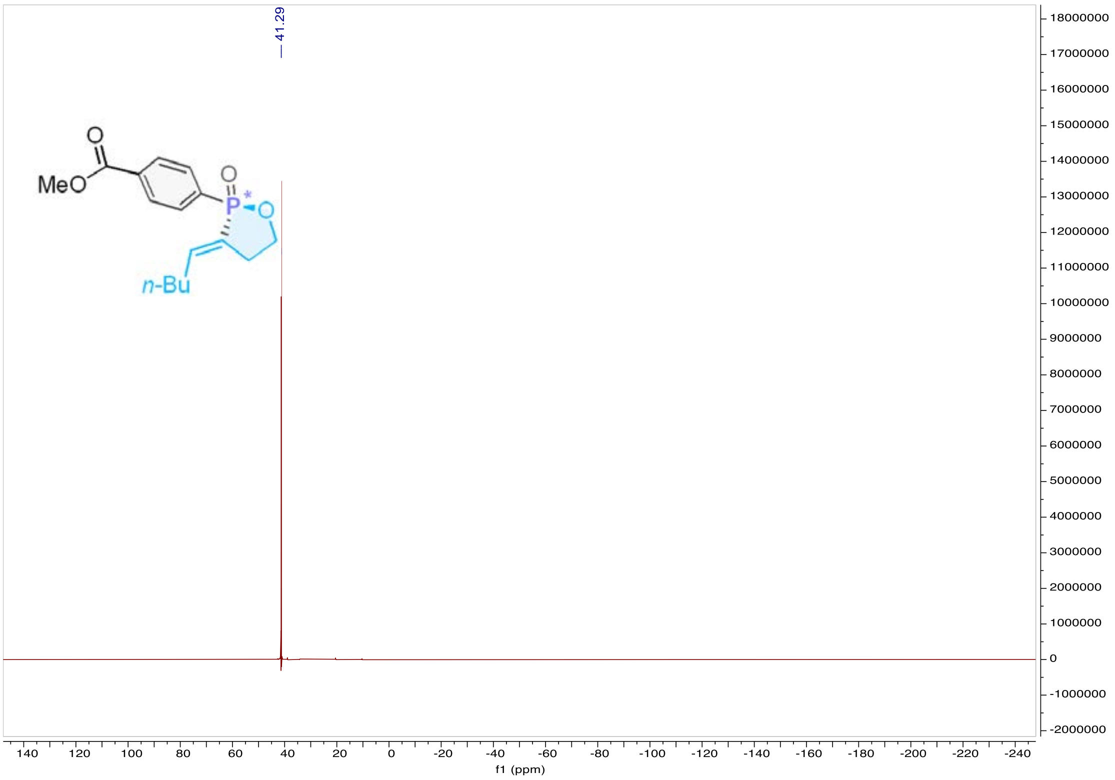

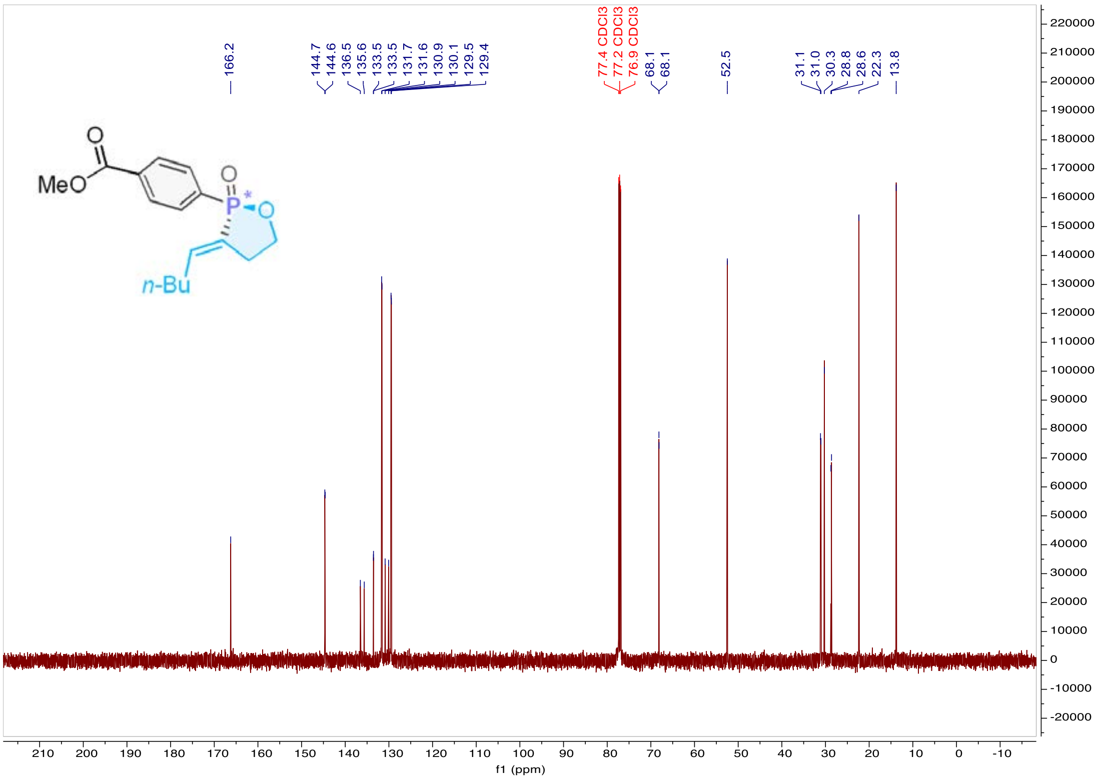

Figure S49. ^{1}H NMR, ^{31}P NMR and ^{13}C NMR spectra for **2g**

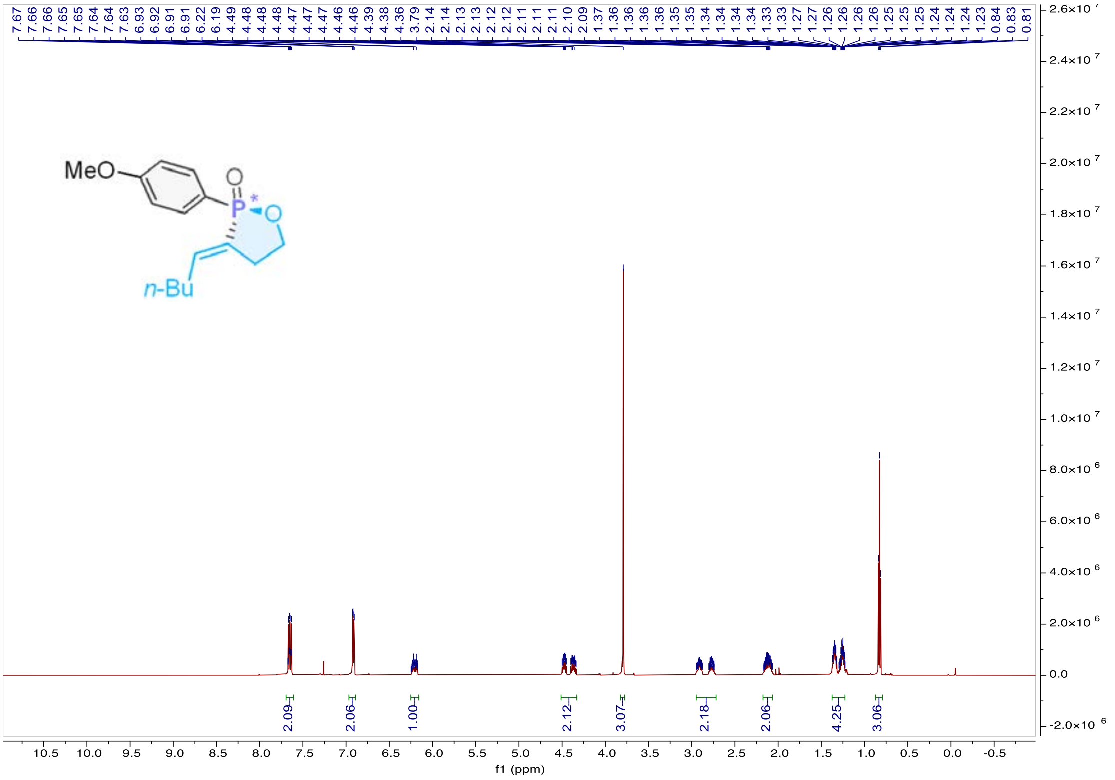
MeO
O
P
*
O
n-Bu
f1 (ppm)

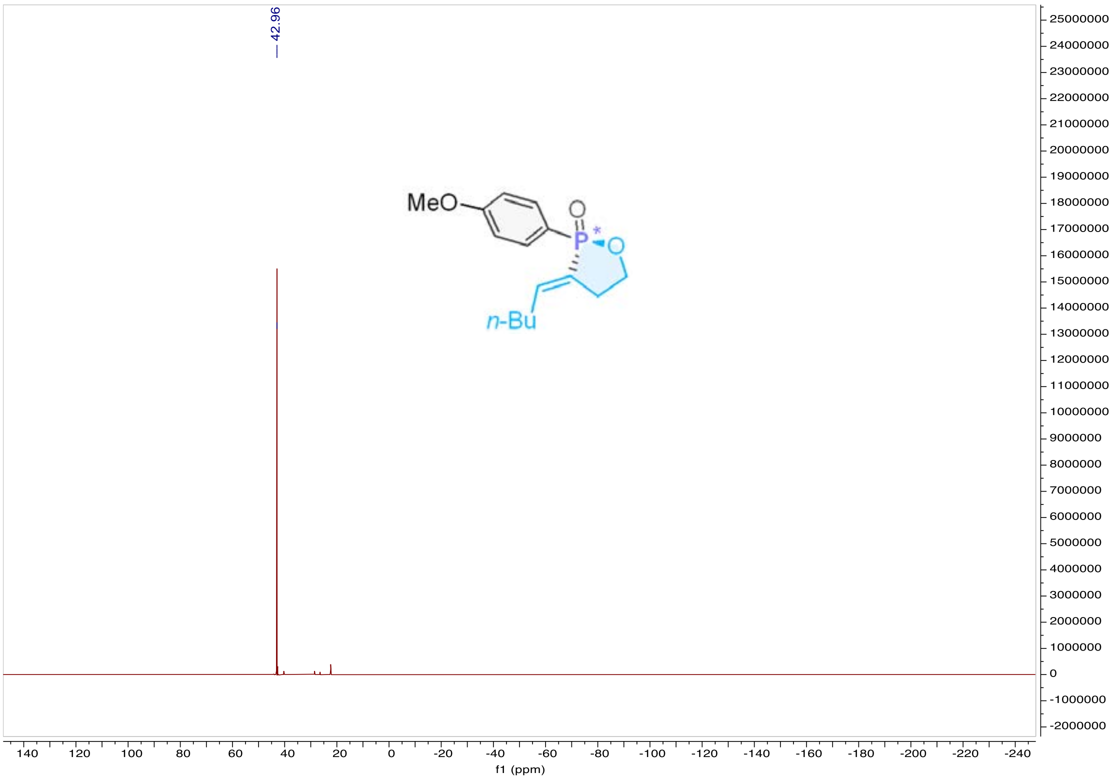
42.96
MeO
O
P
*
O
n-Bu
f1 (ppm)

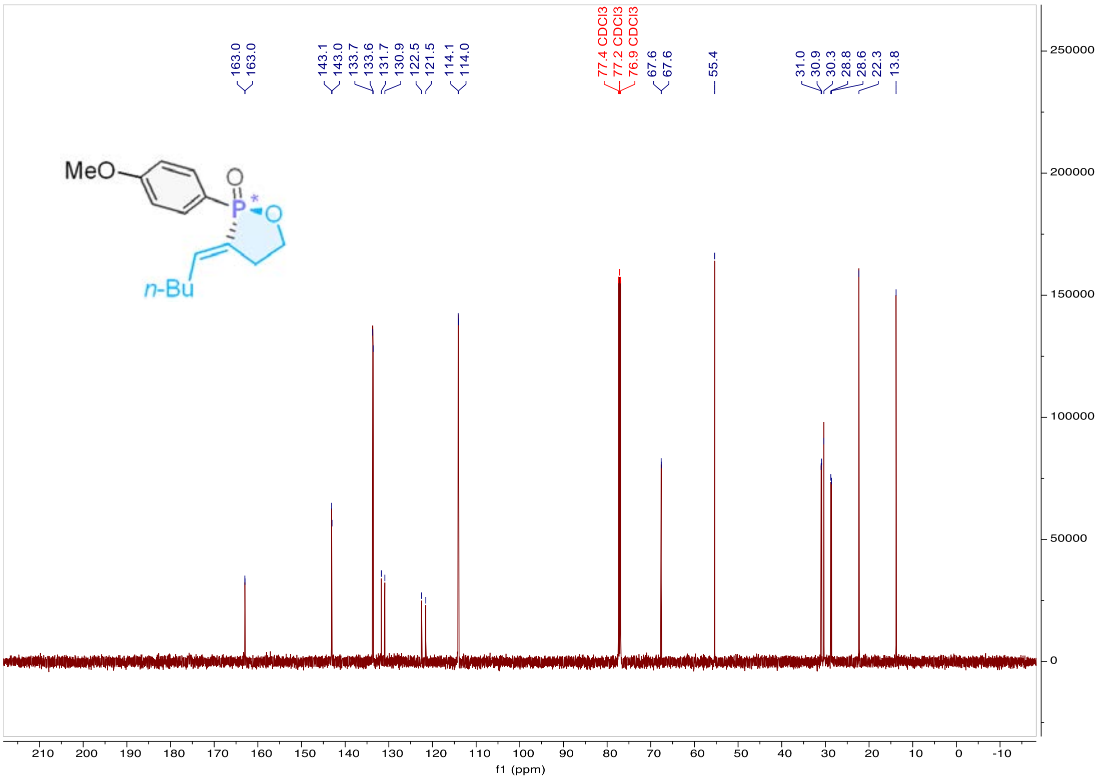

Figure S50. ^{1}H NMR, ^{31}P NMR and ^{13}C NMR spectra for **2h**

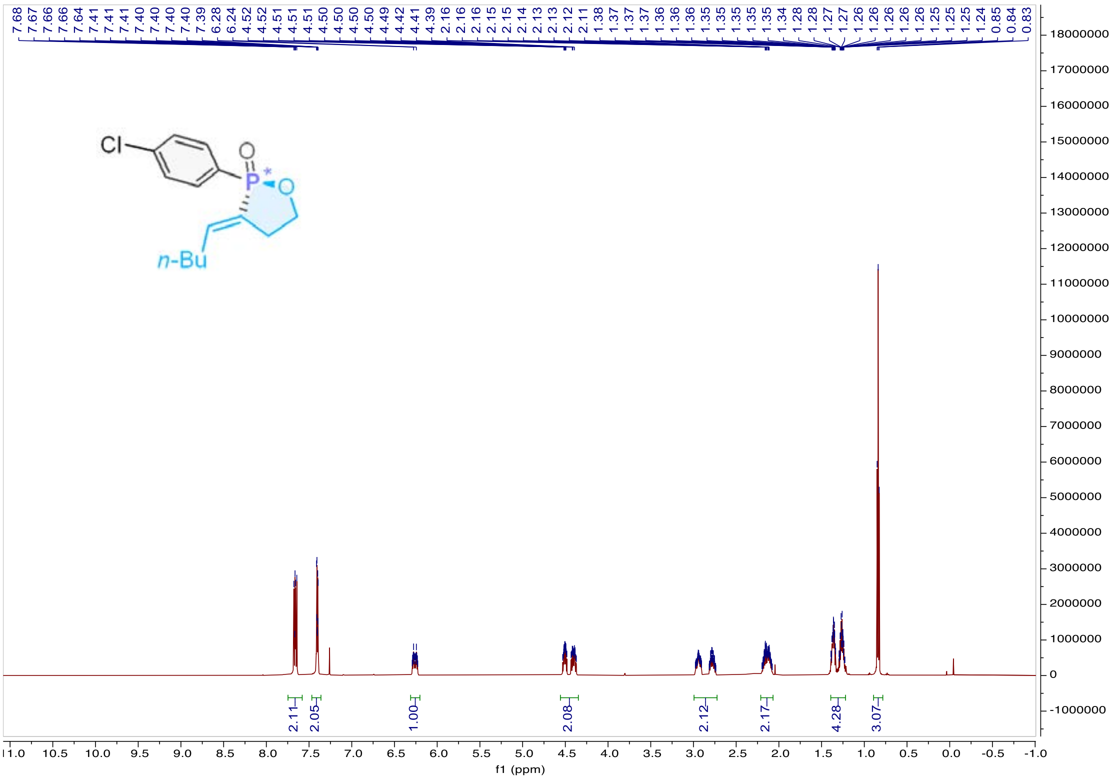
7.68
7.67
7.66
7.66
7.64
7.41
7.41
7.41
7.40
7.40
7.40
7.39
6.28
6.24
4.52
4.52
4.51
4.51
4.51
4.50
4.50
4.50
4.49
4.42
4.41
4.39
2.16
2.16
2.16
2.15
2.15
2.14
2.13
2.12
2.12
2.11
1.38
1.37
1.37
1.37
1.36
1.36
1.35
1.35
1.35
1.35
1.34
1.28
1.28
1.27
1.27
1.26
1.26
1.26
1.26
1.26
1.25
1.25
1.25
1.24
0.85
0.84
0.83
Cl
O
P
*
O
n-Bu
2.11
2.05
1.00
2.08
2.12
2.17
4.28
3.07
f1 (ppm)

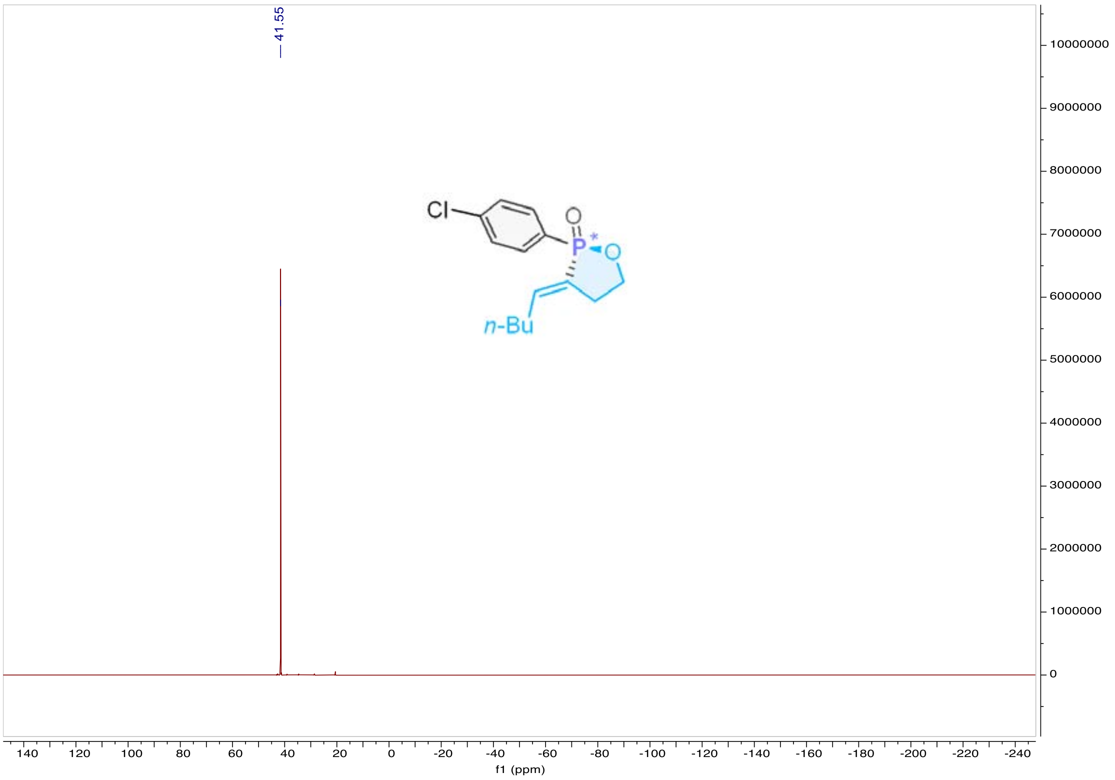
41.55
Cl
O
P
*
O
n-Bu
f1 (ppm)

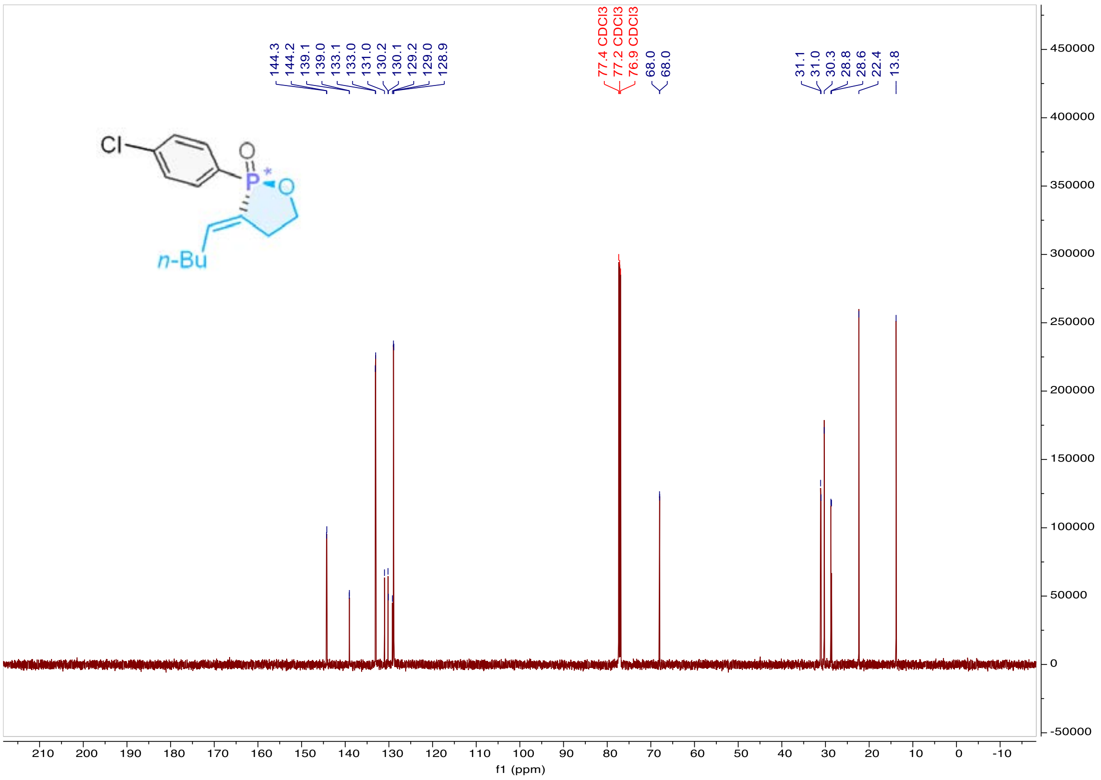

Figure S51. ^{1}H NMR, ^{31}P NMR and ^{13}C NMR spectra for **2i**

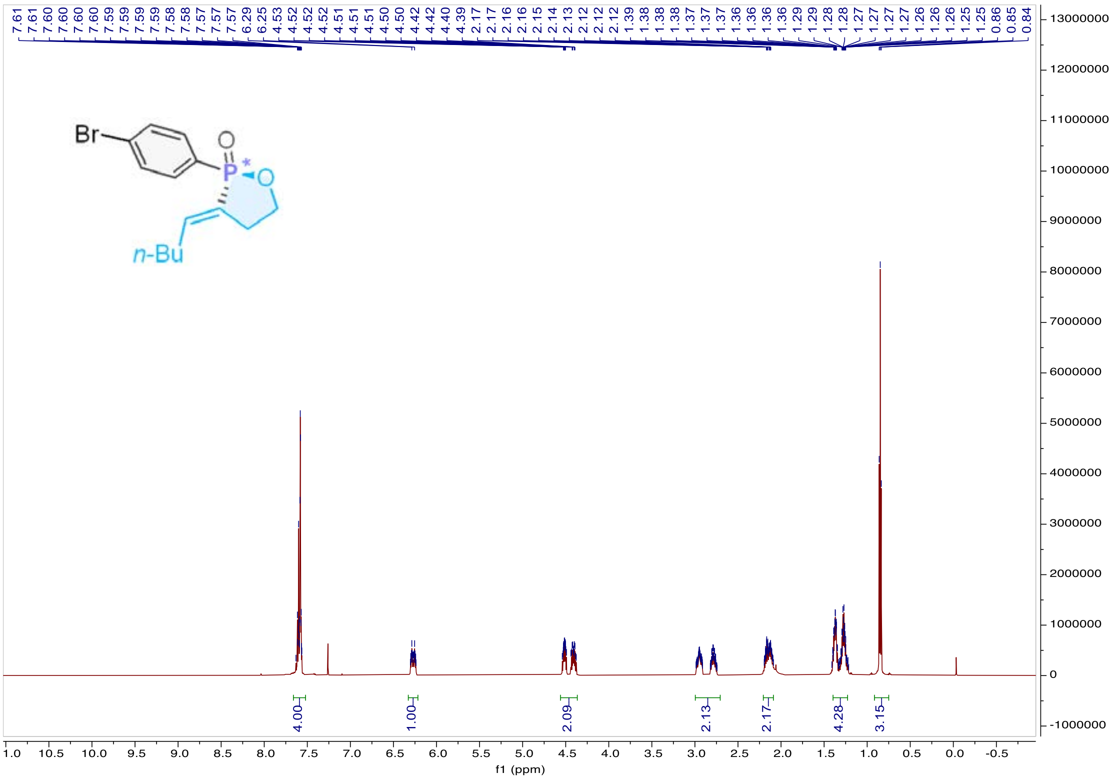

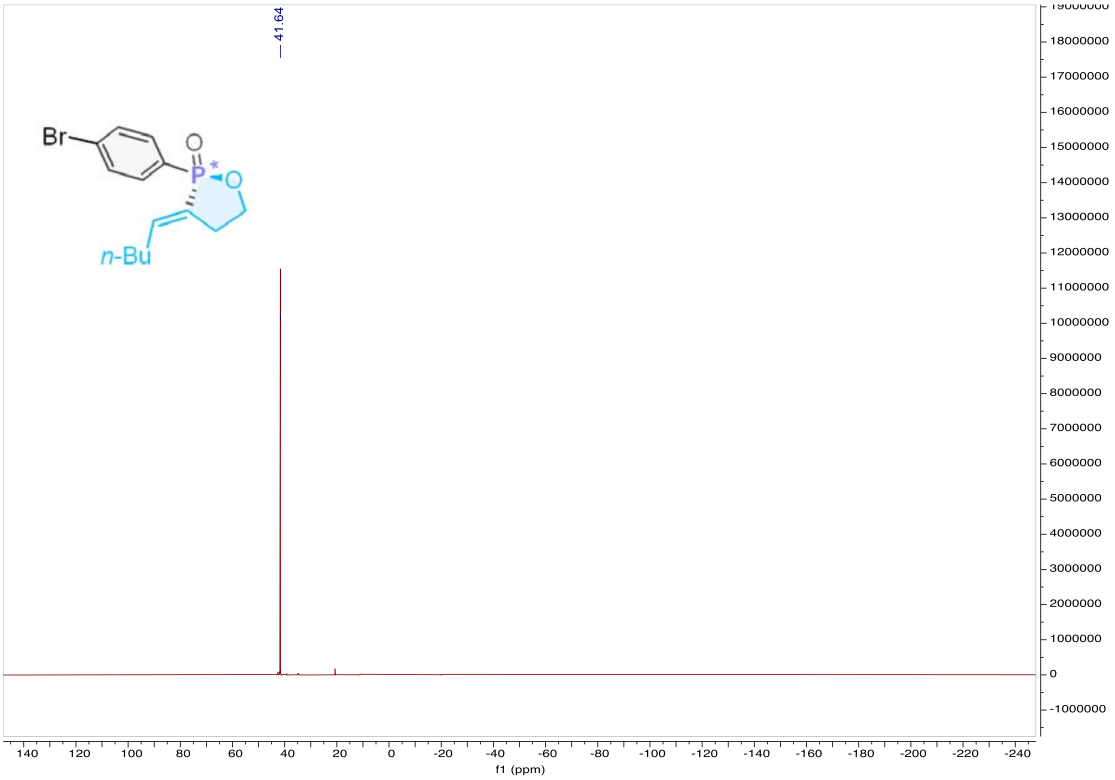

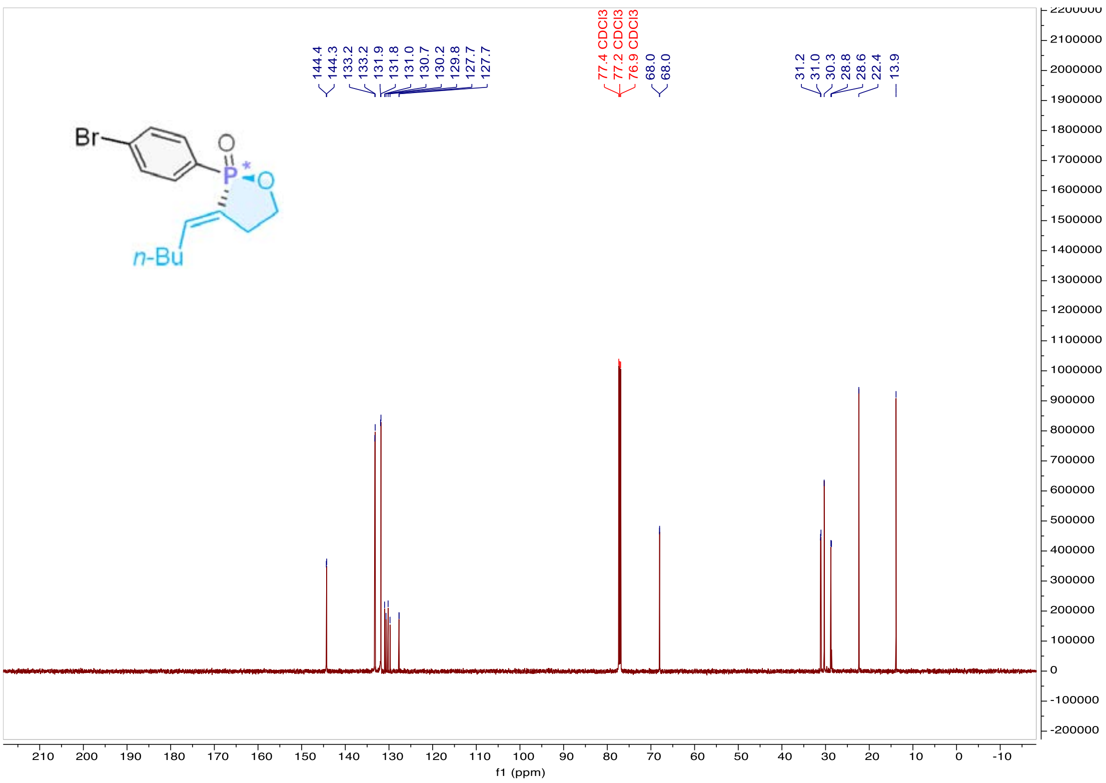

Figure S52. ^{1}H NMR, ^{31}P NMR and ^{13}C NMR spectra for **2j**

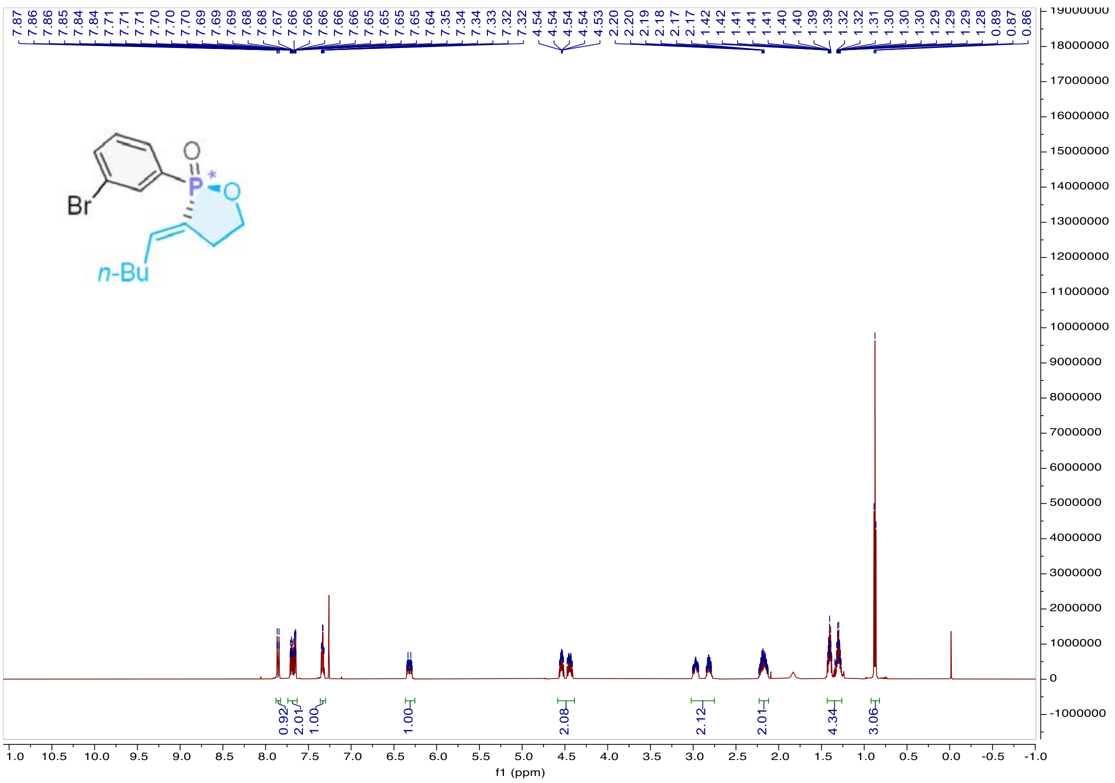

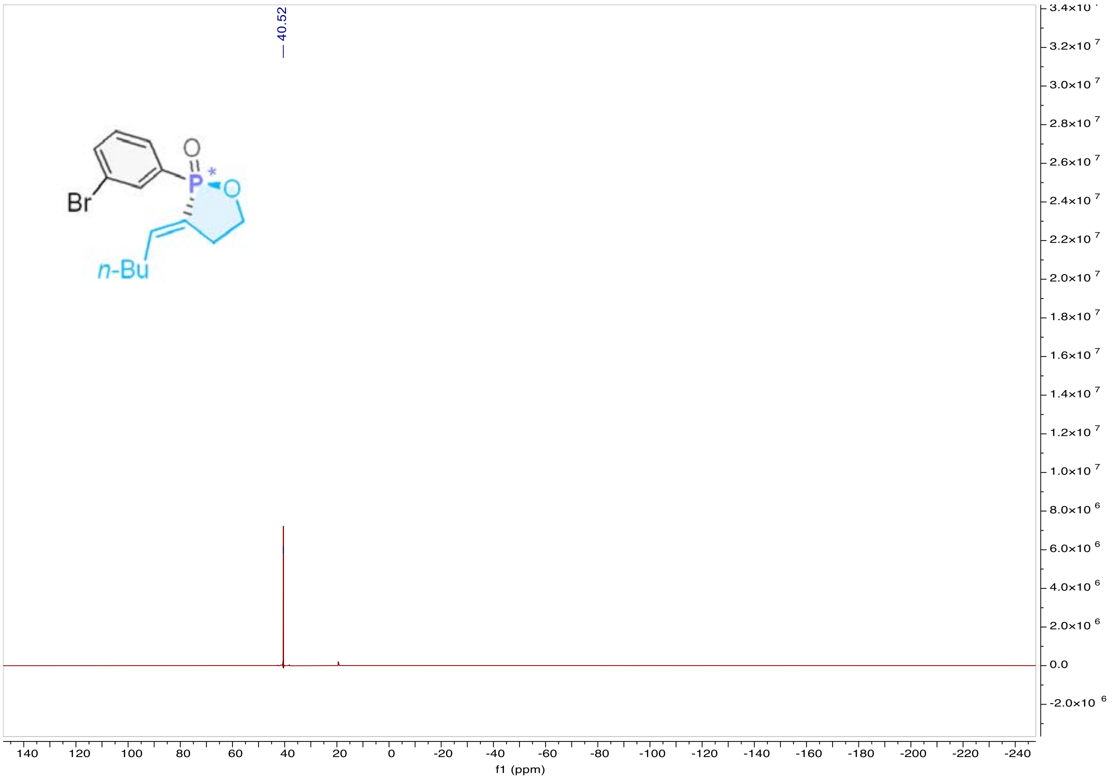

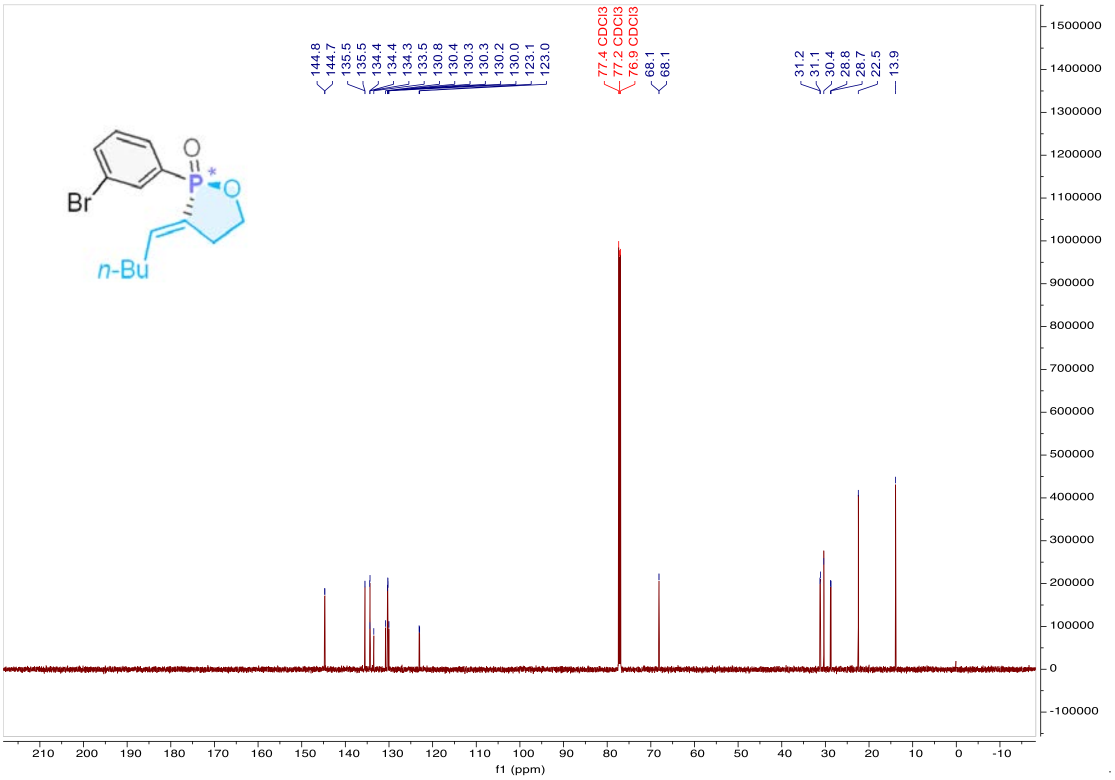

Figure S53. ^{1}H NMR, ^{31}P NMR and ^{13}C NMR spectra for **2k**

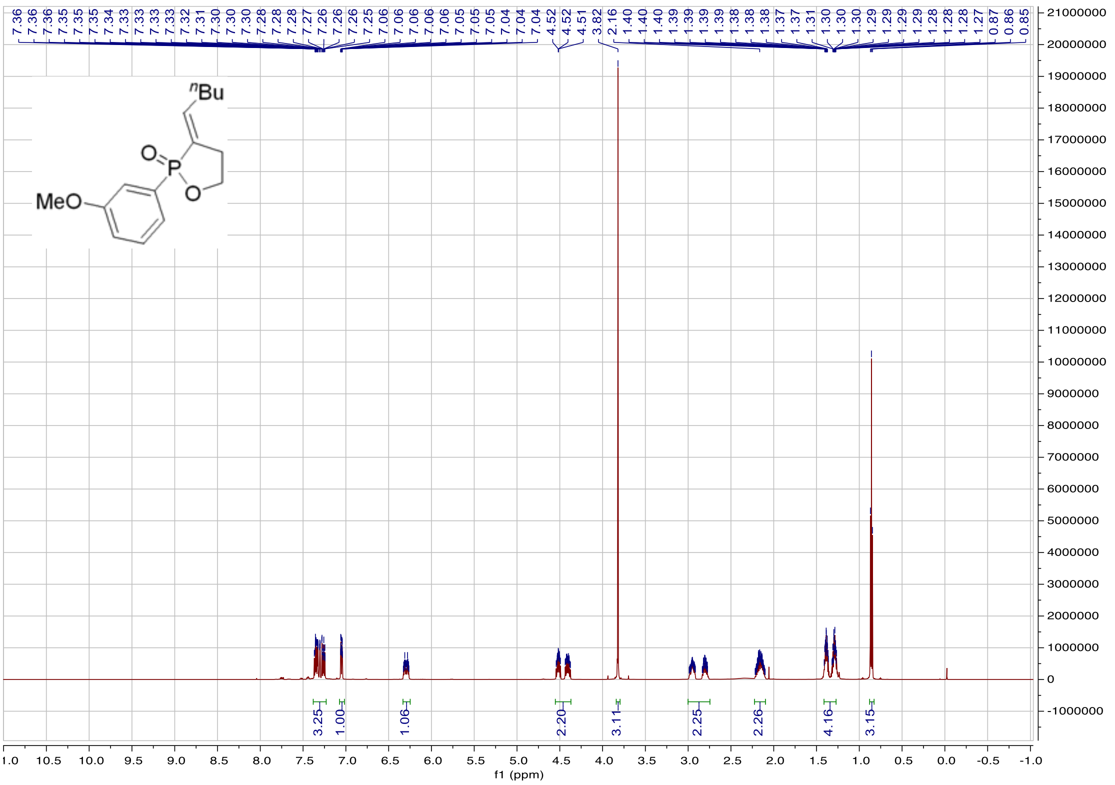

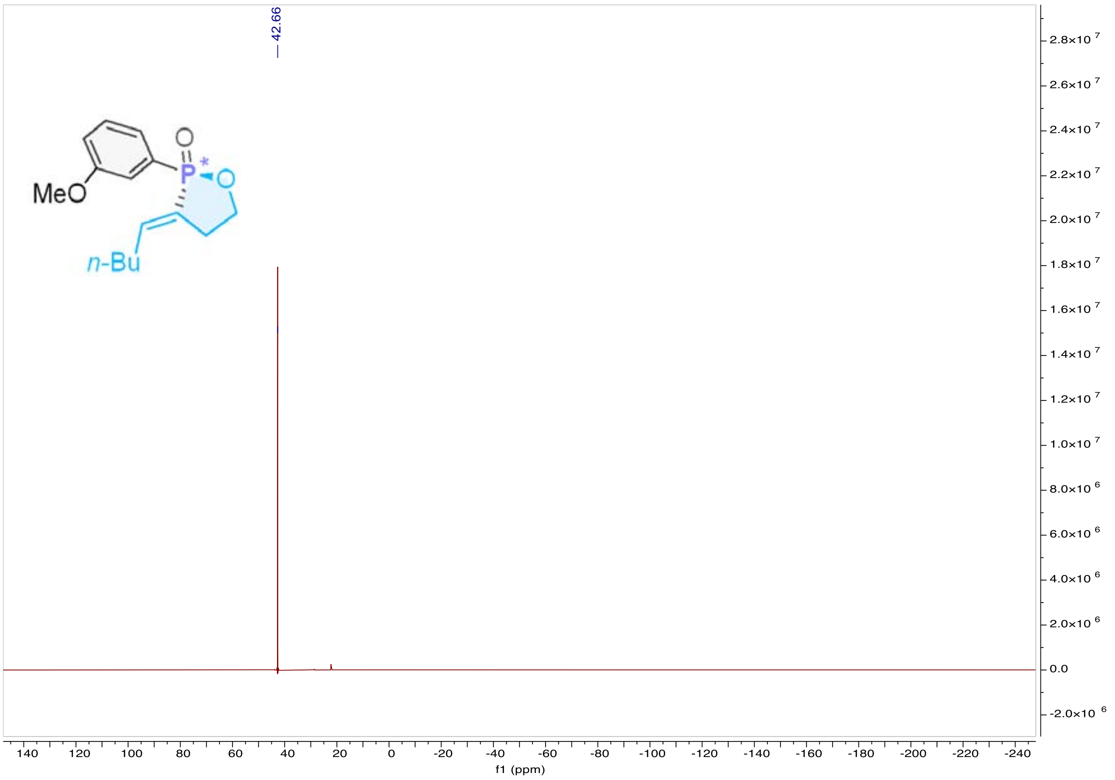

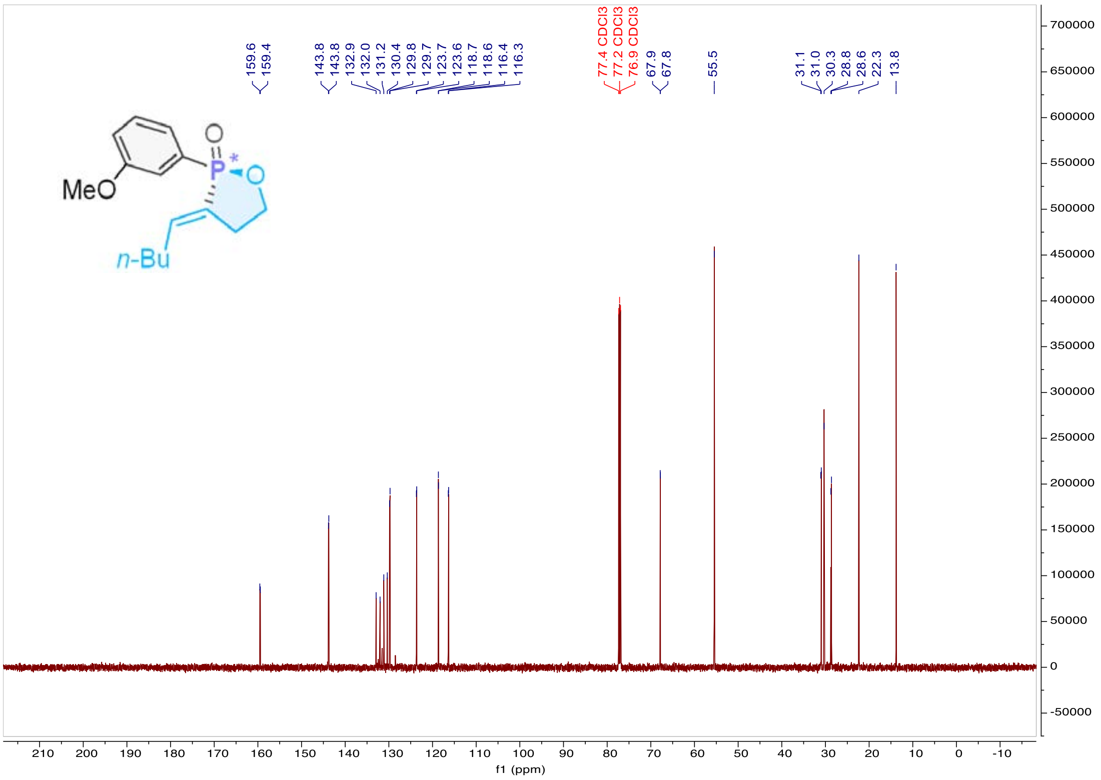

Figure S54. ^{1}H NMR, ^{31}P NMR and ^{13}C NMR spectra for **2l**

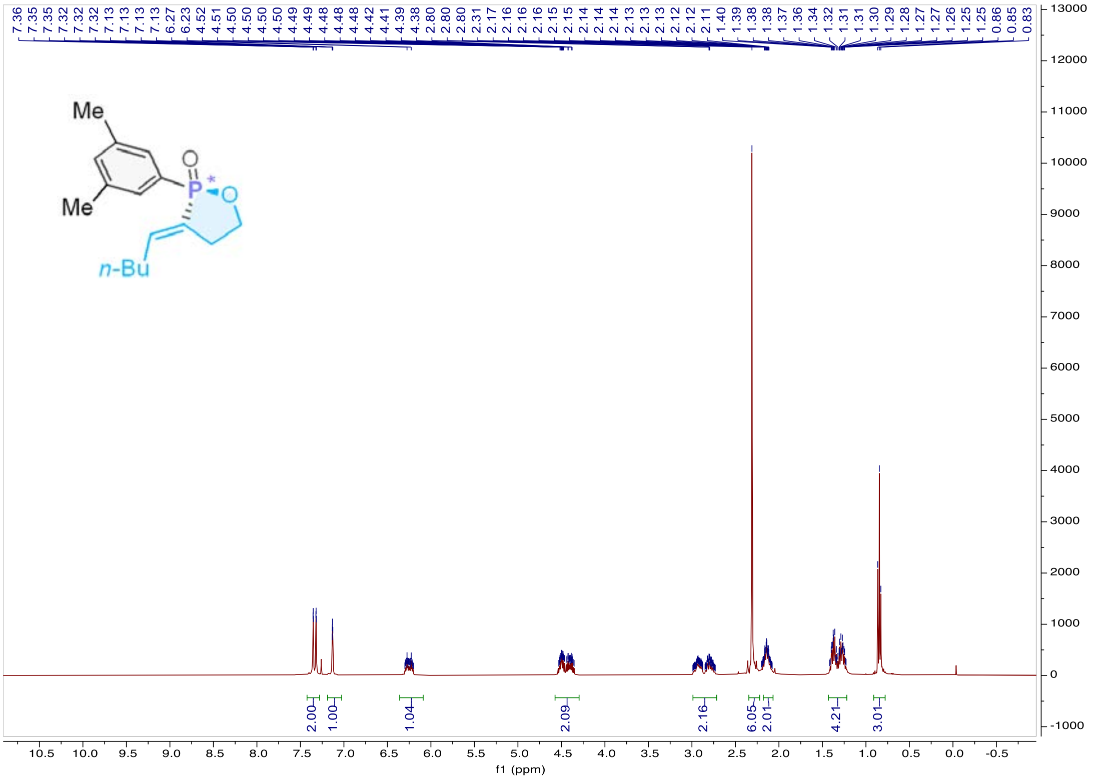
7.36 7.35 7.35 7.32 7.32 7.32 7.13 7.13 7.13 7.13 6.27 6.23 4.52 4.51 4.50 4.50 4.50 4.49 4.49 4.48 4.48 4.42 4.41 4.39 4.38 2.80 2.80 2.80 2.31 2.17 2.16 2.16 2.15 2.15 2.14 2.14 2.14 2.13 2.13 2.13 2.12 2.12 2.11 1.40 1.39 1.38 1.38 1.37 1.36 1.34 1.32 1.31 1.31 1.30 1.29 1.28 1.27 1.27 1.26 1.25 1.25 0.86 0.85 0.83
Me
Me
O
P
*
O
n-Bu
2.00
1.00
1.04
2.09
2.16
6.05
2.01
4.21
3.01
f1 (ppm)

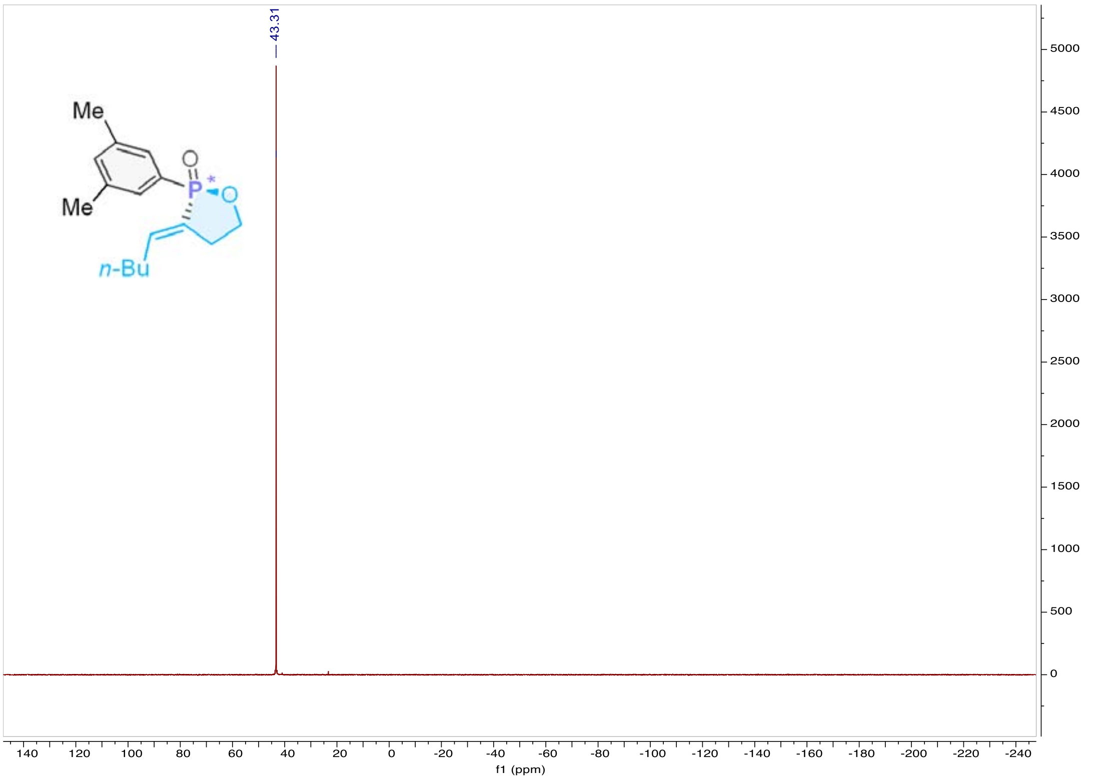
43.31
Me
Me
O
P
*
O
n-Bu
f1 (ppm)

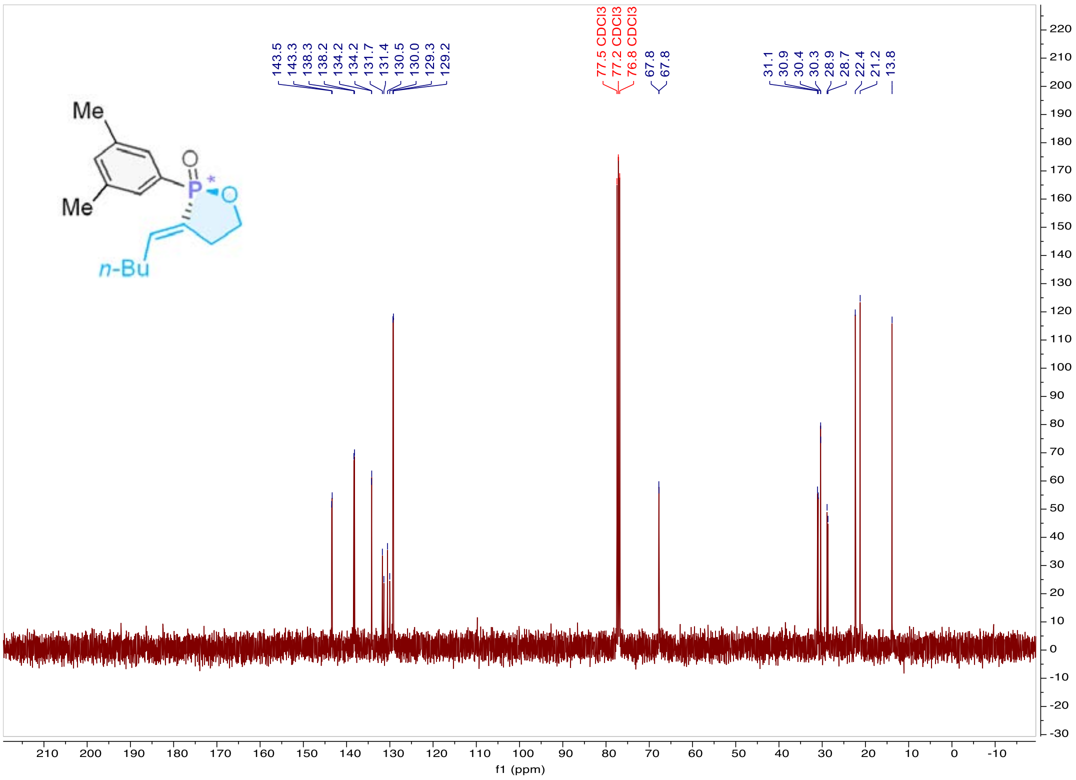

Figure S55. ^{1}H NMR, ^{31}P NMR and ^{13}C NMR spectra for **2m**

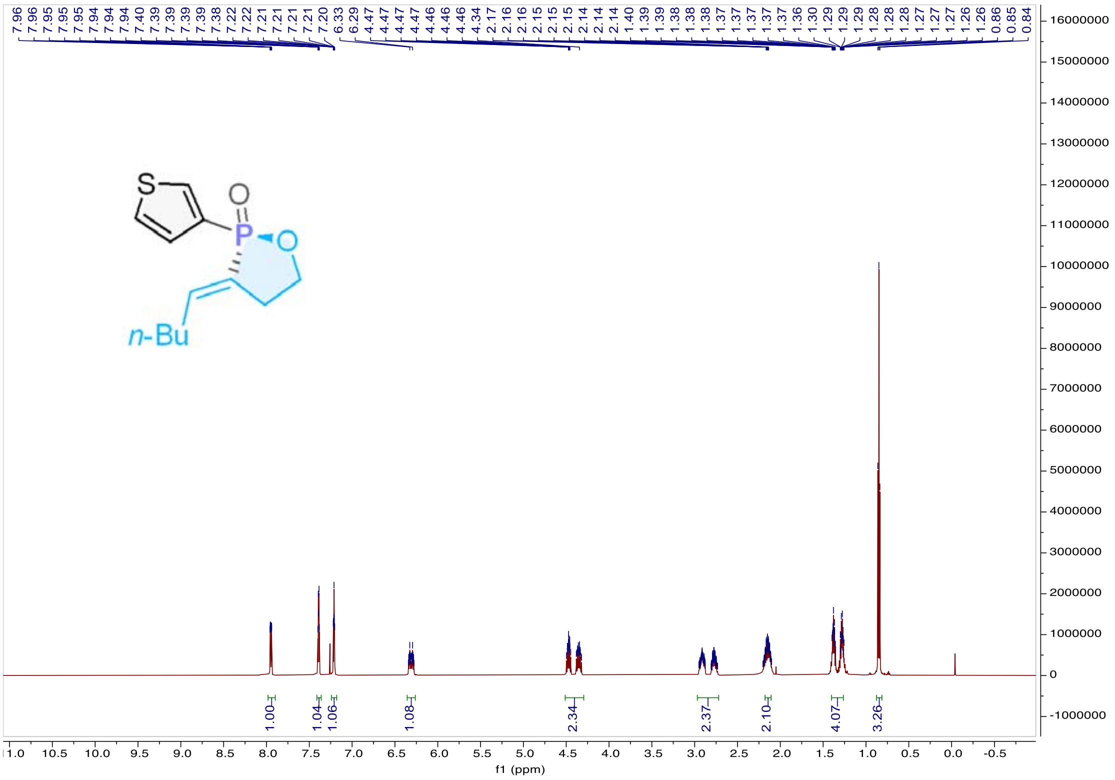
n-Bu
f1 (ppm)

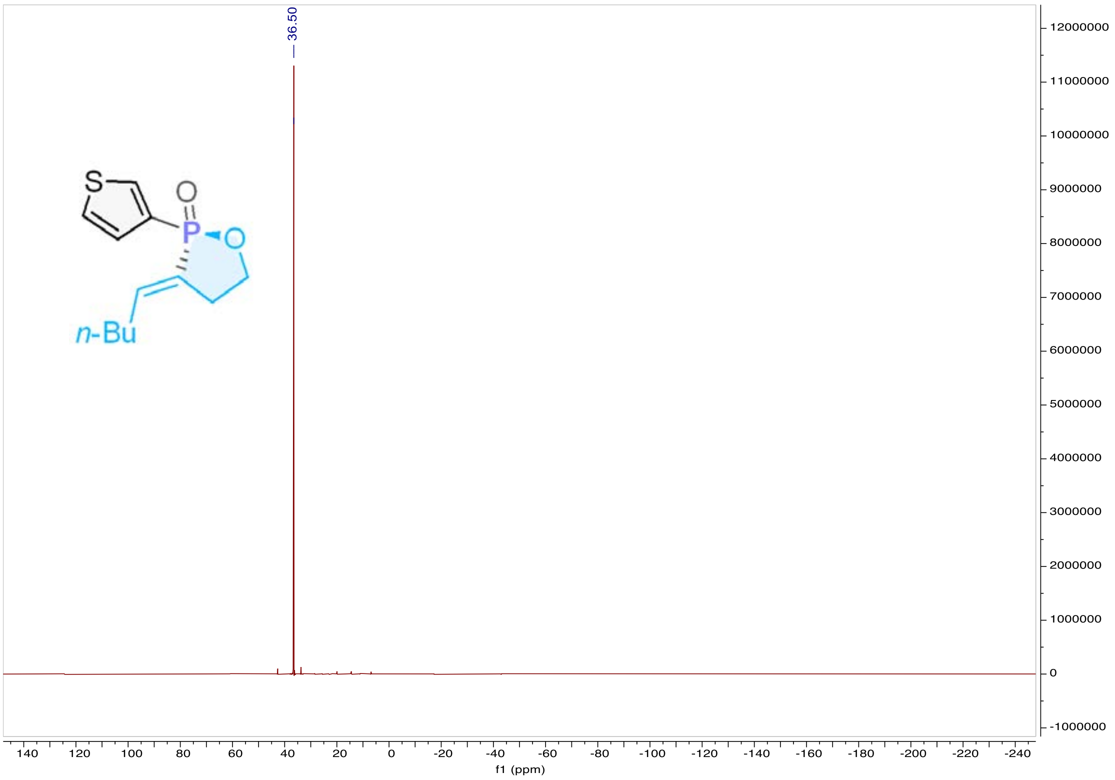
36.50
n-Bu
f1 (ppm)

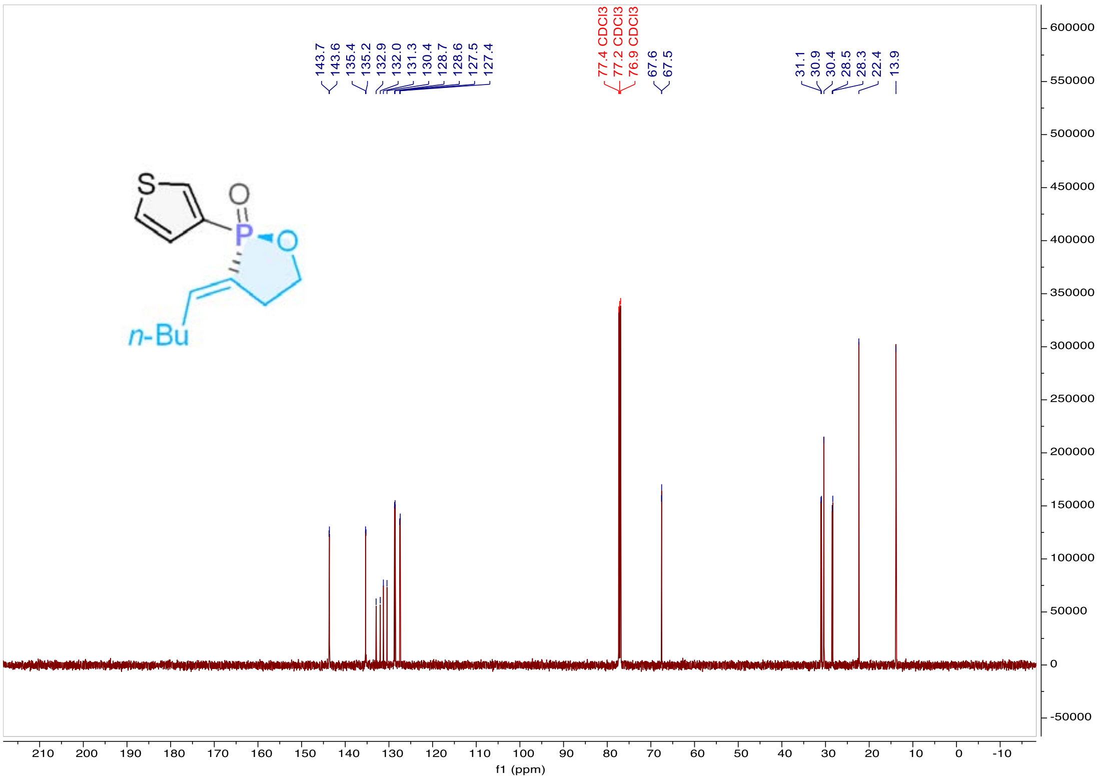

Figure S56. ^{1}H NMR, ^{31}P NMR and ^{13}C NMR spectra for **2n**

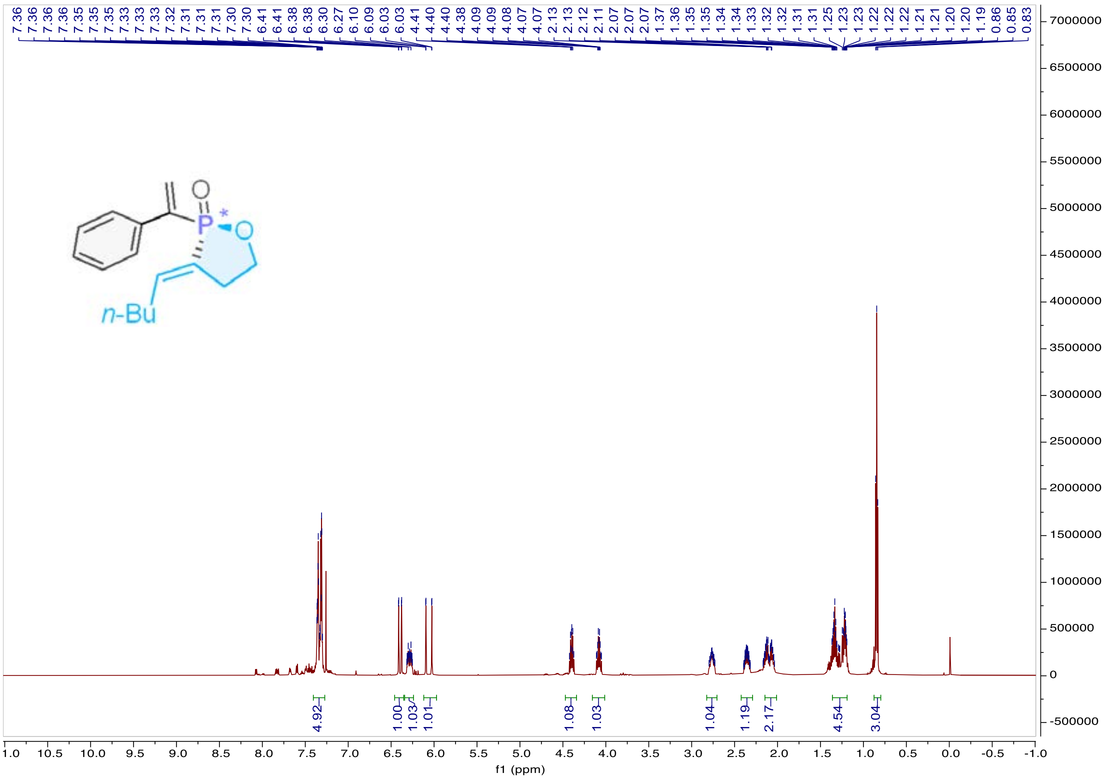

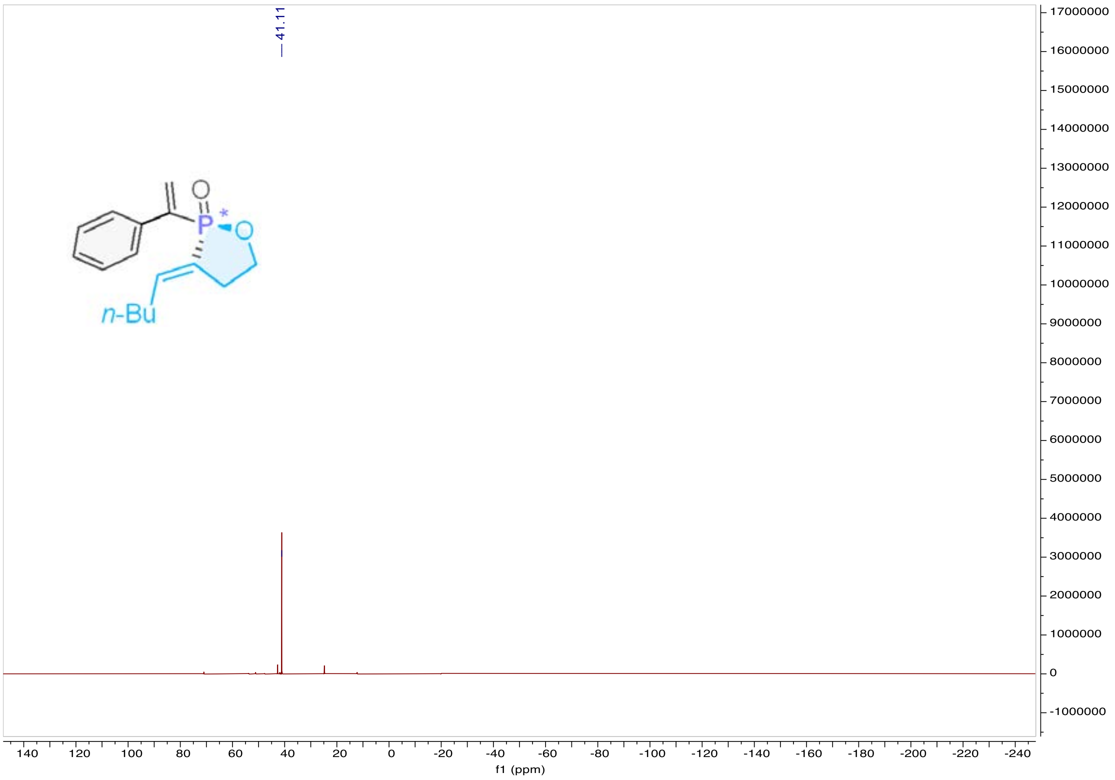

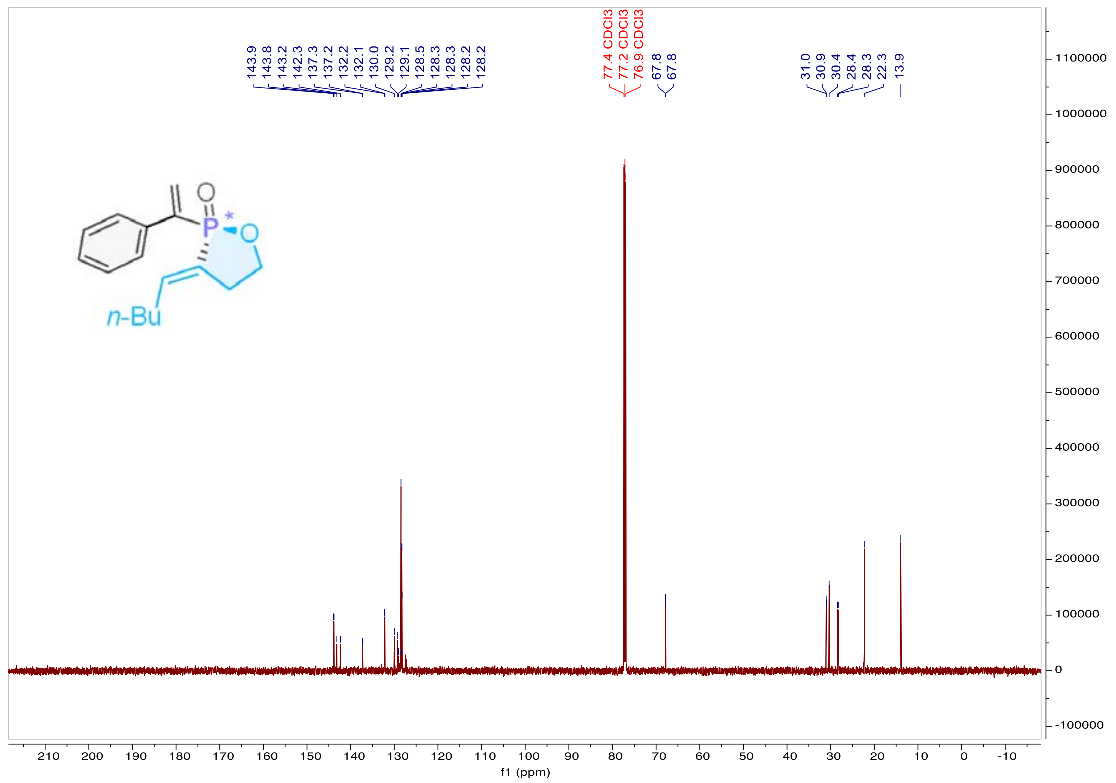

Figure S57. ^{1}H NMR, ^{31}P NMR and ^{13}C NMR spectra for **2o**

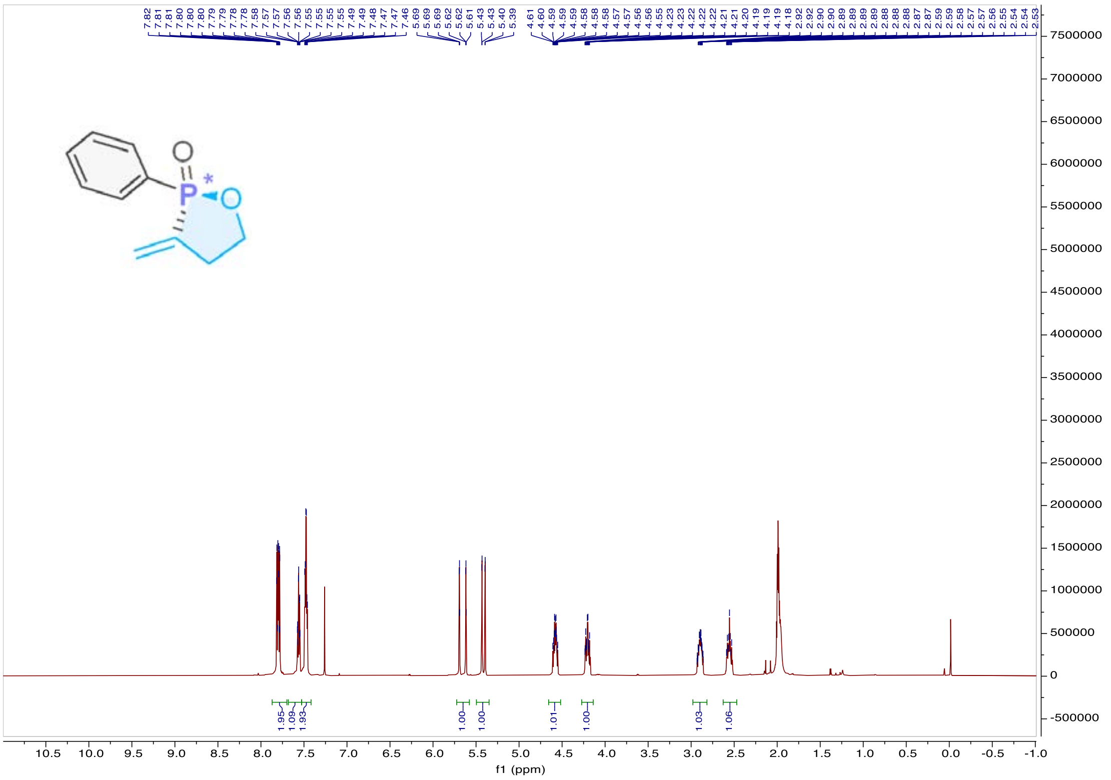

7.82 7.81 7.80 7.80 7.80 7.79 7.78 7.78 7.58 7.57 7.57 7.56 7.55 7.55 7.55 7.55 7.49 7.49 7.48 7.47 7.47 7.46 5.69 5.69 5.62 5.61 5.43 5.43 5.40 5.39 4.61 4.60 4.59 4.59 4.59 4.58 4.58 4.58 4.57 4.57 4.56 4.55 4.23 4.23 4.22 4.22 4.22 4.21 4.21 4.20 4.19 4.19 4.19 4.18 2.92 2.92 2.90 2.90 2.89 2.89 2.89 2.89 2.88 2.88 2.88 2.87 2.87 2.59 2.59 2.58 2.57 2.57 2.56 2.55 2.54 2.54 2.53
O
P
*
O
1.95
1.09
1.93
1.00
1.00
1.01
1.00
1.03
1.06
f1 (ppm)

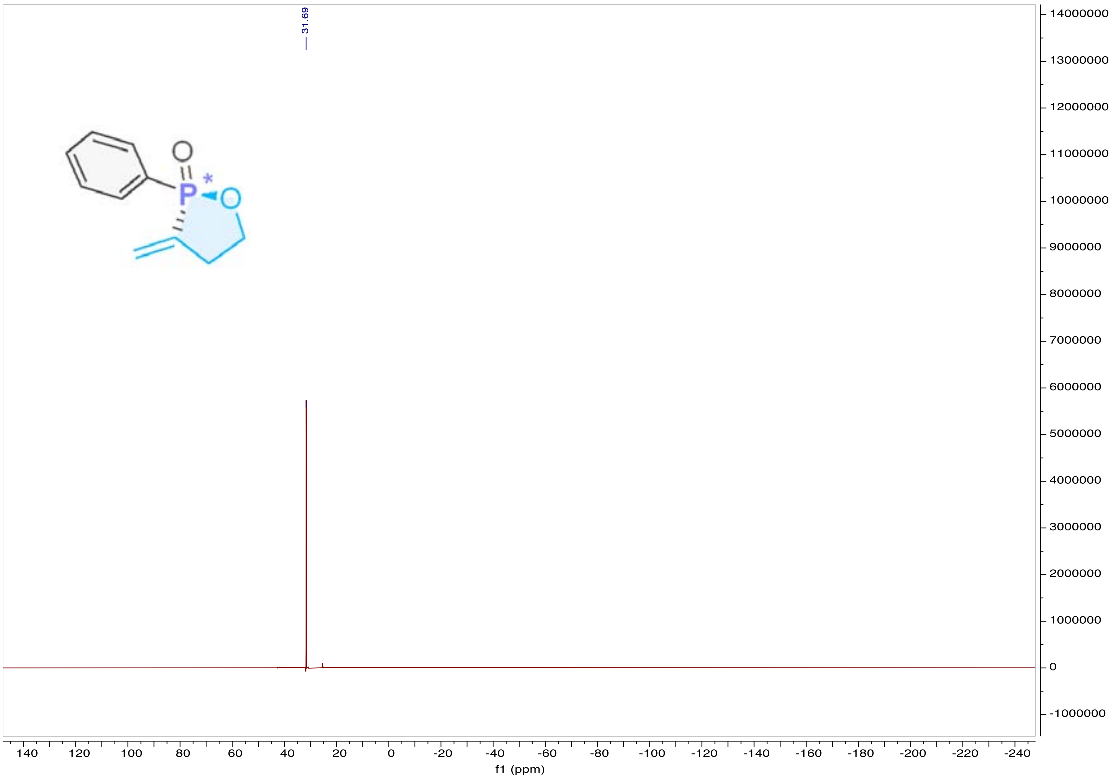

31.69
O
P
*
O
f1 (ppm)

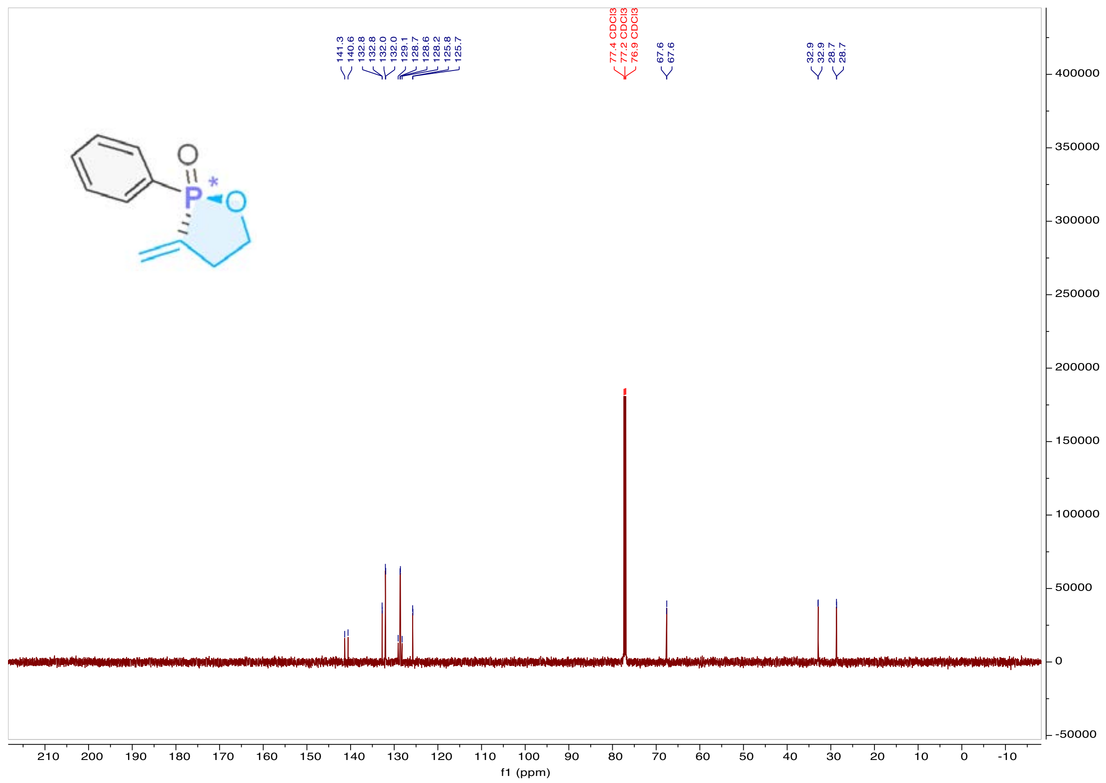

Figure S58. ^{1}H NMR, ^{31}P NMR and ^{13}C NMR spectra for **2p**

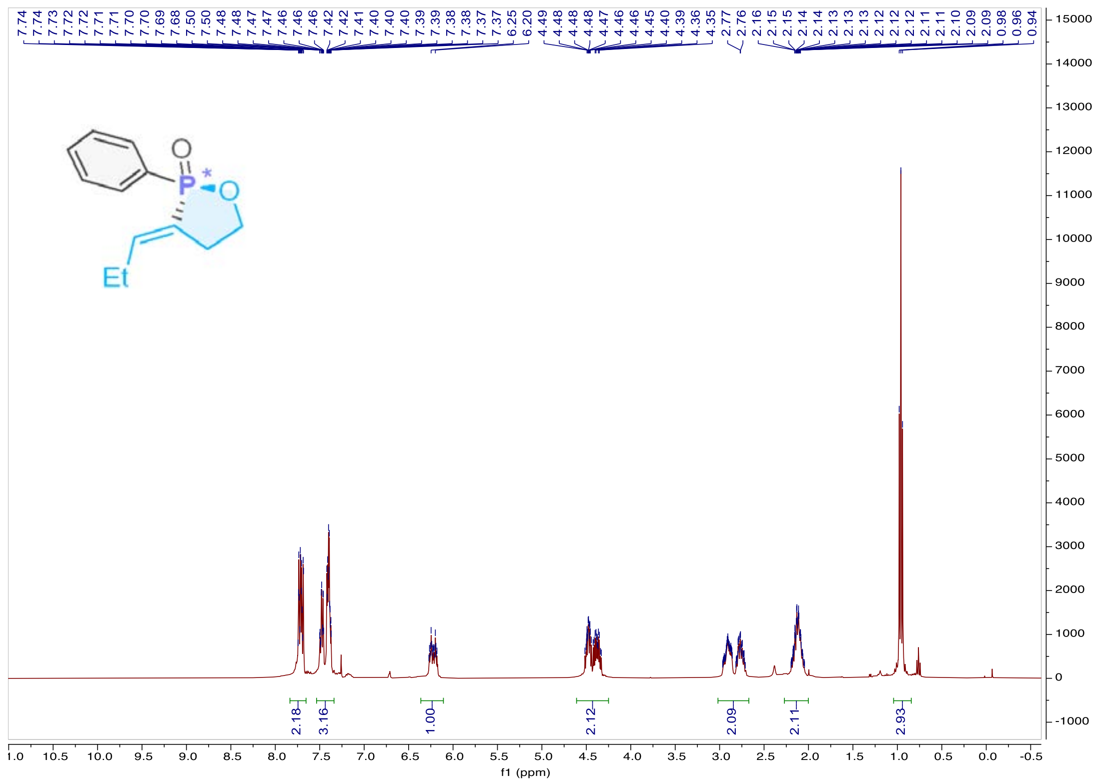

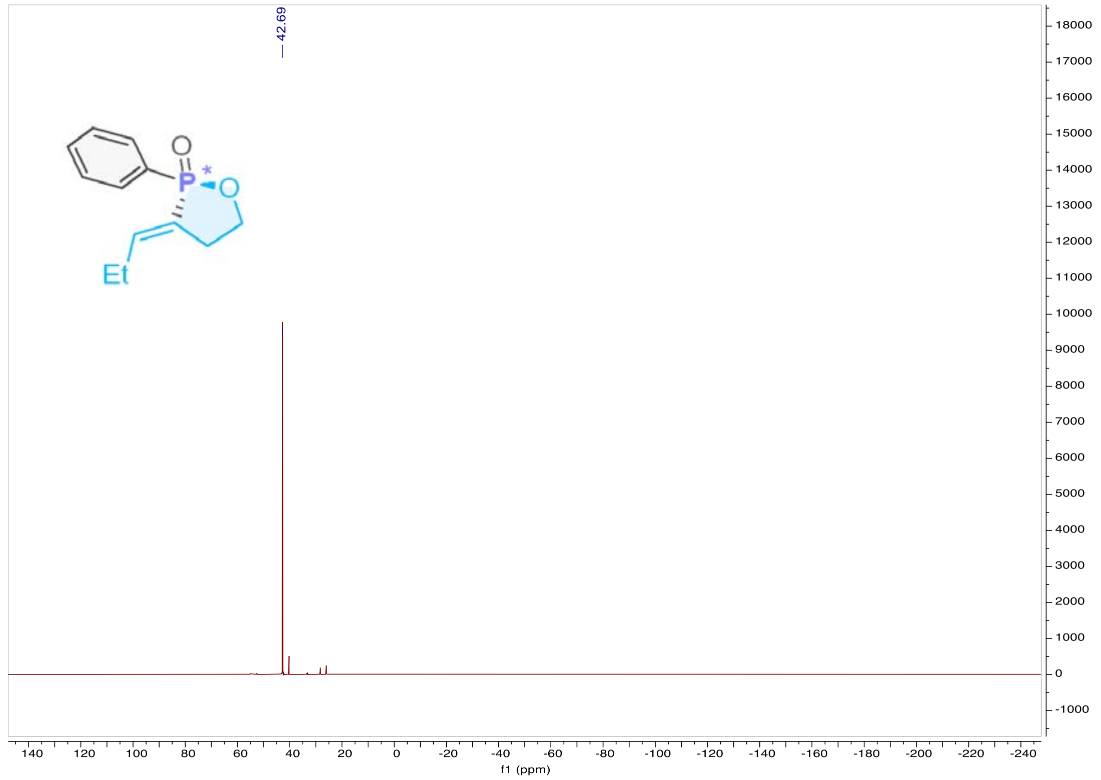

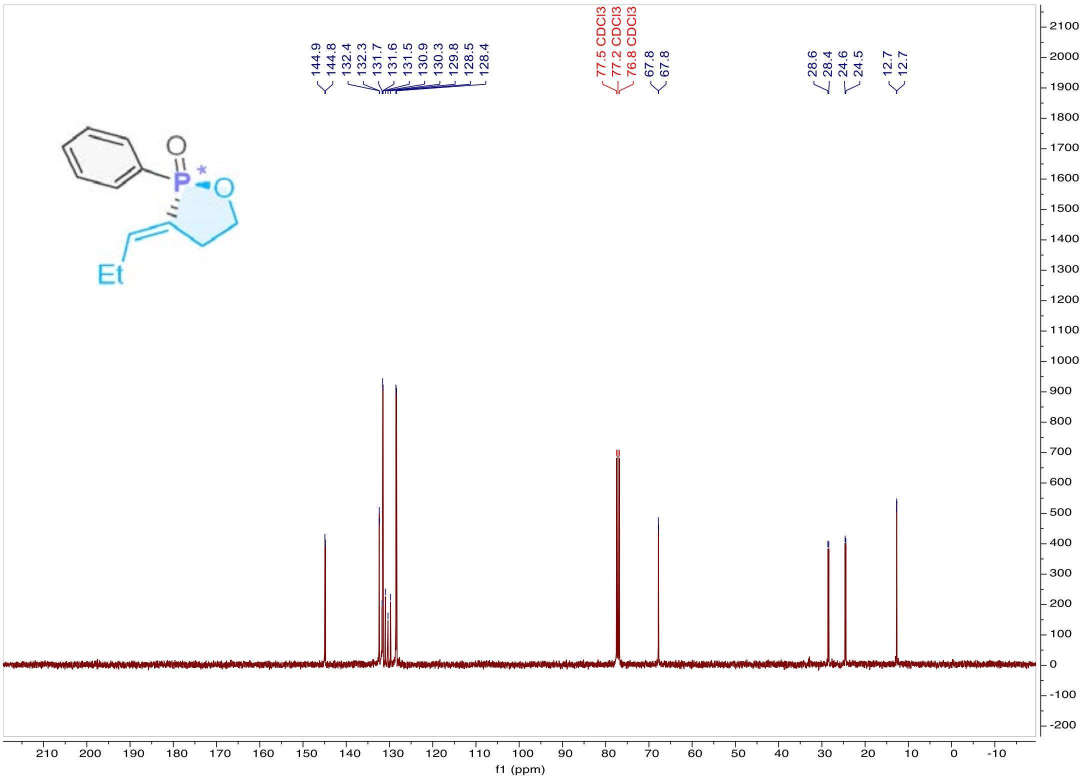

Figure S59. ^{1}H NMR, ^{31}P NMR and ^{13}C NMR spectra for **2q**

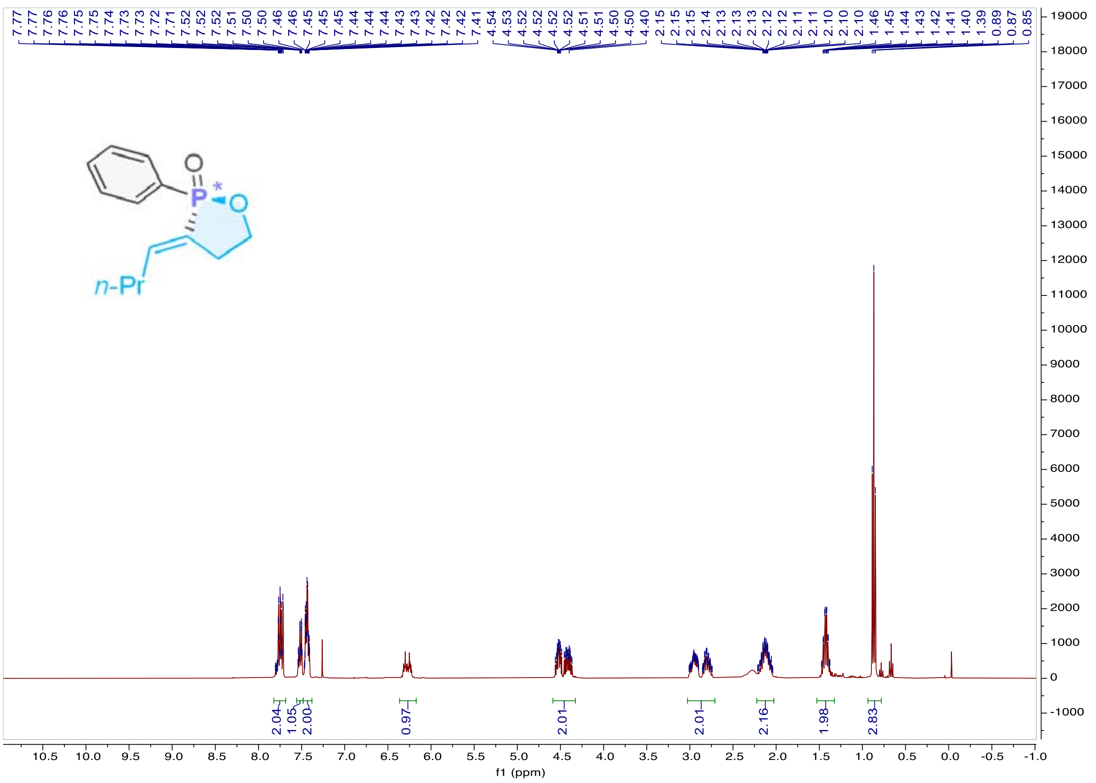

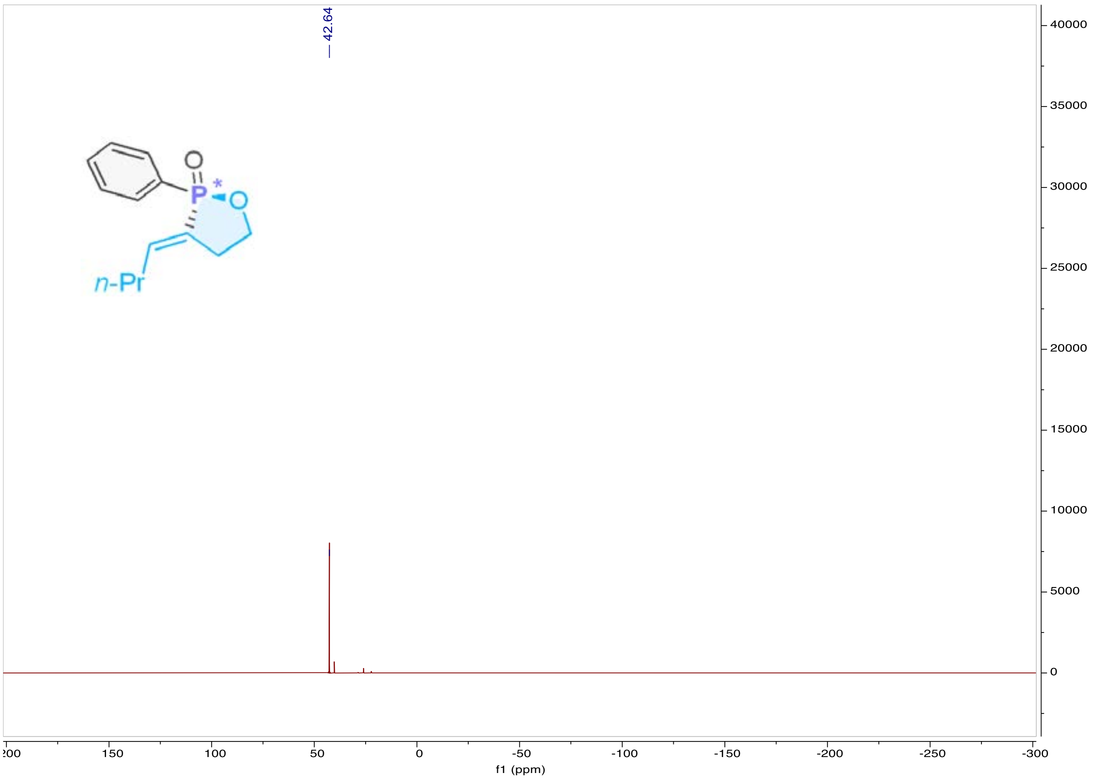

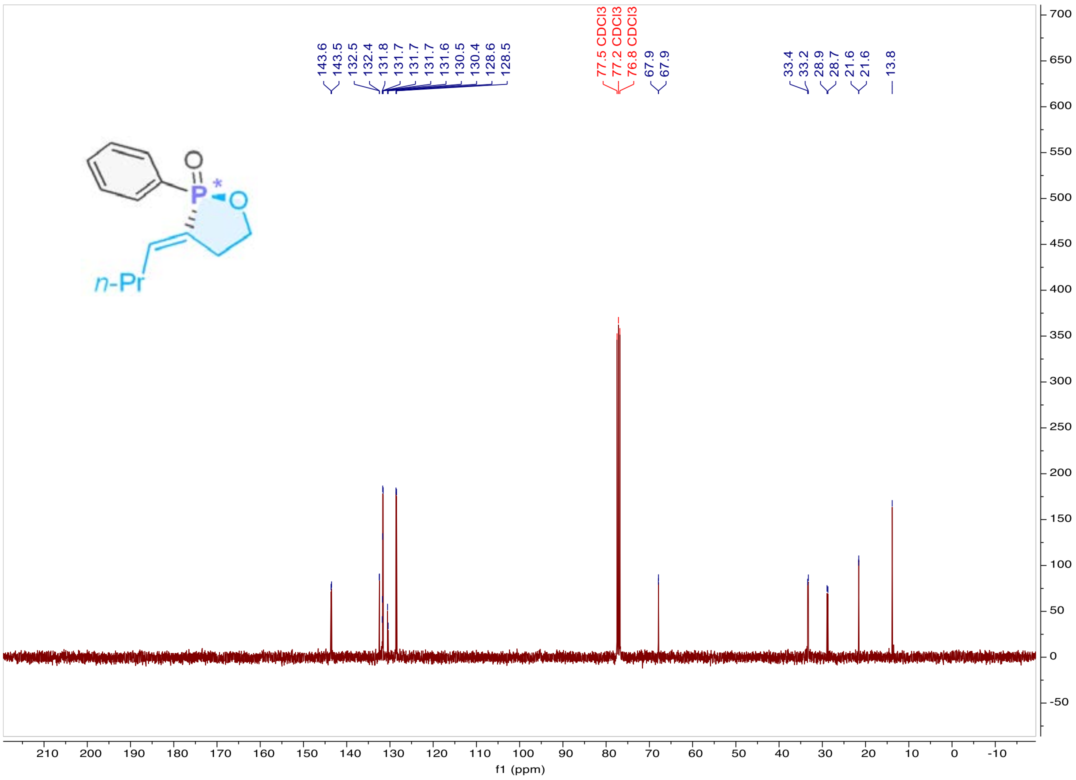

Figure S60. ^{1}H NMR, ^{31}P NMR and ^{13}C NMR spectra for **2r**

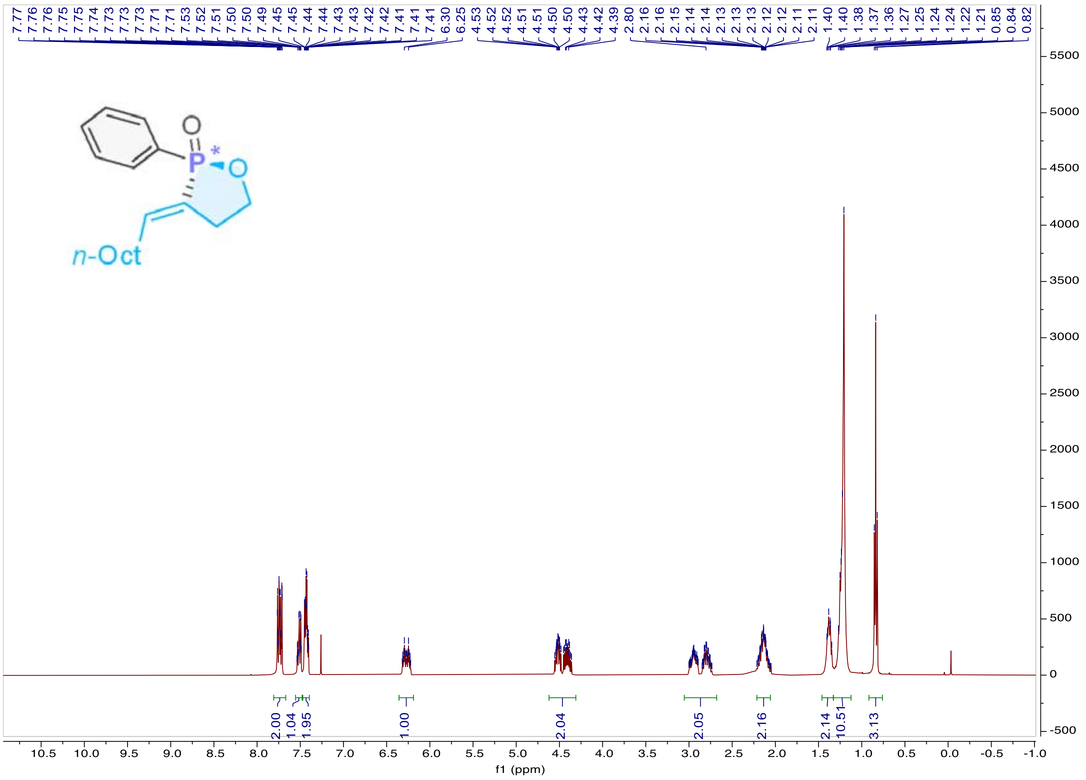
n-Oct
f1 (ppm)

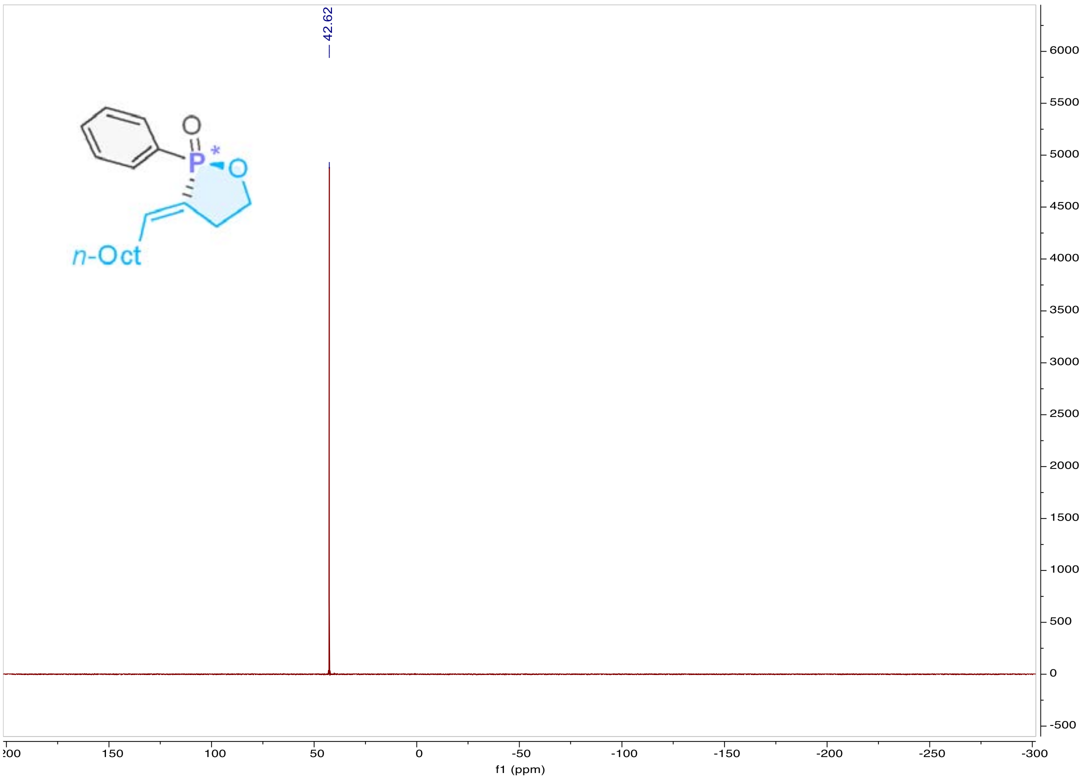
n-Oct
42.62
f1 (ppm)

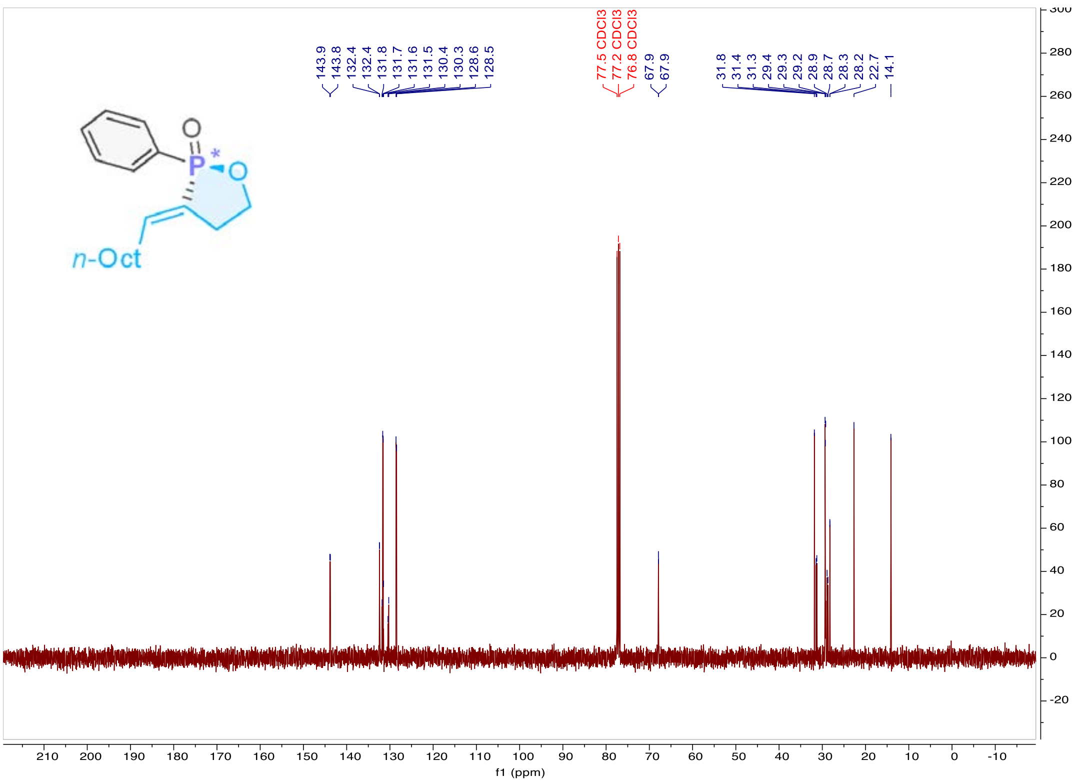

Figure S61. ^{1}H NMR, ^{31}P NMR and ^{13}C NMR spectra for **2s**

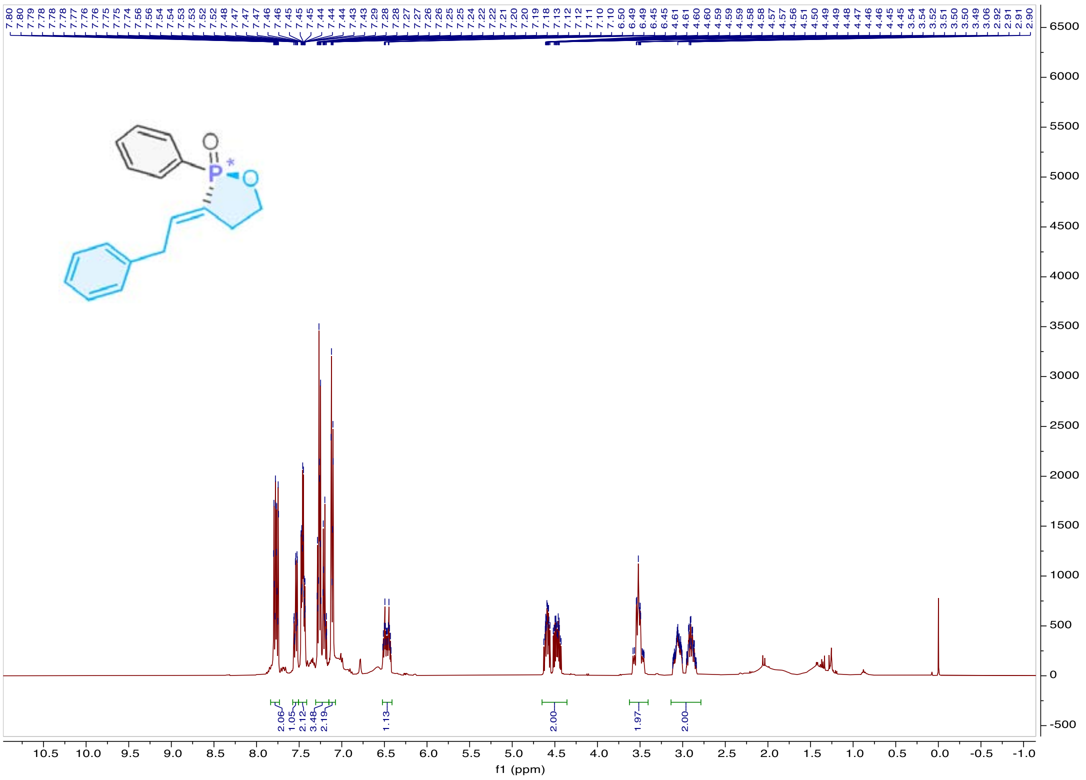
f1 (ppm)

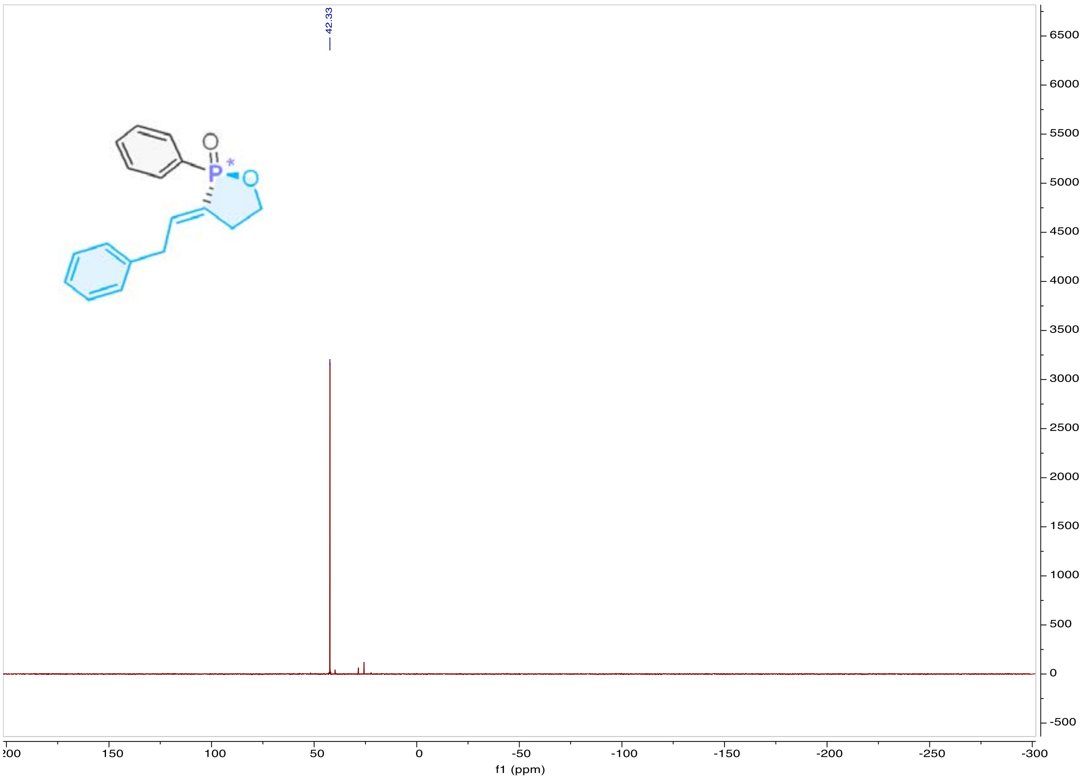
42.33
f1 (ppm)

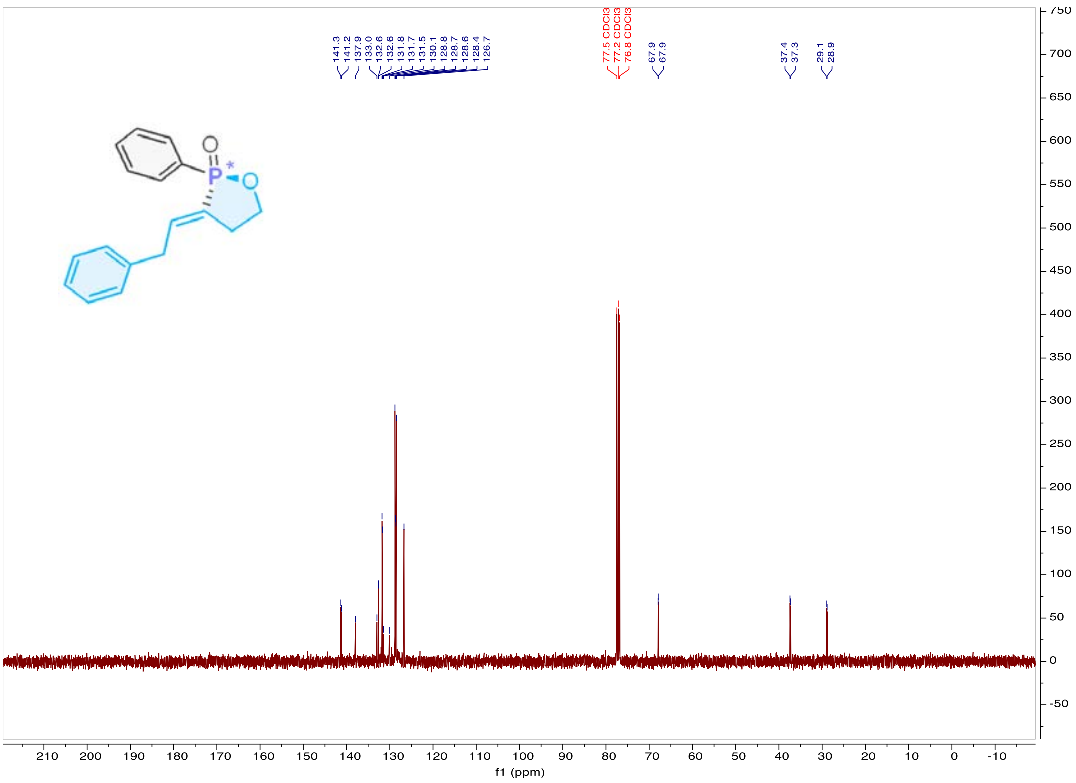

Figure S62. ^{1}H NMR, ^{31}P NMR and ^{13}C NMR spectra for **2t**

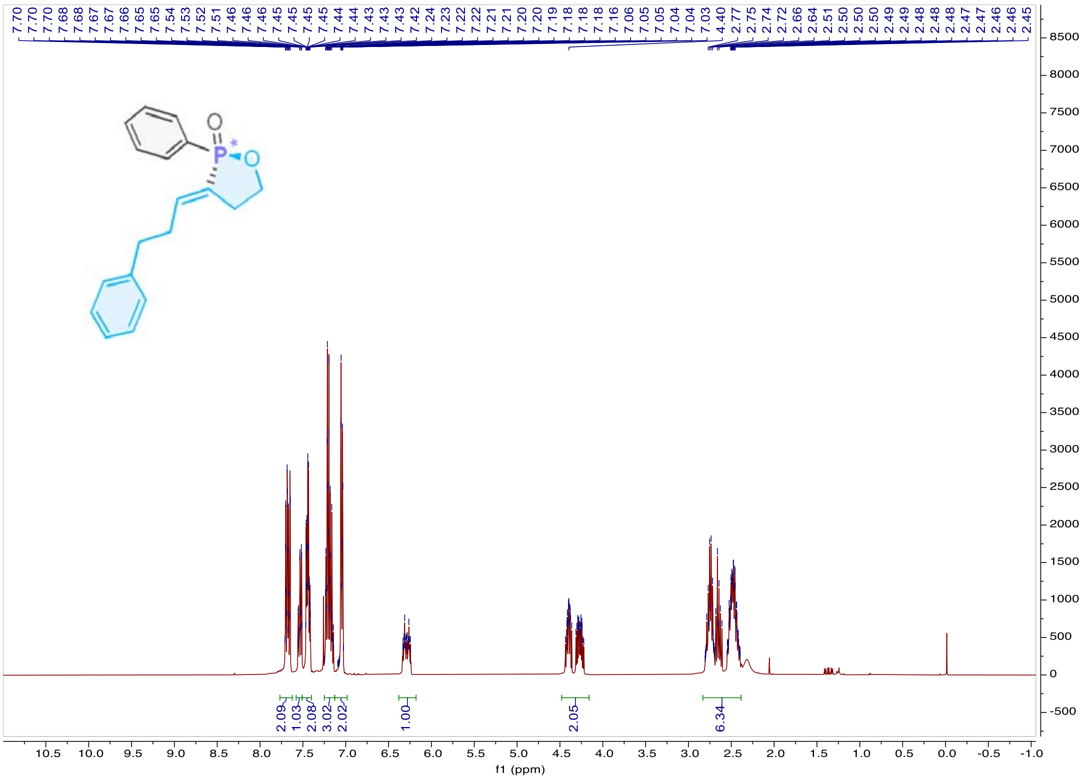

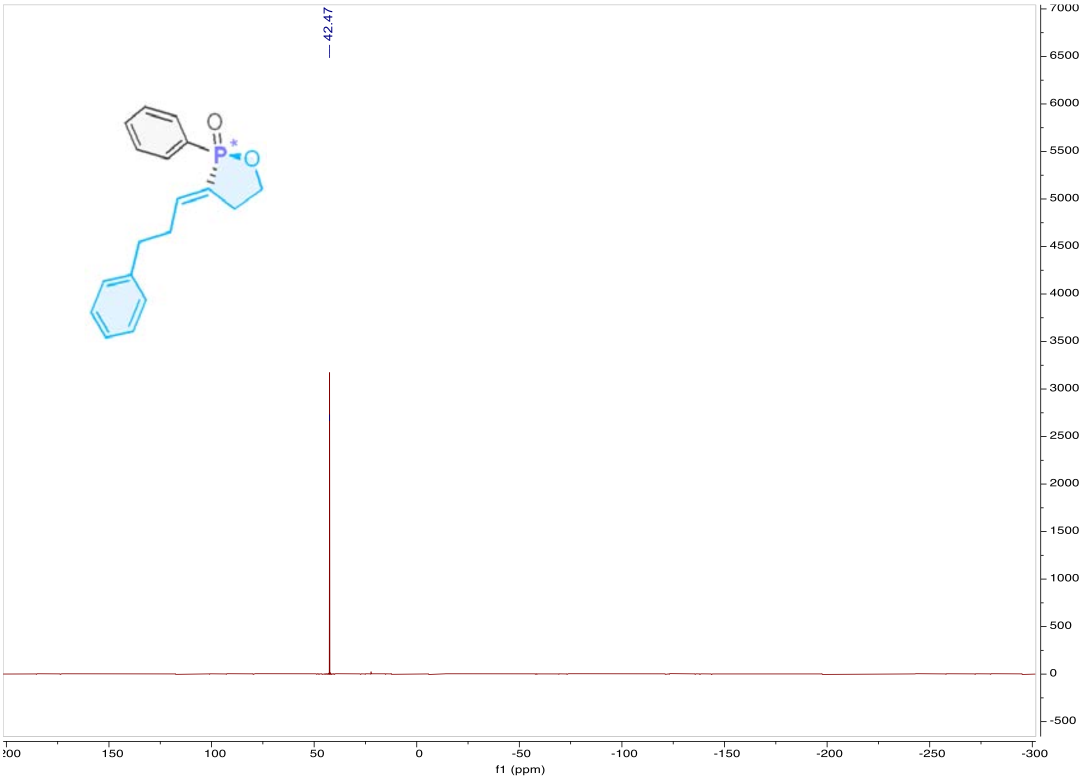

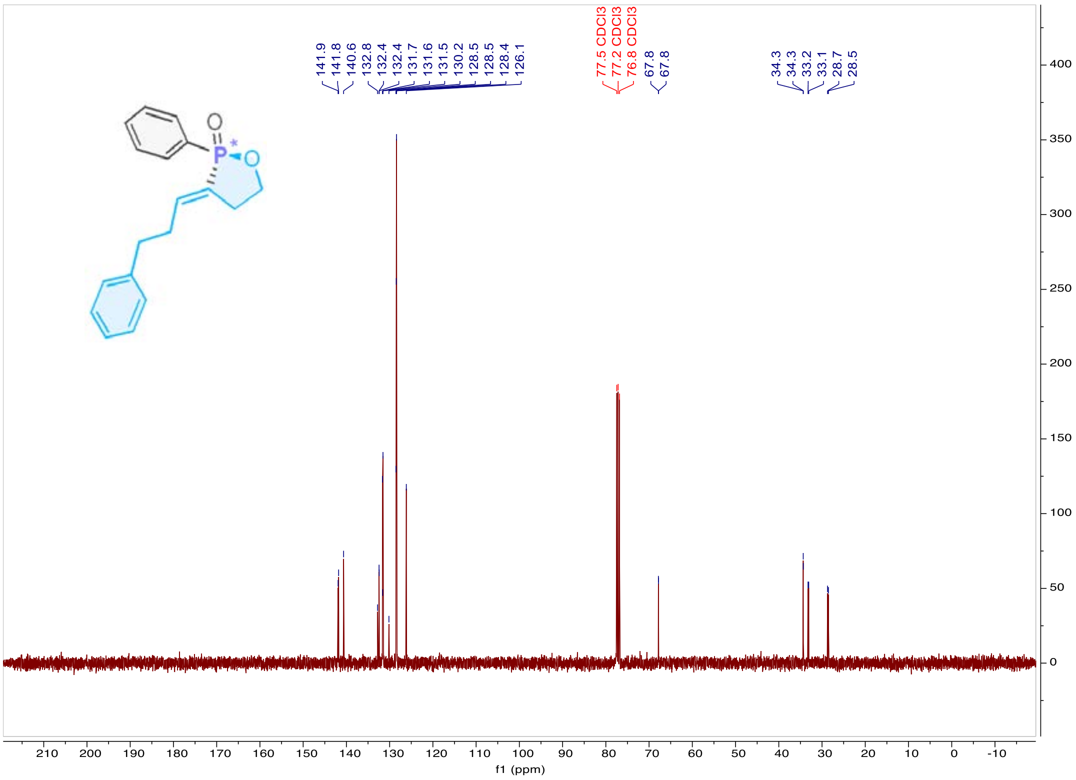

Figure S63. ^{1}H NMR, ^{31}P NMR and ^{13}C NMR spectra for **2u**

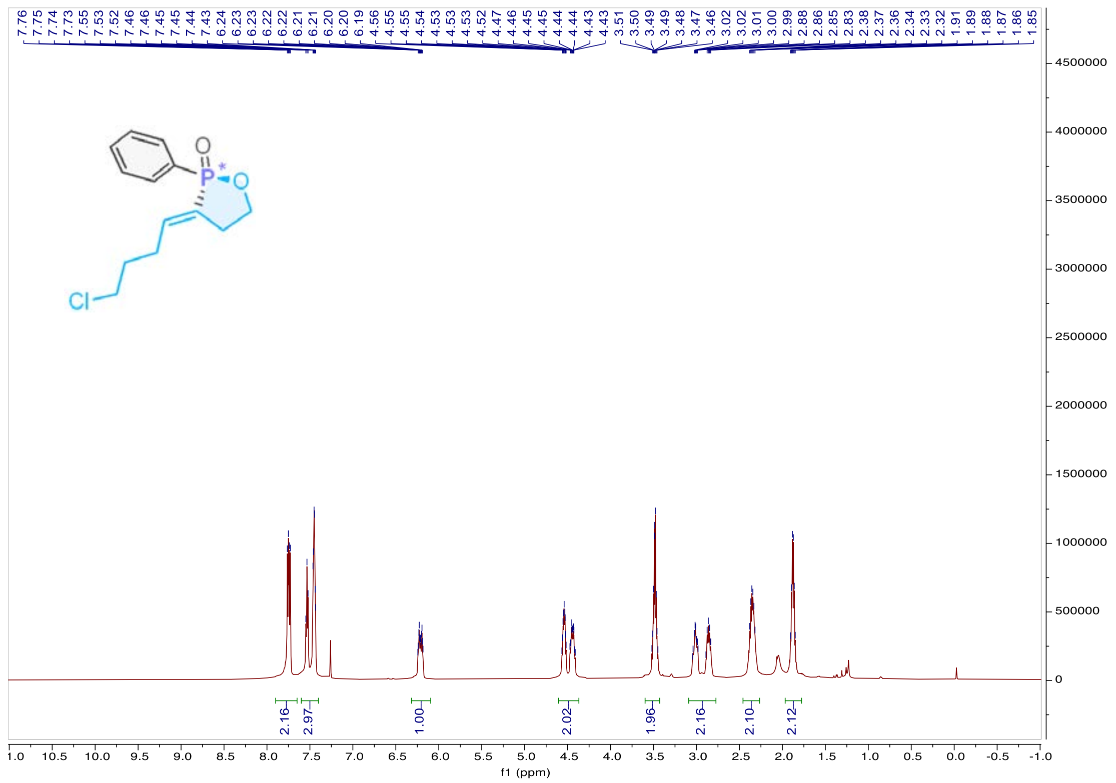

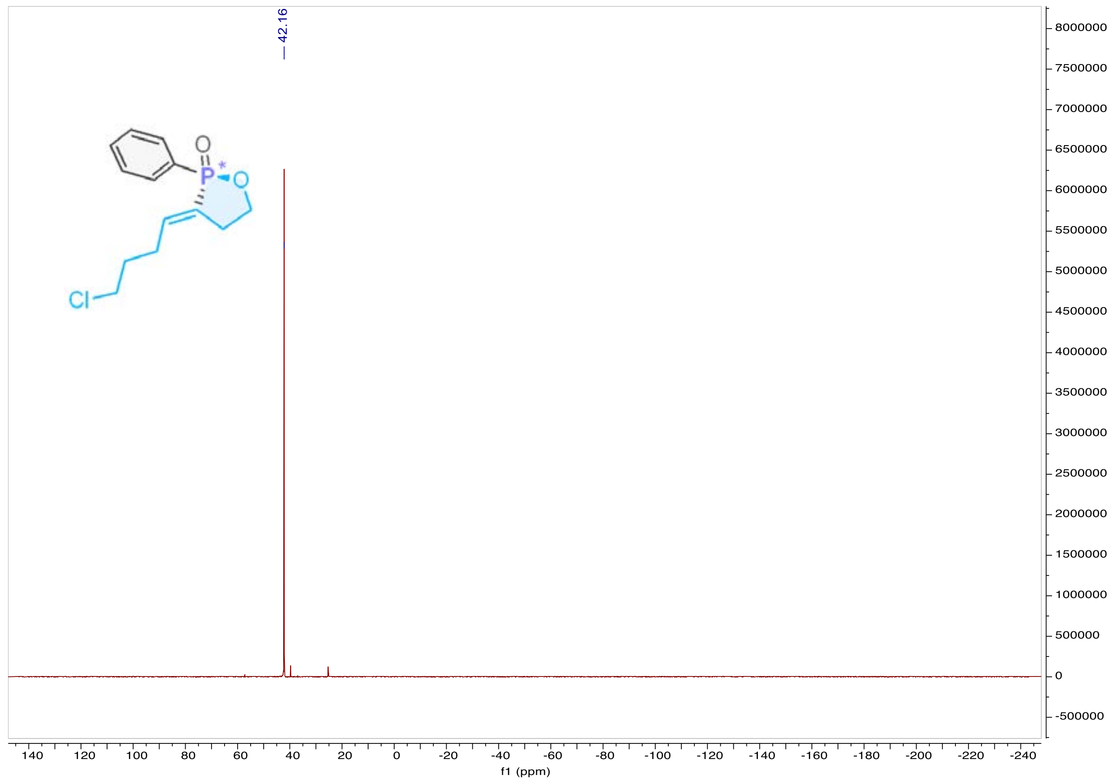

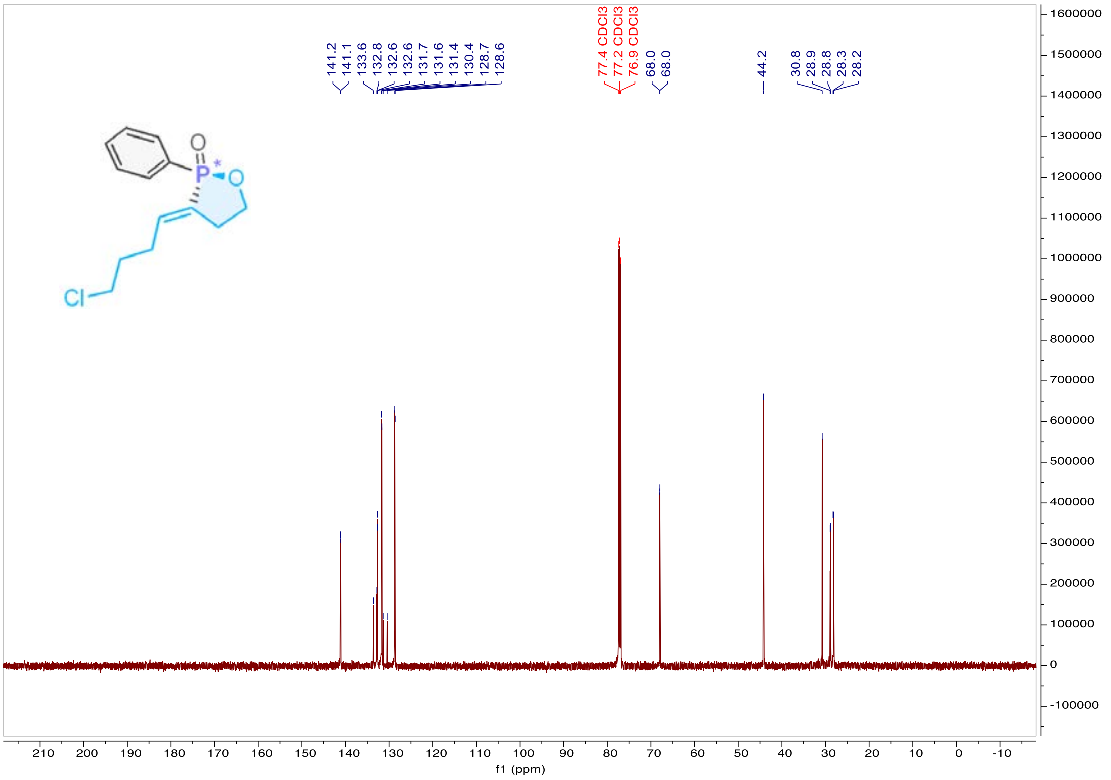

Figure S64. ^{1}H NMR, ^{31}P NMR and ^{13}C NMR spectra for **2v**

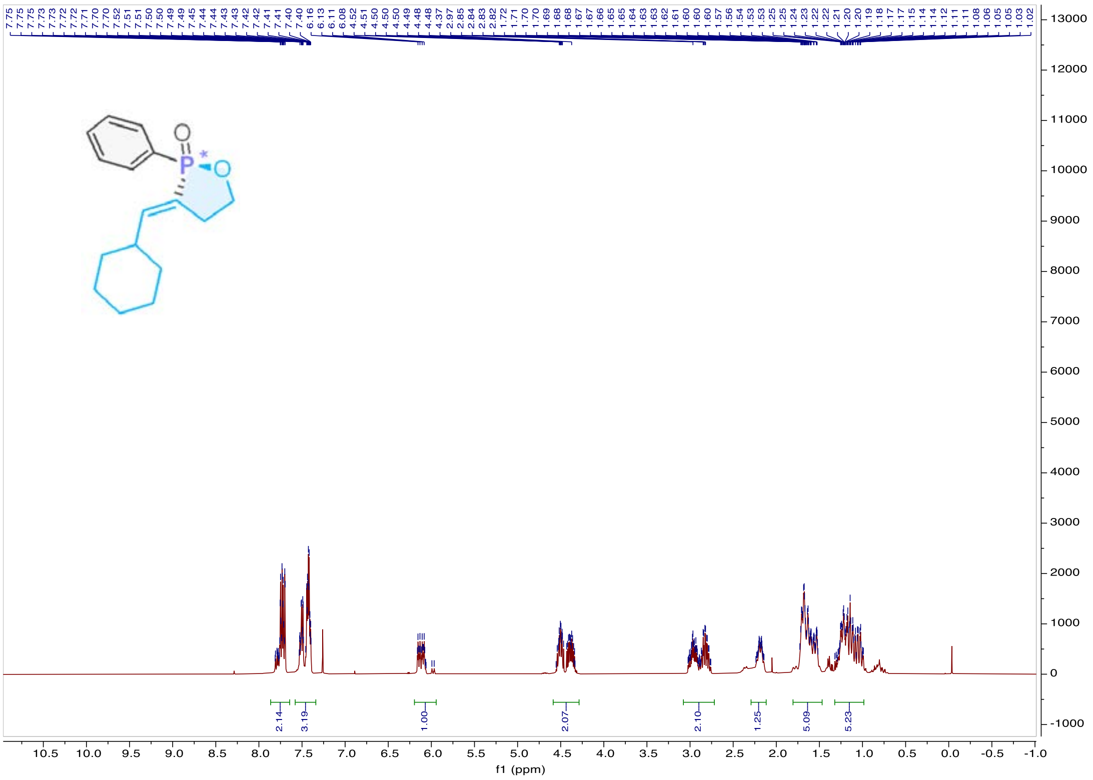

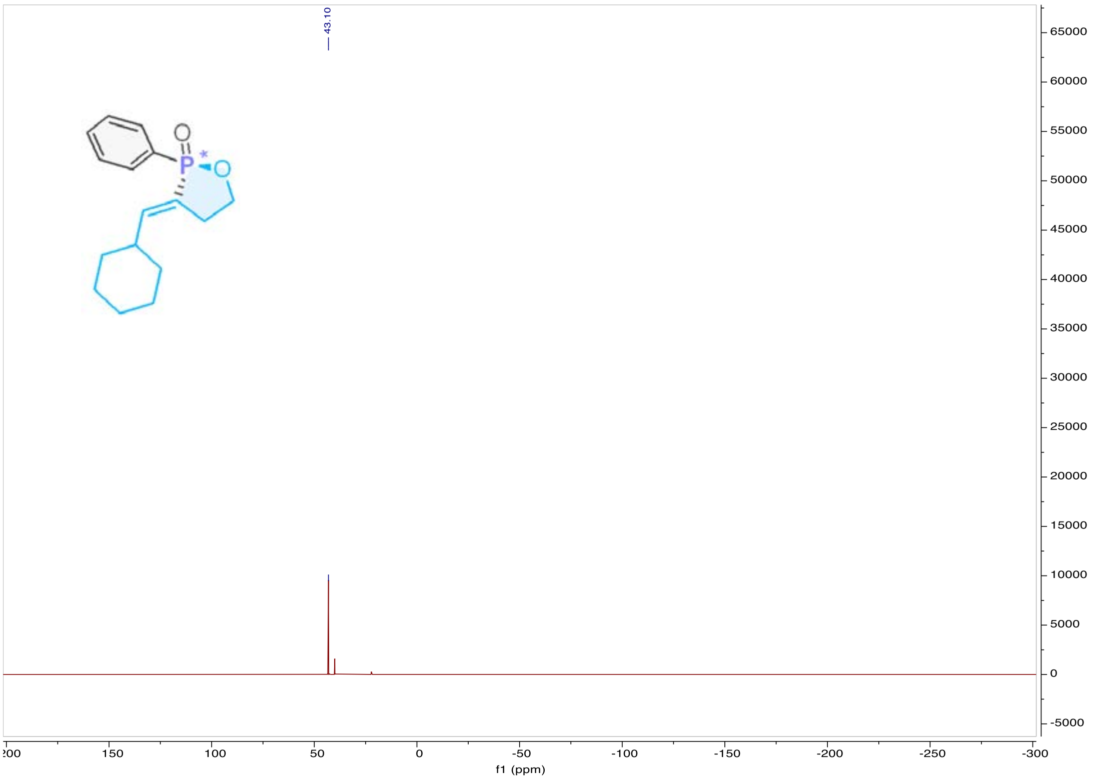

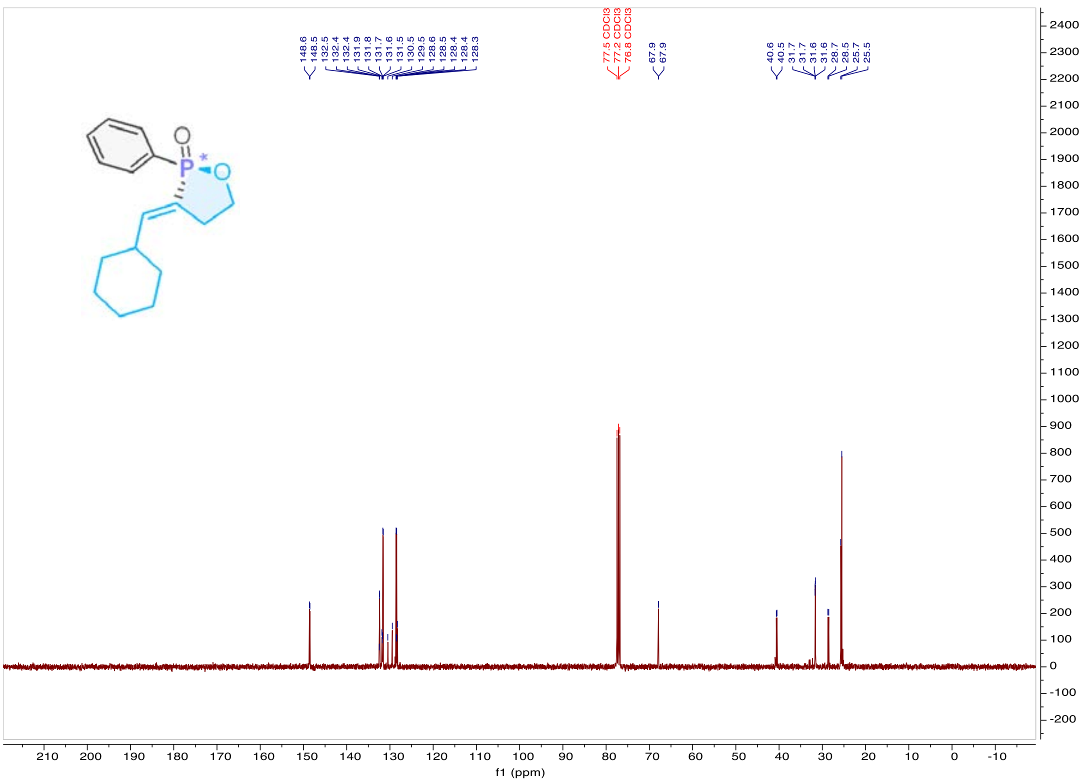

Figure S65. ^{1}H NMR, ^{31}P NMR and ^{13}C NMR spectra for **2w**

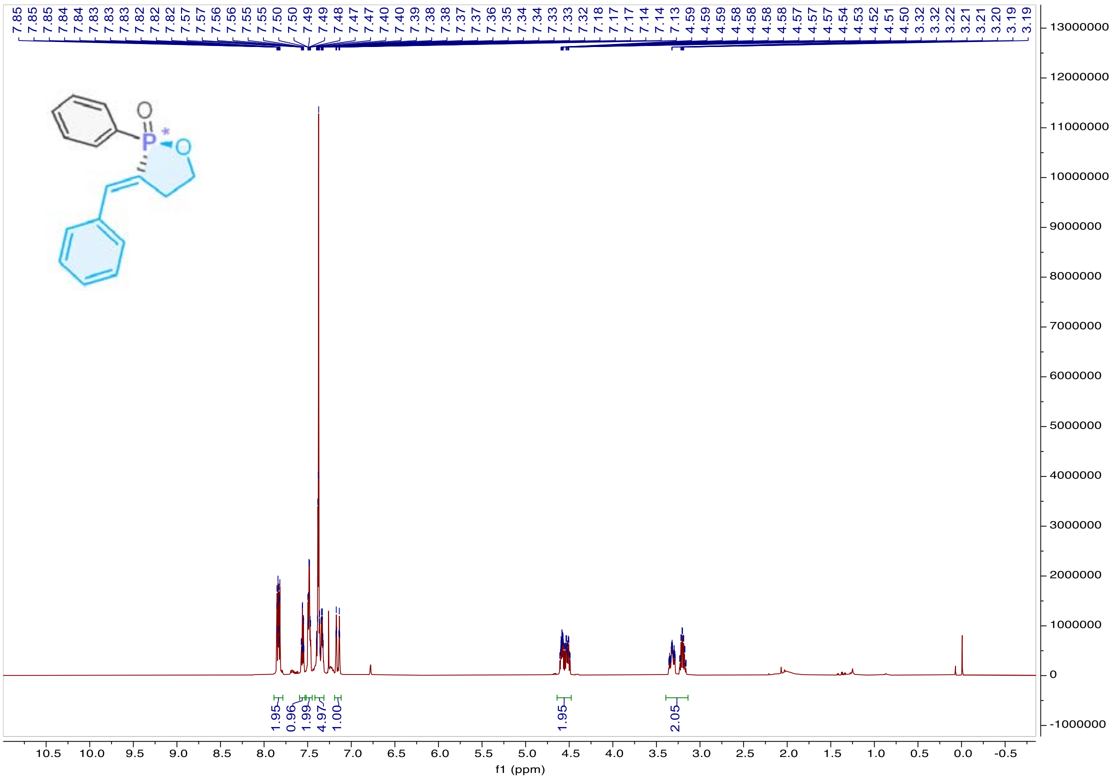

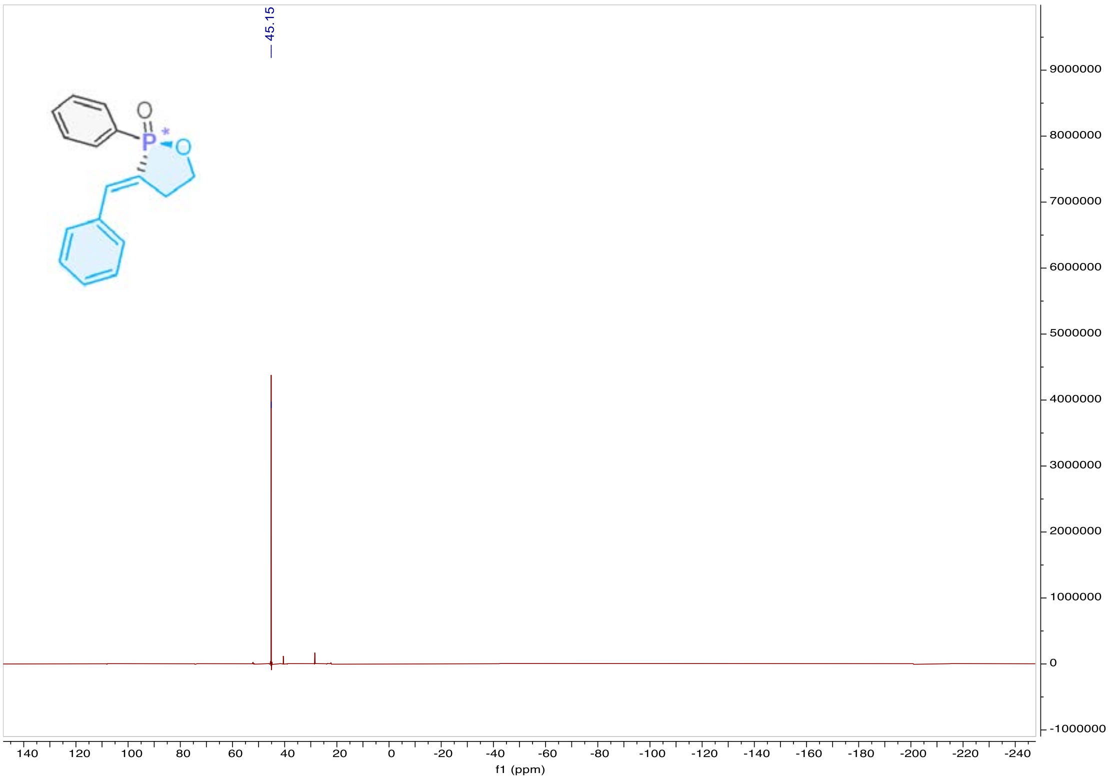

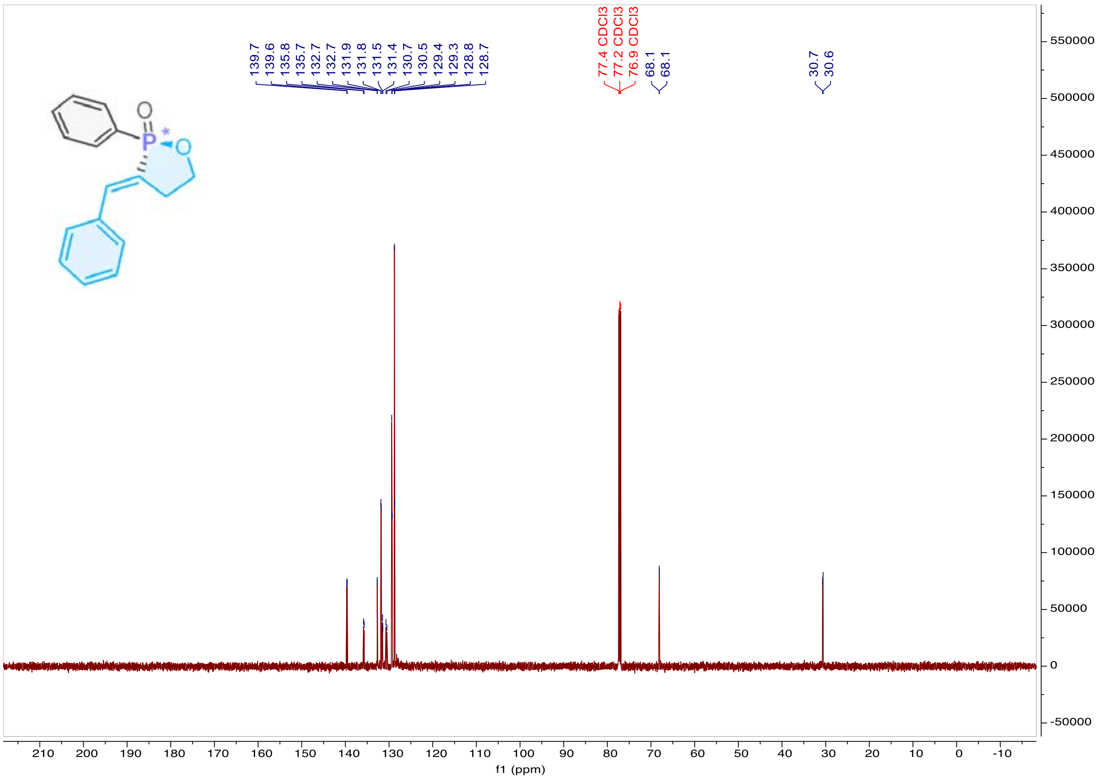

Figure S66. ^{1}H NMR, ^{31}P NMR and ^{13}C NMR spectra for **2x**

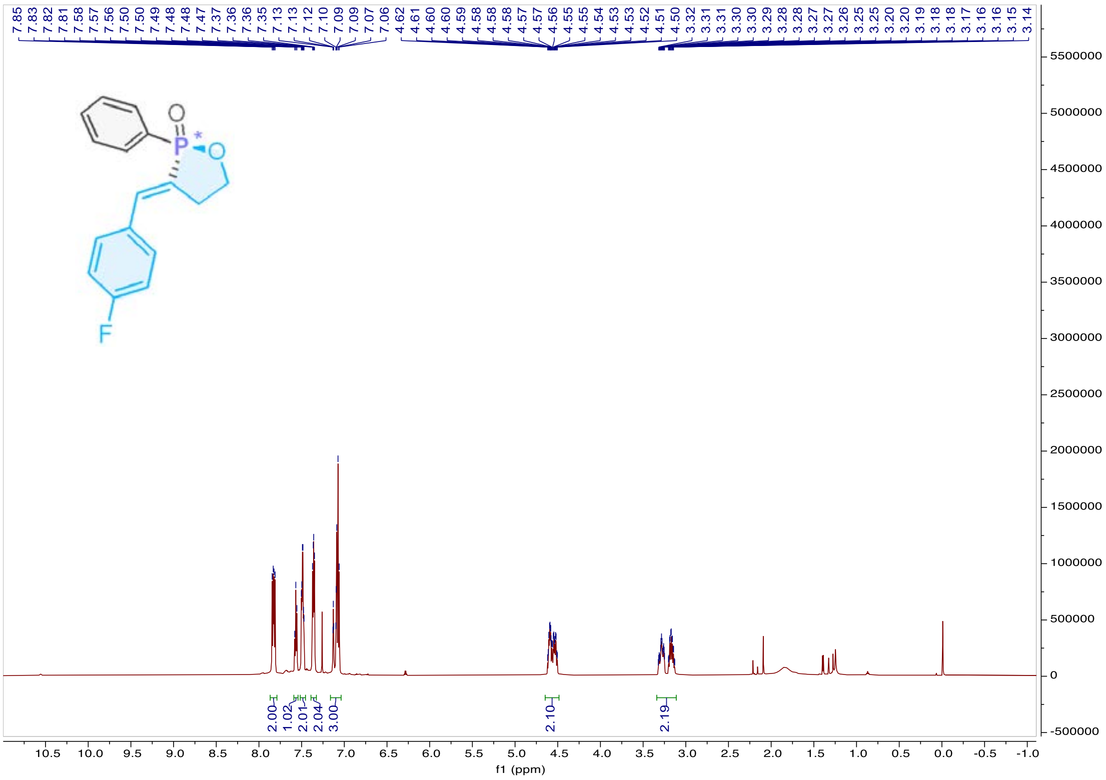

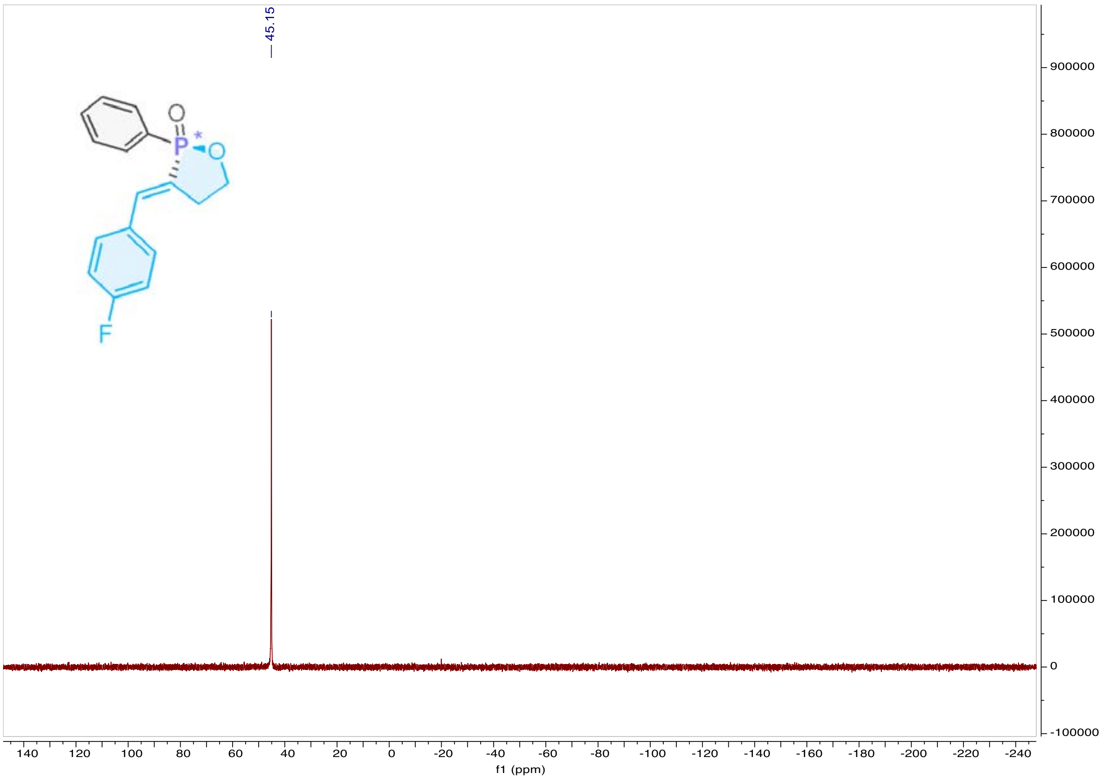

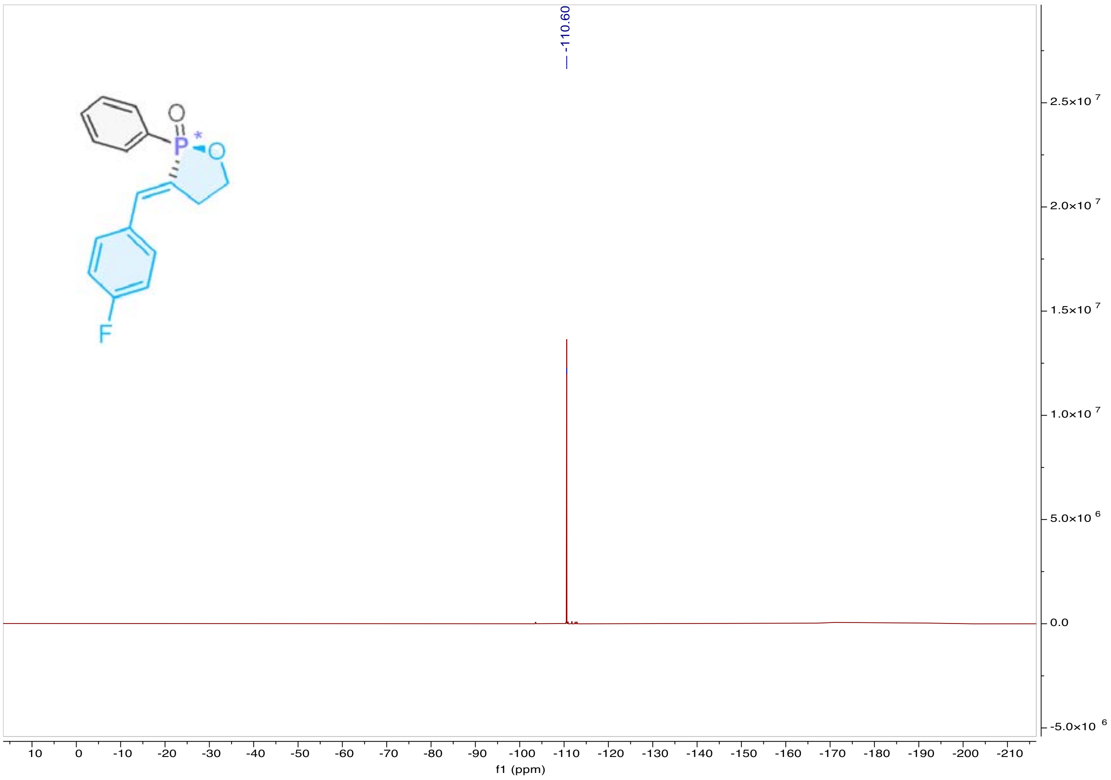

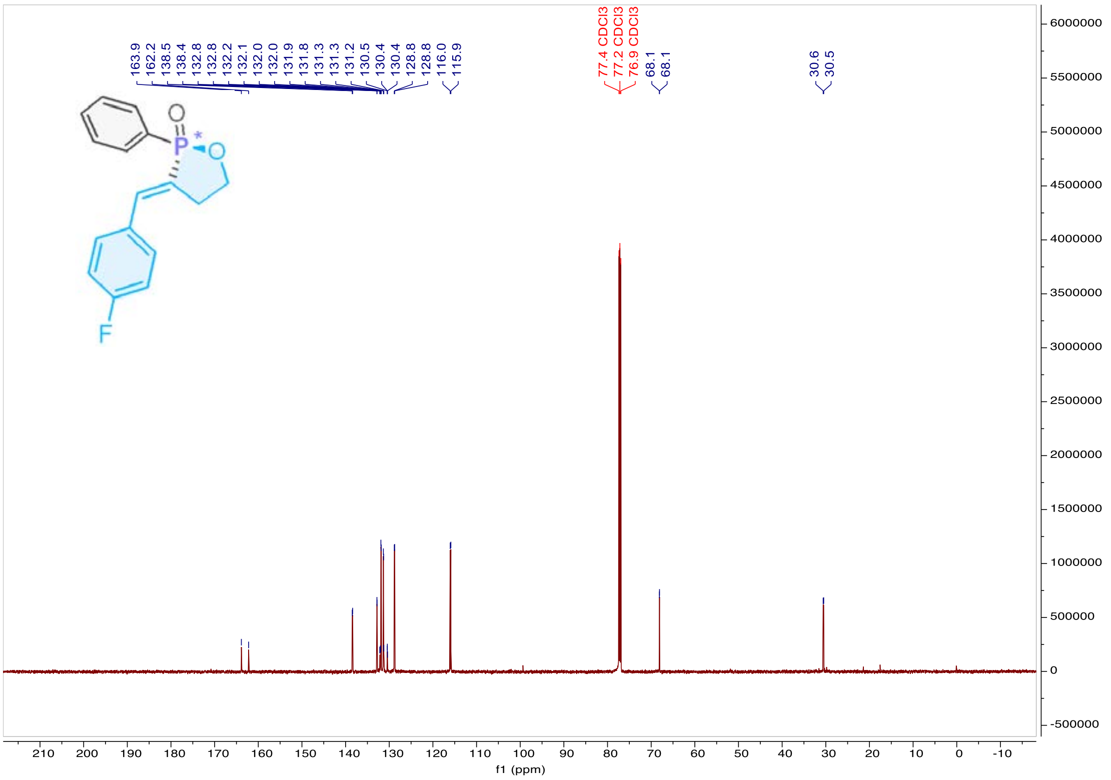

Figure S67. ^{1}H NMR, ^{19}F NMR, ^{31}P NMR and ^{13}C NMR spectra for **2y**

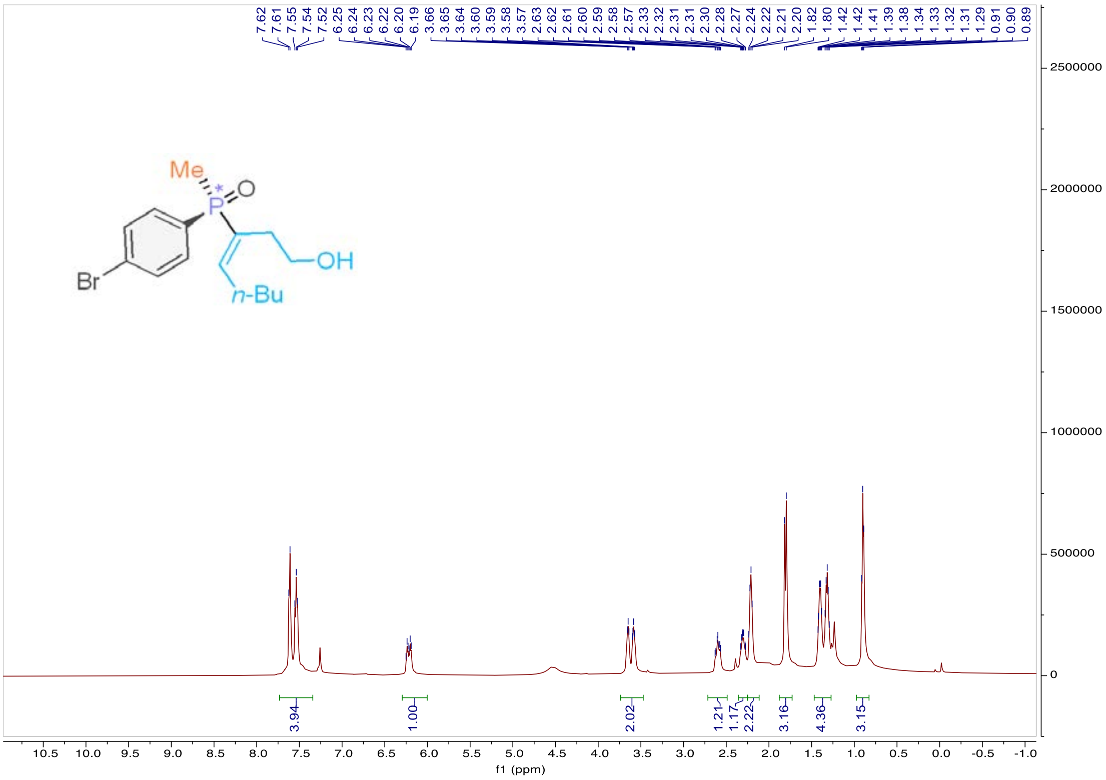
7.62
7.61
7.55
7.54
7.52
6.25
6.24
6.23
6.22
6.20
6.19
3.66
3.65
3.64
3.60
3.59
3.58
3.57
2.63
2.62
2.61
2.60
2.59
2.58
2.57
2.33
2.32
2.31
2.30
2.28
2.27
2.24
2.22
2.21
2.20
1.82
1.80
1.42
1.42
1.41
1.39
1.38
1.34
1.33
1.32
1.31
1.29
0.91
0.90
0.89
Me
P*
O
Br
OH
n-Bu
3.94
1.00
2.02
1.21
1.17
2.22
3.16
4.36
3.15
f1 (ppm)

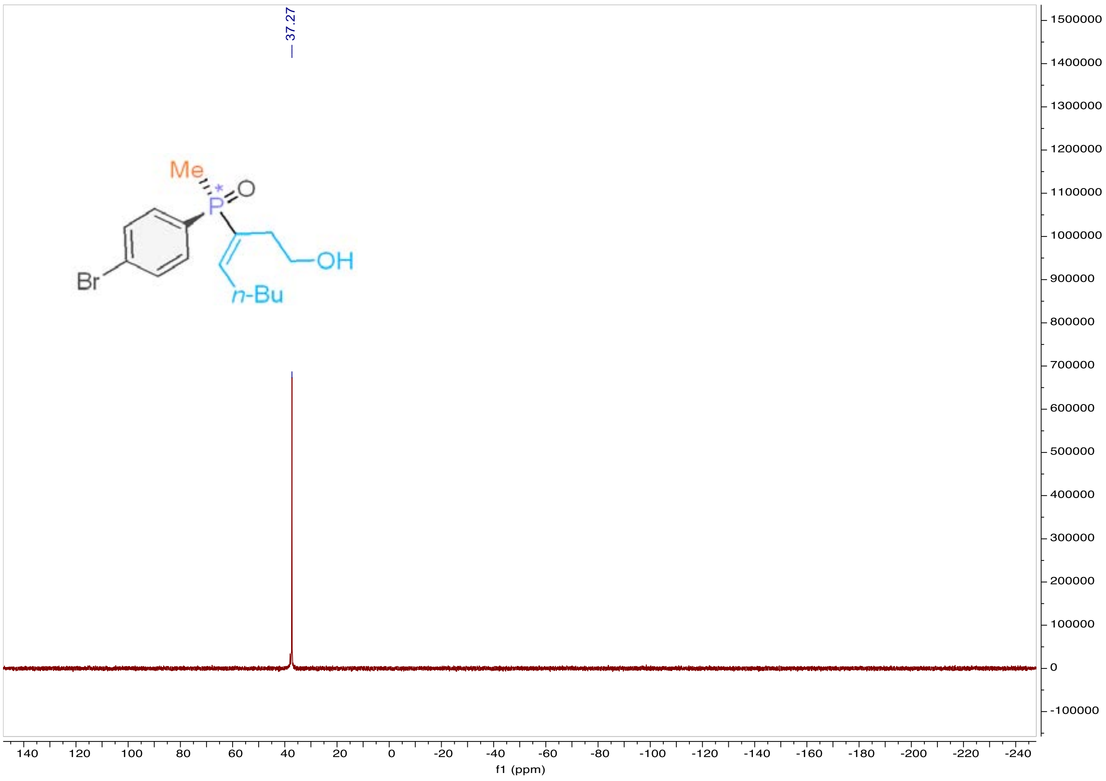
37.27
Me
P*
O
Br
OH
n-Bu
f1 (ppm)

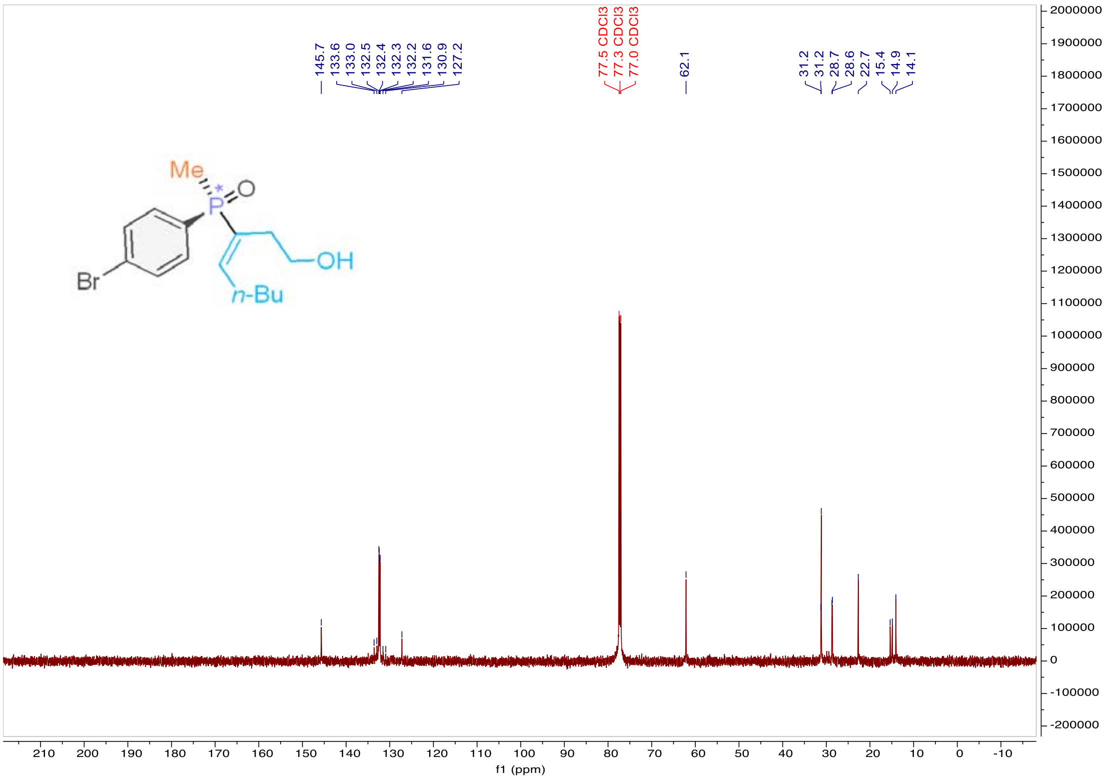

Figure S68. ^{1}H NMR, ^{31}P NMR and ^{13}C NMR spectra for **3**

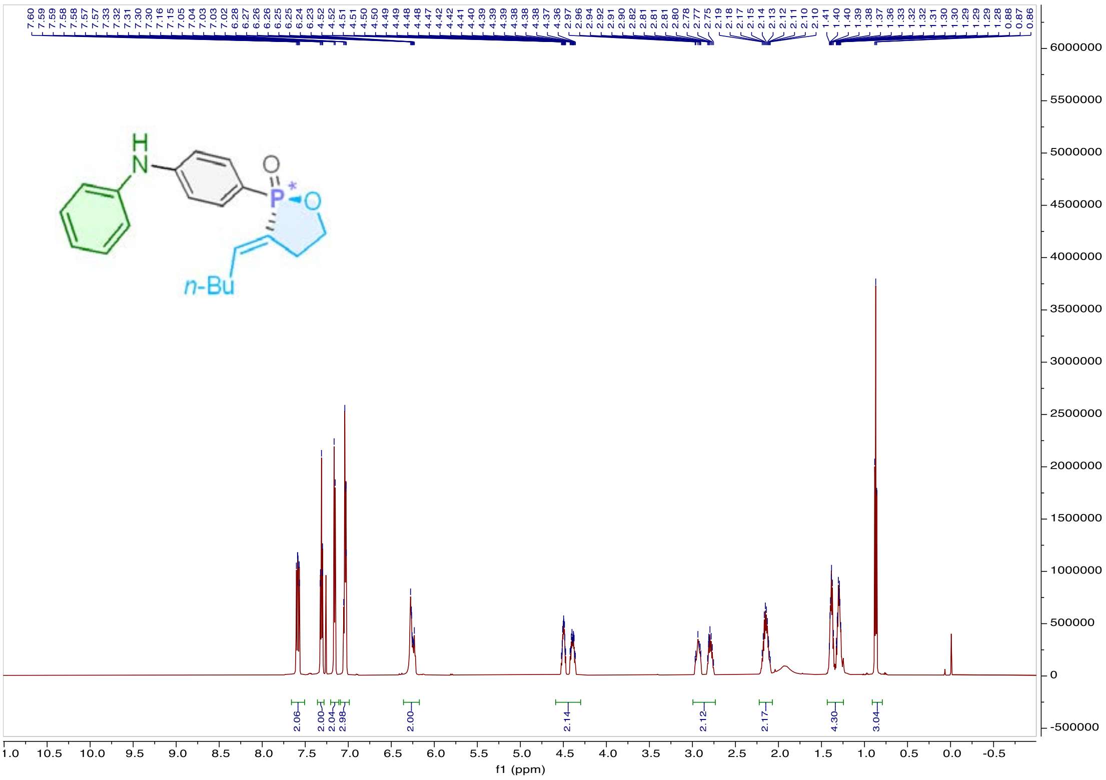

f1 (ppm)
n-Bu

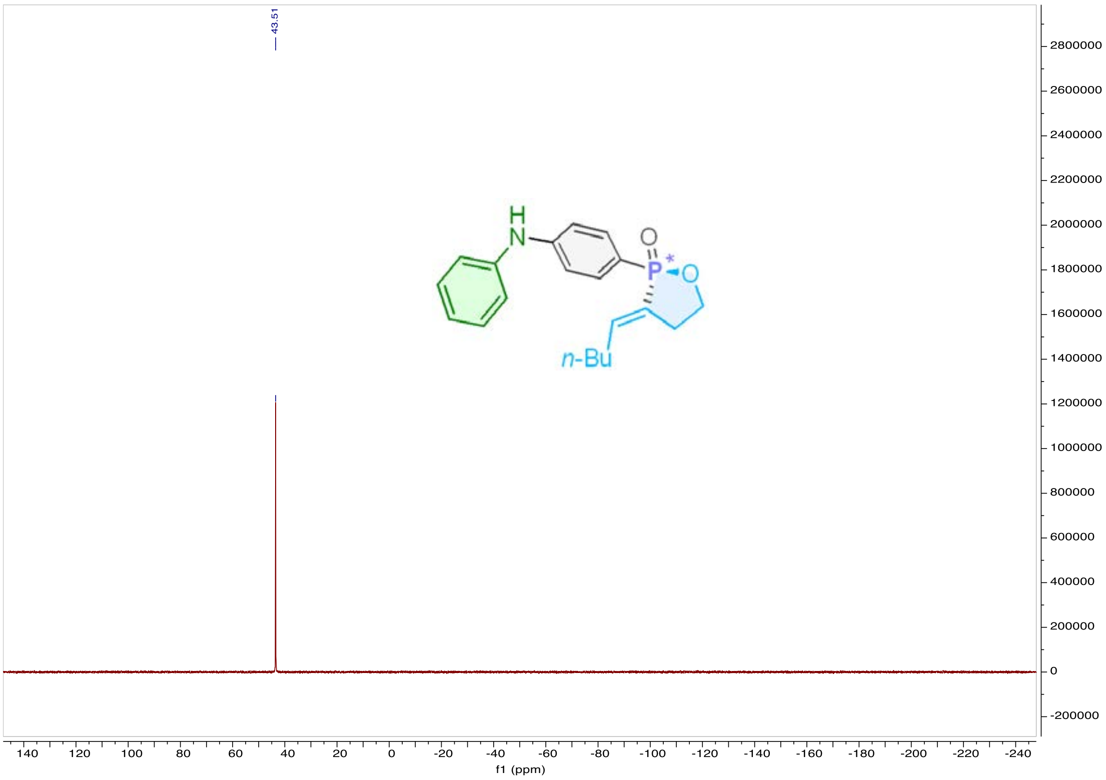

43.51
f1 (ppm)
n-Bu

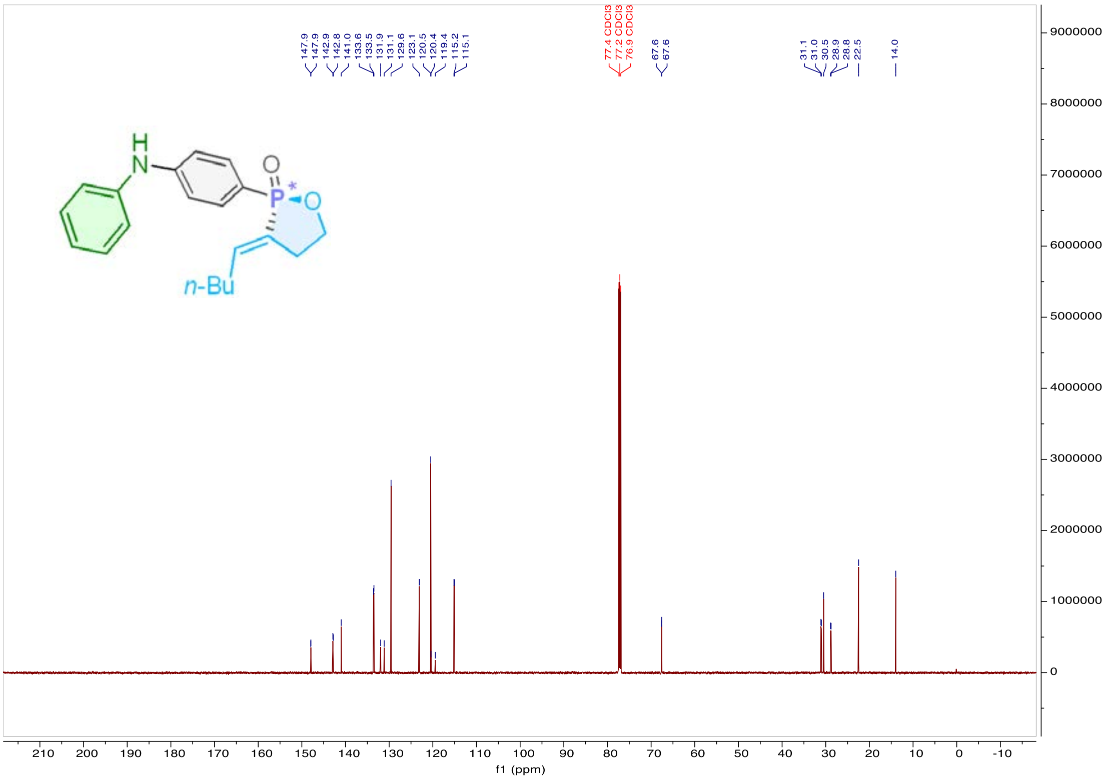

Figure S69. ^{1}H NMR, ^{31}P NMR and ^{13}C NMR spectra for **5**

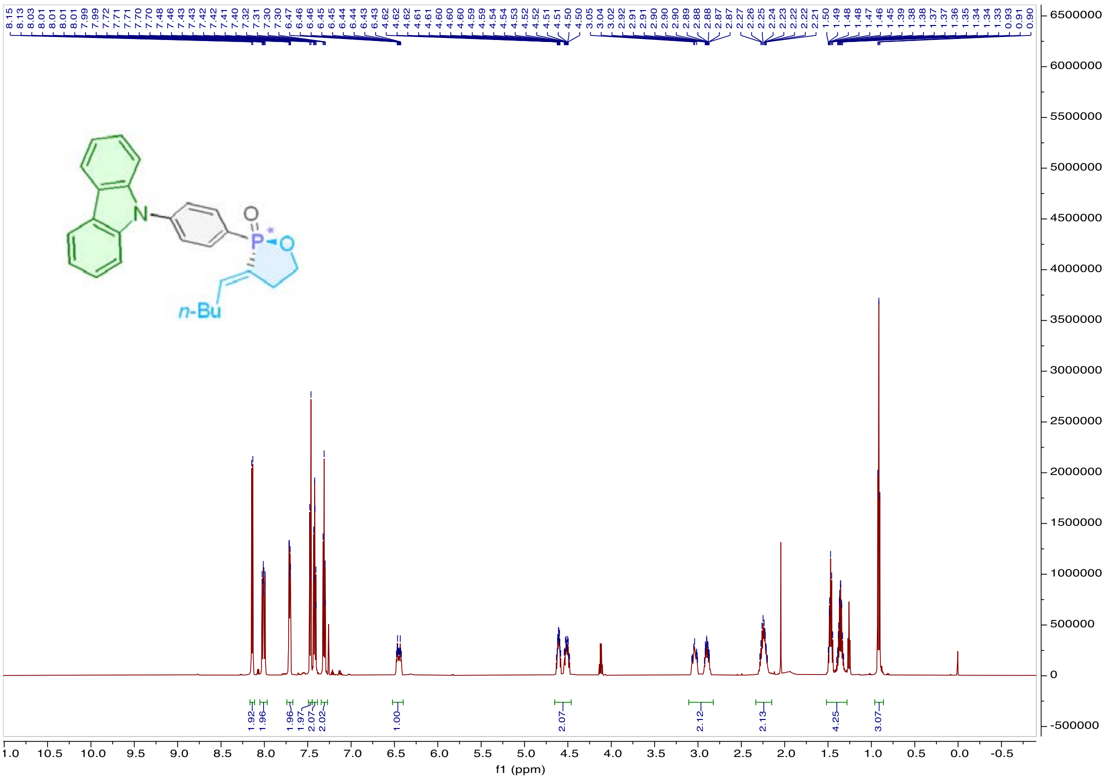

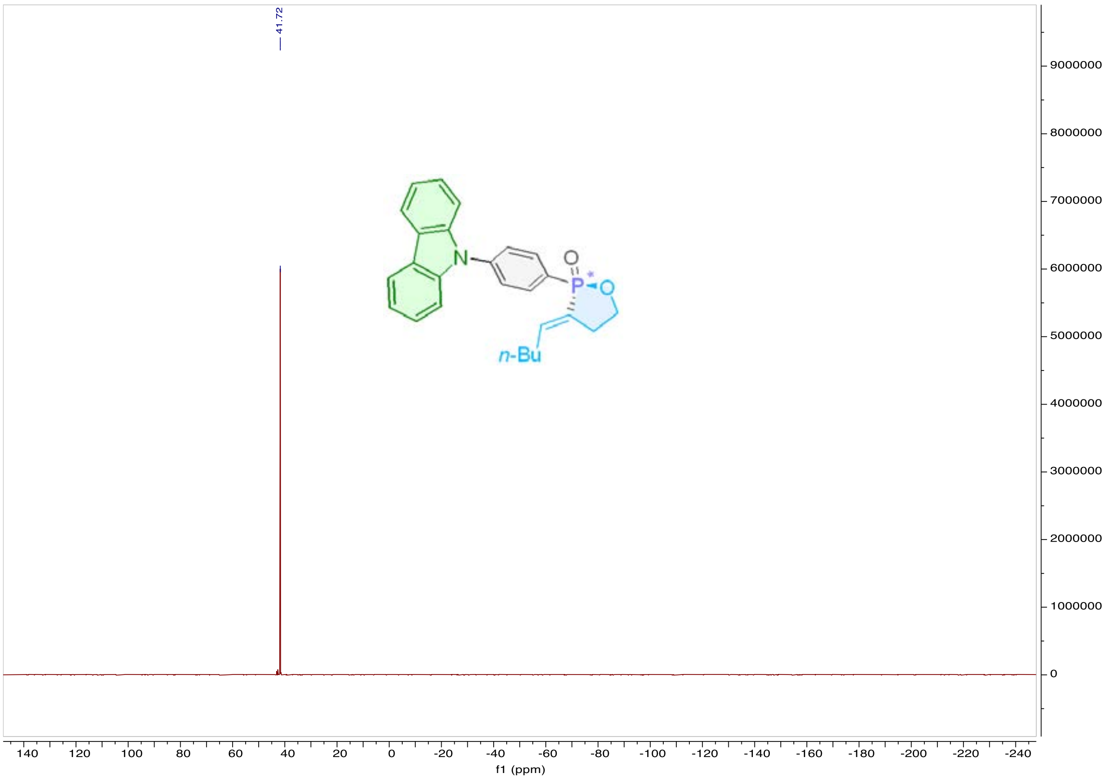

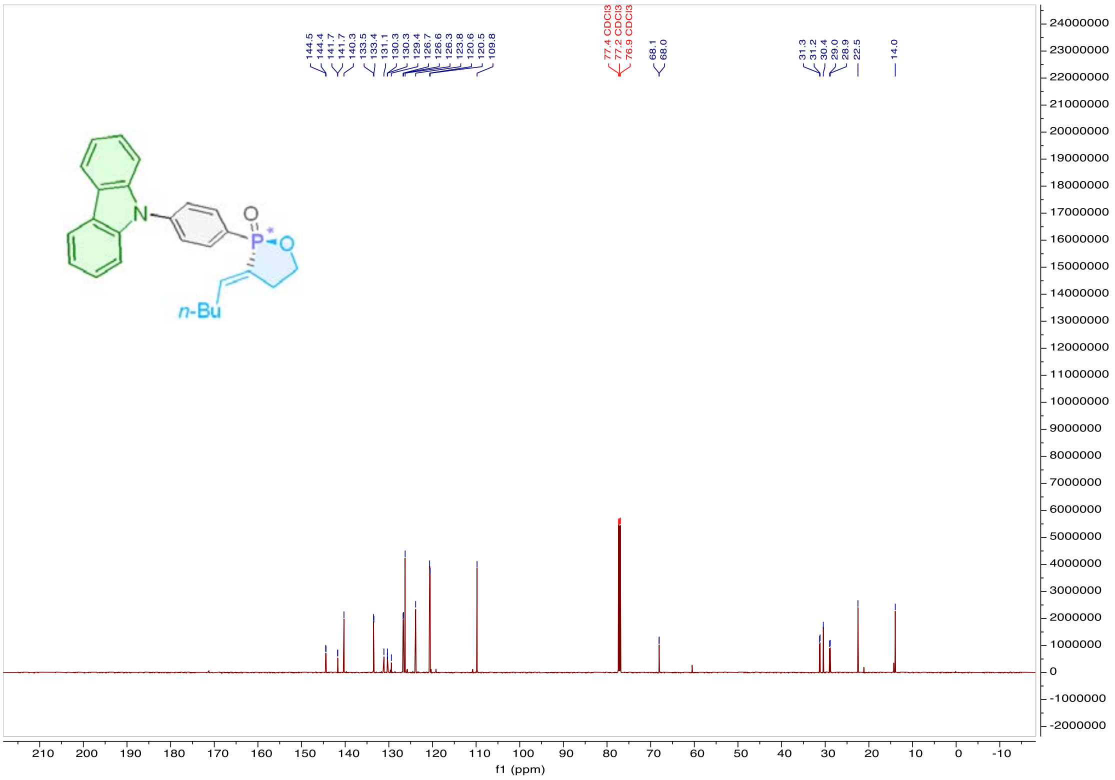

Figure S70. ^{1}H NMR, ^{31}P NMR and ^{13}C NMR spectra for **6**

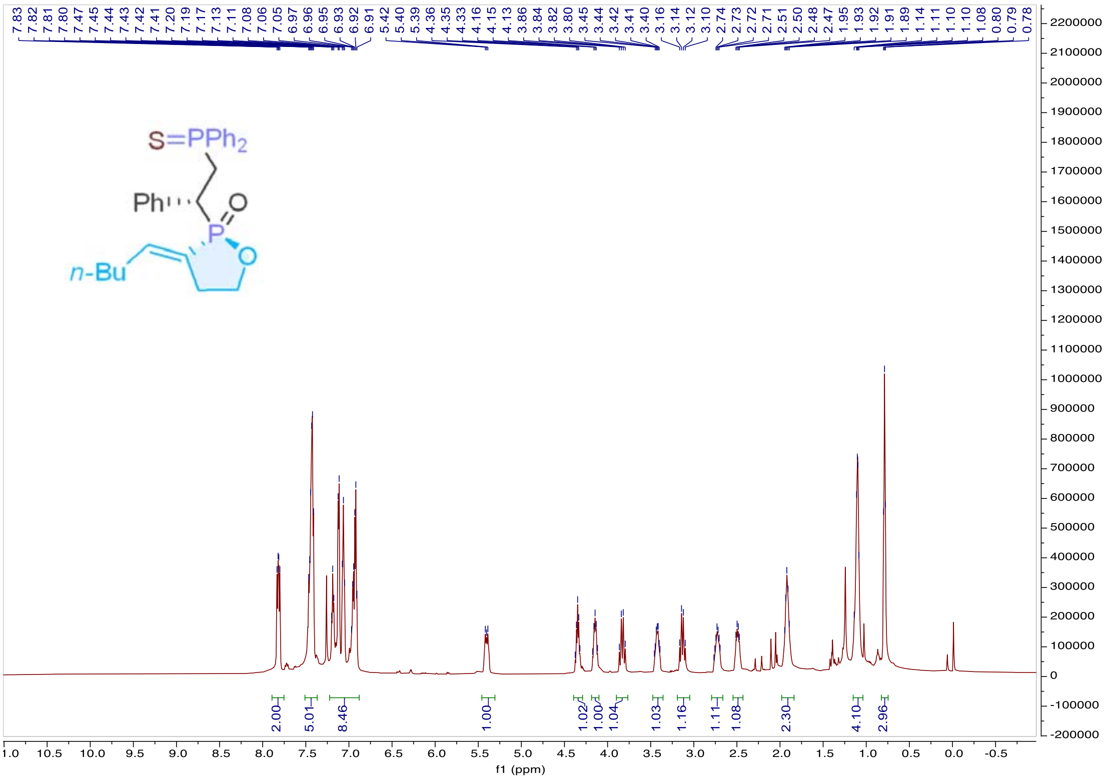

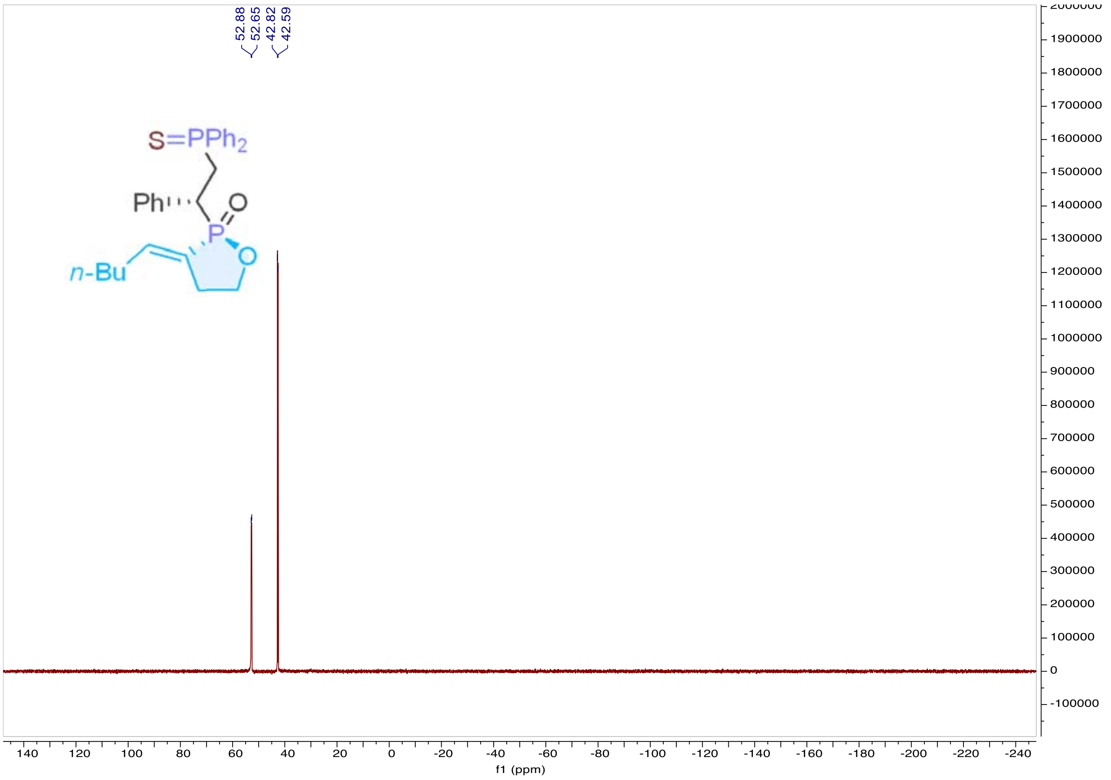

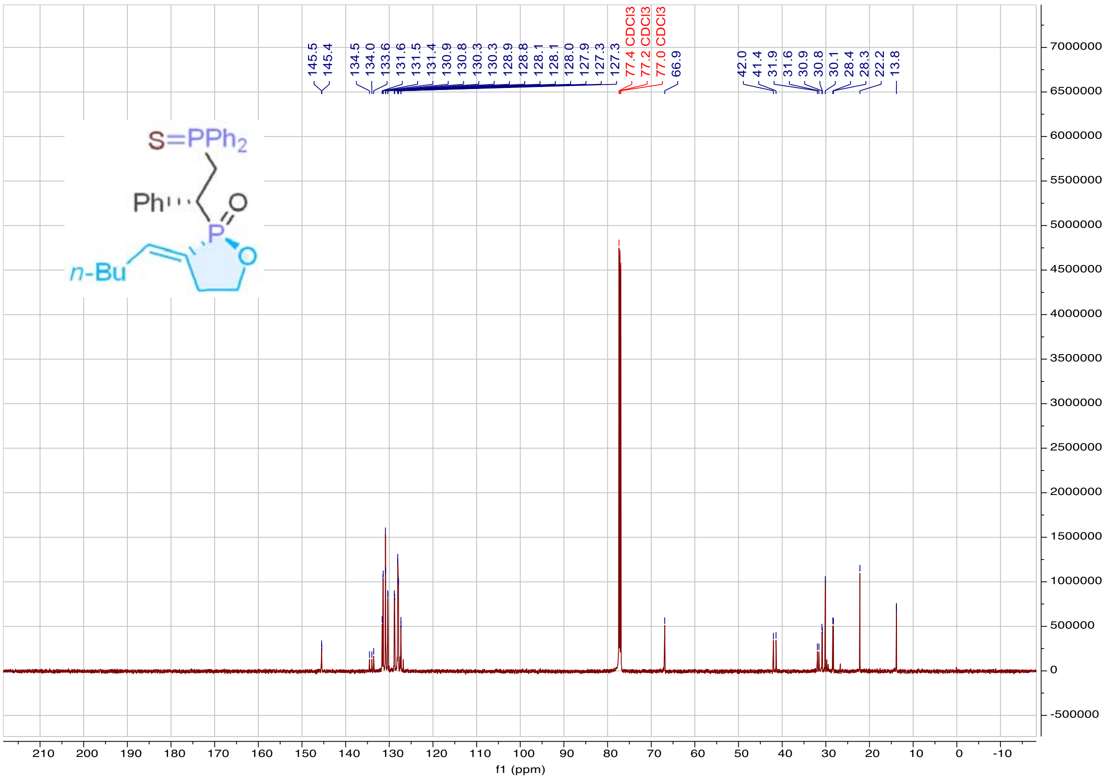

Figure S71. ^{1}H NMR, ^{31}P NMR and ^{13}C NMR spectra for **7a**

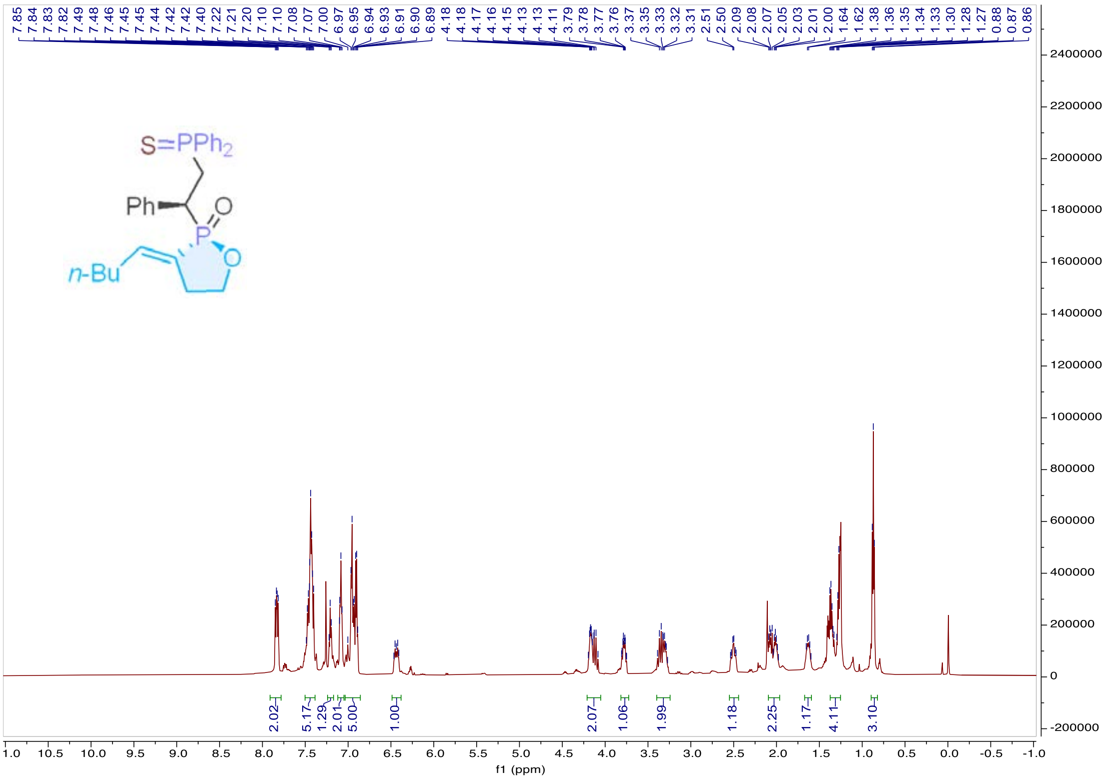
S=PPh2
Ph
O
P
O
n-Bu
f1 (ppm)

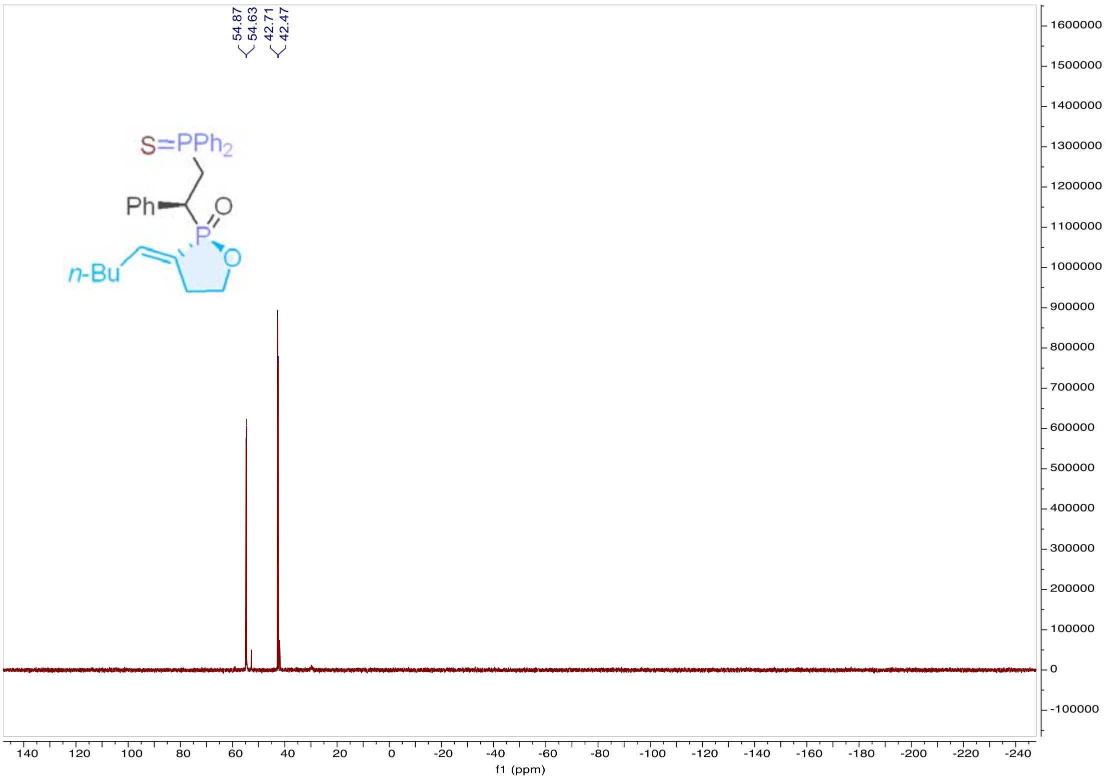
54.87
54.63
42.71
42.47
S=PPh2
Ph
O
P
O
n-Bu
f1 (ppm)

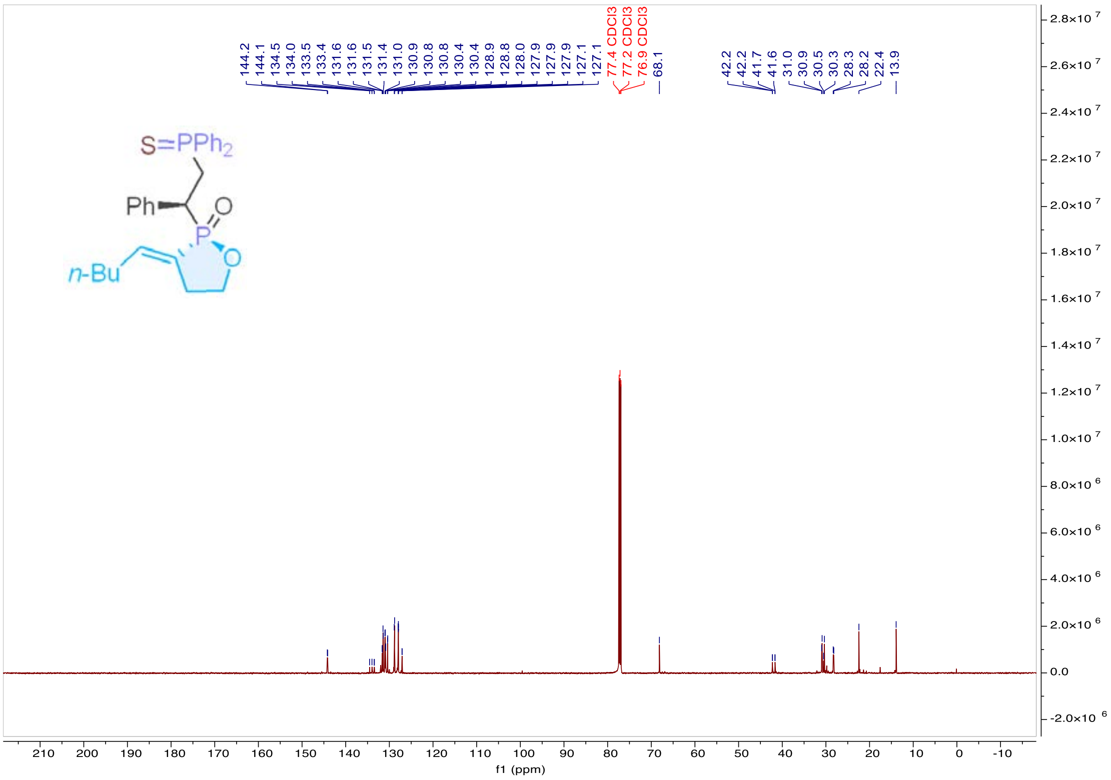

Figure S72. ^{1}H NMR, ^{31}P NMR and ^{13}C NMR spectra for **7b**

10. HPLC spectrum

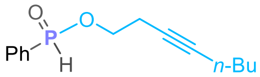

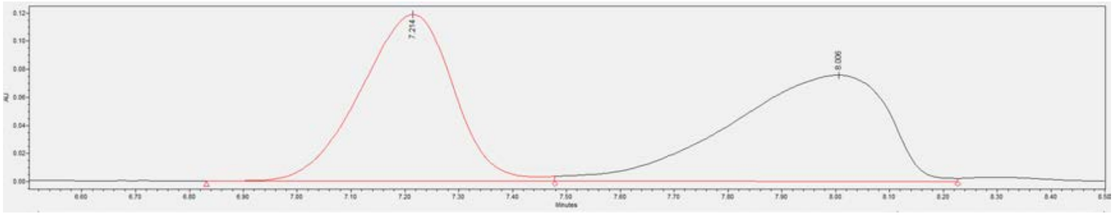

	Name	Retention Time (min)	Area (μV*sec)	% Area	Height (μV)	Int Type	Amount	Units	Peak Type	Peak Codes
1		7.214	1424280	48.19	118518	Bv			Unknown	
2		8.006	1531096	51.81	75706	vV			Unknown	

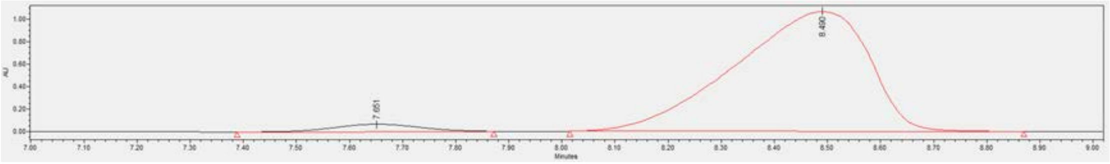

	Name	Retention Time (min)	Area (μV*sec)	% Area	Height (μV)	Int Type	Amount	Units	Peak Type	Peak Codes
1		7.651	728005	3.77	65921	bb			Unknown	
2		8.490	18598078	96.23	1063535	bb			Unknown	

Figure S73. HPLC spectrum for **1a**

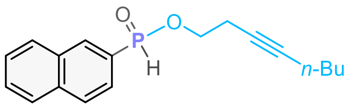

	Name	Retention Time (min)	Area (µV*sec)	% Area	Height (µV)	Int Type	Amount	Units	Peak Type	Peak Codes
1		9.948	44648341	49.40	2210004	VV			Unknown	
2		12.793	45737211	50.60	1906407	Vb			Unknown	

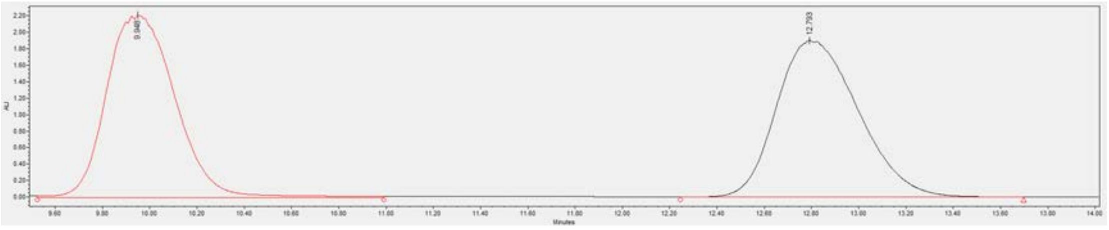

	Name	Retention Time (min)	Area (µV*sec)	% Area	Height (µV)	Int Type	Amount	Units	Peak Type	Peak Codes
1		10.045	4129629	7.04	210827	bb			Unknown	
2		12.772	54521655	92.96	2057909	bb			Unknown	

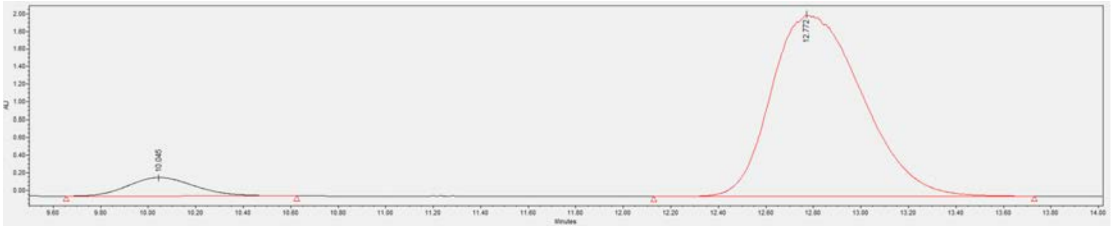

Figure S74. HPLC spectrum for **1b**

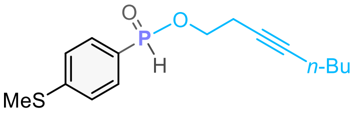

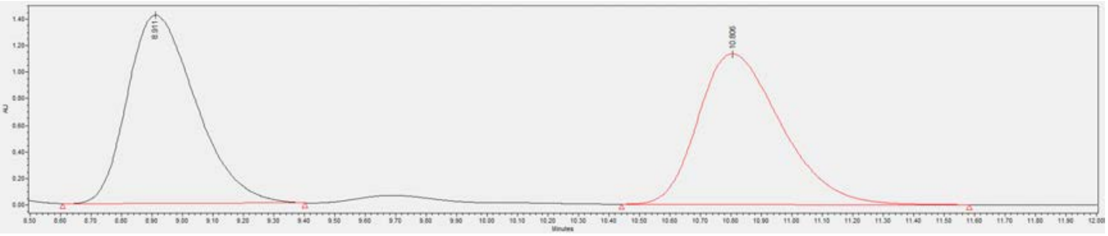

E	Name	Retention Time (min)	Area (µV*sec)	% Area	Height (µV)	Int Type	Amount	Units	Peak Type	Peak Codes
1		8.911	22152247	50.78	1416955	bb			Unknown	
2		10.806	21471558	49.22	1132720	bb			Unknown	

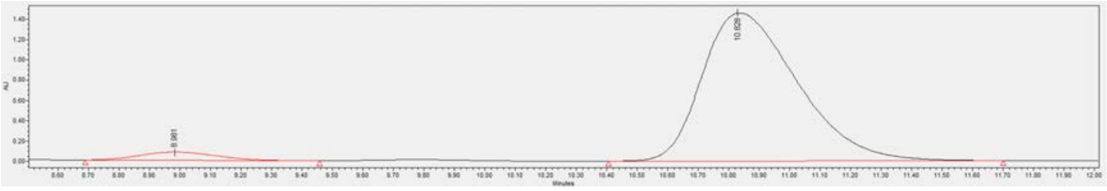

E	Name	Retention Time (min)	Area (µV*sec)	% Area	Height (µV)	Int Type	Amount	Units	Peak Type	Peak Codes
1		8.981	1401866	4.26	81150	bb			Unknown	
2		10.828	31503018	95.74	1454899	bb			Unknown	

Figure S75. HPLC spectrum for **1c**

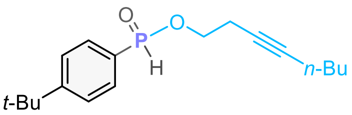

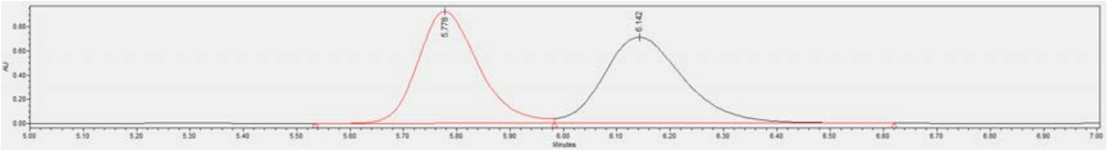

	Name	Retention Time (min)	Area (µV*sec)	% Area	Height (µV)	Int Type	Amount	Units	Peak Type	Peak Codes
1		5.778	7344382	49.17	924311	Vv			Unknown	
2		6.142	7592271	50.83	713062	vB			Unknown	

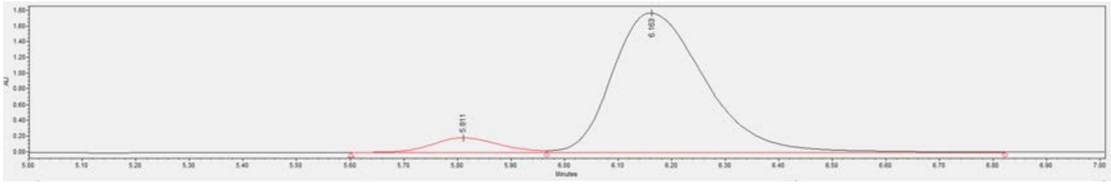

	Name	Retention Time (min)	Area (µV*sec)	% Area	Height (µV)	Int Type	Amount	Units	Peak Type	Peak Codes
1		5.811	1626302	7.11	186987	bV			Unknown	
2		6.163	21244723	92.89	1773465	VV			Unknown	

Figure S76. HPLC spectrum for **1d**

Oct-3-yn-1-yl [1,1'-biphenyl]-4-ylphosphinate (1e)

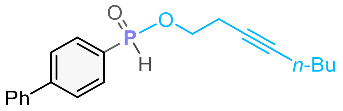

	Name	Retention Time (min)	Area (µV*sec)	% Area	Height (µV)	Int Type	Amount	Units	Peak Type	Peak Codes
1		21.633	16486581	50.60	421032	bV			Unknown	
2		23.543	16093817	49.40	379032	Vb			Unknown	

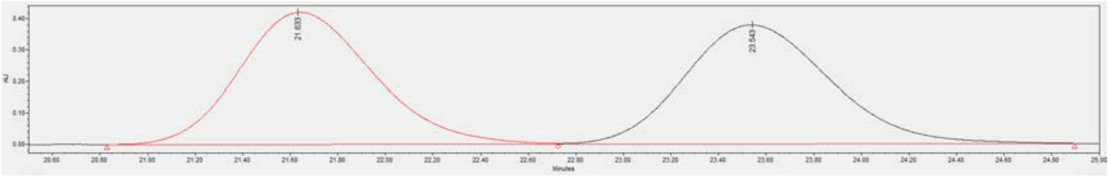

	Name	Retention Time (min)	Area (µV*sec)	% Area	Height (µV)	Int Type	Amount	Units	Peak Type	Peak Codes
1		21.424	75267755	95.03	1663824	bV			Unknown	
2		23.567	3936447	4.97	90912	Vb			Unknown	

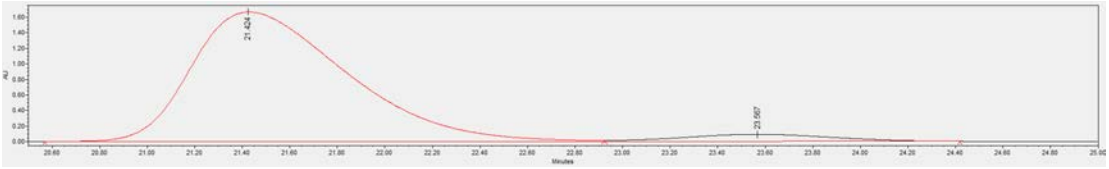

Figure S77. HPLC spectrum for **1e**

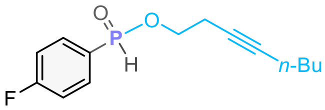

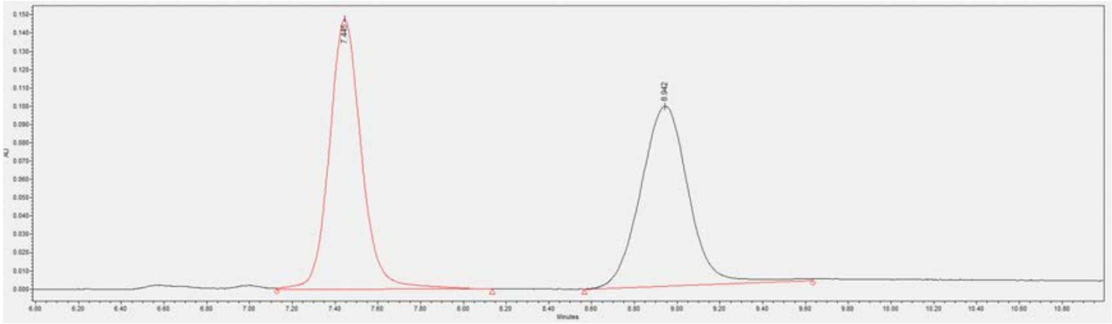

	Name	Retention Time (min)	Area (µV*sec)	% Area	Height (µV)	Int Type	Amount	Units	Peak Type	Peak Codes
1		7.445	1538861	50.23	147505	VB			Unknown	
2		8.942	1524709	49.77	98400	BV			Unknown	

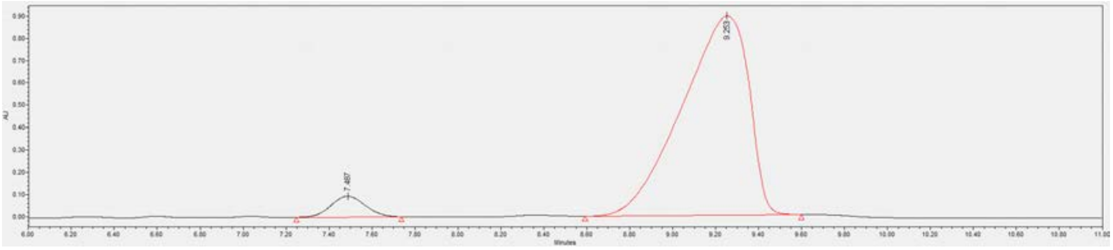

	Name	Retention Time (min)	Area (µV*sec)	% Area	Height (µV)	Int Type	Amount	Units	Peak Type	Peak Codes
1		7.487	1041543	5.12	93827	bb			Unknown	
2		9.253	19317190	94.88	895390	bb			Unknown	

Figure S78. HPLC spectrum for **1f**

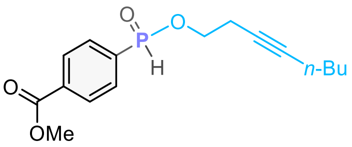

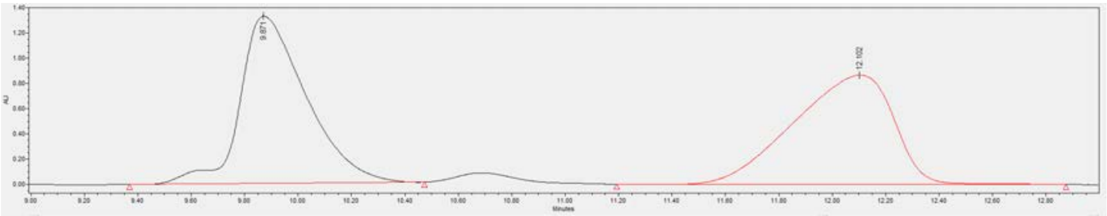

E	Name	Retention Time (min)	Area (µV*sec)	% Area	Height (µV)	Int Type	Amount	Units	Peak Type	Peak Codes
1		9.871	22932445	50.39	1327679	bb			Unknown	
2		12.102	22580653	49.61	867670	bb			Unknown	

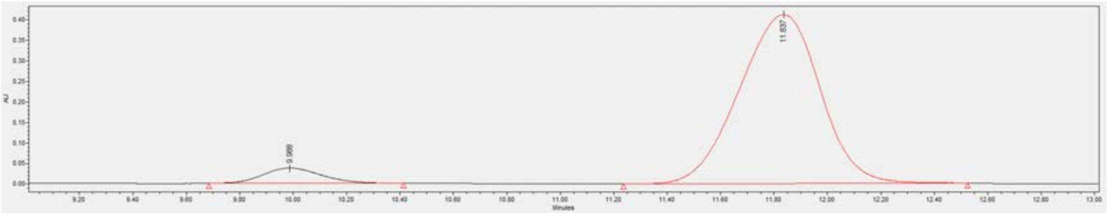

E	Name	Retention Time (min)	Area (µV*sec)	% Area	Height (µV)	Int Type	Amount	Units	Peak Type	Peak Codes
1		9.988	553922	6.00	37076	bb			Unknown	
2		11.837	8673732	94.00	411789	bb			Unknown	

Figure S79. HPLC spectrum for **1g**

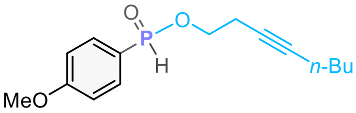

	Name	Retention Time (min)	Area (µV*sec)	% Area	Height (µV)	Int Type	Amount	Units	Peak Type	Peak Codes
1		9.637	7785812	49.61	416145	VV			Unknown	
2		14.110	7909521	50.39	81557	VB			Unknown	

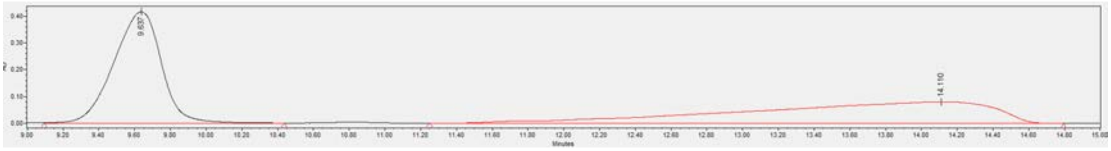

	Name	Retention Time (min)	Area (µV*sec)	% Area	Height (µV)	Int Type	Amount	Units	Peak Type	Peak Codes
1		9.307	616014	2.47	45653	bb			Unknown	
2		12.515	24352145	97.53	472106	bb			Unknown	

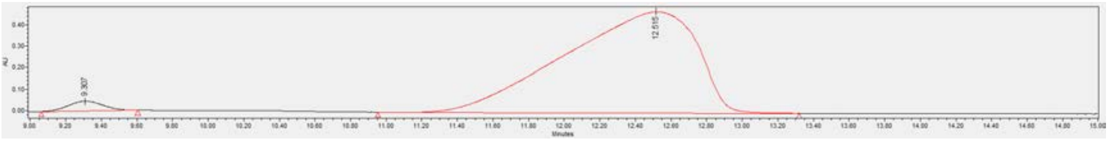

Figure S80. HPLC spectrum for **1h**

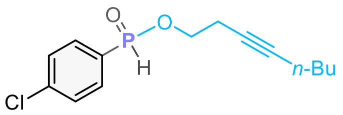

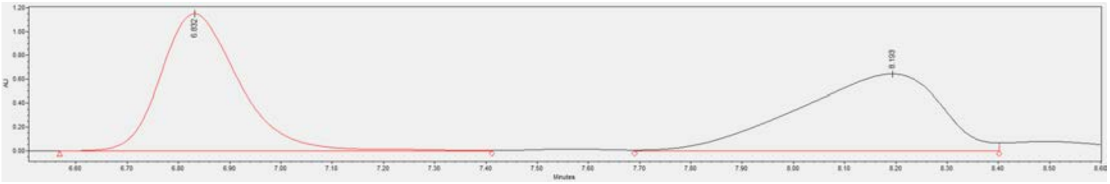

Name	Retention Time (min)	Area (µV*sec)	% Area	Height (µV)	Int Type	Amount	Units	Peak Type	Peak Codes
1		6.832	12249565	49.46	1153585	bV			Unknown	
2		8.193	12517952	50.54	647377	VV			Unknown	

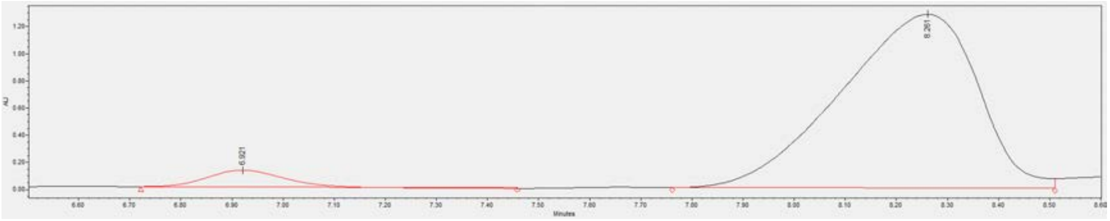

Name	Retention Time (min)	Area (µV*sec)	% Area	Height (µV)	Int Type	Amount	Units	Peak Type	Peak Codes
1		6.921	1354348	5.41	122198	bV			Unknown	
2		8.261	23701614	94.59	1276535	VV			Unknown	

Figure S81. HPLC spectrum for **1i**

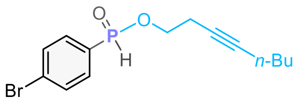

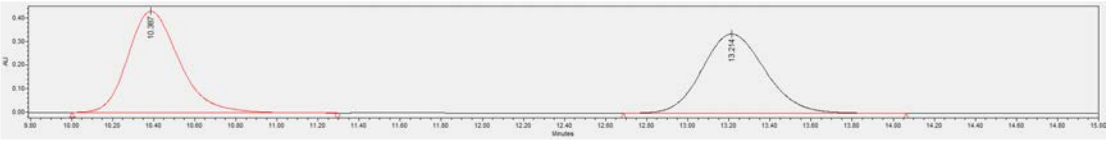

E	Name	Retention Time (min)	Area (µV*sec)	% Area	Height (µV)	Int Type	Amount	Units	Peak Type	Peak Codes
1		10.387	7474424	51.00	431742	bV			Unknown	
2		13.214	7179983	49.00	336651	BB			Unknown	

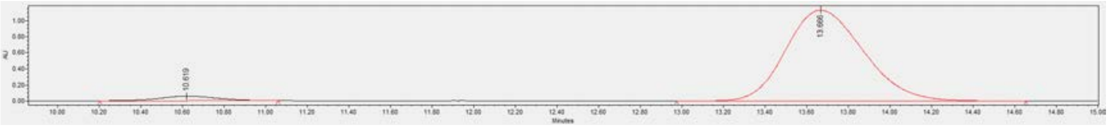

E	Name	Retention Time (min)	Area (µV*sec)	% Area	Height (µV)	Int Type	Amount	Units	Peak Type	Peak Codes
1		10.619	1133875	3.71	57172	bb			Unknown	
2		13.666	29430893	96.29	1133001	bb			Unknown	

Figure S82. HPLC spectrum for **1j**

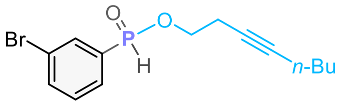

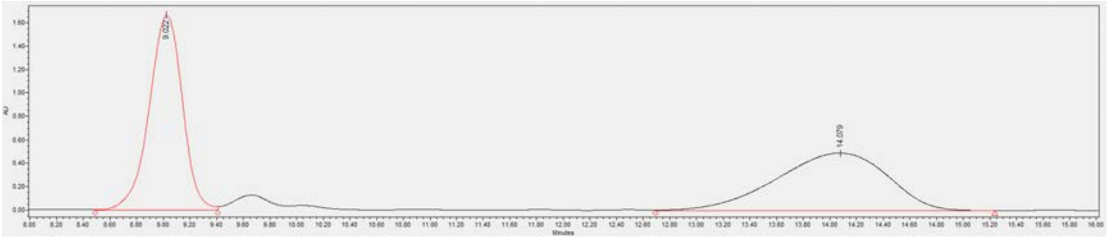

	Name	Retention Time (min)	Area (µV*sec)	% Area	Height (µV)	Int Type	Amount	Units	Peak Type	Peak Codes
1		9.022	29001729	50.47	1665533	VV			Unknown	
2		14.079	28463958	49.53	492692	VB			Unknown	

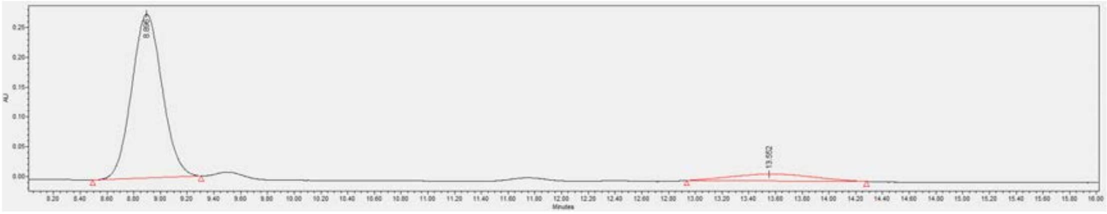

	Name	Retention Time (min)	Area (µV*sec)	% Area	Height (µV)	Int Type	Amount	Units	Peak Type	Peak Codes
1		8.896	4377993	89.98	275617	bb			Unknown	
2		13.552	487626	10.02	11584	bb			Unknown	

Figure S83. HPLC spectrum for **1k**

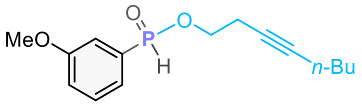

	Name	Retention Time (min)	Area (µV*sec)	% Area	Height (µV)	Int Type	Amount	Units	Peak Type	Peak Codes
1		12.669	16747454	50.06	811248	VV			Unknown	
2		14.831	16705993	49.94	674237	BB			Unknown	

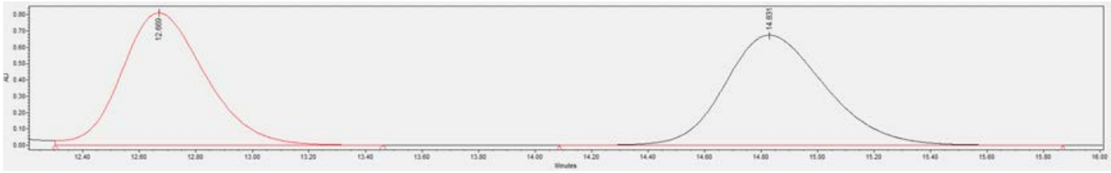

	Name	Retention Time (min)	Area (µV*sec)	% Area	Height (µV)	Int Type	Amount	Units	Peak Type	Peak Codes
1		12.838	1290269	3.29	72385	bb			Unknown	I08
2		14.994	37924124	96.71	1410373	Vb			Unknown	

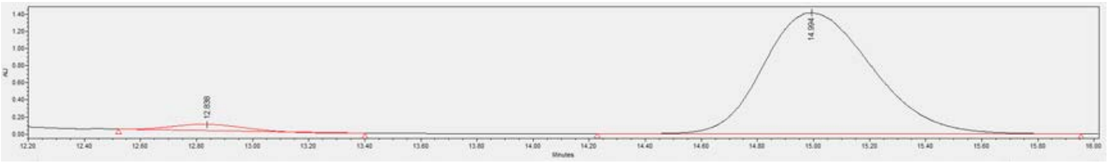

Figure S84. HPLC spectrum for **1l**

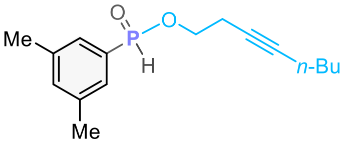

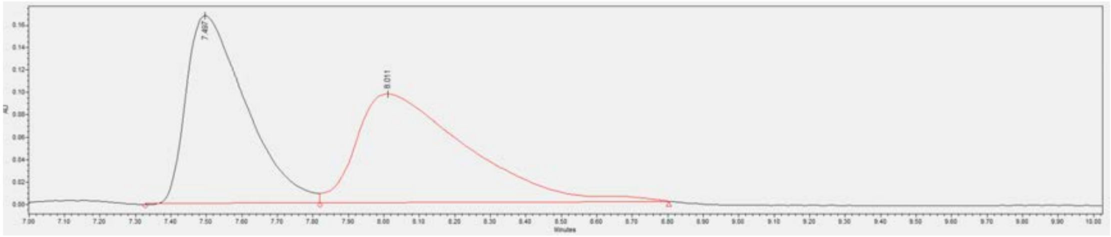

	Name	Retention Time (min)	Area (µV*sec)	% Area	Height (µV)	Int Type	Amount	Units	Peak Type	Peak Codes
1		7.497	1954262	48.28	167383	VV			Unknown	
2		8.011	2093677	51.72	96743	Vb			Unknown	

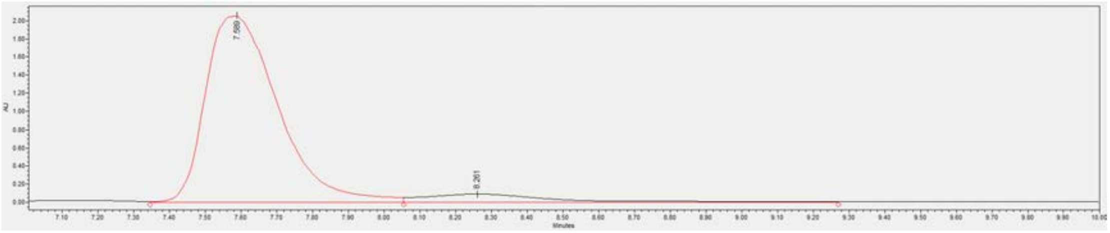

	Name	Retention Time (min)	Area (µV*sec)	% Area	Height (µV)	Int Type	Amount	Units	Peak Type	Peak Codes
1		7.589	28805064	93.22	2055318	VV			Unknown	
2		8.261	2095361	6.78	88882	VV			Unknown	

Figure S85. HPLC spectrum for **1m**

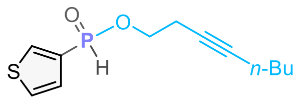

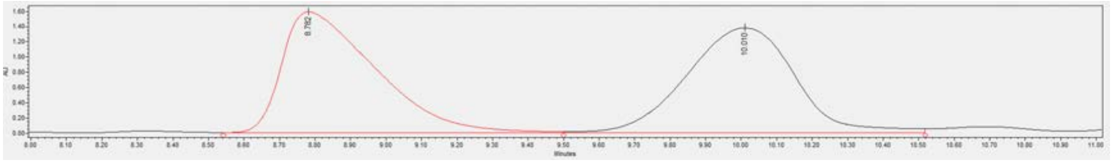

	Name	Retention Time (min)	Area (µV*sec)	% Area	Height (µV)	Int Type	Amount	Units	Peak Type	Peak Codes
1		8.782	29073770	49.97	1591463	VV			Unknown	
2		10.010	29108938	50.03	1377632	VV			Unknown	

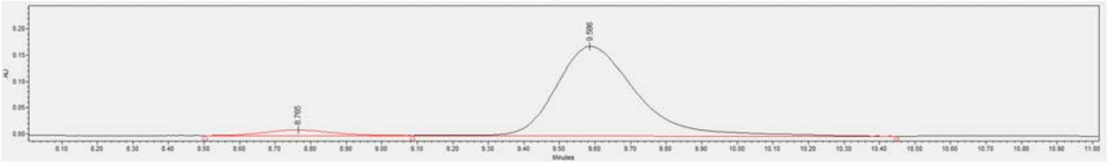

	Name	Retention Time (min)	Area (µV*sec)	% Area	Height (µV)	Int Type	Amount	Units	Peak Type	Peak Codes
1		8.765	175968	6.07	11790	VV			Unknown	
2		9.586	2721600	93.93	171360	VV			Unknown	

Figure S86. HPLC spectrum for **1n**

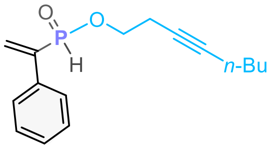

	Name	Retention Time (min)	Area (μV*sec)	% Area	Height (μV)	Int Type	Amount	Units	Peak Type	Peak Codes
1		27.991	28658811	50.32	772860	VV			Unknown	
2		32.389	28290705	49.68	644037	BB			Unknown	

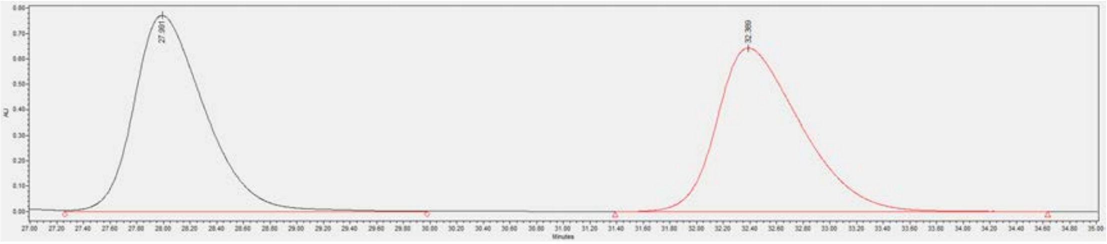

	Name	Retention Time (min)	Area (μV*sec)	% Area	Height (μV)	Int Type	Amount	Units	Peak Type	Peak Codes
1		27.550	26846119	91.41	538789	BB			Unknown	
2		32.235	2524072	8.59	46337	BB			Unknown	

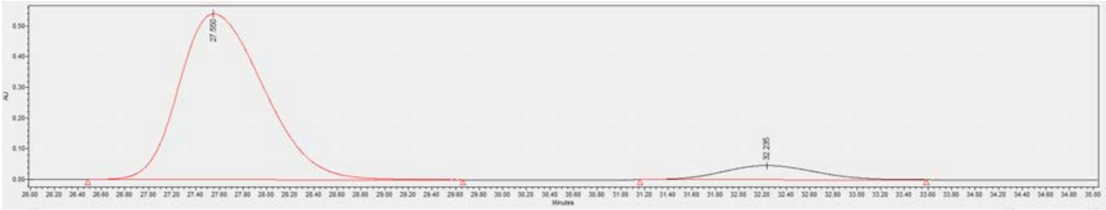

Figure S87. HPLC spectrum for **1o**

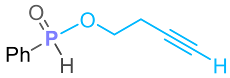

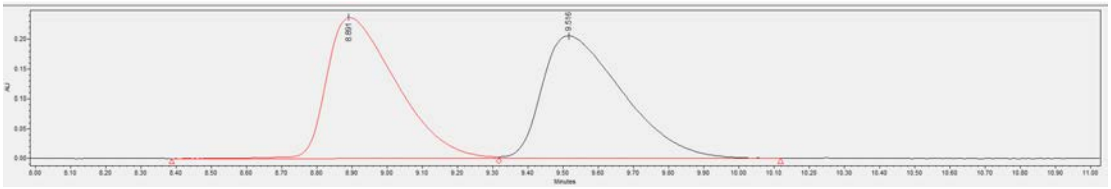

	Name	Retention Time (min)	Area (μV*sec)	% Area	Height (μV)	Int Type	Amount	Units	Peak Type	Peak Codes
1		8.891	3252162	49.91	237554	BV			Unknown	
2		9.516	3263694	50.09	206212	VB			Unknown	

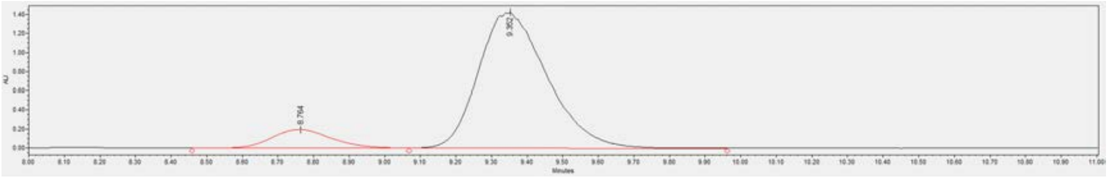

	Name	Retention Time (min)	Area (μV*sec)	% Area	Height (μV)	Int Type	Amount	Units	Peak Type	Peak Codes
1		8.764	2341580	10.89	199635	VV			Unknown	
2		9.352	19163370	89.11	1425418	VV			Unknown	

Figure S88. HPLC spectrum for **1p**

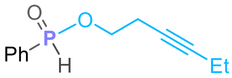

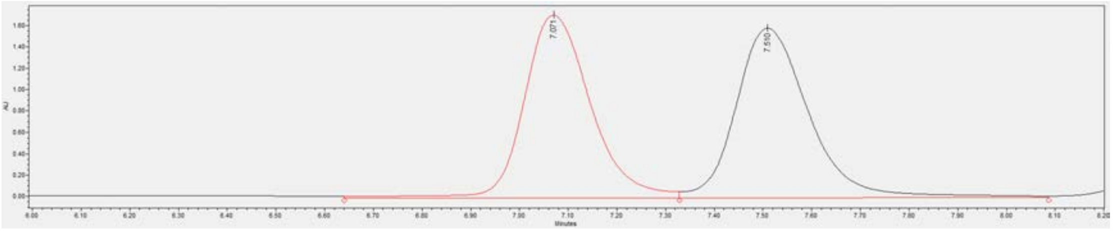

	Name	Retention Time (min)	Area (µV*sec)	% Area	Height (µV)	Int Type	Amount	Units	Peak Type	Peak Codes
1		7.071	16292813	49.57	1713761	VV			Unknown	
2		7.510	16573745	50.43	1587812	VV			Unknown	

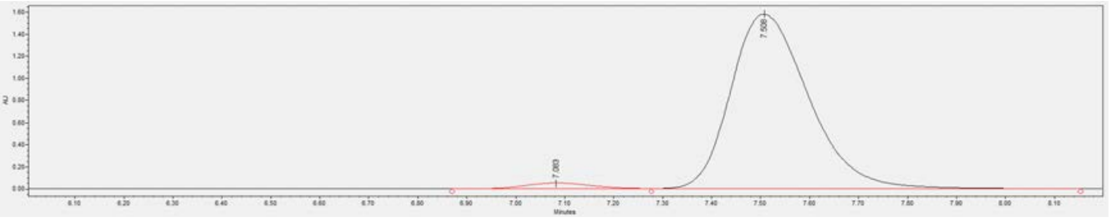

	Name	Retention Time (min)	Area (µV*sec)	% Area	Height (µV)	Int Type	Amount	Units	Peak Type	Peak Codes
1		7.083	527861	2.92	54155	VV			Unknown	
2		7.508	17569922	97.08	1579480	VV			Unknown	

Figure S89. HPLC spectrum for **1q**

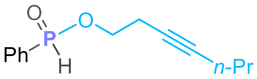

	Name	Retention Time (min)	Area (µV*sec)	% Area	Height (µV)	Int Type	Amount	Units	Peak Type	Peak Codes
1		6.955	3965525	49.62	373546	VV			Unknown	
2		7.705	4026285	50.38	290529	VV			Unknown	

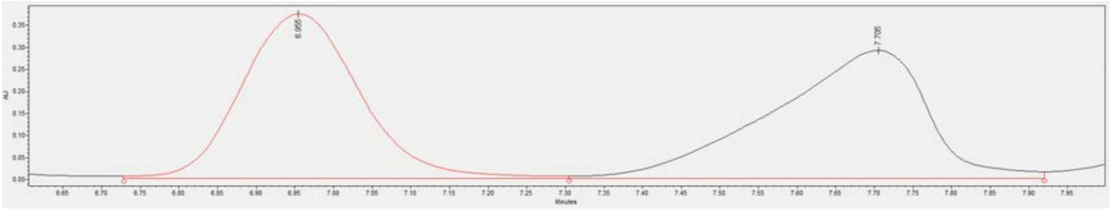

	Name	Retention Time (min)	Area (µV*sec)	% Area	Height (µV)	Int Type	Amount	Units	Peak Type	Peak Codes
1		6.972	481609	4.85	51663	bb			Unknown	
2		7.564	9443131	95.15	840012	bb			Unknown	

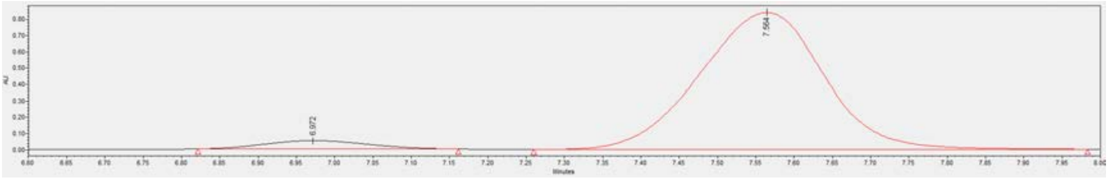

Figure S90. HPLC spectrum for **1r**

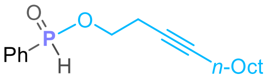

	Name	Retention Time (min)	Area (µV*sec)	% Area	Height (µV)	Int Type	Amount	Units	Peak Type	Peak Codes
1		10.633	7247702	49.92	499499	VV			Unknown	
2		11.447	7271880	50.08	454328	VV			Unknown	

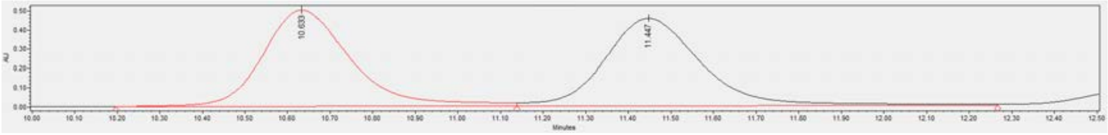

	Name	Retention Time (min)	Area (µV*sec)	% Area	Height (µV)	Int Type	Amount	Units	Peak Type	Peak Codes
1		10.604	1168082	5.18	73518	VV			Unknown	
2		11.375	21391866	94.82	1168659	Vb			Unknown	

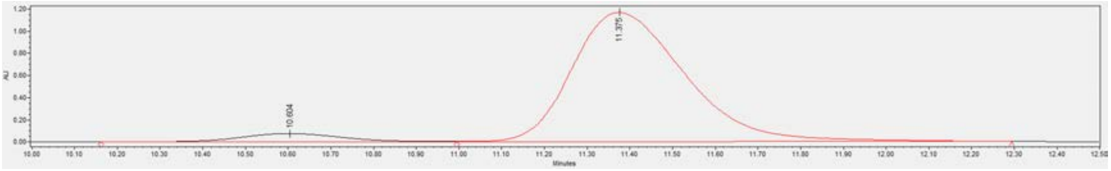

Figure S91. HPLC spectrum for **1s**

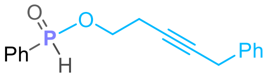

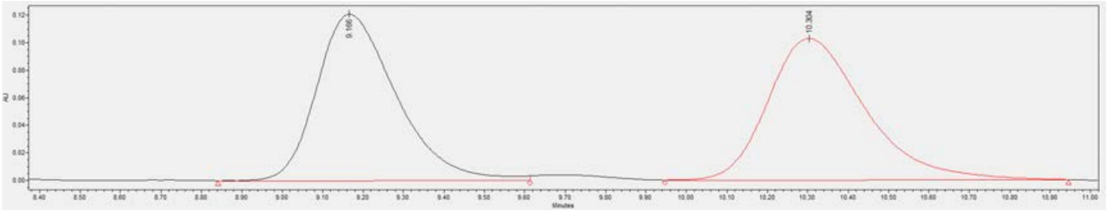

	Name	Retention Time (min)	Area (µV*sec)	% Area	Height (µV)	Int Type	Amount	Units	Peak Type	Peak Codes
1		9.166	1652511	49.03	120900	Bv			Unknown	
2		10.304	1717602	50.97	102833	Vb			Unknown	

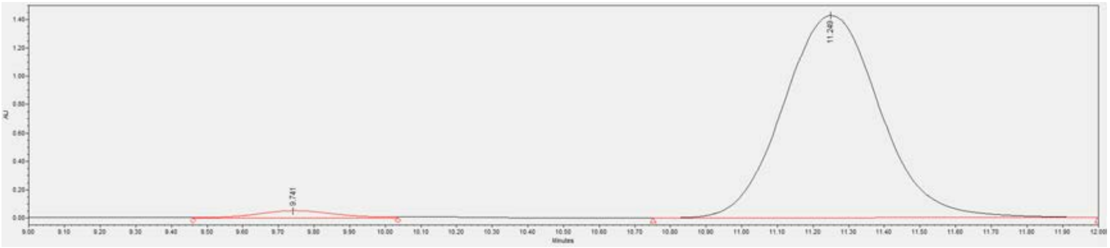

	Name	Retention Time (min)	Area (µV*sec)	% Area	Height (µV)	Int Type	Amount	Units	Peak Type	Peak Codes
1		9.741	803987	2.90	51817	VV			Unknown	
2		11.249	26919586	97.10	1426686	BB			Unknown	

Figure S92. HPLC spectrum for **1t**

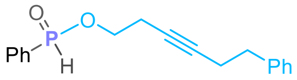

E	Name	Retention Time (min)	Area (µV*sec)	% Area	Height (µV)	Int Type	Amount	Units	Peak Type	Peak Codes
1		8.542	17699377	48.94	1468689	VV			Unknown	
2		9.054	18465028	51.06	1382673	Vb			Unknown	

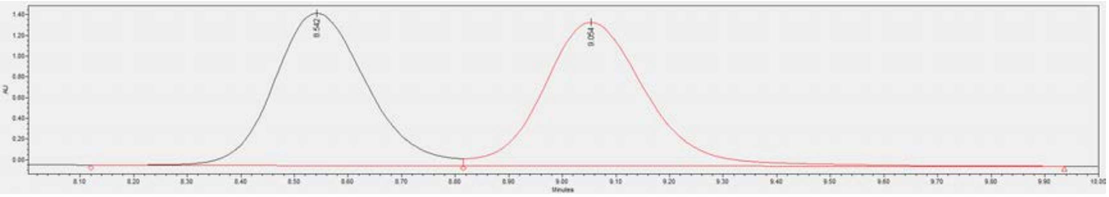

E	Name	Retention Time (min)	Area (µV*sec)	% Area	Height (µV)	Int Type	Amount	Units	Peak Type	Peak Codes
1		8.770	145906	3.47	10521	bV			Unknown	
2		9.257	4059074	96.53	275160	Vb			Unknown	

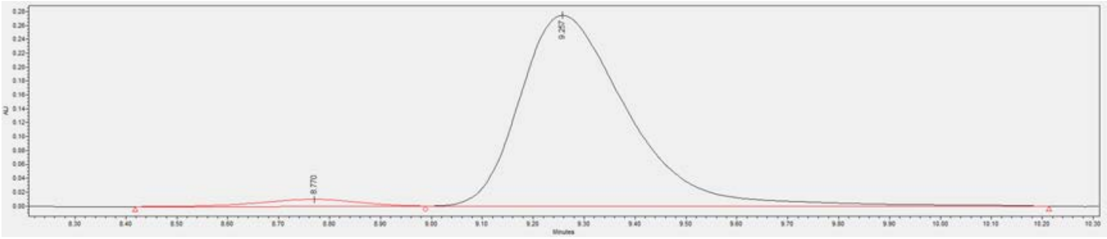

Figure S93. HPLC spectrum for **1u**

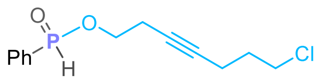

E	Name	Retention Time (min)	Area (μV*sec)	% Area	Height (μV)	Int Type	Amount	Units	Peak Type	Peak Codes
1		9.737	2096493	50.13	141168	VB			Unknown	
2		11.369	2085481	49.87	99492	BV			Unknown	

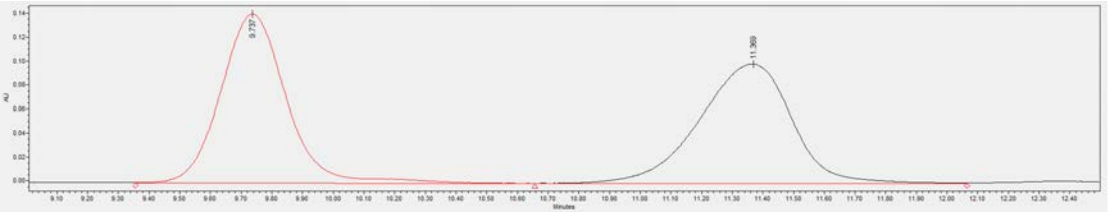

E	Name	Retention Time (min)	Area (μV*sec)	% Area	Height (μV)	Int Type	Amount	Units	Peak Type	Peak Codes
1		9.702	1697800	6.44	111048	bb			Unknown	
2		11.933	24668068	93.56	611959	bb			Unknown	

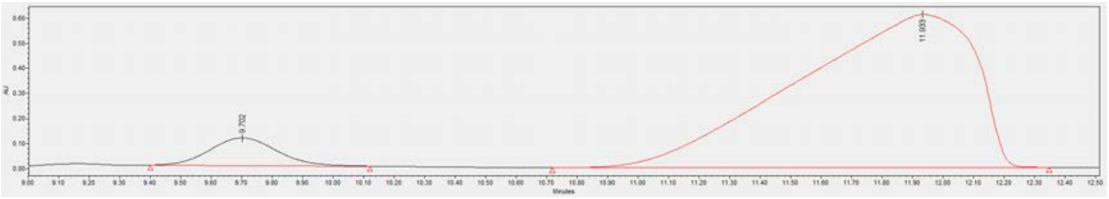

Figure S94. HPLC spectrum for **1v**

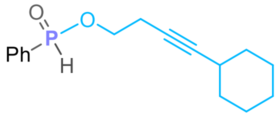

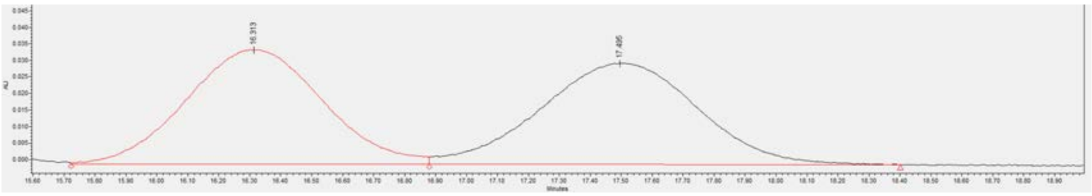

	Name	Retention Time (min)	Area (μV*sec)	% Area	Height (μV)	Int Type	Amount	Units	Peak Type	Peak Codes
1		16.313	1061625	49.54	34572	VV			Unknown	
2		17.495	1081381	50.46	30572	VB			Unknown	

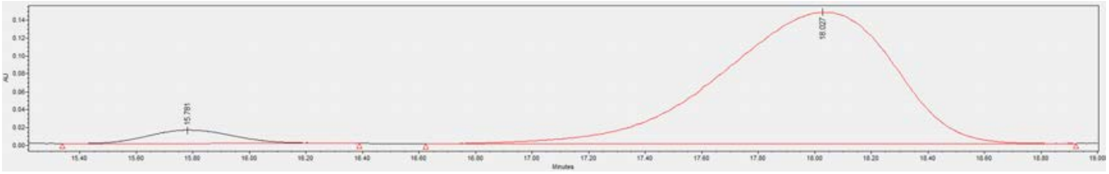

	Name	Retention Time (min)	Area (μV*sec)	% Area	Height (μV)	Int Type	Amount	Units	Peak Type	Peak Codes
1		15.781	324452	4.95	15313	bb			Unknown	
2		18.027	6235439	95.05	147280	bb			Unknown	

Figure S95. HPLC spectrum for **1w**

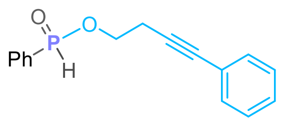

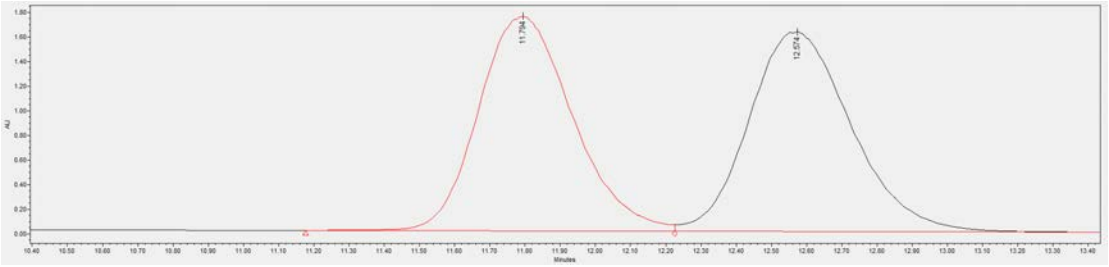

E	Name	Retention Time (min)	Area (μV*sec)	% Area	Height (μV)	Int Type	Amount	Units	Peak Type	Peak Codes
1		11.794	32414426	49.73	1742471	BV			Unknown	
2		12.574	32761641	50.27	1621591	VB			Unknown	

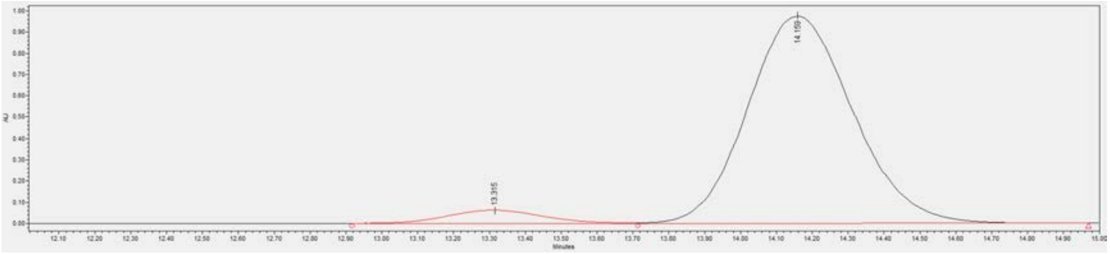

E	Name	Retention Time (min)	Area (μV*sec)	% Area	Height (μV)	Int Type	Amount	Units	Peak Type	Peak Codes
1		13.315	1135453	5.42	62087	VV			Unknown	
2		14.159	19817615	94.58	974935	VB			Unknown	

Figure S96. HPLC spectrum for **1x**

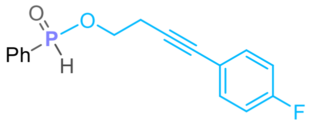

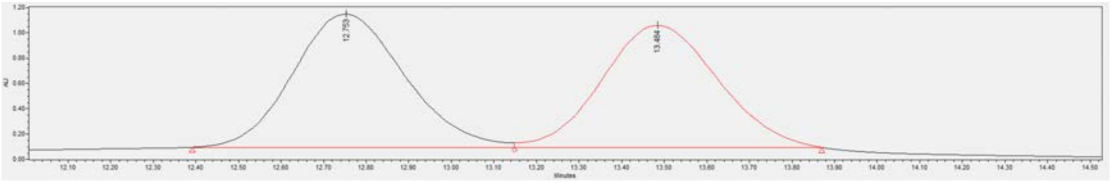

E	Name	Retention Time (min)	Area (µV*sec)	% Area	Height (µV)	Int Type	Amount	Units	Peak Type	Peak Codes
1		12.753	19117880	51.22	1052961	bV			Unknown	
2		13.484	18206681	48.78	963766	Vb			Unknown	

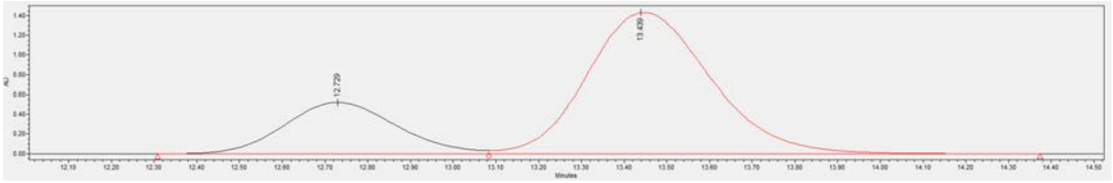

E	Name	Retention Time (min)	Area (µV*sec)	% Area	Height (µV)	Int Type	Amount	Units	Peak Type	Peak Codes
1		12.729	9559773	24.91	523730	BV			Unknown	
2		13.439	28822731	75.09	1431515	Vb			Unknown	

Figure S97. HPLC spectrum for **1y**

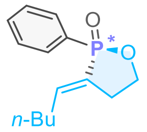

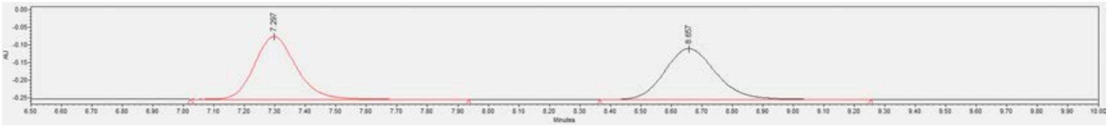

E	Name	Retention Time (min)	Area (µV*sec)	% Area	Height (µV)	Int Type	Amount	Units	Peak Type	Peak Codes
1		7.297	1734076	50.46	178362	VB			Unknown	
2		8.657	1702421	49.54	144177	BB			Unknown	

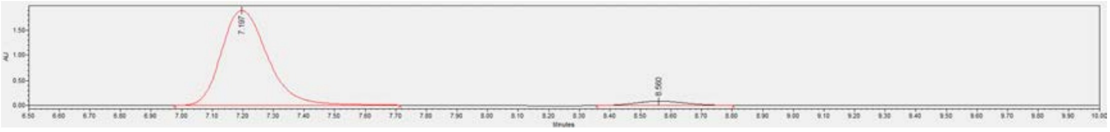

E	Name	Retention Time (min)	Area (µV*sec)	% Area	Height (µV)	Int Type	Amount	Units	Peak Type	Peak Codes
1		7.197	19880090	95.19	1895867	bb			Unknown	
2		8.560	1003982	4.81	88173	bb			Unknown	

Figure S98. HPLC spectrum for **2a**

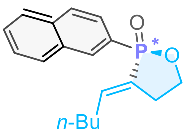

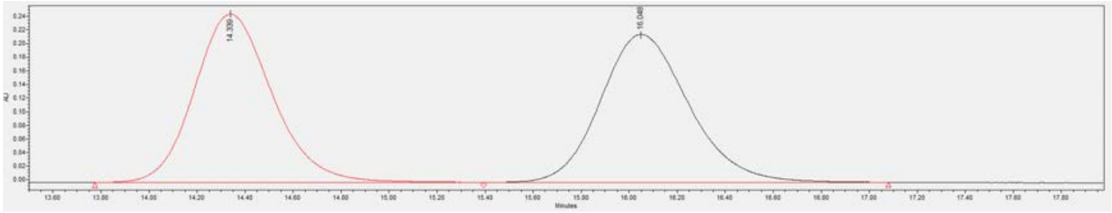

E	Name	Retention Time (min)	Area (μV*sec)	% Area	Height (μV)	Int Type	Amount	Units	Peak Type	Peak Codes
1		14.339	5609121	50.14	247983	BV			Unknown	
2		16.048	5578302	49.86	217698	VB			Unknown	

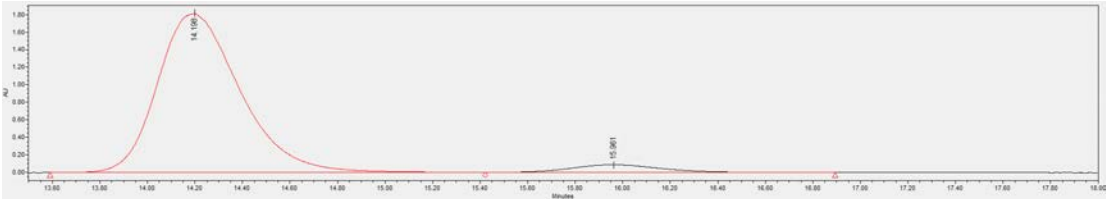

E	Name	Retention Time (min)	Area (μV*sec)	% Area	Height (μV)	Int Type	Amount	Units	Peak Type	Peak Codes
1		14.198	43877464	94.79	1816415	BV			Unknown	
2		15.961	2413694	5.21	89651	VB			Unknown	

Figure S99. HPLC spectrum for **2b**

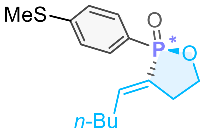

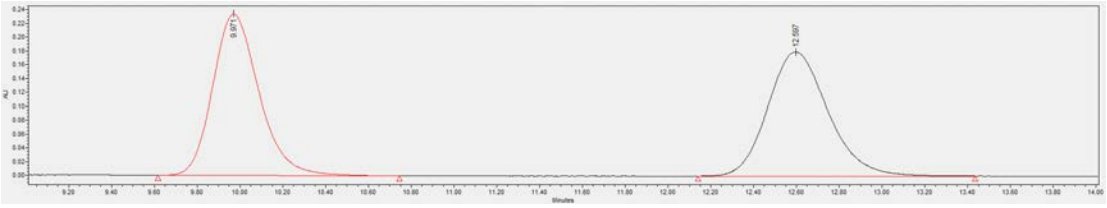

E	Name	Retention Time (min)	Area (µV*sec)	% Area	Height (µV)	Int Type	Amount	Units	Peak Type	Peak Codes
1		9.971	3517986	50.01	233925	BB			Unknown	
2		12.597	3516965	49.99	179742	BB			Unknown	

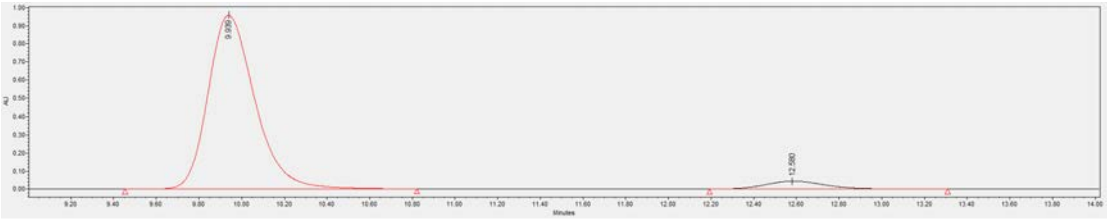

E	Name	Retention Time (min)	Area (µV*sec)	% Area	Height (µV)	Int Type	Amount	Units	Peak Type	Peak Codes
1		9.939	14533756	94.35	956337	BB			Unknown	
2		12.580	870445	5.65	44237	BB			Unknown	

Figure S100. HPLC spectrum for **2c**

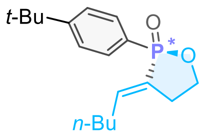

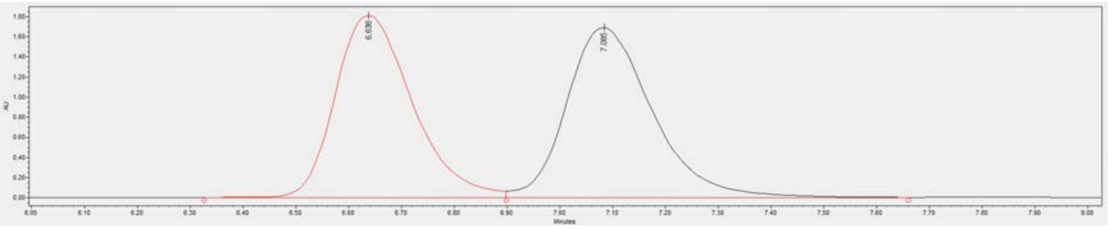

	Name	Retention Time (min)	Area (μV*sec)	% Area	Height (μV)	Int Type	Amount	Units	Peak Type	Peak Codes
1		6.638	18439813	49.05	1807778	VV			Unknown	
2		7.085	19155449	50.95	1690489	VV			Unknown	

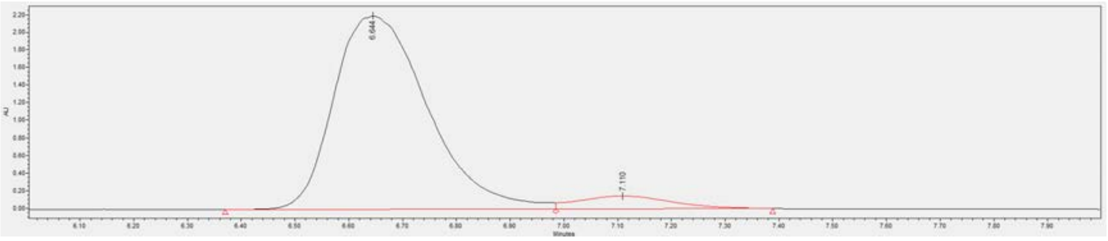

	Name	Retention Time (min)	Area (μV*sec)	% Area	Height (μV)	Int Type	Amount	Units	Peak Type	Peak Codes
1		6.644	26564918	93.86	2198485	BV			Unknown	
2		7.110	1738416	6.14	145501	Vb			Unknown	

Figure S101. HPLC spectrum for **2d**

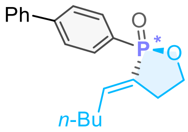

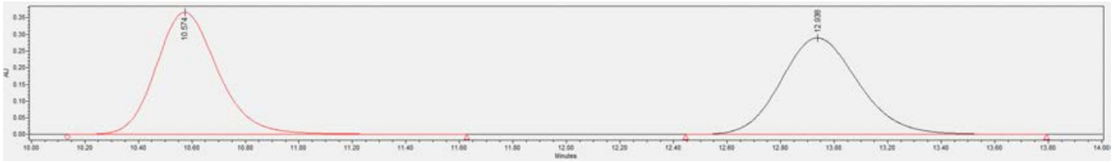

E	Name	Retention Time (min)	Area (µV*sec)	% Area	Height (µV)	Int Type	Amount	Units	Peak Type	Peak Codes
1		10.574	5905131	50.25	365317	VB			Unknown	
2		12.938	5846364	49.75	289269	BB			Unknown	

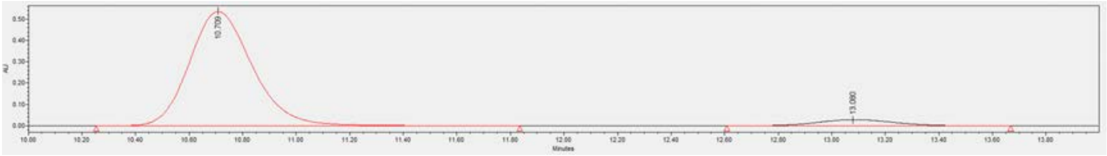

E	Name	Retention Time (min)	Area (µV*sec)	% Area	Height (µV)	Int Type	Amount	Units	Peak Type	Peak Codes
1		10.709	8829783	93.58	537065	BB			Unknown	
2		13.080	605488	6.42	29504	BB			Unknown	

Figure S102. HPLC spectrum for **2e**

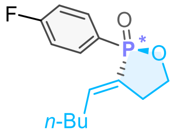

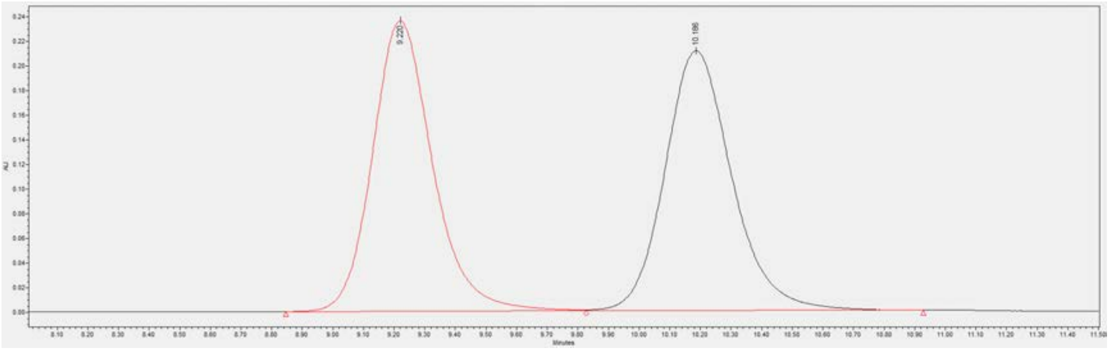

	Name	Retention Time (min)	Area (µV*sec)	% Area	Height (µV)	Int Type	Amount	Units	Peak Type	Peak Codes
1		9.220	3219038	50.12	235762	BV			Unknown	
2		10.186	3204001	49.88	211045	VB			Unknown	

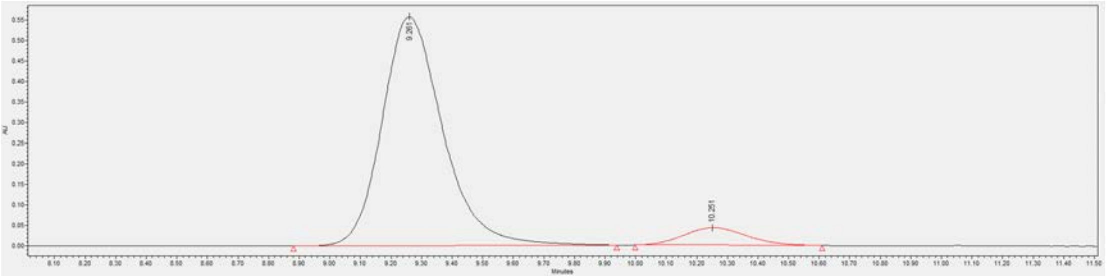

	Name	Retention Time (min)	Area (µV*sec)	% Area	Height (µV)	Int Type	Amount	Units	Peak Type	Peak Codes
1		9.261	7707943	92.61	558316	bb			Unknown	
2		10.251	614984	7.39	42702	bb			Unknown	

Figure S103. HPLC spectrum for **2f**

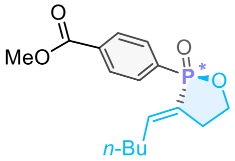

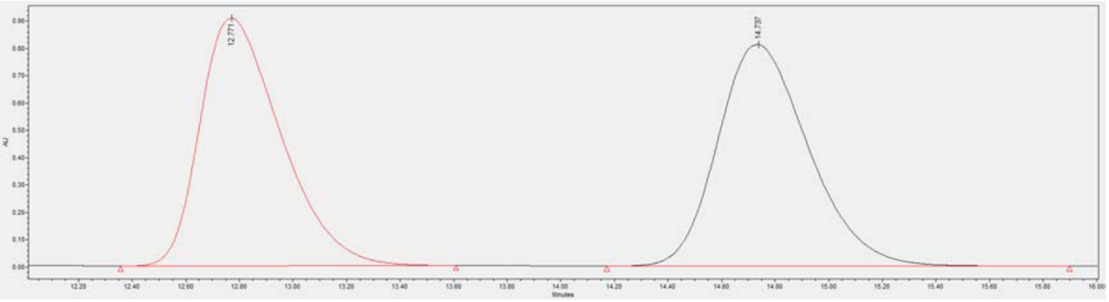

Name	Retention Time (min)	Area (µV*sec)	% Area	Height (µV)	Int Type	Amount	Units	Peak Type	Peak Codes
1		12.771	18979901	49.80	905988	BB			Unknown	
2		14.737	19130357	50.20	810883	BB			Unknown	

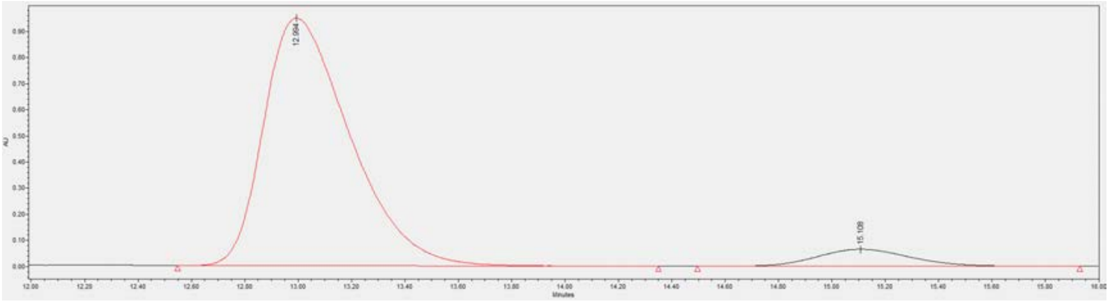

	Name	Retention Time (min)	Area (µV*sec)	% Area	Height (µV)	Int Type	Amount	Units	Peak Type	Peak Codes
1		12.994	21289790	93.00	948119	BB			Unknown	
2		15.108	1602142	7.00	65337	BB			Unknown	

Figure S104. HPLC spectrum for **2g**

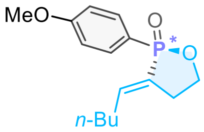

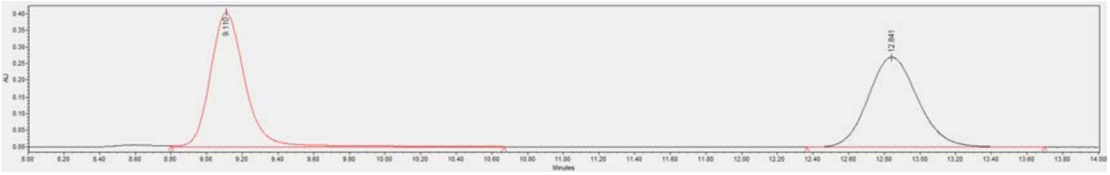

E	Name	Retention Time (min)	Area (µV*sec)	% Area	Height (µV)	Int Type	Amount	Units	Peak Type	Peak Codes
1		9.110	5457200	50.97	404772	VB			Unknown	
2		12.841	5250071	49.03	270446	BB			Unknown	

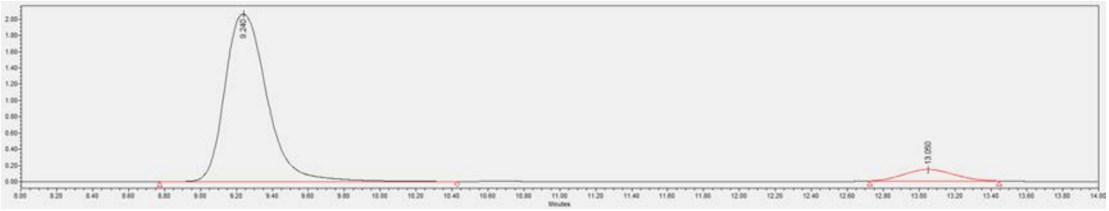

E	Name	Retention Time (min)	Area (µV*sec)	% Area	Height (µV)	Int Type	Amount	Units	Peak Type	Peak Codes
1		9.240	33427853	92.44	2060520	bV			Unknown	
2		13.050	2734750	7.56	141438	bb			Unknown	

Figure S105. HPLC spectrum for **2h**

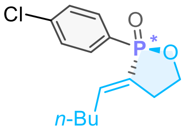

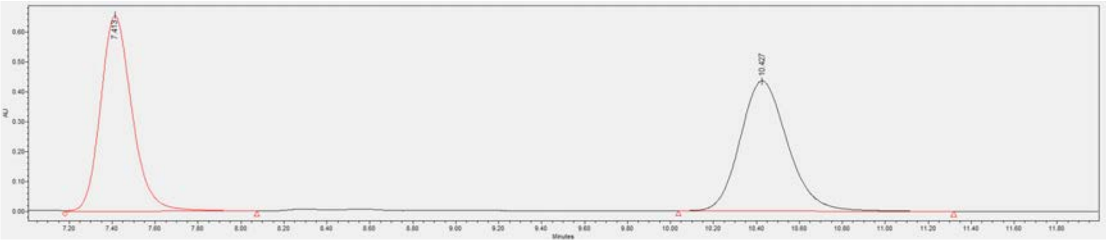

E	Name	Retention Time (min)	Area (µV*sec)	% Area	Height (µV)	Int Type	Amount	Units	Peak Type	Peak Codes
1		7.413	6662618	49.94	654515	VB			Unknown	
2		10.427	6679414	50.06	434782	BB			Unknown	

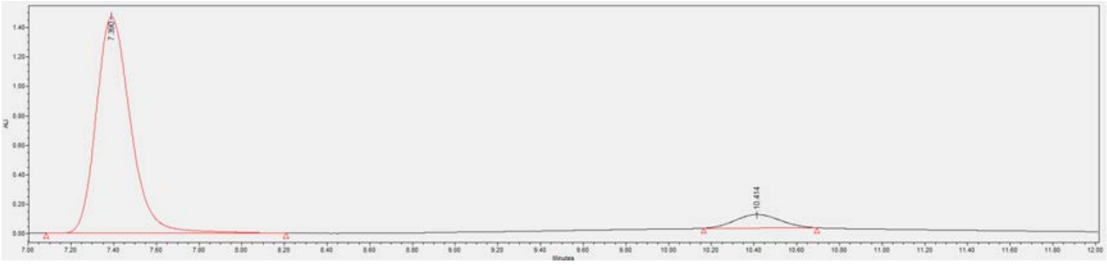

E	Name	Retention Time (min)	Area (µV*sec)	% Area	Height (µV)	Int Type	Amount	Units	Peak Type	Peak Codes
1		7.390	16405217	92.44	1470898	bb			Unknown	
2		10.414	1341504	7.56	92079	bb			Unknown	

Figure S106. HPLC spectrum for **2i**

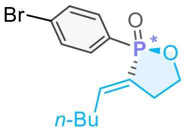

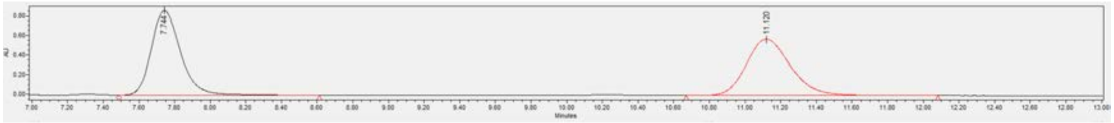

	Name	Retention Time (min)	Area (μV*sec)	% Area	Height (μV)	Int Type	Amount	Units	Peak Type	Peak Codes
1		7.744	9887395	49.98	865316	VB			Unknown	
2		11.120	9897087	50.02	572981	BB			Unknown	

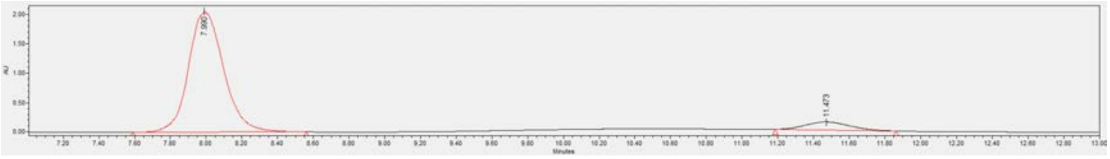

	Name	Retention Time (min)	Area (μV*sec)	% Area	Height (μV)	Int Type	Amount	Units	Peak Type	Peak Codes
1		7.990	28030859	92.16	2052781	bb			Unknown	
2		11.473	2385081	7.84	138348	bb			Unknown	

Figure S107. HPLC spectrum for **2j**

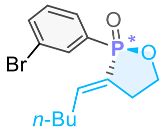

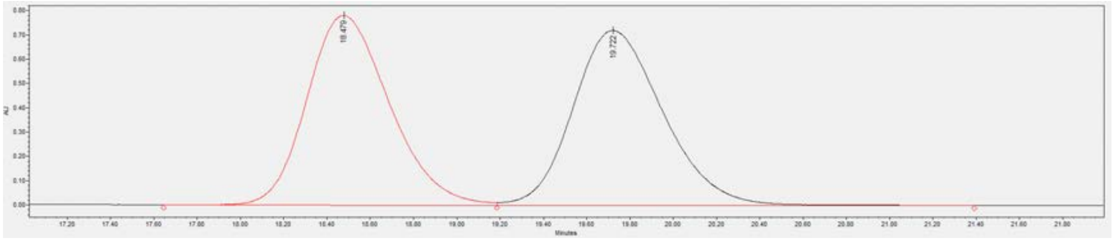

Name	Retention Time (min)	Area (µV*sec)	% Area	Height (µV)	Int Type	Amount	Units	Peak Type	Peak Codes
1		18.479	20807024	49.85	781844	VV			Unknown	
2		19.722	20931697	50.15	719924	VV			Unknown	

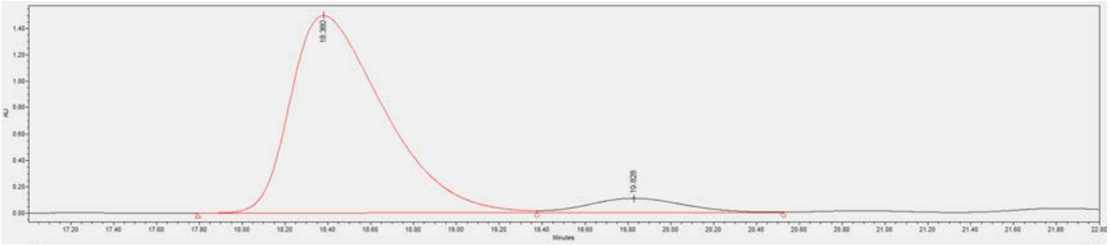

	Name	Retention Time (min)	Area (µV*sec)	% Area	Height (µV)	Int Type	Amount	Units	Peak Type	Peak Codes
1		18.380	45385224	92.95	1498525	BV			Unknown	
2		19.828	3442669	7.05	110175	VV			Unknown	

Figure S108. HPLC spectrum for **2k**

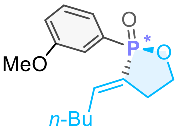

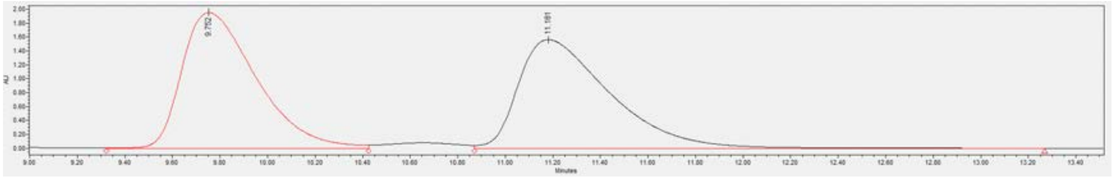

	Name	Retention Time (min)	Area (µV*sec)	% Area	Height (µV)	Int Type	Amount	Units	Peak Type	Peak Codes
1		9.752	42347747	50.36	1954749	VV			Unknown	
2		11.181	41746247	49.64	1564215	VB			Unknown	

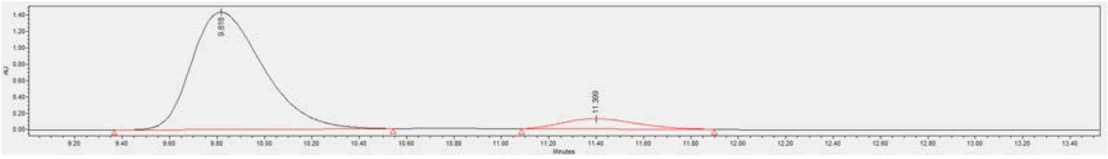

	Name	Retention Time (min)	Area (µV*sec)	% Area	Height (µV)	Int Type	Amount	Units	Peak Type	Peak Codes
1		9.818	30046513	91.61	1435634	bb			Unknown	
2		11.399	2752578	8.39	126108	bb			Unknown	

Figure S109. HPLC spectrum for **2l**

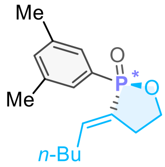

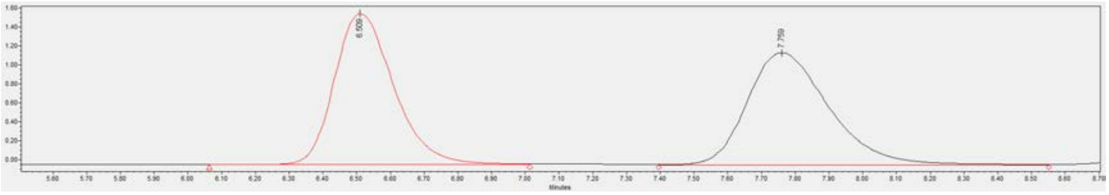

	Name	Retention Time (min)	Area (μV*sec)	% Area	Height (μV)	Int Type	Amount	Units	Peak Type	Peak Codes
1		6.509	19506049	49.61	1589384	BV			Unknown	
2		7.759	19812912	50.39	1179126	VV			Unknown	

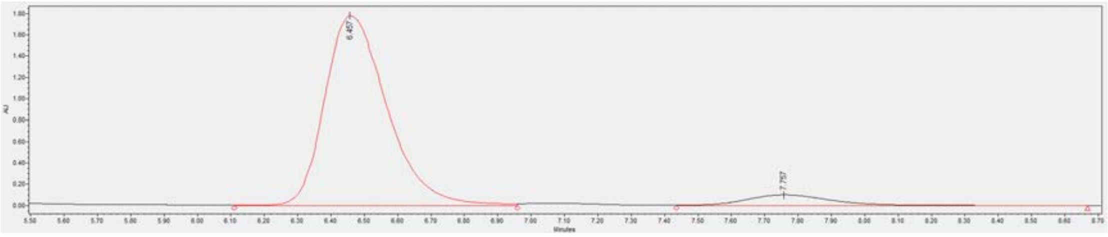

	Name	Retention Time (min)	Area (μV*sec)	% Area	Height (μV)	Int Type	Amount	Units	Peak Type	Peak Codes
1		6.457	23157935	92.86	1781322	VV			Unknown	
2		7.757	1780639	7.14	101073	VB			Unknown	

Figure S110. HPLC spectrum for **2m**

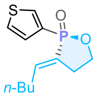

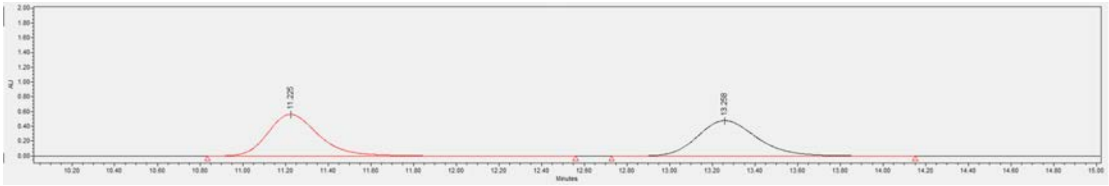

E	Name	Retention Time (min)	Area (μV*sec)	% Area	Height (μV)	Int Type	Amount	Units	Peak Type	Peak Codes
1		11.225	9336091	50.23	564490	BB			Unknown	
2		13.258	9249170	49.77	479222	BB			Unknown	

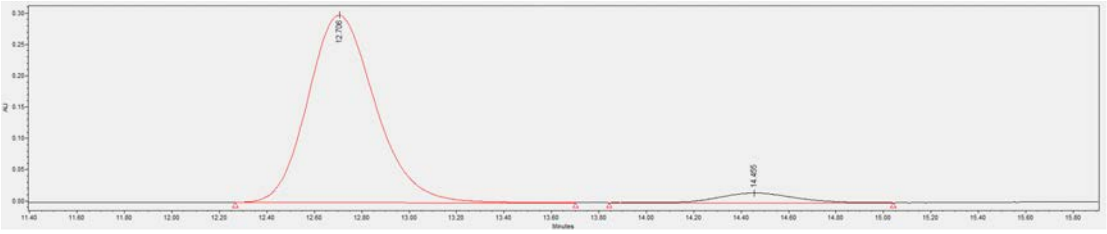

E	Name	Retention Time (min)	Area (μV*sec)	% Area	Height (μV)	Int Type	Amount	Units	Peak Type	Peak Codes
1		12.706	5913085	94.56	299475	BB			Unknown	
2		14.455	340345	5.44	15545	BB			Unknown	

Figure S111. HPLC spectrum for **2n**

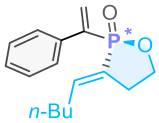

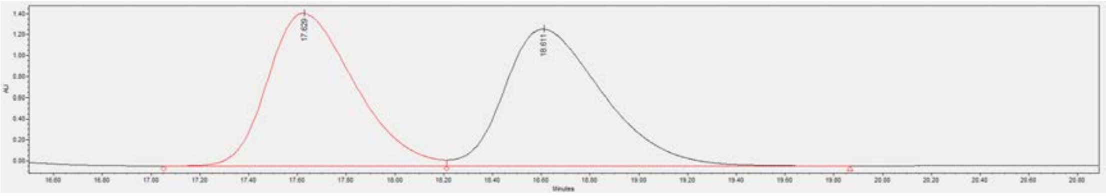

E	Name	Retention Time (min)	Area (µV*sec)	% Area	Height (µV)	Int Type	Amount	Units	Peak Type	Peak Codes
1		17.629	35963658	49.67	1453179	VV			Unknown	
2		18.611	36439883	50.33	1303344	VB			Unknown	

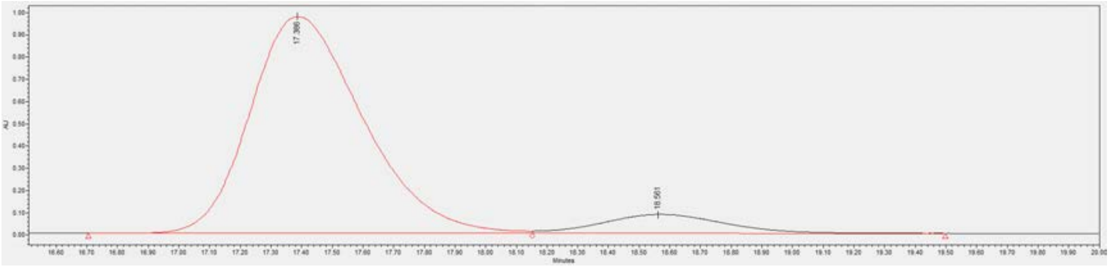

E	Name	Retention Time (min)	Area (µV*sec)	% Area	Height (µV)	Int Type	Amount	Units	Peak Type	Peak Codes
1		17.386	24918636	91.34	974045	BV			Unknown	
2		18.561	2361793	8.66	85092	VB			Unknown	

Figure S112. HPLC spectrum for **2o**

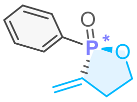

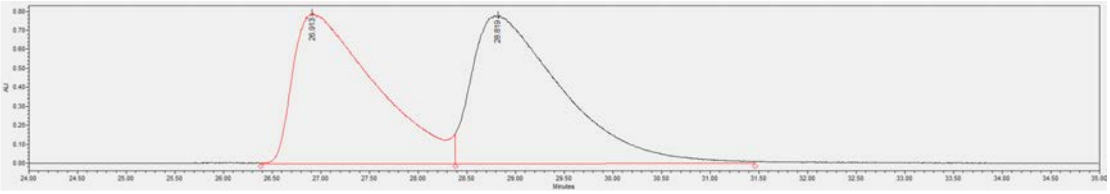

E	Name	Retention Time (min)	Area (µV*sec)	% Area	Height (µV)	Int Type	Amount	Units	Peak Type	Peak Codes
1		26.913	46431534	48.07	793502	Vv			Unknown	
2		28.819	50158387	51.93	781452	vV			Unknown	

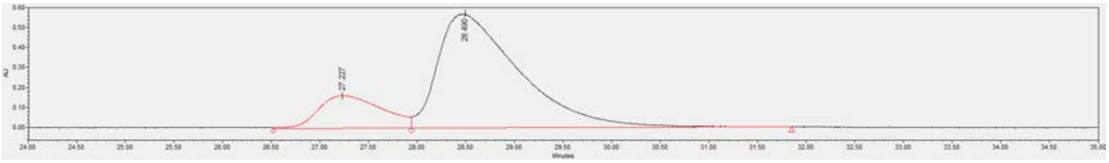

E	Name	Retention Time (min)	Area (µV*sec)	% Area	Height (µV)	Int Type	Amount	Units	Peak Type	Peak Codes
1		27.227	7465958	18.37	165248	Vv			Unknown	
2		28.490	33181637	81.63	573344	vb			Unknown	

Figure S113. HPLC spectrum for **2p**

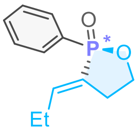

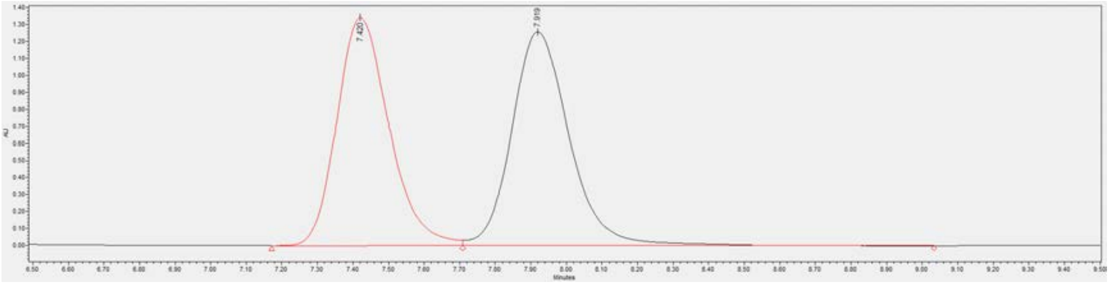

Name	Retention Time (min)	Area (µV*sec)	% Area	Height (µV)	Int Type	Amount	Units	Peak Type	Peak Codes
1		7.420	13687777	49.14	1342943	bV			Unknown	
2		7.919	14169554	50.86	1258198	VV			Unknown	

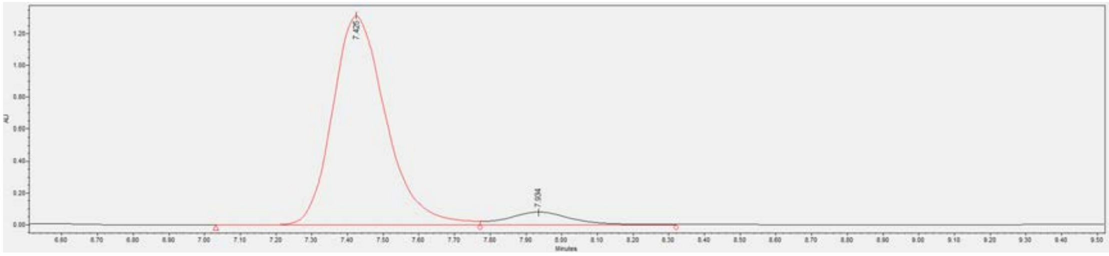

	Name	Retention Time (min)	Area (µV*sec)	% Area	Height (µV)	Int Type	Amount	Units	Peak Type	Peak Codes
1		7.425	13623658	93.20	1313612	BV			Unknown	
2		7.934	994483	6.80	80723	VV			Unknown	

Figure S114. HPLC spectrum for **2q**

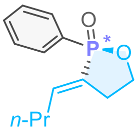

E	Name	Retention Time (min)	Area (µV*sec)	% Area	Height (µV)	Int Type	Amount	Units	Peak Type	Peak Codes
1		7.420	904375	49.42	90858	BB			Unknown	
2		8.616	925644	50.58	76340	BB			Unknown	

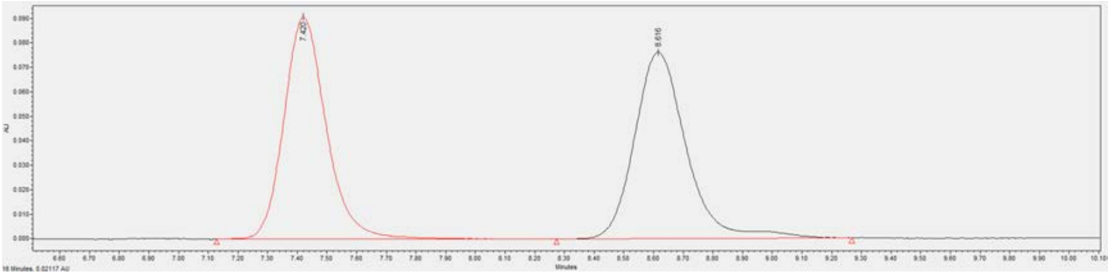

E	Name	Retention Time (min)	Area (µV*sec)	% Area	Height (µV)	Int Type	Amount	Units	Peak Type	Peak Codes
1		7.260	18253253	93.56	1603305	BV			Unknown	
2		8.456	1256955	6.44	98108	VB			Unknown	

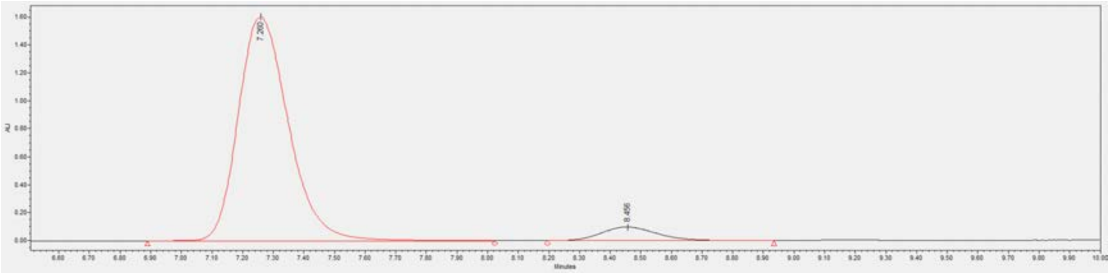

Figure S115. HPLC spectrum for **2r**

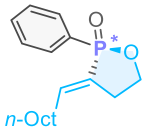

	Name	Retention Time (min)	Area (µV*sec)	% Area	Height (µV)	Int Type	Amount	Units	Peak Type	Peak Codes
1		8.864	696379	49.55	43416	BV			Unknown	
2		9.944	709013	50.45	39309	VB			Unknown	

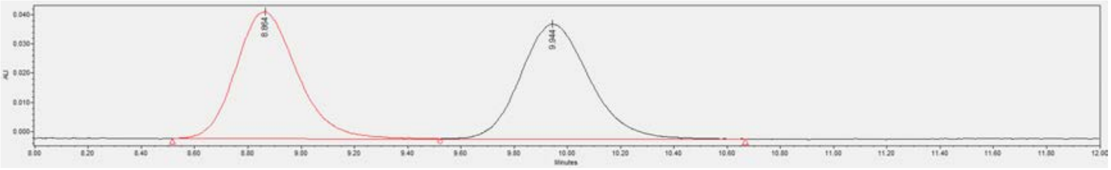

	Name	Retention Time (min)	Area (µV*sec)	% Area	Height (µV)	Int Type	Amount	Units	Peak Type	Peak Codes
1		8.616	989831	4.83	64027	bb			Unknown	
2		9.595	19509363	95.17	981229	bb			Unknown	

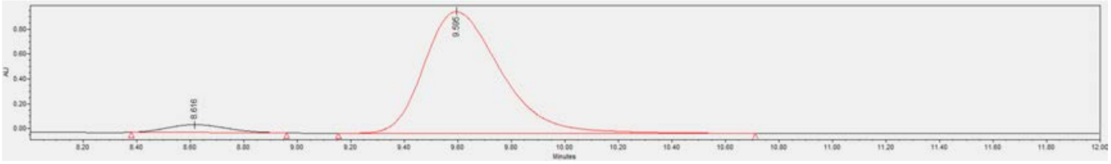

Figure S116. HPLC spectrum for **2s**

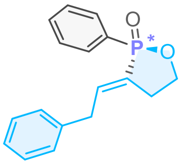

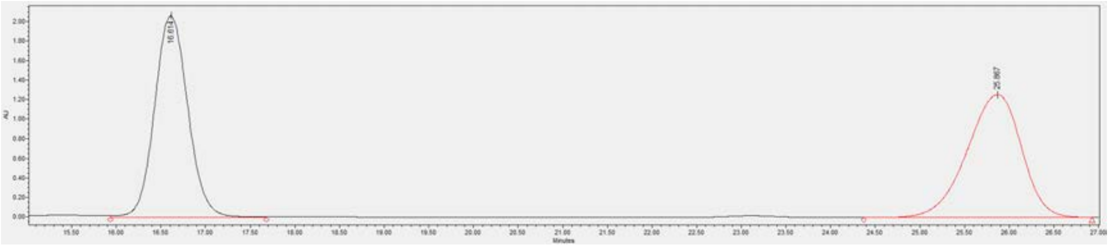

E	Name	Retention Time (min)	Area (μV*sec)	% Area	Height (μV)	Int Type	Amount	Units	Peak Type	Peak Codes
1		16.614	53435069	50.04	2064278	VV			Unknown	
2		25.867	53347140	49.96	1256492	Vb			Unknown	

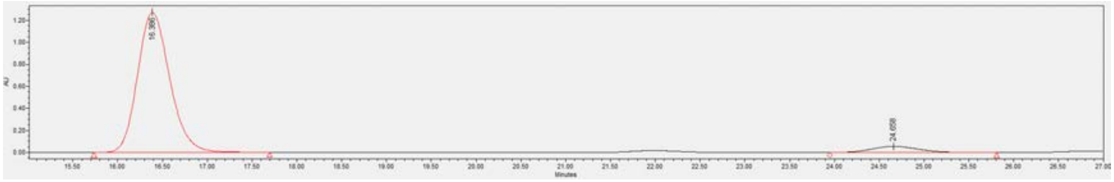

E	Name	Retention Time (min)	Area (μV*sec)	% Area	Height (μV)	Int Type	Amount	Units	Peak Type	Peak Codes
1		16.386	31664184	93.92	1268170	BB			Unknown	
2		24.658	2049483	6.08	56065	VB			Unknown	

Figure S117. HPLC spectrum for **2t**

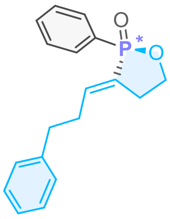

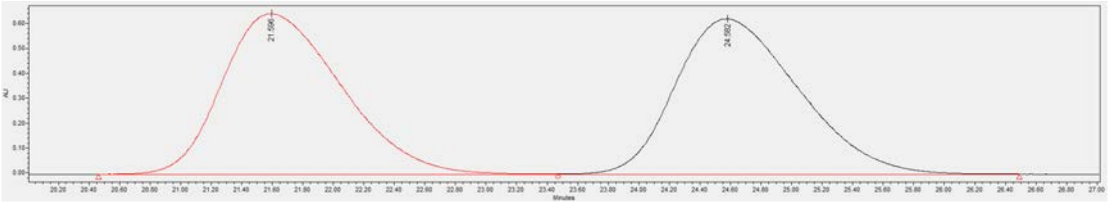

	Name	Retention Time (min)	Area (µV*sec)	% Area	Height (µV)	Int Type	Amount	Units	Peak Type	Peak Codes
1		21.596	34935167	50.03	644740	BV			Unknown	
2		24.582	34896861	49.97	624192	VB			Unknown	

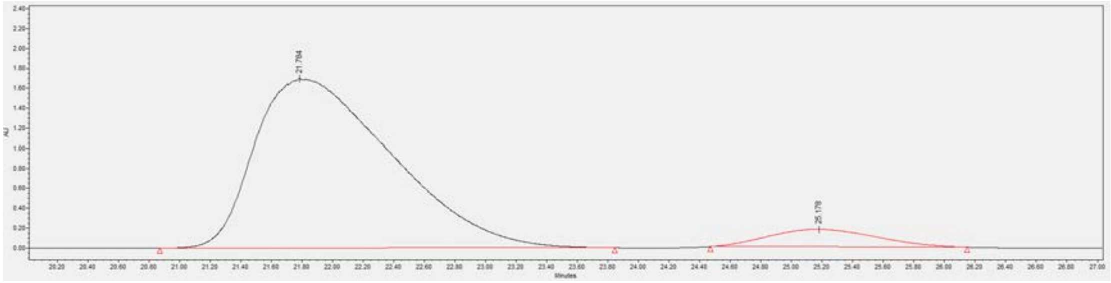

	Name	Retention Time (min)	Area (µV*sec)	% Area	Height (µV)	Int Type	Amount	Units	Peak Type	Peak Codes
1		21.784	105397477	92.47	1698777	bb			Unknown	
2		25.178	8584224	7.53	175711	bb			Unknown	

Figure S118. HPLC spectrum for **2u**

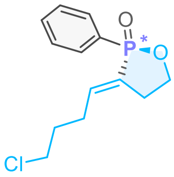

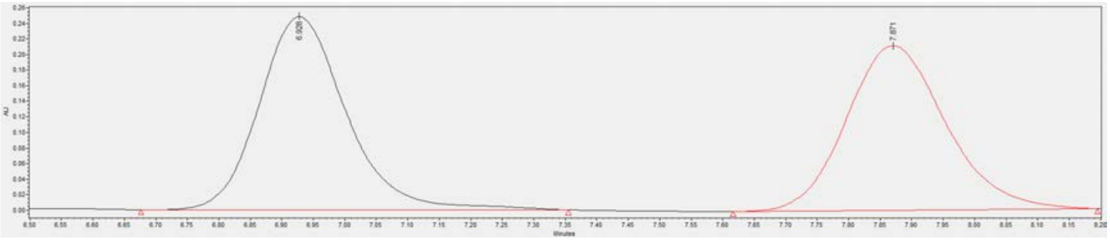

	Name	Retention Time (min)	Area (μV*sec)	% Area	Height (μV)	Int Type	Amount	Units	Peak Type	Peak Codes
1		6.928	2506268	50.79	251105	VV			Unknown	
2		7.871	2427931	49.21	213288	VB			Unknown	

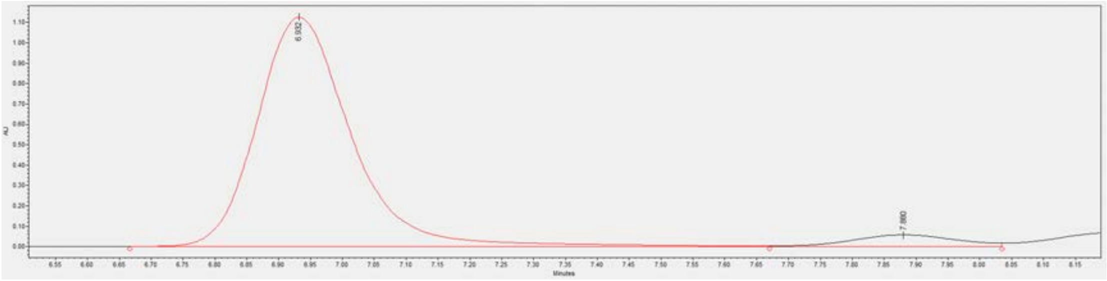

	Name	Retention Time (min)	Area (μV*sec)	% Area	Height (μV)	Int Type	Amount	Units	Peak Type	Peak Codes
1		6.932	11468212	94.79	1124210	VV			Unknown	
2		7.880	630815	5.21	56592	VV			Unknown	

Figure S119. HPLC spectrum for **2v**

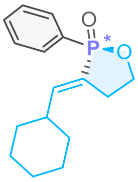

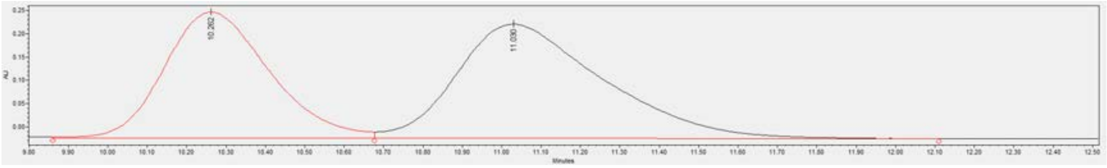

E	Name	Retention Time (min)	Area (µV*sec)	% Area	Height (µV)	Int Type	Amount	Units	Peak Type	Peak Codes
1		10.262	5036403	44.09	271252	VV			Unknown	
2		11.030	6385433	55.91	246281	VV			Unknown	

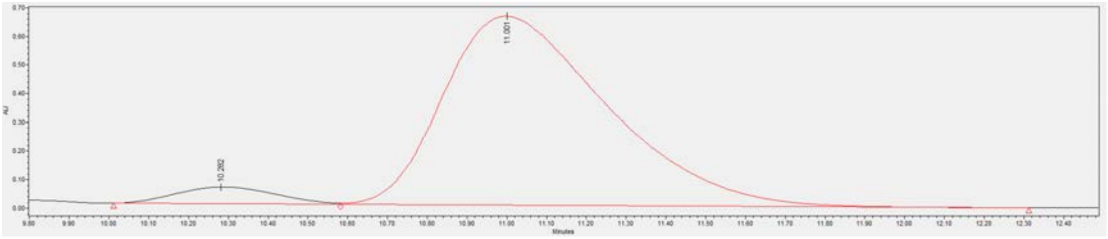

E	Name	Retention Time (min)	Area (µV*sec)	% Area	Height (µV)	Int Type	Amount	Units	Peak Type	Peak Codes
1		10.282	1018154	5.12	58567	bV			Unknown	
2		11.001	18856191	94.88	659406	Vb			Unknown	

Figure S120. HPLC spectrum for **2w**

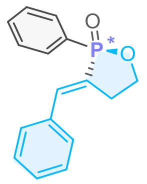

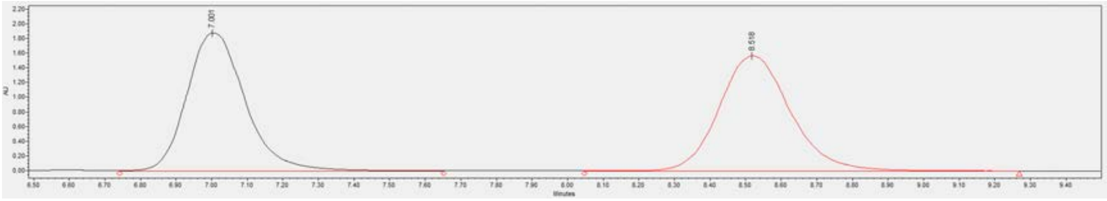

	Name	Retention Time (min)	Area (µV*sec)	% Area	Height (µV)	Int Type	Amount	Units	Peak Type	Peak Codes
1		7.001	21267917	49.49	1876245	VV			Unknown	
2		8.518	21709134	50.51	1563308	VB			Unknown	

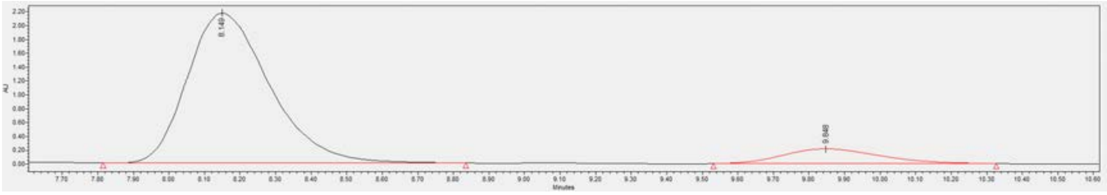

	Name	Retention Time (min)	Area (µV*sec)	% Area	Height (µV)	Int Type	Amount	Units	Peak Type	Peak Codes
1		8.149	34856760	89.62	2163336	bb			Unknown	
2		9.848	4037341	10.38	213688	bb			Unknown	

Figure S121. HPLC spectrum for **2x**

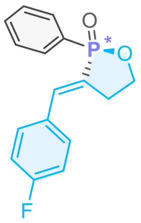

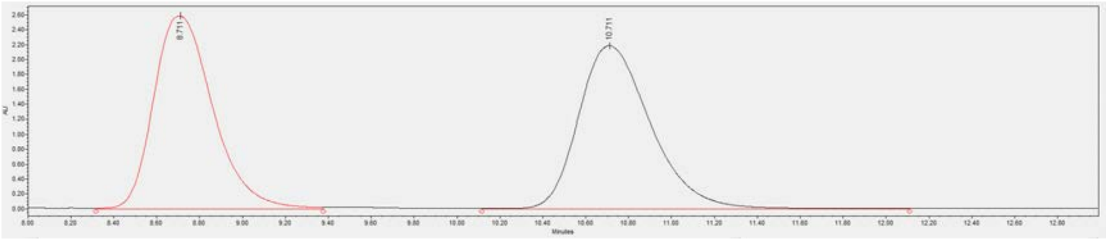

	Name	Retention Time (min)	Area (µV*sec)	% Area	Height (µV)	Int Type	Amount	Units	Peak Type	Peak Codes
1		8.711	49643028	49.38	2588359	VV			Unknown	
2		10.711	50886299	50.62	2190087	VV			Unknown	

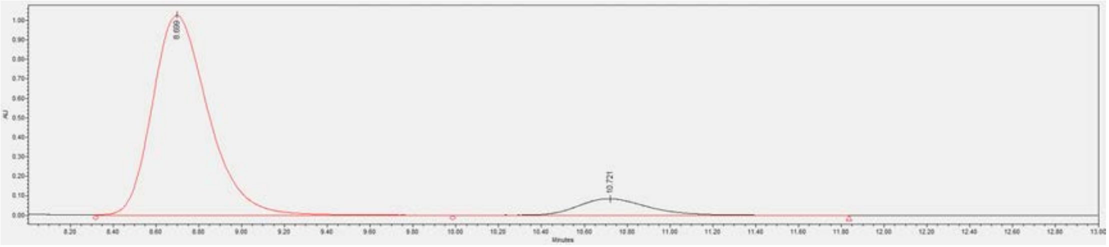

	Name	Retention Time (min)	Area (µV*sec)	% Area	Height (µV)	Int Type	Amount	Units	Peak Type	Peak Codes
1		8.699	18668600	90.39	1027348	VV			Unknown	
2		10.721	1985173	9.61	85698	VB			Unknown	

Figure S122. HPLC spectrum for **2y**

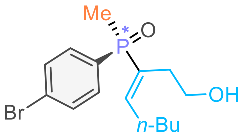

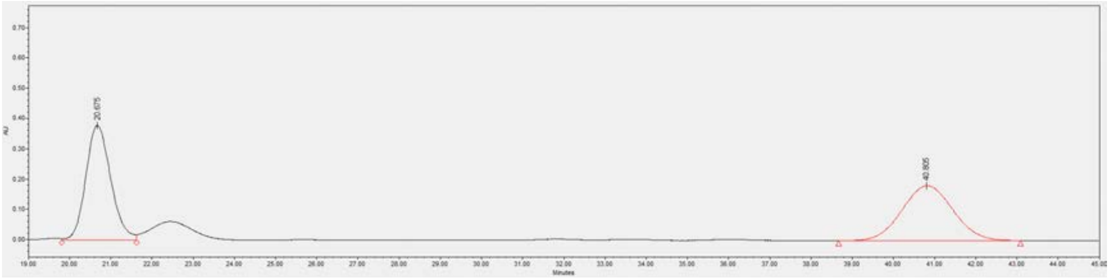

	Name	Retention Time (min)	Area (µV*sec)	% Area	Height (µV)	Int Type	Amount	Units	Peak Type	Peak Codes
1		20.675	15990982	50.35	379283	VV			Unknown	
2		40.805	15766041	49.65	182757	BB			Unknown	

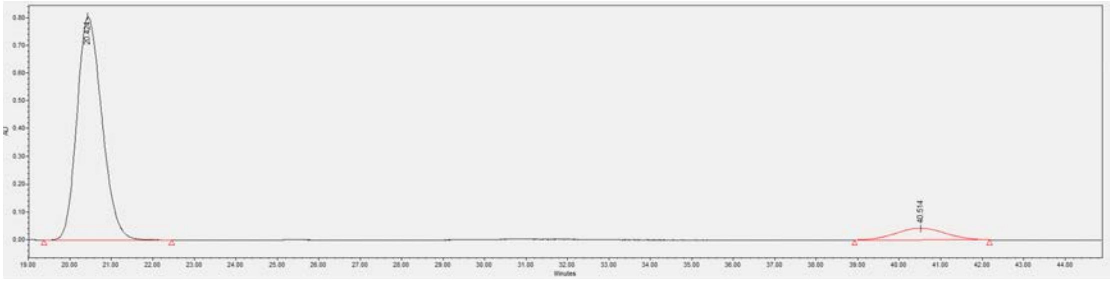

	Name	Retention Time (min)	Area (µV*sec)	% Area	Height (µV)	Int Type	Amount	Units	Peak Type	Peak Codes
1		20.424	33777043	90.53	805120	bb			Unknown	
2		40.514	3531862	9.47	42458	bb			Unknown	

Figure S123. HPLC spectrum for **3**

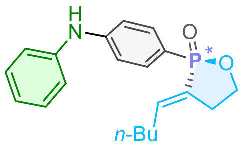

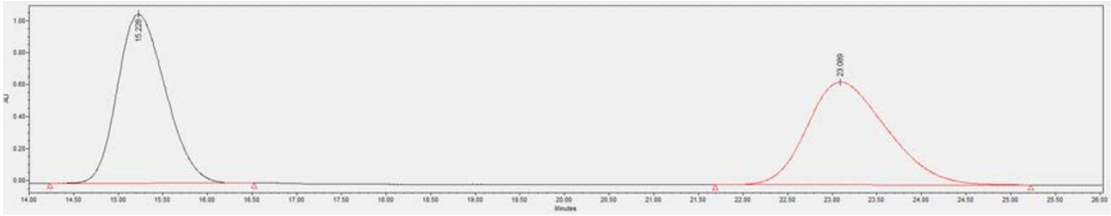

E	Name	Retention Time (min)	Area (µV*sec)	% Area	Height (µV)	Int Type	Amount	Units	Peak Type	Peak Codes
1		15.228	39616690	49.75	1058093	bb			Unknown	
2		23.089	40021994	50.25	643272	bb			Unknown	

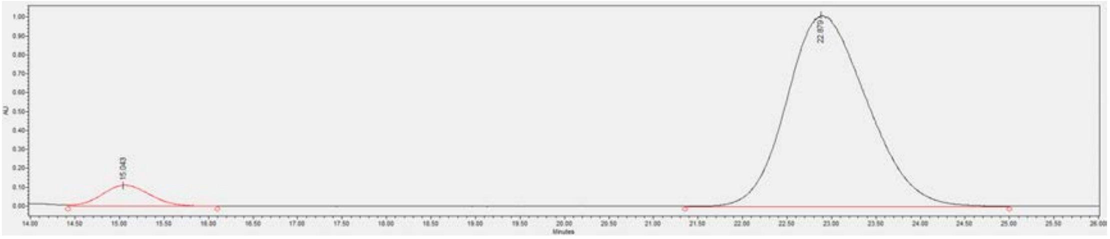

E	Name	Retention Time (min)	Area (µV*sec)	% Area	Height (µV)	Int Type	Amount	Units	Peak Type	Peak Codes
1		15.043	4249269	6.28	112165	VV			Unknown	
2		22.879	63426480	93.72	1011493	VV			Unknown	

Figure S124. HPLC spectrum for **5**

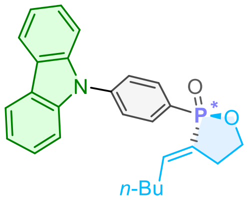

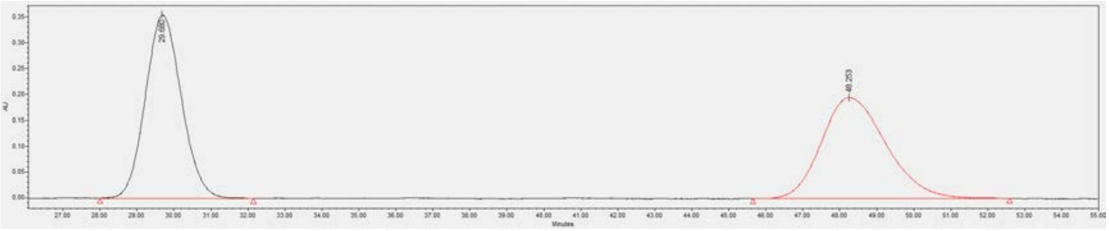

	Name	Retention Time (min)	Area (µV*sec)	% Area	Height (µV)	Int Type	Amount	Units	Peak Type	Peak Codes
1		29.680	24088508	50.38	355163	bb			Unknown	
2		48.253	23727139	49.62	195585	bb			Unknown	

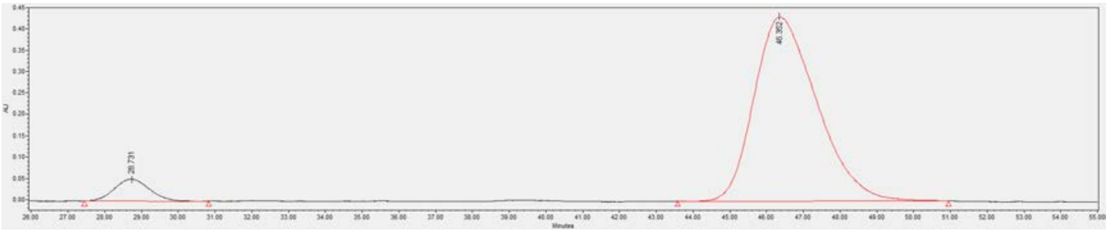

	Name	Retention Time (min)	Area (µV*sec)	% Area	Height (µV)	Int Type	Amount	Units	Peak Type	Peak Codes
1		28.731	3403885	6.24	51537	bb			Unknown	
2		46.352	51168727	93.76	431634	bb			Unknown	

Figure S125. HPLC spectrum for **6**

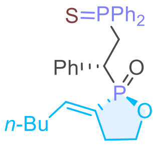

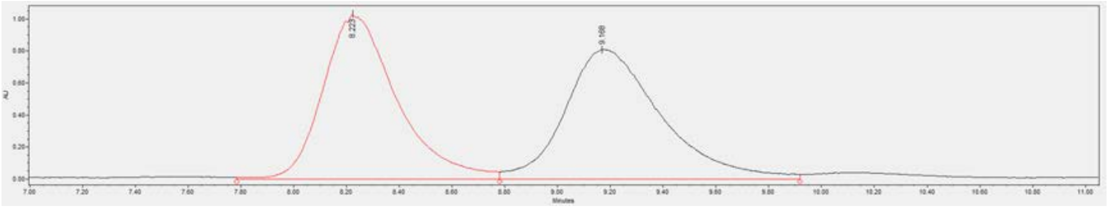

	Name	Retention Time (min)	Area (µV*sec)	% Area	Height (µV)	Int Type	Amount	Units	Peak Type	Peak Codes
1		8.223	19980627	49.44	1032429	VV			Unknown	
2		9.168	20432899	50.56	810251	VV			Unknown	

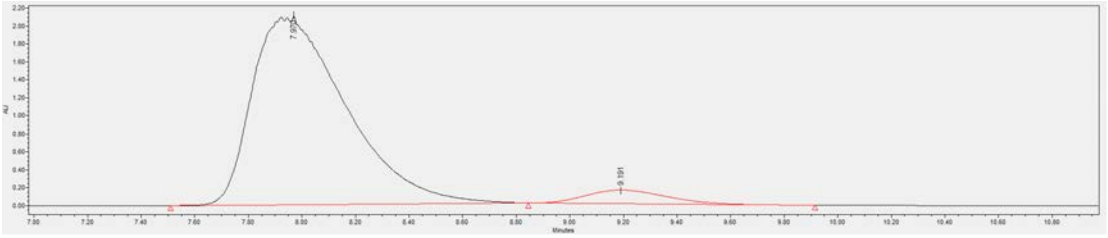

	Name	Retention Time (min)	Area (µV*sec)	% Area	Height (µV)	Int Type	Amount	Units	Peak Type	Peak Codes
1		7.970	50893224	94.14	2107717	bb			Unknown	
2		9.191	3165655	5.86	151964	bb			Unknown	

Figure S126. HPLC spectrum for **7a**

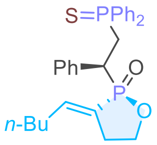

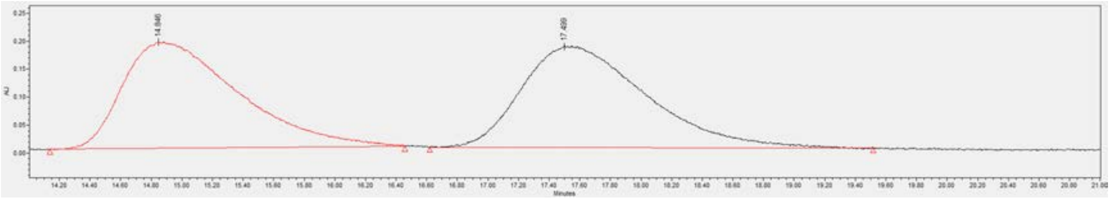

	Name	Retention Time (min)	Area (µV*sec)	% Area	Height (µV)	Int Type	Amount	Units	Peak Type	Peak Codes
1		14.846	9958716	49.43	190542	bb			Unknown	
2		17.499	10186511	50.57	180956	bb			Unknown	

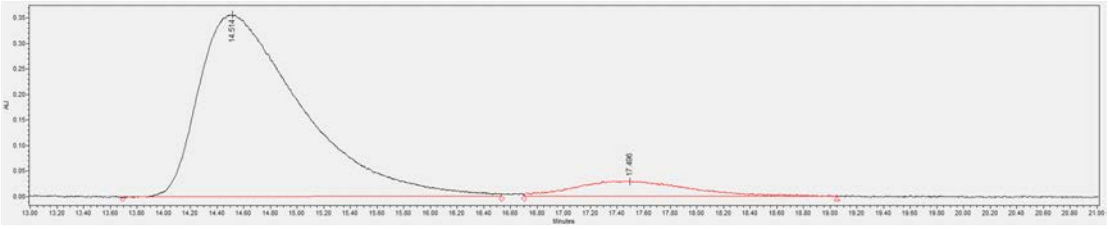

	Name	Retention Time (min)	Area (µV*sec)	% Area	Height (µV)	Int Type	Amount	Units	Peak Type	Peak Codes
1		14.514	18994503	91.75	356033	VV			Unknown	
2		17.496	1708754	8.25	29191	Vb			Unknown	

Figure S127. HPLC spectrum for **7b**

11. Calculation Data

All calculations were performed using Gaussian 16, Revision A.03 package.[5] All of the reactants, intermediates, transition states, products were optimized by the DFT with the ωB97X-D functional.[6] For geometry optimizations and frequency calculations, BS-I basis set system was employed. In BS-I, we employed LANL2DZ basis set for Rh with effective core potentials, 6-31G(d) basis sets for C, H, O, P, F, and Cl. All the stationary structures were characterized with no imaginary frequency and the transition state structures (TSs) were characterized with a single imaginary frequency. Intrinsic reaction coordinate (IRC) calculations were performed on the TSs. For single point energy calculation and time-dependent density functional theory (TD-DFT) calculation, a better basis system BS-II was used. In BS-II, we employed SDD basis set for Rh with effective core potentials, 6-311++G(d,p) basis sets for C, H, O, P, F, and Cl. The solvent effect of toluene was evaluated through the SMD method.[7] All reported energies are free energies at a concentration of 1 M and a temperature of 298.15 K. ECD spectra of (S)-2a were performed using Multiwfn 3.8.[8]

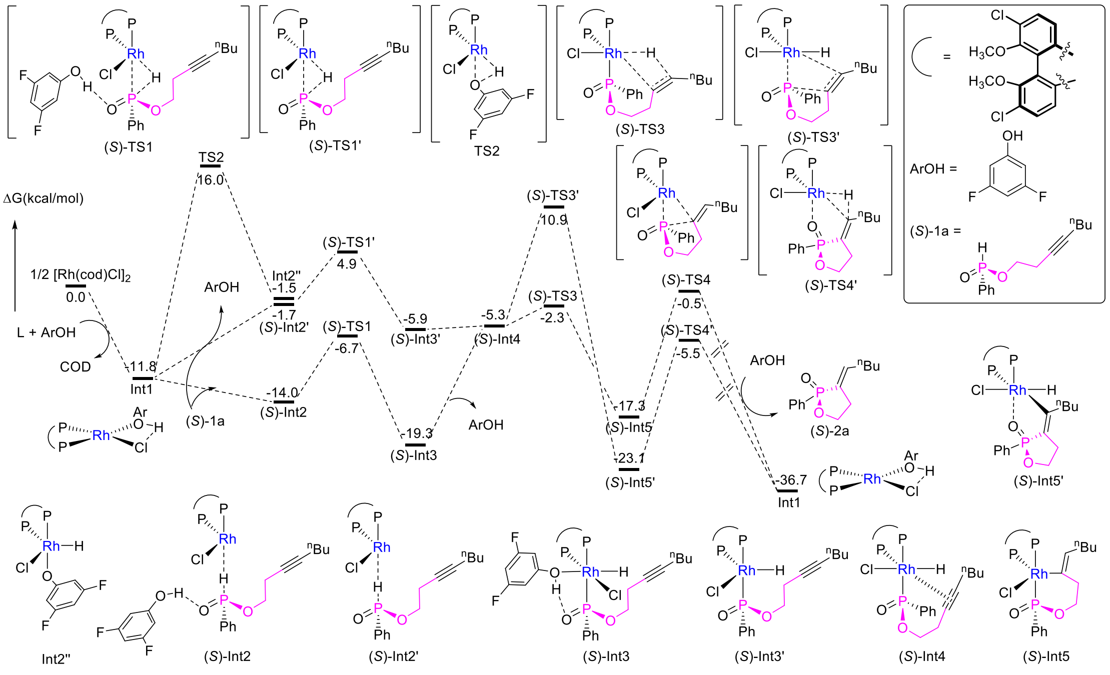

Figure S128. DFT-calculated reaction pathway for synthesis of substrate **(S)-1a**

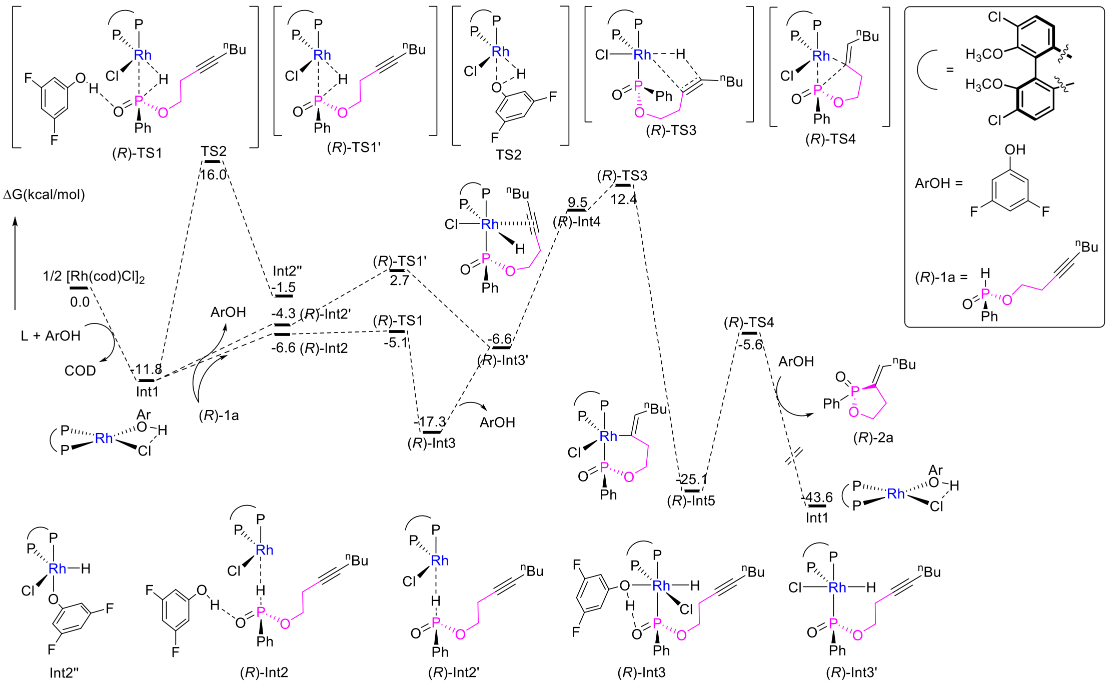

Figure S129. DFT-calculated reaction pathway for synthesis of substrate **(R)-1a**

Cartesian coordinates of the optimized structures:

1/2[Rh(cod)Cl]2

E = -1763.47010058 a.u.

0 1

C	-2.10915600	-6.29958600	11.39274500
H	-2.22802000	-7.13102400	12.09483200
C	-0.62356000	-5.94159000	11.21642300
C	-2.76047100	-6.65313900	10.06364000
H	-3.45967000	-5.91746800	9.66992700
C	-2.87730000	-7.96035400	9.56285900
H	-3.67141800	-8.13720100	8.83798100
C	-2.32973900	-9.21166200	10.21786900
H	-2.98642300	-9.53260700	11.03949700
C	-0.88109700	-9.04189100	10.70887400
C	0.03373900	-6.70549000	10.08680000
H	0.89622000	-6.20667300	9.64748800
C	-0.09643000	-8.07978200	9.82767800
H	0.66812800	-8.53092600	9.19693900
H	-0.85982000	-8.69103400	11.74584800
H	-0.38202900	-10.01582000	10.70951600
H	-0.07006300	-6.09038300	12.15486100
H	-0.54064100	-4.87646900	10.97167400
C	-0.27187400	-2.11233500	8.66125700
H	0.27574300	-2.06560300	9.60801000
H	-0.14659300	-1.13164100	8.19134100
C	-1.75700900	-2.39780200	8.94371500
C	0.34695100	-3.18713500	7.77938800
H	1.04871000	-3.85662600	8.27380900

C	0.43483700	-3.10936300	6.37931500
H	1.21198300	-3.70769600	5.90451500
C	-0.11584200	-1.99152400	5.51793200
H	-0.11328400	-2.34965500	4.48313600
H	0.55347900	-1.11949900	5.54810000
C	-1.55149200	-1.59399700	5.90425400
C	-2.44647600	-3.10789300	7.79823300
H	-3.31068400	-3.70203100	8.09119100
C	-2.34085000	-2.78204500	6.43619100
H	-3.12351000	-3.16292400	5.78178500
H	-1.54641700	-0.79275300	6.65054000
H	-2.29246000	-1.47230800	9.20047100
H	-1.83327400	-3.05417300	9.81810500
Rh	-1.10506600	-4.45776000	6.88940100
Rh	-1.34336900	-6.96323500	8.51023800
Cl	-2.88886400	-6.15796100	6.76061600
Cl	0.41491800	-6.40357200	6.87222900
H	-2.06354200	-1.18659900	5.02710000
H	-2.35876200	-10.00853100	9.46741600
H	-2.63777400	-5.45030400	11.83738400

L

E = -3219.011987 a.u.

0 1

C	0.03683000	-0.74526200	-0.03585900
C	-0.79868800	0.36152900	0.10704100
H	2.04121600	-1.45785300	-0.31680100
C	1.40778100	-0.58427700	-0.20730000
C	-0.24792200	1.65446600	0.06320100

C	1.13546200	1.82323200	-0.08126800
C	1.94655800	0.69329000	-0.22208800
H	3.01858700	0.81308300	-0.34213400
C	-1.18525400	2.81531700	0.12418400
C	-1.37185800	3.57560600	-1.04310900
C	-1.90380100	3.12614900	1.28618300
C	-2.30781500	4.60904900	-1.04655100
C	-2.83416200	4.16881700	1.25159600
C	-3.04565500	4.90418500	0.09483300
H	-3.40067500	4.41331100	2.14453000
H	-3.76876400	5.71226000	0.07239000
P	1.83424100	3.53549700	-0.01385200
P	-1.53022700	2.18453100	2.83407800
C	-3.19903800	2.15967800	3.60991500
C	-4.04276500	1.10977800	3.22524800
C	-3.66784700	3.10249200	4.52995600
C	-5.33308100	1.01603900	3.73641300
H	-3.67793400	0.36546100	2.52120900
C	-4.95663500	3.00098300	5.04917800
H	-3.02241800	3.91745000	4.84541900
C	-5.79232400	1.96116600	4.65135700
H	-5.97822600	0.19822500	3.42829800
H	-5.30824200	3.73879900	5.76479200
H	-6.79684000	1.88386100	5.05719500
C	-0.62897700	3.45272000	3.81483300
C	-0.34668800	3.14985300	5.15435500
C	-0.09268200	4.62054700	3.26583900
C	0.43121000	4.00253800	5.92825700
H	-0.74312100	2.23814800	5.59620900

C	0.70510600	5.46364200	4.03608800
H	-0.27749500	4.87178700	2.22582200
C	0.96573300	5.16094000	5.36743400
H	0.63196400	3.75563400	6.96693900
H	1.13217000	6.35393200	3.58481800
H	1.59141000	5.81798300	5.96419800
C	2.74056900	3.49149700	1.58614200
C	3.60534500	4.55788500	1.86925600
C	2.50214600	2.54145900	2.58305200
C	4.23330100	4.65689200	3.10524100
H	3.79307200	5.31588400	1.11183000
C	3.11649800	2.65212400	3.82814400
H	1.82100800	1.71582200	2.40090700
C	3.98643100	3.70414700	4.09152000
H	4.90820300	5.48502800	3.30226700
H	2.90332400	1.91464000	4.59581600
H	4.46283500	3.78841700	5.06384200
C	3.18106200	3.38587100	-1.26096500
C	2.82867900	3.66170300	-2.58832000
C	4.49865400	3.01475200	-0.97432400
C	3.76914200	3.55046600	-3.60750700
H	1.80953600	3.96507700	-2.81546000
C	5.44157300	2.91380700	-1.99467600
H	4.79199400	2.80884800	0.05124200
C	5.07856700	3.17740300	-3.31253200
H	3.48223100	3.76558200	-4.63289700
H	6.46210100	2.62650500	-1.75785900
H	5.81520700	3.09824000	-4.10678400
Cl	-2.54702000	5.54400000	-2.49631100

Cl	-0.64616600	-2.34656300	0.00894200
O	-0.59477900	3.36724900	-2.14525300
O	-2.12594700	0.17477600	0.36337400
C	-2.99577700	0.24565200	-0.75528300
H	-2.68043800	-0.44984600	-1.54258000
H	-3.98265600	-0.04262700	-0.39060100
H	-3.04388300	1.26484100	-1.15619800
C	-1.01984500	2.34369900	-3.03097700
H	-2.05296200	2.51233200	-3.35838900
H	-0.35278000	2.39324900	-3.89274100
H	-0.93310500	1.35491300	-2.56582200

ArOH

E = -505.765334356 a.u.

0 1

C	-0.81488900	0.92734400	-0.00280400
C	0.57178400	0.89663700	-0.01343900
C	1.25687500	2.11023700	-0.02062200
C	0.57172500	3.32384400	-0.01492800
C	-0.81491000	3.29309200	-0.00419200
C	-1.53981600	2.11019100	-0.00065200
H	1.10786700	-0.04442300	-0.00129100
H	1.10786200	4.26488600	-0.00400100
H	-2.62228600	2.11021700	0.00950800
O	2.63179500	2.11040500	0.01906600
H	2.97563200	2.10781100	-0.88232000
F	-1.48504700	4.45190500	0.00156300
F	-1.48491000	-0.23152500	0.00435000

COD

E = -311.928954704 a.u.

0 1

C	-2.04038300	-6.29118100	11.48401100
H	-1.93827000	-7.06743400	12.24505900
C	-0.65398500	-5.84822400	10.98546800
C	-2.96873000	-6.72323800	10.37372000
H	-3.64237600	-5.94551400	10.01457500
C	-3.04971900	-7.90643600	9.76105000
H	-3.79979100	-7.99221800	8.97422700
C	-2.26824700	-9.17695300	9.97550200
H	-2.92772800	-9.91868400	10.45051600
C	-0.95929700	-9.09143100	10.77937000
C	0.18411500	-6.85552100	10.24123200
H	1.01292700	-6.40380000	9.69526300
C	0.07197900	-8.18274700	10.15320300
H	0.80384600	-8.68939000	9.52444800
H	-1.16809100	-8.80764200	11.81287400
H	-0.54081100	-10.10341300	10.83205800
H	-0.07345200	-5.47250600	11.84135500
H	-0.78785800	-4.98064900	10.32437600
H	-2.03597500	-9.59030100	8.98410200
H	-2.49244100	-5.43465300	11.99784300

(S)-1a

E = -1036.45371918 a.u.

0 1

P	-0.34256500	-0.04863100	0.69938600
C	0.54110100	-1.60692600	0.86253500

C	1.72389300	-1.71434900	1.60016400
C	0.02275100	-2.72233800	0.20186400
C	2.38389700	-2.93558800	1.67362900
H	2.11894900	-0.84978800	2.12578600
C	0.68632600	-3.94366600	0.27982200
H	-0.90161200	-2.62410000	-0.35956900
C	1.86523200	-4.04858800	1.01279400
H	3.30037600	-3.02251100	2.24927800
H	0.28230500	-4.81273800	-0.23021800
H	2.38280400	-5.00156700	1.07252100
O	-0.26453700	0.50224600	2.21959000
C	-0.82258500	1.79186900	2.49111300
H	-0.33834300	2.55023100	1.86140500
H	-1.89355000	1.78547500	2.26798500
C	-0.57656700	2.09400600	3.96617900
H	0.50097100	2.04676100	4.16359600
H	-1.04741400	1.30873200	4.56887600
C	-1.10676900	3.40781800	4.33124900
C	-1.55933700	4.49440400	4.59709200
C	-2.09645100	5.82297000	4.89696600
H	-1.55127000	6.24295400	5.75096700
H	-3.14423300	5.73077100	5.21339700
C	-2.00970800	6.77768300	3.69341300
H	-2.49746800	6.30109300	2.83526400
H	-0.95488800	6.91027400	3.42099200
C	-2.65401400	8.14249200	3.95295500
H	-2.62778900	8.71909000	3.02031200
H	-3.71581200	8.00149400	4.19651200
C	-1.97850800	8.95256500	5.06048200

H	-2.42080600	9.95085500	5.14072500
H	-2.08007200	8.47101400	6.03915900
H	-0.90778300	9.07533100	4.85819400
H	0.53267100	0.84135500	0.03481300
O	-1.68058800	-0.13951100	0.06725900

(R)-1a

E = -1036.45140012 a.u.

0 1

P	-0.48318300	-0.14416500	0.67901300
H	-1.81258400	-0.61550600	0.59349600
O	-0.12550100	0.79305700	-0.41392400
C	0.48420800	-1.64899800	0.87224000
C	1.70660200	-1.63421700	1.55020300
C	0.02913700	-2.83105900	0.28500400
C	2.46418800	-2.79629000	1.63808200
H	2.05437200	-0.71747800	2.01589400
C	0.78987600	-3.99323300	0.37428900
H	-0.92175400	-2.84889400	-0.24258700
C	2.00681000	-3.97447300	1.04998700
H	3.41167700	-2.78522700	2.16805600
H	0.43255600	-4.91137000	-0.08166500
H	2.60072500	-4.88087900	1.12075500
O	-0.38297700	0.45192600	2.17999500
C	-0.94829500	1.74318300	2.43410500
H	-0.53966600	2.47357300	1.73002800
H	-2.03714900	1.70180200	2.30443000
C	-0.59906700	2.11515800	3.87197800
H	0.49210900	2.11890300	3.97876600

H	-0.98398600	1.33930100	4.54421700
C	-1.15214400	3.42175400	4.22900500
C	-1.62736700	4.49980000	4.49012200
C	-2.17851300	5.82347200	4.78634600
H	-1.81286000	6.14891300	5.76788500
H	-3.27155700	5.75447300	4.87103900
C	-1.81479100	6.86580100	3.71455000
H	-2.12567400	6.48218400	2.73600400
H	-0.72305900	6.97010600	3.67732100
C	-2.45936100	8.23404000	3.95460900
H	-2.22288900	8.88097800	3.10110100
H	-3.55232600	8.12371700	3.96231200
C	-2.00583100	8.92141400	5.24341100
H	-2.42688000	9.92923900	5.31888800
H	-2.32152000	8.36814200	6.13466300
H	-0.91344400	9.01091600	5.27555700

Int1

E = -4294.61963681 a.u.

0 1

Rh	7.69501600	-9.79321800	5.87968100
C	3.41516400	-12.94454800	6.35210800
C	2.29588400	-12.13828900	6.14256100
C	4.63205200	-12.36093800	6.73846600
C	2.35387500	-10.77360700	6.38206300
C	3.53445700	-10.21295900	6.84629400
C	4.68446600	-10.98591700	7.02553200
H	1.47305200	-10.16033400	6.22751200
H	3.55248000	-9.15197600	7.06485100

C	5.83019000	-13.25054800	6.79288700
C	5.86068700	-14.25372000	7.77789200
C	6.88691200	-13.12194500	5.87185300
C	6.96671400	-15.10768600	7.84378900
C	7.94179600	-14.03581300	5.92740900
C	7.98561100	-15.01710100	6.90683900
H	8.75902700	-13.97391800	5.21886100
H	8.82593700	-15.70040600	6.96386600
P	7.04549900	-11.62972300	4.78239100
P	6.26578700	-10.15985300	7.52087300
C	8.37098200	-12.07768000	3.58973000
C	8.14130700	-12.48343600	2.27230800
C	9.69316500	-11.90910400	4.02365900
C	9.21020600	-12.72838000	1.41333300
H	7.12967000	-12.57882500	1.89465900
C	10.75924700	-12.16428300	3.16915900
H	9.88294300	-11.55697100	5.03498500
C	10.51929800	-12.57398400	1.85979100
H	9.01524000	-13.03276800	0.38939800
H	11.77642600	-12.02527100	3.52194600
H	11.35029500	-12.76136700	1.18640000
C	5.51913900	-11.55698200	3.77732000
C	4.98811100	-12.66949100	3.11864500
C	4.88550900	-10.32000700	3.64315200
C	3.84028700	-12.54837900	2.34306600
H	5.47709700	-13.63531700	3.21160900
C	3.73366900	-10.19879700	2.87161200
H	5.30315000	-9.45396000	4.15086400
C	3.20808800	-11.31207000	2.22397700

H	3.43695700	-13.41810600	1.83238500
H	3.25103100	-9.23101100	2.77732400
H	2.30787300	-11.21938600	1.62409400
C	6.88129700	-11.17995100	8.90311500
C	6.02330500	-11.76840400	9.83677300
C	8.25888600	-11.39860500	8.99631900
C	6.53709100	-12.57380800	10.84693600
H	4.94865700	-11.63778300	9.74924000
C	8.76881800	-12.20646000	10.00732800
H	8.91917900	-10.93843900	8.26618600
C	7.90890800	-12.79697600	10.92996200
H	5.86201400	-13.04777200	11.55226000
H	9.83874800	-12.38003100	10.06981400
H	8.30679500	-13.44001600	11.70932500
C	5.67388100	-8.59260000	8.26464000
C	5.46251400	-7.50336200	7.41098700
C	5.40606500	-8.45265500	9.62694200
C	4.96682100	-6.30418900	7.90695800
H	5.70283400	-7.59832600	6.35552500
C	4.92225100	-7.24506800	10.12496000
H	5.58797500	-9.27650000	10.30898100
C	4.69636500	-6.17322900	9.26753500
H	4.80888600	-5.46734500	7.23343100
H	4.72660600	-7.14345700	11.18823400
H	4.32043000	-5.23273100	9.65939000
Cl	8.84287300	-7.92541600	6.94288000
C	8.00832900	-8.29321500	3.32765000
C	7.26244300	-7.18101300	3.72344100
C	6.23819800	-6.76517500	2.88868700

C	5.93086100	-7.39472400	1.69064500
C	6.71736100	-8.48224400	1.33922700
C	7.76070300	-8.94570100	2.12329700
H	7.45206500	-6.68270500	4.66799600
H	5.12187700	-7.05119000	1.05910700
H	8.35285100	-9.80051900	1.82229800
O	8.98787500	-8.81893100	4.12903500
H	9.23909600	-8.17867300	4.83813600
F	6.45250700	-9.11481600	0.18919500
F	5.49479700	-5.71534800	3.26605900
Cl	7.11964800	-16.26451800	9.13947200
Cl	0.80579500	-12.86111300	5.60581700
O	4.85309600	-14.27945800	8.67734100
O	3.28540800	-14.29893900	6.28105900
C	4.08936100	-15.47254900	8.83374600
H	4.33122700	-15.94318800	9.79017100
H	3.03768200	-15.18198000	8.80957000
H	4.27921200	-16.17627300	8.01906300
C	3.57449500	-14.94248500	5.05262400
H	3.24494400	-15.97603300	5.17311800
H	3.03098900	-14.47880600	4.22352500
H	4.65098400	-14.92586000	4.84900600

TS2

E = -4294.56007969 a.u.

0 1

Rh	7.47133400	-9.55214400	5.97397500
C	3.25967000	-12.69189400	6.62263200
C	2.13257800	-11.86875300	6.64932100

C	4.52080200	-12.16057200	6.93658300
C	2.23418200	-10.54310700	7.04438500
C	3.46849800	-10.03959900	7.42819100
C	4.62023500	-10.82983100	7.37916100
H	1.34814700	-9.91859900	7.07388100
H	3.52810700	-9.01246400	7.76888100
C	5.70206900	-13.06488900	6.79497500
C	5.78433900	-14.15504000	7.68044300
C	6.69202800	-12.87245900	5.81151900
C	6.87736600	-15.02387600	7.59373700
C	7.72841700	-13.80351200	5.70886000
C	7.82699300	-14.86484000	6.59636600
H	8.48656800	-13.69823600	4.94328900
H	8.65630800	-15.56054900	6.53070900
P	6.73408800	-11.34078600	4.76480600
P	6.24556000	-10.06279000	7.79990700
C	8.05832300	-11.65628400	3.53967900
C	7.84307500	-11.84751100	2.17427700
C	9.37662900	-11.59721700	4.01691600
C	8.92176000	-12.01812400	1.31103600
H	6.84176400	-11.81887300	1.76255800
C	10.45019500	-11.77079200	3.15301100
H	9.56722400	-11.40296700	5.07012100
C	10.22384300	-11.98843600	1.79623500
H	8.73796700	-12.14415600	0.24893700
H	11.46335100	-11.71691400	3.53870900
H	11.06113600	-12.10480200	1.11555200
C	5.14640600	-11.35154900	3.86699900
C	4.79631200	-12.40086500	3.01171600

C	4.25227900	-10.29534500	4.05014400
C	3.58669100	-12.37421700	2.32564600
H	5.46877000	-13.24458900	2.88196000
C	3.02974900	-10.28316200	3.38790500
H	4.51472800	-9.48558000	4.72408100
C	2.69969400	-11.31749300	2.51715300
H	3.33129400	-13.18583500	1.65096300
H	2.33883100	-9.46173400	3.54803500
H	1.75116900	-11.30221300	1.98949700
C	7.01421700	-11.21982700	8.98110000
C	6.24998500	-11.95293500	9.89394200
C	8.40069700	-11.39646000	8.94155400
C	6.86387900	-12.85955000	10.75044400
H	5.16834500	-11.85884900	9.90349000
C	9.00966300	-12.30733100	9.79860900
H	9.00004700	-10.80694000	8.25387900
C	8.24308700	-13.04151200	10.69973300
H	6.25900600	-13.44283400	11.43706500
H	10.08600300	-12.44306600	9.75961300
H	8.71926600	-13.76158900	11.35828400
C	5.75570500	-8.58378700	8.75789300
C	5.61478400	-7.36108600	8.09364000
C	5.46276300	-8.66102400	10.12146100
C	5.16668400	-6.23839400	8.78035600
H	5.88176700	-7.28304000	7.04470600
C	5.02355200	-7.53280400	10.80787100
H	5.59121400	-9.59420000	10.65935300
C	4.86939800	-6.32267900	10.13817100
H	5.06921200	-5.29289400	8.25618700

H	4.80804700	-7.60084700	11.86984300
H	4.52964200	-5.44274500	10.67626600
Cl	8.83436600	-8.16620000	7.32912500
C	7.94223600	-8.49940000	2.97529500
C	6.70314400	-8.56993100	2.31435100
C	6.67576900	-8.76586800	0.94449800
C	7.81961500	-8.89169900	0.17229300
C	9.02825500	-8.80409900	0.85443600
C	9.12203100	-8.61150900	2.21753400
H	5.77406100	-8.46271900	2.86053700
H	7.77599200	-9.04620100	-0.89789400
H	10.08577700	-8.56692300	2.70889600
O	8.04649200	-8.29726500	4.28195800
H	6.70757600	-8.58919600	5.01264400
F	10.16229200	-8.94325600	0.14449500
F	5.47926100	-8.84635000	0.33378900
Cl	7.10033200	-16.28930300	8.76991700
Cl	0.58139600	-12.52379000	6.21268600
O	4.84397200	-14.25199700	8.64525500
O	3.10898900	-14.02337400	6.38832600
C	4.06960500	-15.44589100	8.74607900
H	4.37127600	-16.00691000	9.63403400
H	3.02523700	-15.14025500	8.82755000
H	4.18562500	-16.07044200	7.85678800
C	3.23689600	-14.47469000	5.04961800
H	2.92430000	-15.52026800	5.05527600
H	2.59465100	-13.89980400	4.37539400
H	4.27711000	-14.40312000	4.71466500

Int2”

E = -4294.58482921 a.u.

0 1

Rh	7.42693600	-9.51806200	5.90430000
C	3.25999200	-12.72025100	6.62147300
C	2.11835500	-11.91806500	6.62401500
C	4.50994800	-12.15933300	6.93398100
C	2.19301700	-10.58443400	6.99821600
C	3.41338800	-10.05432200	7.38932800
C	4.58078900	-10.82355700	7.36342300
H	1.29600800	-9.97521300	7.00957800
H	3.44868200	-9.02270500	7.71934900
C	5.70307500	-13.04940300	6.80675300
C	5.79035800	-14.12407200	7.71124600
C	6.68383300	-12.87785000	5.80889300
C	6.87730500	-15.00036100	7.63064700
C	7.70547000	-13.82646700	5.70724800
C	7.80939900	-14.87116200	6.61327700
H	8.44634600	-13.75449700	4.92244100
H	8.63018500	-15.57662400	6.54486700
P	6.72223200	-11.37335000	4.71594000
P	6.18565900	-10.01355400	7.78266600
C	7.99675600	-11.74408800	3.45607000
C	7.72204400	-11.82577400	2.08990000
C	9.32987900	-11.81171400	3.88485300
C	8.75209000	-12.03309700	1.17662700
H	6.71384200	-11.68791800	1.71918100
C	10.35391700	-12.02734000	2.97277500
H	9.57476200	-11.67434000	4.93437400

C	10.06532200	-12.14709600	1.61524500
H	8.52303200	-12.07592000	0.11687100
H	11.38078500	-12.07526800	3.32107800
H	10.86777000	-12.29521300	0.89938200
C	5.11703300	-11.39925300	3.84723500
C	4.78354300	-12.48444000	3.02893000
C	4.20599400	-10.35218100	3.98841800
C	3.57226700	-12.50663600	2.34721200
H	5.47634200	-13.31435400	2.91913000
C	2.98215800	-10.38708300	3.32876200
H	4.44401400	-9.51038500	4.62936400
C	2.66630000	-11.45980800	2.50142400
H	3.33162500	-13.34749400	1.70367100
H	2.28016900	-9.56966900	3.45701200
H	1.71614000	-11.48111700	1.97696700
C	7.01506200	-11.13250000	8.95622300
C	6.29735100	-11.90721600	9.87184300
C	8.40958500	-11.22868400	8.91188300
C	6.96503000	-12.78123300	10.72204300
H	5.21259800	-11.86999100	9.88879200
C	9.07303500	-12.10468300	9.76490100
H	8.97447700	-10.60366600	8.22583100
C	8.35223400	-12.88454800	10.66552500
H	6.39689700	-13.39885900	11.41000400
H	10.15533800	-12.17699000	9.72325500
H	8.87106700	-13.57775700	11.32065600
C	5.64968800	-8.55533900	8.74773800
C	5.44367400	-7.33665600	8.09253300
C	5.37749400	-8.65160100	10.11442900

C	4.95105800	-6.24003400	8.79059700
H	5.69519600	-7.23681900	7.04226800
C	4.89430500	-7.54878700	10.81251800
H	5.55468700	-9.58031200	10.64611100
C	4.67467100	-6.34429600	10.15145900
H	4.80296000	-5.29781100	8.27246000
H	4.69493800	-7.63259400	11.87648300
H	4.30013200	-5.48394100	10.69798700
Cl	8.60867200	-7.95029800	7.23839500
C	8.37218800	-8.73462400	3.14062400
C	7.06462000	-8.54876500	2.64046900
C	6.86133800	-8.44526100	1.27581000
C	7.88494700	-8.49701200	0.34382300
C	9.16797000	-8.63577800	0.86621900
C	9.43821700	-8.74270700	2.21282400
H	6.21956700	-8.47932400	3.31333500
H	7.70280300	-8.41198600	-0.71928900
H	10.45315600	-8.87730100	2.56497800
O	8.65215500	-8.92132700	4.40652200
H	6.17346400	-8.82078600	5.44719400
F	10.19577100	-8.69326000	-0.00226700
F	5.59731300	-8.29699500	0.83149600
Cl	7.11270300	-16.23712800	8.83374600
Cl	0.58289100	-12.60857400	6.18720600
O	4.85826300	-14.19965900	8.68582500
O	3.13541700	-14.05966800	6.42012300
C	4.08713400	-15.39213900	8.82512300
H	4.39305000	-15.92411000	9.72929000
H	3.04219800	-15.08677000	8.90013100

H	4.20242000	-16.04348200	7.95541000
C	3.26486900	-14.54860600	5.09495300
H	2.97297600	-15.59940100	5.13469700
H	2.60768300	-14.00750700	4.40757800
H	4.30167300	-14.46719700	4.75187900

(S)-Int2

E = -5331.1051667 a.u.

0 1

Rh	6.42767500	-9.00347200	4.35379100
C	5.02115600	-13.83713500	5.51262800
C	4.25422800	-14.30170900	4.43923100
C	5.06277800	-12.45396800	5.77701200
C	3.59162100	-13.40905500	3.60707400
C	3.64327300	-12.04946700	3.86397800
C	4.35609400	-11.55887500	4.95978700
H	3.00795800	-13.79026400	2.77634300
H	3.09979500	-11.37491000	3.21364300
C	5.80087700	-12.00845700	7.00028400
C	5.23604100	-12.37313400	8.23691300
C	6.96953900	-11.22981200	6.96558800
C	5.83820100	-11.94186200	9.42095500
C	7.57417500	-10.85814300	8.17064400
C	7.01443900	-11.20811900	9.38930100
H	8.48839300	-10.27622700	8.16581700
H	7.47724800	-10.89927000	10.31987200
P	7.63441600	-10.55377800	5.38092700
P	4.49629200	-9.73542400	5.22866600
C	9.25649800	-9.85565700	5.87937300

C	10.43418900	-10.60419400	5.91687200
C	9.29307200	-8.50918500	6.25560000
C	11.62515500	-10.01558900	6.33559100
H	10.43655300	-11.64268700	5.60355700
C	10.47943000	-7.92574100	6.68307800
H	8.38865100	-7.90919600	6.20241500
C	11.64985800	-8.67921100	6.72356700
H	12.53719800	-10.60481100	6.35123400
H	10.47750100	-6.88110300	6.98049900
H	12.58110600	-8.22424600	7.04822300
C	8.02411200	-12.03793300	4.39082000
C	8.53489000	-13.19764300	4.98296500
C	7.86286000	-11.98266900	3.00381100
C	8.91464600	-14.27629400	4.19076800
H	8.63378400	-13.25813200	6.06417700
C	8.21913500	-13.07533500	2.21846800
H	7.47437800	-11.07774800	2.54525700
C	8.75236200	-14.21786300	2.80806500
H	9.32884400	-15.16751100	4.65369400
H	8.08895200	-13.02593100	1.14196200
H	9.04169800	-15.06411600	2.19186200
C	4.02585200	-9.53404200	6.98412400
C	2.96298300	-10.26023700	7.53249600
C	4.73736900	-8.64476300	7.79030200
C	2.60661500	-10.08970800	8.86419000
H	2.43399600	-10.98775200	6.92373100
C	4.37397800	-8.47096800	9.12374500
H	5.58905300	-8.11464700	7.36836400
C	3.31009400	-9.18972300	9.66035600

H	1.79497900	-10.67453100	9.28472000
H	4.92732100	-7.78426600	9.75658700
H	3.04197100	-9.06301400	10.70476500
C	3.06485800	-9.04506100	4.30735000
C	3.19302400	-8.90187000	2.91842000
C	1.85758600	-8.69092700	4.91757100
C	2.11279700	-8.47092300	2.15190100
H	4.13513000	-9.14006500	2.42767900
C	0.78654100	-8.24767500	4.14941000
H	1.74817400	-8.74765300	5.99410500
C	0.90631900	-8.15156300	2.76665600
H	2.21584200	-8.38798700	1.07611600
H	-0.14345600	-7.97293500	4.63824700
H	0.06508700	-7.81631100	2.16704200
Cl	8.27355500	-8.36802100	2.96818200
C	4.64174400	-8.18777200	-0.13346500
C	4.03846800	-6.94920200	-0.38895400
C	2.96930800	-6.91128200	-1.26765400
C	2.46582500	-8.03350500	-1.91057200
C	3.10503400	-9.23627900	-1.63825500
C	4.17622300	-9.34489100	-0.77026600
H	4.37552600	-6.05070700	0.11254400
H	1.62980800	-7.97335800	-2.59503300
H	4.64293000	-10.30281700	-0.57581000
O	5.66008000	-8.33440600	0.72807600
H	5.79817100	-7.50806900	1.23868200
H	5.93165400	-7.13173200	4.40370200
F	2.65291300	-10.34650700	-2.24222200
F	2.38001000	-5.72819400	-1.50464000

P	5.45698900	-5.94874200	3.74531500
C	3.93604500	-5.57978100	4.61140300
C	2.77145100	-5.30716300	3.89648300
C	3.91842000	-5.63539500	6.00852300
C	1.58727000	-5.06551600	4.58684400
H	2.79621400	-5.30124700	2.81123500
C	2.72821500	-5.41745300	6.69011900
H	4.83438000	-5.84191100	6.55654200
C	1.56558800	-5.12495800	5.97732800
H	0.67690500	-4.85098300	4.03593800
H	2.70699400	-5.47622000	7.77388200
H	0.63579300	-4.94997000	6.51092600
O	6.46367400	-4.85038600	4.33037300
C	7.88674000	-5.02716100	4.21011900
H	8.13773700	-6.09049700	4.14271400
H	8.21686400	-4.53275400	3.29245300
C	8.54055000	-4.38486200	5.42668000
H	8.29724200	-3.31583100	5.44642500
H	9.62776200	-4.46620700	5.30375800
C	8.13107900	-5.00336100	6.68952100
C	7.77992700	-5.49657500	7.73456000
C	7.38716700	-6.09603200	9.01186800
H	6.38524600	-6.52911000	8.90821400
H	7.30016500	-5.30775200	9.77127300
C	8.36975200	-7.17227100	9.49543700
H	9.35510000	-6.71103200	9.64065300
H	8.49593500	-7.92192600	8.70759200
C	7.92269300	-7.86532400	10.78065200
H	7.66505500	-7.11663400	11.54203000

H	7.00673100	-8.43695400	10.58039400
C	9.00119100	-8.79907400	11.32843100
H	8.64383500	-9.36210700	12.19720300
H	9.32403500	-9.51821600	10.56537600
H	9.88873700	-8.23468900	11.63553000
O	5.34687100	-6.06102900	2.26260100
Cl	5.09192100	-12.26661200	10.96320100
Cl	4.00454100	-16.00975200	4.16137900
O	4.06256100	-13.04664700	8.22021800
O	5.66582700	-14.63473200	6.39695100
C	4.00078300	-14.33590900	8.82574200
H	3.40672800	-14.28529100	9.74197600
H	3.52453300	-15.00483200	8.10548900
H	5.00027500	-14.71481000	9.05295100
C	6.47473400	-15.71902100	5.95894300
H	5.95691100	-16.66792300	6.11902500
H	6.74687000	-15.61769800	4.90676900
H	7.38258800	-15.68996300	6.56765600

(S)-Int2’

E = -4825.29640761 a.u.

0 1

Rh	6.55686800	-9.13192300	4.47501200
C	5.07666900	-14.00512200	5.07388800
C	3.96484300	-14.12351400	4.23239400
C	5.36081700	-12.74132400	5.63916900
C	3.12169900	-13.03388100	4.03243200
C	3.38825800	-11.81065200	4.61704100
C	4.52779300	-11.64399500	5.40793000

H	2.25632100	-13.15620900	3.39028200
H	2.71563000	-10.98194200	4.43270400
C	6.59168900	-12.62810300	6.47981200
C	6.54699500	-13.20729000	7.75746500
C	7.74751100	-11.95735400	6.04907100
C	7.65589000	-13.10450400	8.59906200
C	8.85646000	-11.91046400	6.89864600
C	8.81354600	-12.47522700	8.16422700
H	9.76643800	-11.41489700	6.58305600
H	9.67116900	-12.41342400	8.82521600
P	7.75612500	-11.00128500	4.46377000
P	5.04820600	-9.94944100	5.93111000
C	9.54582400	-10.71149600	4.18956900
C	10.34069900	-11.54650400	3.40109500
C	10.14103400	-9.63426000	4.85314700
C	11.71313300	-11.32660700	3.31045200
H	9.89488200	-12.36367900	2.84470700
C	11.51394000	-9.43046700	4.78261400
H	9.51831000	-8.94879900	5.42031700
C	12.30426600	-10.28171800	4.01455000
H	12.31950100	-11.97430900	2.68435600
H	11.95502200	-8.58316600	5.29731400
H	13.37565900	-10.11661300	3.94627900
C	7.25526800	-12.24238900	3.22008500
C	7.70251100	-13.56613300	3.29396700
C	6.38543600	-11.86357600	2.19482800
C	7.28128800	-14.49980100	2.35408300
H	8.35335800	-13.87621600	4.10644100
C	5.95799900	-12.80420100	1.26230100

H	6.04913400	-10.83247800	2.13689200
C	6.40274500	-14.12077900	1.34138700
H	7.62890100	-15.52675400	2.42019100
H	5.27389200	-12.50595400	0.47398900
H	6.06025200	-14.85451200	0.61792200
C	5.37829500	-10.11305100	7.72193800
C	4.52320600	-10.80450300	8.58554100
C	6.55696200	-9.55924900	8.22549400
C	4.82696700	-10.90650300	9.93840900
H	3.63810100	-11.29755200	8.19376600
C	6.86360900	-9.66440800	9.57849900
H	7.24366000	-9.07198300	7.53715300
C	5.99349700	-10.33100900	10.43738700
H	4.16491000	-11.45653200	10.59963900
H	7.78865000	-9.24097900	9.95816000
H	6.23644500	-10.42548000	11.49139400
C	3.49407400	-8.98315100	5.78858600
C	3.19121100	-8.46407100	4.52259200
C	2.61594200	-8.74023000	6.84776000
C	2.02866200	-7.72915200	4.31556900
H	3.87433900	-8.62823000	3.69078100
C	1.44744900	-8.01668100	6.63534700
H	2.85099200	-9.08725500	7.84735600
C	1.14897400	-7.51422700	5.37157200
H	1.82943100	-7.31003600	3.33530500
H	0.77823300	-7.82680900	7.46903200
H	0.24160400	-6.93837700	5.21510000
Cl	7.65809000	-8.33981800	2.52564700
P	5.38569800	-5.83989400	4.89327200

C	4.63958300	-5.83634700	6.52744500
C	3.40432100	-5.22033200	6.71706700
C	5.27829100	-6.47381700	7.59227900
C	2.81714200	-5.23218200	7.97968800
H	2.90914900	-4.74935700	5.87370500
C	4.67648700	-6.51275300	8.84337300
H	6.24002900	-6.95378000	7.43696200
C	3.44717500	-5.88307900	9.03674200
H	1.85733100	-4.74796500	8.13305700
H	5.16232500	-7.03831000	9.66009300
H	2.97737700	-5.90516600	10.01597000
O	6.75184900	-5.04385100	5.16959400
C	7.86836300	-5.20200200	4.27237000
H	7.59506600	-5.80404700	3.40070500
H	8.15904400	-4.20281500	3.94200700
C	8.99503100	-5.88787800	5.04060400
H	8.59706900	-6.83616400	5.43031200
H	9.28061200	-5.27051500	5.90080000
C	10.13177900	-6.17010600	4.16822300
C	10.98966500	-6.47325400	3.37588700
C	11.93960700	-6.99990100	2.39536200
H	12.78071900	-7.46851300	2.92543600
H	12.36760200	-6.18581600	1.79601700
C	11.26989700	-8.04067400	1.48137600
H	10.51628800	-7.54118700	0.86138600
H	10.71674400	-8.75372400	2.10133700
C	12.27064100	-8.78760500	0.60342000
H	12.80987400	-8.07843000	-0.04011100
H	13.02760000	-9.26116700	1.24497500

C	11.59396000	-9.85618400	-0.25313900
H	12.31887700	-10.39616200	-0.87232400
H	11.07527000	-10.58374700	0.38191600
H	10.84600300	-9.40996400	-0.91808200
H	5.87779600	-7.18356400	4.74645300
O	4.51270700	-5.39104500	3.78538900
Cl	3.56581200	-15.57295800	3.33080500
Cl	7.58659500	-13.72259900	10.22825800
O	5.99167400	-14.95076200	5.38597900
O	5.37931500	-13.77211800	8.15297200
C	5.77141800	-16.34319500	5.26408800
H	5.89138400	-16.67883900	4.23159400
H	6.54074100	-16.80521700	5.88669200
H	4.78379500	-16.63376200	5.63401800
C	5.37039900	-15.16250700	8.44869000
H	6.29478200	-15.64524600	8.11858700
H	5.24509500	-15.31676000	9.52384300
H	4.52304300	-15.60211600	7.91445700

(S)-TS1

E = -5331.09787474 a.u.

0 1

Rh	6.41500900	-8.52396400	4.46382300
C	4.99878500	-13.48316200	5.00770300
C	4.36010600	-13.78239200	3.79987100
C	5.02001100	-12.14732300	5.46087500
C	3.79904200	-12.76991400	3.03322100
C	3.79937600	-11.46578600	3.49608200
C	4.38267400	-11.13800100	4.72215100

H	3.34077800	-13.00943000	2.07965900
H	3.32074600	-10.71189800	2.88640400
C	5.72695800	-11.87525700	6.74931600
C	5.14622300	-12.37167900	7.92897300
C	6.91578700	-11.13199100	6.80775500
C	5.74552700	-12.08627200	9.15830500
C	7.51578100	-10.90516700	8.05002300
C	6.93225700	-11.37023100	9.21829300
H	8.44308700	-10.34811500	8.11495500
H	7.38647700	-11.16778500	10.18216300
P	7.60578100	-10.32411000	5.29890300
P	4.44218700	-9.35792200	5.24315800
C	9.24310300	-9.73412600	5.88000800
C	10.39161600	-10.52663000	5.86396800
C	9.32277500	-8.43203100	6.38421200
C	11.59494300	-10.02830900	6.35813200
H	10.36443500	-11.52760800	5.44789200
C	10.52126900	-7.93843500	6.88339400
H	8.44577400	-7.79144900	6.36838900
C	11.66201300	-8.73726900	6.87279900
H	12.48381300	-10.65151900	6.33107400
H	10.55703000	-6.92265300	7.26623100
H	12.60312200	-8.35116000	7.25330800
C	7.92856500	-11.70534300	4.15104000
C	8.45368100	-12.92507700	4.58918900
C	7.66584400	-11.51251800	2.79177100
C	8.75351500	-13.92598700	3.67055700
H	8.62110400	-13.09633200	5.64987000
C	7.93842000	-12.53015800	1.88158700

H	7.26263400	-10.56558800	2.44289300
C	8.48993100	-13.73195500	2.31609800
H	9.18242000	-14.86326700	4.01372900
H	7.73276100	-12.37256800	0.82693400
H	8.71485300	-14.51818200	1.60148700
C	4.03011200	-9.38387100	7.01970600
C	2.97718200	-10.17431800	7.49435900
C	4.74295200	-8.58071000	7.90953600
C	2.63210900	-10.15183600	8.83902400
H	2.44555100	-10.83315500	6.81429000
C	4.38784600	-8.55395100	9.25619300
H	5.58340900	-7.99634600	7.54177900
C	3.33466000	-9.33466000	9.72104800
H	1.82925400	-10.78613500	9.20014800
H	4.93761800	-7.92869300	9.95164800
H	3.07248900	-9.32009600	10.77446200
C	2.92779400	-8.64626600	4.47648100
C	2.94607400	-8.39871100	3.10003600
C	1.76250600	-8.35985200	5.19374900
C	1.80849400	-7.94953700	2.43960300
H	3.86410100	-8.53739800	2.54455400
C	0.62868700	-7.89464400	4.53460800
H	1.73030400	-8.48347400	6.26912800
C	0.64113600	-7.70826300	3.15638700
H	1.84456700	-7.77215200	1.37019600
H	-0.26654600	-7.67309200	5.10783000
H	-0.24930500	-7.35435500	2.64513800
Cl	8.42053900	-8.05212900	3.19146800
C	4.99197100	-9.26786000	0.51524200

C	4.17521400	-8.30447800	-0.08631400
C	3.10245100	-8.74256700	-0.84488900
C	2.80133400	-10.08108500	-1.04801500
C	3.64946300	-10.99779000	-0.44162800
C	4.73822300	-10.63152000	0.32660000
H	4.34730200	-7.24670700	0.07743900
H	1.94806300	-10.39214500	-1.63614700
H	5.35646800	-11.37777100	0.81080000
O	6.01658200	-8.94401700	1.31706000
H	6.00325100	-7.99085200	1.57134500
H	6.20450200	-7.02215600	5.08459600
F	3.38076200	-12.30798400	-0.58410700
F	2.28566400	-7.81937700	-1.37960400
P	5.63549300	-6.16183300	3.81788400
C	4.13500400	-5.57458400	4.63726600
C	3.03345600	-5.16356200	3.89061100
C	4.09457800	-5.52509600	6.03257900
C	1.88987100	-4.70944800	4.54103700
H	3.07288200	-5.21792000	2.80759900
C	2.94689700	-5.08369000	6.67956700
H	4.96571500	-5.81999800	6.61295100
C	1.84383800	-4.67523400	5.93194300
H	1.02763700	-4.39733500	3.95968700
H	2.91381000	-5.05734900	7.76462100
H	0.94497700	-4.33174800	6.43595900
O	6.59311600	-4.90687700	4.15454700
C	8.00581400	-4.93455600	4.36182100
H	8.37612900	-5.95995900	4.39466100
H	8.47266700	-4.42796200	3.51248700

C	8.32539800	-4.19143400	5.65517300
H	7.86557500	-3.19666700	5.62081700
H	9.41198700	-4.04135100	5.69556600
C	7.89243800	-4.89271800	6.86775600
C	7.56825400	-5.44027700	7.89530000
C	7.23588000	-6.07953700	9.17245800
H	6.17989300	-6.37623800	9.17004200
H	7.33944400	-5.33843800	9.97629300
C	8.13270000	-7.28953500	9.47556900
H	9.17889800	-6.96194900	9.43270700
H	8.01442100	-8.03441000	8.68136500
C	7.86048500	-7.95121000	10.82595400
H	7.77031600	-7.18633700	11.60906100
H	6.89896300	-8.47854500	10.78935500
C	8.96715800	-8.93571900	11.20398400
H	8.72128100	-9.48842000	12.11675400
H	9.13919000	-9.66276600	10.40130400
H	9.91505300	-8.41229000	11.37147500
O	5.43218400	-6.44172800	2.35223000
Cl	4.98190800	-12.57364100	10.64859100
Cl	4.14742400	-15.42586300	3.24298200
O	3.96235600	-13.01875400	7.82439800
O	5.54777500	-14.39251800	5.84571500
C	3.87415200	-14.36660500	8.27978100
H	3.27015600	-14.41004200	9.18992700
H	3.39676400	-14.94235800	7.48397900
H	4.86490000	-14.78495400	8.47413700
C	6.33344600	-15.48056600	5.37857200
H	5.75230500	-16.40590400	5.40219200

H	6.70797600	-15.30411500	4.36983800
H	7.17949300	-15.56099500	6.06675800

(S)-TS1’

E = -4825.27606275 a.u.

0 1

Rh	6.51456200	-8.92615400	4.66467000
C	5.06192300	-13.90309800	5.01381100
C	3.96982200	-13.99558700	4.14461100
C	5.33628800	-12.65737300	5.62614400
C	3.13810200	-12.89655500	3.95718100
C	3.39337300	-11.69368500	4.58604900
C	4.51222400	-11.54798000	5.41191500
H	2.28568200	-12.99437100	3.29399500
H	2.71511400	-10.86831800	4.41722400
C	6.55802500	-12.58880000	6.48191700
C	6.50860200	-13.22078800	7.73337700
C	7.72259600	-11.92813700	6.06643700
C	7.62207600	-13.16574000	8.57316800
C	8.83595500	-11.92814600	6.91176800
C	8.78688000	-12.53638500	8.15693200
H	9.75357200	-11.43876200	6.60936600
H	9.64757400	-12.51199900	8.81636700
P	7.72843700	-10.93430800	4.50703500
P	4.96385400	-9.86358300	6.05043000
C	9.51982100	-10.63813500	4.25387100
C	10.31630100	-11.45387700	3.44824500
C	10.11504300	-9.58177200	4.95041400
C	11.68982500	-11.23453800	3.36896000

H	9.87203300	-12.25487700	2.86779700
C	11.48705300	-9.37285600	4.88357500
H	9.49269700	-8.91489800	5.53960500
C	12.27920800	-10.20567400	4.09675500
H	12.29733100	-11.86822300	2.72967000
H	11.92788600	-8.53755100	5.41743500
H	13.35051100	-10.03809900	4.03361700
C	7.22970800	-12.12886700	3.22092800
C	7.67070100	-13.45650400	3.23402400
C	6.35542900	-11.70147200	2.21857500
C	7.24062100	-14.34502000	2.25525700
H	8.32459600	-13.80375700	4.02873300
C	5.91945300	-12.59665200	1.24622400
H	6.02630700	-10.66613600	2.19980600
C	6.35975400	-13.91691400	1.26413800
H	7.58474500	-15.37516400	2.27208200
H	5.23389600	-12.25994500	0.47495600
H	6.01210600	-14.61570600	0.50930000
C	5.33986500	-10.12740800	7.81511000
C	4.50212200	-10.88550400	8.63991700
C	6.51570300	-9.58954700	8.34131500
C	4.82326400	-11.07405900	9.97870400
H	3.61766200	-11.36130600	8.22672200
C	6.83726000	-9.78066800	9.68119000
H	7.18934900	-9.04711100	7.68377100
C	5.98769000	-10.51673600	10.50201800
H	4.17662100	-11.67728700	10.60771500
H	7.75922100	-9.36775300	10.07902000
H	6.24402800	-10.67740800	11.54469000

C	3.35007600	-8.99439900	5.99483800
C	2.91987600	-8.52715600	4.74597500
C	2.53414600	-8.80025200	7.11138700
C	1.67646100	-7.92076000	4.61064000
H	3.56889300	-8.60717400	3.87905600
C	1.29274200	-8.18748800	6.97252000
H	2.86960200	-9.09846100	8.09774700
C	0.85516900	-7.76003600	5.72277900
H	1.36489600	-7.55662500	3.63694700
H	0.67274100	-8.03280200	7.85034300
H	-0.11444000	-7.28163100	5.61961900
Cl	7.76102200	-8.32051400	2.70275700
P	5.71084000	-6.51645300	4.87127600
C	4.79239600	-6.13434800	6.39290100
C	3.54415300	-5.52293900	6.28808000
C	5.31822300	-6.42599900	7.65086100
C	2.82521700	-5.21348900	7.43869600
H	3.14019600	-5.31191800	5.30314000
C	4.59255500	-6.13398700	8.80021700
H	6.29744100	-6.88873400	7.73690700
C	3.34352700	-5.52541600	8.69295100
H	1.84995900	-4.74300700	7.35464500
H	5.00132300	-6.38104800	9.77557600
H	2.77372300	-5.29534400	9.58885800
O	6.85392900	-5.37475900	5.05118100
C	8.00544700	-5.29472400	4.20331600
H	7.85781700	-5.87157900	3.28979900
H	8.13139800	-4.24011900	3.94650200
C	9.21897500	-5.81984700	4.97720600

H	8.91619500	-6.76158800	5.45286700
H	9.48013400	-5.12072100	5.78057600
C	10.34988100	-6.09280100	4.09445300
C	11.19989200	-6.43011900	3.30729100
C	12.12240400	-7.01701000	2.33512900
H	12.94181900	-7.51518100	2.87248100
H	12.58682000	-6.23896100	1.71547000
C	11.39576200	-8.04428500	1.44937500
H	10.67120600	-7.52020600	0.81468700
H	10.80512900	-8.70714700	2.09048900
C	12.35034900	-8.87151300	0.59223700
H	12.93013900	-8.21241800	-0.06893300
H	13.07859800	-9.37125200	1.24697600
C	11.61205900	-9.92081800	-0.23691000
H	12.30418800	-10.52142500	-0.83749500
H	11.04772500	-10.59704600	0.41579300
H	10.89481800	-9.44889100	-0.91782700
H	6.74778900	-7.69963500	5.66903200
O	4.86904500	-6.39974800	3.64409500
Cl	3.57627000	-15.41931600	3.20237200
Cl	7.55272900	-13.84828300	10.17654600
O	5.96921700	-14.85830100	5.31637100
O	5.33578200	-13.79172000	8.10430300
C	5.76292700	-16.24592600	5.13041600
H	5.90185000	-16.53520200	4.08640100
H	6.52740700	-16.72738300	5.74402900
H	4.77289200	-16.56058400	5.47282700
C	5.31704900	-15.19102700	8.35439000
H	6.23937500	-15.66883400	8.01179700

H	5.18715800	-15.37963700	9.42346600
H	4.46852500	-15.60704400	7.80339100

(S)-Int3

E = -5331.11949281 a.u.

0 1

Rh	6.35291300	-8.40627400	4.38640400
C	4.94552700	-13.36942200	5.01368200
C	4.27478300	-13.68734700	3.82818900
C	4.98481400	-12.02578200	5.44651900
C	3.70480900	-12.68488600	3.05720100
C	3.72840500	-11.37249900	3.49434700
C	4.34019800	-11.02303600	4.70063000
H	3.22571300	-12.93891200	2.11823400
H	3.23871700	-10.62931000	2.88154800
C	5.70445500	-11.76564800	6.73118100
C	5.11950300	-12.25880200	7.91074000
C	6.92409500	-11.07455900	6.78498100
C	5.74153700	-12.01158800	9.13685300
C	7.54610500	-10.88810300	8.02337400
C	6.95605500	-11.34332900	9.19233700
H	8.49774200	-10.37293800	8.08355600
H	7.42894900	-11.17344300	10.15338700
P	7.62199100	-10.28789900	5.27161800
P	4.35841900	-9.23109900	5.19777400
C	9.26902000	-9.71532500	5.84286900
C	10.40223300	-10.53103000	5.81059600
C	9.38351900	-8.42023400	6.35889100
C	11.62019300	-10.06466200	6.29890300

H	10.34889300	-11.52910200	5.38972200
C	10.59723800	-7.95780800	6.85215500
H	8.52869700	-7.75279000	6.36562100
C	11.72026900	-8.78015000	6.82469200
H	12.49417300	-10.70792600	6.25983000
H	10.65730200	-6.94665700	7.24379400
H	12.67240700	-8.41795700	7.20102900
C	7.95617300	-11.67428200	4.13319900
C	8.40709900	-12.91745500	4.58684300
C	7.79559000	-11.45566700	2.76173700
C	8.72563800	-13.91966300	3.67546300
H	8.50590600	-13.10289500	5.65369100
C	8.08800600	-12.47208400	1.85627900
H	7.46005000	-10.48740800	2.40112700
C	8.55943900	-13.70077600	2.30966100
H	9.09630000	-14.87628900	4.03267600
H	7.95701600	-12.29437000	0.79304500
H	8.79916700	-14.48755800	1.60062900
C	4.00244600	-9.26813000	6.98615300
C	2.94007400	-10.05673100	7.44542700
C	4.75031400	-8.52930400	7.90306900
C	2.61639500	-10.08886700	8.79506700
H	2.38347200	-10.67586200	6.74866400
C	4.42387800	-8.56616300	9.25589000
H	5.59710400	-7.93966400	7.56439700
C	3.35679900	-9.33802200	9.70301000
H	1.80388300	-10.72063200	9.13806500
H	5.00933600	-7.99625600	9.96929000
H	3.11587500	-9.37031500	10.76091700

C	2.81121800	-8.57921100	4.44855200
C	2.80426600	-8.35685700	3.06846300
C	1.64624400	-8.32035000	5.17411600
C	1.64515500	-7.97118500	2.40861600
H	3.72228000	-8.45775000	2.50831100
C	0.48886900	-7.91295000	4.51607400
H	1.63197600	-8.41046100	6.25297200
C	0.47646100	-7.76047900	3.13377500
H	1.66928400	-7.81855300	1.33412600
H	-0.40621400	-7.70804100	5.09546900
H	-0.43339900	-7.45332000	2.62685200
Cl	8.38982400	-7.95168600	3.16765700
C	5.00706200	-9.27507100	0.69653500
C	4.19661200	-8.39224300	-0.02654400
C	3.26391700	-8.92913000	-0.89754400
C	3.10092300	-10.29242300	-1.09854800
C	3.94004400	-11.12398100	-0.37097600
C	4.89383800	-10.65631900	0.51484100
H	4.26849200	-7.32102300	0.12734000
H	2.35673900	-10.68447400	-1.77926000
H	5.50882500	-11.34336400	1.08245400
O	5.88331500	-8.82984000	1.61633000
H	5.84035800	-7.83015200	1.69198200
H	6.53913900	-7.69332400	5.71063600
F	3.80122600	-12.45404900	-0.51342200
F	2.45197700	-8.08807100	-1.55672700
P	5.59226500	-6.29466600	3.72365500
C	4.10819100	-5.67868500	4.58989300
C	2.99538000	-5.24257700	3.87606600

C	4.09792500	-5.63232100	5.98529200
C	1.86969900	-4.78340000	4.55541900
H	3.01646100	-5.27577500	2.79140700
C	2.96967400	-5.18758500	6.66426900
H	4.98552900	-5.92645000	6.53929300
C	1.85153400	-4.76461800	5.94737800
H	1.00008800	-4.45200100	3.99534100
H	2.96531400	-5.16457700	7.75037400
H	0.96737700	-4.41706800	6.47457800
O	6.52990500	-5.08083300	4.28011600
C	7.93374800	-5.00083800	4.44644000
H	8.38311500	-5.99056100	4.51223900
H	8.36176500	-4.48625700	3.57973500
C	8.21292700	-4.20251200	5.71807900
H	7.66908400	-3.25175000	5.67310400
H	9.28354900	-3.96170400	5.74292900
C	7.85291700	-4.91776200	6.94623200
C	7.58254300	-5.48245300	7.98013400
C	7.31039900	-6.13465100	9.26434900
H	6.24866600	-6.40485300	9.32064300
H	7.47976400	-5.41122500	10.07311400
C	8.19204600	-7.37043200	9.49675600
H	9.24234100	-7.06528100	9.41088600
H	8.01977500	-8.09495100	8.69290200
C	7.96666200	-8.05403700	10.84505500
H	7.90816100	-7.30121000	11.64260000
H	7.00045000	-8.57501000	10.83557600
C	9.08230400	-9.04746600	11.16982300
H	8.86369500	-9.61825900	12.07845200

H	9.23124000	-9.75829000	10.34823700
H	10.03497300	-8.52737900	11.31941800
O	5.37451400	-6.29603900	2.21722700
Cl	4.97856900	-12.49312900	10.62933600
Cl	4.02914000	-15.33742000	3.30786300
O	3.91823800	-12.87327000	7.80883600
O	5.50808100	-14.26753700	5.85440000
C	3.79495800	-14.22149400	8.25522300
H	3.18147500	-14.25500800	9.15932700
H	3.31101200	-14.78193400	7.45221200
H	4.77354700	-14.66410200	8.45670300
C	6.25374000	-15.38544200	5.39225900
H	5.64872300	-16.29406800	5.44240800
H	6.61608400	-15.23648100	4.37451600
H	7.10843900	-15.47697600	6.06806600

(S)-Int3'

E = -4825.29007342 a.u.

0 1

Rh	6.51503600	-8.81059900	4.72443000
C	5.10534500	-13.79585200	4.97759000
C	4.05075100	-13.87236400	4.06131100
C	5.37065000	-12.55503800	5.60879400
C	3.25397300	-12.75667900	3.82869400
C	3.49597200	-11.55983800	4.47265500
C	4.56772900	-11.43372800	5.36249500
H	2.43323800	-12.83923400	3.12485900
H	2.83456800	-10.72775900	4.27559100
C	6.56542300	-12.52114100	6.50676000

C	6.47655400	-13.18147400	7.74124900
C	7.75805700	-11.88951400	6.12606700
C	7.57219600	-13.16229900	8.60671800
C	8.85011000	-11.91854400	6.99730200
C	8.75711600	-12.54289400	8.23248300
H	9.78439900	-11.44474900	6.71899200
H	9.60069600	-12.54589400	8.91418900
P	7.80756200	-10.90652000	4.56666400
P	4.94276200	-9.75957000	6.08103300
C	9.59621400	-10.58357300	4.34185100
C	10.42376700	-11.38722100	3.55307900
C	10.15560300	-9.49709100	5.02107300
C	11.78840600	-11.12332600	3.47191600
H	10.00854000	-12.21356500	2.98693700
C	11.51962900	-9.23884400	4.94746800
H	9.51499600	-8.84174300	5.60286500
C	12.34043300	-10.05740600	4.17640500
H	12.41829500	-11.74895500	2.84653700
H	11.93029500	-8.37754400	5.46343300
H	13.40470900	-9.85202900	4.10803800
C	7.36805800	-12.12171800	3.27523300
C	7.82925800	-13.44226500	3.32142500
C	6.52976800	-11.72185500	2.23145000
C	7.45947700	-14.34772000	2.33328800
H	8.45764900	-13.76858100	4.14517800
C	6.15028700	-12.63522400	1.25203000
H	6.18666800	-10.69299700	2.18034400
C	6.61329200	-13.94661000	1.30134200
H	7.82696900	-15.36927900	2.37222500

H	5.49242200	-12.31815300	0.44898700
H	6.31338100	-14.65808600	0.53794400
C	5.27136600	-10.10875100	7.84140500
C	4.36966600	-10.89371800	8.56983700
C	6.42607600	-9.64179700	8.47021400
C	4.60274000	-11.17049900	9.91117200
H	3.49907300	-11.31904800	8.07994600
C	6.66250200	-9.92529400	9.81068800
H	7.15925000	-9.07845500	7.90386100
C	5.74630000	-10.68091300	10.53583900
H	3.90278500	-11.79140400	10.46060400
H	7.57049900	-9.56478500	10.28384500
H	5.93531700	-10.90974300	11.57996100
C	3.31182400	-8.92660100	6.01748900
C	2.87940100	-8.44738300	4.77357400
C	2.49484400	-8.74900000	7.13579300
C	1.63190300	-7.84976700	4.64425100
H	3.53059500	-8.49462800	3.90610700
C	1.24792500	-8.14661400	7.00174700
H	2.83530100	-9.04185700	8.12144500
C	0.80753600	-7.70960100	5.75690500
H	1.32243300	-7.47148600	3.67554300
H	0.62845500	-8.00341200	7.88174000
H	-0.16495200	-7.23640100	5.65825400
Cl	7.59848800	-8.29634500	2.65620600
P	5.67718000	-6.59443300	4.84085500
C	4.84358800	-6.19833400	6.42448100
C	3.60410600	-5.56194200	6.38633000
C	5.42050500	-6.50286300	7.65603000

C	2.93966500	-5.25666700	7.57081900
H	3.16640000	-5.32644300	5.42130200
C	4.75104800	-6.21678300	8.84111700
H	6.40248600	-6.96518900	7.68793500
C	3.50479300	-5.59446600	8.79811600
H	1.97015800	-4.76769900	7.53482600
H	5.20205400	-6.47618800	9.79489100
H	2.97782500	-5.36887800	9.72117000
O	6.90004800	-5.53010400	5.10258000
C	7.98073400	-5.33628400	4.19693700
H	7.82458000	-5.88672100	3.26926700
H	8.02773200	-4.26751900	3.96883700
C	9.26822900	-5.80603200	4.88645000
H	9.01567000	-6.73311100	5.41674000
H	9.57604500	-5.07810100	5.64652700
C	10.33915100	-6.09565800	3.93693600
C	11.14372400	-6.46045600	3.11478400
C	12.00880900	-7.08839700	2.11602600
H	12.87760400	-7.53455400	2.62067000
H	12.40667900	-6.34298300	1.41530200
C	11.24424600	-8.18460200	1.35359000
H	10.46174100	-7.71683500	0.74432200
H	10.71968500	-8.81588500	2.07868600
C	12.15079500	-9.04875500	0.48092900
H	12.66562900	-8.42349500	-0.26171300
H	12.93628200	-9.49281600	1.10919700
C	11.37631700	-10.16089000	-0.22426600
H	12.03494200	-10.78682800	-0.83651000
H	10.87554900	-10.80423500	0.50880200

H	10.60116200	-9.74486100	-0.87760100
H	7.20045800	-8.42140900	6.02203600
O	4.79099500	-6.27146500	3.67573100
Cl	3.65138300	-15.29543300	3.12091700
Cl	7.45712900	-13.88333100	10.19080600
O	5.98132300	-14.76167400	5.32869000
O	5.28974000	-13.74935900	8.06844700
C	5.83783300	-16.13817200	5.03473100
H	6.01921000	-16.34375300	3.97765700
H	6.60534500	-16.62952400	5.63633800
H	4.85366200	-16.51671500	5.32446500
C	5.25114500	-15.15422400	8.28562300
H	6.18413600	-15.63166600	7.97317800
H	5.07372700	-15.36326100	9.34393600
H	4.42507900	-15.55225600	7.68908300

(S)-Int4

E = -4825.30334699 a.u.

0 1

Rh	5.13790200	-8.68915100	4.80534800
C	5.85185400	-14.05322500	5.70573100
C	5.02663600	-14.77709800	4.84653500
C	5.56671700	-12.70736500	5.98336100
C	3.89135100	-14.19314800	4.30704100
C	3.57390400	-12.88393100	4.63592900
C	4.39597200	-12.11746200	5.46682100
H	3.24721700	-14.76719400	3.65025500
H	2.66159000	-12.46232000	4.23481200
C	6.52335100	-11.98180200	6.87039800

C	6.57613000	-12.38850100	8.21987600
C	7.32883900	-10.92688100	6.40689300
C	7.38051500	-11.67963700	9.11500600
C	8.12905000	-10.25143500	7.33637700
C	8.15172900	-10.61496400	8.67255400
H	8.74098600	-9.41246100	7.03619100
H	8.76151100	-10.06485500	9.38089700
P	7.12024100	-10.27342000	4.68642200
P	3.88573300	-10.37135600	5.87281400
C	8.68486200	-9.44516400	4.18074500
C	8.57558000	-8.72843200	2.98061400
C	9.94226100	-9.56685800	4.77827600
C	9.69122900	-8.12643500	2.41109300
H	7.61023300	-8.66092100	2.48506200
C	11.05621700	-8.94427000	4.21684000
H	10.07575800	-10.17000300	5.66960100
C	10.93325900	-8.21900500	3.03619900
H	9.58791500	-7.58043600	1.47844100
H	12.02410900	-9.04250200	4.69952300
H	11.80324700	-7.73957300	2.59750200
C	7.26420800	-11.72113200	3.55167600
C	8.49171900	-12.37740700	3.40090200
C	6.18227100	-12.11931300	2.76683500
C	8.62163600	-13.43799800	2.51287100
H	9.35787500	-12.05607200	3.97177600
C	6.31130300	-13.18987600	1.88539700
H	5.24840500	-11.57549000	2.82383600
C	7.52499800	-13.85569200	1.76054300
H	9.58008600	-13.93839100	2.40806500

H	5.45632700	-13.49248200	1.28880100
H	7.62360500	-14.68777000	1.06967700
C	3.97923700	-10.39149000	7.70844200
C	3.47962800	-11.50971900	8.39025100
C	4.50433200	-9.33321300	8.45132800
C	3.50553300	-11.56402600	9.77756300
H	3.09078700	-12.35659300	7.83330900
C	4.53552600	-9.39098600	9.84117800
H	4.88677700	-8.44973800	7.95473900
C	4.03717100	-10.50452000	10.50754400
H	3.13203100	-12.44734600	10.28548200
H	4.94615200	-8.55363900	10.39632100
H	4.07106200	-10.55284500	11.59173300
C	2.09228600	-10.39352000	5.51060200
C	1.69358600	-10.30315600	4.17174200
C	1.12147200	-10.47614300	6.51035600
C	0.34686200	-10.35970800	3.83974500
H	2.43786400	-10.15504700	3.39429300
C	-0.22891000	-10.50559300	6.17246100
H	1.40341300	-10.50253400	7.55646100
C	-0.61778800	-10.46065600	4.83821600
H	0.05172100	-10.28772000	2.79767400
H	-0.97586100	-10.56353900	6.95870100
H	-1.67195600	-10.48659400	4.57758800
Cl	4.52828400	-9.22404000	2.39492000
P	3.36086600	-7.14064700	5.08308600
C	3.41237500	-6.47310500	6.77654200
C	4.44398100	-5.64645600	7.22771600
C	2.42313400	-6.89398600	7.66520800

C	4.50040400	-5.26729600	8.56468400
H	5.18928000	-5.29213700	6.52210100
C	2.47876000	-6.51099100	9.00364500
H	1.61864900	-7.52468200	7.29723500
C	3.52085500	-5.70732000	9.45629200
H	5.30068100	-4.61954100	8.91236800
H	1.71160300	-6.84774900	9.69468900
H	3.56668000	-5.41280000	10.50109000
O	3.76679800	-5.75988000	4.27461900
C	4.01912900	-5.90163100	2.88469900
H	3.44930500	-6.73808000	2.47212500
H	3.69290400	-4.97676700	2.40423100
C	5.52752800	-6.12777700	2.63718400
H	6.04323100	-5.17038600	2.49638900
H	5.67006800	-6.74044200	1.74274700
C	6.13567500	-6.76375600	3.80831400
C	6.73299200	-6.95896700	4.86242800
C	7.68871000	-6.84198300	5.97336600
H	7.97830100	-7.83869200	6.30346100
H	7.18675800	-6.37647900	6.83099000
C	8.94352300	-6.06139500	5.56723800
H	8.67968400	-5.01517100	5.36657500
H	9.32923300	-6.47803900	4.63065600
C	10.03260300	-6.13658500	6.63595800
H	9.66429100	-5.70403800	7.57591500
H	10.25228400	-7.19370200	6.84187000
C	11.31710100	-5.43300900	6.20405200
H	12.08991300	-5.49975500	6.97675100
H	11.71274100	-5.88677900	5.28787100

H	11.13621000	-4.37147000	6.00009200
H	5.52498600	-8.37964300	6.26064600
O	1.98019200	-7.59870500	4.74394800
Cl	5.40784100	-16.43432600	4.47847700
Cl	7.38294100	-12.06377000	10.81500200
O	6.87245800	-14.68492000	6.34862000
O	5.76103300	-13.39362700	8.60317700
C	8.14428600	-14.67912900	5.72038500
H	8.07687100	-15.02267600	4.68378100
H	8.58228800	-13.67552900	5.74184700
H	8.76622100	-15.36486000	6.29875900
C	6.32691900	-14.55780000	9.20008600
H	7.40929200	-14.59364700	9.05136300
H	6.09983500	-14.57285000	10.26912600
H	5.87535000	-15.41860500	8.70436400

(S)-TS3

E = -4825.29488015 a.u.

0 1

Rh	5.15598200	-8.66340800	4.66219600
C	5.93999100	-14.02160100	5.78101600
C	5.06387400	-14.81543100	5.04154500
C	5.64132100	-12.66984700	6.00433200
C	3.86863700	-14.29228000	4.57317600
C	3.54561200	-12.97139600	4.84811700
C	4.41678000	-12.13775700	5.55445600
H	3.18652500	-14.92107600	4.01163300
H	2.59430600	-12.58928300	4.49880900
C	6.64240400	-11.86822200	6.76994400

C	6.79380300	-12.16887000	8.13946000
C	7.39583600	-10.84040800	6.18050800
C	7.63235400	-11.37436100	8.92482000
C	8.23647700	-10.07558600	6.99916700
C	8.34826900	-10.32983900	8.35576400
H	8.80764900	-9.25582800	6.58193900
H	8.98754000	-9.71520600	8.98071200
P	7.09030500	-10.27692200	4.44767600
P	3.92669500	-10.36700500	5.86075400
C	8.65014600	-9.48452100	3.89277600
C	8.51364800	-8.59751000	2.81947900
C	9.93031700	-9.75103700	4.38940300
C	9.62470700	-7.95185800	2.28862600
H	7.52765200	-8.43016100	2.39433100
C	11.04234600	-9.09877100	3.86210100
H	10.06978700	-10.46640500	5.19409800
C	10.89002200	-8.18740800	2.82062600
H	9.50032700	-7.26229400	1.45915000
H	12.02919000	-9.30894000	4.26408700
H	11.75693900	-7.67427500	2.41538600
C	7.09972700	-11.75514300	3.35303900
C	8.28504500	-12.45084600	3.08927500
C	5.92440000	-12.15312700	2.71398000
C	8.28386600	-13.55253300	2.24191100
H	9.21983100	-12.13157000	3.53947200
C	5.92250000	-13.26325800	1.87427000
H	5.01716800	-11.57967900	2.85265000
C	7.09705300	-13.97033700	1.64292700
H	9.21112700	-14.08427600	2.04858500

H	4.99719700	-13.56545800	1.39388900
H	7.09383600	-14.83487600	0.98582900
C	4.08476500	-10.27973700	7.69101600
C	3.67797200	-11.36655100	8.47523900
C	4.59929900	-9.15014300	8.32880300
C	3.78949200	-11.32259900	9.85957500
H	3.30178000	-12.26782200	8.00105300
C	4.71698300	-9.10752600	9.71380600
H	4.89426100	-8.28364100	7.74945300
C	4.31447700	-10.19413300	10.48266600
H	3.48963400	-12.18372800	10.44818100
H	5.11676800	-8.21547300	10.18556200
H	4.41646000	-10.16647300	11.56331600
C	2.12462000	-10.41083000	5.55237500
C	1.70160600	-10.29398300	4.22367000
C	1.17403000	-10.55748000	6.56668200
C	0.34898000	-10.37332000	3.91353500
H	2.42745000	-10.11089300	3.43650400
C	-0.18041200	-10.61120000	6.25203800
H	1.47995900	-10.61262300	7.60550300
C	-0.59339600	-10.53031600	4.92483000
H	0.03356600	-10.27395300	2.87998000
H	-0.91243400	-10.71680300	7.04739000
H	-1.65119300	-10.57345000	4.68172600
Cl	4.51869600	-9.11147200	2.30862100
P	3.37747800	-7.10607200	4.81259300
C	3.18777500	-6.55897100	6.54011800
C	4.09250100	-5.69720100	7.16481200
C	2.13434000	-7.10504200	7.27570500

C	3.96370400	-5.41060600	8.52034600
H	4.88177900	-5.23894900	6.57768100
C	2.00591200	-6.81650000	8.63178800
H	1.42032400	-7.75254400	6.77537800
C	2.92472300	-5.97879100	9.25747500
H	4.66554600	-4.73438500	9.00119900
H	1.18957400	-7.25076600	9.20137800
H	2.82707600	-5.75885900	10.31687700
O	3.93642800	-5.68206900	4.18512800
C	4.51574600	-5.72486300	2.88892600
H	4.02706800	-6.48605300	2.27566600
H	4.35584900	-4.74326700	2.43736300
C	6.02987600	-6.02650000	2.98523500
H	6.60521400	-5.10422800	3.12765500
H	6.35953400	-6.50468200	2.05765300
C	6.24829000	-6.90408800	4.14911000
C	6.61146000	-7.07198800	5.34258500
C	7.48798500	-6.73376000	6.49654100
H	7.49020000	-7.54916400	7.22547200
H	7.06992800	-5.86231800	7.01687100
C	8.91816500	-6.44024100	6.02445100
H	8.91433700	-5.53655200	5.40176400
H	9.25695400	-7.25443900	5.37420100
C	9.89882800	-6.27445400	7.18356000
H	9.57584300	-5.44927700	7.83197400
H	9.87577600	-7.18087000	7.80566200
C	11.32530500	-6.02803300	6.69573900
H	12.02582600	-5.93977800	7.53231100
H	11.66094200	-6.85098000	6.05388300

H	11.38422600	-5.10483300	6.10815800
H	5.72249000	-8.21180800	6.07547600
O	2.06254400	-7.52160200	4.24245600
Cl	5.46154900	-16.48186300	4.73524100
Cl	7.75236500	-11.63285700	10.64415400
O	7.03416000	-14.58724300	6.36341300
O	6.04104700	-13.16657000	8.65039500
C	8.24461800	-14.56196500	5.62433200
H	8.10442200	-14.97178100	4.61936000
H	8.63160500	-13.53980700	5.54784300
H	8.94927400	-15.18120100	6.18243500
C	6.69409000	-14.27664000	9.26174300
H	7.75790400	-14.29726700	9.01087600
H	6.57060300	-14.22796000	10.34674000
H	6.22256500	-15.17792800	8.86659400

(S)-TS3'

E = -4825.28128662 a.u.

0 1

Rh	5.01222400	-8.99589200	4.83717200
C	6.51476200	-14.02116900	5.03649900
C	6.07357700	-14.54827500	3.82337600
C	5.82956000	-12.92728500	5.60318200
C	4.96741900	-14.00772100	3.18314400
C	4.27714500	-12.95712300	3.76279400
C	4.67344700	-12.41497900	4.99266600
H	4.65905500	-14.40051300	2.22063600
H	3.43917600	-12.53247300	3.22655900
C	6.47067100	-12.31747200	6.80393600

C	6.48115800	-13.05126500	7.99692000
C	7.13806800	-11.08095800	6.73677600
C	7.11111300	-12.53105600	9.12759500
C	7.79249500	-10.60361400	7.87523800
C	7.77265800	-11.31381600	9.06763700
H	8.33263800	-9.66599600	7.84581000
H	8.27752700	-10.92926800	9.94709000
P	7.09371000	-10.07475000	5.18170200
P	3.75997100	-10.91186200	5.59348400
C	8.39185800	-8.80270000	5.48240100
C	9.61100200	-8.81871800	4.80169400
C	8.13525400	-7.75255200	6.37587300
C	10.55416100	-7.81403200	5.01321000
H	9.82746300	-9.60334200	4.08523500
C	9.08514000	-6.76465100	6.60249300
H	7.17864900	-7.68725600	6.88651600
C	10.29840600	-6.78935000	5.91700300
H	11.48980200	-7.83746800	4.46261200
H	8.85979100	-5.96566600	7.30176100
H	11.03503300	-6.00858300	6.08110100
C	7.84745900	-11.21265900	3.96849200
C	8.96098000	-11.98317900	4.32891300
C	7.31486900	-11.32057300	2.68323800
C	9.54324300	-12.83972900	3.40480400
H	9.35807000	-11.93075200	5.33854300
C	7.90547500	-12.18206900	1.76117500
H	6.42735100	-10.75426300	2.41379000
C	9.01726400	-12.93726000	2.11731100
H	10.39506200	-13.44626100	3.69548800

H	7.48014100	-12.26814100	0.76612600
H	9.46617800	-13.61736600	1.39965400
C	3.45930000	-11.10137100	7.40718600
C	2.34012400	-11.75519900	7.93095500
C	4.34877400	-10.50389500	8.30226100
C	2.14643100	-11.84712600	9.30674000
H	1.59177900	-12.17978100	7.27391900
C	4.16970200	-10.60826900	9.67647600
H	5.18806300	-9.93338800	7.92553200
C	3.06706400	-11.28678400	10.18675700
H	1.26509100	-12.35416000	9.68825800
H	4.89139800	-10.14714600	10.34466700
H	2.91834700	-11.36409200	11.25964300
C	2.09412100	-11.17505400	4.87401000
C	1.42344700	-10.10409200	4.28811900
C	1.46486400	-12.42692500	4.90921800
C	0.14195500	-10.26645700	3.77215900
H	1.92051400	-9.14739200	4.22016900
C	0.18476400	-12.59217500	4.39271700
H	1.98394800	-13.28739200	5.32267900
C	-0.48228300	-11.50776600	3.82738300
H	-0.36424500	-9.42022800	3.31764400
H	-0.28764400	-13.56939000	4.42632100
H	-1.48141600	-11.63525600	3.42115500
Cl	4.27911700	-9.72251500	2.49698700
P	3.44073300	-7.37423300	6.00610200
C	4.53424600	-6.52084100	7.18496500
C	5.32201300	-5.42395700	6.83197000
C	4.60098000	-7.03773700	8.48087600

C	6.19163800	-4.86565700	7.76434100
H	5.25805700	-5.01131800	5.83134800
C	5.47822800	-6.48221800	9.40764500
H	3.96156000	-7.87244000	8.75069500
C	6.27961400	-5.40043100	9.04853300
H	6.80127500	-4.01028400	7.48728600
H	5.52855800	-6.88833200	10.41380100
H	6.96259000	-4.96533000	9.77269100
O	2.63207200	-6.06311700	5.42567100
C	2.19643300	-6.17259000	4.08251500
H	1.52864600	-7.03491000	3.96511000
H	1.62929400	-5.26910500	3.85325400
C	3.44769600	-6.31303900	3.21315800
H	3.82596400	-5.32498500	2.92230500
H	3.25413600	-6.89674200	2.30742200
C	4.52126400	-6.98105900	3.99560100
C	5.76645800	-7.26397500	3.94798300
C	7.00560700	-6.78392600	3.28653100
H	7.79091800	-6.62410700	4.03403400
H	6.79753500	-5.80612100	2.82976400
C	7.50752600	-7.76297900	2.22162800
H	6.72692300	-7.91362900	1.46677600
H	7.66115700	-8.74192800	2.68371600
C	8.81401300	-7.31829100	1.56959300
H	8.65992200	-6.37605800	1.02647900
H	9.55302300	-7.10770600	2.35457300
C	9.36386700	-8.38284000	0.62116200
H	10.30621200	-8.06577600	0.16171800
H	9.54418200	-9.32351800	1.15559900

H	8.65084700	-8.59668500	-0.18328800
H	5.47983000	-8.59130900	6.25446200
O	2.48315900	-8.31472100	6.66608500
Cl	6.95179400	-15.83954500	3.04792800
Cl	7.07590500	-13.42527600	10.61894100
O	7.64633400	-14.44691900	5.64877200
O	5.93298500	-14.29385200	8.05053400
C	7.67829800	-15.77829500	6.15724300
H	8.30911400	-16.40458000	5.52068100
H	8.09448500	-15.72615200	7.16538600
H	6.67271800	-16.20295300	6.21464300
C	4.53125000	-14.34073400	8.29203800
H	4.29526800	-15.38706600	8.49208400
H	4.26088300	-13.72625900	9.15671400
H	3.97428800	-13.99666700	7.41451200

(S)-Int5

E = -4825.33078428 a.u.

0 1

Rh	5.75382200	-8.19083500	4.70029400
C	4.92595700	-13.30308700	5.28330400
C	3.73732200	-13.37737400	4.55790500
C	5.31203000	-12.09466300	5.89257200
C	2.91357200	-12.26875100	4.44145200
C	3.26817300	-11.09639800	5.08480800
C	4.44340700	-10.99395100	5.83766200
H	2.00623900	-12.31960900	3.85085700
H	2.61075300	-10.24401800	4.98212600
C	6.67764400	-12.09319400	6.49806700

C	6.87089600	-12.87142100	7.64865600
C	7.76210200	-11.43827600	5.88815000
C	8.15909100	-13.01209200	8.17063300
C	9.04575100	-11.66824400	6.39107100
C	9.24658100	-12.44765500	7.52117100
H	9.90735000	-11.21582400	5.91620900
H	10.24433300	-12.59075300	7.92171000
P	7.46690000	-10.23921300	4.49986500
P	4.80009300	-9.32740000	6.53139400
C	9.12107500	-9.48196200	4.24793700
C	9.71029300	-9.42658700	2.98274700
C	9.74690100	-8.81519400	5.30831700
C	10.90867700	-8.74547300	2.79077100
H	9.22272700	-9.89306000	2.13470200
C	10.94411500	-8.13583100	5.11624900
H	9.31267200	-8.84272600	6.30226600
C	11.53044400	-8.10028400	3.85399300
H	11.34772800	-8.70952900	1.79850000
H	11.41604000	-7.63090400	5.95397600
H	12.46289900	-7.56558200	3.69982900
C	7.22530400	-11.35635900	3.07435800
C	8.22577500	-12.25958700	2.69109200
C	6.00974900	-11.33508500	2.39124500
C	8.01462100	-13.12603000	1.62649400
H	9.17243400	-12.28201500	3.22528700
C	5.79505000	-12.22388700	1.33784500
H	5.23240300	-10.61980800	2.65180500
C	6.79231800	-13.11268700	0.95197100
H	8.79764100	-13.81654200	1.32687800

H	4.84488700	-12.20118200	0.81375100
H	6.62387500	-13.79457600	0.12337100
C	5.94966700	-9.54040700	7.94428800
C	5.58044500	-10.32725300	9.04013200
C	7.18018200	-8.88613400	7.95724800
C	6.45093300	-10.48758200	10.10988000
H	4.62403400	-10.84053100	9.04378400
C	8.04987500	-9.03881600	9.03391900
H	7.46136300	-8.25340400	7.12034400
C	7.68894500	-9.84681100	10.10681200
H	6.16914100	-11.12602900	10.94088000
H	9.00865500	-8.52867900	9.02920500
H	8.36991700	-9.97930500	10.94198100
C	3.30624200	-8.88244800	7.52111800
C	2.15650700	-9.66318600	7.64360700
C	3.40473800	-7.71239800	8.28524700
C	1.11867800	-9.26213000	8.48354900
H	2.05697100	-10.59518100	7.10141400
C	2.37484800	-7.31683700	9.12580200
H	4.29516000	-7.10036700	8.22467100
C	1.22019200	-8.09030600	9.22304700
H	0.22922700	-9.88014900	8.55927500
H	2.47786300	-6.40204600	9.70271400
H	0.40930600	-7.78498300	9.87764200
Cl	6.98315500	-7.55027400	2.75891000
P	3.84511500	-8.03226700	3.50038700
H	5.95620600	-6.67123400	7.16489100
O	3.67665300	-9.24617200	2.64467100
C	2.29364700	-7.75522800	4.44058400

C	1.16638200	-8.45649000	3.99580000
C	2.15710400	-6.83768300	5.48336600
C	-0.07245200	-8.24801400	4.59302900
H	1.27793100	-9.16285800	3.17837200
C	0.91280600	-6.61527700	6.06571200
H	3.03098200	-6.31645300	5.86189600
C	-0.20133200	-7.32302600	5.62643100
H	-0.93958000	-8.80220200	4.24546900
H	0.82230000	-5.90591000	6.88228500
H	-1.16870100	-7.15755200	6.09192900
O	3.91473000	-6.69031800	2.59612600
C	4.00688000	-5.40829100	3.19929300
H	3.08793700	-5.20772800	3.76476600
H	4.04819900	-4.70256600	2.36687400
C	5.24170400	-5.21714400	4.08100100
H	6.12566800	-5.22886700	3.43793100
H	5.15172000	-4.20941100	4.50263200
C	5.45693400	-6.23284500	5.17804200
C	5.75948900	-5.88514200	6.43819800
C	5.86821200	-4.50748100	7.04575600
H	6.88740300	-4.36024700	7.43158300
H	5.70709800	-3.71978700	6.30336900
C	4.87321100	-4.32579000	8.20052500
H	3.85547300	-4.51633500	7.82986100
H	5.06797100	-5.08732600	8.97060100
C	4.92986600	-2.94236600	8.84696500
H	4.73208800	-2.17787700	8.08395200
H	5.94794100	-2.75530300	9.21372300
C	3.93338500	-2.79119100	9.99534300

H	3.98623900	-1.79436400	10.44521000
H	4.13068500	-3.52602500	10.78510400
H	2.90586100	-2.94651000	9.64509100
Cl	8.40960200	-13.84614000	9.68117400
Cl	3.28951400	-14.87304400	3.79068700
O	5.77247500	-13.37011200	8.26059200
O	5.67884200	-14.42496600	5.44520900
C	5.64898000	-14.78456900	8.40763700
H	5.66974300	-15.03916600	9.47044900
H	4.69266200	-15.07624200	7.96758900
H	6.44989300	-15.31027200	7.88361200
C	6.67868000	-14.65578200	4.45757100
H	6.96660200	-15.70388400	4.55531600
H	6.28958300	-14.47096800	3.45155400
H	7.54701800	-14.01356000	4.63408500

(S)-Int5’

E = -4825.34278646 a.u.

0 1

Rh	5.03020400	-9.10229300	4.76743800
C	6.70787100	-14.05730500	5.15655200
C	6.26350700	-14.70184800	4.00195900
C	5.97762800	-12.96114300	5.65692400
C	5.12185900	-14.26279000	3.34619800
C	4.39342400	-13.20236900	3.85822300
C	4.78275600	-12.55995300	5.04041800
H	4.81702100	-14.74170800	2.42226200
H	3.52929000	-12.85254600	3.30817500
C	6.59902600	-12.22975500	6.79978900

C	6.66086700	-12.88530600	8.03713000
C	7.18528400	-10.95813900	6.65221900
C	7.26349400	-12.26052600	9.12858800
C	7.81512400	-10.37589800	7.75532200
C	7.84828700	-11.01126400	8.98858700
H	8.29580300	-9.41038300	7.66601100
H	8.33394800	-10.54281000	9.83756700
P	7.07267400	-10.02214300	5.05142200
P	3.83031500	-11.05977500	5.56265800
C	8.36408500	-8.72207300	5.22109700
C	9.54339000	-8.74570000	4.47467400
C	8.11749100	-7.62478400	6.05860600
C	10.45937700	-7.69919900	4.56719900
H	9.74477900	-9.56412100	3.79274300
C	9.04120400	-6.59372300	6.16590700
H	7.18142700	-7.55955900	6.60518000
C	10.21476300	-6.62465800	5.41447200
H	11.36286200	-7.72689100	3.96575100
H	8.83135900	-5.75408300	6.82188900
H	10.92881900	-5.80942500	5.48282400
C	7.76480100	-11.22919200	3.86742800
C	8.94561900	-11.90971400	4.19262000
C	7.12851600	-11.48313900	2.65191600
C	9.49870300	-12.81276200	3.29492000
H	9.42209300	-11.74982900	5.15580900
C	7.68836200	-12.39246100	1.75803800
H	6.19554200	-10.97813500	2.41232100
C	8.87244200	-13.05037300	2.07243800
H	10.40679700	-13.34571100	3.55783100

H	7.18645100	-12.58789200	0.81555800
H	9.30009600	-13.76451300	1.37524400
C	3.51636200	-11.16657200	7.38698300
C	2.43225600	-11.86414100	7.93641700
C	4.35838900	-10.47279900	8.25990500
C	2.23110600	-11.90397500	9.31267700
H	1.72381200	-12.37197800	7.29368500
C	4.17536400	-10.53166800	9.63798200
H	5.16518800	-9.86962400	7.86320600
C	3.11201400	-11.25122100	10.17186800
H	1.38057700	-12.44829600	9.71247900
H	4.86258400	-9.99779500	10.28720800
H	2.96192800	-11.29338400	11.24672200
C	2.15699100	-11.38702800	4.88258000
C	1.41214400	-10.33305600	4.35009400
C	1.58234700	-12.66601800	4.93905800
C	0.11387000	-10.55615700	3.89528400
H	1.84775100	-9.34421800	4.28727200
C	0.28871600	-12.88435300	4.48122200
H	2.15313900	-13.50512800	5.32778100
C	-0.45032800	-11.82473100	3.95868800
H	-0.45099000	-9.72790200	3.47859900
H	-0.13865000	-13.88170900	4.52820400
H	-1.46047900	-11.99298900	3.59638700
Cl	4.14904700	-9.69205200	2.49013800
P	3.69948100	-6.61227100	5.56621000
C	4.14108800	-6.47546000	7.31220300
C	5.17683900	-5.62248300	7.70532600
C	3.47124600	-7.24294800	8.26462000

C	5.53515000	-5.53405700	9.04588900
H	5.71031800	-5.03851900	6.95939600
C	3.82027100	-7.13870400	9.60834500
H	2.69551500	-7.93323400	7.94779100
C	4.85147700	-6.28884200	9.99857000
H	6.34351300	-4.87547000	9.34976200
H	3.29408800	-7.73616500	10.34626000
H	5.12709700	-6.21532100	11.04665500
O	2.82389600	-5.27339900	5.28107100
C	3.29686000	-4.59465200	4.09445400
H	2.77325200	-5.00906600	3.22650900
H	3.02624700	-3.54408700	4.21317400
C	4.81472000	-4.82541400	3.97058200
H	5.35805100	-4.10257500	4.59509900
H	5.13534500	-4.67405100	2.93577300
C	5.00979100	-6.25068100	4.44822100
C	5.73572600	-7.29524100	3.99461800
C	6.81027000	-7.01685000	2.96559300
H	7.71112800	-6.71472200	3.51976200
H	6.52150800	-6.12713800	2.38792700
C	7.18968700	-8.11944400	1.98736200
H	6.32417400	-8.38887600	1.37404700
H	7.44870800	-9.02344100	2.53955700
C	8.38182800	-7.74278400	1.11037500
H	8.12770500	-6.87522800	0.48569300
H	9.21675000	-7.42622000	1.75233500
C	8.83125100	-8.90527000	0.22600600
H	9.68399300	-8.62922400	-0.40409000
H	9.12389600	-9.76831300	0.83653300

H	8.01711600	-9.23317600	-0.43055500
H	5.44847000	-8.65250400	6.19743700
O	3.05424400	-7.95503000	5.28347600
Cl	7.18383000	-16.01017000	3.30843100
Cl	7.29219900	-13.06315000	10.67155800
O	7.87691500	-14.37010200	5.76478000
O	6.18923900	-14.15344100	8.17122200
C	8.00644700	-15.65645300	6.36566800
H	8.67494200	-16.27982000	5.76589800
H	8.42584500	-15.50418300	7.36230100
H	7.03428400	-16.14594900	6.46524200
C	4.79750100	-14.27001000	8.44361200
H	4.62770300	-15.31686000	8.70003100
H	4.50651000	-13.63018900	9.28293700
H	4.20512500	-14.00422600	7.56217100

(S)-TS4

E = -4825.30621905 a.u.

0 1

Rh	5.71299800	-8.29826200	4.65635000
C	5.23607500	-13.48915700	5.41848700
C	4.15024100	-13.68586100	4.56831000
C	5.48575200	-12.21051800	5.95011800
C	3.32175300	-12.62526700	4.22843100
C	3.52848200	-11.38543400	4.80812100
C	4.56606900	-11.17681300	5.72633700
H	2.52564600	-12.76843900	3.50672500
H	2.94000800	-10.54897800	4.45280100
C	6.81673200	-11.98872800	6.58058000

C	7.11431600	-12.65792000	7.77716500
C	7.76964400	-11.15492700	5.96160500
C	8.38424800	-12.50803300	8.34243500
C	9.04571900	-11.08136900	6.52691700
C	9.35340100	-11.75259400	7.70191000
H	9.81610900	-10.47589100	6.06803100
H	10.33899000	-11.65917300	8.14482900
P	7.34695600	-10.06364700	4.50043500
P	4.76111500	-9.47071300	6.39675200
C	8.97974200	-9.30255700	4.15226600
C	9.83334000	-9.73774300	3.14053100
C	9.37009300	-8.22256400	4.95211700
C	11.06587400	-9.11713600	2.94669500
H	9.53304000	-10.54070600	2.47733000
C	10.60243700	-7.61151600	4.76597800
H	8.69526500	-7.84825300	5.71639000
C	11.45715300	-8.06073700	3.76085500
H	11.71581300	-9.45870200	2.14668000
H	10.88970700	-6.77374300	5.39437900
H	12.41765800	-7.57838000	3.60589000
C	7.00433700	-11.25795000	3.16580800
C	7.85447800	-12.34561200	2.93650200
C	5.86774500	-11.08520800	2.37288200
C	7.57788200	-13.24823100	1.91643700
H	8.73471200	-12.48802900	3.55870100
C	5.58503200	-12.00674300	1.36706200
H	5.19875400	-10.24346300	2.53773100
C	6.43483600	-13.08302100	1.13548100
H	8.24707000	-14.08529900	1.73826000

H	4.69403300	-11.87126100	0.76210400
H	6.21045300	-13.79527800	0.34679500
C	5.78382100	-9.53958800	7.91859200
C	5.43620500	-10.36798000	8.99052300
C	6.85152600	-8.65383000	8.05625600
C	6.17252500	-10.33378000	10.16683700
H	4.60439200	-11.05906600	8.89516400
C	7.58430500	-8.61130200	9.24079900
H	7.09625100	-7.99031700	7.23114800
C	7.24858800	-9.45550700	10.29370600
H	5.91370400	-11.00113000	10.98257600
H	8.41745400	-7.92117700	9.33538700
H	7.82367200	-9.43286500	11.21447500
C	3.15027400	-9.07031300	7.23429500
C	1.90602100	-9.59908600	6.88304600
C	3.19498300	-8.15387100	8.29722400
C	0.74972800	-9.21313300	7.55500700
H	1.81400900	-10.32710100	6.09018700
C	2.03934500	-7.75752800	8.95822500
H	4.14550500	-7.75995400	8.64117000
C	0.80718900	-8.28477300	8.58657300
H	-0.20249400	-9.63762300	7.25374500
H	2.10989400	-7.04555000	9.77546300
H	-0.09920100	-7.97981400	9.10076200
Cl	6.84560000	-7.65215300	2.61162800
P	3.61638900	-7.66409600	3.63343000
H	4.44574300	-6.82497100	6.76612300
O	3.43978000	-8.99757900	2.95781400
C	2.00863000	-7.32062000	4.47024100

C	0.94714100	-8.11361300	4.01087400
C	1.73110600	-6.30486500	5.38771100
C	-0.35038900	-7.89857600	4.46059600
H	1.15692500	-8.89511000	3.28852500
C	0.43301200	-6.09756000	5.84689600
H	2.52548300	-5.66807600	5.75529200
C	-0.61152700	-6.89030700	5.38408500
H	-1.15895400	-8.52078700	4.08752600
H	0.24174900	-5.31077700	6.57075900
H	-1.62377300	-6.72295600	5.74122300
O	3.67862300	-6.54368900	2.45775900
C	4.01884400	-5.21072600	2.79675100
H	3.13907400	-4.71810400	3.23535000
H	4.27561700	-4.70727300	1.86298700
C	5.18391700	-5.21469800	3.78708100
H	6.12125700	-5.39999400	3.26072500
H	5.23546200	-4.23892000	4.28174100
C	4.91094000	-6.30435600	4.78988900
C	4.82988900	-6.05806800	6.10851500
C	5.20068200	-4.77374700	6.81172800
H	6.16557500	-4.40562200	6.44046300
H	4.46532100	-3.98940400	6.57467300
C	5.26418800	-4.96642200	8.32832000
H	4.27370700	-5.27768500	8.69049500
H	5.94797100	-5.79675900	8.55736000
C	5.71111100	-3.71786800	9.08533400
H	5.02829300	-2.88886500	8.85536600
H	6.70106900	-3.41034500	8.72274800
C	5.76344600	-3.93780800	10.59630000

H	6.09305000	-3.03552600	11.12143400
H	6.45700400	-4.74801300	10.85010600
H	4.77664900	-4.21221700	10.98726800
Cl	8.74716800	-13.19330300	9.90447700
Cl	3.85139800	-15.26542200	3.89958800
O	6.10477500	-13.31314400	8.39309400
O	6.01124100	-14.54534100	5.79178900
C	6.25233700	-14.69939700	8.69318900
H	6.33910500	-14.83281000	9.77470100
H	5.36054400	-15.20514100	8.31928000
H	7.13021800	-15.12193900	8.19832500
C	7.10478200	-14.88652400	4.95415500
H	7.47544800	-15.84506300	5.32231700
H	6.79181700	-14.98662800	3.91065900
H	7.89619800	-14.13287800	5.02794200

(S)-TS4’

E = -4825.31047286 a.u.

0 1

Rh	5.33399500	-8.93995200	4.72973000
C	5.82304200	-14.18864800	4.93691900
C	5.09735000	-14.62308500	3.82832700
C	5.51769900	-12.95271900	5.53104000
C	4.03811500	-13.87469900	3.33558500
C	3.68487700	-12.69554300	3.97237900
C	4.40490800	-12.22871800	5.07439100
H	3.48436400	-14.22267500	2.47087000
H	2.84323600	-12.13030600	3.59278700
C	6.39617800	-12.49089300	6.64941400

C	6.36218900	-13.26097600	7.82853000
C	7.23290800	-11.35850000	6.56710500
C	7.17731300	-12.90636300	8.90695200
C	8.08505600	-11.08466300	7.64085200
C	8.06101900	-11.84570800	8.79906100
H	8.77953700	-10.25732900	7.59600600
H	8.70626400	-11.59624900	9.63427500
P	7.21143600	-10.20126300	5.09911200
P	4.00416600	-10.57282300	5.76285200
C	8.63059400	-9.07334000	5.46382600
C	9.78381600	-9.00647000	4.68107500
C	8.48919700	-8.15812800	6.51800000
C	10.77439000	-8.06247700	4.95038700
H	9.90642600	-9.65872500	3.82509000
C	9.47802400	-7.22313000	6.79227600
H	7.58842800	-8.17166500	7.12519700
C	10.62806500	-7.17125700	6.00549300
H	11.65459800	-8.02066900	4.31596600
H	9.34459500	-6.52632700	7.61413500
H	11.39760100	-6.43318600	6.21033300
C	7.84071800	-11.32764700	3.79185900
C	9.10514500	-11.91999500	3.91653500
C	7.05783200	-11.61195300	2.67324900
C	9.60362200	-12.73197100	2.90523300
H	9.70497700	-11.74674800	4.80637400
C	7.54605900	-12.45963900	1.68028500
H	6.08241700	-11.14363700	2.55805500
C	8.82108400	-13.00445200	1.78268900
H	10.59333100	-13.16885200	3.00234700

H	6.92489900	-12.67357600	0.81640600
H	9.20618000	-13.64637400	0.99574100
C	4.14774900	-10.75771700	7.58082600
C	3.61676300	-11.86255400	8.25527800
C	4.78704200	-9.75559800	8.31127500
C	3.72329300	-11.95888500	9.63756000
H	3.15034300	-12.66711700	7.69459200
C	4.89073200	-9.85000900	9.69654800
H	5.21194700	-8.90719300	7.78062300
C	4.35925800	-10.95154300	10.36067800
H	3.32923800	-12.83211300	10.14755400
H	5.39510100	-9.06512000	10.25273200
H	4.45202400	-11.03486200	11.43944100
C	2.19353500	-10.35626600	5.51276100
C	1.72372400	-10.00714700	4.23968800
C	1.27533200	-10.48201500	6.56078300
C	0.36215300	-9.83861000	4.01703100
H	2.43083400	-9.83654200	3.43123300
C	-0.08634400	-10.29707800	6.33373700
H	1.60687400	-10.71412200	7.56561200
C	-0.54821200	-9.98427500	5.06022300
H	0.01798000	-9.57090400	3.02254100
H	-0.78367300	-10.39756800	7.16047100
H	-1.61073800	-9.84185800	4.88432800
Cl	4.47127200	-9.49807800	2.27672300
P	3.83189600	-6.30666300	5.01054400
C	3.76691100	-5.82333700	6.74306200
C	4.20588900	-4.56503400	7.16454100
C	3.32187700	-6.75906000	7.67860400

C	4.20644400	-4.24974800	8.51824300
H	4.54321400	-3.83319600	6.43543000
C	3.32284300	-6.44029500	9.03342300
H	2.97745600	-7.72976200	7.33548100
C	3.76735100	-5.18896300	9.45174300
H	4.54694200	-3.27274400	8.84754100
H	2.98244400	-7.17419900	9.75762700
H	3.77044600	-4.94034600	10.50914100
O	2.98992000	-5.15429800	4.24318300
C	3.68472000	-4.72345600	3.04800900
H	3.42828900	-5.40452800	2.23055700
H	3.31458200	-3.72202100	2.82349300
C	5.19677100	-4.76809000	3.33615400
H	5.50077800	-3.86856100	3.88866900
H	5.75894200	-4.79645500	2.39896600
C	5.36165400	-6.02347900	4.16204400
C	6.21574200	-7.06447100	4.07444600
C	7.48923300	-6.93551800	3.28109200
H	8.33226600	-6.95580300	3.98181800
H	7.52507800	-5.94316100	2.81165500
C	7.69380900	-8.00549400	2.21013500
H	6.83993800	-8.00998500	1.52429800
H	7.67502300	-8.99341700	2.67589600
C	9.00496300	-7.84348400	1.44323200
H	8.95877800	-6.94733600	0.80950500
H	9.82735800	-7.67262600	2.15231800
C	9.31537800	-9.07200900	0.58841800
H	10.23753300	-8.94227000	0.01115300
H	9.42861200	-9.96519100	1.21605500

H	8.49913100	-9.27416100	-0.11414500
H	6.15001200	-7.75963800	5.45901700
O	3.42001900	-7.75420800	4.87240800
Cl	7.05033900	-13.74107900	10.43236600
Cl	5.51307100	-16.13703000	3.07190300
O	5.44220400	-14.24693200	7.90433400
O	6.76003400	-15.01186800	5.48437700
C	5.86263200	-15.58175300	8.17850000
H	5.54045000	-15.86807500	9.18305200
H	5.39224300	-16.22385600	7.43187500
H	6.94727300	-15.68209500	8.09449600
C	8.09546700	-14.85574300	5.03381700
H	8.64054500	-15.73252400	5.38920500
H	8.14175900	-14.80908800	3.94158300
H	8.54050300	-13.94835700	5.45467200

(S)-2a

E = -1036.51778359 a.u.

0 1

P	-1.44947100	-1.17108700	2.27515100
H	-0.14528700	0.28898900	4.42650400
O	-1.24730400	-2.59172400	2.65344400
C	-2.85883400	-0.42502400	3.12659800
C	-3.61410400	-1.21653700	3.99318300
C	-3.20188800	0.91664800	2.93297300
C	-4.70749200	-0.66962700	4.66026200
H	-3.33524300	-2.25641200	4.13387900
C	-4.29285400	1.46078900	3.59988000
H	-2.61021500	1.53939900	2.26692500

C	-5.04606700	0.66610700	4.46369000
H	-5.29436800	-1.28707600	5.33352500
H	-4.55787400	2.50282000	3.44787900
H	-5.89835900	1.09215000	4.98507700
O	-1.70297600	-0.89514600	0.68367600
C	-0.78918100	0.02605400	0.08212900
H	-1.28408200	1.00186900	0.00137900
H	-0.56996000	-0.33915800	-0.92347800
C	0.46737800	0.12928400	0.96241500
H	1.16042400	-0.68520900	0.71696100
H	0.98763700	1.07649900	0.79373900
C	-0.04085000	-0.03610800	2.37297300
C	0.38939800	0.52437700	3.50463400
C	1.57697500	1.42599000	3.66515700
H	1.95659100	1.74829400	2.68863000
H	1.27069200	2.33278400	4.20507700
C	2.70760700	0.74045900	4.44836900
H	2.32394600	0.40171800	5.42046200
H	3.01896200	-0.16336700	3.90873700
C	3.91407300	1.65396500	4.66677400
H	3.59276900	2.55537200	5.20584500
H	4.29065800	1.99518800	3.69303900
C	5.03963000	0.96800500	5.43919400
H	5.89181000	1.64024900	5.58266900
H	5.39784400	0.08029700	4.90542700
H	4.69656400	0.64455100	6.42866400

(R)-Int2

E = -5331.09705665 a.u.

0 1

Rh	6.48257600	-8.91076500	4.64141500
C	4.80884600	-13.72118900	4.64771600
C	4.05083600	-13.77569800	3.47234300
C	4.90670500	-12.48751600	5.33062500
C	3.34374300	-12.65576400	3.04594500
C	3.41298000	-11.46424900	3.74260500
C	4.21703400	-11.35685100	4.87959500
H	2.76526900	-12.71803600	2.13089100
H	2.86117000	-10.61273800	3.36529800
C	5.79052400	-12.44219900	6.53538000
C	5.32584400	-13.07063400	7.70061900
C	7.02430200	-11.77379300	6.54280600
C	6.08761500	-13.00587300	8.86848400
C	7.78201300	-11.75950200	7.71728500
C	7.31803000	-12.36493900	8.87462800
H	8.74139100	-11.25713700	7.74235700
H	7.89954400	-12.32770800	9.78924400
P	7.58660200	-10.79508700	5.08064400
P	4.57602300	-9.68084800	5.57630700
C	9.34981900	-10.49021300	5.47769300
C	10.36734000	-11.37353700	5.11599200
C	9.66850000	-9.34322000	6.21250600
C	11.68309900	-11.12001900	5.49653200
H	10.14533600	-12.25289800	4.52101800
C	10.97937400	-9.09852300	6.60196500
H	8.88263500	-8.63333700	6.45493800
C	11.99031600	-9.98832500	6.24452900
H	12.46907200	-11.80833200	5.20033300

H	11.21498600	-8.20206900	7.16750500
H	13.01738400	-9.79205900	6.53816100
C	7.56791800	-12.00946100	3.71598400
C	7.91889900	-13.34736000	3.92619500
C	7.17027300	-11.58874300	2.44356700
C	7.87061800	-14.25672000	2.87529200
H	8.19476100	-13.68909200	4.91965100
C	7.12262100	-12.50526800	1.39660000
H	6.89428200	-10.55135800	2.27470700
C	7.46665800	-13.83723800	1.61000900
H	8.13922200	-15.29508200	3.04764000
H	6.81622400	-12.17243100	0.40917000
H	7.41641000	-14.54986200	0.79216200
C	4.40804800	-9.85975900	7.38851000
C	3.40576200	-10.64685700	7.96559600
C	5.29975300	-9.17150700	8.21124500
C	3.30502400	-10.74952700	9.34667700
H	2.72963300	-11.21364900	7.33189000
C	5.19250800	-9.26730100	9.59705300
H	6.09220700	-8.58281400	7.75461000
C	4.19808200	-10.05807500	10.16431300
H	2.54392200	-11.38620700	9.78541400
H	5.89591100	-8.73614000	10.23117700
H	4.12602500	-10.15042500	11.24414300
C	3.08848400	-8.69233500	5.13333700
C	2.99253700	-8.20197300	3.82622500
C	2.08454400	-8.36649100	6.05009500
C	1.89464200	-7.44427400	3.42893000
H	3.78776200	-8.40188800	3.11742000

C	0.99642300	-7.59221700	5.65910300
H	2.15696700	-8.69510000	7.07968300
C	0.89066700	-7.14246800	4.34559200
H	1.83431900	-7.08316000	2.40816100
H	0.23539700	-7.33334500	6.38935400
H	0.03799900	-6.54314900	4.04077900
Cl	8.33145100	-8.17113800	3.31514700
C	4.38906100	-9.01142100	0.74999800
C	3.52861200	-7.95952700	0.41611700
C	2.28935500	-8.27204600	-0.11629400
C	1.86236400	-9.56980400	-0.35304000
C	2.75869600	-10.57687800	-0.02201600
C	4.00720000	-10.33802600	0.52056700
H	3.80534300	-6.92997500	0.61091000
H	0.88509000	-9.78529700	-0.76438700
H	4.65846400	-11.15520800	0.80486000
O	5.58791500	-8.80529900	1.31875400
H	5.66183900	-7.88755000	1.66072900
P	5.85455600	-5.85088500	3.80243000
H	6.11347000	-6.91839400	4.72717900
O	5.27301800	-6.31929900	2.50845300
C	7.33145100	-4.85515400	3.64173500
C	7.78438900	-4.06087100	4.69860200
C	8.04177300	-4.90379300	2.44067500
C	8.94800400	-3.31535900	4.55086000
H	7.22270000	-4.01622000	5.62649500
C	9.20162900	-4.15103300	2.29692900
H	7.68882600	-5.53626300	1.63390700
C	9.65453600	-3.36139700	3.35039500

H	9.30156000	-2.69551400	5.36904400
H	9.75721700	-4.19000900	1.36554000
H	10.56410200	-2.77871400	3.23705700
O	4.94378700	-4.92201300	4.74840100
C	3.99767000	-5.48312400	5.66595800
H	3.06721500	-5.70264900	5.13959500
H	4.39178600	-6.41585100	6.08577200
C	3.76953100	-4.46730100	6.77916000
H	3.38422700	-3.53766500	6.34450400
H	4.73218100	-4.22827600	7.24776800
C	2.83673200	-4.99159500	7.77715500
C	2.06910200	-5.44828300	8.58843000
C	1.14785900	-5.98393900	9.59220200
H	0.11705700	-5.72413500	9.31602200
H	1.34047000	-5.48559800	10.55178700
C	1.25586500	-7.50463400	9.78486800
H	2.29305800	-7.77222600	10.02043400
H	1.01463100	-8.00812900	8.84039000
C	0.31606900	-8.00486300	10.88206600
H	0.53727300	-7.47429100	11.81806500
H	-0.71776100	-7.74325000	10.61848600
C	0.41892000	-9.51031900	11.11583900
H	-0.29423300	-9.84461000	11.87638100
H	0.21527200	-10.06641500	10.19327600
H	1.42495900	-9.78395500	11.45190500
F	2.37714800	-11.85420300	-0.21039600
F	1.44532800	-7.26270400	-0.38967500
Cl	3.96511900	-15.17564000	2.42103000
Cl	5.47344300	-13.67852200	10.35602100

O	5.54431400	-14.70530900	5.21435800
O	4.09129000	-13.63231100	7.66866400
C	5.38011900	-16.08327100	4.94011500
H	5.84314000	-16.36088800	3.99057200
H	5.89343300	-16.59486400	5.75738000
H	4.32599700	-16.37573200	4.93559000
C	3.98472500	-15.03428700	7.87933400
H	4.96655300	-15.51562200	7.85468800
H	3.50776400	-15.23259500	8.84305100
H	3.36461200	-15.43807600	7.07382900

(R)-Int2’

E = -4825.29110324 a.u.

0 1

Rh	6.28489000	-8.70687000	4.01955100
C	4.83610000	-13.52864000	5.28220200
C	3.85553600	-13.84795500	4.33526100
C	5.02125700	-12.17364000	5.62771400
C	3.02125100	-12.85869400	3.82338300
C	3.17998700	-11.53796900	4.20028100
C	4.19908300	-11.18111100	5.08540600
H	2.25999400	-13.13735200	3.10302900
H	2.52777100	-10.78248400	3.77716700
C	6.13109100	-11.82562300	6.56863100
C	5.94036900	-12.13135300	7.92624700
C	7.29766600	-11.16163300	6.15323400
C	6.90763600	-11.75240900	8.85931500
C	8.26726200	-10.83743500	7.10645000
C	8.07483200	-11.12398200	8.44851400

H	9.17791900	-10.33237200	6.80630000
H	8.81922000	-10.84293700	9.18543900
P	7.49539700	-10.50539600	4.43377800
P	4.60737000	-9.40485200	5.33011000
C	9.30060100	-10.20292300	4.32124900
C	10.20169600	-11.21275300	3.97320900
C	9.78795400	-8.93075500	4.64095500
C	11.57003800	-10.96010100	3.95945200
H	9.84265300	-12.19719700	3.69388200
C	11.15688700	-8.68644500	4.63783700
H	9.09484100	-8.12744600	4.87557300
C	12.04998900	-9.69875500	4.29819200
H	12.25940500	-11.75051600	3.67779400
H	11.51880700	-7.69430200	4.88488200
H	13.11806300	-9.50144200	4.28598700
C	7.17211000	-11.94516600	3.35591500
C	7.54761100	-13.24257100	3.71939900
C	6.51755500	-11.73158900	2.14030900
C	7.26442600	-14.31411900	2.88038700
H	8.02148900	-13.42412100	4.67967700
C	6.23428800	-12.80745800	1.30377800
H	6.23393100	-10.72057900	1.86032700
C	6.60380800	-14.09761500	1.67323000
H	7.54486900	-15.32037500	3.17731500
H	5.71977000	-12.63547400	0.36337000
H	6.37000300	-14.93700300	1.02521200
C	4.66170900	-9.17232000	7.14131600
C	3.76406600	-9.79333200	8.01503300
C	5.67147100	-8.35762000	7.65808300

C	3.86428100	-9.58489000	9.38500700
H	3.01329200	-10.47696500	7.62816400
C	5.77085500	-8.15060900	9.03109000
H	6.39057700	-7.91018200	6.97580300
C	4.86280400	-8.75759600	9.89403000
H	3.17335000	-10.08424100	10.05678600
H	6.56574100	-7.52479800	9.42668400
H	4.94570300	-8.60473000	10.96561800
C	3.09809000	-8.51433900	4.78079700
C	3.07087000	-8.06522400	3.45325100
C	2.03735400	-8.18685300	5.62964100
C	2.00491300	-7.30247700	2.98670000
H	3.89546600	-8.30410500	2.78244800
C	0.97400700	-7.42144000	5.16232000
H	2.06190100	-8.47624400	6.67406900
C	0.95511200	-6.97881700	3.84225800
H	2.00737000	-6.94741200	1.96125000
H	0.16920500	-7.15410100	5.84027000
H	0.13032800	-6.37017900	3.48406100
Cl	7.56984400	-7.93280900	2.18870700
P	5.98862900	-5.36379500	4.66312900
H	5.64192400	-6.74628200	4.51530100
O	5.48208600	-4.51402300	3.56170200
C	7.75703400	-5.31456400	4.98005000
C	8.28347800	-5.54346400	6.25548900
C	8.61002500	-5.06762000	3.90206400
C	9.65968600	-5.50816200	6.45277700
H	7.61498400	-5.72385300	7.09116100
C	9.98600400	-5.03569400	4.10475600

H	8.19460100	-4.90964600	2.91292300
C	10.50833700	-5.24608800	5.37832400
H	10.06982200	-5.67358600	7.44454200
H	10.64903000	-4.84793600	3.26645100
H	11.58289000	-5.20630300	5.53616200
O	5.42019400	-5.09071400	6.15439000
C	4.05655700	-5.41747900	6.43205200
H	3.38921300	-4.79013200	5.83282100
H	3.87322700	-6.46351600	6.17552200
C	3.82514200	-5.20844000	7.92499900
H	3.88914100	-4.14096100	8.16463300
H	4.63012400	-5.71579300	8.46890000
C	2.53709500	-5.76356100	8.34121100
C	1.48292200	-6.26015400	8.65495200
C	0.23986600	-6.92139900	9.05644500
H	-0.48874200	-6.87009300	8.23567900
H	-0.21160000	-6.37826500	9.89711000
C	0.46285900	-8.38831700	9.45398800
H	1.19163300	-8.42502100	10.27305400
H	0.92355900	-8.92210600	8.61309900
C	-0.82341500	-9.10036500	9.86685400
H	-1.28139400	-8.56847500	10.71144300
H	-1.54746400	-9.05276000	9.04234500
C	-0.57781800	-10.55907000	10.24969000
H	-1.50724200	-11.05995500	10.53893800
H	-0.14296600	-11.11609000	9.41129900
H	0.11843200	-10.63069700	11.09365900
Cl	3.65494800	-15.45384000	3.66681200
Cl	6.64426100	-12.00256600	10.56572300

O	5.71596300	-14.37971700	5.85979500
O	4.75897500	-12.69883700	8.27761200
C	5.40776800	-15.73591300	6.13239600
H	5.63565200	-16.37627100	5.27768300
H	6.04613200	-16.01542500	6.97346100
H	4.35861500	-15.86384600	6.41564300
C	4.78118400	-13.96197800	8.92729100
H	5.74575400	-14.46013900	8.79085500
H	4.58072100	-13.84856900	9.99591800
H	3.99314500	-14.56727600	8.47127200

(R)-TS1

E = -5331.08711051 a.u.

0 1

Rh	6.42167700	-8.72598000	4.52823200
C	4.83270200	-13.61728600	4.63015200
C	4.08585000	-13.69961900	3.44906400
C	4.92040700	-12.36813800	5.28827000
C	3.39754600	-12.58461900	2.98155000
C	3.46104800	-11.37663300	3.64893200
C	4.23788000	-11.24721900	4.80318400
H	2.82893200	-12.66763000	2.06180200
H	2.91635200	-10.53532800	3.24103000
C	5.79527000	-12.30220800	6.49698400
C	5.33817300	-12.91948500	7.66985200
C	7.02634100	-11.63153700	6.48832000
C	6.10431900	-12.83473200	8.83368000
C	7.78881900	-11.59478300	7.65890100
C	7.33057100	-12.18560900	8.82660100

H	8.74674400	-11.08931200	7.67244500
H	7.91480900	-12.13231100	9.73868300
P	7.57440000	-10.70083700	4.99172200
P	4.51739600	-9.55082500	5.48703100
C	9.32098700	-10.32840900	5.39536200
C	10.37089600	-11.17988700	5.05336500
C	9.59409500	-9.15659600	6.10962900
C	11.67531000	-10.86970900	5.43186300
H	10.18348600	-12.07675300	4.47288100
C	10.89347500	-8.85497300	6.49579900
H	8.78345300	-8.46978400	6.33679200
C	11.93827200	-9.71294700	6.15749800
H	12.48725700	-11.53329000	5.14986300
H	11.09385300	-7.93867300	7.04281500
H	12.95652500	-9.47199400	6.44812400
C	7.57973200	-11.97157900	3.68244600
C	7.98882000	-13.28375200	3.94435800
C	7.12782100	-11.62747500	2.40515600
C	7.95349800	-14.24133200	2.93666900
H	8.30500900	-13.56666900	4.94439200
C	7.08709000	-12.59377800	1.40390200
H	6.80957300	-10.61101000	2.19058700
C	7.49598600	-13.89824700	1.66637400
H	8.27551700	-15.25742300	3.14633400
H	6.73929600	-12.32078500	0.41197700
H	7.45595700	-14.64874200	0.88259900
C	4.41982200	-9.69653800	7.30420600
C	3.46185000	-10.50732100	7.92354500
C	5.29869700	-8.95008900	8.08938200

C	3.39010000	-10.57210500	9.30846400
H	2.79862800	-11.12119800	7.32099600
C	5.21970000	-9.00897400	9.47910500
H	6.05751800	-8.34112700	7.60464700
C	4.26823200	-9.82051500	10.08808000
H	2.66372800	-11.22542300	9.78005600
H	5.91094000	-8.42948400	10.08329300
H	4.21722600	-9.88093500	11.17124100
C	2.96895000	-8.65818800	5.06382000
C	2.82991300	-8.16255500	3.76415800
C	1.96259200	-8.39702400	5.99806200
C	1.69410700	-7.44989700	3.39298000
H	3.62753200	-8.30230200	3.04605500
C	0.83730000	-7.66542300	5.63363800
H	2.06510200	-8.72990100	7.02320700
C	0.69513000	-7.19900100	4.32949300
H	1.60147700	-7.08079900	2.37727200
H	0.07628600	-7.45151900	6.37821900
H	-0.18447900	-6.62844800	4.04710700
Cl	8.30978400	-8.31062800	3.09037200
C	4.32465400	-9.08120200	0.70578100
C	3.45809200	-8.01447900	0.43905900
C	2.21441100	-8.30105800	-0.09683300
C	1.78545700	-9.58506800	-0.39846700
C	2.68804100	-10.60633100	-0.13516900
C	3.94398000	-10.39275300	0.40034700
H	3.73440800	-6.99718000	0.69162100
H	0.80276300	-9.77964600	-0.80726200
H	4.60325100	-11.22189300	0.62467700

O	5.52355600	-8.90593300	1.27970400
H	5.60536900	-8.00058400	1.66339000
P	5.93344100	-6.31007800	3.93199900
H	6.18856500	-7.29569700	5.27886900
O	5.27254500	-6.51980500	2.59352000
C	7.46052300	-5.35669200	3.84748400
C	8.17512000	-5.07664200	5.01216300
C	7.91499000	-4.89597100	2.61470100
C	9.34744500	-4.33463500	4.94298900
H	7.81880900	-5.44284500	5.97220700
C	9.08529500	-4.14580100	2.54938100
H	7.35773300	-5.13903200	1.71602400
C	9.80147400	-3.86826900	3.71006900
H	9.90791800	-4.11849400	5.84773900
H	9.44403700	-3.78790400	1.58924700
H	10.71930300	-3.29004600	3.65543800
O	5.11076300	-5.30094000	4.91203200
C	4.05888000	-5.70883500	5.77449800
H	3.14171900	-5.86935000	5.20295000
H	4.32709700	-6.64487700	6.27846800
C	3.85234900	-4.62038900	6.82352200
H	3.54640400	-3.69059300	6.33073700
H	4.80794300	-4.42066000	7.32309000
C	2.84703500	-5.05145100	7.79576400
C	2.02283700	-5.47245000	8.57012300
C	1.04789200	-5.99901800	9.52646100
H	0.03139000	-5.84140000	9.14142200
H	1.11264100	-5.42553100	10.46086000
C	1.25516800	-7.49075700	9.83174200

H	2.28566400	-7.65168200	10.17021800
H	1.14646100	-8.06757200	8.90480800
C	0.26949100	-8.01174400	10.87668400
H	0.37876200	-7.42848000	11.80093700
H	-0.75712600	-7.84065100	10.52530600
C	0.46329800	-9.49522100	11.18399600
H	-0.26043600	-9.84744400	11.92616000
H	0.34139500	-10.10168100	10.27890600
H	1.46900900	-9.68433600	11.57653000
F	2.30753300	-11.87397800	-0.38094200
F	1.36448300	-7.28021800	-0.30489000
Cl	3.97661200	-15.13112900	2.44440200
Cl	5.49801800	-13.49175800	10.33081600
O	5.55781700	-14.58292500	5.23703600
O	4.10707300	-13.48909500	7.65027600
C	5.48361700	-15.96270300	4.93462000
H	5.96509400	-16.19053900	3.98114300
H	6.03018800	-16.45252200	5.74340900
H	4.45120600	-16.32351900	4.92515700
C	4.01225900	-14.89152200	7.87000800
H	4.99911300	-15.36264800	7.86215600
H	3.52527600	-15.08471100	8.82965900
H	3.40730000	-15.30889300	7.05995100

(R)-TS1'

E = -4825.27291511 a.u.

0 1

Rh	6.34379200	-8.62029000	4.44379600
C	4.81464800	-13.56346800	4.90964300

C	3.95197300	-13.73789200	3.82036600
C	4.96164600	-12.26493000	5.44963400
C	3.22299200	-12.65934800	3.32651200
C	3.34686100	-11.39992500	3.88038100
C	4.22950500	-11.18726600	4.94210000
H	2.55598600	-12.82374800	2.48749400
H	2.76236300	-10.58392400	3.47307800
C	5.94828500	-12.08608800	6.55829500
C	5.61486500	-12.59451500	7.82214000
C	7.15940700	-11.40343700	6.36990900
C	6.47868800	-12.37665200	8.89750900
C	8.02031000	-11.23164900	7.45668900
C	7.68034000	-11.70611900	8.71479400
H	8.96091500	-10.70820200	7.32932700
H	8.33976700	-11.54892700	9.56134000
P	7.53798000	-10.59216000	4.75677800
P	4.53770300	-9.45889700	5.50669200
C	9.31861800	-10.18848200	4.91056700
C	10.32786200	-11.11167200	4.62932400
C	9.66919800	-8.90755800	5.35071700
C	11.66483900	-10.76186900	4.79820900
H	10.08138500	-12.09986800	4.25641600
C	11.00380700	-8.56092900	5.52073600
H	8.89511600	-8.16708300	5.52722800
C	12.00504200	-9.48906500	5.24694700
H	12.44155100	-11.48473300	4.56695400
H	11.25523700	-7.55405900	5.83974900
H	13.04934100	-9.21659600	5.36870200
C	7.40560000	-11.95944500	3.55275800

C	7.83918000	-13.25500500	3.85452700
C	6.80940300	-11.70298700	2.31531500
C	7.68602300	-14.27834300	2.92551600
H	8.26247000	-13.47462800	4.83053600
C	6.64311700	-12.73364400	1.39522900
H	6.48783100	-10.69361600	2.07641600
C	7.08038800	-14.02010800	1.69781700
H	8.03036000	-15.28048200	3.16499500
H	6.17055700	-12.52868400	0.43971700
H	6.94561500	-14.82321300	0.97947300
C	4.43798300	-9.52475800	7.32711400
C	3.47372700	-10.30740400	7.97221300
C	5.33820400	-8.77830300	8.08935800
C	3.40902400	-10.33525300	9.35913600
H	2.80099200	-10.93082700	7.39035700
C	5.26540600	-8.80061300	9.47943700
H	6.10646700	-8.19582100	7.58841800
C	4.30151200	-9.57721300	10.11429100
H	2.67356500	-10.96289600	9.85107000
H	5.97143300	-8.21992600	10.06486200
H	4.25503900	-9.60769300	11.19867300
C	3.03827200	-8.54670100	4.98215700
C	3.02572600	-8.00466000	3.69197000
C	1.96116800	-8.29964900	5.83819800
C	1.94794900	-7.23548400	3.26449000
H	3.87722900	-8.13785000	3.03098300
C	0.88949800	-7.52469500	5.41084900
H	1.97339400	-8.67127500	6.85576900
C	0.87927200	-6.99430600	4.12260900

H	1.96930100	-6.79876900	2.27186500
H	0.07166700	-7.31941100	6.09500400
H	0.04785600	-6.37695400	3.79569300
Cl	7.81311400	-8.25916200	2.56587400
P	5.97298800	-6.10998300	4.31306600
H	6.17156100	-7.42075400	5.48910600
O	5.15547900	-5.86870600	3.08901000
C	7.64387600	-5.41833800	4.31661900
C	8.39621800	-5.39720000	5.49311700
C	8.19871800	-4.96288600	3.12255000
C	9.70133000	-4.91765900	5.47512600
H	7.95723900	-5.74750000	6.42401000
C	9.50492000	-4.48147600	3.10811700
H	7.60790900	-5.00335900	2.21363100
C	10.25633000	-4.46219500	4.27968100
H	10.28295400	-4.89104100	6.39266400
H	9.94015000	-4.13157800	2.17703300
H	11.27716500	-4.09098000	4.26326400
O	5.41464500	-5.29579100	5.63226600
C	4.16976000	-5.68010600	6.19390200
H	3.38023600	-5.66560300	5.43503100
H	4.24297300	-6.69641100	6.59785100
C	3.83793800	-4.72406700	7.33574600
H	3.67125700	-3.71673100	6.93823900
H	4.70214300	-4.66690000	8.00852600
C	2.66316700	-5.20564900	8.06401200
C	1.70803200	-5.67790800	8.63054200
C	0.58551000	-6.29190700	9.34126400
H	-0.31568000	-6.25523700	8.71403200

H	0.35548900	-5.70457700	10.24027200
C	0.87360600	-7.74708200	9.74179700
H	1.79387000	-7.78018400	10.33744300
H	1.08005000	-8.33467500	8.83880100
C	-0.27693800	-8.38556400	10.51752200
H	-0.46963900	-7.80273700	11.42820800
H	-1.19575700	-8.33215700	9.91788700
C	0.00470500	-9.83994000	10.89073300
H	-0.82631600	-10.27808200	11.45291500
H	0.16228700	-10.44987200	9.99326800
H	0.90718700	-9.91715400	11.50870700
Cl	3.72751800	-15.25287900	2.96770300
Cl	6.03442200	-12.89988200	10.50083800
O	5.59803300	-14.47740300	5.52147800
O	4.40972300	-13.20153600	7.96328500
C	5.52142800	-15.87626300	5.32497100
H	5.89936100	-16.16465200	4.34168900
H	6.16451900	-16.29890000	6.09972200
H	4.50233200	-16.25088400	5.45605600
C	4.39198200	-14.57533500	8.33165100
H	5.38476100	-15.02435900	8.23647800
H	4.04082200	-14.68360700	9.36141500
H	3.70107300	-15.08313000	7.65274300

(R)-Int3

E = -5331.10422071 a.u.

0 1

Rh	6.42936900	-8.64622100	4.40846600
C	4.77802800	-13.52550300	4.71988300

C	4.01575700	-13.62247900	3.54954400
C	4.91180500	-12.26104200	5.34126300
C	3.34495000	-12.50662300	3.06271300
C	3.44395600	-11.28422800	3.69865300
C	4.24327700	-11.13739900	4.83582700
H	2.76941400	-12.59858000	2.14831900
H	2.91051200	-10.44244000	3.27704900
C	5.82253700	-12.19229300	6.52318300
C	5.39034500	-12.77235600	7.72466500
C	7.07688600	-11.57017500	6.45314100
C	6.19490900	-12.67534100	8.86131000
C	7.87798400	-11.52302900	7.59764300
C	7.43757100	-12.06090300	8.79722900
H	8.85395100	-11.05395900	7.56292200
H	8.05134300	-11.99808300	9.68908300
P	7.60564700	-10.71621700	4.90676600
P	4.49996100	-9.41071200	5.46023100
C	9.38215000	-10.40189900	5.22857800
C	10.37428000	-11.31271400	4.86281100
C	9.75038600	-9.22683500	5.89358700
C	11.70927200	-11.05951000	5.16891900
H	10.11653300	-12.21619600	4.32111700
C	11.08083700	-8.98041200	6.20718500
H	8.99664100	-8.48772000	6.14347100
C	12.06474800	-9.89783900	5.84544700
H	12.47186900	-11.77195800	4.86906300
H	11.35085400	-8.05929400	6.71435400
H	13.10660400	-9.69993900	6.07914900
C	7.54744100	-12.01759200	3.62947300

C	7.87262300	-13.34800700	3.91384400
C	7.14837700	-11.66357300	2.33730400
C	7.79461200	-14.31497200	2.91749500
H	8.15780600	-13.63448500	4.92182700
C	7.06686100	-12.63746100	1.34561200
H	6.90751800	-10.62965100	2.10544400
C	7.38447200	-13.96172500	1.63400900
H	8.04838400	-15.34622300	3.14564600
H	6.75578100	-12.35687500	0.34366000
H	7.31146600	-14.71906700	0.85944500
C	4.46504300	-9.49738400	7.28428600
C	3.58427200	-10.36946600	7.93427600
C	5.27334800	-8.65354600	8.04784900
C	3.52567200	-10.40654000	9.32118400
H	2.97312300	-11.05613500	7.35644900
C	5.20506700	-8.68290800	9.43826200
H	5.96074100	-7.97107500	7.55738500
C	4.33622800	-9.56189500	10.07586500
H	2.86205700	-11.11081700	9.81134700
H	5.83876300	-8.02136200	10.02043900
H	4.29448700	-9.59675600	11.16034300
C	2.90254700	-8.60296200	5.04434900
C	2.76348700	-8.03288700	3.77795800
C	1.84338500	-8.50522500	5.95069600
C	1.58084800	-7.40233800	3.40611900
H	3.59663600	-8.04150000	3.09116100
C	0.66960200	-7.85393100	5.58837200
H	1.93587900	-8.90375200	6.95301500
C	0.53091100	-7.30873600	4.31424200

H	1.49607500	-6.97480200	2.41214100
H	-0.13655700	-7.76828100	6.31094200
H	-0.38776000	-6.80146100	4.03573700
Cl	8.32883800	-8.25170600	3.01042500
C	4.35067100	-9.00837600	0.92829700
C	3.50589700	-7.98049200	0.49300600
C	2.37557400	-8.32503800	-0.22750800
C	2.04565200	-9.63299500	-0.55351700
C	2.92605800	-10.61224300	-0.11745600
C	4.07283000	-10.33895200	0.60590400
H	3.71165900	-6.94725600	0.75088600
H	1.15024700	-9.87512900	-1.11070200
H	4.71637600	-11.13834500	0.94954300
O	5.42969200	-8.75247600	1.69286600
H	5.47524300	-7.77240500	1.89341800
P	5.95156000	-6.37564200	3.95004000
H	6.89741200	-8.11157300	5.74115300
O	5.22185800	-6.28155000	2.61410600
C	7.47542900	-5.37967100	3.91272200
C	8.26896400	-5.25276500	5.05306600
C	7.84784500	-4.74116600	2.73333700
C	9.43078800	-4.49214200	5.01458600
H	7.97739500	-5.75459800	5.97304000
C	9.01015200	-3.97456700	2.69586700
H	7.22599800	-4.85456700	1.85097600
C	9.80275900	-3.85187600	3.83293200
H	10.04802400	-4.39610900	5.90360800
H	9.30028100	-3.47825700	1.77433800
H	10.71258300	-3.25909900	3.80038500

O	5.26679300	-5.47204500	5.13970900
C	4.11860700	-5.82009800	5.87974600
H	3.22683400	-5.82150300	5.24547900
H	4.23578500	-6.81633000	6.31368200
C	3.95893100	-4.81479400	7.01812700
H	3.78371100	-3.81463400	6.60625400
H	4.89724600	-4.76985200	7.58419400
C	2.85643700	-5.22924500	7.88642800
C	1.94448100	-5.65350600	8.55308500
C	0.87098000	-6.21177300	9.37591900
H	-0.06702100	-6.21421700	8.80419000
H	0.69740800	-5.56452800	10.24593300
C	1.18606800	-7.63781100	9.85185100
H	2.13248800	-7.63030800	10.40574100
H	1.35771300	-8.27739100	8.97760100
C	0.07763500	-8.23304000	10.71768400
H	-0.06651300	-7.60504200	11.60706200
H	-0.87172200	-8.20645000	10.16557500
C	0.37726100	-9.66855500	11.14598200
H	-0.41568900	-10.07053800	11.78494000
H	0.47087100	-10.32415500	10.27236500
H	1.31980400	-9.72230600	11.70376400
F	2.63578700	-11.89877700	-0.38571000
F	1.54085000	-7.34471300	-0.60644900
Cl	3.87611600	-15.07393700	2.58016400
Cl	5.62075700	-13.26913200	10.39711900
O	5.48865500	-14.49727900	5.33420700
O	4.14709400	-13.31600200	7.75529400
C	5.31178200	-15.88433400	5.11544900

H	5.76197900	-16.20176600	4.17252600
H	5.83208400	-16.36580300	5.94617500
H	4.25588700	-16.16879300	5.13579300
C	4.02460500	-14.70321700	8.04526300
H	4.99765800	-15.20154800	8.01920700
H	3.57301000	-14.84058200	9.03127600
H	3.37495000	-15.13826500	7.28062400

(R)-Int3'

E = -4825.2946838 a.u.

0 1

Rh	6.30016800	-8.43935000	4.51376800
C	4.92785900	-13.34316400	4.87349200
C	4.10004600	-13.54186500	3.76230300
C	5.04720700	-12.03510000	5.40408400
C	3.38695400	-12.47569900	3.22046400
C	3.47220500	-11.21096100	3.76583600
C	4.30005600	-10.97774300	4.86684200
H	2.76066100	-12.65465000	2.35378000
H	2.90530600	-10.40353400	3.31945700
C	6.02354100	-11.85235400	6.52524800
C	5.68439100	-12.36334700	7.78747800
C	7.25867500	-11.21139900	6.33857600
C	6.54430500	-12.15015600	8.86675000
C	8.11915100	-11.05162500	7.42595800
C	7.75907700	-11.50198300	8.68736700
H	9.07659000	-10.55934800	7.29558400
H	8.41646900	-11.35249800	9.53695000
P	7.65689600	-10.48410200	4.69123900

P	4.45125900	-9.23667000	5.45156600
C	9.43808500	-10.05230800	4.80084900
C	10.45432400	-10.88137700	4.31396500
C	9.78773600	-8.82634400	5.38157500
C	11.78886400	-10.50371700	4.42882700
H	10.20924500	-11.82087600	3.83147100
C	11.12335400	-8.46001900	5.51044000
H	9.01450800	-8.14728200	5.73070100
C	12.12773100	-9.29829800	5.03621000
H	12.56499600	-11.15649300	4.04048600
H	11.37150100	-7.50711000	5.96625900
H	13.17016400	-9.00789500	5.12829300
C	7.59745800	-11.94601200	3.59565800
C	8.11408100	-13.18064800	4.00946400
C	7.01156600	-11.82987200	2.33295000
C	8.06093800	-14.28037200	3.16121500
H	8.54364400	-13.28503600	5.00203100
C	6.94287700	-12.94149700	1.49590600
H	6.59656900	-10.87633100	2.01773500
C	7.46722900	-14.16331900	1.90497500
H	8.47411600	-15.23184200	3.48441700
H	6.47241700	-12.84781700	0.52207800
H	7.41121900	-15.02704200	1.24883700
C	4.35184200	-9.32125500	7.27434700
C	3.46518400	-10.22276100	7.87469900
C	5.10203200	-8.46287300	8.07936300
C	3.34282900	-10.27247600	9.25752400
H	2.89925400	-10.92038100	7.26528500
C	4.97049700	-8.50904500	9.46391200

H	5.78320100	-7.75316300	7.62100400
C	4.09709100	-9.41609700	10.05477700
H	2.67166800	-10.99494100	9.70998200
H	5.55836100	-7.83614800	10.08054400
H	4.00832200	-9.46167000	11.13594300
C	2.88744900	-8.41842800	4.95769800
C	2.80651400	-7.80854500	3.70148400
C	1.79149100	-8.34807400	5.82844900
C	1.63941700	-7.15034200	3.32305400
H	3.65597100	-7.82673900	3.02604000
C	0.63423100	-7.68278000	5.44607800
H	1.84257500	-8.78675400	6.81736900
C	0.55544800	-7.08324400	4.19050100
H	1.59900100	-6.66816400	2.35204200
H	-0.20229700	-7.62548500	6.13652600
H	-0.34632600	-6.55410900	3.89649000
Cl	5.82756600	-8.57465300	2.08252900
P	5.75284700	-6.21358200	4.59983400
H	6.77199800	-8.27685000	5.98278200
O	4.82290300	-5.53315600	3.65348000
C	7.53577300	-5.85979600	4.38554800
C	8.33766500	-5.42370400	5.45129800
C	8.11663200	-6.09716400	3.13277600
C	9.69306400	-5.19925800	5.25264900
H	7.88321900	-5.24326400	6.41951400
C	9.48377900	-5.89314000	2.94938500
H	7.49759200	-6.44176100	2.30928400
C	10.26874800	-5.44148000	4.00333500
H	10.30645100	-4.83459800	6.07225200

H	9.92934500	-6.08588700	1.97850100
H	11.33270100	-5.27858200	3.85668000
O	5.56186200	-5.67455200	6.14391900
C	4.22531300	-5.56224100	6.62265500
H	3.64008500	-4.91171600	5.96621800
H	3.74806300	-6.54649500	6.63384700
C	4.26973700	-5.02154500	8.05206100
H	4.50082500	-3.95061400	8.04998300
H	5.08172600	-5.52628700	8.58988000
C	2.99598700	-5.30477000	8.71327400
C	1.93461200	-5.65824100	9.16538700
C	0.68440500	-6.21044800	9.68799500
H	-0.14708200	-5.92686600	9.02889100
H	0.45925300	-5.78116300	10.67298900
C	0.76499600	-7.74164900	9.79145700
H	1.57691700	-8.00796100	10.47873100
H	1.05914000	-8.14339800	8.81411400
C	-0.53841500	-8.39395200	10.24471600
H	-0.83232600	-7.99011700	11.22285000
H	-1.34330800	-8.12698500	9.54639500
C	-0.41851700	-9.91499700	10.33093000
H	-1.36067000	-10.37662000	10.64376900
H	-0.14057200	-10.33994800	9.35893200
H	0.35449300	-10.20513200	11.05249000
Cl	3.90177200	-15.07402000	2.93393400
Cl	6.08458700	-12.66053800	10.47072800
O	5.69679500	-14.24201000	5.52491700
O	4.48266400	-12.97943000	7.92207700
C	5.68149300	-15.63987600	5.31147500

H	6.09330400	-15.90132800	4.33453800
H	6.32534200	-16.04340900	6.09592000
H	4.67627000	-16.05713900	5.41802600
C	4.47382800	-14.35141800	8.29757600
H	5.47265400	-14.79027100	8.22162900
H	4.10692800	-14.45687100	9.32214300
H	3.80006100	-14.87053400	7.61021500

(R)-Int4

E = -4825.28330573 a.u.

0 1

Rh	8.65818500	-10.25255500	5.73990700
C	4.05284800	-13.05168000	6.61145900
C	3.13159300	-12.08042000	7.01202500
C	5.42897200	-12.84192900	6.84565900
C	3.56274600	-10.90359700	7.60597200
C	4.91173700	-10.71018100	7.85354000
C	5.85982200	-11.68980600	7.53311400
H	2.83974300	-10.13659300	7.86167500
H	5.22064300	-9.77114000	8.29507100
C	6.35784800	-13.87045900	6.27544200
C	6.31318600	-15.15687500	6.83085400
C	7.20264700	-13.62045800	5.17266200
C	7.14923400	-16.15947700	6.33765200
C	8.01642400	-14.64799300	4.69232500
C	8.00344500	-15.90842300	5.27461000
H	8.67123800	-14.47095800	3.84723800
H	8.64491100	-16.69748900	4.89786200
P	7.26214500	-11.95174100	4.37474900

P	7.65115500	-11.33810700	7.83051700
C	8.03175500	-12.21711100	2.72940800
C	7.32428600	-12.16110600	1.52170200
C	9.42740600	-12.33504500	2.69545600
C	8.00015500	-12.24683900	0.30827000
H	6.24801300	-12.03272000	1.51608400
C	10.09576700	-12.42809600	1.47927500
H	10.00503900	-12.29994700	3.61413500
C	9.38552700	-12.38746700	0.28415000
H	7.43945400	-12.19609800	-0.62063200
H	11.17863000	-12.48790100	1.47894800
H	9.91044100	-12.44358000	-0.66504200
C	5.47178400	-11.75765800	3.98574000
C	4.79304800	-12.79444100	3.33135000
C	4.75584300	-10.61789100	4.35765400
C	3.44575400	-12.67213900	3.01654700
H	5.32002500	-13.71030300	3.07984000
C	3.40538600	-10.49613200	4.04319600
H	5.24395900	-9.83766000	4.92882400
C	2.74978600	-11.51743400	3.36475500
H	2.93456300	-13.48854600	2.51613600
H	2.86088600	-9.60855700	4.35141900
H	1.69380800	-11.42575800	3.12993200
C	8.33793500	-12.83871800	8.68717600
C	8.24210800	-12.97016400	10.08005800
C	9.00220500	-13.84093100	7.97573200
C	8.78401000	-14.07121300	10.73461300
H	7.75228000	-12.20917800	10.67517400
C	9.54333600	-14.94369500	8.63040600

H	9.12614200	-13.75534500	6.90724400
C	9.44055700	-15.06349100	10.01132200
H	8.69797900	-14.14554700	11.81468300
H	10.05297300	-15.70516300	8.04829200
H	9.87116900	-15.92005800	10.52156200
C	7.75866700	-10.17368200	9.25699100
C	6.79745100	-10.00430800	10.26037500
C	8.99070700	-9.52166800	9.39601200
C	7.04268100	-9.16376900	11.34368200
H	5.85647600	-10.54265600	10.21671500
C	9.24098200	-8.69606900	10.48626100
H	9.76333500	-9.66422600	8.64601400
C	8.26246700	-8.50334300	11.45768100
H	6.27983700	-9.03711800	12.10610800
H	10.20114500	-8.19575600	10.56698400
H	8.45213900	-7.84812600	12.30243500
Cl	10.49651000	-11.77869700	6.08927600
P	10.23738600	-9.15599900	4.30706600
H	9.45886200	-9.32959700	6.66410000
O	11.15174900	-10.03297900	3.51809600
C	11.11294500	-7.93168900	5.34923800
C	11.88042600	-8.40551200	6.41983800
C	11.01304000	-6.55732400	5.13171300
C	12.51303700	-7.51187900	7.27689400
H	11.96346200	-9.47708600	6.58475600
C	11.65310100	-5.66445300	5.98990400
H	10.42476200	-6.18831200	4.29921500
C	12.39534800	-6.13787700	7.06693700
H	13.10486500	-7.88811600	8.10672900

H	11.56528800	-4.59519000	5.81775100
H	12.88524500	-5.43941300	7.73961400
O	9.45757300	-8.07664600	3.32397400
C	8.41210700	-8.62682800	2.55008500
H	8.34173400	-8.04955500	1.62518900
H	8.62949000	-9.66628400	2.28294000
C	7.10097200	-8.52682600	3.34034900
H	6.36316800	-9.22945400	2.93879900
H	6.68386100	-7.51680700	3.24649900
C	7.33475300	-8.74971100	4.78030200
C	7.34838800	-8.48224800	5.99290200
C	7.13472400	-7.60908900	7.16436800
H	6.88699100	-8.19400900	8.05345700
H	6.26499200	-6.97650300	6.94151900
C	8.35399100	-6.72797500	7.46310900
H	8.59131500	-6.12604700	6.57884300
H	9.22877800	-7.36576200	7.63508600
C	8.12941500	-5.82412700	8.67285500
H	7.30732500	-5.12546700	8.46479300
H	7.81042400	-6.43645600	9.52626400
C	9.38865800	-5.04560400	9.04721900
H	9.21771100	-4.40081900	9.91589300
H	10.21030500	-5.73137000	9.28418100
H	9.72442500	-4.41613600	8.21554200
Cl	1.43196700	-12.26868100	6.67358100
Cl	7.11512000	-17.73902300	7.06760900
O	3.69438500	-14.14603500	5.90645100
O	5.41324900	-15.45801100	7.80553400
C	2.82171100	-15.10217400	6.50482500

H	1.83554200	-15.04424000	6.03704100
H	3.26380700	-16.08561000	6.33603700
H	2.73228700	-14.93774000	7.58155400
C	5.81350700	-15.18903600	9.14418900
H	5.06514200	-15.66340400	9.78119600
H	6.80020400	-15.61193600	9.35411100
H	5.83232900	-14.11158800	9.33529700

(R)-TS3

E = -4825.27216136 a.u.

0 1

Rh	8.39475400	-10.16049500	5.82271000
C	3.99193600	-12.97384500	6.46619300
C	3.06208200	-12.00595300	6.85863200
C	5.34928000	-12.80786800	6.81038000
C	3.47184200	-10.86454700	7.53318500
C	4.80779700	-10.69917700	7.86235500
C	5.75297800	-11.68596800	7.55697700
H	2.74407300	-10.09862200	7.77889400
H	5.11337600	-9.77762800	8.34258900
C	6.29818100	-13.85717900	6.31395400
C	6.14265700	-15.14012700	6.86111300
C	7.26343500	-13.64591700	5.30413800
C	6.98421400	-16.17976300	6.46636200
C	8.06140400	-14.71986000	4.90436200
C	7.94387500	-15.97265300	5.48912600
H	8.79097700	-14.58820000	4.11552400
H	8.58175600	-16.79090100	5.17344600
P	7.43423100	-12.00077500	4.44376700

P	7.53351200	-11.36502900	7.87635900
C	8.45653500	-12.34054400	2.96356700
C	7.98575200	-12.15499600	1.65990100
C	9.80442900	-12.66699000	3.15613700
C	8.83150800	-12.34871900	0.57227900
H	6.96153800	-11.84903100	1.47833200
C	10.63643900	-12.88864900	2.06694100
H	10.21757400	-12.70184800	4.15933000
C	10.15262100	-12.73396400	0.77244900
H	8.45200700	-12.19559200	-0.43362900
H	11.67882800	-13.13808400	2.23665500
H	10.81010500	-12.88762300	-0.07817800
C	5.71579100	-11.91476900	3.79264000
C	5.23393600	-12.90062800	2.92099100
C	4.83699900	-10.93525900	4.24497500
C	3.92501700	-12.85413300	2.45952800
H	5.88532900	-13.70995500	2.60509800
C	3.52461900	-10.88448500	3.78381700
H	5.16781400	-10.24822800	5.01372600
C	3.07007000	-11.83706300	2.87982200
H	3.56693600	-13.62213000	1.78080700
H	2.85088600	-10.11957800	4.15873300
H	2.04451700	-11.80518300	2.52473700
C	8.20746700	-12.90594000	8.64705000
C	7.87867600	-13.21927900	9.97191600
C	9.08747800	-13.74212600	7.95922300
C	8.39366500	-14.35737400	10.58181100
H	7.22156100	-12.57137300	10.54291800
C	9.59103800	-14.88955900	8.56505600

H	9.39785100	-13.48647500	6.95667800
C	9.24535000	-15.20316200	9.87472400
H	8.12726600	-14.58268500	11.61040300
H	10.26559200	-15.53156600	8.00721800
H	9.64552100	-16.09511300	10.34765300
C	7.68891000	-10.28116700	9.35302400
C	6.72043000	-10.04274000	10.33216200
C	8.97615300	-9.75538000	9.53219700
C	7.01669600	-9.25018900	11.43977200
H	5.73272100	-10.48414200	10.25093000
C	9.27327200	-8.98022100	10.64668100
H	9.74849900	-9.96179300	8.79368500
C	8.28993700	-8.71207400	11.59663200
H	6.24982400	-9.06449400	12.18595500
H	10.27366700	-8.57510500	10.76582500
H	8.51912700	-8.09484300	12.46010100
Cl	10.54024600	-11.30907100	6.29748200
P	9.52585800	-9.03854900	4.06966800
H	9.00571500	-9.03810100	6.76361300
O	10.38543400	-9.88526200	3.19513600
C	10.46379000	-7.64468500	4.79983600
C	11.52240900	-7.93964100	5.66538900
C	10.18744200	-6.31217900	4.48421700
C	12.28125700	-6.91310000	6.21787900
H	11.73677700	-8.97573100	5.91216600
C	10.95851300	-5.28670400	5.02869400
H	9.37369300	-6.08235000	3.80584800
C	12.00325600	-5.58492100	5.89925200
H	13.09746400	-7.15104700	6.89407500

H	10.74088400	-4.25357600	4.77194200
H	12.60288000	-4.78526300	6.32566200
O	8.45713500	-8.13338800	3.18180400
C	7.11336600	-8.51684900	3.01990500
H	6.73938300	-8.01652600	2.12244100
H	7.02098000	-9.59784600	2.86501000
C	6.30921200	-8.08140300	4.24532900
H	5.27342100	-8.41537500	4.15307800
H	6.30438600	-6.98610000	4.29597200
C	6.98385500	-8.58402000	5.47140800
C	7.52383700	-8.22197500	6.55271000
C	7.62995600	-7.28811500	7.69938100
H	7.28493600	-7.78526400	8.61322200
H	6.91247600	-6.48347700	7.48740700
C	9.02434600	-6.70308500	7.92313600
H	9.35415800	-6.18417200	7.01817700
H	9.73706500	-7.52157700	8.08151500
C	9.06759100	-5.75204200	9.11653100
H	8.39656100	-4.90173500	8.93295000
H	8.68132600	-6.26667900	10.00640800
C	10.48098100	-5.24145100	9.38818700
H	10.50246500	-4.55119600	10.23812800
H	11.15923100	-6.07426100	9.60938400
H	10.88177500	-4.72033200	8.51201000
Cl	1.38779600	-12.13522600	6.38708700
Cl	6.80330400	-17.75456500	7.18489800
O	3.67641000	-14.00824900	5.66156500
O	5.11435700	-15.40310300	7.71428000
C	2.71959400	-14.97286800	6.09301100

H	1.80480600	-14.86976400	5.50398600
H	3.16444400	-15.95663000	5.93173700
H	2.49246400	-14.86027700	7.15582700
C	5.36917400	-15.21309600	9.09818400
H	4.51956800	-15.65257200	9.62384600
H	6.29378300	-15.71324600	9.40216900
H	5.44072900	-14.14646000	9.33583900

(R)-Int5

E = -4825.34308687 a.u.

0 1

Rh	8.64425200	-10.51059000	5.37917100
C	4.20590600	-13.72624500	6.49016200
C	3.12368900	-13.21731300	7.21297300
C	5.46788400	-13.13218400	6.63540800
C	3.30487500	-12.18297200	8.11951100
C	4.55338300	-11.59256500	8.25043300
C	5.63821000	-12.01995000	7.48109400
H	2.46020100	-11.82148600	8.69591600
H	4.66852900	-10.77457000	8.95143300
C	6.63573400	-13.80974700	5.99050800
C	7.01086400	-15.03210600	6.57746200
C	7.43539000	-13.27154400	4.96567400
C	8.20634800	-15.65157700	6.20898400
C	8.67201000	-13.87098100	4.67708400
C	9.05355600	-15.05625600	5.28733800
H	9.38455600	-13.36794700	4.02652700
H	10.01110400	-15.50853300	5.05442700
P	7.10934300	-11.66739200	4.12029400

P	7.27332200	-11.14742800	7.53641200
C	7.19287900	-11.92568800	2.29642100
C	6.39460300	-11.09620500	1.49530100
C	8.04936000	-12.83104800	1.66637000
C	6.46386100	-11.15965700	0.10935400
H	5.69652800	-10.39974300	1.94678300
C	8.10703500	-12.90176500	0.27822000
H	8.69134900	-13.48384000	2.23682800
C	7.32388100	-12.06363400	-0.50598200
H	5.83707200	-10.50406400	-0.48763400
H	8.78173600	-13.61355600	-0.18681100
H	7.38012300	-12.11604100	-1.58912400
C	5.33730900	-11.26647300	4.29231000
C	4.36759500	-12.14015900	3.78861500
C	4.94586200	-10.06068900	4.87300800
C	3.02001800	-11.82686600	3.90442500
H	4.66513500	-13.07991900	3.33378800
C	3.59335600	-9.74391000	4.97784500
H	5.70055400	-9.37228200	5.24448000
C	2.63110400	-10.62914700	4.50276900
H	2.27235600	-12.52468900	3.54161100
H	3.29448000	-8.80405000	5.43319600
H	1.57643100	-10.38843700	4.59639700
C	8.19312700	-12.22538600	8.69791300
C	7.79721000	-12.35233500	10.03407600
C	9.26698800	-12.98133800	8.22719500
C	8.47406400	-13.21562400	10.88785100
H	6.95982200	-11.76977600	10.40934300
C	9.93521900	-13.85778800	9.07855400

H	9.59576600	-12.87791300	7.19863400
C	9.54429300	-13.97130600	10.40905900
H	8.16675300	-13.30036000	11.92594900
H	10.76701600	-14.44267900	8.69913600
H	10.07253900	-14.64701200	11.07520600
C	6.97195400	-9.60481600	8.47014900
C	5.91148100	-8.75356600	8.13284800
C	7.94562500	-9.14543300	9.36436700
C	5.80905100	-7.48822700	8.70045400
H	5.15882800	-9.07488900	7.42035500
C	7.84892700	-7.87177500	9.91760500
H	8.80029600	-9.76817500	9.60367000
C	6.78153200	-7.04045300	9.59068300
H	4.97499200	-6.84588000	8.43329300
H	8.61912500	-7.52820600	10.60115900
H	6.71049100	-6.04636900	10.02201500
Cl	10.37209400	-9.99870800	6.97196000
P	10.13546300	-10.43100600	3.66212600
H	8.38401800	-8.07695300	6.84085700
O	10.55359500	-11.83077200	3.30509300
C	11.59302500	-9.35927800	3.88432200
C	12.79632700	-9.80756600	3.33435600
C	11.53935300	-8.11100900	4.50878500
C	13.93279100	-9.00648400	3.39631500
H	12.83109000	-10.78737400	2.86827700
C	12.67611600	-7.31101500	4.56372400
H	10.61806700	-7.77967400	4.97637800
C	13.87266900	-7.75650200	4.00709000
H	14.86736600	-9.36084700	2.97088600

H	12.63032700	-6.34414300	5.05688700
H	14.76090600	-7.13280500	4.05749900
O	9.40999000	-9.75676900	2.35071900
C	8.85163700	-8.45903100	2.43414800
H	9.63272800	-7.72723700	2.68070300
H	8.48126300	-8.23514400	1.43004300
C	7.69739300	-8.34920300	3.43428900
H	6.88834700	-9.00684300	3.10261100
H	7.30991500	-7.32645900	3.35581800
C	8.06650900	-8.65009000	4.86190300
C	8.04027500	-7.75975200	5.86035900
C	7.60789200	-6.31545100	5.82019100
H	6.62762300	-6.22539200	6.31230600
H	7.47851900	-5.94655400	4.79640700
C	8.60274400	-5.41917500	6.56501400
H	9.57964300	-5.46627400	6.06405600
H	8.75574600	-5.82325100	7.57508300
C	8.14236900	-3.96564700	6.66506500
H	7.98065400	-3.56225400	5.65613800
H	7.16614200	-3.93309400	7.16896300
C	9.13597800	-3.08167500	7.41702100
H	8.78449100	-2.04643300	7.48132700
H	9.29144600	-3.44893100	8.43824400
H	10.11118700	-3.07504000	6.91665000
Cl	8.65938700	-17.15531400	6.95680100
Cl	1.51495900	-13.83054500	6.93837000
O	6.19092600	-15.63953500	7.47751400
O	4.08212900	-14.70773900	5.56912000
C	6.42030100	-15.32825600	8.84970600

H	7.48397900	-15.39263400	9.09736800
H	6.05213500	-14.32486200	9.08467900
H	5.86311300	-16.07038100	9.42347400
C	3.62923800	-15.99602400	5.98603700
H	3.56842900	-16.06234900	7.07428700
H	2.64932000	-16.19543300	5.54494600
H	4.35938400	-16.72589200	5.62996900

(R)-TS4

E = -4825.31527124 a.u.

0 1

Rh	8.54836700	-10.66907900	5.45157300
C	4.08902800	-13.55429700	6.77266100
C	3.13823600	-12.87474400	7.53777200
C	5.42516400	-13.11651900	6.78386800
C	3.51235200	-11.81553900	8.35039500
C	4.82950600	-11.38286900	8.35152200
C	5.79402500	-11.99387800	7.54474600
H	2.76564000	-11.31211200	8.95474400
H	5.09456000	-10.54024300	8.97760100
C	6.40600900	-13.93827300	6.00941600
C	6.64095100	-15.23422200	6.50180600
C	7.07244700	-13.51211200	4.84619800
C	7.57378600	-16.06335800	5.87887900
C	7.98359400	-14.37705200	4.23225400
C	8.24653100	-15.63789900	4.74399000
H	8.51029600	-14.06962500	3.33858900
H	8.96488100	-16.29081400	4.26097900
P	6.99500300	-11.77568200	4.21599700

P	7.51794900	-11.31807800	7.46026000
C	7.21048600	-11.93461200	2.38987900
C	6.14033900	-11.79636100	1.49616900
C	8.49441600	-12.15158300	1.87479500
C	6.34867900	-11.89206700	0.12387400
H	5.13872200	-11.59945000	1.85936600
C	8.69595100	-12.25287200	0.50167500
H	9.35399300	-12.19201500	2.53857100
C	7.62613100	-12.12592000	-0.37778000
H	5.50692700	-11.77951200	-0.55311300
H	9.70250200	-12.40799600	0.12650300
H	7.78688300	-12.19706400	-1.44964500
C	5.24809300	-11.25109900	4.35595800
C	4.18752200	-12.11333500	4.06257600
C	4.97418500	-9.93808500	4.74264900
C	2.87375500	-11.67343500	4.17493100
H	4.38560700	-13.14499500	3.78616700
C	3.65854200	-9.49544100	4.84930200
H	5.79980700	-9.26994800	4.97229700
C	2.60749500	-10.36416000	4.57035900
H	2.05871200	-12.36198600	3.97619100
H	3.45623700	-8.47408500	5.15847400
H	1.58039000	-10.02565400	4.66786200
C	8.42548000	-12.51913100	8.49586400
C	8.13791600	-12.69244200	9.85318400
C	9.39484800	-13.31914000	7.88778200
C	8.83067800	-13.63948600	10.60004900
H	7.37087800	-12.08625800	10.32873700
C	10.07131600	-14.28204400	8.63143300

H	9.63128400	-13.17154400	6.83748800
C	9.79764200	-14.43605600	9.98798400
H	8.61306500	-13.75967900	11.65720400
H	10.82214100	-14.90117500	8.15085100
H	10.33648300	-15.17791200	10.57003500
C	7.40133800	-9.75170700	8.40784800
C	6.53414500	-8.75712100	7.93742300
C	8.25176000	-9.45459900	9.47406300
C	6.49643200	-7.50468800	8.53815600
H	5.87746900	-8.96628800	7.09917900
C	8.21759700	-8.19676500	10.07106100
H	8.96904300	-10.18983900	9.81960600
C	7.34128200	-7.22036400	9.60905700
H	5.81546100	-6.74649700	8.16169600
H	8.89229200	-7.97886300	10.89317600
H	7.32145700	-6.23880500	10.07365800
Cl	10.35839100	-10.11549700	7.00670400
P	10.21624000	-9.89510500	3.81443300
H	9.18105800	-8.02623900	6.19369800
O	10.79008800	-11.28514600	3.76984700
C	11.51818100	-8.70246600	4.26200500
C	12.71026200	-9.22697000	4.76346900
C	11.40746100	-7.32590400	4.05280400
C	13.77260400	-8.38100700	5.06672600
H	12.78639600	-10.29662300	4.92218900
C	12.47094600	-6.48176300	4.35404500
H	10.48951300	-6.89980700	3.66192900
C	13.65425100	-7.00885900	4.86629400
H	14.69244200	-8.79593600	5.46779800

H	12.37501700	-5.41245700	4.18815500
H	14.48306100	-6.34932000	5.10760200
O	9.81487900	-9.50027200	2.27716300
C	8.78310400	-8.57625700	1.98123900
H	9.22082100	-7.57406400	1.88532500
H	8.36899300	-8.86982400	1.01276200
C	7.72284500	-8.59875000	3.08283700
H	7.00778300	-9.39769500	2.88217800
H	7.15922600	-7.66035100	3.07291800
C	8.42844900	-8.80087000	4.40329500
C	8.55691200	-7.82715500	5.32538400
C	7.91677600	-6.46249900	5.30192000
H	6.85652500	-6.55392800	5.58550500
H	7.92263900	-6.03545300	4.28965100
C	8.61225600	-5.50570000	6.27188400
H	9.66189500	-5.38689300	5.96950700
H	8.63161100	-5.96858600	7.26734800
C	7.93634000	-4.13949200	6.36129400
H	7.91843600	-3.67078700	5.36780500
H	6.88564700	-4.27704000	6.65404800
C	8.62511600	-3.20782600	7.35699800
H	8.12374100	-2.23581100	7.41150100
H	8.62602600	-3.64431900	8.36270600
H	9.66904000	-3.03303100	7.07260600
Cl	7.87806100	-17.65025400	6.52481300
Cl	1.45010400	-13.29660800	7.42380000
O	5.91475800	-15.72591800	7.54215100
O	3.75828000	-14.55085100	5.91963600
C	6.40299200	-15.43479400	8.84703800

H	7.48180900	-15.60606800	8.91091900
H	6.18227200	-14.39868100	9.12033700
H	5.88158900	-16.11394400	9.52364300
C	3.18844500	-15.75165700	6.44026300
H	3.21389400	-15.76249700	7.53192700
H	2.15652700	-15.84173300	6.09141600
H	3.78847400	-16.58417400	6.06702300

(R)-2a

E = -1036.51812494 a.u.

0 1

P	6.83762400	-6.70484700	-4.32950800
H	6.12410400	-5.41436600	-1.82167000
O	6.90984700	-8.14625900	-3.98469400
C	8.35428400	-5.81772500	-3.90009600
C	9.44583000	-6.54907200	-3.42884800
C	8.45512500	-4.42947300	-4.03594400
C	10.63123600	-5.89666300	-3.09968400
H	9.35184100	-7.62553800	-3.32284800
C	9.63864500	-3.77995900	-3.70583800
H	7.60301000	-3.85315900	-4.38710800
C	10.72761700	-4.51494700	-3.23846200
H	11.47884700	-6.46781300	-2.73321800
H	9.71373200	-2.70176300	-3.81097600
H	11.65195700	-4.00669600	-2.97962700
O	6.56276000	-6.37986700	-5.90860800
C	5.44419800	-5.52447200	-6.15595800
H	5.81427700	-4.50359000	-6.31367300
H	4.96608000	-5.86920400	-7.07526100

C	4.50202200	-5.57498100	-4.94184000
H	3.84074000	-6.44689600	-5.02100300
H	3.87674500	-4.67878800	-4.89365800
C	5.42267400	-5.72923500	-3.75691200
C	5.30987100	-5.23091900	-2.52479900
C	4.18979600	-4.38219900	-2.00113400
H	3.82214200	-4.81736500	-1.06178500
H	3.34327200	-4.37987000	-2.69717500
C	4.64848600	-2.94001700	-1.73742100
H	5.00302000	-2.50028100	-2.67985300
H	5.51324400	-2.95069200	-1.05961000
C	3.54566100	-2.06326400	-1.14378300
H	2.68026200	-2.06264900	-1.82002400
H	3.19931900	-2.50801400	-0.20140300
C	4.00362400	-0.62704800	-0.89583400
H	3.19830300	-0.01975100	-0.47018300
H	4.84928200	-0.59916700	-0.19902700
H	4.32592900	-0.15055200	-1.82896800

(S)-2a-conformer 1

E = -1036.51930781 a.u.

0 1

O	-1.35306600	0.96694000	-0.45768100
C	-0.63390500	2.01982800	-1.11126700
C	-1.53462300	3.26450800	-1.11812700
H	0.29788900	2.21396400	-0.56754500
H	-0.39078800	1.66505200	-2.11450700
H	-0.94852800	4.17597500	-1.27014700
H	-2.26055800	3.19118300	-1.93890500

P	-2.26733600	1.50865900	0.78641500
C	-3.87799500	0.77099300	0.43206200
C	-4.35149700	0.63801900	-0.87645900
C	-4.68033000	0.37511700	1.50430800
C	-5.61925400	0.11717500	-1.10920400
H	-3.71966800	0.92445100	-1.71222000
C	-5.94904200	-0.14722000	1.26865800
H	-4.29507200	0.46710800	2.51533700
C	-6.41883200	-0.27322600	-0.03600300
H	-5.98269900	0.00882000	-2.12675800
H	-6.56918200	-0.45942300	2.10351700
H	-7.40844400	-0.68175000	-0.21906300
O	-1.81776900	1.25874200	2.17896400
C	-2.72158000	4.23784300	0.94639700
H	-3.16520200	3.99881900	1.91411400
C	-2.24072500	3.22983100	0.21660500
C	-2.75206600	5.68682900	0.56272900
H	-2.27403400	6.27795600	1.35623500
H	-2.17689700	5.85920900	-0.35445200
C	-4.18959200	6.19252300	0.36567300
H	-4.67076000	5.60545800	-0.42782300
H	-4.76910200	6.00707700	1.28049700
C	-4.25173800	7.67974600	0.01650000
H	-3.66754600	7.86212200	-0.89553100
H	-3.76662800	8.25868800	0.81372100
C	-5.68177500	8.17903300	-0.18278300
H	-5.70072000	9.24495300	-0.43207200
H	-6.17829600	7.63524700	-0.99474500
H	-6.27786800	8.03611100	0.72589100

(S)-2a-conformer 2

E = -1036.51597524 a.u.

0 1

O	-1.25509100	0.69473200	0.13696600
C	-0.37430500	1.58742500	-0.55374900
C	-1.20626900	2.78131100	-1.04601500
H	0.41638500	1.91746000	0.13022500
H	0.07840700	1.02224900	-1.37060500
H	-0.56279800	3.63398300	-1.26827100
H	-1.73205600	2.50717300	-1.97038600
P	-2.40684000	1.48881300	0.98296800
C	-3.91991100	0.65878800	0.44631400
C	-4.08747100	0.21782200	-0.86956500
C	-4.95893700	0.50632000	1.36681300
C	-5.28640500	-0.36673600	-1.26152700
H	-3.27229900	0.31511800	-1.58074700
C	-6.15834800	-0.08006200	0.97224600
H	-4.81090100	0.83617800	2.39071800
C	-6.32279800	-0.51329900	-0.34074600
H	-5.41193000	-0.71448500	-2.28259800
H	-6.96286800	-0.20254500	1.69114700
H	-7.25845300	-0.97175100	-0.64759400
O	-2.29657400	1.56832300	2.46147700
C	-2.84865000	4.14171800	0.46021100
H	-3.48201500	4.02677300	1.34192600
C	-2.19846700	3.04339900	0.06540300
C	-2.88624500	5.53527000	-0.10277600
H	-3.93879500	5.75121900	-0.34316200

H	-2.63979200	6.22891600	0.71462200
C	-2.02265200	5.86555700	-1.31861400
H	-0.96137900	5.75573700	-1.06129200
H	-2.22934300	5.15506300	-2.12948800
C	-2.26358300	7.28868100	-1.82565500
H	-2.07048500	8.00063100	-1.01205600
H	-3.32332800	7.40355300	-2.08999800
C	-1.39374500	7.63993500	-3.03126000
H	-1.58538900	8.66068400	-3.37735200
H	-0.32866500	7.56468000	-2.78301600
H	-1.58926300	6.95969200	-3.86840700

(S)-2a-conformer 3

E = -1036.51948581 a.u.

0 1

O	-1.59547300	1.10269300	-0.92478400
C	-0.81732800	2.19790500	-1.42450100
C	-1.50410500	3.49640100	-0.97000800
H	0.20215100	2.13044600	-1.02697400
H	-0.78035100	2.09404900	-2.50996900
H	-0.79972800	4.33495000	-0.94525000
H	-2.30419400	3.75734100	-1.67510200
P	-2.10313000	1.38554500	0.60447000
C	-3.82854900	0.84992800	0.58764400
C	-4.74124100	1.37988100	-0.33018600
C	-4.26105500	-0.06304200	1.54946900
C	-6.07688600	0.99952300	-0.28394500
H	-4.40696100	2.09284200	-1.07953000
C	-5.59923900	-0.44831100	1.59009600

H	-3.54272100	-0.46038300	2.26021200
C	-6.50510300	0.08368300	0.67727500
H	-6.78452200	1.41415800	-0.99563700
H	-5.93431000	-1.16077000	2.33778800
H	-7.54924100	-0.21324400	0.71345000
O	-1.32347900	0.78952600	1.71872600
C	-2.60330200	4.03010600	1.28258400
H	-3.00235800	3.61734800	2.20880000
C	-2.08346800	3.18437900	0.39151800
C	-2.78936600	5.50588300	1.06377000
H	-2.84372100	6.02164300	2.02954400
H	-1.93027200	5.92248500	0.52398200
C	-4.07396200	5.78871900	0.25978800
H	-4.18322300	6.87410500	0.13519400
H	-3.96090000	5.36920200	-0.74937400
C	-5.34082100	5.21393200	0.89696900
H	-5.44862200	5.61171600	1.91526100
H	-5.23831900	4.12568000	0.99570200
C	-6.59630500	5.52608700	0.08512500
H	-7.48940200	5.10318800	0.55632500
H	-6.74743900	6.60740500	-0.01353900
H	-6.52142600	5.10740700	-0.92568200

(S)-2a-conformer 4

E = -1036.52074849 a.u.

0 1

O	-1.35292400	0.70311400	-0.22112100
C	-0.60509000	1.63217900	-1.01588400
C	-1.54438100	2.78908900	-1.38820900

H	0.25151300	1.99701000	-0.43690700
H	-0.23734900	1.08428100	-1.88541600
H	-0.97884200	3.67815700	-1.68250600
H	-2.17882700	2.49431900	-2.23472400
P	-2.43127800	1.44031000	0.76460900
C	-3.96108500	0.55454600	0.39230000
C	-4.25501600	0.10913900	-0.89973000
C	-4.88543200	0.35962700	1.42097400
C	-5.46624100	-0.52150500	-1.16072900
H	-3.52708600	0.23849100	-1.69531600
C	-6.09689800	-0.27330200	1.15740000
H	-4.63869500	0.69332100	2.42436700
C	-6.38801000	-0.71042200	-0.13221200
H	-5.68994700	-0.87229400	-2.16377900
H	-6.81185000	-0.42883000	1.95969400
H	-7.33311500	-1.20508000	-0.33679900
O	-2.15193900	1.51351800	2.22089000
C	-2.90215700	4.13678700	0.32622700
H	-3.40630000	4.09825800	1.29297000
C	-2.37641700	3.00579000	-0.14722000
C	-2.81937400	5.49413900	-0.30900200
H	-2.50674500	5.41368800	-1.35681900
H	-3.82000000	5.94451700	-0.31018900
C	-1.85176200	6.42274300	0.44674900
H	-1.94669900	7.43754900	0.03923800
H	-2.15350500	6.48098200	1.50155200
C	-0.39190300	5.97494600	0.36076600
H	-0.09082800	5.93765900	-0.69593500
H	-0.30223200	4.95221800	0.74839000

C	0.55287400	6.89774000	1.12823900
H	1.59150800	6.56135300	1.04662100
H	0.50063600	7.92384100	0.74570500
H	0.29293100	6.92485600	2.19255200

(S)-2a-conformer 5

E = -1036.5186163 a.u.

0 1

O	-1.39040400	0.81860900	-0.53262300
C	-0.65519100	1.81006000	-1.26029900
C	-1.50276300	3.09126800	-1.28802200
H	0.30521800	1.98686200	-0.76236400
H	-0.46810400	1.40199500	-2.25524400
H	-0.88569200	3.96890400	-1.50329700
H	-2.26373500	3.01434700	-2.07608400
P	-2.22941100	1.45297200	0.72077700
C	-3.88245200	0.77049800	0.46417400
C	-4.41642700	0.60375900	-0.81691400
C	-4.65364100	0.45371800	1.58443900
C	-5.71309800	0.12796700	-0.97487800
H	-3.80990500	0.82850500	-1.68947700
C	-5.95145900	-0.02366700	1.42369200
H	-4.22208400	0.57112800	2.57389500
C	-6.48124600	-0.18350000	0.14602900
H	-6.12383000	-0.00682700	-1.97105400
H	-6.54753000	-0.27459700	2.29591300
H	-7.49354900	-0.55698000	0.02139400
O	-1.73345900	1.24438000	2.10445200
C	-2.56274900	4.20343200	0.77543800

H	-2.97596000	4.02590700	1.76950500
C	-2.15487400	3.14485400	0.07328600
C	-2.54653800	5.63486100	0.33040400
H	-2.00059100	6.22790000	1.07553400
H	-2.01124600	5.74213600	-0.62065900
C	-3.97214200	6.19119000	0.17675500
H	-4.52480200	5.54762200	-0.51889800
H	-4.49195800	6.12238800	1.14237500
C	-4.01933800	7.63794300	-0.32503300
H	-5.06817000	7.91171300	-0.49301600
H	-3.52585000	7.69704300	-1.30464100
C	-3.38473800	8.65068800	0.62884800
H	-3.52358300	9.67323500	0.26310600
H	-3.83758300	8.58677900	1.62545800
H	-2.30756800	8.48660200	0.74069800

(S)-2a-conformer 6

E = -1036.51499732 a.u.

0 1

O	-1.27641000	0.63772700	-0.14430800
C	-0.43910800	1.56814200	-0.83928000
C	-1.27422100	2.82324800	-1.13415800
H	0.42719300	1.81312100	-0.21362100
H	-0.09040600	1.06993100	-1.74581000
H	-0.62613500	3.67590900	-1.34205600
H	-1.89720400	2.65080100	-2.02205600
P	-2.30746800	1.37906500	0.88515000
C	-3.89431000	0.63636300	0.44188800
C	-4.21252400	0.32224900	-0.88253300

C	-4.83561600	0.42315100	1.45112000
C	-5.46386300	-0.19701600	-1.19436900
H	-3.47339600	0.46665600	-1.66521900
C	-6.08784100	-0.09758300	1.13658200
H	-4.57081900	0.65409400	2.47855300
C	-6.40235400	-0.40455700	-0.18458500
H	-5.70659500	-0.44651500	-2.22299700
H	-6.81638200	-0.26759000	1.92360100
H	-7.37909300	-0.81195400	-0.42912200
O	-2.04145900	1.32169900	2.34476100
C	-2.73413600	4.07303900	0.63511300
H	-3.28948800	3.88767000	1.55657300
C	-2.14551000	3.00491400	0.08918100
C	-2.80194300	5.51039500	0.19836500
H	-3.86134500	5.72114400	-0.00891100
H	-2.55764500	6.13281200	1.07184400
C	-1.94520800	5.95946700	-0.98479300
H	-0.89048000	5.76284700	-0.75780500
H	-2.19790300	5.36826200	-1.87468900
C	-2.10417400	7.44918400	-1.31235200
H	-1.36971600	7.71392900	-2.08273700
H	-1.84645800	8.04397900	-0.42568000
C	-3.49980600	7.83826200	-1.80214700
H	-3.52794800	8.88997300	-2.10485600
H	-3.79380300	7.23250600	-2.66766000
H	-4.26010000	7.70188800	-1.02575000

(S)-2a-conformer 7

E = -1036.51925172 a.u.

0 1

O	-1.46975200	0.82136000	-0.84513000
C	-0.64230300	1.89195200	-1.31811700
C	-1.35059800	3.21064300	-0.96892000
H	0.34008200	1.83228500	-0.83526000
H	-0.51707400	1.74745800	-2.39239500
H	-0.64528000	4.04794000	-0.93278800
H	-2.10030400	3.44213600	-1.73704400
P	-2.12722100	1.16605700	0.61253900
C	-3.85019500	0.66127200	0.41794700
C	-4.60053100	1.05681800	-0.69380800
C	-4.45072300	-0.08442400	1.43282500
C	-5.94276500	0.70936800	-0.78735800
H	-4.13150000	1.63025500	-1.48888200
C	-5.79514700	-0.43530800	1.33492500
H	-3.85486400	-0.38574700	2.28911800
C	-6.53982400	-0.03698500	0.22857400
H	-6.52393400	1.01530400	-1.65209500
H	-6.26061200	-1.01880900	2.12349000
H	-7.58881600	-0.30869300	0.15447600
O	-1.49280200	0.59637700	1.82802500
C	-2.51340600	3.84784100	1.22430200
H	-2.97997600	3.47246300	2.13522000
C	-2.02265600	2.95733700	0.36081100
C	-2.55637500	5.33437500	1.00477500
H	-2.61647300	5.84777500	1.97051900
H	-1.63079900	5.67370200	0.52385500
C	-3.75139200	5.74582700	0.11972800
H	-3.77972000	6.84194800	0.06217200

H	-3.57260600	5.38236800	-0.90029000
C	-5.10938300	5.22046200	0.59844600
H	-5.11453300	4.12391800	0.54366700
H	-5.87891500	5.56567200	-0.10293500
C	-5.48179500	5.66480700	2.01278900
H	-6.49269400	5.33573700	2.27344900
H	-4.79934100	5.24757100	2.76198300
H	-5.44907300	6.75710900	2.10501100

(S)-2a-conformer 8

E = -1036.51745385 a.u.

0 1

O	-1.46809700	0.51483600	-0.54044500
C	-0.82443100	1.22685800	-1.60541800
C	-1.79545100	2.31278900	-2.09512500
H	0.10398000	1.67416600	-1.23191700
H	-0.57998500	0.49364600	-2.37614900
H	-1.26682300	3.10142000	-2.63922600
H	-2.53515900	1.86978700	-2.77548900
P	-2.39567800	1.48550000	0.39496300
C	-3.95960400	0.58475700	0.45596000
C	-4.44796300	-0.11285800	-0.65270300
C	-4.71211900	0.63604300	1.63126100
C	-5.68177900	-0.75006100	-0.58638500
H	-3.85360800	-0.17226000	-1.55985500
C	-5.94654500	-0.00420700	1.69552800
H	-4.31468300	1.16682200	2.49108600
C	-6.43173000	-0.69370400	0.58741000
H	-6.05684200	-1.29643500	-1.44661400

H	-6.52774300	0.03207400	2.61197200
H	-7.39462000	-1.19367300	0.63862500
O	-1.90984600	1.88085800	1.74227300
C	-2.89490000	4.03738300	-0.54400800
H	-3.28075200	4.21351800	0.45990300
C	-2.46179800	2.81014100	-0.83561500
C	-2.83271200	5.22961000	-1.45513900
H	-2.91061700	4.90966900	-2.50086800
H	-3.69526700	5.87783500	-1.25672900
C	-1.54326300	6.05892500	-1.28293500
H	-0.68330700	5.45441300	-1.60221100
H	-1.60643100	6.90236200	-1.98133800
C	-1.28805400	6.58882000	0.13831800
H	-2.24446400	6.88024700	0.59446200
H	-0.69960600	7.51141900	0.06302100
C	-0.54234400	5.61953100	1.06076000
H	-0.36162900	6.08013500	2.03786200
H	-1.08338100	4.68426400	1.23030400
H	0.43302100	5.35947000	0.63229200

12. Quantum chemical ECD calculation method

All calculations were carried out by Gaussian 16, Revision A.03 package[5]. Density functional theory (DFT) method ωB97X-D [6] along with 6-31G* basis set were used for geometry optimization calculations by using Gaussian09 defaults settings. Eight conformers of (*S*)-**2a** were located. WB97XD /6-311G** calculations were performed on se veral stable optimized conformers (which are not higher in free energy than the most stable one by ~3 kcal/mol: six lowest-energy conformers for (*S*)-**2a** to obtain overall ECD spectra by including ECD spectra of each conformer weighted by Boltzmann distribution.
Multiwfn 3.8[7]

Table S7. Energies (Hartree) of the optimized conformers of (*S*)-**2a** at WB97XD/6-31G(d) level.

Conformer	Electronic energy (E)	E + ZPE	Free energy (G)
**1*S***	-1036.5193078	-1036.210345	-1036.25571
**2*S***	-1036.5159752	-1036.206684	-1036.25086
**3*S***	-1036.5194858	-1036.210344	-1036.25477
**4*S***	-1036.5186163	-1036.211497	-1036.25450
**5*S***	-1036.5149973	-1036.209493	-1036.25011
**6*S***	-1036.5192517	-1036.205716	-1036.25440
**7*S***	-1036.5174539	-1036.209861	-1036.25259
**8*S***	-1036.5207485	-1036.208067	-1036.25585

Table S8. Relative energies (kcal/mol) of the optimized conformers of (*S*)-**2a** at WB97XD/6-311G(d,p) level.

Conformer	ΔE	ΔE+ZPE	ΔG	Boltzmann distribution factor at 298.15 K
**1*S***	0.0000000	0.0000000	0.0000000	41.46%
**2*S***	2.0912193	2.297312279	3.0478136	0.23%
**3*S***	-0.1116967	0.000627509	0.5923690	7.93%
**4*S***	0.4339291	-0.722890944	0.7599140	31.12%
**5*S***	2.7048734	0.534638094	3.5134257	12.09%
**6*S***	0.0351970	2.904741475	0.8226650	0.11%
**7*S***	1.1633775	0.303714598	1.9597122	6.59%
**8*S***	-0.9040404	1.429466641	-0.0878513	0.47%

**Calculated ECD Data** wavelength (nm)	Δε of *S*-**2a**
190	-17.15
191	-19.33
192	-21.09
193	-22.24
194	-22.85
195	-22.94
196	-22.51
197	-21.59
198	-20.22
199	-18.44
200	-16.31
201	-13.89
202	-11.23
203	-8.42
204	-5.52
205	-2.59
206	0.30
207	3.08
208	5.71
209	8.26
210	10.44
211	12.36
212	13.99
213	15.33
214	16.37
215	17.13
216	17.62
217	17.85
218	17.84
219	17.64
220	17.25
221	16.72
222	16.06
223	15.31
224	14.49
225	13.58
226	12.69
227	11.79
228	10.90
229	10.04

230	9.20
231	8.40
232	7.65
233	6.94
234	6.28
235	5.66
236	5.10
237	4.58
238	4.11
239	3.68
240	3.29
241	2.91
242	2.60
243	2.31
244	2.05
245	1.82
246	1.61
247	1.43
248	1.26
249	1.11
250	0.98
251	0.86
252	0.76
253	0.66
254	0.58
255	0.51
256	0.44
257	0.38
258	0.33
259	0.29
260	0.25

Cartesian Coordinates of optimized conformers

1S

0 1

O	-1.35306600	0.96694000	-0.45768100
C	-0.63390500	2.01982800	-1.11126700
C	-1.53462300	3.26450800	-1.11812700
H	0.29788900	2.21396400	-0.56754500
H	-0.39078800	1.66505200	-2.11450700
H	-0.94852800	4.17597500	-1.27014700
H	-2.26055800	3.19118300	-1.93890500
P	-2.26733600	1.50865900	0.78641500
C	-3.87799500	0.77099300	0.43206200

C	-4.35149700	0.63801900	-0.87645900
C	-4.68033000	0.37511700	1.50430800
C	-5.61925400	0.11717500	-1.10920400
H	-3.71966800	0.92445100	-1.71222000
C	-5.94904200	-0.14722000	1.26865800
H	-4.29507200	0.46710800	2.51533700
C	-6.41883200	-0.27322600	-0.03600300
H	-5.98269900	0.00882000	-2.12675800
H	-6.56918200	-0.45942300	2.10351700
H	-7.40844400	-0.68175000	-0.21906300
O	-1.81776900	1.25874200	2.17896400
C	-2.72158000	4.23784300	0.94639700
H	-3.16520200	3.99881900	1.91411400
C	-2.24072500	3.22983100	0.21660500
C	-2.75206600	5.68682900	0.56272900
H	-2.27403400	6.27795600	1.35623500
H	-2.17689700	5.85920900	-0.35445200
C	-4.18959200	6.19252300	0.36567300
H	-4.67076000	5.60545800	-0.42782300
H	-4.76910200	6.00707700	1.28049700
C	-4.25173800	7.67974600	0.01650000
H	-3.66754600	7.86212200	-0.89553100
H	-3.76662800	8.25868800	0.81372100
C	-5.68177500	8.17903300	-0.18278300
H	-5.70072000	9.24495300	-0.43207200
H	-6.17829600	7.63524700	-0.99474500
H	-6.27786800	8.03611100	0.72589100

2S

0 1

O	-1.25509100	0.69473200	0.13696600
C	-0.37430500	1.58742500	-0.55374900
C	-1.20626900	2.78131100	-1.04601500
H	0.41638500	1.91746000	0.13022500
H	0.07840700	1.02224900	-1.37060500
H	-0.56279800	3.63398300	-1.26827100
H	-1.73205600	2.50717300	-1.97038600
P	-2.40684000	1.48881300	0.98296800
C	-3.91991100	0.65878800	0.44631400
C	-4.08747100	0.21782200	-0.86956500
C	-4.95893700	0.50632000	1.36681300
C	-5.28640500	-0.36673600	-1.26152700
H	-3.27229900	0.31511800	-1.58074700
C	-6.15834800	-0.08006200	0.97224600

H	-4.81090100	0.83617800	2.39071800
C	-6.32279800	-0.51329900	-0.34074600
H	-5.41193000	-0.71448500	-2.28259800
H	-6.96286800	-0.20254500	1.69114700
H	-7.25845300	-0.97175100	-0.64759400
O	-2.29657400	1.56832300	2.46147700
C	-2.84865000	4.14171800	0.46021100
H	-3.48201500	4.02677300	1.34192600
C	-2.19846700	3.04339900	0.06540300
C	-2.88624500	5.53527000	-0.10277600
H	-3.93879500	5.75121900	-0.34316200
H	-2.63979200	6.22891600	0.71462200
C	-2.02265200	5.86555700	-1.31861400
H	-0.96137900	5.75573700	-1.06129200
H	-2.22934300	5.15506300	-2.12948800
C	-2.26358300	7.28868100	-1.82565500
H	-2.07048500	8.00063100	-1.01205600
H	-3.32332800	7.40355300	-2.08999800
C	-1.39374500	7.63993500	-3.03126000
H	-1.58538900	8.66068400	-3.37735200
H	-0.32866500	7.56468000	-2.78301600
H	-1.58926300	6.95969200	-3.86840700

3S

0 1

O	-1.59547300	1.10269300	-0.92478400
C	-0.81732800	2.19790500	-1.42450100
C	-1.50410500	3.49640100	-0.97000800
H	0.20215100	2.13044600	-1.02697400
H	-0.78035100	2.09404900	-2.50996900
H	-0.79972800	4.33495000	-0.94525000
H	-2.30419400	3.75734100	-1.67510200
P	-2.10313000	1.38554500	0.60447000
C	-3.82854900	0.84992800	0.58764400
C	-4.74124100	1.37988100	-0.33018600
C	-4.26105500	-0.06304200	1.54946900
C	-6.07688600	0.99952300	-0.28394500
H	-4.40696100	2.09284200	-1.07953000
C	-5.59923900	-0.44831100	1.59009600
H	-3.54272100	-0.46038300	2.26021200
C	-6.50510300	0.08368300	0.67727500
H	-6.78452200	1.41415800	-0.99563700
H	-5.93431000	-1.16077000	2.33778800
H	-7.54924100	-0.21324400	0.71345000

O	-1.32347900	0.78952600	1.71872600
C	-2.60330200	4.03010600	1.28258400
H	-3.00235800	3.61734800	2.20880000
C	-2.08346800	3.18437900	0.39151800
C	-2.78936600	5.50588300	1.06377000
H	-2.84372100	6.02164300	2.02954400
H	-1.93027200	5.92248500	0.52398200
C	-4.07396200	5.78871900	0.25978800
H	-4.18322300	6.87410500	0.13519400
H	-3.96090000	5.36920200	-0.74937400
C	-5.34082100	5.21393200	0.89696900
H	-5.44862200	5.61171600	1.91526100
H	-5.23831900	4.12568000	0.99570200
C	-6.59630500	5.52608700	0.08512500
H	-7.48940200	5.10318800	0.55632500
H	-6.74743900	6.60740500	-0.01353900
H	-6.52142600	5.10740700	-0.92568200

4S

0 1

O	-1.35292400	0.70311400	-0.22112100
C	-0.60509000	1.63217900	-1.01588400
C	-1.54438100	2.78908900	-1.38820900
H	0.25151300	1.99701000	-0.43690700
H	-0.23734900	1.08428100	-1.88541600
H	-0.97884200	3.67815700	-1.68250600
H	-2.17882700	2.49431900	-2.23472400
P	-2.43127800	1.44031000	0.76460900
C	-3.96108500	0.55454600	0.39230000
C	-4.25501600	0.10913900	-0.89973000
C	-4.88543200	0.35962700	1.42097400
C	-5.46624100	-0.52150500	-1.16072900
H	-3.52708600	0.23849100	-1.69531600
C	-6.09689800	-0.27330200	1.15740000
H	-4.63869500	0.69332100	2.42436700
C	-6.38801000	-0.71042200	-0.13221200
H	-5.68994700	-0.87229400	-2.16377900
H	-6.81185000	-0.42883000	1.95969400
H	-7.33311500	-1.20508000	-0.33679900
O	-2.15193900	1.51351800	2.22089000
C	-2.90215700	4.13678700	0.32622700
H	-3.40630000	4.09825800	1.29297000
C	-2.37641700	3.00579000	-0.14722000
C	-2.81937400	5.49413900	-0.30900200

H	-2.50674500	5.41368800	-1.35681900
H	-3.82000000	5.94451700	-0.31018900
C	-1.85176200	6.42274300	0.44674900
H	-1.94669900	7.43754900	0.03923800
H	-2.15350500	6.48098200	1.50155200
C	-0.39190300	5.97494600	0.36076600
H	-0.09082800	5.93765900	-0.69593500
H	-0.30223200	4.95221800	0.74839000
C	0.55287400	6.89774000	1.12823900
H	1.59150800	6.56135300	1.04662100
H	0.50063600	7.92384100	0.74570500
H	0.29293100	6.92485600	2.19255200

5S

0 1

O	-1.39040400	0.81860900	-0.53262300
C	-0.65519100	1.81006000	-1.26029900
C	-1.50276300	3.09126800	-1.28802200
H	0.30521800	1.98686200	-0.76236400
H	-0.46810400	1.40199500	-2.25524400
H	-0.88569200	3.96890400	-1.50329700
H	-2.26373500	3.01434700	-2.07608400
P	-2.22941100	1.45297200	0.72077700
C	-3.88245200	0.77049800	0.46417400
C	-4.41642700	0.60375900	-0.81691400
C	-4.65364100	0.45371800	1.58443900
C	-5.71309800	0.12796700	-0.97487800
H	-3.80990500	0.82850500	-1.68947700
C	-5.95145900	-0.02366700	1.42369200
H	-4.22208400	0.57112800	2.57389500
C	-6.48124600	-0.18350000	0.14602900
H	-6.12383000	-0.00682700	-1.97105400
H	-6.54753000	-0.27459700	2.29591300
H	-7.49354900	-0.55698000	0.02139400
O	-1.73345900	1.24438000	2.10445200
C	-2.56274900	4.20343200	0.77543800
H	-2.97596000	4.02590700	1.76950500
C	-2.15487400	3.14485400	0.07328600
C	-2.54653800	5.63486100	0.33040400
H	-2.00059100	6.22790000	1.07553400
H	-2.01124600	5.74213600	-0.62065900
C	-3.97214200	6.19119000	0.17675500
H	-4.52480200	5.54762200	-0.51889800
H	-4.49195800	6.12238800	1.14237500

C	-4.01933800	7.63794300	-0.32503300
H	-5.06817000	7.91171300	-0.49301600
H	-3.52585000	7.69704300	-1.30464100
C	-3.38473800	8.65068800	0.62884800
H	-3.52358300	9.67323500	0.26310600
H	-3.83758300	8.58677900	1.62545800
H	-2.30756800	8.48660200	0.74069800

6S

0 1

O	-1.27641000	0.63772700	-0.14430800
C	-0.43910800	1.56814200	-0.83928000
C	-1.27422100	2.82324800	-1.13415800
H	0.42719300	1.81312100	-0.21362100
H	-0.09040600	1.06993100	-1.74581000
H	-0.62613500	3.67590900	-1.34205600
H	-1.89720400	2.65080100	-2.02205600
P	-2.30746800	1.37906500	0.88515000
C	-3.89431000	0.63636300	0.44188800
C	-4.21252400	0.32224900	-0.88253300
C	-4.83561600	0.42315100	1.45112000
C	-5.46386300	-0.19701600	-1.19436900
H	-3.47339600	0.46665600	-1.66521900
C	-6.08784100	-0.09758300	1.13658200
H	-4.57081900	0.65409400	2.47855300
C	-6.40235400	-0.40455700	-0.18458500
H	-5.70659500	-0.44651500	-2.22299700
H	-6.81638200	-0.26759000	1.92360100
H	-7.37909300	-0.81195400	-0.42912200
O	-2.04145900	1.32169900	2.34476100
C	-2.73413600	4.07303900	0.63511300
H	-3.28948800	3.88767000	1.55657300
C	-2.14551000	3.00491400	0.08918100
C	-2.80194300	5.51039500	0.19836500
H	-3.86134500	5.72114400	-0.00891100
H	-2.55764500	6.13281200	1.07184400
C	-1.94520800	5.95946700	-0.98479300
H	-0.89048000	5.76284700	-0.75780500
H	-2.19790300	5.36826200	-1.87468900
C	-2.10417400	7.44918400	-1.31235200
H	-1.36971600	7.71392900	-2.08273700
H	-1.84645800	8.04397900	-0.42568000
C	-3.49980600	7.83826200	-1.80214700
H	-3.52794800	8.88997300	-2.10485600

H	-3.79380300	7.23250600	-2.66766000
H	-4.26010000	7.70188800	-1.02575000

7S

0 1

O	-1.46975200	0.82136000	-0.84513000
C	-0.64230300	1.89195200	-1.31811700
C	-1.35059800	3.21064300	-0.96892000
H	0.34008200	1.83228500	-0.83526000
H	-0.51707400	1.74745800	-2.39239500
H	-0.64528000	4.04794000	-0.93278800
H	-2.10030400	3.44213600	-1.73704400
P	-2.12722100	1.16605700	0.61253900
C	-3.85019500	0.66127200	0.41794700
C	-4.60053100	1.05681800	-0.69380800
C	-4.45072300	-0.08442400	1.43282500
C	-5.94276500	0.70936800	-0.78735800
H	-4.13150000	1.63025500	-1.48888200
C	-5.79514700	-0.43530800	1.33492500
H	-3.85486400	-0.38574700	2.28911800
C	-6.53982400	-0.03698500	0.22857400
H	-6.52393400	1.01530400	-1.65209500
H	-6.26061200	-1.01880900	2.12349000
H	-7.58881600	-0.30869300	0.15447600
O	-1.49280200	0.59637700	1.82802500
C	-2.51340600	3.84784100	1.22430200
H	-2.97997600	3.47246300	2.13522000
C	-2.02265600	2.95733700	0.36081100
C	-2.55637500	5.33437500	1.00477500
H	-2.61647300	5.84777500	1.97051900
H	-1.63079900	5.67370200	0.52385500
C	-3.75139200	5.74582700	0.11972800
H	-3.77972000	6.84194800	0.06217200
H	-3.57260600	5.38236800	-0.90029000
C	-5.10938300	5.22046200	0.59844600
H	-5.11453300	4.12391800	0.54366700
H	-5.87891500	5.56567200	-0.10293500
C	-5.48179500	5.66480700	2.01278900
H	-6.49269400	5.33573700	2.27344900
H	-4.79934100	5.24757100	2.76198300
H	-5.44907300	6.75710900	2.10501100

8S

0 1

O	-1.46809700	0.51483600	-0.54044500
C	-0.82443100	1.22685800	-1.60541800
C	-1.79545100	2.31278900	-2.09512500
H	0.10398000	1.67416600	-1.23191700
H	-0.57998500	0.49364600	-2.37614900
H	-1.26682300	3.10142000	-2.63922600
H	-2.53515900	1.86978700	-2.77548900
P	-2.39567800	1.48550000	0.39496300
C	-3.95960400	0.58475700	0.45596000
C	-4.44796300	-0.11285800	-0.65270300
C	-4.71211900	0.63604300	1.63126100
C	-5.68177900	-0.75006100	-0.58638500
H	-3.85360800	-0.17226000	-1.55985500
C	-5.94654500	-0.00420700	1.69552800
H	-4.31468300	1.16682200	2.49108600
C	-6.43173000	-0.69370400	0.58741000
H	-6.05684200	-1.29643500	-1.44661400
H	-6.52774300	0.03207400	2.61197200
H	-7.39462000	-1.19367300	0.63862500
O	-1.90984600	1.88085800	1.74227300
C	-2.89490000	4.03738300	-0.54400800
H	-3.28075200	4.21351800	0.45990300
C	-2.46179800	2.81014100	-0.83561500
C	-2.83271200	5.22961000	-1.45513900
H	-2.91061700	4.90966900	-2.50086800
H	-3.69526700	5.87783500	-1.25672900
C	-1.54326300	6.05892500	-1.28293500
H	-0.68330700	5.45441300	-1.60221100
H	-1.60643100	6.90236200	-1.98133800
C	-1.28805400	6.58882000	0.13831800
H	-2.24446400	6.88024700	0.59446200
H	-0.69960600	7.51141900	0.06302100
C	-0.54234400	5.61953100	1.06076000
H	-0.36162900	6.08013500	2.03786200
H	-1.08338100	4.68426400	1.23030400
H	0.43302100	5.35947000	0.63229200

The absolute configuration of **2a** was confirmed as the *S*-configuration by electronic circular dichroism (ECD) spectra. A comparison between the experimental and calculated ECD spectra of each enantiomer using a time-dependent DFT method was performed for confirming the absolute stereochemistry of a-carbon. The measured ECD spectrum of (*S*)-**2a** showed positive Cotton effects at 225 (Δε+5.5) nm as well as the negative one at 202 (Δε -12.2) nm, which matched with those of the calculated spectra for the S configurations (Figure. 1). Hence, the absolute configuration of **2a** from Rh/(*R*)-**L6** was confirmed as *S*.

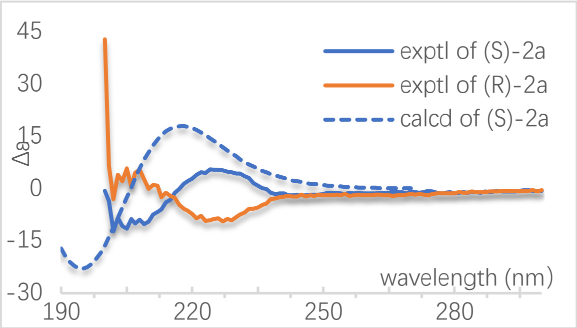

Figure S130. Calculated and experimental ECD spectra of **2a**

13. References

(1) Armarego, W. L. F. Purification of Laboratory Chemicals, 8th ed. Butterworth-Heinemann. Oxford UK. **2017**.

(2) Zhang, Y.-Q.; Funken, N.; Winterscheid, P.; Gansäuer, A. Hydroxy-Directed, Fluoride-Catalyzed Epoxide Hydrosilylation for the Synthesis of 1, 4-Diols. *Angew. Chem. Int. Ed.* **2015,** *54,* 6931–6934.

(3] Montchamp, J.-L.; Dumond, Y. R. Synthesis of monosubstituted phosphinic acids: palladium-catalyzed cross-coupling reactions of anilinium hypophosphite. *J. Am. Chem. Soc.* **2001,** *123*, 510–511.

(4) Belabassi, Y.; Bravo-Altamirano, K.; Montchamp, J.-L. Regiocontrol in the palladium-catalyzed hydrophosphinylation of terminal alkynes. *J. Organomet. Chem.* **2011,** *696*, 106–111.

(5) Gaussian 16, Frisch, M. J.; Trucks, G. W.; Schlegel, H. B.; Scuseria, G. E.; Robb, M. A.; Cheeseman, J. R.;; Scalmani, G.; Barone, V.; Petersson, G. A.; Nakatsuji, H.;; Li, X.; Caricato, M.; Marenich, A. V.; Bloino, J.; Janesko, B. G.; Gomperts, R.; Mennucci, B.; Hratchian, H. P.; Ortiz, J. V.; Izmaylov, A. F.; Sonnenberg, J. L.; Williams, F. Ding, Lipparini, F.; Egidi, F.; Goings, J.; Peng, B.; Petrone, A.; Henderson, T.; Ranasinghe, D.; Zakrzewski, V. G.; Gao, J.; Rega, N.; Zheng, W.; Liang, M.; Hada, M.; Ehara, M.; Toyota, K.; Fukuda, R.; Hasegawa, J.; Ishida, M.; Nakajima, T.; Honda, Y.; Kitao, O.; Nakai, H.; Vreven, T.; Throssell, K.; Montgomery Jr.; J. A.; Peralta, J. E.; Ogliaro, F.; Bearpark, M. J.; Heyd, J. J.; Brothers, E. N.; Kudin, K. N.; Staroverov, V. N.; Keith, T. A.; Kobayashi, R.; Normand, J.; Raghavachari, K.; Rendell, A. P.; Burant, J. C.; Iyengar, S. S.; Tomasi, J.; Cossi, M.; Millam, J. M.; Klene, M.; Adamo, C.; Cammi, R.; Ochterski, J. W.; Martin, R. L.; Morokuma, K.; Farkas, O.; Foresman, J. B.; Fox, D. J. Gaussian, Inc. Wallingford CT. **2016**.

(6) Chai, J.-D.; Head-Gordon, M. Long-range corrected hybrid density functionals with damped atom-atom dispersion corrections, *Phys. Chem. Chem. Phys.* **2008,** *10*, 6615–6620.

(7) Marenich, A. V.; Cramer, C. J.; Truhlar, D. G. Universal Solvation Model Based on Solute Electron Density and on a Continuum Model of the Solvent Defined by the Bulk Dielectric Constant and Atomic Surface Tensions. *J. Phys. Chem. B.* **2009,** *113*, 6378.

(8) Lu, T.; Chen, F. W. Multiwfn: A multifunctional wavefunction analyzer. *J. Comput. Chem.* **2012,** *33*, 580.
